# Exome sequencing identifies frequent mutation of MLL2 in non–small cell lung carcinoma from Chinese patients

**DOI:** 10.1038/srep06036

**Published:** 2014-08-12

**Authors:** Shanye Yin, Jing Yang, Bin Lin, Wenjun Deng, Yuchao Zhang, Xianfu Yi, Yufang Shi, Yong Tao, Jun Cai, Chung-I Wu, Guoping Zhao, Laurence D. Hurst, Jie Zhang, Landian Hu, Xiangyin Kong

**Affiliations:** 1 State Key Laboratory of Medical Genomics, Institute of Health Sciences, Shanghai Jiao Tong University School of Medicine and Shanghai Institutes for Biological Sciences, Chinese Academy of Sciences, Shanghai 200025, People's Republic of China; 2 CAS Key Laboratory of Genome Sciences and Information, Beijing Institute of Genomics, Chinese Academy of Sciences, Beijing 100029, People's Republic of China; 3 Shanghai-MOST Laboratory of Disease and Health Genomics, Chinese National Human Genome Center at Shanghai, Shanghai 201203, People's Republic of China; 4 Department of Biology and Biochemistry, University of Bath, Claverton Down, Bath, BA2 7AY, UK; 5 Department of pathology, Shanghai Chest Hospital, Shanghai 200025, People's Republic of China; 6 These authors contributed equally to this work.

## Abstract

Lung cancer is the most common cause of cancer mortality worldwide, with an estimated 1.4 million deaths each year. Here we report whole-exome sequencing of nine tumor/normal tissue pairs from Chinese patients with non-small cell lung carcinoma (NSCLC). This allows us to identify a number of significantly mutated genes in NSCLC, which were highly enriched in DNA damage repair, NF-κB pathway, JAK/STAT signaling and chromatin modification. Notably, we identify a histone-lysine methyltransferase gene, namely, MLL2, as one of the most significantly mutated genes in our screen. In a following validation study, we identify deleterious mutations of MLL2 in 12 out of 105 (11.4%) NSCLC patients. Additionally, reduced or lost expression of MLL2 was commonly observed in tumor tissues as compared with paired adjacent non-tumor tissues regardless of mutation status. Together, our study defines the landscape of somatic mutations in Chinese NSCLC and supports the role of MLL2 mutation in the pathogenesis of the disease.

Lung cancer is the most commonly diagnosed cancer as well as the leading cause of cancer deaths worldwide. It has been classified into two major types, including NSCLC and small cell lung cancer (SCLC)^1^. About 85 percent of all lung cancers are identified as NSCLC, which comprises three histological subtypes: adenocarcinoma (ADC), squamous cell carcinoma (SCC) and large cell lung carcinomas (LCC)^1^,^2^. Although great efforts have been made to develop new diagnostic and therapeutic approaches for lung cancer, however, the prognosis remains poor due to diagnosis at an advanced stage and a high rate of recurrence^1^.

Cancer is a highly heterogeneous disease associated with various environmental exposures and genetic susceptibility which leads to unrestrained cell proliferation and neoplasm formation. The accumulation of somatic DNA alterations is considered as one of the contributing factors in carcinogenesis^2^,^3^,^4^. These alterations can occur at different levels from point mutations to gain or loss of large DNA fragments, resulting in altered expression of the corresponding genes or impaired protein functions. Over the last few years, extensive research has investigated the genetic alterations associated with lung cancer^2^,^5^,^6^,^7^,^8^,^9^. These efforts have led to the discovery of several reproducible alterations underlying disease and have already proven successful to facilitate clinical decision making in NSCLC^1^,^10^,^11^. For example, genetic abnormality of the tumor suppressor gene TP53 is commonly observed in patients and has been shown to be associated with poorer survival prognosis and increased cellular resistance to therapy^1^,^5^. Activating EGFR mutations, observed in about 15% NSCLC patients, are associated with increased sensitivity to EGFR tyrosine kinase inhibitors. Consequently, EGFR has become a target for anti-cancer drug therapy while evaluation for EGFR mutation status is used as a standard of care in advanced-stage NSCLC^8^,^12^. KRAS plays an important role in regulating several signaling pathways downstream from EGFR and are also found to be frequently mutated in NSCLC^13^. In line with this, KRAS mutations and EGFR mutations are found to be mutually exclusive in larger series of patients. Additionally, recent large-scale, whole genome sequencing studies have identified frequent mutations in tumor suppressors such as NF1, RB1, NF1, CDKN2A, SMARCA4 and KEAP1^5^,^6^,^7^,^14^,^15^. Activating mutations in BRAF, ERBB4, and PIK3CA which are associated with tumor cell growth and survival are also found to be common^16^,^17^,^18^. Moreover, recurrent somatic mutations in the splicing factor gene U2AF1, chromatin remodeling gene ARID1 and matrix remodeling protein MXRA5 have also been reported^9^,^19^,^20^. These findings have largely expanded our understanding of the disease mechanisms, providing useful information on novel therapeutic targets and predictive biomarkers.

Here we used massively parallel sequencing to sequence the exomes of nine NSCLCs and matched normal adjacent tissue pairs. This allows us to identify a number of known and previously unreported genes that are significantly mutated in these patients. Pathway analysis and network reconstruction revealed several key signaling cascades affected in NSCLC including cell-cycle progressing, JAK/STAT signaling and chromatin remodeling. These results provide new insights into the molecular pathogenesis of NSCLC in Chinese patients.

## Results

### Overview of genetic alterations

In this study, we performed whole-exome sequencing of paired tumour/control DNA from nine Chinese patients with NSCLC, including eight ADCs and one SCC, with a mean depth of 104× and 93.8% of bases covered to at least 20× (Table 1, Supplementary Table 1). Altogether we identified 3618 high-confidence somatic mutations, of which 1556 (43%) were in protein coding regions. These NSCLCs display a large variation in the number of somatic mutations (Supplementary table 2), with a mean of 132 nonsynonymous and 40 synonymous mutations in protein-coding sequences as well as 229 mutations in introns or intergenic regions (Table 1, Supplementary Tables 2, 3). In line with previous results^5^,^6^,^9^,^15^,^17^, we found that tumors from smokers (smk) have significantly higher number of exonic mutations compared to nonsmokers (nonsmk) (324 ± 119 per smk *vs* 52 ± 16 per nonsmk, *p* = 0.001, Student's t-test). Consistent with previous studies^9^,^16^,^17^, we observed that mutations occurred predominantly at G:C base pairs (69.1%), with G:C > A:T (14.7%), C:G > T:A (14.2%), C:G > A:T (12.2%) and G:C > T:A (11.9%) to be the most commonly observed transversions.

We then selected a subset of nonsynonymous mutations from genes that are mutated in multiple patients for further validation. Using Sequenom MassARRAY genotyping, we validated 101 out of the 108 (93.5%) nonsynonymous mutations examined (Supplementary Table 3), indicating a very low false-positive rate of our results. Possible impact of all nonsynonymous substitutions were predicted based on functional prediction algorithms PolyPhen and Grantham, as well as conservation prediction algorithms PhastCons and GERP. Altogether, 533 out of 1190 (44.8%) nonsynonymous mutations were predicted to be highly deleterious as supported by three or four algorithms (Supplementary Table 4).

### Frequently mutated genes in NSCLC

This systematic approach allowed us to identify several well-described target genes as well as a number of previously unreported genes that are recurrently mutated in our NSCLC cohort. Notably, we found nonsynonymous mutations in 62 census cancer genes as reported in Catalogue of Somatic Mutations in Cancer (COSMIC) database, indicating that they were highly mutated in NSCLC and causally linked to the pathogenesis of the disease. This included many known genes associated with lung cancer, such as TP53, EGFR, KRAS and ERBB4. Indeed, TP53 was the second most frequently mutated gene identified in this study, with two nonsense and three missense mutations in five tumor samples (56%). A missense mutation and a mutation in 5′-UTR region of EGFR were identified in two different samples. We also found one missense mutation of KRAS and one missense mutation of ERBB4 in different patients. TTN is the most frequently mutated proteins in our study which contains 13 missense mutations in 6 out of 9 samples (67%). This is consistent with results from COSMIC that 244 out of the 467 (52%) lung cancer samples sequenced have somatic mutations in TTN. TTN encodes a very large, high abundant protein of striated muscle. However, considering the large size of the protein and prevalence of TTN mutations identified in other studies unrelated to cancer, it is presently hard to determine whether mutations in TTN act as drivers or are only passengers in NSCLC^6^.

We then used MuSiC algorithm^21^ to identify potential drivers that have a significant higher mutation rate than background mutation rates with three independent tests, including FCPT (Fisher's combined P-value test), LRT (Likelihood ratio test) and CT (Convolution test) (see method). Altogether we identified nine genes that are potential cancer drivers supported by all three tests (Table 2, p < 0.05). Benchmarking the results with TTN gene, we found that TTN is much less likely to be a cancer driver as predicted by LRT than the other two methods (Supplementary Table 5). Based on LRT method, TP53 was identified as most significantly mutated gene, followed by MLL2, which had three missense and two nonsense mutations in three out of nine samples (33%). MLL2 is a member of the myeloid/lymphoid or mixed-lineage leukemia (MLL) gene family, encodes a histone lysine methyltransferase that play an important role in epigenetic regulation of gene expression^22^,^23^. Recent studies showed that MLL2-inactivating mutations were frequently observed in several cancers^19^,^24^,^25^,^26^,^27^, establishing it as a novel tumor suppressor. Frequent mutation of MLL2 was also observed in COSMIC lung cancer cohort (64 out of 431, 15%), we thus suggest that MLL2 might be a novel cancer gene associated with NSCLC^22^,^27^. Additionally, we found that NEK1, a serine/threonine kinase involved in cell cycle regulation, was mutated more frequently than expected by chance. Other significantly mutated proteins include PAPPA2, which encodes a member of the pappalysin family of metzincin metalloproteinases and plays an important role in insulin-like growth factor signaling pathway. CDH10 encodes a cadherin superfamily, integral membrane protein that mediates calcium-dependent cell-cell adhesion and might play a significant role in cancer progression and metastasis.

In addition to mutations, we also identified small insertions/deletions (indels) as well as copy number variations (CNVs) in these nine patients using different computational approaches (see methods). All together we identified 93 high confident indels in these nine samples (supplementary Table 6). We found indel mutations in several key regulators of cell death including NOTCH2, CASPA8AP2, CUL2, PRUNE2 and RPS27L, as well as those involved in Ras signaling, including GMIP, ECT2L and ERBB2. We also detected CNV events in 2870 genes, of which, 301 contains deleterious mutations and eight contains indels (supplementary Table 7). For example, we observed copy number loss in a number of cell cycle regulators including CKS2, NEK1 and LATS2. However, compared with detecting mutations and indels, extracting information of CNVs from exome sequencing data is very challenging as the results are highly affected by sequencing depth and the noncontiguous nature of the captured exons. We thus suggest that the CNV results reported here should be interpreted with care.

### Altered pathways and gene modules in NSCLC

**Figure 1:**
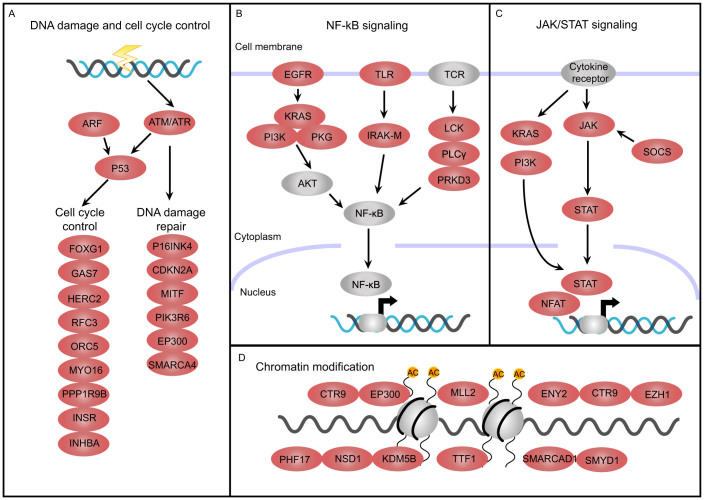
Core Signaling Pathways in NSCLC. Mutated genes are significantly enriched in several important signaling pathways including: (A) DNA damage and cell cycle control; (B) NF-kB signaling; (C) JAK/STAT signaling and (D) Chromatin modification. Genes with deleterious mutations are shown in red, while other key players of the signaling pathway were shown in grey.

Although it seems that the mutations are tremendously diverse and complex, however, they might disturb genes involved in the same signaling pathway or regulatory module^6^,^28^,^29^,^30^. We thus performed pathway analysis to identify signaling cascades that contain a larger number of mutated genes than expected by chance (Supplementary table 8). Interestingly, mutated genes were significantly enrichment in DNA damage checkpoint and cell cycle control (Figure 1A). In response to DNA damage, eukaryotic cells activate ATM/ATR and TP53 to initiating signaling cascade involved in cell cycle arrest and DNA repair. Multiple components of the signaling pathway were mutated in six out of the nine NSCLC cases. Moreover, we also observed significant enrichment of mutated genes in NF-kB signaling, which controls the expression of many genes involved in cell growth and immune responses. Although no somatic mutation was detected in any components of NF-kB complex, we found frequent mutations in a wide range of membrane receptors and their downstream effectors that might result in perturbed NF-kB function in six cases. As shown in Figure 1B, some of the key cascades involved EGFR/KRAS pathway, TLR/IRAK-M pathway and TCR/LCK pathway. Interestingly, in line with previous study^5^, we found that JAK-STAT pathway was also significantly disrupted in NSCLC. JAK-STAT pathway is one of the important signaling pathways downstream of cytokine receptors, which functions in plays an important role in regulating cell proliferation and migration. Somatic mutations in several key components of the pathway, including JAK, STAT and SOCS (Figure 1C), were identified in four cases. Moreover, there is growing evidence suggesting that aberrant epigenetic modifications are playing an important role in the tumorigenesis. Recurrent mutations of chromatin-modifying genes have been recently discovered in other cancers^19^,^24^,^25^,^27^,^31^ but their implication in lung cancer had not yet been reported. Here we found frequent mutations in several modifiers in six tumors, including some of the most frequently mutated gene MLL2 (Figure 1D). As epigenetic aberrations are potentially reversible, targeting abnormal chromatin-modifiers might be a promising strategy for the treatment of cancer. Other core signaling pathways that were mutated in at least six patients included the Axonal Guidance Signaling, Hereditary Breast Cancer Signaling, PTEN Signaling and p70S6K Signaling, many of which have been implicated in other cancers (Supplementary Table 8).

We then mapped mutant genes into a global signaling network using cytoscape^32^,^33^ (Supplementary Figure 1A). To enhance quality of the analysis and to reduce network complicity, only physical interactions and regulatory interactions were used for network construction. Using GeneMANIA^33^, we also identified several tightly connected modules that contains a significantly higher number of genes involved in the same signaling pathway. Interestingly, we find that the most significantly affected module is involved in Tyrosin kinase activity of JAK-STAT cascade (Supplementary Figure 1B). This is in line with our pathway analysis and supports the roles of alternations in JAK-STAT module as driver mutations in NSCLC. Moreover, we also identified a closely connected sub-network that plays an important role in G2/M transition (Supplementary Figure 1C).

### Validation study of MLL2 mutations in NSCLC

**Figure 2:**
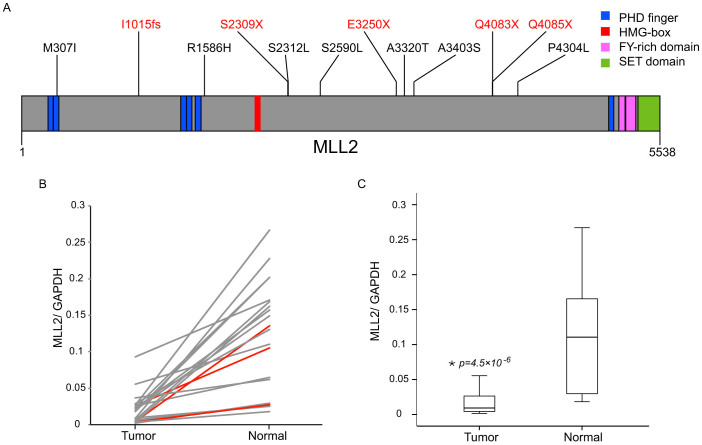
Somatic Mutations and gene expression of MLL2 in NSCLC. (A) Schematic representation of somatic mutations identified in MLL2 shown in the context of the known domain structures. Numbers refer to amino acid residues. Frame-shift and nonsense mutations are shown in red; other missense mutations are shown in black. (B) Pair-wise comparisons of MLL2 expression in NSCLC tumors and adjacent normal tissues. Relative abundance of MLL2 was measured based on the ratio between fluorescence emission intensity values between MLL2 and GAPDH in the same sample obtained by quantitative real-time PCR. Patients with loss-of-function mutations in MLL2 are in red while others are in grey. (C) The distribution of MLL2 expression levels between tumor and normal tissues.

The frequent mutations of genes involved in chromatin modification and the discovery of MLL2 as one of the most frequently mutated genes in NSCLC lead us to study this gene in greater detail. We then resequenced the coding regions of MLL2 in the discovery cohort as well as an additional 96 NSCLC samples by targeted sequencing. The two cohorts together encompass 105 NSCLC cancers, including 81 ADC and 24 SCC. Specifically, all exons of MLL2 in each tumor tissue were amplified using long-range PCR, followed by barcoded sequencing on the Illumina Genome Analyzer with an average coverage of ~1000× per sample. After removing known mutations in dbSNP and 1000 Genomes, we resequenced all nonsynonmous mutations in tumor/normal pairs to screen for novel somatic mutations using Sequenom followed by Sanger sequencing. All together we identified inactivating mutations or protein deficiency of MLL2 in 12 out of 105 (11.4%) individuals (Figure 2A, Table 3), which was similar to that reported in COSMIC database (64/431, 15%), indicating that MLL2 is frequently mutated in NSCLC.

We then used quantitative real-time PCR to study MLL2 expression in 23 NSCLC samples with good RNA quality, including the nine samples in the discovery cohort. Somatic mutations of MLL2 were identified only in three tumors in the discovery cohort. Interestingly, we found that MLL2 expression was either silenced or significantly reduced in all tumor tissues compared with adjacent non-tumor lung tissues (Figure 2B, C, p = 4.6 × 10^−6^, Student's t-test), regardless of the mutation status. All together, we found that MLL2 was frequently mutated and repressed in NSCLC, supporting its role as a critical tumor suppressor.

## Discussion

Our understanding of the genetic variations that underlie the pathogenesis of NSCLC has taken great strides during the last few years^5^,^9^,^15^,^16^,^18^. These findings will continue to reshape the landscape of clinical care by providing useful information regarding diagnosis, prognosis, targeted therapy and clinical trials. Here we performed exom sequencing of NSCLCs in Chinese patients. This allowed us to identify a number of previously unreported mutated genes and pathway alterations in NSCLC, providing evidence of common dysfunction in cell cycle control, NF-kB signaling and chromatin modification.

In addition to key players in lung cancer such as TP53 and EGFR, we had also found frequent mutations in several potential cancer genes yet to be associated with lung cancer in our initial screen. Among them MLL2 was the most striking novel candidate in NSCLC. MLL2 is a histone lysine methyltransferase that forms a complex with other co-factors including ASH2L, RbBP5, WDR5, DPY30, PA1 and UTX, which has been shown to play essential roles in epigenetic regulation of transcription^22^,^34^. A recent combinatorial analysis of the MLL2 binding profile and gene expression profile in MLL2 knockout cells identified several direct targets of MLL2^22^. Notably, their results revealed that MLL2 binding sites were highly overlapped with p53-targeted regions^22^ and a wide range of genes involved in p53 pathway, cAMP-mediated signaling, and cholestasis signaling were significantly downregulated in MLL2 knockout cells^22^. Moreover, recent genetics studies also found mutations in MLL2 or other genes encoding subunits of MLL2 complex to be common in a variety of cancer types^19^,^22^,^24^,^25^,^27^,^31^, supporting its role as a tumor suppressor. MLL2 protein contains several evolutionarily conserved domains including clusters of PHD (plant homeodomain) domain at the N-terminus, and a SET (suppressor of variegation, enhancer of zeste, trithorax) domain at the C-terminus, which is essential for the DNA binding ability of MLL2. In our validation screen for MLL2 somatic mutation, we identified a high proportion of nonsense mutations which result in truncated proteins of MLL2 lacking the SET domain (Figure 2A).

Interestingly, we observed that MLL2 expression was significantly repressed in tumor tissues compared with adjacent normal tissues. We found no evidence that reduced expression of MLL2 is caused by mutations, as MLL2 expression is also reduced in tumor samples bearing no MLL2 mutation. We also found no indels, CNVs or other structural variations of MLL2 in our exome sequencing data (Supplementary Table 6–7). This suggest that MLL2 expression in tumors might be caused by reduced transcription or RNA stability. Whichever the cause, altered expression of MLL2 may abolish MLL2 mediated histone methylation activities and disrupt expression of downstream target genes. We then asked whether mutation and low expression of MLL2 are prognostic for poor lung cancer survival. Using UCSC cancer browser database^35^,^36^, we obtained MLL2 mutation spectrum, expression profile and clinical information of a cohort of 167 TCGA lung squamous cell carcinoma (LUSC) patients. Interestingly, we found that patients bearing MLL2 mutations are more likely to have worse survival outcome than those with normal MLL2 (Supplementary Figure 1A, Log Rank p = 0.07), supporting the role of mutant MLL2 in driving tumor growth and progression. In contrast, no significant difference was observed between patients with relative high or low MLL2 expression in tumors samples (Supplementary Figure 1B, Log Rank p = 0.53). However, we might not be able to draw a substantive conclusion at this stage as the number of cases with MLL2 mutation was small (n = 33). Future large scale sequencing or functional studies are required to investigate the role of MLL2 in NSCLC.

Personalized medicine with targeted therapy is a major improvement over conventional chemotherapy^1^,^3^,^11^,^37^. For example, targeted therapy for EGFR has proven successful in treatment of patients with sensitive mutations and is now considered to be the standard of care in advanced-stage NSCLC^1^,^8^,^12^. Cancer genome sequencing is thus essential to selecting appropriate therapy strategies and finding novel therapeutic targets. In addition to EGFR, we had identified several mutated genes that were potential therapeutic targets for the treatment of NSCLC^38^, including ABL2, BCL11A, FLT3, JAK2, KIT, MMP3, MTOR, PARG, PI3K, RAD51, AP1, RXRA and TLR4. Comprehensive identification of therapeutic targets and incorporation of molecular testing in clinical decision making will hold a key to improving NSCLC patients' outcomes in the future.

## Methods

### Tumor tissues

105 pairs of fresh frozen NSCLC tumors and matched adjacent normal lung tissues, including nine pairs used for exome sequencing, were obtained from the Pathology Core at Shanghai Chest hostpital affilitated to Shanghai Jiaotong University. The clinical information associated with the NSCLC samples used in this study is provided in Supplementary Table 7. Primary tumor tissues were sectioned, stained, and reviewed by pathologists before they were micro-dissected to obtain a high purity of the tumor cells (>90%). The study protocol was approved by institutional review board of Shanghai Chest hospital and Shanghai Institutes for Biological Sciences. Informed consent was obtained from all subjects and all experiments were performed in accordance with relevant guidelines.

### NimbleGen exome capture and sequencing

The qualified genomic DNA sample was randomly fragmented by Covaris and the size of the library fragments is mainly distributed between 250 bp and 300 bp. Then adapters were ligated to both ends of the resulting fragments. Extracted DNA was then amplified by ligation-mediated PCR (LM-PCR), purified, and hybridized to the NimbleGen 44 M human exome array for enrichment. Each captured library was then loaded on Hiseq2000 platform, and we performed high-throughput sequencing for each captured library. Each sample was sequenced at the mean depth of 100× to achieve high sensitivity and accuracy for mutations detection. Raw image files were processed by Illumina basecalling Software 1.7 for base-calling with default parameters and the sequences of each individual were generated as 90 bp pair-end reads.

### SNPs and CNVs detection

SOAPaligner^39^(soap2.21) was used to align the clean reads to the human reference genome (NCBI human genome assembly build 37). Based on results from SOAPaligner, software SOAPsnp^21^ was used to assemble the consensus sequence and call genotypes in target regions. Small indels were detected using SOAPindel while CNVs were detected using CEQer^40^. To minimize false-positives in somatic mutations, we set the minimum snp quality is equal or larger than 80, minimum coverage as 8× in normal and 15× in tumor, the minimum reads of variant allele as 5, and the minimum proportion of variant allele as 15% in tumor. We also filtered mutations reported in HapMap, dbSNP 137 and1000 genomes. Then we used Seattle Seq Annotation to functionally annotate and categorize somatic variants detected from diverse genomes. Functional importance of the identified mutations were predicted based on 1) polyPhen, which predicts the impact of an amino acid substitution on the structure and function of the protein; 2) Grantham, which predicts the impact by chemical dissimilarity; 3) PhastCons and 4) GERP which both predicted conservation of the mutant site by multiple alignments.We considered nonsynonmous mutations located within a conserved site with strong influence on protein function to be highly deleterious if they passed at least three out of the four filters (polyPhenScore > 0.9, granthamScore > 100, scorePhastCons > 0.9 and consScoreGERP > 3). SeattleSeq Annotation (http://snp.gs.washington.edu/SeattleSeqAnnotation137/).

### Significnatly mutated genes

We use MuSic (the Mutational Significance in Cancer 0.4) to identify significantly mutated genes which have a observable higher mutation rate than background mutation rates. Several basic elements were inputted into MuSiC, including the TCGA MAF format files of somatic mutations of 9 lung cancer patients, the BAM files for the 9 lung cancer samples and their matched normal, and the target exons regions coordinated with the MAF files above (hg19). We applied SMG (the significantly mutated gene) test, a module in MuSiC, to compare each category of nonsynonymous mutation rates to the appropriate BMR. The probability that a gene contains significantly more mutations was determined by three independent test including FCPT, LRT and CT integrated as part of the algorithm.

### Confirmation of somatic mutations using Sequenom MassARRAY

Subsets of nonsynonymous mutations from genes that are mutated in multiple patients identified in our initial screen were selected for validation using Sequenom MassARRAY platform. Genotyping of each tumor/normal pair involved an initial locus-specific PCR reaction, followed by single base extension using mass-modified dideoxynucleotide terminators of a primer which anneals immediately upstream of the potential polymorphic site. PCR primers and extension primers were designed by MassARRAY Assay Design 3.1 software with the default parameters. The extension products were nanodispensed to SpectroCHIP II-G384 chips and analyzed by MassARRAY Analyzer and MassARRAY Typer 4.0 software.

### Pathway analysis and network reconstruction

Ingenuity Pathway Analysis (IPA) was performed to identify signaling pathways that contain higher number of mutant genes than expected by chance. Network reconstruction and Gene ontology based module search was performed with GeneMANIA^33^ in cytoscape^32^. Protein pairwise relationships extracted only from direct protein-protein interactions and curated pathways. For each node the top 20 related genes and at most 20 attributes were used using automatic weighting.

### Targeted sequencing of MLL2

We used 15 long-range PCR to amplify the coding regions of the MLL2 gene in 105 NSCLC tumor samples (primers in Supplementary Table 8). Amplicons of each sample were mixed into 4 pools and used 4 barcode-tagged primer pairs to prepare libraries for paired-end sequencing analysis on an Illumina Genome Analyzer II. Altogether, ~5.5 G base pairs of raw data and ~3.6 million pairs of reads were produced with an average coverage of ~1000× in each sample. After removing known mutations in dbSNP and 1000 Genomes, we resequenced all nonsynonmous mutations in tumor/normal pairs in each pool to screen for novel somatic mutations using Sequenom. All somatic mutations reported in Figure 2A were subsequently confirmed by Sanger sequencing.

### Quantitative real-time PCR and pathway analysis

Quantitative real-time PCR was performed in 23 paired tumor and adjacent non-tumor tissues using the ABI PRISM 7900 System according to the manufacturer's protocol. MLL2 was amplified using M-F: 5′-GGAGCTGCCACTCATGATCA-3′ and M-R: 5′-TGTTGGTCTCGCCTGTGAAG-3′. Housekeeping gene GAPDH (Glyceraldehyde 3-phosphate dehydrogenase) was used as reference and amplified using G-F: 5′- AAGGTGAAGGTCGGAGTCAA-3′ and G-R: 5′-AATGAAGGGGTCATTGATGG-3′. Student's t-test was used to compare levels of expression between tumor and adjacent non-tumor tissues.

## Supplementary Material

Supplementary Information Supplementary Information

## Figures and Tables

**Table 1:** Summary metrics of exome sequencing

Sequence reads and coverages	Mean
Reads mapped to genome:	99066407.83
Reads mapped to exons:	63947604.22
Data mapped to exons (Mb):	4606.81
Mean depth of exons:	104.24
Coverage of exons (%):	99.57
Average read length (bp):	89.98
Rate of nucleotide mismatch (%):	0.3
Fraction of exon covered > = 10X:	97.15
Fraction of exon covered > = 20X:	93.82
Fraction of exon covered > = 30X:	89.1
Fraction of exon covered > = 40X:	82.82
Fraction of exon covered > = 50X:	75.66

Summary of somatic mutations	Mean
Intergenic	21.22
Intron	207.89
Coding-synonymous	39.67
Missense	103
Stop-gained	7.56
Stop-lost	0.44
Splice site	4
3′-UTR	11.33
5′-UTR	5.89

**Table 2:** Genes with somatic mutation rates significantly higher than background

Gene Symbol	NO. of Mutations	Gene Length	P value-FCPT	P value-LRT	P value-CT
**TP53**	5	2261	1.68E-05	4.96E-08	1.14E-08
**MLL2**	5	18074	2.66E-03	2.57E-06	1.76E-05
**NEK1**	2	4366	2.92E-02	6.17E-06	1.12E-04
**OR52A1**	2	942	7.72E-03	9.85E-06	4.23E-06
**IGSF11**	3	2739	1.50E-02	3.66E-05	3.76E-05
**BBS7**	2	2395	3.59E-02	1.08E-04	5.60E-05
**PAPPA2**	3	7061	4.19E-02	1.79E-04	2.51E-04
**CDH10**	3	4036	2.46E-02	5.53E-04	9.03E-05
**TTN**	13	138148	1.21E-03	6.61E-04	4.48E-05

**Table 3:** Mll2 mutations detected in exome sequencing and screening samples

Position	Ref	Var	Type	Function	Change
**chr12:49447023**	C	C/T	ADC	missense	M307I
**chr12:49444329**	G	A/G	SCC	frameshift	I1015fs
**chr12:49438733**	C	C/T	ADC	missense	R1586H
**chr12:49434627**	G	G/T	ADC	stop-gained	S2309X
**chr12:49434618**	G	A/G	ADC	missense	S2312L
**chr12:49433784**	G	A/G	ADC	missense	S2590L
**chr12:49431391**	C	A/C	ADC	stop-gained	E3250X
**chr12:49431181**	C	C/T	SCC	missense	A3320T
**chr12:49430932**	C	A/C	ADC	missense	A3403S
**chr12:49426241**	G	A/G	ADC	stop-gained	Q4083X
**chr12:49426235**	G	A/G	ADC	stop-gained	Q4085X
**chr12:49425577**	G	A/G	ADC	missense	P4304L
