# Supplementary material for: Exome sequencing identifies frequent mutation of MLL2 in non–small cell lung carcinoma from Chinese patients
Source: Sci Rep. 2014 Aug 12;4:6036. doi: 10.1038/srep06036 (PMC5381403; doi:10.1038/srep06036)
Supplement: Supplementary Information [file srep06036-s1.pdf]

Supplementary Information

Exome sequencing identifies frequent mutation of MLL2 in non–small cell lung carcinoma from Chinese patients

Shanye Yin[1*], Jing Yang[1*], Bin Lin[1*], Wenjun Deng[1*], Yuchao Zhang[1], Xianfu Yi[1], Yufang Shi[1], Yong Tao[2], Jun Cai[2], Chung-I Wu[2], Guoping Zhao[3], Laurence D. Hurst[4], Jie Zhang[5], Landian Hu[1#], Xiangyin Kong[1#]

[1]State Key Laboratory of Medical Genomics, Institute of Health Sciences, Shanghai Jiao Tong University School of Medicine and Shanghai Institutes for Biological Sciences, Chinese Academy of Sciences, Shanghai 200025, People's Republic of China.

[2]CAS Key Laboratory of Genome Sciences and Information, Beijing Institute of Genomics, Chinese Academy of Sciences, Beijing 100029, People's Republic of China.

[3]Shanghai-MOST Laboratory of Disease and Health Genomics, Chinese National Human Genome Center at Shanghai, Shanghai 201203, People's Republic of China.

[4]Department of Biology and Biochemistry, University of Bath, Claverton Down, Bath, BA2 7AY, UK.

[5]Department of pathology, Shanghai Chest Hospital, Shanghai 200025, People's Republic of China.

[*]These authors contributed equally to this work.

[#]Corresponding authors:

Landian Hu Email: ldhu@sibs.ac.cn Fax:+86-21-64678976 Tel: +86-21-63852639 or **Xiangyin Kong** Email: xykong@sibs.ac.cn Fax:+86-21-64678976 Tel:+86-21-54920605

Index of all supplementary figures and tables

Supplementary Figures

Supplementary Figure 1.Signaling network of mutant genes. (A) Mapping mutant genes identified in our exome sequencing study onto a gene interaction network. Only genes contains physical interaction or regulatory interaction are shown. (B) Gene module of JAK/STAT induced Tyrosin kinase activity. (C) Gene module of G2/M cell cycle transition. Red line indicates physical interaction while blue line indicates that two proteins are involved in the same signaling pathway.

Supplementary Figure 2. Relationship between alternations in MLL2 gene and survival outcomes. (A) Kaplan-Meier Plot of the survival outcomes of patients with MLL2 mutation (red) and normal MLL2 (black) over time. Percent survival is on the Y-axis and time is on the X- (yellow) and low MLL2 expression (black). MLL2 expression higher or lower than the median value is considered high or low, separately.

Supplementary Tables

Supplementary Table 1. Summary of data quality in each sample.

Supplementary Table 2. Number of somatic mutations in each sample.

Supplementary Table 3. All high-confident somatic mutations.

Supplementary Table 4. All highly deleterious mutations.

Supplementary Table 5. Significantly mutated genes.

Supplementary Table 6. Insertion and deletion.

Supplementary Table 7. Copy number variations.

Supplementary Table 8. Signaling pathways with significant enrichment of mutated genes.

Supplementary Table 9. Clinical information of NSCLC patients.

Supplementary Table 10. Primers to amplify exons of MLL2.

Supplementary Figure 1

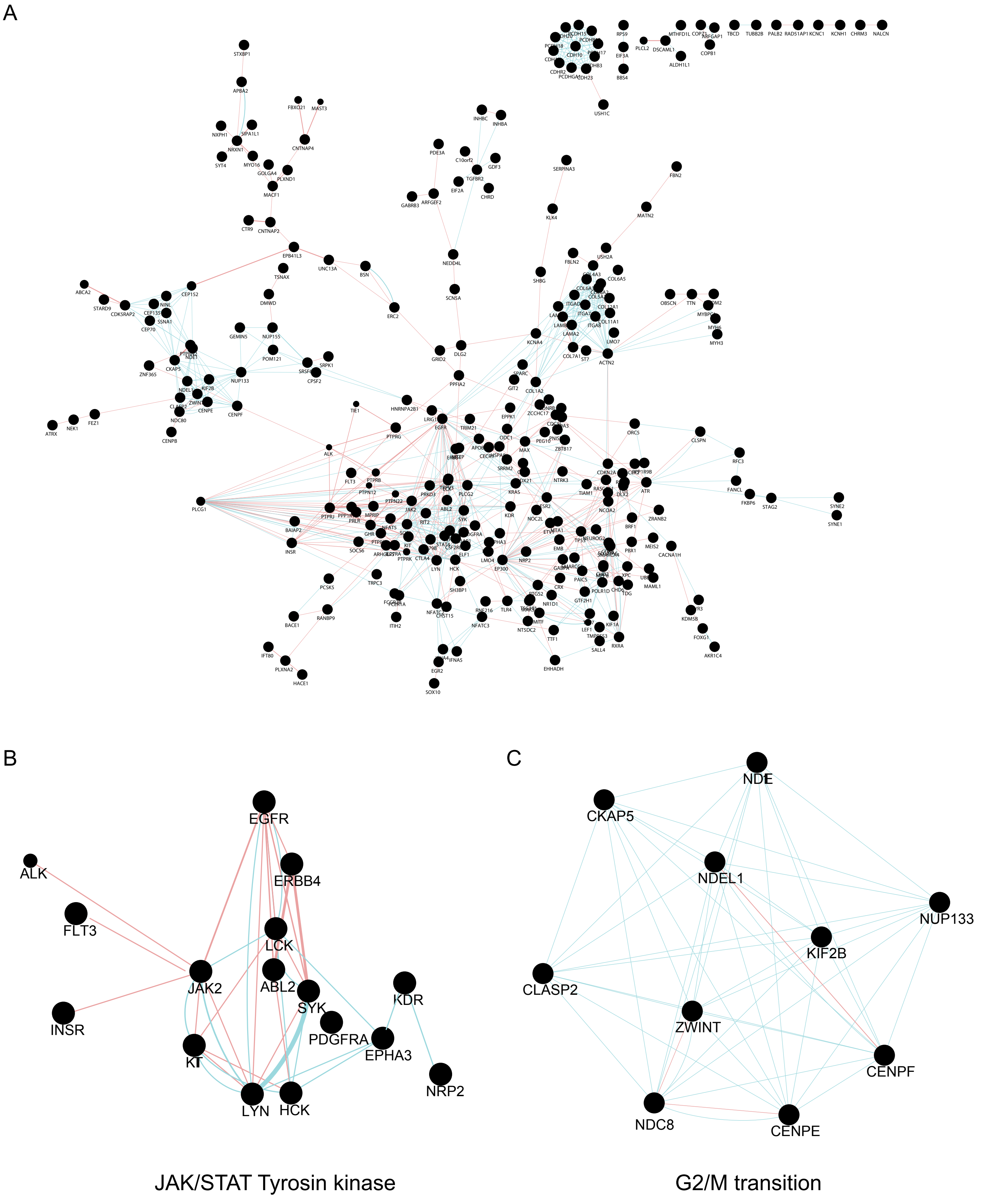

Supplementary Figure 2

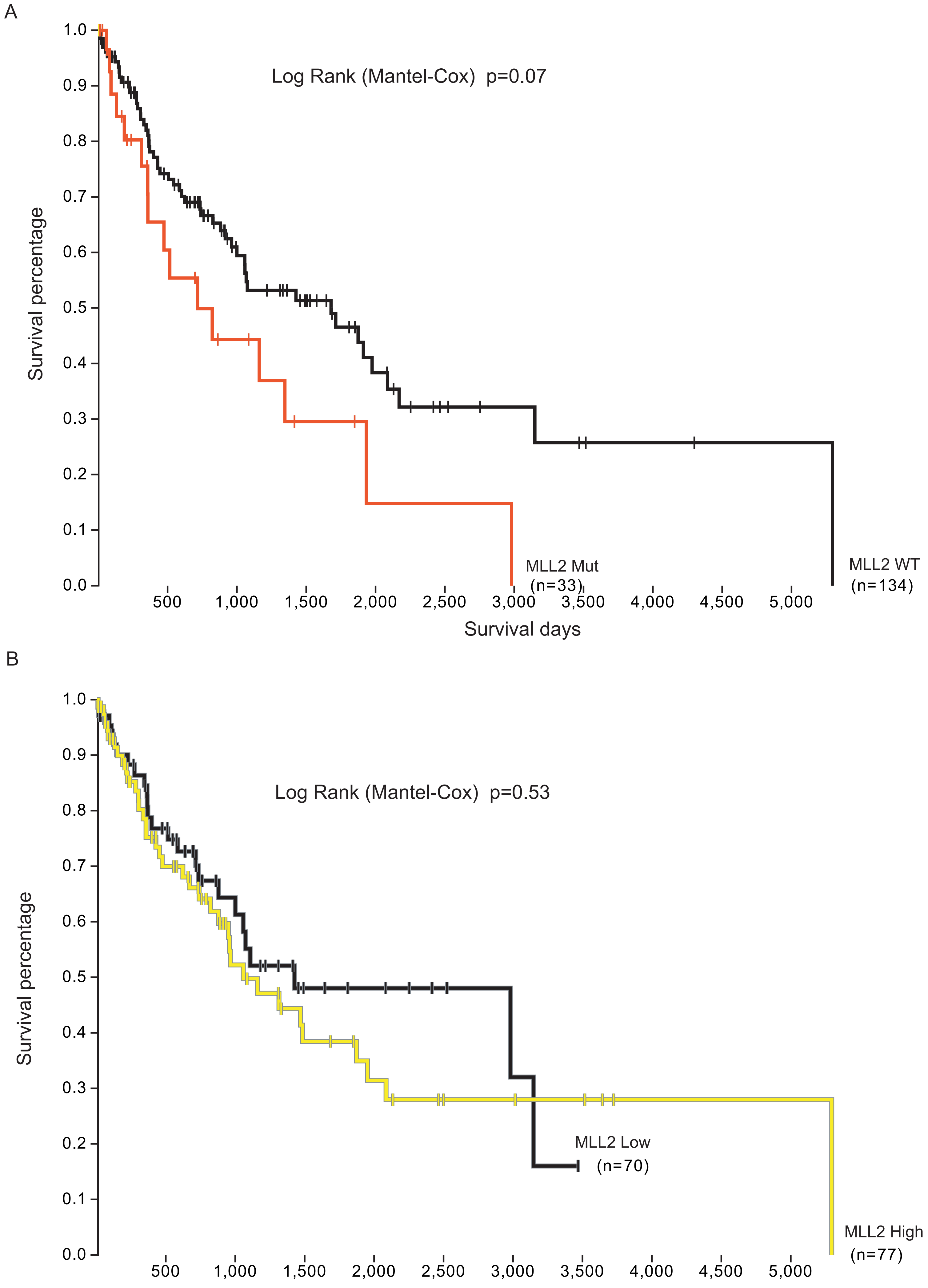

Supplementary Table 1. Summary of data quality in each sample

Samples	A1T	A1N	A2T	A2T	A3N	A3T	A4T	A4N	A5T	A5T	A6N	A6T	A7T	A7N	A8T	A8T	A9N	A9T
Reads mapped to genome:	1.13E+08	9.6E+07	1.06E+08	8.1E+07	8.9E+07	9.4E+07	1.17E+08	1.15E+08	8.9E+07	1.02E+08	1.18E+08	7.6E+07	9.4E+07	1.03E+08	1.05E+08	1.1E+08	8.7E+07	9E+07
Reads mapped to exons:	65732277	6.3E+07	71448777	5.4E+07	5.7E+07	6.1E+07	73866569	74140335	6.1E+07	66605165	62928977	5.2E+07	6.1E+07	68941943	63095328	72992294	5.9E+07	6.2E+07
Data mapped to exons (Mb):	4757.51	4567.27	5135.55	3895.13	4104.83	4401.97	5307.71	5329.36	4406.83	4802.44	4511.76	3756.7	4416.7	4965.08	4542.02	5266.55	4264.35	4490.64
Mean depth of exons:	107.8	103.49	116.1	88.06	92.8	99.52	120.27	120.76	99.63	108.57	102	84.93	99.85	112.25	102.68	119.34	96.4	101.52
Coverage of exons (%):	99.65	99.52	99.58	99.51	99.44	99.49	99.49	99.45	99.54	99.55	99.46	99.28	99.48	99.52	99.42	99.56	99.5	99.37
Average read length (bp):	89.94	89.93	89.94	89.92	89.94	89.92	89.94	89.91	89.94	89.91	89.95	89.92	89.95	89.93	89.93	89.92	89.92	89.93
Rate of nucleotide mismatch (%)	0.24	0.32	0.23	0.33	0.25	0.3	0.25	0.33	0.24	0.33	0.26	0.32	0.24	0.33	0.34	0.33	0.3	0.32
Fraction of exon covered >=4X:	98.95	98.72	98.9	98.59	98.62	98.69	98.82	98.72	98.89	98.86	98.59	98.23	98.66	98.75	98.48	98.84	98.65	98.37
Fraction of exon covered >=10X:	97.54	97.16	97.72	96.61	97.08	97.07	97.64	97.42	97.63	97.52	96.93	96.13	97.09	97.31	96.87	97.45	96.93	96.47
Fraction of exon covered >=20X:	94.55	93.75	95.31	91.95	93.71	93.47	95.38	94.79	94.77	94.7	93.22	91.29	93.55	94.3	93.59	94.59	93.05	92.43
Fraction of exon covered >=30X:	90.21	88.98	91.93	85.2	88.47	88.29	92.22	91.11	90.29	90.68	87.88	84.45	88.4	90.06	88.94	90.63	87.33	86.94
Fraction of exon covered >=40X:	84.41	82.85	87.35	76.89	81.24	81.66	87.91	86.34	84.04	85.34	81.01	76.06	81.76	84.57	82.83	85.56	80.03	80.25
Fraction of exon covered >=50X:	77.47	75.72	81.63	67.91	72.71	74.05	82.49	80.58	76.4	78.81	73.28	66.97	74.14	78.16	75.58	79.58	71.86	72.86
Fraction of exon covered >=60X:	69.9	68.08	75	58.95	63.66	66.05	76.13	74.19	68.01	71.54	65.26	57.81	66.07	71.23	67.77	73.07	63.4	65.24
Fraction of exon covered >=70X:	62.21	60.34	67.85	50.53	54.82	58.12	69.23	67.54	59.55	63.98	57.43	49.24	58.08	64.1	59.88	66.36	55.23	57.75
Fraction of exon covered >=80X:	54.81	52.93	60.62	42.96	46.65	50.6	62.22	60.88	51.47	56.54	50.18	41.56	50.56	57.13	52.33	59.72	47.69	50.67

Supplementary Table 2. Number of somatic mutations in each sample

Samples	A1	A2	A3	A4	A5	A6	A7	A8	A9
Gender	female	male	male	female	male	male	male	female	male
Smoking status	nonsmk	nonsmk	smk	nonsmk	nonsmk	smk	smk	nonsmk	smk
Intergenic	7	19	39	11	11	29	22	8	45
Intron	81	105	399	59	77	337	225	85	503
Coding-synonymous	21	13	55	12	16	73	48	22	106
Missense	38	23	197	8	31	155	139	34	302
Stop-gained	3	1	18	2	1	10	8	0	25
Stop-lost	0	0	1	0	0	0	0	0	3
Splice site	0	3	9	0	2	5	9	0	8
3'-UTR	3	5	19	3	4	17	17	5	29
5'-UTR	2	3	8	1	1	9	5	2	22
All	155	172	745	96	143	635	473	156	1043

Supplementary Table 3. All high-confident somatic mutations

Sample	Chromosome	Position	Gene Symbol	Accession	ReferenceBase	SampleAlleles	FunctionGVS	AminoAcids	ProteinPosition	cDNAPosition	Validated
A1	17	7577535	TP53	NM_000546.5	C	C/G	missense	ARG,THR	249/394	746	Yes
A3	2	37873324	CDC42EP3	NM_006449.3	C	A/C	missense	GLY,VAL	136/255	407	Yes
A3	17	7802715	CHD3	NM_001005271.2	T	A/T	missense	TRP,ARG	859/2060	2575	Yes
A3	8	113348909	CSMD3	NM_052900.2	C	A/C	stop-gained	GLU,stop	2227/3539	6679	Yes
A3	17	11514997	DNAH9	NM_001372.3	A	A/C	missense	GLN,HIS	268/4487	804	Yes
A3	1	216741423	ESRRG	NM_001134285.2	C	A/C	missense	VAL,LEU	180/436	538	Yes
A3	5	74130407	FAM169A	NM_015566.2	C	A/C	stop-gained	GLU,stop	112/671	334	Yes
A3	1	161647334	FCGR2B	NM_001002273.2	C	A/C	missense	ASN,LYS	288/291	864	Yes
A3	14	47566170	MDGA2	NM_001113498.2	C	A/C	missense	GLY,VAL	361/1026	1082	Yes
A3	12	49426235	MLL2	NM_003482.3	G	A/G	stop-gained	GLN,stop	4085/5538	12253	Yes
A3	3	108188955	MYH15	NM_014981.1	T	A/T	missense	GLU,ASP	516/1947	1548	Yes
A3	6	56716233	none	none	A	A/C	intergenic	none	NA	NA	Yes
A3	20	43561711	PABPC1L	NM_001124756.1	A	A/T	splice-3	none	NA	NA	Yes
A3	3	129286421	PLXND1	NM_015103.2	T	A/T	missense	THR,SER	1334/1926	4000	Yes
A3	15	42927169	STARD9	NM_020759.2	C	C/T	missense-near-splice	PRO,LEU	117/4701	350	Yes
A3	14	64469765	SYNE2	NM_015180.4	G	G/T	missense	ASP,TYR	1372/6886	4114	Yes
A3	17	7577127	TP53	NM_000546.5	C	A/C	stop-gained	GLU,stop	271/394	811	Yes
A3	2	179480115	TTN	NM_001256850.1	C	C/T	missense	ARG,HIS	14545/34351	43634	Yes
A3	2	179614620	TTN	NM_001256850.1	C	A/C	intron	none	NA	NA	Yes
A3	2	179639851	TTN	NM_001256850.1	C	C/G	missense	CYS,SER	2196/34351	6587	Yes
A3	6	158924230	TULP4	NM_001007466.2	G	A/G	intron	none	NA	NA	Yes
A5	11	108160428	ATM	NM_000051.3	G	G/T	missense	VAL,PHE	1446/3057	4336	Yes
A5	12	49447023	MLL2	NM_003482.3	C	C/T	missense	MET,ILE	307/5538	921	Yes
A5	17	7578478	TP53	NM_000546.5	G	C/G	missense	PRO,ARG	151/394	452	Yes
A6	17	79101614	AATK	NM_001080395.2	G	G/T	missense	LEU,MET	171/1375	511	Yes
A6	9	119858392	ASTN2	NM_014010.4	A	A/T	missense	SER,THR	352/1289	1054	Yes
A6	9	119976798	ASTN2	NM_014010.4	C	C/G	missense	ARG,PRO	285/1289	854	Yes
A6	5	24491727	CDH10	NM_006727.3	C	C/T	missense	GLY,ARG	612/789	1834	Yes
A6	3	130113888	COL6A5	NM_153264.5	G	A/G	missense	GLY,ARG	1050/2527	3148	Yes
A6	11	14502328	COPB1	NM_001144061.1	T	C/T	missense	MET,VAL	397/954	1189	Yes
A6	8	113293516	CSMD3	NM_052900.2	G	A/G	missense	THR,ILE	2963/3539	8888	Yes
A6	5	74137469	FAM169A	NM_015566.2	G	G/T	stop-gained	CYS,stop	11/671	33	Yes
A6	17	34074146	GAS2L2	NM_139285.3	G	G/T	missense	THR,LYS	325/881	974	Yes
A6	1	220275522	IARS2	NM_018060.3	G	G/T	missense	ARG,LEU	201/1013	602	Yes
A6	1	209788657	LAMB3	NM_000228.2	G	G/T	missense	HIS,ASN	1160/1173	3478	Yes
A6	1	209800312	LAMB3	NM_000228.2	C	A/C	missense	GLN,HIS	499/1173	1497	Yes
A6	5	32229973	MTMR12	NM_001040446.1	C	A/C	missense	VAL,PHE	719/748	2155	Yes
A6	10	55782806	PCDH15	NM_001142763.1	G	G/T	missense	ALA,GLU	796/1963	2387	Yes
A6	10	56106181	PCDH15	NM_001142763.1	C	C/G	missense	ASP,HIS	185/1963	553	Yes
A6	8	110418608	PKHD1L1	NM_177531.4	T	A/T	missense	LEU,MET	572/4244	1714	Yes
A6	20	13850834	SEL1L2	NM_025229.1	G	G/T	missense	LEU,ILE	374/689	1120	Yes

A6	6	152457899	SYNE1	NM_033071.3	A	A/T	missense	CYS,SER	8457/8750		25369	Yes
A6	7	11446643	THSD7A	NM_015204.2	T	G/T	missense	GLN,PRO	1319/1658		3956	Yes
A6	9	120475773	TLR4	NM_003266.3	A	A/T	missense	HIS,LEU	416/800		1247	Yes
A6	17	7577544	TP53	NM_000546.5	A	A/G	missense	MET,THR	246/394		737	Yes
A6	2	179540651	TTN	NM_001256850.1	T	C/T	missense	LYS,GLU	11138/34351		33412	Yes
A6	2	179629344	TTN	NM_001256850.1	C	C/G	missense	GLU,GLN	3300/34351		9898	Yes
A7	10	102753251	C10orf2	NM_001163812.1	C	C/G	utr-3	none	NA	NA		Yes
A7	7	50135945	C7orf72	NM_001161834.2	T	A/T	stop-gained	TYR,stop	88/439		264	Yes
A7	5	13789001	DNAH5	NM_001369.2	T	A/T	missense	HIS,LEU	2824/4625		8471	Yes
A7	18	5416223	EPB41L3	NM_012307.2	G	G/T	missense	PRO,HIS	554/1088		1661	Yes
A7	2	141528455	LRP1B	NM_018557.2	C	C/G	missense	CYS,SER	1874/4600		5621	Yes
A7	12	49433784	MLL2	NM_003482.3	G	A/G	missense	SER,LEU	2590/5538		7769	Yes
A7	12	49434618	MLL2	NM_003482.3	G	A/G	missense	SER,LEU	2312/5538		6935	Yes
A7	12	49436527	MLL2	NM_003482.3	G	A/G	stop-gained	GLN,stop	1927/5538		5779	Yes
A7	8	110431421	PKHD1L1	NM_177531.4	C	A/C	stop-gained	SER,stop	819/4244		2456	Yes
A7	5	128301847	SLC27A6	NM_001017372.1	T	G/T	missense	LEU,ARG	6/620		17	Yes
A7	17	7579533	TP53	NM_000546.5	G	A/G	stop-gained	GLN,stop	52/394		154	Yes
A7	1	216062253	USH2A	NM_206933.2	C	C/G	missense	GLY,ARG	2580/5203		7738	Yes
A8	11	83674033	DLG2	NM_001142699.1	G	C/G	missense	SER,CYS	412/976		1235	Yes
A9	4	123333851	ADAD1	NM_001159285.1	G	A/G	missense	ARG,LYS	368/566		1103	Yes
A9	14	105404821	AHNAK2	NM_138420.2	G	C/G	missense	SER,CYS	5656/5796		16967	Yes
A9	2	21228975	APOB	NM_000384.2	G	G/T	missense	LEU,MET	3589/4564		10765	Yes
A9	11	63419978	ATL3	NM_015459.3	T	C/T	missense	ILE,VAL	159/542		475	Yes
A9	1	17320148	ATP13A2	NM_001141973.1	C	C/G	missense	LEU,PHE	570/1176		1710	Yes
A9	X	76872153	ATRX	NM_000489.3	C	T/T	missense	GLU,LYS	1832/2493		5494	Yes
A9	5	24505272	CDH10	NM_006727.3	C	C/G	missense	ASP,HIS	448/789		1342	Yes
A9	5	24537735	CDH10	NM_006727.3	A	A/G	missense	SER,PRO	94/789		280	Yes
A9	2	228147099	COL4A3	NM_000091.4	G	G/T	missense	GLY,VAL	836/1671		2507	Yes
A9	2	228160027	COL4A3	NM_000091.4	C	C/G	missense	ALA,GLY	1187/1671		3560	Yes
A9	4	155156011	DCHS2	NM_017639.3	G	G/T	missense	PRO,THR	2810/2917		8428	Yes
A9	17	11790283	DNAH9	NM_001372.3	G	C/G	splice-5	none	NA	NA		Yes
A9	1	216824408	ESRRG	NM_001134285.2	C	A/C	missense	ALA,SER	143/436		427	Yes
A9	12	50757063	FAM186A	NM_001145475.1	C	A/C	stop-gained	GLU,stop	93/2352		277	Yes
A9	1	240374482	FMN2	NM_020066.4	A	A/T	stop-gained	LYS,stop	1338/1723		4012	Yes
A9	17	34071874	GAS2L2	NM_139285.3	C	C/G	stop-lost	stop,SER	881/881		2642	Yes
A9	2	141128384	LRP1B	NM_018557.2	C	C/T	missense	ASP,ASN	3635/4600		10903	Yes
A9	1	76272757	MSH4	NM_002440.3	G	G/T	missense	MET,ILE	173/937		519	Yes
A9	7	100647681	MUC12	NM_001164462.1	C	C/G	missense	GLN,GLU	4613/5336		13837	Yes
A9	3	108172870	MYH15	NM_014981.1	C	A/C	missense	GLN,HIS	814/1947		2442	Yes
A9	2	50280503	NRXN1	NM_001135659.1	G	A/G	missense	SER,LEU	1385/1548		4154	Yes
A9	1	228480406	OBSCN	NM_001098623.1	C	C/T	missense	ARG,CYS	3596/7969		10786	Yes
A9	3	129278549	PLXND1	NM_015103.2	G	C/G	coding-synonymous	none	1737/1926		5211	Yes
A9	12	81661781	PPFIA2	NM_001220473.1	G	G/T	missense	SER,ARG	1132/1248		3396	Yes

A9	8	52370222	PXDNL	NM_144651.4	G	A/G	missense	SER,LEU	273/1464		818	Yes
A9	15	34129911	RYR3	NM_001036.3	G	C/G	missense	LEU,PHE	3910/4871		11730	Yes
A9	2	165987782	SCN3A	NM_001081676.1	C	A/C	missense	GLY,VAL	797/1952		2390	Yes
A9	20	13869128	SEL1L2	NM_025229.1	C	C/T	missense	GLY,ARG	194/689		580	Yes
A9	22	38041455	SH3BP1	NM_018957.3	G	A/G	missense	GLU,LYS	288/702		862	Yes
A9	5	128351719	SLC27A6	NM_001017372.1	A	A/G	missense	MET,VAL	371/620		1111	Yes
A9	19	11138536	SMARCA4	NM_001128844.1	A	A/T	stop-gained	LYS,stop	1098/1648		3292	Yes
A9	X	123181202	STAG2	NM_001042749.1	A	C/C	splice-3	none	NA	NA		Yes
A9	15	42983332	STARD9	NM_020759.2	C	C/T	missense	ARG,CYS	3186/4701		9556	Yes
A9	6	152730761	SYNE1	NM_033071.3	G	A/G	missense	ALA,VAL	2112/8750		6335	Yes
A9	14	64628872	SYNE2	NM_015180.4	G	A/G	missense	ALA,THR	5393/6886		16177	Yes
A9	2	179429821	TTN	NM_001256850.1	C	C/G	missense	ARG,PRO	25372/34351		76115	Yes
A9	2	179444468	TTN	NM_001256850.1	A	A/G	missense	TYR,HIS	20845/34351		62533	Yes
A9	1	215972345	USH2A	NM_206933.2	C	A/C	missense	ASP,TYR	3288/5203		9862	Yes
A9	15	62242580	VPS13C	NM_001018088.2	C	C/T	missense	ASP,ASN	1525/3629		4573	Yes
A9	19	38383560	WDR87	NM_031951.3	A	A/T	missense	ILE,ASN	889/2874		2666	Yes
A9	2	168101434	XIRP2	NM_001079810.3	C	A/C	intron	none	NA	NA		Yes
A2	3	121414109	GOLGB1	NM_001256486.1	T	C/T	missense	LYS,ARG	1754/3270		5261	No
A5	20	43541447	PABPC1L	NM_001124756.1	G	A/G	missense	ALA,THR	114/615		340	No
A6	19	38383487	WDR87	NM_031951.3	C	A/C	missense	GLN,HIS	913/2874		2739	No
A7	17	11532779	DNAH9	NM_001372.3	G	G/T	stop-gained	GLU,stop	466/4487		1396	No
A7	1	152188314	HRNR	NM_001009931.1	G	A/G	missense	ARG,CYS	1931/2851		5791	No
A9	22	38044432	SH3BP1	NM_018957.3	G	A/G	missense	GLU,LYS	436/702		1306	No
A9	7	116759715	ST7	NM_018412.3	C	C/G	missense	SER,CYS	112/555		335	No
A1	17	79101635	AATK	NM_001080395.2	C	C/T	missense	VAL,MET	164/1375		490	
A1	X	153008788	ABCD1	NM_000033.3	G	A/G	missense	ARG,GLN	660/746		1979	
A1	15	100594016	ADAMTS17	NM_139057.2	C	C/G	intron	none	NA	NA		
A1	1	112031663	ADORA3	NM_001081976.1	A	A/G	intron	none	NA	NA		
A1	6	151670390	AKAP12	NM_005100.3	G	A/G	coding-synonymous	none	288/1783		864	
A1	7	91642057	AKAP9	NM_005751.4	G	C/G	intron	none	NA	NA		
A1	10	5032241	AKR1C2	NM_001354.5	A	A/G	intron	none	NA	NA		
A1	10	5149558	AKR1C3	NM_001253908.1	A	A/T	intron	none	NA	NA		
A1	11	67786318	ALDH3B1	NM_000694.2	C	C/T	missense	ARG,CYS	117/468		349	
A1	3	129811972	ALG1L2	NM_001136152.1	G	C/G	missense	GLN,HIS	94/216		282	
A1	2	112616040	ANAPC1	NM_022662.3	T	C/T	intron	none	NA	NA		
A1	9	38595668	ANKRD18A	NM_147195.2	C	A/C	stop-gained	GLU,stop	557/993		1669	
A1	4	125590931	ANKRD50	NM_001167882.1	A	A/G	coding-synonymous	none	988/1251		2964	
A1	16	332558	ARHGDIG	NM_001176.3	C	C/G	intron	none	NA	NA		
A1	7	143885448	ARHGEF35	NM_001003702.2	G	C/G	missense	ALA,GLY	10/485		29	
A1	3	57310648	ASB14	NM_001142733.2	C	C/T	missense	ARG,HIS	565/588		1694	
A1	X	119512478	ATP1B4	NM_001142447.2	G	G/T	intron	none	NA	NA		
A1	1	110030395	ATXN7L2	NM_153340.4	G	A/G	coding-synonymous	none	223/723		669	
A1	20	48273127	B4GALT5	NM_004776.3	C	C/G	missense	ARG,SER	76/389		228	

A1	20	48273145	B4GALT5	NM_004776.3	C	A/C	coding-synonymous	none	70/389		210
A1	20	48273184	B4GALT5	NM_004776.3	C	C/T	coding-synonymous	none	57/389		171
A1	16	1397518	BAIAP3	NM_001199096.1	G	C/G	intron	none	NA	NA	
A1	20	52612765	BCAS1	NM_003657.2	T	G/T	intron	none	NA	NA	
A1	12	105388230	C12orf45	NM_152318.2	G	C/G	intron	none	NA	NA	
A1	14	91671004	C14orf159	NM_001102366.1	A	A/C	intron	none	NA	NA	
A1	17	32904422	C17orf102	NM_207454.2	G	G/T	utr-3	none	NA	NA	
A1	13	24893145	C1QTNF9	NM_178540.3	C	C/T	intron	none	NA	NA	
A1	1	57373622	C8A	NM_000562.2	A	A/G	intron	none	NA	NA	
A1	9	96098011	C9orf129	NM_001098808.1	C	C/T	intron	none	NA	NA	
A1	15	63638835	CA12	NM_001218.3	C	C/G	coding-synonymous	none	60/355		180
A1	12	2602462	CACNA1C	NM_000719.6	C	C/T	coding-synonymous	none	341/2139		1023
A1	17	46926661	CALCOCO2	NM_001261390.1	A	A/G	coding-synonymous	none	179/471		537
A1	12	96704971	CDK17	NM_001170464.2	A	A/G	intron	none	NA	NA	
A1	12	88525031	CEP290	NM_025114.3	G	A/G	intron	none	NA	NA	
A1	20	40102012	CHD6	NM_032221.3	C	C/T	missense	ASP,ASN	872/2716		2614
A1	16	74444238	CLEC18B	NM_001011880.2	C	C/T	intron	none	NA	NA	
A1	11	66046044	CNIH2	NM_182553.1	T	G/T	intron	none	NA	NA	
A1	9	137642325	COL5A1	NM_000093.3	C	C/T	intron	none	NA	NA	
A1	10	70715988	DDX21	NM_004728.3	G	A/G	missense	GLY,ARG	3/784		7
A1	8	7343878	DEFB106B	NM_001040704.1	G	A/G	coding-synonymous	none	9/66		27
A1	17	76562713	DNAH17	NM_173628.3	T	C/T	missense	ILE,VAL	518/4463		1552
A1	16	68109354	DUS2L	NM_017803.3	G	G/T	coding-synonymous	none	343/494		1029
A1	2	109527099	EDAR	NM_022336.3	G	A/G	intron	none	NA	NA	
A1	8	132991192	EFR3A	NM_015137.4	G	A/G	coding-synonymous	none	475/822		1425
A1	2	99998583	EIF5B	NM_015904.3	G	A/G	intron	none	NA	NA	
A1	11	34515135	ELF5	NM_001243080.1	G	A/G	intron	none	NA	NA	
A1	5	453852	EXOC3	NM_007277.4	G	A/G	stop-gained	TRP,stop	244/746		732
A1	3	197894798	FAM157A	NM_001145248.1	T	C/T	intron	none	NA	NA	
A1	3	197896856	FAM157A	NM_001145248.1	A	A/T	intron	none	NA	NA	
A1	3	197896870	FAM157A	NM_001145248.1	C	C/T	intron	none	NA	NA	
A1	9	90749760	FAM75C2	NM_001166137.1	A	A/G	missense	PHE,LEU	38/1135		112
A1	12	29474882	FAR2	NM_018099.3	C	A/C	intron	none	NA	NA	
A1	19	40368274	FCGBP	NM_003890.2	C	A/C	intron	none	NA	NA	
A1	5	39153391	FYB	NM_001243093.1	T	C/T	intron	none	NA	NA	
A1	22	25023249	GGT1	NM_001032364.2	C	C/T	intron	none	NA	NA	
A1	17	42428156	GRN	NM_002087.2	C	C/T	coding-synonymous	none	232/594		696
A1	10	115336896	HABP2	NM_001177660.1	G	A/G	intron	none	NA	NA	
A1	15	28456220	HERC2	NM_004667.5	G	A/G	missense	LEU,PHE	2333/4835		6997
A1	15	28499738	HERC2	NM_004667.5	C	C/T	intron	none	NA	NA	
A1	12	54677085	HNRNPA1	NM_002136.2	G	G/T	intron	none	NA	NA	
A1	2	198363534	HSPD1	NM_002156.4	C	C/T	coding-synonymous	none	13/574		39
A1	2	27669309	IFT172	NM_001168364.1	A	A/G	utr-3	none	NA	NA	

A1	3	49066804	IMPDH2	NM_000884.2	G	A/G	utr-5	none	NA	NA
A1	11	17801111	KCNC1	NM_001112741.1	G	G/T	missense	GLY,VAL	538/586	1613
A1	17	2597728	KIAA0664	NM_015229.3	G	A/G	intron	none	NA	NA
A1	4	123270710	KIAA1109	NM_015312.3	G	G/T	missense	GLY,VAL	4515/5006	13544
A1	10	24783445	KIAA1217	NM_001098500.1	A	A/G	missense	MET,VAL	486/1265	1456
A1	4	37446864	KIAA1239	NM_001144990.1	A	A/T	missense	ASP,VAL	1085/1743	3254
A1	3	113379446	KIAA2018	NM_001009899.2	C	A/C	missense	LYS,ASN	361/2246	1083
A1	20	16506949	KIF16B	NM_001199865.1	C	C/T	intron	none	NA	NA
A1	20	60908913	LAMA5	NM_005560.3	C	C/G	intron	none	NA	NA
A1	19	55112394	LILRA1	NM_006863.1	T	C/T	utr-3	none	NA	NA
A1	2	102504146	MAP4K4	NM_001242559.1	T	G/T	intron	none	NA	NA
A1	X	138680737	MCF2	NM_001099855.1	C	C/T	intron	none	NA	NA
A1	X	119739359	MCTS1	NM_001137554.1	G	A/G	missense	GLU,LYS	38/183	112
A1	17	60072495	MED13	NM_005121.2	T	G/T	intron	none	NA	NA
A1	19	42861030	MEGF8	NM_001410.2	C	C/G	missense	ALA,GLY	1509/2779	4526
A1	12	82783615	METTL25	NM_032230.2	G	C/G	missense	GLU,GLN	149/604	445
A1	7	156798528	MNX1	NM_001165255.1	G	A/G	missense	ARG,CYS	86/190	256
A1	6	84772820	MRAP2	NM_138409.2	G	A/G	intron	none	NA	NA
A1	11	73499106	MRPL48	NM_016055.5	G	C/G	intron	none	NA	NA
A1	3	195508343	MUC4	NM_004532.5	A	A/T	intron	none	NA	NA
A1	11	1266696	MUC5B	NM_002458.2	A	A/C	coding-synonymous	none	2862/5763	8586
A1	6	76532558	MYO6	NM_004999.3	G	G/T	coding-synonymous	none	59/1286	177
A1	8	71041125	NCOA2	NM_006540.2	A	T/T	missense	SER,THR	1139/1465	3415
A1	2	240900612	NDUFA10	NM_004544.3	G	A/G	intron	none	NA	NA
A1	17	29557960	NF1	NM_000267.3	C	C/T	intron	none	NA	NA
A1	17	29557966	NF1	NM_000267.3	G	A/G	intron	none	NA	NA
A1	16	69727393	NFAT5	NM_001113178.2	C	C/T	missense	PRO,LEU	1221/1549	3662
A1	2	89521584	none	none	G	C/G	intergenic	none	NA	NA
A1	9	125240210	none	none	A	A/T	near-gene-5	none	NA	NA
A1	10	5194972	none	none	C	A/C	intergenic	none	NA	NA
A1	11	55111684	none	none	A	A/C	near-gene-3	none	NA	NA
A1	14	106234049	none	none	T	C/T	intergenic	none	NA	NA
A1	21	31964656	none	none	G	G/T	near-gene-5	none	NA	NA
A1	22	23135008	none	none	G	A/G	intergenic	none	NA	NA
A1	11	114392941	NXPE1	NM_152315.2	A	A/G	missense	PHE,LEU	323/406	967
A1	7	103841381	ORC5	NM_002553.3	A	A/C	missense	VAL,GLY	64/436	191
A1	16	21742020	OTOA	NM_001161683.1	T	C/T	intron	none	NA	NA
A1	12	80663826	OTOGL	NM_173591.3	A	A/G	intron	none	NA	NA
A1	22	43272830	PACSIN2	NM_001184970.1	C	C/G	intron	none	NA	NA
A1	10	55892600	PCDH15	NM_001142763.1	T	C/T	intron	none	NA	NA
A1	20	2819735	PCED1A	NM_022760.3	A	A/C	intron	none	NA	NA
A1	12	15132193	PDE6H	NM_006205.2	G	C/G	intron	none	NA	NA
A1	22	21063376	PI4KA	NM_002650.2	C	C/T	intron	none	NA	NA

A1	7	47884758	PKD1L1	NM_138295.3	A	A/G	intron	none	NA	NA	
A1	16	71981414	PKD1L3	NM_181536.1	C	C/G	missense	LYS,ASN	1233/1733		3695
A1	8	110471797	PKHD1L1	NM_177531.4	A	A/T	intron	none	NA	NA	
A1	9	131482535	PKN3	NM_013355.3	G	A/G	coding-synonymous	none	810/890		2430
A1	2	179363807	PLEKHA3	NM_019091.3	A	A/T	intron	none	NA	NA	
A1	9	139307194	PMPCA	NM_015160.1	T	A/T	intron	none	NA	NA	
A1	7	102309118	POLR2J2	NM_032959.5	A	A/C	intron	none	NA	NA	
A1	7	102279468	POLR2J2	NM_001114403.2	C	C/G	intron	none	NA	NA	
A1	7	76241234	POMZP3	NM_012230.3	G	G/T	intron	none	NA	NA	
A1	19	30106281	POP4	NM_006627.2	C	C/T	coding-synonymous	none	219/221		657
A1	9	33796429	PRSS3	NM_001197097.2	G	A/G	intron	none	NA	NA	
A1	9	33799163	PRSS3	NM_001197097.2	C	A/C	coding-synonymous	none	257/262		771
A1	19	40480545	PSMC4	NM_006503.3	G	G/T	intron	none	NA	NA	
A1	1	71477992	PTGER3	NM_001126044.1	C	A/C	missense	CYS,PHE	358/391		1073
A1	21	45533966	PWP2	NM_005049.2	G	A/G	intron	none	NA	NA	
A1	7	4871641	RADIL	NM_018059.4	T	G/T	intron	none	NA	NA	
A1	7	102257175	RASA4	NM_001079877.2	C	A/C	utr-5	none	NA	NA	
A1	12	114397612	RBM19	NM_001146698.1	C	C/T	intron	none	NA	NA	
A1	3	29925598	RBMS3	NM_001003792.2	G	A/G	intron	none	NA	NA	
A1	12	56114970	RDH5	NM_001199771.1	T	A/T	missense	MET,LYS	1/319		2
A1	6	111711313	REV3L	NM_002912.3	T	C/T	missense	ILE,VAL	245/3131		733
A1	14	93118898	RIN3	NM_024832.3	C	A/C	missense	GLN,LYS	502/986		1504
A1	10	99160200	RRP12	NM_001145114.1	C	C/T	coding-synonymous	none	77/1237		231
A1	7	6836078	RSPH10B2	NM_001099697.1	A	A/C	intron	none	NA	NA	
A1	1	237608920	RYR2	NM_001035.2	T	A/T	intron	none	NA	NA	
A1	7	83823738	SEMA3A	NM_006080.2	G	A/G	intron	none	NA	NA	
A1	9	37920031	SHB	NM_003028.2	T	C/T	intron	none	NA	NA	
A1	19	51920278	SIGLEC10	NM_001171156.1	G	C/G	intron	none	NA	NA	
A1	13	21729965	SKA3	NM_001166017.1	G	A/G	intron	none	NA	NA	
A1	20	35242441	SLA2	NM_032214.3	A	A/T	intron	none	NA	NA	
A1	X	129498781	SLC25A14	NM_003951.2	C	C/T	intron	none	NA	NA	
A1	11	20652159	SLC6A5	NM_004211.3	A	A/T	intron	none	NA	NA	
A1	10	108339122	SORCS1	NM_001013031.2	C	C/T	intron	none	NA	NA	
A1	16	2817796	SRRM2	NM_016333.3	C	C/T	missense	ARG,TRP	2423/2753		7267
A1	17	35802615	TADA2A	NM_001166105.1	T	C/T	intron	none	NA	NA	
A1	9	75441827	TMC1	NM_138691.2	G	A/G	coding-synonymous	none	682/761		2046
A1	3	127292479	TPRA1	NM_001136053.1	G	A/G	missense	THR,MET	336/374		1007
A1	13	20038663	TPTE2	NM_001141968.1	G	A/G	intron	none	NA	NA	
A1	6	111913193	TRAF3IP2	NM_001164281.2	C	C/G	missense	GLU,GLN	33/565		97
A1	X	47517091	UXT	NM_004182.3	G	C/G	intron	none	NA	NA	
A1	3	51457234	VPRBP	NM_001171904.1	C	A/C	stop-gained	GLY,stop	1010/1454		3028
A1	3	184577767	VPS8	NM_001009921.2	C	C/T	intron	none	NA	NA	
A1	3	184682289	VPS8	NM_001009921.2	G	A/G	missense	ALA,THR	1073/1429		3217

A1	2	20132162	WDR35	NM_001006657.1	A	A/G	missense	ILE,THR	913/1182		2738
A1	11	123598994	ZNF202	NM_003455.2	C	C/G	intron	none	NA	NA	
A1	16	89795778	ZNF276	NM_001113525.1	A	A/C	intron	none	NA	NA	
A1	19	20117946	ZNF682	NM_001077349.1	T	G/T	missense	GLU,ALA	90/467		269
A1	7	88389324	ZNF804B	NM_181646.2	C	C/T	missense	LEU,PHE	12/1350		34
A1	19	53848764	ZNF845	NM_138374.1	A	A/G	coding-synonymous	none	7/971		21
A2	9	107550886	ABCA1	NM_005502.3	G	G/T	intron	none	NA	NA	
A2	16	16308311	ABCC6	NM_001171.5	G	A/G	intron	none	NA	NA	
A2	17	1003720	ABR	NM_001092.4	G	A/G	intron	none	NA	NA	
A2	11	67048131	ADRBK1	NM_001619.3	T	G/T	intron	none	NA	NA	
A2	10	51753967	AGAP6	NM_001077665.2	G	A/G	intron	none	NA	NA	
A2	1	26648704	AIM1L	NM_001039775.3	C	C/T	coding-synonymous	none	1616/1662		4848
A2	3	125831587	ALDH1L1	NM_001270364.1	G	A/G	intron	none	NA	NA	
A2	11	69949137	ANO1	NM_018043.5	C	C/G	intron	none	NA	NA	
A2	11	419795	ANO9	NM_001012302.2	A	A/C	intron	none	NA	NA	
A2	20	61910068	ARFGAP1	NM_018209.2	T	G/T	intron	none	NA	NA	
A2	20	47630086	ARFGEF2	NM_006420.2	G	C/G	intron	none	NA	NA	
A2	3	137991808	ARMC8	NM_001267041.1	G	A/G	splice-3	none	NA	NA	
A2	10	51974489	ASAH2	NM_001143974.1	T	C/T	intron	none	NA	NA	
A2	1	44451105	B4GALT2	NM_001005417.2	T	G/T	intron	none	NA	NA	
A2	21	11098814	BAGE2	NM_182482.2	C	A/C	utr-5	none	NA	NA	
A2	8	143607870	BAI1	NM_001702.2	A	A/C	intron	none	NA	NA	
A2	10	43315629	BMS1	NM_014753.3	T	C/T	intron	none	NA	NA	
A2	22	23964371	C22orf43	NM_016449.3	G	C/G	intron	none	NA	NA	
A2	2	85622008	CAPG	NM_001256139.1	G	A/G	utr-3	none	NA	NA	
A2	19	36640554	CAPNS1	NM_001003962.1	T	G/T	intron	none	NA	NA	
A2	16	2236911	CASKIN1	NM_020764.3	T	G/T	intron	none	NA	NA	
A2	15	43924652	CATSPER2	NM_054020.2	G	A/G	intron	none	NA	NA	
A2	17	20769710	CCDC144NL	NM_001004306.1	A	A/G	intron	none	NA	NA	
A2	15	43025209	CDAN1	NM_138477.2	A	A/G	intron	none	NA	NA	
A2	1	227219196	CDC42BPA	NM_003607.3	G	A/G	missense	SER,LEU	1158/1720		3473
A2	11	64604195	CDC42BPG	NM_017525.2	T	G/T	intron	none	NA	NA	
A2	11	618279	CDHR5	NM_001171968.1	C	C/T	intron	none	NA	NA	
A2	9	123249624	CDK5RAP2	NM_001011649.1	G	A/G	missense	THR,ILE	525/1815		1574
A2	11	125830912	CDON	NM_001243597.1	C	C/T	coding-synonymous	none	1263/1288		3789
A2	2	169312930	CERS6	NM_001256126.1	T	G/T	utr-5	none	NA	NA	
A2	14	21859080	CHD8	NM_001170629.1	A	A/G	intron	none	NA	NA	
A2	15	43991374	CKMT1A	NM_001015001.1	T	C/T	utr-3	none	NA	NA	
A2	5	157285895	CLINT1	NM_001195555.1	T	C/T	intron	none	NA	NA	
A2	9	140161630	COBRA1	NM_015456.3	G	A/G	intron	none	NA	NA	
A2	6	55988962	COL21A1	NM_030820.3	C	C/T	intron	none	NA	NA	
A2	21	47552150	COL6A2	NM_001849.3	C	C/T	missense	SER,LEU	915/1020		2744
A2	17	14095752	COX10	NM_001303.3	A	A/G	intron	none	NA	NA	

A2	16	3794997	CREBBP	NM_001079846.1	T	C/T	intron	none	NA	NA	
A2	19	18871085	CRTC1	NM_001098482.1	G	G/T	intron	none	NA	NA	
A2	5	149441000	CSF1R	NM_005211.3	A	A/C	intron	none	NA	NA	
A2	1	162741962	DDR2	NM_001014796.1	A	A/T	missense	LYS,ASN	551/856		1653
A2	12	31237922	DDX11	NM_001257144.1	G	C/G	missense	ARG,THR	167/971		500
A2	22	19050602	DGCR2	NM_001173533.1	C	A/C	intron	none	NA	NA	
A2	5	61699021	DIMT1	NM_014473.2	C	C/G	intron	none	NA	NA	
A2	15	45411272	DUOXA1	NM_144565.2	G	A/G	intron	none	NA	NA	
A2	10	64575656	EGR2	NM_000399.3	C	C/T	missense	GLY,GLU	45/477		134
A2	3	184910262	EHHADH	NM_001166415.1	T	C/T	missense	SER,GLY	546/628		1636
A2	19	14876693	EMR2	NM_013447.2	G	A/G	intron	none	NA	NA	
A2	12	12022812	ETV6	NM_001987.4	C	C/G	coding-synonymous	none	306/453		918
A2	6	65611952	EYS	NM_001142800.1	T	A/T	intron	none	NA	NA	
A2	10	51859716	FAM21A	NM_001005751.1	C	A/C	intron	none	NA	NA	
A2	10	46269017	FAM21C	NM_001169106.1	C	A/C	intron	none	NA	NA	
A2	11	92620165	FAT3	NM_001008781.2	G	C/G	splice-3	none	NA	NA	
A2	17	18668225	FBXW10	NM_001267585.1	G	A/G	intron	none	NA	NA	
A2	7	4801940	FOXK1	NM_001037165.1	A	A/C	missense	THR,PRO	683/734		2047
A2	4	190881849	FRG1	NM_004477.2	G	G/T	intron	none	NA	NA	
A2	10	49384130	FRMPD2	NM_001018071.3	C	C/T	intron	none	NA	NA	
A2	5	154305493	GEMIN5	NM_001252156.1	T	G/T	missense	ASN,HIS	407/1508		1219
A2	17	36484002	GPR179	NM_001004334.2	A	A/C	missense	ILE,SER	1817/2368		5450
A2	7	74554651	GTF2IRD2B	NM_001003795.2	G	A/G	intron	none	NA	NA	
A2	16	27514145	GTF3C1	NM_001520.3	C	C/T	intron	none	NA	NA	
A2	19	36104926	HAUS5	NM_015302.1	C	C/T	intron	none	NA	NA	
A2	19	597386	HCN2	NM_001194.3	G	C/G	intron	none	NA	NA	
A2	10	69785443	HERC4	NM_015601.3	G	A/G	intron	none	NA	NA	
A2	6	12131111	HIVEP1	NM_002114.2	A	A/T	coding-synonymous	none	2107/2719		6321
A2	2	209110270	IDH1	NM_005896.2	C	C/T	intron	none	NA	NA	
A2	17	25912650	KSR1	NM_014238.1	G	A/G	intron	none	NA	NA	
A2	19	48656981	LIG1	NM_000234.1	C	C/T	intron	none	NA	NA	
A2	19	54726665	LILRB3	NM_001081450.1	G	A/G	intron	none	NA	NA	
A2	6	161021922	LPA	NM_005577.2	A	A/G	intron	none	NA	NA	
A2	11	65308196	LTBP3	NM_001130144.2	C	C/T	intron	none	NA	NA	
A2	14	65560493	MAX	NM_002382.3	C	C/G	missense	ARG,PRO	35/161		104
A2	2	172195620	METTL8	NM_024770.3	T	C/T	intron	none	NA	NA	
A2	17	17039564	MPRIP	NM_015134.3	G	C/G	missense	SER,THR	179/1039		536
A2	16	86588190	MTHFSD	NM_001159377.1	A	A/G	intron	none	NA	NA	
A2	3	195509886	MUC4	NM_004532.5	G	C/G	intron	none	NA	NA	
A2	3	108147263	MYH15	NM_014981.1	T	A/T	intron	none	NA	NA	
A2	18	3174076	MYOM1	NM_003803.3	A	A/C	intron	none	NA	NA	
A2	1	148004386	NBPF14	NM_015383.1	G	C/G	utr-3	none	NA	NA	
A2	12	45173637	NELL2	NM_001145107.1	G	G/T	stop-gained	CYS,stop	218/867		654

A2	1	149282920	none	none	T	C/T	intergenic	none	NA	NA	
A2	2	133015283	none	none	G	C/G	intergenic	none	NA	NA	
A2	3	75679809	none	none	C	A/C	intergenic	none	NA	NA	
A2	3	75679833	none	none	C	C/T	intergenic	none	NA	NA	
A2	5	57878871	none	none	C	A/C	near-gene-5	none	NA	NA	
A2	5	177483029	none	none	T	G/T	intergenic	none	NA	NA	
A2	5	177483035	none	none	A	A/G	intergenic	none	NA	NA	
A2	5	177483055	none	none	C	C/T	intergenic	none	NA	NA	
A2	7	142139551	none	none	T	A/T	intergenic	none	NA	NA	
A2	10	49203135	none	none	C	A/C	near-gene-3	none	NA	NA	
A2	11	10831043	none	none	C	C/G	near-gene-5	none	NA	NA	
A2	11	48367140	none	none	C	C/T	intergenic	none	NA	NA	
A2	11	55872381	none	none	G	G/T	near-gene-5	none	NA	NA	
A2	14	106054693	none	none	C	A/C	intergenic	none	NA	NA	
A2	16	21396929	none	none	A	A/T	intergenic	none	NA	NA	
A2	19	54261393	none	none	T	C/T	intergenic	none	NA	NA	
A2	19	54261421	none	none	C	C/G	intergenic	none	NA	NA	
A2	19	54261427	none	none	A	A/G	intergenic	none	NA	NA	
A2	19	54261432	none	none	A	A/G	intergenic	none	NA	NA	
A2	7	24324824	NPY	NM_000905.3	C	A/C	intron	none	NA	NA	
A2	11	79113051	ODZ4	NM_001098816.2	A	A/G	intron	none	NA	NA	
A2	1	248112930	OR2L13	NM_001001963.1	T	C/T	coding-synonymous	none	257/313		771
A2	11	55322086	OR4C15	NM_001001920.1	C	A/C	missense	LEU,ILE	102/371		304
A2	11	4928674	OR51A7	NM_001004749.1	T	C/T	coding-synonymous	none	25/313		75
A2	12	80672951	OTOGL	NM_173591.3	G	G/T	intron	none	NA	NA	
A2	12	121666210	P2RX4	NM_001256796.1	C	C/T	intron	none	NA	NA	
A2	2	206023569	PARD3B	NM_057177.6	G	A/G	missense	GLU,LYS	520/1137		1558
A2	11	125617748	PATE1	NM_138294.2	G	G/T	intron	none	NA	NA	
A2	10	55626630	PCDH15	NM_001142763.1	G	A/G	intron	none	NA	NA	
A2	5	140710444	PCDHGA1	NM_018912.2	C	C/T	missense	ARG,CYS	65/932		193
A2	5	140753995	PCDHGB3	NM_018912.2	T	A/T	intron	none	NA	NA	
A2	2	120404627	PCDP1	NM_001029996.3	G	C/G	splice-5	none	NA	NA	
A2	10	112645142	PDCD4	NM_001199492.1	A	A/T	intron	none	NA	NA	
A2	16	20387314	PDILT	NM_174924.1	C	C/G	intron	none	NA	NA	
A2	2	239184691	PER2	NM_022817.2	G	A/G	intron	none	NA	NA	
A2	18	10705602	PIEZO2	NM_022068.2	C	C/G	missense	ASP,HIS	1798/2753		5392
A2	15	42302332	PLA2G4E	NM_001206670.1	C	A/C	intron	none	NA	NA	
A2	22	38539332	PLA2G6	NM_001004426.1	G	G/T	intron	none	NA	NA	
A2	5	41382519	PLCXD3	NM_001005473.2	C	C/T	missense	ARG,GLN	74/322		221
A2	10	118386254	PNLIPRP2	NM_005396.4	T	G/T	intron	none	NA	NA	
A2	7	142460087	PRSS1	NM_002769.4	A	A/C	intron	none	NA	NA	
A2	9	8465681	PTPRD	NM_001040712.2	G	C/G	intron	none	NA	NA	
A2	7	121652541	PTPRZ1	NM_001206838.1	C	C/G	intron	none	NA	NA	

A2	11	110118604	RDX	NM_001260492.1	A	A/G	intron	none	NA	NA	
A2	13	34403994	RFC3	NM_002915.3	A	A/T	missense	ASP,VAL	138/357		413
A2	19	33493664	RHPN2	NM_033103.4	C	C/T	intron	none	NA	NA	
A2	19	33493715	RHPN2	NM_033103.4	C	C/T	intron	none	NA	NA	
A2	19	33493722	RHPN2	NM_033103.4	A	A/G	coding-synonymous	none	315/687		945
A2	14	21775957	RPGRIP1	NM_020366.3	T	C/T	coding-synonymous	none	290/1287		868
A2	7	6825450	RSPH10B2	NM_001099697.1	C	C/T	intron	none	NA	NA	
A2	5	256573	SDHA	NM_004168.2	A	A/G	utr-3	none	NA	NA	
A2	22	42952282	SERHL2	NM_014509.3	G	A/G	intron	none	NA	NA	
A2	2	85662102	SH2D6	NM_198482.1	A	A/C	coding-synonymous	none	8/176		24
A2	5	54624727	SKIV2L2	NM_015360.4	A	A/G	intron	none	NA	NA	
A2	5	127520438	SLC12A2	NM_001046.2	T	C/T	intron	none	NA	NA	
A2	8	11189681	SLC35G5	NM_054028.1	A	A/G	utr-3	none	NA	NA	
A2	5	150853213	SLC36A1	NM_078483.2	G	C/G	intron	none	NA	NA	
A2	5	482162	SLC9A3	NM_004174.2	G	C/G	intron	none	NA	NA	
A2	4	123978263	SPATA5	NM_145207.2	T	C/T	intron	none	NA	NA	
A2	12	53467049	SPRYD3	NM_032840.2	A	A/C	intron	none	NA	NA	
A2	11	62592870	STX5	NM_001244666.1	C	C/T	intron	none	NA	NA	
A2	22	24578987	SUSD2	NM_019601.3	T	C/T	intron	none	NA	NA	
A2	12	102122814	SYCP3	NM_001177948.1	C	C/T	intron	none	NA	NA	
A2	6	152757317	SYNE1	NM_033071.3	C	A/C	intron	none	NA	NA	
A2	7	99711461	TAF6	NM_001190415.1	A	A/C	intron	none	NA	NA	
A2	17	80828207	TBCD	NM_005993.4	C	C/T	missense	ARG,CYS	476/1193		1426
A2	16	30098182	TBX6	NM_004608.3	A	A/G	intron	none	NA	NA	
A2	5	149772167	TCOF1	NM_000356.3	T	G/T	intron	none	NA	NA	
A2	8	133978713	TG	NM_003235.4	G	C/G	intron	none	NA	NA	
A2	10	98319418	TM9SF3	NM_020123.3	G	A/G	missense	ARG,TRP	220/590		658
A2	8	22881807	TNFRSF10B	NM_003842.4	G	C/G	intron	none	NA	NA	
A2	22	35719026	TOM1	NM_001135729.1	A	A/G	coding-synonymous	none	41/461		123
A2	6	41118873	TREML1	NM_178174.2	T	G/T	intron	none	NA	NA	
A2	19	31770414	TSHZ3	NM_020856.2	G	A/G	coding-synonymous	none	95/1082		285
A2	21	46020463	TSPEAR	NM_144991.2	G	C/G	intron	none	NA	NA	
A2	5	94849197	TTC37	NM_014639.3	C	C/T	intron	none	NA	NA	
A2	7	138950992	UBN2	NM_173569.3	G	A/G	intron	none	NA	NA	
A2	1	19432162	UBR4	NM_020765.2	C	C/G	intron	none	NA	NA	
A2	3	41979669	ULK4	NM_017886.2	T	C/T	missense	ILE,MET	53/1276		159
A2	22	30165487	UQCR10	NM_001003684.1	A	A/G	intron	none	NA	NA	
A2	11	64004573	VEGFB	NM_001243733.1	G	A/G	intron	none	NA	NA	
A2	2	168107146	XIRP2	NM_001079810.3	C	A/C	intron	none	NA	NA	
A2	1	180695848	XPR1	NM_001135669.1	T	G/T	intron	none	NA	NA	
A2	14	100728689	YY1	NM_003403.3	T	C/T	missense	ILE,THR	243/415		728
A2	14	100728720	YY1	NM_003403.3	A	A/G	coding-synonymous	none	253/415		759
A2	19	2867339	ZNF556	NM_024967.1	C	C/T	utr-5	none	NA	NA	

A2	19	7082101	ZNF557	NM_001044387.1	G	G/T	intron	none	NA	NA	
A2	19	58371430	ZNF587	NM_001204817.1	C	C/T	coding-synonymous	none	549/575		1647
A2	4	53577	ZNF595	NM_182524.2	G	A/G	intron	none	NA	NA	
A2	7	63709601	ZNF679	NM_153363.2	T	C/T	intron	none	NA	NA	
A2	8	7217399	ZNF705G	NM_001164457.1	T	G/T	intron	none	NA	NA	
A3	16	70310868	AARS	NM_001605.2	C	A/C	splice-5	none	NA	NA	
A3	1	94528699	ABCA4	NM_000350.2	C	A/C	missense	VAL,LEU	577/2274		1729
A3	17	67283622	ABCA5	NM_018672.3	G	C/G	intron	none	NA	NA	
A3	17	67133334	ABCA6	NM_080284.2	T	G/T	intron	none	NA	NA	
A3	19	1042816	ABCA7	NM_019112.3	G	G/T	coding-synonymous	none	190/2147		570
A3	7	150731572	ABCB8	NM_007188.3	T	A/T	intron	none	NA	NA	
A3	1	179077016	ABL2	NM_001136000.2	T	G/T	missense	GLN,PRO	1011/1065		3032
A3	4	8034253	ABLIM2	NM_001130083.1	C	A/C	intron	none	NA	NA	
A3	1	1231409	ACAP3	NM_030649.2	T	A/T	coding-synonymous	none	496/835		1488
A3	17	42847435	ADAM11	NM_002390.4	G	G/T	intron	none	NA	NA	
A3	4	175897790	ADAM29	NM_001130703.1	G	G/T	missense	GLY,CYS	372/821		1114
A3	16	77387820	ADAMTS18	NM_199355.2	G	G/T	intron	none	NA	NA	
A3	5	178551913	ADAMTS2	NM_014244.4	C	A/C	intron	none	NA	NA	
A3	15	79082250	ADAMTS7	NM_014272.3	G	G/T	intron	none	NA	NA	
A3	10	1245865	ADARB2	NM_018702.3	G	A/G	intron	none	NA	NA	
A3	7	31146190	ADCYAP1R1	NM_001118.4	G	C/G	coding-synonymous	none	433/469		1299
A3	20	4228494	ADRA1D	NM_000678.3	C	C/T	missense-near-splice	GLY,SER	371/573		1111
A3	11	67052709	ADRBK1	NM_001619.3	C	A/C	intron	none	NA	NA	
A3	2	100266005	AFF3	NM_001025108.1	C	A/C	intron	none	NA	NA	
A3	4	74352590	AFM	NM_001133.2	C	C/T	intron	none	NA	NA	
A3	10	51754359	AGAP6	NM_001077665.2	T	C/T	intron	none	NA	NA	
A3	1	77987614	AK5	NM_012093.3	G	C/G	missense	GLU,GLN	446/537		1336
A3	6	109980406	AKD1	NM_001145128.2	T	C/T	intron	none	NA	NA	
A3	10	5246369	AKR1C4	NM_001818.3	G	A/G	missense	MET,ILE	94/324		282
A3	3	125856694	ALDH1L1	NM_001270364.1	G	A/G	stop-gained	ARG,stop	406/913		1216
A3	3	183961365	ALG3	NM_001006941.2	G	G/T	missense	PRO,GLN	281/391		842
A3	14	78146185	ALKBH1	NM_006020.2	A	A/T	intron	none	NA	NA	
A3	2	233322469	ALPI	NM_001631.3	A	A/T	intron	none	NA	NA	
A3	11	44289182	ALX4	NM_021926.3	G	G/T	intron	none	NA	NA	
A3	3	149488534	ANKUB1	NM_001144960.1	G	G/T	intron	none	NA	NA	
A3	12	5939653	ANO2	NM_020373.2	T	C/T	missense	TYR,CYS	259/999		776
A3	11	26621374	ANO3	NM_031418.2	C	A/C	intron	none	NA	NA	
A3	12	45742283	ANO6	NM_001025356.2	A	A/G	intron	none	NA	NA	
A3	17	34009640	AP2B1	NM_001030006.1	G	G/T	intron	none	NA	NA	
A3	5	115319073	AQPEP	NM_173800.4	T	A/T	missense	ILE,ASN	262/991		785
A3	15	32917635	ARHGAP11A	NM_014783.3	G	A/G	intron	none	NA	NA	
A3	10	99006074	ARHGAP19	NM_001204300.1	C	C/T	coding-synonymous	none	287/466		861
A3	10	24884775	ARHGAP21	NM_020824.3	G	A/G	intron	none	NA	NA	

A3	17	36646848	ARHGAP23	NM_001199417.1	A	A/G	intron	none	NA	NA	
A3	19	47493021	ARHGAP35	NM_004491.4	G	G/T	intron	none	NA	NA	
A3	19	7509142	ARHGEF18	NM_001130955.1	G	G/T	missense	LYS,ASN	283/1174		849
A3	19	7521275	ARHGEF18	NM_001130955.1	G	A/G	missense	VAL,ILE	535/1174		1603
A3	3	9845997	ARPC4	NM_001024959.2	C	C/G	intron	none	NA	NA	
A3	1	177012489	ASTN1	NM_004319.1	G	C/G	intron	none	NA	NA	
A3	3	194150881	ATP13A3	NM_024524.3	G	G/T	coding-synonymous	none	899/1227		2697
A3	3	10428215	ATP2B2	NM_001001331.2	G	C/G	intron	none	NA	NA	
A3	2	71187100	ATP6V1B1	NM_001692.3	C	C/G	stop-gained	TYR,stop	159/514		477
A3	1	47123911	ATPAF1	NM_001042546.2	A	A/C	intron	none	NA	NA	
A3	3	142180838	ATR	NM_001184.3	C	A/C	missense	TRP,LEU	2379/2645		7136
A3	17	42271514	ATXN7L3	NM_001098833.1	C	A/C	utr-3	none	NA	NA	
A3	12	74931999	ATXN7L3B	NM_001136262.1	A	A/C	missense	GLU,ALA	36/98		107
A3	17	47246904	B4GALNT2	NM_001159387.1	A	A/G	coding-synonymous	none	445/507		1335
A3	20	48256228	B4GALT5	NM_004776.3	C	A/C	missense	ASP,TYR	302/389		904
A3	16	88110136	BANP	NM_001173539.1	C	C/T	intron	none	NA	NA	
A3	2	160205459	BAZ2B	NM_013450.2	A	A/G	intron	none	NA	NA	
A3	4	122747026	BBS7	NM_176824.2	C	A/C	missense	ASP,TYR	713/716		2137
A3	3	107492079	BBX	NM_001142568.1	G	G/T	missense	GLY,VAL	504/942		1511
A3	19	45322264	BCAM	NM_001013257.2	C	C/G	intron	none	NA	NA	
A3	20	61869263	BIRC7	NM_022161.2	G	A/G	missense	ASP,ASN	120/281		358
A3	8	22038047	BMP1	NM_001199.3	A	A/T	intron	none	NA	NA	
A3	1	39988219	BMP8A	NM_181809.3	C	C/T	intron	none	NA	NA	
A3	1	92444245	BRDT	NM_001242805.1	A	A/G	intron	none	NA	NA	
A3	10	93723972	BTAF1	NM_003972.2	A	A/G	intron	none	NA	NA	
A3	10	102749015	C10orf2	NM_001163812.1	T	C/T	missense	PHE,LEU	350/583		1048
A3	12	88380205	C12orf50	NM_152589.1	T	A/T	intron	none	NA	NA	
A3	12	97158129	C12orf63	XM_003118942.3	G	C/G	intron	none	NA	NA	
A3	14	90754552	C14orf102	NM_017970.3	T	A/T	intron	none	NA	NA	
A3	14	90755352	C14orf102	NM_017970.3	C	C/T	coding-synonymous	none	789/1165		2367
A3	14	74187921	C14orf43	NM_001043318.1	G	C/G	intron	none	NA	NA	
A3	15	81435928	C15orf26	NM_173528.2	C	C/T	intron	none	NA	NA	
A3	15	74032549	C15orf59	NM_001039614.1	C	C/T	coding-synonymous	none	197/294		591
A3	17	62078574	C17orf72	NM_001164257.1	C	A/C	missense	PRO,HIS	125/190		374
A3	19	40842186	C19orf47	NM_001256440.1	C	A/C	intron	none	NA	NA	
A3	1	244642885	C1orf101	NM_001130957.1	C	C/G	intron	none	NA	NA	
A3	1	170928641	C1orf129	NM_001163629.1	A	A/T	missense	LYS,MET	64/862		191
A3	1	1021247	C1orf159	NM_017891.4	C	A/C	intron	none	NA	NA	
A3	20	2795722	C20orf141	NM_001256538.1	C	A/C	intron	none	NA	NA	
A3	20	34562450	C20orf152	NM_001207076.1	G	A/G	intron	none	NA	NA	
A3	20	61162086	C20orf166	NM_178463.3	C	A/C	intron	none	NA	NA	
A3	20	3312914	C20orf194	NM_001009984.2	T	C/T	intron	none	NA	NA	
A3	3	14802946	C3orf20	NM_001184957.1	T	C/T	intron	none	NA	NA	

A3	5	179070425	C5orf60	NM_001142306.1	C	C/T	intron	none	NA	NA	
A3	5	41158779	C6	NM_000065.2	G	G/T	coding-synonymous	none	655/935		1965
A3	6	2623767	C6orf195	NM_152554.2	G	A/G	missense	PRO,LEU	97/128		290
A3	5	40931024	C7	NM_000587.2	A	A/G	intron	none	NA	NA	
A3	5	40931032	C7	NM_000587.2	G	C/G	intron	none	NA	NA	
A3	5	40934306	C7	NM_000587.2	A	A/G	intron	none	NA	NA	
A3	7	108524462	C7orf66	NM_001024607.1	G	A/G	intron	none	NA	NA	
A3	7	50169456	C7orf72	NM_001161834.2	C	A/C	intron	none	NA	NA	
A3	9	139888333	C9orf142	NM_183241.1	A	A/C	utr-3	none	NA	NA	
A3	19	13563929	CACNA1A	NM_000068.3	A	A/T	intron	none	NA	NA	
A3	9	140878583	CACNA1B	NM_000718.3	G	A/G	intron	none	NA	NA	
A3	12	2613466	CACNA1C	NM_000719.6	G	G/T	intron	none	NA	NA	
A3	1	181741441	CACNA1E	NM_000721.3	G	A/G	intron	none	NA	NA	
A3	3	50417343	CACNA2D2	NM_001005505.1	G	A/G	intron	none	NA	NA	
A3	12	1995357	CACNA2D4	NM_172364.4	A	A/T	intron	none	NA	NA	
A3	2	27461420	CAD	NM_004341.3	G	A/G	missense	ARG,GLN	1661/2226		4982
A3	3	85961698	CADM2	NM_001167674.1	G	G/T	intron	none	NA	NA	
A3	3	85961704	CADM2	NM_001167674.1	A	A/G	intron	none	NA	NA	
A3	11	15098867	CALCB	NM_000728.3	C	A/C	missense	THR,ASN	87/128		260
A3	12	121701550	CAMKK2	NM_001270485.1	T	G/T	intron	none	NA	NA	
A3	3	49898284	CAMKV	NM_024046.3	G	G/T	missense-near-splice	LEU,ILE	214/502		640
A3	11	104897104	CASP1	NM_001223.4	T	C/T	intron	none	NA	NA	
A3	9	69247454	CBWD6	NM_001085457.1	T	C/T	intron	none	NA	NA	
A3	11	124829124	CCDC15	NM_025004.2	G	G/T	missense	LEU,PHE	97/952		291
A3	2	26652583	CCDC164	NM_145038.2	C	C/T	stop-gained	GLN,stop	210/741		628
A3	13	103383662	CCDC168	NM_001146197.1	C	C/G	missense	ARG,THR	6462/7082		19385
A3	4	26490945	CCKAR	NM_000730.2	G	C/G	missense	LEU,VAL	92/429		274
A3	17	34431819	CCL4	NM_002984.2	T	C/T	intron	none	NA	NA	
A3	12	7632425	CD163	NM_004244.5	G	G/T	intron	none	NA	NA	
A3	12	7521452	CD163L1	NM_174941.4	T	A/T	intron	none	NA	NA	
A3	3	112649710	CD200R1	NM_138806.3	G	A/G	intron	none	NA	NA	
A3	1	157803225	CD5L	NM_005894.2	C	A/C	missense	GLY,CYS	266/348		796
A3	17	45234725	CDC27	NM_001114091.1	T	C/T	coding-synonymous	none	167/831		501
A3	10	73572642	CDH23	NM_001171933.1	A	A/G	missense	ILE,VAL	970/1115		2908
A3	5	26903590	CDH9	NM_016279.3	G	C/G	intron	none	NA	NA	
A3	5	26916079	CDH9	NM_016279.3	A	A/G	intron	none	NA	NA	
A3	5	175992592	CDHR2	NM_001171976.1	T	A/T	intron	none	NA	NA	
A3	4	76521586	CDKL2	NM_003948.3	A	A/C	intron	none	NA	NA	
A3	4	76522069	CDKL2	NM_003948.3	T	C/T	intron	none	NA	NA	
A3	16	80667016	CDYL2	NM_152342.2	C	C/T	missense	SER,ASN	245/507		734
A3	22	46805608	CELSR1	NM_014246.1	C	A/C	intron	none	NA	NA	
A3	4	104032071	CENPE	NM_001813.2	T	A/T	missense	LYS,ASN	2546/2702		7638
A3	4	104059458	CENPE	NM_001813.2	C	A/C	intron	none	NA	NA	

A3	12	88454603	CEP290	NM_025114.3	T	A/T	intron	none	NA	NA	
A3	1	203153729	CHI3L1	NM_001276.2	C	C/T	missense	GLY,ARG	97/384		289
A3	3	184102932	CHRD	NM_003741.2	G	G/T	missense	GLY,VAL	575/956		1724
A3	1	240071088	CHRM3	NM_000740.2	G	G/T	missense	ALA,SER	113/591		337
A3	15	32460014	CHRNA7	NM_000746.5	T	C/T	intron	none	NA	NA	
A3	12	120166562	CIT	NM_001206999.1	C	A/C	intron	none	NA	NA	
A3	19	7830395	CLEC4M	NM_001144904.1	T	A/T	intron	none	NA	NA	
A3	2	55445211	CLHC1	NM_001135598.1	T	C/T	intron	none	NA	NA	
A3	11	123065600	CLMP	NM_024769.2	C	C/G	intron	none	NA	NA	
A3	16	57974237	CNGB1	NM_001297.4	G	G/T	intron	none	NA	NA	
A3	8	87751677	CNGB3	NM_019098.4	G	G/T	intron	none	NA	NA	
A3	16	76555235	CNTNAP4	NM_033401.3	T	C/T	intron	none	NA	NA	
A3	2	125204375	CNTNAP5	NM_130773.2	T	A/T	missense	LEU,GLN	260/1307		779
A3	14	31358757	COCH	NM_001135058.1	T	C/T	intron	none	NA	NA	
A3	1	103343639	COL11A1	NM_001190709.1	T	G/T	missense	ASN,THR	1747/1768		5240
A3	9	101751576	COL15A1	NM_001855.3	C	A/C	intron	none	NA	NA	
A3	7	94056605	COL1A2	NM_000089.3	G	G/T	missense	ALA,SER	1089/1367		3265
A3	9	137687013	COL5A1	NM_000093.3	G	C/G	intron	none	NA	NA	
A3	2	189921592	COL5A2	NM_000393.3	A	A/T	intron	none	NA	NA	
A3	3	48630819	COL7A1	NM_000094.3	T	A/T	stop-gained	LYS,stop	164/2945		490
A3	18	346224	COLEC12	NM_130386.2	C	A/C	intron	none	NA	NA	
A3	12	54741818	COPZ1	NM_016057.1	C	C/T	missense	HIS,TYR	144/178		430
A3	17	14095639	COX10	NM_001303.3	A	A/G	intron	none	NA	NA	
A3	19	36642513	COX7A1	NM_001864.2	C	C/T	intron	none	NA	NA	
A3	4	15034741	CPEB2	NM_001177381.1	A	A/G	intron	none	NA	NA	
A3	4	8608463	CPZ	NM_001014447.2	G	A/G	splice-3	none	NA	NA	
A3	1	17271851	CROCC	NM_014675.3	C	C/G	intron	none	NA	NA	
A3	7	151133436	CRYGN	NM_144727.1	G	C/G	intron	none	NA	NA	
A3	22	37326541	CSF2RB	NM_000395.2	C	A/C	missense	SER,ARG	281/898		843
A3	8	2886696	CSMD1	NM_033225.5	G	C/G	intron	none	NA	NA	
A3	8	113967098	CSMD3	NM_052900.2	T	C/T	intron	none	NA	NA	
A3	8	68044426	CSPP1	NM_024790.6	C	C/T	intron	none	NA	NA	
A3	5	11397395	CTNND2	NM_001332.2	C	A/C	intron	none	NA	NA	
A3	11	10789992	CTR9	NM_014633.3	C	C/T	missense	ALA,VAL	688/1174		2063
A3	4	74703255	CXCL6	NM_002993.3	T	C/T	intron	none	NA	NA	
A3	9	105767433	CYLC2	NM_001340.3	T	A/T	missense	SER,THR	174/349		520
A3	19	41355896	CYP2A6	NM_000762.5	T	C/T	intron	none	NA	NA	
A3	19	41382512	CYP2A7	NM_000764.2	G	G/T	missense	PRO,HIS	408/495		1223
A3	7	99315401	CYP3A7	NM_000765.3	G	G/T	intron	none	NA	NA	
A3	8	65527467	CYP7B1	NM_004820.3	G	G/T	intron	none	NA	NA	
A3	11	61505190	DAGLA	NM_006133.2	G	A/G	missense	VAL,MET	516/1043		1546
A3	11	31284495	DCDC1	NM_181807.3	A	A/C	utr-3	none	NA	NA	
A3	11	6653205	DCHS1	NM_003737.2	G	G/T	intron	none	NA	NA	

A3		8	105361189	DCSTAMP	NM_001257317.1	C	A/C	missense	HIS,ASN	137/284		409
A3		12	132623733	DDX51	NM_175066.3	A	A/G	utr-3	none	NA	NA	
A3		9	19334838	DENND4C	NM_017925.4	A	A/G	intron	none	NA	NA	
A3		22	32270229	DEPDC5	NM_001136029.2	C	C/G	intron	none	NA	NA	
A3		7	14216581	DGKB	NM_004080.2	A	A/C	intron	none	NA	NA	
A3	X		50213275	DGKK	NM_001013742.2	G	A/A	missense	PRO,SER	135/1272		403
A3		17	9676258	DHRS7C	NM_001105571.2	G	G/T	intron	none	NA	NA	
A3		10	454974	DIP2C	NM_014974.2	C	C/G	intron	none	NA	NA	
A3		11	83874329	DLG2	NM_001142699.1	G	A/G	intron	none	NA	NA	
A3		10	124345439	DMBT1	NM_004406.2	C	A/A	intron	none	NA	NA	
A3		7	21789434	DNAH11	NM_003777.3	A	A/G	intron	none	NA	NA	
A3		17	7679296	DNAH2	NM_020877.2	C	A/C	intron	none	NA	NA	
A3		5	13811877	DNAH5	NM_001369.2	G	G/T	missense	THR,ASN	2429/4625		7286
A3		5	13871162	DNAH5	NM_001369.2	C	A/C	intron	none	NA	NA	
A3		2	84808112	DNAH6	NM_001370.1	G	A/G	intron	none	NA	NA	
A3		2	85008580	DNAH6	NM_001370.1	G	G/T	intron	none	NA	NA	
A3		6	38705780	DNAH8	NM_001206927.1	A	A/G	intron	none	NA	NA	
A3		1	172050953	DNM3	NM_001136127.1	G	A/G	intron	none	NA	NA	
A3		7	154519415	DPP6	NM_001039350.1	G	G/T	intron	none	NA	NA	
A3		5	31504582	DROSHA	NM_001100412.1	T	A/T	intron	none	NA	NA	
A3	X		100490953	DRP2	NM_001171184.1	G	A/A	utr-5	none	NA	NA	
A3		18	28662887	DSC2	NM_004949.3	C	A/C	intron	none	NA	NA	
A3		20	30452668	DUSP15	NM_001012644.1	T	C/T	intron	none	NA	NA	
A3		1	167086503	DUSP27	NM_001080426.1	A	A/T	intron	none	NA	NA	
A3		1	167095849	DUSP27	NM_001080426.1	G	C/G	missense	ARG,THR	494/1159		1481
A3		17	7132893	DVL2	NM_004422.2	T	C/T	intron	none	NA	NA	
A3		11	103006448	DYNC2H1	NM_001080463.1	G	G/T	splice-3	none	NA	NA	
A3		11	103039373	DYNC2H1	NM_001080463.1	A	A/T	intron	none	NA	NA	
A3		2	44014419	DYNC2LI1	NM_001193464.1	G	A/G	intron	none	NA	NA	
A3		2	71762612	DYSF	NM_001130455.1	A	A/C	intron	none	NA	NA	
A3		13	78470780	EDNRB	NM_000115.3	C	C/T	utr-3	none	NA	NA	
A3		8	144668390	EEF1D	NM_001130053.2	C	A/C	missense-near-splice	GLY,VAL	429/648		1286
A3		14	90397855	EFCAB11	NM_145231.2	G	A/G	intron	none	NA	NA	
A3		8	133015495	EFR3A	NM_015137.4	C	C/T	missense	HIS,TYR	775/822		2323
A3		7	55086863	EGFR	NM_005228.3	C	N/N	utr-5	none	NA	NA	
A3		2	88890375	EIF2AK3	NM_004836.5	T	C/T	coding-synonymous	none	321/1117		963
A3		1	36367952	EIF2C1	NM_012199.2	G	C/G	intron	none	NA	NA	
A3		19	11569298	ELAVL3	NM_001420.3	G	G/T	coding-synonymous	none	154/368		462
A3		13	41507652	ELF1	NM_001145353.1	T	A/T	missense	GLN,LEU	566/596		1697
A3		7	36934405	ELMO1	NM_001039459.2	G	G/T	intron	none	NA	NA	
A3		6	11011159	ELOVL2	NM_017770.3	A	A/G	intron	none	NA	NA	
A3		1	79385863	ELTD1	NM_022159.3	C	A/C	intron	none	NA	NA	
A3		8	109455869	EMC2	NM_014673.3	G	G/T	utr-5	none	NA	NA	

A3	18	2891552	EMILIN2	NM_032048.2	G	A/G	missense	ARG,GLN	476/1054		1427
A3	20	34800310	EPB41L1	NM_001258329.1	C	C/T	intron	none	NA	NA	
A3	3	89462359	EPHA3	NM_005233.5	G	G/T	stop-gained	GLU,stop	611/984		1831
A3	8	144942936	EPPK1	NM_031308.1	C	C/T	missense	ALA,THR	1496/2421		4486
A3	17	62125076	ERN1	NM_001433.3	G	A/G	intron	none	NA	NA	
A3	14	64716250	ESR2	NM_001040275.1	G	A/G	intron	none	NA	NA	
A3	11	128359427	ETS1	NM_001143820.1	T	A/T	intron	none	NA	NA	
A3	4	5699218	EVC2	NM_001166136.1	G	A/G	intron	none	NA	NA	
A3	19	7913872	EVI5L	NM_001159944.1	C	C/T	coding-synonymous	none	131/806		393
A3	14	103573678	EXOC3L4	NM_001077594.1	G	A/G	intron	none	NA	NA	
A3	4	89652458	FAM13A	NM_001015045.2	T	G/T	intron	none	NA	NA	
A3	3	197896920	FAM157A	NM_001145248.1	G	A/G	intron	none	NA	NA	
A3	10	126517954	FAM175B	NM_032182.3	T	C/T	intron	none	NA	NA	
A3	15	29415937	FAM189A1	NM_015307.1	G	G/T	intron	none	NA	NA	
A3	22	48885496	FAM19A5	NM_001082967.1	G	G/T	missense	SER,ILE	31/133		92
A3	15	49868985	FAM227B	NM_152647.2	C	C/G	missense	ASP,HIS	167/509		499
A3	6	116832976	FAM26E	NM_001139444.2	T	A/T	intron	none	NA	NA	
A3	6	116836770	FAM26E	NM_001139444.2	G	A/G	intron	none	NA	NA	
A3	1	190203511	FAM5C	NM_199051.1	G	G/T	missense	GLN,LYS	239/767		715
A3	6	24830955	FAM65B	NM_014722.2	T	C/T	intron	none	NA	NA	
A3	9	90536687	FAM75C1	NM_001145124.1	C	C/T	missense	THR,ILE	622/1189		1865
A3	2	163039878	FAP	NM_004460.2	G	G/T	intron	none	NA	NA	
A3	11	13749097	FAR1	NM_032228.5	A	A/C	intron	none	NA	NA	
A3	3	13663411	FBLN2	NM_001004019.1	C	A/C	stop-gained	CYS,stop	764/1232		2292
A3	5	127686559	FBN2	NM_001999.3	C	C/T	splice-5	none	NA	NA	
A3	12	117624403	FBXO21	NM_015002.2	C	A/C	intron	none	NA	NA	
A3	11	72696029	FCHSD2	NM_014824.2	C	A/C	intron	none	NA	NA	
A3	1	157665297	FCRL3	NM_052939.3	G	A/G	coding-synonymous	none	411/735		1233
A3	15	68582542	FEM1B	NM_015322.3	G	A/G	coding-synonymous	none	282/628		846
A3	11	125315884	FEZ1	NM_005103.4	T	C/T	utr-3	none	NA	NA	
A3	3	14860589	FGD5	NM_152536.3	G	G/T	missense	GLY,VAL	4/1463		11
A3	5	44310459	FGF10	NM_004465.1	G	C/G	intron	none	NA	NA	
A3	6	167446086	FGFR1OP	NM_007045.2	A	A/T	intron	none	NA	NA	
A3	14	86089608	FLRT2	NM_013231.4	C	C/T	missense	ARG,TRP	584/661		1750
A3	1	240371223	FMN2	NM_020066.4	C	C/T	coding-synonymous	none	1037/1723		3111
A3	1	240371970	FMN2	NM_020066.4	C	A/C	coding-synonymous	none	1286/1723		3858
A3	14	29237403	FOXG1	NM_005249.4	C	A/C	coding-synonymous	none	306/490		918
A3	9	85964812	FRMD3	NM_001244959.1	C	A/C	intron	none	NA	NA	
A3	14	31055982	G2E3	NM_017769.3	T	G/T	coding-synonymous	none	32/707		96
A3	9	101258882	GABBR2	NM_005458.7	T	A/T	intron	none	NA	NA	
A3	15	26825465	GABRB3	NM_000814.5	C	A/C	splice-5	none	NA	NA	
A3	15	27765288	GABRG3	NM_033223.4	T	G/T	intron	none	NA	NA	
A3	6	89910941	GABRR1	NM_001256703.1	T	C/T	missense	THR,ALA	56/463		166

A3	14	88406452	GALC	NM_000153.3	C	A/C	intron	none	NA	NA
A3	2	31155113	GALNT14	NM_001253826.1	T	A/T	intron	none	NA	NA
A3	11	62400863	GANAB	NM_198334.1	C	A/C	intron	none	NA	NA
A3	19	36035787	GAPDHS	NM_014364.4	T	A/T	intron	none	NA	NA
A3	15	45658247	GATM	NM_001482.2	G	G/T	missense	HIS,GLN	325/424	975
A3	4	72611841	GC	NM_000583.3	G	G/T	intron	none	NA	NA
A3	8	21560441	GFRA2	NM_001165038.1	G	G/T	missense	PRO,GLN	260/360	779
A3	20	33433309	GGT7	NM_178026.2	C	A/C	intron	none	NA	NA
A3	7	150438102	GIMAP5	NM_001199577.1	G	G/T	intron	none	NA	NA
A3	4	80328431	GK2	NM_033214.2	T	A/T	coding-synonymous	none	308/554	924
A3	6	49494382	GLYATL3	NM_001010904.1	C	C/T	stop-gained	GLN,stop	208/289	622
A3	1	167038461	GPA33	NM_005814.1	A	A/C	intron	none	NA	NA
A3	12	110895454	GPN3	NM_001164372.1	T	C/T	intron	none	NA	NA
A3	2	26538424	GPR113	NM_001145168.1	G	G/T	coding-synonymous	none	296/1080	888
A3	12	131488869	GPR133	NM_198827.3	C	A/C	intron	none	NA	NA
A3	4	158238934	GRIA2	NM_000826.3	C	C/T	intron	none	NA	NA
A3	X	122599397	GRIA3	NM_000828.4	A	G/G	intron	none	NA	NA
A3	1	37335273	GRIK3	NM_000831.3	C	A/C	intron	none	NA	NA
A3	7	126542591	GRM8	NM_000845.2	G	G/T	intron	none	NA	NA
A3	11	18380134	GTF2H1	NM_001142307.1	C	C/T	coding-synonymous	none	472/549	1414
A3	11	106849154	GUCY1A2	NM_000855.2	A	A/T	intron	none	NA	NA
A3	19	547310	GZMM	NM_001258351.1	G	C/G	utr-5	none	NA	NA
A3	5	82969285	HAPLN1	NM_001884.3	C	A/C	missense	ASP,TYR	20/355	58
A3	11	5269511	HBG1	NM_000559.2	C	C/T	utr-3	none	NA	NA
A3	2	240098010	HDAC4	NM_006037.3	T	C/T	intron	none	NA	NA
A3	15	50545870	HDC	NM_002112.3	G	G/T	intron	none	NA	NA
A3	14	73965039	HEATR4	NM_001220484.1	T	G/T	missense	GLN,PRO	789/1027	2366
A3	5	41034109	HEATR7B2	NM_173489.4	G	G/T	intron	none	NA	NA
A3	7	43283364	HECW1	NM_015052.3	C	A/C	intron	none	NA	NA
A3	15	63926222	HERC1	NM_003922.3	C	A/C	intron	none	NA	NA
A3	7	35673563	HERPUD2	NM_022373.4	G	A/G	intron	none	NA	NA
A3	1	91731478	HFM1	NM_001017975.3	G	A/G	intron	none	NA	NA
A3	8	133111179	HHLA1	NM_001145095.1	G	A/G	missense	ALA,VAL	77/532	230
A3	6	27799167	HIST1H4K	NM_003541.2	T	A/T	missense	ILE,PHE	47/104	139
A3	1	185939689	HMCN1	NM_031935.2	T	C/T	intron	none	NA	NA
A3	7	27182657	HOXA5	NM_019102.3	G	A/G	intron	none	NA	NA
A3	11	113779994	HTR3B	NM_006028.4	C	A/C	intron	none	NA	NA
A3	4	3123155	HTT	NM_002111.6	T	G/T	coding-synonymous	none	423/3143	1269
A3	14	76525753	IFT43	NM_001102564.1	G	G/T	intron	none	NA	NA
A3	3	118647545	IGSF11	NM_001015887.1	C	A/C	stop-gained	GLY,stop	79/432	235
A3	3	118753419	IGSF11	NM_001015887.1	T	A/T	missense	GLN,LEU	4/432	11
A3	11	18735881	IGSF22	NM_173588.3	C	A/C	missense	GLY,CYS	581/1327	1741
A3	1	117120207	IGSF3	NM_001007237.1	C	A/C	intron	none	NA	NA

A3	12	68646516	IL22	NM_020525.4	G	A/G	intron	none	NA	NA	
A3	1	67635164	IL23R	NM_144701.2	C	C/T	coding-synonymous	none	70/630		210
A3	3	3116531	IL5RA	NM_000564.4	A	A/G	coding-synonymous	none	371/421		1111
A3	3	100948467	IMPG2	NM_016247.3	G	A/G	intron	none	NA	NA	
A3	1	62231924	INADL	NM_176877.2	T	A/T	intron	none	NA	NA	
A3	12	57828911	INHBC	NM_005538.2	G	A/G	missense	GLY,GLU	81/353		242
A3	1	38348406	INPP5B	NM_005540.2	T	C/T	intron	none	NA	NA	
A3	10	121551787	INPP5F	NM_014937.3	G	A/G	intron	none	NA	NA	
A3	7	155095658	INSIG1	NM_005542.4	A	A/T	intron	none	NA	NA	
A3	19	7166398	INSR	NM_000208.2	G	A/G	missense	THR,MET	543/1383		1628
A3	4	128635109	INTU	NM_015693.3	A	A/G	missense	SER,GLY	860/943		2578
A3	3	158983119	IQCJ	NM_001042705.2	C	C/G	missense	ALA,GLY	136/160		407
A3	7	123101440	IQUB	NM_178827.4	C	C/T	missense	ASP,ASN	660/792		1978
A3	12	66638740	IRAK3	NM_001142523.1	G	G/T	missense	VAL,PHE	306/536		916
A3	5	1880713	IRX4	NM_016358.2	C	C/T	intron	none	NA	NA	
A3	3	37574718	ITGA9	NM_002207.2	A	A/T	intron	none	NA	NA	
A3	10	7763715	ITIH2	NM_002216.2	G	C/G	missense	ARG,THR	281/947		842
A3	10	7615225	ITIH5	NM_001001851.2	G	A/G	intron	none	NA	NA	
A3	3	4715766	ITPR1	NM_001099952.2	A	A/T	intron	none	NA	NA	
A3	8	75233316	JPH1	NM_020647.2	C	A/C	coding-synonymous	none	69/662		207
A3	3	124113367	KALRN	NM_001024660.3	T	A/T	intron	none	NA	NA	
A3	2	97278699	KANSL3	NM_001115016.2	C	A/C	intron	none	NA	NA	
A3	2	170371002	KBTBD10	NM_006063.2	A	A/C	intron	none	NA	NA	
A3	11	30032691	KCNA4	NM_002233.3	G	G/T	missense	PRO,GLN	512/654		1535
A3	20	49620875	KCNG1	NM_002237.3	A	A/C	missense	TYR,ASP	415/514		1243
A3	14	63453879	KCNH5	NM_139318.3	G	G/T	coding-synonymous	none	154/989		460
A3	10	78729625	KCNMA1	NM_001014797.2	G	G/T	intron	none	NA	NA	
A3	9	138650472	KCNT1	NM_020822.2	C	C/T	intron	none	NA	NA	
A3	9	138675851	KCNT1	NM_020822.2	G	G/T	intron	none	NA	NA	
A3	1	196288683	KCNT2	NM_198503.2	G	A/G	missense	PRO,LEU	760/1136		2279
A3	8	36665010	KCNU1	NM_001031836.2	G	G/T	intron	none	NA	NA	
A3	1	202724575	KDM5B	NM_006618.3	A	A/T	stop-gained	TYR,stop	454/1545		1362
A3	12	22697178	KIAA0528	NM_014802.1	C	A/C	intron	none	NA	NA	
A3	16	27789308	KIAA0556	NM_015202.2	G	C/G	intron	none	NA	NA	
A3	1	155903326	KIAA0907	NM_014949.2	C	A/C	intron	none	NA	NA	
A3	5	5462512	KIAA0947	NM_015325.2	G	C/G	missense	GLY,ALA	1022/2267		3065
A3	4	57193841	KIAA1211	NM_020722.1	G	G/T	coding-synonymous	none	1191/1234		3573
A3	2	99439470	KIAA1211L	NM_207362.2	T	A/T	coding-synonymous	none	422/963		1266
A3	18	59942600	KIAA1468	NM_020854.3	C	C/T	intron	none	NA	NA	
A3	1	33237091	KIAA1522	NM_001198972.1	C	A/C	missense	PRO,THR	712/1036		2134
A3	1	233482283	KIAA1804	NM_032435.2	A	A/C	missense	LYS,GLN	301/1037		901
A3	17	72341085	KIF19	NM_153209.3	C	C/T	coding-synonymous	none	256/999		768
A3	2	241708490	KIF1A	NM_001244008.1	A	A/T	intron	none	NA	NA	

A3	14	104640584	KIF26A	NM_015656.1	A	A/G	coding-synonymous	none	710/1883		2130
A3	9	86485622	KIF27	NM_017576.1	C	C/T	intron	none	NA	NA	
A3	1	169951059	KIFAP3	NM_001204514.1	T	G/T	intron	none	NA	NA	
A3	7	129765652	KLHDC10	NM_014997.3	A	A/T	intron	none	NA	NA	
A3	13	70370830	KLHL1	NM_020866.2	A	A/T	intron	none	NA	NA	
A3	4	166226883	KLHL2	NM_001161521.1	C	C/T	intron	none	NA	NA	
A3	19	51411676	KLK4	NM_004917.3	G	G/T	missense	PRO,GLN	184/255		551
A3	7	149420833	KRBA1	NM_032534.2	G	G/T	stop-gained	GLU,stop	261/1031		781
A3	2	48925889	LHCGR	NM_000233.3	G	G/T	missense	SER,TYR	244/700		731
A3	2	48936276	LHCGR	NM_000233.3	G	G/T	intron	none	NA	NA	
A3	19	55144678	LILRB1	NM_001081637.1	C	A/C	coding-synonymous	none	390/653		1170
A3	19	54782146	LILRB2	NM_001080978.2	T	A/T	missense	HIS,LEU	409/598		1226
A3	19	51885631	LIM2	NM_001161748.1	G	G/T	intron	none	NA	NA	
A3	12	49498371	LMBR1L	NM_018113.2	G	A/G	intron	none	NA	NA	
A3	5	36104136	LMBRD2	NM_001007527.1	C	C/G	utr-3	none	NA	NA	
A3	1	87797844	LMO4	NM_006769.3	A	A/C	missense	LYS,THR	49/166		146
A3	4	62936624	LPHN3	NM_015236.4	T	C/T	stop-lost	stop,GLN	1470/1470		4408
A3	17	56345081	LPO	NM_001160102.1	C	A/C	intron	none	NA	NA	
A3	X	114405013	LRCH2	NM_001243963.1	A	G/G	intron	none	NA	NA	
A3	3	66463390	LRIG1	NM_015541.2	C	C/G	missense	GLN,HIS	232/1094		696
A3	12	57593585	LRP1	NM_002332.2	C	C/T	intron	none	NA	NA	
A3	2	170013889	LRP2	NM_004525.2	G	G/T	missense	SER,TYR	4004/4656		12011
A3	2	170099607	LRP2	NM_004525.2	A	A/G	intron	none	NA	NA	
A3	4	186288294	LRP2BP	NM_018409.3	G	A/G	utr-3	none	NA	NA	
A3	1	70478652	LRRC7	NM_020794.2	T	A/T	intron	none	NA	NA	
A3	7	91774221	LRRD1	NM_001161528.1	G	G/T	missense	THR,LYS	855/861		2564
A3	12	40631920	LRRK2	NM_198578.3	C	C/G	intron	none	NA	NA	
A3	12	40645032	LRRK2	NM_198578.3	A	A/G	splice-3	none	NA	NA	
A3	11	47306485	MADD	NM_001135943.1	C	A/C	intron	none	NA	NA	
A3	19	35800926	MAG	NM_001199216.1	G	A/G	missense	VAL,MET	436/602		1306
A3	4	6594789	MAN2B2	NM_015274.1	G	G/T	intron	none	NA	NA	
A3	6	100390726	MCHR2	NM_001040179.1	C	A/C	intron	none	NA	NA	
A3	8	48882470	MCM4	NM_005914.3	G	A/G	coding-synonymous	none	429/864		1287
A3	11	86157441	ME3	NM_001014811.1	G	G/T	missense	ALA,ASP	490/605		1469
A3	1	171753178	METTL13	NM_001007239.1	A	A/C	missense-near-splic	GLN,PRO	151/544		452
A3	15	89444909	MFGE8	NM_001114614.1	G	A/G	missense	ALA,VAL	248/336		743
A3	1	205568123	MFSD4	NM_181644.4	C	A/C	intron	none	NA	NA	
A3	17	4793806	MINK1	NM_001024937.3	G	G/T	intron	none	NA	NA	
A3	3	69988436	MITF	NM_000248.3	T	G/T	intron	none	NA	NA	
A3	8	89086827	MMP16	NM_005941.4	C	C/G	intron	none	NA	NA	
A3	11	102706833	MMP3	NM_002422.3	T	C/T	utr-3	none	NA	NA	
A3	6	39893587	MOCS1	NM_001075098.3	G	C/G	missense	GLN,GLU	85/386		253
A3	7	24681389	MPP6	NM_016447.2	G	G/T	stop-gained	GLU,stop	58/541		172

A3	17	17050413	MPRIP	NM_015134.3	G	C/G	intron	none	NA	NA	
A3	11	68667971	MRPL21	NM_181514.1	C	A/C	intron	none	NA	NA	
A3	3	49723739	MST1	NM_020998.3	A	A/C	intron	none	NA	NA	
A3	14	105933073	MTA1	NM_001203258.1	A	A/T	utr-3	none	NA	NA	
A3	6	151358205	MTHFD1L	NM_001242767.1	C	C/T	coding-synonymous	none	934/980		2802
A3	6	151358213	MTHFD1L	NM_001242767.1	C	C/T	missense	ALA,VAL	937/980		2810
A3	7	100652262	MUC12	NM_001164462.1	T	A/T	intron	none	NA	NA	
A3	19	8973814	MUC16	NM_024690.2	G	A/G	intron	none	NA	NA	
A3	3	195456635	MUC20	NM_001098516.1	G	G/T	intron	none	NA	NA	
A3	3	195506364	MUC4	NM_004532.5	G	C/G	intron	none	NA	NA	
A3	3	195507379	MUC4	NM_004532.5	G	C/G	intron	none	NA	NA	
A3	11	1023420	MUC6	NM_005961.2	A	A/G	intron	none	NA	NA	
A3	19	50726201	MYH14	NM_001077186.1	G	C/G	intron	none	NA	NA	
A3	19	50810500	MYH14	NM_001077186.1	T	A/T	intron	none	NA	NA	
A3	17	10424507	MYH2	NM_001100112.1	G	G/T	utr-3	none	NA	NA	
A3	14	23851272	MYH6	NM_002471.3	G	C/G	missense-near-splice	GLN,GLU	1933/1940		5797
A3	17	10310326	MYH8	NM_002472.2	A	A/G	intron	none	NA	NA	
A3	2	171243611	MYO3B	NM_001083615.3	C	A/C	intron	none	NA	NA	
A3	6	76589468	MYO6	NM_004999.3	A	A/T	intron	none	NA	NA	
A3	15	72142280	MYO9A	NM_006901.3	A	A/T	intron	none	NA	NA	
A3	8	2005754	MYOM2	NM_003970.2	T	G/T	missense	LEU,ARG	139/1466		416
A3	13	101728282	NALCN	NM_052867.2	G	C/G	missense	THR,SER	1299/1739		3896
A3	17	80438855	NARF	NM_001038618.2	C	C/G	intron	none	NA	NA	
A3	18	55280969	NARS	NM_004539.3	T	A/T	intron	none	NA	NA	
A3	11	20057524	NAV2	NM_001111018.1	G	C/G	missense	VAL,LEU	866/2366		2596
A3	1	145297544	NBPF10	NM_001039703.4	T	C/T	intron	none	NA	NA	
A3	1	145299981	NBPF10	NM_001039703.4	A	A/G	intron	none	NA	NA	
A3	12	54925234	NCKAP1L	NM_001184976.1	C	C/T	intron	none	NA	NA	
A3	12	124904693	NCOR2	NM_001077261.3	C	C/T	intron	none	NA	NA	
A3	18	2599124	NDC80	NM_006101.2	C	C/T	missense	PRO,LEU	443/643		1328
A3	16	15781348	NDE1	NM_001143979.1	G	A/G	missense	ARG,LYS	168/336		503
A3	8	91884296	NECAB1	NM_022351.4	A	A/C	intron	none	NA	NA	
A3	8	91961873	NECAB1	NM_022351.4	T	A/T	intron	none	NA	NA	
A3	6	11306100	NEDD9	NM_001142393.1	A	A/T	intron	none	NA	NA	
A3	4	170512000	NEK1	NM_001199397.1	C	C/T	intron	none	NA	NA	
A3	16	68157000	NFATC3	NM_004555.3	A	A/G	missense	LYS,ARG	405/1069		1214
A3	4	48037572	NIPAL1	NM_207330.1	C	C/G	intron	none	NA	NA	
A3	16	3599306	NLRC3	NM_178844.2	T	C/T	intron	none	NA	NA	
A3	1	880127	NOC2L	NM_015658.3	C	A/C	missense	ALA,SER	733/750		2197
A3	7	156752755	NOM1	NM_138400.1	A	A/C	missense	LYS,GLN	507/861		1519
A3	1	31769687	none	none	C	A/C	near-gene-5	none	NA	NA	
A3	1	35686257	none	none	C	C/T	intergenic	none	NA	NA	
A3	1	158576050	none	none	G	G/T	near-gene-5	none	NA	NA	

A3	2	8357293	none	none	T	A/T	intergenic	none	NA	NA
A3	2	89160391	none	none	C	C/T	intergenic	none	NA	NA
A3	2	90121522	none	none	C	A/C	intergenic	none	NA	NA
A3	2	90211565	none	none	G	C/G	intergenic	none	NA	NA
A3	2	90211601	none	none	G	G/T	intergenic	none	NA	NA
A3	2	232374013	none	none	G	G/T	intergenic	none	NA	NA
A3	3	75680079	none	none	G	G/T	intergenic	none	NA	NA
A3	6	7986306	none	none	G	A/G	intergenic	none	NA	NA
A3	7	72338156	none	none	A	A/G	intergenic	none	NA	NA
A3	7	72338316	none	none	T	A/T	intergenic	none	NA	NA
A3	7	89581872	none	none	C	C/G	intergenic	none	NA	NA
A3	7	89975939	none	none	G	G/T	near-gene-5	none	NA	NA
A3	7	143771294	none	none	T	C/T	near-gene-5	none	NA	NA
A3	7	153109949	none	none	A	A/G	intergenic	none	NA	NA
A3	7	153110050	none	none	C	C/G	intergenic	none	NA	NA
A3	8	97247888	none	none	C	A/C	near-gene-5	none	NA	NA
A3	9	21471603	none	none	T	C/T	intergenic	none	NA	NA
A3	9	118777426	none	none	T	C/T	intergenic	none	NA	NA
A3	11	48267582	none	none	T	C/T	near-gene-3	none	NA	NA
A3	11	55872401	none	none	C	A/C	near-gene-5	none	NA	NA
A3	11	89486274	none	none	C	A/C	intergenic	none	NA	NA
A3	11	124310033	none	none	C	C/T	near-gene-3	none	NA	NA
A3	12	131155228	none	none	C	A/C	intergenic	none	NA	NA
A3	14	22689801	none	none	G	C/G	intergenic	none	NA	NA
A3	14	23019414	none	none	C	C/T	intergenic	none	NA	NA
A3	14	106366451	none	none	G	C/G	intergenic	none	NA	NA
A3	14	106667426	none	none	T	A/T	intergenic	none	NA	NA
A3	15	20657730	none	none	G	A/G	intergenic	none	NA	NA
A3	19	22605181	none	none	C	A/C	near-gene-5	none	NA	NA
A3	19	55049118	none	none	T	C/T	intergenic	none	NA	NA
A3	20	4713362	none	none	G	A/G	intergenic	none	NA	NA
A3	20	26049667	none	none	T	A/T	intergenic	none	NA	NA
A3	20	45159005	none	none	T	C/T	intergenic	none	NA	NA
A3	21	19776014	none	none	C	C/T	near-gene-5	none	NA	NA
A3	22	17395441	none	none	C	A/C	intergenic	none	NA	NA
A3	17	45668303	NPEPPS	NM_006310.3	C	C/T	intron	none	NA	NA
A3	5	32783146	NPR3	NM_000908.3	G	A/G	intron	none	NA	NA
A3	4	149116106	NR3C2	NM_000901.4	T	A/T	intron	none	NA	NA
A3	9	102590501	NR4A3	NM_006981.3	T	C/T	coding-synonymous	none	59/627	177
A3	7	129394899	NRF1	NM_001040110.1	A	A/T	missense	THR,SER	464/504	1390
A3	2	51149877	NRXN1	NM_001135659.1	G	C/G	intron	none	NA	NA
A3	5	176694614	NSD1	NM_022455.4	G	G/T	missense	CYS,PHE	1733/2697	5198
A3	12	104187893	NT5DC3	NM_001031701.2	C	A/C	intron	none	NA	NA

A3	12	5603345	NTF3	NM_001102654.1	G	G/T	intron	none	NA	NA	
A3	1	156849109	NTRK1	NM_001007792.1	T	A/T	coding-synonymous	none	631/761		1893
A3	1	156785989	NTRK1	NM_001007792.1	C	A/C	intron	none	NA	NA	
A3	5	37351390	NUP155	NM_004298.2	G	G/T	missense	PRO,THR	150/1333		448
A3	1	228465587	OBSCN	NM_001098623.1	G	G/T	intron	none	NA	NA	
A3	1	228497196	OBSCN	NM_001098623.1	G	A/G	coding-synonymous	none	4316/7969		12948
A3	1	228543749	OBSCN	NM_001098623.1	G	G/T	intron	none	NA	NA	
A3	2	10581806	ODC1	NM_002539.1	C	C/T	missense	GLY,GLU	357/462		1070
A3	10	50943396	OGDHL	NM_001143996.1	A	A/T	missense-near-splice	TYR,ASN	914/954		2740
A3	1	102269978	OLFM3	NM_058170.2	T	C/T	missense	TYR,CYS	398/459		1193
A3	13	53602994	OLFM4	NM_006418.4	T	A/T	missense	LEU,HIS	8/511		23
A3	1	247614913	OR2B11	NM_001004492.1	C	A/C	coding-synonymous	none	124/318		372
A3	1	248201558	OR2L2	NM_001004686.2	T	C/T	utr-5	none	NA	NA	
A3	1	248202526	OR2L2	NM_001004686.2	G	C/G	utr-3	none	NA	NA	
A3	11	55135678	OR4A15	NM_001005275.1	C	A/C	missense	PRO,THR	107/345		319
A3	14	20444320	OR4K15	NM_001005486.1	G	C/G	missense	ASP,HIS	215/349		643
A3	11	5565854	OR52H1	NM_001005289.1	G	A/G	coding-synonymous	none	300/321		900
A3	11	5809909	OR52N1	NM_001001913.1	G	G/T	coding-synonymous	none	46/321		138
A3	11	58207643	OR5B12	NM_001004733.2	C	A/C	utr-5	none	NA	NA	
A3	12	55523699	OR9K2	NM_001005243.1	C	A/C	missense	HIS,GLN	49/336		147
A3	2	26683483	OTOF	NM_004802.3	G	A/G	intron	none	NA	NA	
A3	2	26760487	OTOF	NM_194248.2	G	G/T	intron	none	NA	NA	
A3	4	4199402	OTOP1	NM_177998.1	G	G/T	missense	ARG,SER	387/613		1159
A3	11	73988086	P4HA3	NM_182904.3	G	G/T	missense	GLN,LYS	427/545		1279
A3	1	17685496	PADI4	NM_012387.2	G	G/T	intron	none	NA	NA	
A3	14	97001003	PAPOLA	NM_001252006.1	G	A/G	utr-3	none	NA	NA	
A3	1	176680997	PAPPA2	NM_020318.2	A	A/T	missense	LEU,PHE	1226/1792		3678
A3	14	20813694	PARP2	NM_001042618.1	G	C/G	intron	none	NA	NA	
A3	21	47767236	PCNT	NM_006031.5	C	C/G	intron	none	NA	NA	
A3	9	78710491	PCSK5	NM_001190482.1	G	G/T	intron	none	NA	NA	
A3	9	78710897	PCSK5	NM_001190482.1	G	A/G	missense	SER,ASN	329/1861		986
A3	11	72290512	PDE2A	NM_001143839.3	C	A/C	intron	none	NA	NA	
A3	12	20786627	PDE3A	NM_000921.4	C	A/C	missense-near-splice	SER,ARG	587/1142		1761
A3	19	18330224	PDE4C	NM_000923.4	G	A/G	intron	none	NA	NA	
A3	21	44182246	PDE9A	NM_001001567.1	C	A/C	missense	PRO,GLN	320/534		959
A3	7	95221694	PDK4	NM_002612.3	G	C/G	intron	none	NA	NA	
A3	7	92148198	PEX1	NM_000466.2	T	A/T	intron	none	NA	NA	
A3	4	37857327	PGM2	NM_018290.3	G	C/G	missense	LYS,ASN	567/613		1701
A3	11	118520719	PHLDB1	NM_001144758.2	G	A/G	intron	none	NA	NA	
A3	22	21064803	PI4KA	NM_002650.2	T	C/T	intron	none	NA	NA	
A3	17	16120796	PIGL	NM_004278.3	C	A/C	intron	none	NA	NA	
A3	1	207112476	PIGR	NM_002644.3	C	A/C	stop-gained	GLU,stop	126/765		376
A3	10	3212296	PITRM1	NM_001242307.1	C	A/C	missense-near-splice	GLN,HIS	53/1039		159

A3	16	72001727	PKD1L3	NM_181536.1	C	C/G	intron	none	NA	NA	
A3	8	110412269	PKHD1L1	NM_177531.4	C	A/C	intron	none	NA	NA	
A3	8	110519795	PKHD1L1	NM_177531.4	A	A/T	intron	none	NA	NA	
A3	16	81953096	PLCG2	NM_002661.3	G	G/T	missense	GLY,CYS	688/1266		2062
A3	1	16054780	PLEKHM2	NM_015164.2	G	G/T	missense	GLY,TRP	617/1020		1849
A3	19	4511223	PLIN4	NM_001080400.1	C	C/T	missense	VAL,MET	903/1358		2707
A3	19	50369626	PNKP	NM_007254.3	C	A/C	intron	none	NA	NA	
A3	10	118196194	PNLIPRP3	NM_001011709.2	T	C/T	intron	none	NA	NA	
A3	14	39646810	PNN	NM_002687.3	C	A/C	missense	SER,TYR	114/718		341
A3	3	121187380	POLQ	NM_199420.3	A	A/G	intron	none	NA	NA	
A3	10	79745109	POLR3A	NM_007055.3	A	A/C	intron	none	NA	NA	
A3	7	39046571	POU6F2	NM_001166018.1	C	A/C	intron	none	NA	NA	
A3	4	23814614	PPARGC1A	NM_013261.3	G	G/T	intron	none	NA	NA	
A3	1	12919240	PRAMEF2	NM_023014.1	C	C/T	intron	none	NA	NA	
A3	1	12942840	PRAMEF4	NM_001009611.2	G	A/G	intron	none	NA	NA	
A3	5	23509762	PRDM9	NM_020227.2	T	C/T	intron	none	NA	NA	
A3	5	23527725	PRDM9	NM_020227.2	A	A/G	missense	ASN,SER	843/895		2528
A3	20	47364426	PREX1	NM_020820.3	G	C/G	intron	none	NA	NA	
A3	8	68999897	PREX2	NM_024870.2	C	C/T	intron	none	NA	NA	
A3	7	151552179	PRKAG2	NM_016203.3	T	C/T	intron	none	NA	NA	
A3	17	64524051	PRKCA	NM_002737.2	C	C/T	intron	none	NA	NA	
A3	4	16024946	PROM1	NM_001145847.1	T	A/T	intron	none	NA	NA	
A3	10	25140290	PRTFDC1	NM_020200.5	G	A/G	intron	none	NA	NA	
A3	10	104175964	PSD	NM_002779.3	C	C/G	intron	none	NA	NA	
A3	14	90735723	PSMC1	NM_002802.2	C	C/T	intron	none	NA	NA	
A3	8	97316438	PTDSS1	NM_014754.1	A	A/T	intron	none	NA	NA	
A3	2	209309726	PTH2R	NM_005048.2	C	A/C	intron	none	NA	NA	
A3	1	198704138	PTPRC	NM_002838.4	A	A/T	intron	none	NA	NA	
A3	1	44069828	PTPRF	NM_002840.3	G	G/T	missense	SER,ILE	1002/1908		3005
A3	11	48181557	PTPRJ	NM_002843.3	A	A/G	missense	ILE,VAL	1172/1338		3514
A3	7	157929196	PTPRN2	NM_002847.3	T	C/T	intron	none	NA	NA	
A3	7	157985141	PTPRN2	NM_002847.3	C	A/C	missense	GLY,CYS	143/1016		427
A3	12	57677714	R3HDM2	NM_014925.3	C	A/C	missense	ARG,LEU	341/977		1022
A3	1	229438753	RAB4A	NM_004578.2	C	C/T	intron	none	NA	NA	
A3	7	66236972	RABGEF1	NM_014504.2	C	C/G	missense	PRO,ARG	29/492		86
A3	9	134501198	RAPGEF1	NM_005312.2	T	C/T	intron	none	NA	NA	
A3	20	35632248	RBL1	NM_002895.2	G	C/G	missense	PRO,ALA	965/1069		2893
A3	20	55967790	RBM38	NM_017495.5	G	G/T	coding-synonymous	none	106/240		318
A3	2	87205263	RGPD1	NM_001024457.3	T	C/T	intron	none	NA	NA	
A3	1	241100063	RGS7	NM_002924.4	T	G/T	intron	none	NA	NA	
A3	19	33490429	RHPN2	NM_033103.4	G	G/T	intron	none	NA	NA	
A3	12	107245339	RIC8B	NM_018157.2	G	G/T	missense	ALA,SER	425/521		1273
A3	2	152273516	RIF1	NM_001177663.1	G	G/T	intron	none	NA	NA	

A3	6	72922882	RIMS1	NM_001168407.1	T	A/T	coding-synonymous	none	19/1013	57
A3	8	104922225	RIMS2	NM_001100117.2	T	A/T	intron	none	NA	NA
A3	18	40503661	RIT2	NM_002930.2	A	A/T	missense	VAL,ASP	101/218	302
A3	1	182544842	RNASEL	NM_021133.3	T	A/T	intron	none	NA	NA
A3	7	5751380	RNF216	NM_207111.3	G	G/T	intron	none	NA	NA
A3	18	13740097	RNMT	NM_003799.1	A	A/T	intron	none	NA	NA
A3	9	94616237	ROR2	NM_004560.3	G	A/G	intron	none	NA	NA
A3	10	92634752	RPP30	NM_001104546.1	G	A/G	intron	none	NA	NA
A3	14	75376696	RPS6KL1	NM_031464.4	C	A/C	missense	ASP,TYR	274/550	820
A3	19	54710219	RPS9	NM_001013.3	G	G/T	missense	GLY,VAL	99/195	296
A3	17	78935145	RPTOR	NM_001163034.1	G	G/T	intron	none	NA	NA
A3	2	74667118	RTKN	NM_001015055.1	A	A/C	intron	none	NA	NA
A3	3	187419712	RTP2	NM_001004312.2	C	A/C	intron	none	NA	NA
A3	19	39034441	RYR1	NM_000540.2	G	G/T	missense	ALA,SER	3980/5039	11938
A3	1	237794631	RYR2	NM_001035.2	C	A/C	intron	none	NA	NA
A3	1	237919714	RYR2	NM_001035.2	A	A/T	intron	none	NA	NA
A3	15	34147114	RYR3	NM_001036.3	G	A/G	splice-5	none	NA	NA
A3	1	153516367	S100A4	NM_002961.2	C	C/T	coding-synonymous	none	58/102	174
A3	11	18108730	SAAL1	NM_138421.2	T	C/T	coding-synonymous	none	271/475	813
A3	18	76753438	SALL3	NM_171999.3	C	C/G	missense	PRO,ALA	483/1301	1447
A3	3	38781037	SCN10A	NM_006514.2	C	A/C	missense	GLY,VAL	750/1957	2249
A3	2	166850678	SCN1A	NM_001165963.1	C	A/C	coding-synonymous	none	1610/2010	4830
A3	17	62021340	SCN4A	NM_000334.4	G	G/T	intron	none	NA	NA
A3	3	38645204	SCN5A	NM_000335.4	G	G/T	missense-near-splic	THR,LYS	630/2016	1889
A3	8	97605692	SDC2	NM_002998.3	G	G/T	intron	none	NA	NA
A3	9	139357349	SEC16A	NM_014866.1	C	C/T	missense	GLY,ASP	1628/2358	4883
A3	10	12198868	SEC61A2	NM_001142627.1	C	C/T	intron	none	NA	NA
A3	19	4556045	SEMA6B	NM_032108.3	A	A/G	coding-synonymous	none	142/889	426
A3	14	95081028	SERPINA3	NM_001085.4	T	A/T	missense	LEU,MET	84/424	250
A3	3	47162359	SETD2	NM_014159.6	C	A/C	missense	ARG,ILE	1256/2565	3767
A3	4	140468218	SETD7	NM_030648.2	C	A/C	intron	none	NA	NA
A3	9	135150809	SETX	NM_015046.5	T	A/T	splice-3	none	NA	NA
A3	11	64533661	SF1	NM_001178030.1	C	A/C	intron	none	NA	NA
A3	11	65829449	SF3B2	NM_006842.2	C	C/T	coding-synonymous	none	653/896	1957
A3	10	7409607	SFMBT2	NM_001018039.1	T	A/T	intron	none	NA	NA
A3	17	7535214	SHBG	NM_001040.3	G	A/G	missense	ALA,THR	245/403	733
A3	19	52129270	SIGLEC5	NM_003830.3	C	A/C	intron	none	NA	NA
A3	19	51767473	SIGLECL1	NM_173635.1	C	A/C	intron	none	NA	NA
A3	17	6599210	SLC13A5	NM_001143838.1	G	G/T	missense	ALA,ASP	297/523	890
A3	13	99378356	SLC15A1	NM_005073.3	A	A/G	intron	none	NA	NA
A3	20	61588932	SLC17A9	NM_022082.3	G	G/T	missense-near-splic	GLY,TRP	133/437	397
A3	10	119013063	SLC18A2	NM_003054.4	C	C/G	intron	none	NA	NA
A3	3	38350424	SLC22A14	NM_004803.3	G	G/T	intron	none	NA	NA

A3	6	110756948	SLC22A16	NM_033125.3	G	G/T	intron	none	NA	NA	
A3	6	160664743	SLC22A2	NM_003058.3	G	G/T	missense	PHE,LEU	380/556		1140
A3	3	170723722	SLC2A2	NM_000340.1	A	A/G	intron	none	NA	NA	
A3	2	27480941	SLC30A3	NM_003459.4	G	C/G	intron	none	NA	NA	
A3	8	118173921	SLC30A8	NM_001172811.1	G	G/T	intron	none	NA	NA	
A3	1	1599997	SLC35E2B	NM_001110781.1	C	A/C	intron	none	NA	NA	
A3	17	33520392	SLC35G3	NM_152462.2	G	C/G	missense	ALA,GLY	312/339		935
A3	8	11188806	SLC35G5	NM_054028.1	G	C/G	missense	GLY,ALA	64/339		191
A3	5	150859026	SLC36A1	NM_078483.2	C	C/T	missense	ARG,CYS	379/477		1135
A3	14	21467587	SLC39A2	NM_001256588.1	C	C/T	utr-5	none	NA	NA	
A3	1	75677050	SLC44A5	NM_001130058.1	C	C/G	intron	none	NA	NA	
A3	17	19616004	SLC47A2	NM_001099646.1	A	A/G	intron	none	NA	NA	
A3	4	72313127	SLC4A4	NM_001098484.2	T	C/T	intron	none	NA	NA	
A3	22	32480776	SLC5A1	NM_000343.3	C	C/T	intron	none	NA	NA	
A3	5	492096	SLC9A3	NM_004174.2	G	A/G	missense	ALA,VAL	101/835		302
A3	12	21471776	SLCO1A2	NM_021094.3	C	C/T	missense	GLU,LYS	48/671		142
A3	5	101794281	SLCO6A1	NM_173488.3	T	A/T	intron	none	NA	NA	
A3	4	20569076	SLIT2	NM_004787.1	C	C/G	intron	none	NA	NA	
A3	19	44254513	SMG9	NM_019108.2	T	A/T	intron	none	NA	NA	
A3	8	51363365	SNTG1	NM_018967.2	A	A/T	intron	none	NA	NA	
A3	11	130749637	SNX19	NM_014758.2	T	C/T	intron	none	NA	NA	
A3	11	16119155	SOX6	NM_001145811.1	C	C/T	coding-synonymous-	none	326/802		978
A3	11	16255994	SOX6	NM_001145811.1	A	A/T	intron	none	NA	NA	
A3	5	151043705	SPARC	NM_003118.3	C	A/C	missense	ASP,TYR	276/304		826
A3	2	228883009	SPHKAP	NM_001142644.1	C	A/C	missense	ARG,ILE	854/1701		2561
A3	1	158585008	SPTA1	NM_003126.2	G	C/G	coding-synonymous	none	2262/2420		6786
A3	20	42088515	SRSF6	NM_006275.5	T	C/T	missense	CYS,ARG	121/345		361
A3	1	54692860	SSBP3	NM_001009955.2	G	C/G	intron	none	NA	NA	
A3	9	140084436	SSNA1	NM_003731.2	T	C/T	utr-3	none	NA	NA	
A3	2	191898336	STAT4	NM_001243835.1	C	C/T	missense	VAL,ILE	578/749		1732
A3	4	27009569	STIM2	NM_001169117.1	A	A/G	intron	none	NA	NA	
A3	8	99719502	STK3	NM_001256312.1	G	A/G	missense	THR,ILE	158/520		473
A3	7	23776744	STK31	NM_001260504.1	T	G/T	intron	none	NA	NA	
A3	9	130416015	STXBP1	NM_001032221.3	A	A/T	missense	SER,CYS	37/595		109
A3	14	53235676	STYX	NM_001130701.1	A	A/C	intron	none	NA	NA	
A3	1	172579248	SUCO	NM_014283.3	G	G/T	missense	ARG,ILE	1205/1255		3614
A3	12	118852326	SUDS3	NM_022491.2	A	A/C	utr-3	none	NA	NA	
A3	9	113275496	SVEP1	NM_153366.3	C	A/C	intron	none	NA	NA	
A3	6	152647655	SYNE1	NM_033071.3	T	G/T	coding-synonymous	none	4952/8750		14856
A3	6	152690568	SYNE1	NM_033071.3	C	C/T	intron	none	NA	NA	
A3	17	35900600	SYNRG	NM_001163544.1	C	A/C	missense	ARG,LEU	1005/1237		3014
A3	1	16721482	SZRD1	NM_001114600.1	G	G/T	intron	none	NA	NA	
A3	11	764704	TALDO1	NM_006755.1	A	A/G	intron	none	NA	NA	

A3	2	95539759	TEKT4	NM_144705.2	A	A/G	missense	MET,VAL	207/436		619
A3	17	56643044	TEX14	NM_001201457.1	C	A/C	intron	none	NA	NA	
A3	2	121995398	TFCP2L1	NM_014553.2	C	C/T	missense	ALA,THR	297/480		889
A3	3	30691868	TGFBR2	NM_001024847.2	A	A/T	stop-gained	LYS,stop	149/593		445
A3	12	56815651	TIMELESS	NM_003920.3	C	C/T	intron	none	NA	NA	
A3	9	75403468	TMC1	NM_138691.2	G	A/G	intron	none	NA	NA	
A3	1	205241097	TMCC2	NM_001242925.1	G	G/T	missense	VAL,LEU	581/632		1741
A3	7	98452844	TMEM130	NM_001134450.1	C	C/T	intron	none	NA	NA	
A3	5	87493579	TMEM161B	NM_153354.3	C	A/C	missense	ALA,SER	365/488		1093
A3	9	74327290	TMEM2	NM_001135820.1	G	C/G	intron	none	NA	NA	
A3	5	109904142	TMEM232	NM_001039763.3	C	A/C	intron	none	NA	NA	
A3	1	45120613	TMEM53	NM_024587.2	A	A/G	missense	LEU,PRO	151/278		452
A3	3	170824504	TNIK	NM_001161560.1	T	A/T	intron	none	NA	NA	
A3	4	122085237	TNIP3	NM_001128843.2	T	C/T	missense	GLU,GLY	85/370		254
A3	1	179815860	TOR1AIP2	NM_001199260.1	C	A/C	missense	GLU,ASP	253/471		759
A3	12	113722746	TPCN1	NM_001143819.1	G	G/T	intron	none	NA	NA	
A3	1	186289335	TPR	NM_003292.2	C	A/C	intron	none	NA	NA	
A3	1	186332441	TPR	NM_003292.2	T	C/T	intron	none	NA	NA	
A3	21	10951509	TPTE	NM_199259.2	C	A/C	intron	none	NA	NA	
A3	20	30345110	TPX2	NM_012112.4	A	A/G	intron	none	NA	NA	
A3	11	4411255	TRIM21	NM_003141.3	C	A/C	stop-gained	GLU,stop	129/476		385
A3	3	32933087	TRIM71	NM_001039111.1	A	A/G	coding-synonymous	none	797/869		2391
A3	4	8456559	TRMT44	NM_152544.2	A	A/G	intron	none	NA	NA	
A3	11	101325900	TRPC6	NM_004621.5	A	A/T	intron	none	NA	NA	
A3	19	49693419	TRPM4	NM_001195227.1	G	A/G	intron	none	NA	NA	
A3	2	234875455	TRPM8	NM_024080.4	A	A/T	intron	none	NA	NA	
A3	7	98576057	TRRAP	NM_001244580.1	A	A/G	intron	none	NA	NA	
A3	19	50251405	TSKS	NM_021733.1	G	A/G	coding-synonymous	none	172/593		516
A3	2	122513227	TSN	NM_001261401.1	G	C/G	utr-5	none	NA	NA	
A3	1	115604858	TSPAN2	NM_005725.4	G	A/G	intron	none	NA	NA	
A3	21	46012133	TSPEAR	NM_144991.2	T	G/T	intron	none	NA	NA	
A3	9	135281197	TTF1	NM_001205296.1	T	A/T	intron	none	NA	NA	
A3	17	46863379	TTLL6	NM_001130918.1	C	C/T	intron	none	NA	NA	
A3	2	179452075	TTN	NM_001256850.1	G	G/T	missense	ALA,ASP	19647/34351		58940
A3	2	179527933	TTN	NM_001256850.1	A	A/C	intron	none	NA	NA	
A3	2	179571478	TTN	NM_001256850.1	T	C/T	intron	none	NA	NA	
A3	2	179596522	TTN	NM_001256850.1	G	G/T	missense	LEU,MET	5377/34351		16129
A3	2	179613524	TTN	NM_001256850.1	G	G/T	intron	none	NA	NA	
A3	12	3031414	TULP3	NM_001160408.1	A	A/G	intron	none	NA	NA	
A3	6	7891996	TXNDC5	NM_001145549.2	G	A/G	intron	none	NA	NA	
A3	12	104742220	TXNRD1	NM_001093771.2	G	A/G	utr-3	none	NA	NA	
A3	15	41857165	TYRO3	NM_006293.3	C	A/C	intron	none	NA	NA	
A3	1	162560255	UAP1	NM_003115.4	G	G/T	missense	GLY,CYS	438/506		1312

A3	20	3102667	UBOX5	NM_001267584.1	T	A/T	coding-synonymous	none	206/513		618
A3	15	43330446	UBR1	NM_174916.2	C	A/C	intron	none	NA	NA	
A3	15	54803975	UNC13C	NM_001080534.1	C	A/C	missense	GLN,LYS	1802/2215		5404
A3	15	54916001	UNC13C	NM_001080534.1	A	A/C	missense	ILE,LEU	2070/2215		6208
A3	15	91491716	UNC45A	NM_001039675.1	G	G/T	intron	none	NA	NA	
A3	17	76799726	USP36	NM_025090.3	G	C/G	missense	ARG,GLY	851/1124		2551
A3	11	11954620	USP47	NM_017944.3	A	A/G	missense	GLU,GLY	506/1288		1517
A3	17	5045959	USP6	NM_004505.2	T	A/T	intron	none	NA	NA	
A3	1	108247157	VAV3	NM_006113.4	A	A/G	intron	none	NA	NA	
A3	9	79985858	VPS13A	NM_001018037.1	A	A/G	intron	none	NA	NA	
A3	8	100472932	VPS13B	NM_017890.4	C	C/T	intron	none	NA	NA	
A3	1	159827945	VSIG8	NM_001013661.1	G	G/T	missense	ALA,ASP	122/415		365
A3	19	990856	WDR18	NM_024100.3	G	G/T	missense	TRP,LEU	201/433		602
A3	1	241964578	WDR64	NM_144625.4	C	C/G	utr-3	none	NA	NA	
A3	9	95992246	WNK2	NM_006648.3	G	A/G	intron	none	NA	NA	
A3	1	168549200	XCL1	NM_002995.2	A	A/T	intron	none	NA	NA	
A3	2	168106798	XIRP2	NM_001079810.3	G	G/T	intron	none	NA	NA	
A3	3	38414342	XYLB	NM_005108.3	C	C/G	intron	none	NA	NA	
A3	1	33099426	ZBTB8OS	NM_178547.2	A	A/G	intron	none	NA	NA	
A3	14	75538122	ZC2HC1C	NM_001042430.1	C	C/T	intron	none	NA	NA	
A3	9	88923417	ZCCHC6	NM_001185059.1	A	A/G	coding-synonymous	none	1255/1496		3765
A3	16	67434803	ZDHHC1	NM_013304.2	T	A/T	intron	none	NA	NA	
A3	3	45000669	ZDHHC3	NM_001135179.1	G	A/G	missense	ALA,VAL	87/300		260
A3	10	114192080	ZDHHC6	NM_022494.1	G	C/G	intron	none	NA	NA	
A3	8	135490694	ZFAT	NM_001029939.3	C	A/C	utr-3	none	NA	NA	
A3	16	68592421	ZFP90	NM_133458.2	G	A/G	missense	GLY,GLU	69/637		206
A3	7	64439412	ZNF117	NM_015852.3	G	C/G	missense	HIS,GLN	179/484		537
A3	12	133779660	ZNF268	NM_001165881.2	A	A/G	missense	GLN,ARG	463/948		1388
A3	7	99674801	ZNF3	NM_017715.2	G	C/G	intron	none	NA	NA	
A3	2	95818698	ZNF514	NM_032788.1	T	C/T	intron	none	NA	NA	
A3	19	40511851	ZNF546	NM_178544.3	A	A/T	intron	none	NA	NA	
A3	19	56902987	ZNF582	NM_144690.1	G	G/T	intron	none	NA	NA	
A3	19	52496219	ZNF615	NM_001199324.1	C	C/T	missense	GLY,ARG	715/743		2143
A3	1	40960674	ZNF642	NM_198494.2	T	A/T	missense	MET,LYS	175/527		524
A3	19	56969492	ZNF667	NM_022103.3	T	A/T	intron	none	NA	NA	
A3	8	144776548	ZNF707	NM_001100598.1	G	G/T	missense	GLY,CYS	322/372		964
A3	4	289725	ZNF732	NM_001137608.1	C	A/C	intron	none	NA	NA	
A3	6	35257983	ZNF76	NM_003427.3	T	A/T	intron	none	NA	NA	
A3	9	99581597	ZNF782	NM_001001662.1	G	G/T	coding-synonymous	none	236/700		708
A3	19	12502379	ZNF799	NM_001080821.2	T	C/T	missense	LYS,ARG	278/644		833
A3	19	53856761	ZNF845	NM_138374.1	T	C/T	missense	CYS,ARG	945/971		2833
A3	20	47877057	ZNFX1	NM_021035.2	C	A/C	missense	GLY,CYS	787/1919		2359
A4	11	67432919	ALDH3B2	NM_000695.3	G	A/G	coding-synonymous	none	181/386		543

A4		5	74433833	ANKRD31	NM_001164443.1	G	A/G	intron	none	NA	NA	
A4		5	74433839	ANKRD31	NM_001164443.1	G	A/G	intron	none	NA	NA	
A4		4	36162273	ARAP2	NM_015230.3	C	A/C	intron	none	NA	NA	
A4		16	18806845	ARL6IP1	NM_015161.1	G	A/G	missense	ARG,CYS	117/204		349
A4		16	18806858	ARL6IP1	NM_015161.1	T	C/T	coding-synonymous	none	112/204		336
A4		8	131193153	ASAP1	NM_001247996.1	T	A/T	intron	none	NA	NA	
A4		8	131193166	ASAP1	NM_001247996.1	T	A/T	intron	none	NA	NA	
A4		7	99063777	ATP5J2	NM_001003713.2	G	C/G	utr-5	none	NA	NA	
A4		5	34922637	BRIX1	NM_018321.3	C	A/C	intron	none	NA	NA	
A4		11	61553664	C11orf9	NM_001127392.1	A	A/C	utr-3	none	NA	NA	
A4		9	140953706	CACNA1B	NM_000718.3	G	A/G	intron	none	NA	NA	
A4		12	88472950	CEP290	NM_025114.3	T	C/T	coding-synonymous	none	1761/2480		5283
A4		2	122208378	CLASP1	NM_001142273.1	C	C/T	intron	none	NA	NA	
A4		16	76501363	CNTNAP4	NM_033401.3	A	A/G	missense	GLN,ARG	533/1309		1597
A4	X		107683501	COL4A5	NM_000495.4	T	G/T	intron	none	NA	NA	
A4		22	42523309	CYP2D6	NM_000106.5	C	C/T	intron	none	NA	NA	
A4		7	50607598	DDC	NM_000790.3	C	C/T	intron	none	NA	NA	
A4		17	61889373	DDX42	NM_007372.2	G	G/T	stop-gained	GLU,stop	494/939		1480
A4		10	124359323	DMBT1	NM_004406.2	A	A/T	intron	none	NA	NA	
A4		5	34947679	DNAJC21	NM_001012339.2	A	A/T	intron	none	NA	NA	
A4		2	225761013	DOCK10	NM_014689.2	G	A/G	stop-gained-near-spl	ARG,stop	139/2187		415
A4		7	76133863	DTX2	NM_001102594.1	C	C/T	intron	none	NA	NA	
A4		8	120581493	ENPP2	NM_001040092.2	C	C/T	missense	GLY,ARG	679/864		2035
A4		12	132547126	EP400	NM_015409.4	G	A/G	coding-synonymous	none	2738/3124		8214
A4		15	43502388	EPB42	NM_000119.2	G	A/G	intron	none	NA	NA	
A4		14	100594935	EVL	NM_016337.2	A	A/C	coding-synonymous	none	189/419		567
A4	X		154114301	F8	NM_000132.3	G	C/G	intron	none	NA	NA	
A4	X		154114509	F8	NM_000132.3	T	C/T	intron	none	NA	NA	
A4	X		153736694	FAM3A	NM_001171132.1	T	G/T	intron	none	NA	NA	
A4		3	81635297	GBE1	NM_000158.3	T	C/T	coding-synonymous	none	427/703		1281
A4		14	39602849	GEMIN2	NM_001009182.1	C	C/T	intron	none	NA	NA	
A4		15	52415104	GNB5	NM_006578.3	A	A/C	intron	none	NA	NA	
A4		1	155733045	GON4L	NM_001037533.1	C	C/T	intron	none	NA	NA	
A4	X		122586205	GRIA3	NM_000828.4	T	C/T	intron	none	NA	NA	
A4		12	42481540	GXYLT1	NM_001099650.1	T	C/T	utr-3	none	NA	NA	
A4		9	21187406	IFNA4	NM_021068.2	G	C/G	missense	ALA,GLY	42/190		125
A4		14	88651826	KCNK10	NM_021161.4	A	A/C	utr-3	none	NA	NA	
A4		1	215345280	KCNK2	NM_001017424.2	T	C/T	intron	none	NA	NA	
A4		6	39304410	KIF6	NM_145027.4	A	A/C	intron	none	NA	NA	
A4		17	39261778	KRTAP4-9	NM_001146041.1	A	A/G	coding-synonymous	none	46/211		138
A4		11	1619403	KRTAP5-2	NM_001004325.1	A	A/G	coding-synonymous	none	26/178		78
A4		4	151753153	LRBA	NM_001199282.2	G	A/G	intron	none	NA	NA	
A4	X		149566600	MAMLD1	NM_001177465.1	A	A/G	intron	none	NA	NA	

A4	2	170678576	METTL5	NM_014168.2	T	A/T	intron	none	NA	NA
A4	11	118375409	MLL	NM_001197104.1	G	G/T	coding-synonymous	none	2934/3973	8802
A4	7	104753246	MLL5	NM_018682.3	A	A/C	coding-synonymous	none	1681/1859	5043
A4	3	195507717	MUC4	NM_004532.5	G	G/T	intron	none	NA	NA
A4	3	40085814	MYRIP	NM_015460.2	A	A/C	intron	none	NA	NA
A4	1	16908777	NBPF1	NM_017940.3	G	C/G	intron	none	NA	NA
A4	1	148251802	none	none	C	C/G	intergenic	none	NA	NA
A4	2	87601728	none	none	A	A/G	intergenic	none	NA	NA
A4	2	90121732	none	none	A	A/G	intergenic	none	NA	NA
A4	7	142131356	none	none	G	G/T	intergenic	none	NA	NA
A4	7	150446954	none	none	C	C/G	intergenic	none	NA	NA
A4	10	134760307	none	none	T	C/T	intergenic	none	NA	NA
A4	11	1619556	none	none	T	C/T	near-gene-5	none	NA	NA
A4	14	106361456	none	none	G	G/T	intergenic	none	NA	NA
A4	15	20663279	none	none	C	C/T	intergenic	none	NA	NA
A4	22	21829074	none	none	T	C/T	intergenic	none	NA	NA
A4	X	133680382	none	none	C	C/T	intergenic	none	NA	NA
A4	9	87475814	NTRK2	NM_001018064.1	A	A/G	intron	none	NA	NA
A4	1	27272600	NUDC	NM_006600.3	A	A/T	intron	none	NA	NA
A4	11	5565938	OR52H1	NM_001005289.1	A	A/G	coding-synonymous	none	272/321	816
A4	3	196509562	PAK2	NM_002577.4	T	C/T	coding-synonymous	none	15/525	45
A4	5	140755049	PCDHGB3	NM_018912.2	A	A/C	intron	none	NA	NA
A4	11	103870762	PDGFD	NM_025208.4	T	A/T	intron	none	NA	NA
A4	9	96375842	PHF2	NM_005392.3	C	C/T	intron	none	NA	NA
A4	21	44438181	PKNOX1	NM_004571.3	G	A/G	intron	none	NA	NA
A4	12	81735054	PPFIA2	NM_001220473.1	A	A/C	intron	none	NA	NA
A4	16	90127826	PRDM7	NM_001098173.1	T	A/T	intron	none	NA	NA
A4	14	62016387	PRKCH	NM_006255.3	C	C/T	intron	none	NA	NA
A4	2	231943505	PSMD1	NM_001191037.1	C	C/T	intron	none	NA	NA
A4	3	61975376	PTPRG	NM_002841.3	G	A/G	missense	ASP,ASN	90/1446	268
A4	12	81013916	PTPRQ	NM_001145026.1	T	C/T	intron	none	NA	NA
A4	1	156348012	RHBG	NM_001256395.1	C	C/G	intron	none	NA	NA
A4	7	6820672	RSPH10B2	NM_001099697.1	A	A/G	intron	none	NA	NA
A4	13	32360691	RXFP2	NM_001166058.1	T	A/T	intron	none	NA	NA
A4	19	39412767	SARS2	NM_001145901.1	C	C/T	intron	none	NA	NA
A4	22	50906148	SBF1	NM_002972.2	A	A/G	intron	none	NA	NA
A4	8	20038348	SLC18A1	NM_001135691.2	G	C/G	intron	none	NA	NA
A4	15	67473822	SMAD3	NM_001145102.1	A	A/C	intron	none	NA	NA
A4	2	17927256	SMC6	NM_001142286.1	G	G/T	intron	none	NA	NA
A4	17	1704353	SMYD4	NM_052928.2	G	C/G	intron	none	NA	NA
A4	1	54871665	SSBP3	NM_001009955.2	T	C/T	missense	LYS,ARG	6/362	17
A4	2	202344188	STRADB	NM_001206864.1	T	A/T	intron	none	NA	NA
A4	1	115428100	SYCP1	NM_003176.2	T	C/T	intron	none	NA	NA

A4	1	43903588	SZT2	NM_015284.3	G	A/G	intron	none	NA	NA	
A4	22	31010295	TCN2	NM_000355.3	T	G/T	intron	none	NA	NA	
A4	21	45994578	TSPEAR	NM_144991.2	C	A/C	intron	none	NA	NA	
A4	9	100367219	TSTD2	NM_139246.4	C	C/T	intron	none	NA	NA	
A4	2	179416642	TTN	NM_001256850.1	C	C/T	missense	GLY,ARG	28688/34351		86062
A4	7	66520742	TYW1	NM_018264.2	T	A/T	intron	none	NA	NA	
A4	5	36048791	UGT3A2	NM_001168316.1	C	C/G	intron	none	NA	NA	
A4	1	16271569	ZBTB17	NM_001242884.1	C	C/T	missense	GLU,LYS	182/722		544
A4	7	30329158	ZNRF2	NM_147128.3	G	A/G	intron	none	NA	NA	
A5	11	105962117	AASDHPPT	NM_015423.2	A	A/G	coding-synonymous	none	202/310		606
A5	7	152497540	ACTR3B	NM_001040135.2	A	A/T	intron	none	NA	NA	
A5	4	175897504	ADAM29	NM_001130703.1	C	C/T	coding-synonymous	none	276/821		828
A5	8	131796088	ADCY8	NM_001115.2	G	A/G	intron	none	NA	NA	
A5	1	109380312	AKNAD1	NM_152763.4	T	C/T	missense	ILE,VAL	466/837		1396
A5	12	47472266	AMIGO2	NM_001143668.1	C	C/T	missense	GLY,ARG	174/523		520
A5	10	37482263	ANKRD30A	NM_052997.2	C	C/T	intron	none	NA	NA	
A5	2	97909530	ANKRD36	NM_001164315.1	T	A/T	intron	none	NA	NA	
A5	5	175793371	ARL10	NM_173664.4	G	A/G	intron	none	NA	NA	
A5	19	3913874	ATCAY	NM_033064.4	A	A/C	intron	none	NA	NA	
A5	14	96797797	ATG2B	NM_018036.5	T	C/T	missense	LYS,ARG	549/2079		1646
A5	1	63270823	ATG4C	NM_032852.3	C	C/T	intron	none	NA	NA	
A5	3	142231081	ATR	NM_001184.3	G	A/G	intron	none	NA	NA	
A5	9	96714994	BARX1	NM_021570.3	C	C/T	intron	none	NA	NA	
A5	11	57513407	BTBD18	NM_001145101.1	C	C/T	missense	ARG,HIS	113/713		338
A5	17	41859283	C17orf105	NM_001136483.1	C	A/C	intron	none	NA	NA	
A5	5	37148383	C5orf42	NM_023073.3	C	C/T	intron	none	NA	NA	
A5	16	67100781	CBFB	NM_001755.2	A	A/G	intron	none	NA	NA	
A5	9	69247625	CBWD6	NM_001085457.1	T	C/T	intron	none	NA	NA	
A5	19	14040139	CC2D1A	NM_017721.4	A	A/T	intron	none	NA	NA	
A5	3	156877277	CCNL1	NM_020307.2	A	A/G	intron	none	NA	NA	
A5	7	5965381	CCZ1	NM_015622.5	G	A/G	utr-3	none	NA	NA	
A5	3	121810436	CD86	NM_001206924.1	T	C/T	intron	none	NA	NA	
A5	5	176004739	CDHR2	NM_001171976.1	G	A/G	coding-synonymous	none	484/1311		1452
A5	17	15500034	CDRT1	NM_006382.3	A	A/G	intron	none	NA	NA	
A5	15	49085683	CEP152	NM_001194998.1	G	C/G	intron	none	NA	NA	
A5	15	93557925	CHD2	NM_001271.3	G	A/G	splice-3	none	NA	NA	
A5	3	439979	CHL1	NM_001253387.1	C	C/T	missense	PRO,LEU	1039/1209		3116
A5	7	136700108	CHRM2	NM_000739.2	G	C/G	missense	VAL,LEU	166/467		496
A5	8	87738574	CNGB3	NM_019098.4	C	C/G	intron	none	NA	NA	
A5	8	121215879	COL14A1	NM_021110.1	G	A/G	intron	none	NA	NA	
A5	16	12897610	CPPED1	NM_001099455.1	A	A/C	coding-synonymous	none	8/173		24
A5	8	114186041	CSMD3	NM_052900.2	C	C/G	missense	GLY,ARG	207/3539		619
A5	16	67660510	CTCF	NM_001191022.1	T	G/T	coding-synonymous	none	142/400		426

A5	3	37988703	CTDSPL	NM_001008392.1	G	G/T	splice-5	none	NA	NA	
A5	13	36362292	DCLK1	NM_001195415.1	G	C/G	intron	none	NA	NA	
A5	22	19127200	DGCR14	NM_022719.2	C	C/T	missense	ALA,THR	205/477		613
A5	10	124359365	DMBT1	NM_004406.2	G	G/T	intron	none	NA	NA	
A5	10	128904542	DOCK1	NM_001380.3	G	A/G	coding-synonymous	none	795/1851		2385
A5	6	83848516	DOPEY1	NM_001199942.1	G	C/G	coding-synonymous	none	1576/2477		4728
A5	11	117375686	DSCAML1	NM_020693.2	G	A/G	missense	SER,LEU	772/2114		2315
A5	15	45386693	DUOX2	NM_014080.4	T	G/T	intron	none	NA	NA	
A5	1	64020901	EFCAB7	NM_032437.2	G	G/T	intron	none	NA	NA	
A5	3	138351809	FAIM	NM_001033030.1	G	G/T	missense	SER,ILE	177/214		530
A5	2	58392879	FANCL	NM_001114636.1	G	G/T	missense	THR,LYS	229/381		686
A5	1	149763191	FCGR1A	NM_000566.3	G	A/G	utr-3	none	NA	NA	
A5	8	11666258	FDFT1	NM_004462.3	T	C/T	intron	none	NA	NA	
A5	12	4481751	FGF23	NM_020638.2	G	G/T	intron	none	NA	NA	
A5	4	190878447	FRG1	NM_004477.2	T	C/T	intron	none	NA	NA	
A5	15	83451729	FSD2	NM_001007122.2	T	C/T	missense	ILE,VAL	262/750		784
A5	9	101216357	GABBR2	NM_005458.7	C	C/T	missense	ARG,GLN	381/942		1142
A5	10	26505839	GAD2	NM_000818.2	G	A/G	intron	none	NA	NA	
A5	15	72954696	GOLGA6B	NM_018652.4	G	A/G	coding-synonymous	none	317/694		951
A5	1	156565670	GPATCH4	NM_015590.3	G	A/G	intron	none	NA	NA	
A5	13	94958138	GPC6	NM_005708.3	A	A/C	intron	none	NA	NA	
A5	5	90074275	GPR98	NM_032119.3	G	C/G	missense	SER,THR	4233/6307		12698
A5	7	65444573	GUSB	NM_000181.3	C	A/C	intron	none	NA	NA	
A5	6	168377051	HGC6.3	NM_001129895.2	G	C/G	coding-synonymous	none	94/172		282
A5	1	79128462	IFI44	NM_006417.4	G	G/T	missense	TRP,LEU	396/445		1187
A5	3	19479610	KCNH8	NM_144633.2	G	C/G	intron	none	NA	NA	
A5	3	197423577	KIAA0226	NM_001145642.2	C	C/T	intron	none	NA	NA	
A5	16	27760836	KIAA0556	NM_015202.2	T	C/T	intron	none	NA	NA	
A5	17	72350886	KIF19	NM_153209.3	G	A/G	intron	none	NA	NA	
A5	3	183209892	KLHL6	NM_130446.2	G	A/G	coding-synonymous	none	563/622		1689
A5	12	25380276	KRAS	NM_004985.3	T	A/T	missense	GLN,LEU	61/189		182
A5	12	52865351	KRT6C	NM_173086.4	T	C/T	intron	none	NA	NA	
A5	22	33828139	LARGE	NM_004737.4	G	G/T	intron	none	NA	NA	
A5	10	90503151	LIPK	NM_001080518.1	A	A/C	intron	none	NA	NA	
A5	6	57512367	LOC10099648	XM_003846228.1	T	C/T	intron	none	NA	NA	
A5	13	47127513	LRCH1	NM_001164211.1	T	G/T	utr-5	none	NA	NA	
A5	2	141201836	LRP1B	NM_018557.2	A	A/C	intron	none	NA	NA	
A5	8	56910929	LYN	NM_001111097.2	G	A/G	missense	GLU,LYS	338/492		1012
A5	15	66737078	MAP2K1	NM_002755.3	A	A/C	intron	none	NA	NA	
A5	19	876967	MED16	NM_005481.2	C	C/G	intron	none	NA	NA	
A5	15	100252748	MEF2A	NM_001130926.1	G	A/G	coding-synonymous	none	422/498		1266
A5	3	179080301	MFN1	NM_033540.2	G	A/G	intron	none	NA	NA	
A5	22	31346333	MORC2	NM_014941.1	A	A/C	intron	none	NA	NA	

A5	17	18071036	MYO15A	NM_016239.3	G	A/G	missense-near-splice	VAL,MET	3361/3531		10081
A5	19	17291867	MYO9B	NM_001130065.1	G	A/G	intron	none	NA	NA	
A5	13	101717739	NALCN	NM_052867.2	G	A/G	intron	none	NA	NA	
A5	13	35630098	NBEA	NM_015678.4	T	C/T	intron	none	NA	NA	
A5	13	35630103	NBEA	NM_015678.4	C	C/T	intron	none	NA	NA	
A5	1	156003662	none	none	C	A/C	near-gene-3	none	NA	NA	
A5	2	38963040	none	none	G	A/G	near-gene-3	none	NA	NA	
A5	2	90260116	none	none	A	A/C	intergenic	none	NA	NA	
A5	7	142008327	none	none	C	A/C	intergenic	none	NA	NA	
A5	7	142168371	none	none	C	C/T	intergenic	none	NA	NA	
A5	8	133117549	none	none	C	A/C	near-gene-5	none	NA	NA	
A5	14	106478032	none	none	A	A/T	intergenic	none	NA	NA	
A5	14	106552839	none	none	A	A/G	intergenic	none	NA	NA	
A5	14	107083220	none	none	T	G/T	intergenic	none	NA	NA	
A5	20	29589921	none	none	G	C/G	intergenic	none	NA	NA	
A5	21	31986316	none	none	A	A/C	near-gene-5	none	NA	NA	
A5	11	108031963	NPAT	NM_002519.2	T	C/T	missense	ILE,VAL	1284/1428		3850
A5	9	35806233	NPR2	NM_003995.3	G	A/G	intron	none	NA	NA	
A5	3	52562523	NT5DC2	NM_001134231.1	A	A/G	missense	ILE,THR	194/558		581
A5	5	37325894	NUP155	NM_004298.2	T	A/T	intron	none	NA	NA	
A5	X	153461704	OPN1MW	NM_000513.2	C	G/G	utr-3	none	NA	NA	
A5	1	248224451	OR2L13	NM_001004687.1	T	C/T	coding-synonymous	none	156/313		468
A5	9	37002590	PAX5	NM_016734.1	C	C/T	intron	none	NA	NA	
A5	10	95791949	PLCE1	NM_016341.3	G	A/G	coding-synonymous	none	382/2303		1146
A5	12	133218469	POLE	NM_006231.2	T	A/T	intron	none	NA	NA	
A5	19	1091773	POLR2E	NM_002695.3	T	G/T	intron	none	NA	NA	
A5	12	121017475	POP5	NM_015918.3	T	G/T	intron	none	NA	NA	
A5	18	14513570	POTEC	NM_001137671.1	G	A/G	intron	none	NA	NA	
A5	1	13448205	PRAMEF13	NM_001024661.1	G	C/G	missense	ARG,GLY	424/475		1270
A5	7	151552243	PRKAG2	NM_016203.3	T	C/T	intron	none	NA	NA	
A5	16	23847759	PRKCB	NM_002738.6	G	A/G	intron	none	NA	NA	
A5	9	33796495	PRSS3	NM_001197097.2	C	A/C	intron	none	NA	NA	
A5	9	33796519	PRSS3	NM_001197097.2	T	C/T	intron	none	NA	NA	
A5	9	33796892	PRSS3	NM_001197097.2	C	C/T	intron	none	NA	NA	
A5	5	139215197	PSD2	NM_032289.2	G	G/T	intron	none	NA	NA	
A5	19	43258759	PSG8	NM_001130167.1	G	A/G	intron	none	NA	NA	
A5	6	72678679	RIMS1	NM_014989.5	G	A/G	intron	none	NA	NA	
A5	14	21269749	RNASE1	NM_002933.4	C	A/C	utr-3	none	NA	NA	
A5	4	76892660	SDAD1	NM_018115.2	A	A/G	intron	none	NA	NA	
A5	7	4308373	SDK1	NM_001079653.1	G	A/G	coding-synonymous	none	677/680		2031
A5	14	94935660	SERPINA9	NM_001042518.1	G	A/G	missense-near-splice	ALA,VAL	91/336		272
A5	21	37431299	SETD4	NM_001007259.1	A	A/G	intron	none	NA	NA	
A5	17	2279597	SGSM2	NM_001098509.1	G	C/G	intron	none	NA	NA	

A5	7	98643515	SMURF1	NM_001199847.1	C	C/T	intron	none	NA	NA	
A5	2	231176175	SP140	NM_007237.4	G	C/G	missense	GLU,ASP	790/868		2370
A5	7	99795240	STAG3	NM_012447.2	A	A/T	intron	none	NA	NA	
A5	8	70498648	SULF1	NM_001128204.1	C	C/T	stop-gained	ARG,stop	157/872		469
A5	17	15638785	TBC1D26	NM_178571.4	C	C/T	intron	none	NA	NA	
A5	14	96152931	TCL1B	NM_004918.3	C	C/T	missense	ARG,CYS	43/129		127
A5	15	90151987	TICRR	NM_152259.3	G	A/G	intron	none	NA	NA	
A5	16	1279771	TPSB2	NM_024164.5	A	A/C	intron	none	NA	NA	
A5	16	1307050	TPSD1	NM_012217.2	C	C/T	coding-synonymous	none	169/243		507
A5	1	211533153	TRAF5	NM_001033910.2	G	C/G	intron	none	NA	NA	
A5	16	2220691	TRAF7	NM_032271.2	T	C/T	missense	LEU,PRO	103/671		308
A5	4	184633732	TRAPPC11	NM_021942.5	T	G/T	intron	none	NA	NA	
A5	13	99966302	UBAC2	NM_001144072.1	G	C/G	intron	none	NA	NA	
A5	9	33973212	UBAP2	NM_018449.2	C	C/T	missense	GLY,ARG	182/1120		544
A5	3	184742114	VPS8	NM_001009921.2	T	G/T	intron	none	NA	NA	
A5	7	70800582	WBSCR17	NM_022479.1	T	G/T	coding-synonymous	none	95/599		285
A5	19	989769	WDR18	NM_024100.3	C	C/T	missense	THR,ILE	110/433		329
A5	2	20130984	WDR35	NM_001006657.1	C	C/T	intron	none	NA	NA	
A5	20	21314236	XRN2	NM_012255.3	C	C/T	missense	ARG,TRP	305/951		913
A5	14	68273123	ZFYVE26	NM_015346.3	T	A/T	intron	none	NA	NA	
A6	17	67246146	ABCA5	NM_018672.3	T	C/T	intron	none	NA	NA	
A6	17	66877130	ABCA8	NM_007168.2	T	C/T	intron	none	NA	NA	
A6	17	66913410	ABCA8	NM_007168.2	A	A/G	intron	none	NA	NA	
A6	3	111700646	ABHD10	NM_018394.2	C	C/T	missense	THR,MET	53/307		158
A6	14	23549902	ACIN1	NM_001164814.1	T	C/T	coding-synonymous	none	272/1329		816
A6	2	135619696	ACMSD	NM_138326.2	C	C/G	intron	none	NA	NA	
A6	5	80643682	ACOT12	NM_130767.2	C	A/C	coding-synonymous	none	188/556		564
A6	19	51295487	ACPT	NM_033068.2	C	A/C	intron	none	NA	NA	
A6	20	33508924	ACSS2	NM_001076552.2	G	G/T	missense	GLY,VAL	433/715		1298
A6	15	35082821	ACTC1	NM_005159.4	A	A/T	intron	none	NA	NA	
A6	1	236900503	ACTN2	NM_001103.2	C	A/C	missense	LEU,ILE	289/895		865
A6	7	152520233	ACTR3B	NM_001040135.2	G	A/G	intron	none	NA	NA	
A6	5	33535102	ADAMTS12	NM_030955.2	A	A/C	intron	none	NA	NA	
A6	5	33549507	ADAMTS12	NM_030955.2	C	C/T	intron	none	NA	NA	
A6	5	33577292	ADAMTS12	NM_030955.2	T	C/T	intron	none	NA	NA	
A6	5	33614248	ADAMTS12	NM_030955.2	T	C/T	intron	none	NA	NA	
A6	1	167798568	ADCY10	NM_001167749.1	A	A/C	stop-gained	TYR,stop	1076/1458		3228
A6	5	7626564	ADCY2	NM_020546.2	T	A/T	intron	none	NA	NA	
A6	8	132052146	ADCY8	NM_001115.2	C	C/T	missense	GLY,GLU	145/1252		434
A6	2	70903996	ADD2	NM_001185054.1	C	C/T	missense	ASP,ASN	509/727		1525
A6	4	74315178	AFP	NM_001134.1	T	C/T	coding-synonymous	none	395/610		1185
A6	10	51754236	AGAP6	NM_001077665.2	A	A/T	intron	none	NA	NA	
A6	21	45378993	AGPAT3	NM_001037553.1	A	A/G	intron	none	NA	NA	

A6	4	109667765	AGXT2L1	NM_001146590.1	T	A/T	intron	none	NA	NA	
A6	1	247031203	AHCTF1	NM_015446.4	C	A/C	intron	none	NA	NA	
A6	17	7948471	ALOX15B	NM_001039130.1	C	C/T	intron	none	NA	NA	
A6	1	104203448	AMY1A	NM_001008221.1	G	G/T	intron	none	NA	NA	
A6	2	112608522	ANAPC1	NM_022662.3	T	C/T	intron	none	NA	NA	
A6	17	54451913	ANKFN1	NM_153228.2	G	G/T	intron	none	NA	NA	
A6	17	54554821	ANKFN1	NM_153228.2	C	C/T	intron	none	NA	NA	
A6	12	99898198	ANKS1B	NM_152788.4	T	A/T	intron	none	NA	NA	
A6	11	26655794	ANO3	NM_031418.2	G	A/G	coding-synonymous	none	639/982		1917
A6	8	124708060	ANXA13	NM_001003954.1	G	A/G	intron	none	NA	NA	
A6	9	33401407	AQP7	NM_001170.1	A	A/G	intron	none	NA	NA	
A6	15	35168093	AQR	NM_014691.2	C	C/G	intron	none	NA	NA	
A6	4	36075434	ARAP2	NM_015230.3	A	A/G	coding-synonymous	none	1540/1705		4620
A6	8	68211696	ARFGEF1	NM_006421.4	T	A/T	intron	none	NA	NA	
A6	20	47587788	ARFGEF2	NM_006420.2	C	C/T	missense	SER,PHE	441/1786		1322
A6	2	144007922	ARHGAP15	NM_018460.3	C	A/C	intron	none	NA	NA	
A6	7	144068206	ARHGEF5	NM_005435.3	G	G/T	intron	none	NA	NA	
A6	11	3660128	ART5	NM_001079536.1	G	A/G	intron	none	NA	NA	
A6	4	177190130	ASB5	NM_080874.3	C	C/T	missense	VAL,MET	44/330		130
A6	1	176851908	ASTN1	NM_004319.1	A	A/G	intron	none	NA	NA	
A6	9	119216670	ASTN2	NM_001184734.1	G	A/G	intron	none	NA	NA	
A6	9	119770362	ASTN2	NM_014010.4	A	A/G	intron	none	NA	NA	
A6	15	25959071	ATP10A	NM_024490.3	C	A/C	coding-synonymous	none	698/1500		2094
A6	15	25972216	ATP10A	NM_024490.3	G	G/T	intron	none	NA	NA	
A6	17	7558116	ATP1B2	NM_001678.3	C	C/T	intron	none	NA	NA	
A6	12	90020410	ATP2B1	NM_001001323.1	T	C/T	intron	none	NA	NA	
A6	14	92572940	ATXN3	NM_001127696.1	C	C/G	utr-5	none	NA	NA	
A6	X	69264287	AWAT2	NM_001002254.1	G	A/A	coding-synonymous	none	37/334		111
A6	1	179504019	AXDND1	NM_144696.4	G	G/T	stop-gained	GLU,stop	985/1013		2953
A6	17	79073786	BAIAP2	NM_001144888.1	A	A/G	missense	TYR,CYS	183/535		548
A6	1	212860390	BATF3	NM_018664.2	G	G/T	intron	none	NA	NA	
A6	2	160256982	BAZ2B	NM_013450.2	C	A/C	intron	none	NA	NA	
A6	2	60780417	BCL11A	NM_018014.3	G	C/G	utr-5	none	NA	NA	
A6	15	80253533	BCL2A1	NM_001114735.1	T	G/T	intron	none	NA	NA	
A6	16	14758600	BFAR	NM_016561.2	G	G/T	intron	none	NA	NA	
A6	11	102198976	BIRC3	NM_001165.4	T	G/T	intron	none	NA	NA	
A6	2	32664484	BIRC6	NM_016252.3	G	A/G	intron	none	NA	NA	
A6	X	79945432	BRWD3	NM_153252.4	G	T/T	intron	none	NA	NA	
A6	16	49432961	C16orf78	NM_144602.2	C	C/G	intron	none	NA	NA	
A6	16	5097978	C16orf89	NM_001098514.2	C	C/T	intron	none	NA	NA	
A6	17	80363100	C17orf101	NM_024648.2	G	G/T	intron	none	NA	NA	
A6	1	170916796	C1orf129	NM_001163629.1	G	C/G	intron	none	NA	NA	
A6	22	32804254	C22orf28	NM_014306.4	A	A/G	intron	none	NA	NA	

A6	5	41181474	C6	NM_000065.2	G	G/T	missense	ALA,ASP	305/935		914
A6	6	121434187	C6orf170	NM_152730.4	G	G/T	intron	none	NA	NA	
A6	8	61137016	CA8	NM_004056.4	T	C/T	intron	none	NA	NA	
A6	7	81624378	CACNA2D1	NM_000722.2	G	A/G	intron	none	NA	NA	
A6	11	14990319	CALCA	NM_001033952.2	G	G/T	utr-3	none	NA	NA	
A6	6	17539369	CAP2	NM_006366.2	G	A/G	intron	none	NA	NA	
A6	7	142989595	CASP2	NM_001224.4	G	C/G	intron	none	NA	NA	
A6	7	107399152	CBLL1	NM_024814.2	G	G/T	missense	MET,ILE	335/492		1005
A6	16	57760669	CCDC135	NM_032269.5	T	G/T	intron	none	NA	NA	
A6	11	124861574	CCDC15	NM_025004.2	A	A/T	intron	none	NA	NA	
A6	4	77303868	CCDC158	NM_001042784.1	T	A/T	missense	GLU,VAL	270/1114		809
A6	6	151859426	CCDC170	NM_025059.3	G	G/T	stop-gained	GLU,stop	145/716		433
A6	10	118116912	CCDC172	NM_198515.2	G	C/G	missense	ASP,HIS	157/259		469
A6	3	191047401	CCDC50	NM_174908.3	C	C/G	utr-5	none	NA	NA	
A6	12	518391	CCDC77	NM_001130146.1	G	A/G	intron	none	NA	NA	
A6	19	47774479	CCDC9	NM_015603.2	G	G/T	intron	none	NA	NA	
A6	10	35842154	CCNY	NM_145012.4	G	G/T	intron	none	NA	NA	
A6	1	145706743	CD160	NM_007053.2	C	C/T	missense	GLY,SER	6/182		16
A6	12	7654174	CD163	NM_004244.5	T	A/T	intron	none	NA	NA	
A6	19	43865544	CD177	NM_020406.2	C	C/G	intron	none	NA	NA	
A6	3	112643118	CD200R1	NM_138806.3	G	C/G	intron	none	NA	NA	
A6	19	51738670	CD33	NM_001082618.1	G	A/G	intron	none	NA	NA	
A6	17	45234350	CDC27	NM_001114091.1	C	C/T	coding-synonymous	none	257/831		771
A6	1	1575687	CDK11B	NM_033486.1	G	A/G	missense	SER,LEU	316/698		947
A6	13	26911733	CDK8	NM_001260.1	A	A/C	missense	GLN,PRO	53/465		158
A6	9	21974675	CDKN2A	NM_000077.4	A	A/T	splice-5	none	NA	NA	
A6	4	85556408	CDS1	NM_001263.3	T	A/T	intron	none	NA	NA	
A6	22	17669280	CECR1	NM_017424.2	G	G/T	missense	PRO,THR	344/512		1030
A6	22	17982091	CECR2	NM_031413.3	C	C/T	intron	none	NA	NA	
A6	1	196926964	CFHR2	NM_005666.2	T	A/T	intron	none	NA	NA	
A6	7	132470224	CHCHD3	NM_017812.2	A	A/G	utr-3	none	NA	NA	
A6	8	61707437	CHD7	NM_017780.3	G	A/G	intron	none	NA	NA	
A6	21	19629249	CHODL	NM_001204174.1	G	A/G	intron	none	NA	NA	
A6	15	30675718	CHRFAM7A	NM_139320.1	A	A/T	intron	none	NA	NA	
A6	15	101718311	CHSY1	NM_014918.4	T	C/T	missense	ASN,SER	564/803		1691
A6	16	70211149	CLEC18C	NM_173619.2	G	G/T	missense	TRP,CYS	74/447		222
A6	16	70211409	CLEC18C	NM_173619.2	C	C/T	intron	none	NA	NA	
A6	1	155236782	CLK2	NM_003993.2	C	C/T	intron	none	NA	NA	
A6	12	92821806	CLLU1OS	NM_001025232.1	C	C/T	intron	none	NA	NA	
A6	3	150690404	CLRN1	NM_001195794.1	G	G/T	missense	PRO,GLN	31/246		92
A6	2	98994252	CNGA3	NM_001079878.1	G	G/T	missense	GLN,HIS	68/677		204
A6	8	87666109	CNGB3	NM_019098.4	A	A/T	intron	none	NA	NA	
A6	1	86252107	COL24A1	NM_152890.5	C	C/G	missense	GLY,ALA	1330/1715		3989

A6		2	189901676	COL5A2	NM_000393.3	C	C/T	intron	none	NA	NA	
A6		2	238274562	COL6A3	NM_004369.3	G	A/G	missense	HIS,TYR	1873/3178		5617
A6		6	70961768	COL9A1	NM_001851.4	C	A/C	intron	none	NA	NA	
A6		6	71009832	COL9A1	NM_001851.4	T	C/T	coding-synonymous	none	71/922		213
A6		19	17010358	CPAMD8	NM_015692.2	C	A/C	coding-synonymous	none	1639/1933		4917
A6		17	28750721	CPD	NM_001199775.1	G	A/G	intron	none	NA	NA	
A6		17	28789554	CPD	NM_001199775.1	G	A/G	intron	none	NA	NA	
A6		14	92622959	CPSF2	NM_017437.2	G	A/G	missense	GLU,LYS	527/783		1579
A6		10	125601773	CPXM2	NM_198148.2	C	A/C	intron	none	NA	NA	
A6		11	122742057	CRTAM	NM_019604.2	C	A/C	missense	HIS,ASN	378/394		1132
A6		19	48342922	CRX	NM_000554.4	G	A/G	missense	ALA,THR	200/300		598
A6		17	61972644	CSH1	NM_001317.5	T	C/T	intron	none	NA	NA	
A6		17	61973682	CSH1	NM_001317.5	T	G/T	intron	none	NA	NA	
A6		8	2886788	CSMD1	NM_033225.5	C	A/C	intron	none	NA	NA	
A6		8	3253752	CSMD1	NM_033225.5	T	A/T	missense	SER,CYS	853/3565		2557
A6		1	34286026	CSMD2	NM_052896.3	A	A/T	intron	none	NA	NA	
A6		8	113347537	CSMD3	NM_052900.2	G	G/T	intron	none	NA	NA	
A6		8	113516286	CSMD3	NM_052900.2	A	A/T	intron	none	NA	NA	
A6		19	1954014	CSNK1G2	NM_001319.6	C	C/T	intron	none	NA	NA	
A6		8	68090027	CSPP1	NM_024790.6	G	G/T	intron	none	NA	NA	
A6		11	19209620	CSRP3	NM_003476.4	G	A/G	intron	none	NA	NA	
A6	X		120009295	CT47B1	NM_001145718.1	T	C/C	missense	GLN,ARG	77/300		230
A6		2	80831334	CTNNA2	NM_001164883.1	C	C/G	intron	none	NA	NA	
A6		5	11159557	CTNND2	NM_001332.2	C	C/T	intron	none	NA	NA	
A6		12	111733222	CUX2	NM_015267.3	C	A/C	intron	none	NA	NA	
A6		2	180809833	CWC22	NM_020943.2	C	A/C	utr-3	none	NA	NA	
A6		17	36977409	CWC25	NM_017748.3	C	C/T	intron	none	NA	NA	
A6		19	41515325	CYP2B6	NM_000767.4	G	G/T	intron	none	NA	NA	
A6		17	76688411	CYTH1	NM_004762.2	C	A/C	intron	none	NA	NA	
A6	X		85950042	DACH2	NM_001139514.1	G	T/T	missense	TRP,LEU	251/572		752
A6		2	136681995	DARS	NM_001349.2	T	A/T	missense	LYS,ILE	213/502		638
A6		9	122127100	DBC1	NM_014618.2	G	G/T	intron	none	NA	NA	
A6		14	24584764	DCAF11	NM_001163484.1	G	A/G	utr-5	none	NA	NA	
A6		6	24174761	DCDC2	NM_001195610.1	C	C/T	utr-3	none	NA	NA	
A6		11	30894999	DCDC5	NM_020869.3	C	A/C	intron	none	NA	NA	
A6		11	30900401	DCDC5	NM_020869.3	C	C/G	intron	none	NA	NA	
A6		4	155180802	DCHS2	NM_017639.3	T	A/T	coding-synonymous	none	1773/2917		5319
A6		7	50607556	DDC	NM_000790.3	C	A/C	intron	none	NA	NA	
A6		1	153907297	DENND4B	NM_014856.2	C	C/T	coding-synonymous	none	904/1497		2712
A6		20	37631438	DHX35	NM_001190809.1	C	A/C	stop-gained	SER,stop	229/673		686
A6	X		33146120	DMD	NM_000109.3	A	G/G	intron	none	NA	NA	
A6		19	46287514	DMWD	NM_004943.1	C	A/C	missense	GLY,VAL	671/675		2012
A6		12	124289427	DNAH10	NM_207437.3	G	G/T	missense	VAL,LEU	825/4472		2473

A6	7	21584506	DNAH11	NM_003777.3	G	A/G	intron	none	NA	NA
A6	1	225528445	DNAH14	NM_001373.1	G	A/G	intron	none	NA	NA
A6	5	13922163	DNAH5	NM_001369.2	T	A/T	intron	none	NA	NA
A6	6	38828301	DNAH8	NM_001206927.1	A	A/G	coding-synonymous	none	2009/4708	6027
A6	3	132196751	DNAJC13	NM_015268.3	A	A/G	intron	none	NA	NA
A6	3	132249689	DNAJC13	NM_015268.3	T	C/T	intron	none	NA	NA
A6	1	172051115	DNM3	NM_001136127.1	C	C/T	intron	none	NA	NA
A6	19	38706881	DPF1	NM_001135155.1	C	C/G	missense	GLY,ALA	293/415	878
A6	7	154645419	DPP6	NM_001039350.1.	G	C/G	intron	none	NA	NA
A6	18	28604349	DSC3	NM_001941.3	A	A/C	missense	ILE,MET	247/897	741
A6	15	45396502	DUOX2	NM_014080.4	C	A/C	missense	ARG,LEU	799/1549	2396
A6	19	5790049	DUS3L	NM_001161619.1	C	A/C	intron	none	NA	NA
A6	6	292621	DUSP22	NM_020185.3	G	A/G	intron	none	NA	NA
A6	6	311818	DUSP22	NM_020185.3	G	G/T	intron	none	NA	NA
A6	11	103128281	DYNC2H1	NM_001080463.1	A	A/T	intron	none	NA	NA
A6	5	158139095	EBF1	NM_024007.3	C	A/C	intron	none	NA	NA
A6	1	184718653	EDEM3	NM_025191.3	G	C/G	intron	none	NA	NA
A6	11	65638647	EFEMP2	NM_016938.4	G	A/G	coding-synonymous	none	116/444	348
A6	7	55266477	EGFR	NM_005228.3	C	A/C	coding-synonymous	none	923/1211	2769
A6	3	150293456	EIF2A	NM_032025.3	G	C/G	missense	GLN,HIS	468/586	1404
A6	2	88882830	EIF2AK3	NM_004836.5	T	C/T	intron	none	NA	NA
A6	X	129204852	ELF4	NM_001127197.1	G	A/A	intron	none	NA	NA
A6	15	44066347	ELL3	NM_025165.2	G	A/G	intron	none	NA	NA
A6	11	107521590	ELMOD1	NM_001130037.1	T	A/T	intron	none	NA	NA
A6	4	141456930	ELMOD2	NM_153702.3	A	A/G	missense	LYS,ARG	72/294	215
A6	2	85598231	ELMOD3	NM_001135021.1	G	A/G	missense	ARG,GLN	128/382	383
A6	1	79358884	ELTD1	NM_022159.3	G	G/T	intron	none	NA	NA
A6	5	49699258	EMB	NM_198449.2	C	C/T	missense	VAL,MET	211/328	631
A6	18	2892465	EMILIN2	NM_032048.2	C	C/T	coding-synonymous	none	780/1054	2340
A6	4	111397606	ENPEP	NM_001977.3	C	A/C	stop-gained	TYR,stop	12/958	36
A6	18	5443939	EPB41L3	NM_012307.2	G	G/T	intron	none	NA	NA
A6	2	222429198	EPHA4	NM_004438.3	C	A/C	intron	none	NA	NA
A6	12	56487555	ERBB3	NM_001982.3	G	A/G	coding-synonymous	none	496/1343	1488
A6	2	212537922	ERBB4	NM_001042599.1	C	A/C	missense	LYS,ASN	561/1293	1683
A6	11	124626520	ESAM	NM_138961.2	G	G/T	missense	PRO,HIS	123/391	368
A6	18	19119998	ESCO1	NM_052911.2	C	C/T	intron	none	NA	NA
A6	7	13949287	ETV1	NM_001163147.1	C	C/G	missense	ASP,HIS	281/455	841
A6	17	74011051	EVPL	NM_001988.2	T	A/T	missense	GLN,LEU	723/2034	2168
A6	5	76028649	F2R	NM_001992.3	G	G/T	missense	ARG,LEU	200/426	599
A6	9	130287561	FAM129B	NM_001035534.1	A	A/G	intron	none	NA	NA
A6	10	61029581	FAM13C	NM_001001971.2	C	A/C	intron	none	NA	NA
A6	5	175540656	FAM153B	NM_001265615.1	G	C/C	intron	none	NA	NA
A6	14	45478088	FAM179B	NM_015091.2	A	A/G	intron	none	NA	NA

A6	4	2664997	FAM193A	NM_001256666.1	A	A/T	coding-synonymous	none	396/1266		1188
A6	12	62261077	FAM19A2	NM_178539.4	G	A/G	intron	none	NA	NA	
A6	10	47910860	FAM21B	NM_018232.1	C	C/T	intron	none	NA	NA	
A6	22	38993191	FAM227A	NM_001013647.1	C	A/C	intron	none	NA	NA	
A6	10	89118375	FAM22D	NM_001009610.1	C	C/T	intron	none	NA	NA	
A6	6	24865731	FAM65B	NM_014722.2	A	A/G	intron	none	NA	NA	
A6	5	135272567	FBXL21	NM_012159.4	C	C/T	missense	SER,LEU	95/435		284
A6	1	161518770	FCGR3A	NM_000569.6	G	A/G	intron	none	NA	NA	
A6	5	72378719	FCHO2	NM_001146032.1	T	A/T	intron	none	NA	NA	
A6	1	241680404	FH	NM_000143.3	A	A/G	intron	none	NA	NA	
A6	11	128628246	FLI1	NM_001167681.2	A	A/G	intron	none	NA	NA	
A6	1	146687239	FMO5	NM_001144829.1	G	A/G	intron	none	NA	NA	
A6	17	80698791	FN3K	NM_022158.3	G	G/T	intron	none	NA	NA	
A6	1	109280061	FNDC7	NM_001144937.1	A	A/G	intron	none	NA	NA	
A6	12	8200455	FOXJ2	NM_018416.2	G	G/T	intron	none	NA	NA	
A6	13	39263675	FREM2	NM_207361.4	G	C/G	missense	ASP,HIS	732/3170		2194
A6	14	66028436	FUT8	NM_004480.4	G	G/T	intron	none	NA	NA	
A6	14	31084728	G2E3	NM_017769.3	A	A/T	missense	TYR,PHE	616/707		1847
A6	7	151805093	GALNT11	NM_022087.2	T	C/T	intron	none	NA	NA	
A6	2	31360647	GALNT14	NM_001253826.1	C	A/C	intron	none	NA	NA	
A6	18	74980578	GALR1	NM_001480.3	G	G/T	missense	GLY,VAL	257/350		770
A6	17	9821353	GAS7	NM_001130831.1	G	A/G	stop-gained	GLN,stop	364/413		1090
A6	2	237074876	GBX2	NM_001485.2	G	C/G	missense	SER,CYS	243/349		728
A6	4	72618315	GC	NM_000583.3	C	A/C	missense	ALA,SER	439/475		1315
A6	22	25024067	GGT1	NM_001032364.2	C	A/C	coding-synonymous	none	452/570		1356
A6	5	42718813	GHR	NM_000163.4	A	A/T	missense	ASN,TYR	402/639		1204
A6	7	31017089	GHRHR	NM_000823.3	G	A/G	intron	none	NA	NA	
A6	3	172165793	GHSR	NM_004122.2	C	C/T	coding-synonymous	none	137/290		411
A6	8	41387945	GINS4	NM_032336.2	C	A/C	intron	none	NA	NA	
A6	9	6553523	GLDC	NM_000170.2	A	A/T	intron	none	NA	NA	
A6	9	6556316	GLDC	NM_000170.2	G	C/G	intron	none	NA	NA	
A6	12	57859013	GLI1	NM_001160045.1	C	C/G	missense	SER,CYS	42/979		125
A6	1	231396378	GNPAT	NM_014236.3	A	A/G	coding-synonymous	none	129/681		387
A6	3	37343725	GOLGA4	NM_001172713.1	T	C/T	missense	MET,THR	401/2244		1202
A6	7	107114489	GPR22	NM_001161520.1	C	A/C	intron	none	NA	NA	
A6	11	123479502	GRAMD1B	NM_020716.1	G	G/T	missense	ARG,LEU	407/739		1220
A6	2	11742421	GREB1	NM_014668.3	C	A/C	intron	none	NA	NA	
A6	8	145065599	GRINA	NM_000837.1	C	C/T	missense	PRO,SER	70/372		208
A6	10	121085972	GRK5	NM_005308.2	G	G/T	intron	none	NA	NA	
A6	5	70357247	GTF2H2	NM_001515.3	G	G/T	intron	none	NA	NA	
A6	4	144917343	GYPB	NM_002100.4	G	G/T	utr-3	none	NA	NA	
A6	14	23419649	HAUS4	NM_001166269.1	C	C/T	intron	none	NA	NA	
A6	12	48191783	HDAC7	NM_001098416.2	T	C/T	intron	none	NA	NA	

A6	7	18868631	HDAC9	NM_058176.2	G	C/G	intron	none	NA	NA
A6	3	124746397	HEG1	NM_020733.1	A	A/C	intron	none	NA	NA
A6	15	63935648	HERC1	NM_003922.3	C	C/T	coding-synonymous	none	3762/4862	11286
A6	15	72638711	HEXA	NM_000520.4	T	A/T	intron	none	NA	NA
A6	6	25726515	HIST1H2AA	NM_170745.3	G	G/T	missense	PRO,THR	81/132	241
A6	1	185834851	HMCN1	NM_031935.2	C	C/T	intron	none	NA	NA
A6	1	185951573	HMCN1	NM_031935.2	C	C/T	intron	none	NA	NA
A6	7	27222670	HOXA11	NM_005523.5	G	C/G	intron	none	NA	NA
A6	4	8295810	HTRA3	NM_053044.3	C	A/C	intron	none	NA	NA
A6	3	160093614	IFT80	NM_001190241.1	G	C/G	missense	THR,SER	5/641	14
A6	7	50358776	IKZF1	NM_001220765.1	G	G/T	intron	none	NA	NA
A6	8	79717127	IL7	NM_000880.3	G	G/T	intron	none	NA	NA
A6	1	153736715	INTS3	NM_023015.3	T	C/T	intron	none	NA	NA
A6	1	153741566	INTS3	NM_023015.3	G	A/G	intron	none	NA	NA
A6	8	95863735	INTS8	NM_017864.2	G	A/G	intron	none	NA	NA
A6	2	237415794	IQCA1	NM_024726.3	G	A/G	intron	none	NA	NA
A6	2	237415812	IQCA1	NM_024726.3	A	A/G	intron	none	NA	NA
A6	15	78765741	IREB2	NM_004136.2	T	A/T	intron	none	NA	NA
A6	1	209963039	IRF6	NM_001206696.1	T	A/T	coding-synonymous	none	289/373	867
A6	10	7615011	ITIH5	NM_001001851.2	G	C/G	intron	none	NA	NA
A6	10	133977969	JAKMIP3	NM_001105521.2	C	C/T	intron	none	NA	NA
A6	X	8503671	KAL1	NM_000216.2	T	A/A	coding-synonymous	none	601/681	1803
A6	3	124162748	KALRN	NM_001024660.3	G	A/G	intron	none	NA	NA
A6	12	4920098	KCNA6	NM_002235.3	C	A/C	missense	ASN,LYS	297/530	891
A6	1	210857470	KCNH1	NM_002238.3	C	C/T	missense	ARG,GLN	681/963	2042
A6	1	211280738	KCNH1	NM_002238.3	A	A/G	intron	none	NA	NA
A6	2	163369097	KCNH7	NM_033272.3	C	C/G	intron	none	NA	NA
A6	10	78729617	KCNMA1	NM_001014797.2	C	C/T	intron	none	NA	NA
A6	20	62062739	KCNQ2	NM_004518.4	C	C/T	intron	none	NA	NA
A6	6	18222102	KDM1B	NM_153042.3	C	C/T	intron	none	NA	NA
A6	X	44929264	KDM6A	NM_021140.2	C	T/T	coding-synonymous	none	788/1402	2364
A6	4	55958775	KDR	NM_002253.2	C	C/T	intron	none	NA	NA
A6	19	6416597	KHSRP	NM_003685.2	C	C/T	coding-synonymous	none	464/712	1392
A6	4	123097175	KIAA1109	NM_015312.3	G	G/T	intron	none	NA	NA
A6	7	86594376	KIAA1324L	NM_001142749.2	C	C/T	intron	none	NA	NA
A6	7	138603023	KIAA1549	NM_001164665.1	G	C/G	missense	THR,SER	450/1951	1349
A6	3	113378545	KIAA2018	NM_001009899.2	G	C/G	missense	GLN,GLU	662/2246	1984
A6	X	73959233	KIAA2022	NM_001008537.2	A	T/T	utr-3	none	NA	NA
A6	20	16488746	KIF16B	NM_001199865.1	C	C/G	splice-3	none	NA	NA
A6	10	91497252	KIF20B	NM_016195.2	A	A/G	missense	GLU,GLY	845/1781	2534
A6	19	55236667	KIR3DL3	NM_153443.3	G	A/G	intron	none	NA	NA
A6	11	126343429	KIRREL3	NM_001161707.1	G	A/G	intron	none	NA	NA
A6	12	9145089	KLRG1	NM_005810.3	C	C/G	intron	none	NA	NA

A6	10	134980908	KNDC1	NM_152643.6	C	A/C	coding-synonymous	none	42/1750	126
A6	17	39021015	KRT12	NM_000223.3	G	A/G	intron	none	NA	NA
A6	18	6961861	LAMA1	NM_005559.3	C	A/C	intron	none	NA	NA
A6	18	6964690	LAMA1	NM_005559.3	C	A/C	coding-synonymous	none	2436/3076	7308
A6	17	25975782	LGALS9	NM_002308.3	T	G/T	intron	none	NA	NA
A6	4	25005888	LGI2	NM_018176.3	G	G/T	missense	GLN,LYS	275/546	823
A6	5	38528953	LIFR	NM_001127671.1	C	C/G	intron	none	NA	NA
A6	1	226473936	LIN9	NM_001270409.1	A	A/G	intron	none	NA	NA
A6	10	90530848	LIPN	NM_001102469.1	A	A/G	intron	none	NA	NA
A6	1	165180063	LMX1A	NM_001174069.1	G	C/G	intron	none	NA	NA
A6	1	165218832	LMX1A	NM_001174069.1	G	C/G	coding-synonymous	none	103/383	309
A6	13	28155582	LNX2	NM_153371.3	C	A/C	missense	ASP,TYR	87/691	259
A6	1	82402377	LPHN2	NM_012302.2	T	A/T	intron	none	NA	NA
A6	7	133886379	LRGUK	NM_144648.1	G	A/G	intron	none	NA	NA
A6	1	70505206	LRRC7	NM_020794.2	C	C/G	coding-synonymous	none	1195/1538	3585
A6	2	238672775	LRRFIP1	NM_001137550.1	A	A/G	intron	none	NA	NA
A6	12	85531469	LRRIQ1	NM_001079910.1	A	A/G	intron	none	NA	NA
A6	12	40672094	LRRK2	NM_198578.3	G	A/G	intron	none	NA	NA
A6	2	77745719	LRRTM4	NM_001134745.1	T	A/T	missense	ILE,PHE	426/591	1276
A6	X	75649183	MAGEE1	NM_020932.2	C	A/A	missense	THR,ASN	287/958	860
A6	2	210343472	MAP2	NM_001039538.1	G	G/T	intron	none	NA	NA
A6	2	210574582	MAP2	NM_001039538.1	G	A/G	intron	none	NA	NA
A6	17	21201948	MAP2K3	NM_002756.4	G	G/T	intron	none	NA	NA
A6	3	186953593	MASP1	NM_001879.5	G	G/T	intron	none	NA	NA
A6	3	192516829	MB21D2	NM_178496.3	C	A/C	coding-synonymous	none	274/492	822
A6	10	13246108	MCM10	NM_018518.4	G	G/T	intron	none	NA	NA
A6	20	5932849	MCM8	NM_032485.4	G	G/T	intron	none	NA	NA
A6	1	85412607	MCOLN2	NM_153259.2	G	A/G	intron	none	NA	NA
A6	16	3294155	MEFV	NM_000243.2	T	G/T	intron	none	NA	NA
A6	14	21979417	METTL3	NM_019852.3	C	C/G	utr-5	none	NA	NA
A6	3	69990385	MITF	NM_000248.3	A	A/G	splice-3	none	NA	NA
A6	14	75506640	MLH3	NM_001040108.1	T	A/T	missense	THR,SER	1182/1454	3544
A6	7	104753240	MLL5	NM_018682.3	A	A/C	coding-synonymous	none	1679/1859	5037
A6	8	89081862	MMP16	NM_005941.4	C	A/C	intron	none	NA	NA
A6	12	132326284	MMP17	NM_016155.4	G	G/T	coding-synonymous	none	274/604	822
A6	X	14921095	MOSPD2	NM_001177475.1	A	T/T	coding-synonymous	none	119/456	357
A6	11	94209587	MRE11A	NM_005590.3	T	G/T	intron	none	NA	NA
A6	21	26973663	MRPL39	NM_017446.3	A	A/T	intron	none	NA	NA
A6	11	10624765	MRVI1	NM_001098579.2	G	G/T	coding-synonymous	none	585/905	1753
A6	16	70714811	MTSS1L	NM_138383.2	C	C/G	intron	none	NA	NA
A6	19	9065841	MUC16	NM_024690.2	T	C/T	missense	ASP,GLY	7202/14508	21605
A6	3	195506022	MUC4	NM_004532.5	C	C/G	intron	none	NA	NA
A6	3	195506499	MUC4	NM_004532.5	G	A/G	intron	none	NA	NA

A6	3	195507762	MUC4	NM_004532.5	T	G/T	intron	none	NA	NA	
A6	3	195507982	MUC4	NM_004532.5	T	G/T	intron	none	NA	NA	
A6	12	102053497	MYBPC1	NM_001254718.1	T	A/T	missense	ASP,GLU	580/1174		1740
A6	17	10408454	MYH1	NM_005963.3	G	G/T	intron	none	NA	NA	
A6	17	10545920	MYH3	NM_002470.3	T	C/T	missense	LYS,GLU	568/1941		1702
A6	16	46763643	MYLK3	NM_182493.2	C	A/C	intron	none	NA	NA	
A6	2	128331434	MYO7B	NM_001080527.1	C	C/G	intron	none	NA	NA	
A6	2	132241596	MZT2A	NM_001085365.1	T	C/T	utr-3	none	NA	NA	
A6	1	145299619	NBPF10	NM_001039703.4	T	A/T	intron	none	NA	NA	
A6	1	145303760	NBPF10	NM_001039703.4	G	C/G	intron	none	NA	NA	
A6	1	21804950	NBPF3	NM_001256416.1	A	A/G	intron	none	NA	NA	
A6	4	17838719	NCAPG	NM_022346.3	A	A/G	intron	none	NA	NA	
A6	2	240944531	NDUFA10	NM_004544.3	C	A/C	intron	none	NA	NA	
A6	18	9126953	NDUFV2	NM_021074.4	A	A/G	intron	none	NA	NA	
A6	2	152482013	NEB	NM_001164507.1	T	C/T	intron	none	NA	NA	
A6	4	170347383	NEK1	NM_001199397.1	G	A/G	missense	SER,LEU	963/1287		2888
A6	13	52701494	NEK5	NM_199289.1	A	A/C	intron	none	NA	NA	
A6	12	45004645	NELL2	NM_001145107.1	C	A/C	missense	GLY,VAL	568/867		1703
A6	15	73566423	NEO1	NM_001172623.1	A	A/G	intron	none	NA	NA	
A6	15	73567137	NEO1	NM_001172623.1	C	C/T	intron	none	NA	NA	
A6	4	113435955	NEUROG2	NM_024019.3	A	A/G	missense	VAL,ALA	226/273		677
A6	20	50070966	NFATC2	NM_001136021.2	G	C/G	intron	none	NA	NA	
A6	14	51289848	NIN	NM_016350.4	C	C/T	intron	none	NA	NA	
A6	16	69375015	NIP7	NM_001199434.1	A	A/T	intron	none	NA	NA	
A6	10	134598878	NKX6-2	NM_177400.2	G	G/T	missense	ALA,GLU	162/278		485
A6	22	38086692	NOL12	NM_024313.2	G	C/G	intron	none	NA	NA	
A6	18	31599352	NOL4	NM_001198546.1	T	C/T	missense	LYS,ARG	329/575		986
A6	1	26737235	none	none	C	C/G	near-gene-5	none	NA	NA	
A6	1	225965451	none	none	C	A/C	near-gene-5	none	NA	NA	
A6	1	248617181	none	none	C	C/T	near-gene-3	none	NA	NA	
A6	2	38963046	none	none	G	A/G	near-gene-3	none	NA	NA	
A6	2	89246624	none	none	A	A/T	intergenic	none	NA	NA	
A6	2	89986912	none	none	C	A/C	intergenic	none	NA	NA	
A6	2	132723976	none	none	G	C/G	intergenic	none	NA	NA	
A6	4	123091723	none	none	A	A/G	near-gene-5	none	NA	NA	
A6	7	117663348	none	none	T	A/T	intergenic	none	NA	NA	
A6	7	142008403	none	none	C	C/G	intergenic	none	NA	NA	
A6	9	115120692	none	none	C	C/T	intergenic	none	NA	NA	
A6	10	28924764	none	none	G	G/T	intergenic	none	NA	NA	
A6	10	96790768	none	none	C	A/C	intergenic	none	NA	NA	
A6	10	134760212	none	none	T	C/T	intergenic	none	NA	NA	
A6	12	9581952	none	none	C	C/T	intergenic	none	NA	NA	
A6	14	22580405	none	none	A	A/G	intergenic	none	NA	NA	

A6		14	22925535	none	none	A	A/G	intergenic	none	NA	NA	
A6		14	106610260	none	none	C	C/G	intergenic	none	NA	NA	
A6		14	107131623	none	none	T	C/T	intergenic	none	NA	NA	
A6		14	107170175	none	none	G	C/G	intergenic	none	NA	NA	
A6		15	20645639	none	none	G	G/T	intergenic	none	NA	NA	
A6		19	52658103	none	none	A	A/G	near-gene-3	none	NA	NA	
A6		20	29589953	none	none	G	G/T	intergenic	none	NA	NA	
A6		22	22988651	none	none	A	A/G	near-gene-5	none	NA	NA	
A6		22	23256264	none	none	G	G/T	intergenic	none	NA	NA	
A6		22	42898973	none	none	A	A/C	intergenic	none	NA	NA	
A6	X		48164175	none	none	C	T/T	intergenic	none	NA	NA	
A6	X		83480830	none	none	C	A/A	intergenic	none	NA	NA	
A6	X		142605315	none	none	C	T/T	near-gene-5	none	NA	NA	
A6		1	120467760	NOTCH2	NM_024408.3	T	C/T	intron	none	NA	NA	
A6		1	120548389	NOTCH2	NM_001200001.1	A	A/G	intron	none	NA	NA	
A6		18	21141421	NPC1	NM_000271.4	C	C/T	coding-synonymous	none	178/1279		534
A6		1	5993273	NPHP4	NM_015102.3	C	C/T	coding-synonymous	none	412/1427		1236
A6		9	35806520	NPR2	NM_003995.3	A	A/T	missense	TYR,PHE	835/1048		2504
A6		15	88679051	NTRK3	NM_001007156.2	C	A/C	intron	none	NA	NA	
A6		19	41191092	NUMBL	NM_004756.3	G	A/G	intron	none	NA	NA	
A6		9	138439073	OBP2A	NM_014582.2	G	G/T	stop-gained	GLU,stop	86/171		256
A6		1	228401896	OBSCN	NM_001098623.1	C	C/T	missense	PRO,LEU	427/7969		1280
A6		8	133041465	OC90	NM_001080399.2	G	G/T	intron	none	NA	NA	
A6		4	183673029	ODZ3	NM_001080477.1	C	C/T	missense	ARG,CYS	1236/2700		3706
A6		10	50954706	OGDHL	NM_001143996.1	C	C/T	intron	none	NA	NA	
A6		10	45799122	OR13A1	NM_001004297.2	C	A/C	missense	TRP,LEU	250/329		749
A6		11	4929020	OR51A7	NM_001004749.1	G	A/G	missense	VAL,ILE	141/313		421
A6		11	4661212	OR51D1	NM_001004751.2	G	G/T	coding-synonymous	none	64/325		192
A6		11	5172912	OR52A1	NM_012375.2	G	G/T	missense	ARG,SER	230/313		688
A6		11	59211561	OR5A1	NM_001004728.1	T	A/T	missense	VAL,GLU	307/316		920
A6		11	58170842	OR5B3	NM_001005469.1	C	A/C	missense	GLY,VAL	14/315		41
A6		3	97869007	OR5H14	NM_001005514.1	A	A/G	missense	MET,VAL	260/311		778
A6		11	55944738	OR5J2	NM_001005492.1	C	C/T	coding-synonymous	none	215/313		645
A6		11	55944777	OR5J2	NM_001005492.1	C	C/G	missense	ILE,MET	228/313		684
A6		12	31302358	OVOS2	XM_002343151.3	C	A/C	missense-near-splice	CYS,PHE	312/1395		935
A6		8	101725049	PABPC1	NM_002568.3	T	A/T	intron	none	NA	NA	
A6		1	17431497	PADI2	NM_007365.2	T	A/T	missense	GLU,VAL	51/666		152
A6		11	77103608	PAK1	NM_001128620.1	A	A/G	intron	none	NA	NA	
A6		9	112694122	PALM2	NM_001037293.2	G	G/T	intron	none	NA	NA	
A6		12	56726830	PAN2	NM_001127460.2	T	A/T	missense	MET,LEU	17/1203		49
A6		5	6742632	PAPD7	NM_001171805.1	G	G/T	missense	GLU,ASP	96/542		288
A6		1	176640106	PAPPA2	NM_020318.2	G	G/T	missense-near-splice	ARG,SER	664/1792		1992
A6		2	206050507	PARD3B	NM_057177.6	C	C/T	coding-synonymous	none	648/1137		1944

A6	4	75938107	PARM1	NM_015393.3	C	C/T	coding-synonymous	none	172/311		516
A6	13	25043223	PARP4	NM_006437.3	T	C/T	missense	GLN,ARG	691/1725		2072
A6	5	50117290	PARP8	NM_001178055.1	A	A/G	intron	none	NA	NA	
A6	3	122274364	PARP9	NM_001146102.1	C	A/C	missense	LYS,ASN	253/855		759
A6	20	21695362	PAX1	NM_001257096.1	C	C/T	utr-3	none	NA	NA	
A6	11	66627728	PC	NM_000920.3	C	C/T	intron	none	NA	NA	
A6	4	134072609	PCDH10	NM_020815.1	G	C/G	coding-synonymous	none	438/897		1314
A6	10	55569355	PCDH15	NM_001142769.1	G	A/G	intron	none	NA	NA	
A6	13	58240866	PCDH17	NM_001040429.2	A	A/T	missense	ASP,VAL	899/1160		2696
A6	4	138451349	PCDH18	NM_019035.3	C	A/C	missense	ASP,TYR	632/1136		1894
A6	8	52746311	PCMTD1	NM_052937.2	C	A/C	intron	none	NA	NA	
A6	9	78655279	PCSK5	NM_001190482.1	C	C/G	intron	none	NA	NA	
A6	1	144876089	PDE4DIP	NM_001198832.1	C	C/T	intron	none	NA	NA	
A6	4	55129912	PDGFRA	NM_006206.4	C	C/T	missense	PRO,LEU	149/1090		446
A6	16	334529	PDIA2	NM_006849.2	G	C/G	coding-synonymous	none	114/526		342
A6	1	249212205	PGBD2	NM_001017434.1	G	G/T	missense	LYS,ASN	223/342		669
A6	4	129776992	PHF17	NM_024900.3	A	A/T	intron	none	NA	NA	
A6	22	45316287	PHF21B	NM_001135862.2	C	A/C	coding-synonymous	none	68/490		204
A6	15	68438876	PIAS1	NM_016166.1	G	A/G	intron	none	NA	NA	
A6	17	8793251	PIK3R5	NM_001142633.2	G	A/G	intron	none	NA	NA	
A6	2	28814595	PLB1	NM_001170585.1	A	A/G	missense	ASP,GLY	708/1448		2123
A6	2	179348068	PLEKHA3	NM_019091.3	A	A/T	intron	none	NA	NA	
A6	5	151562	PLEKHG4B	NM_052909.3	C	C/T	intron	none	NA	NA	
A6	3	145804503	PLOD2	NM_000935.2	T	A/T	intron	none	NA	NA	
A6	2	86333007	POLR1A	NM_015425.3	C	C/T	utr-5	none	NA	NA	
A6	10	79751053	POLR3A	NM_007055.3	T	G/T	intron	none	NA	NA	
A6	5	89797740	POLR3G	NM_006467.2	A	A/G	missense	LYS,GLU	125/224		373
A6	7	72400529	POM121	NM_001257190.1	G	G/T	missense	LEU,PHE	120/1000		360
A6	14	77765073	POMT2	NM_013382.5	C	A/C	missense	ARG,LEU	322/751		965
A6	2	132021860	POTEE	NM_001083538.1	T	C/T	coding-synonymous	none	944/1076		2832
A6	1	167358654	POU2F1	NM_001198783.1	A	A/G	intron	none	NA	NA	
A6	7	94827692	PPP1R9A	NM_001166160.1	G	G/T	missense	VAL,LEU	596/1375		1786
A6	12	120110698	PRKAB1	NM_006253.4	G	G/T	intron	none	NA	NA	
A6	19	47193030	PRKD2	NM_001079880.1	G	A/G	intron	none	NA	NA	
A6	8	48695009	PRKDC	NM_001081640.1	C	C/G	missense	ALA,PRO	3776/4098		11325
A6	5	35089913	PRLR	NM_000949.5	C	A/C	intron	none	NA	NA	
A6	9	33796858	PRSS3	NM_001197097.2	C	A/C	intron	none	NA	NA	
A6	9	33797783	PRSS3	NM_001197097.2	A	A/T	intron	none	NA	NA	
A6	9	79465629	PRUNE2	NM_015225.2	G	A/G	intron	none	NA	NA	
A6	1	186648190	PTGS2	NM_000963.2	A	A/G	missense-near-splice	SER,PRO	105/605		313
A6	8	142441239	PTP4A3	NM_007079.2	G	G/T	utr-3	none	NA	NA	
A6	18	8088612	PTPRM	NM_001105244.1	G	G/T	intron	none	NA	NA	
A6	11	125769441	PUS3	NM_001134793.1	A	A/C	missense	LYS,GLN	60/300		178

A6		1	158908034	PYHIN1	NM_152501.4	C	A/C	intron	none	NA	NA	
A6		20	42979472	R3HDML	NM_178491.2	C	A/C	utr-3	none	NA	NA	
A6		12	70133580	RAB3IP	NM_022456.3	G	A/G	intron	none	NA	NA	
A6		17	5257800	RABEP1	NM_001083585.1	T	C/T	intron	none	NA	NA	
A6		20	1210413	RAD21L1	NM_001136566.2	A	A/C	intron	none	NA	NA	
A6		12	4668167	RAD51AP1	NM_001130862.1	A	A/T	utr-3	none	NA	NA	
A6		18	9533422	RALBP1	NM_006788.3	A	A/C	missense	GLU,ASP	515/656		1545
A6		8	85097432	RALYL	NM_001100391.1	C	A/C	intron	none	NA	NA	
A6		7	127963518	RBM28	NM_001166135.1	A	A/G	intron	none	NA	NA	
A6		2	178984892	RBM45	NM_152945.2	C	C/T	intron	none	NA	NA	
A6		1	167663442	RCSD1	NM_052862.3	G	G/T	missense	SER,ILE	126/417		377
A6		2	79384678	REG3A	NM_002580.2	G	G/T	intron	none	NA	NA	
A6		5	98115444	RGMB	NM_001012761.2	C	C/T	coding-synonymous	none	140/479		420
A6		2	107042610	RGPD3	NM_001144013.1	G	G/T	intron	none	NA	NA	
A6		4	3417743	RGS12	NM_002926.3	C	C/T	coding-synonymous	none	774/1377		2322
A6		5	63871594	RGS7BP	NM_001029875.1	G	C/G	intron	none	NA	NA	
A6		1	156351346	RHBG	NM_001256395.1	G	G/T	intron	none	NA	NA	
A6		1	113246249	RHOC	NM_001042678.1	G	A/G	intron	none	NA	NA	
A6		9	5339672	RLN1	NM_006911.2	G	G/T	coding-synonymous	none	25/186		75
A6		16	2000751	RPL3L	NM_005061.2	T	G/T	intron	none	NA	NA	
A6		1	153963293	RPS27	NM_001030.4	C	C/T	intron	none	NA	NA	
A6		10	70139112	RUFY2	NM_001042417.1	T	G/T	intron	none	NA	NA	
A6		1	237814753	RYR2	NM_001035.2	A	A/G	coding-synonymous	none	2592/4968		7776
A6		1	237994936	RYR2	NM_001035.2	G	C/G	intron	none	NA	NA	
A6		15	33962623	RYR3	NM_001036.3	T	G/T	missense	VAL,GLY	1909/4871		5726
A6		20	50408036	SALL4	NM_020436.3	C	C/T	missense	ARG,HIS	329/1054		986
A6		7	92763106	SAMD9L	NM_152703.2	C	C/T	missense	GLU,LYS	727/1585		2179
A6		6	148846552	SASH1	NM_015278.3	G	C/G	intron	none	NA	NA	
A6		6	25665242	SCGN	NM_006998.3	C	A/C	missense	SER,ARG	106/277		318
A6		1	161310275	SDHC	NM_001035511.1	G	A/G	intron	none	NA	NA	
A6		22	30866244	SEC14L3	NM_001257378.1	T	A/T	splice-3	none	NA	NA	
A6		1	169676488	SELL	NM_000655.4	T	A/T	missense	GLN,LEU	205/386		614
A6		12	48465592	SENP1	NM_001267594.1	C	C/G	intron	none	NA	NA	
A6		11	94923024	SESN3	NM_144665.2	C	A/C	coding-synonymous	none	148/493		444
A6		3	9483899	SETD5	NM_001080517.1	C	A/C	missense	PHE,LEU	349/1443		1047
A6		22	26743936	SEZ6L	NM_001184773.1	G	C/G	intron	none	NA	NA	
A6		10	7239528	SFMBT2	NM_001018039.1	G	A/G	coding-synonymous	none	560/895		1680
A6		10	7262563	SFMBT2	NM_001018039.1	G	A/G	intron	none	NA	NA	
A6		10	81319055	SFTPA2	NM_001098668.2	T	C/T	intron	none	NA	NA	
A6		7	94257413	SGCE	NM_001099400.1	T	C/T	intron	none	NA	NA	
A6		1	67091458	SGIP1	NM_032291.2	G	A/G	intron	none	NA	NA	
A6	X		19764369	SH3KBP1	NM_001024666.2	C	A/A	intron	none	NA	NA	
A6		5	145428867	SH3RF2	NM_152550.3	C	C/G	intron	none	NA	NA	

A6	19	436344	SHC2	NM_012435.2	C	A/C	intron	none	NA	NA
A6	13	26620941	SHISA2	NM_001007538.1	T	C/T	missense	THR,ALA	200/296	598
A6	6	146215230	SHPRH	NM_001042683.2	C	C/T	intron	none	NA	NA
A6	20	45245376	SLC13A3	NM_001011554.2	C	C/G	intron	none	NA	NA
A6	6	25862162	SLC17A3	NM_001098486.1	C	C/T	coding-synonymous	none	133/499	399
A6	19	15067418	SLC1A6	NM_005071.1	T	A/T	missense	ILE,PHE	347/565	1039
A6	11	63078399	SLC22A10	NM_001039752.3	G	G/T	intron	none	NA	NA
A6	X	129505645	SLC25A14	NM_003951.2	T	C/C	intron	none	NA	NA
A6	7	107414703	SLC26A3	NM_000111.2	A	A/T	intron	none	NA	NA
A6	8	118184767	SLC30A8	NM_001172811.1	A	A/T	intron	none	NA	NA
A6	2	162834419	SLC4A10	NM_001178015.1	C	C/T	intron	none	NA	NA
A6	2	220498747	SLC4A3	NM_005070.3	G	A/G	intron	none	NA	NA
A6	1	110709818	SLC6A17	NM_001010898.2	G	G/T	coding-synonymous	none	89/728	267
A6	2	40342669	SLC8A1	NM_001112800.1	G	A/G	coding-synonymous	none	877/969	2631
A6	12	20903935	SLCO1C1	NM_001145944.1	T	A/T	intron	none	NA	NA
A6	5	101606316	SLCO4C1	NM_180991.4	G	A/G	intron	none	NA	NA
A6	7	128850276	SMO	NM_005631.4	G	A/G	coding-synonymous	none	513/788	1539
A6	22	31486180	SMTN	NM_001207017.1	C	C/T	intron	none	NA	NA
A6	2	88409988	SMYD1	NM_198274.3	C	A/C	missense	PRO,HIS	477/491	1430
A6	2	73449776	SMYD5	NM_006062.2	A	A/G	intron	none	NA	NA
A6	7	26404264	SNX10	NM_001199835.1	T	G/T	intron	none	NA	NA
A6	2	27597210	SNX17	NM_001267059.1	T	C/T	intron	none	NA	NA
A6	10	108458849	SORCS1	NM_001013031.2	C	A/C	intron	none	NA	NA
A6	10	106907253	SORCS3	NM_014978.1	A	A/T	intron	none	NA	NA
A6	X	142596615	SPANXN3	NM_001009609.2	T	A/A	utr-3	none	NA	NA
A6	15	97328127	SPATA8	NM_173499.3	C	C/G	intron	none	NA	NA
A6	5	35792597	SPEF2	NM_024867.3	G	G/T	intron	none	NA	NA
A6	5	80770373	SSBP2	NM_001256732.1	T	A/T	coding-synonymous	none	147/370	441
A6	7	149509586	SSPO	NM_198455.2	C	A/C	intron	none	NA	NA
A6	12	104025431	STAB2	NM_017564.9	T	C/T	coding-synonymous	none	181/2552	543
A6	7	87913530	STEAP4	NM_001205315.1	G	G/T	missense	GLN,LYS	19/460	55
A6	7	23792150	STK31	NM_001260504.1	G	G/T	intron	none	NA	NA
A6	7	134936741	STRA8	NM_182489.1	G	C/G	intron	none	NA	NA
A6	11	22848866	SVIP	NM_148893.1	C	A/C	missense-near-splice	ALA,SER	36/78	106
A6	6	152651132	SYNE1	NM_033071.3	G	G/T	coding-synonymous	none	4825/8750	14475
A6	18	40857031	SYT4	NM_020783.3	C	A/C	intron	none	NA	NA
A6	17	34149744	TAF15	NM_003487.3	G	A/G	missense	GLU,LYS	128/590	382
A6	17	34160853	TAF15	NM_003487.3	G	A/G	intron	none	NA	NA
A6	5	9629818	TAS2R1	NM_019599.2	G	A/G	coding-synonymous	none	109/300	327
A6	X	153642463	TAZ	NM_000116.3	T	C/C	coding-synonymous	none	132/293	396
A6	3	176744032	TBL1XR1	NM_024665.4	A	A/G	intron	none	NA	NA
A6	5	133482154	TCF7	NM_001134851.2	G	C/G	utr-3	none	NA	NA
A6	14	20857465	TEP1	NM_007110.4	G	C/G	intron	none	NA	NA

A6	17	56643176	TEX14	NM_001201457.1	T	A/T	missense	LYS,ILE	1345/1498		4034
A6	1	92193351	TGFBR3	NM_001195683.1	C	C/T	coding-synonymous	none	250/851		750
A6	5	156390240	TIMD4	NM_001146726.1	C	C/G	utr-5	none	NA	NA	
A6	9	103271473	TMEFF1	NM_001198812.1	A	A/T	intron	none	NA	NA	
A6	2	192863735	TMEFF2	NM_016192.2	T	C/T	intron	none	NA	NA	
A6	3	190165386	TMEM207	NM_207316.1	G	A/G	intron	none	NA	NA	
A6	15	43427894	TMEM62	NM_024956.3	C	C/G	intron	none	NA	NA	
A6	21	19701697	TMPRSS15	NM_002772.2	T	G/T	intron	none	NA	NA	
A6	12	83290334	TMTC2	NM_152588.1	T	A/T	coding-synonymous	none	464/837		1392
A6	14	51706920	TMX1	NM_030755.4	C	A/C	utr-5	none	NA	NA	
A6	1	201330392	TNNT2	NM_000364.2	A	A/C	intron	none	NA	NA	
A6	1	175293821	TNR	NM_003285.2	C	A/C	intron	none	NA	NA	
A6	8	144391844	TOP1MT	NM_001258446.1	T	G/T	intron	none	NA	NA	
A6	11	18044507	TPH1	NM_004179.2	G	G/T	intron	none	NA	NA	
A6	9	35689902	TPM2	NM_003289.3	C	A/C	utr-5	none	NA	NA	
A6	2	1507740	TPO	NM_000547.5	G	C/G	missense	GLY,ARG	803/934		2407
A6	5	666061	TPPP	NM_007030.2	G	A/G	intron	none	NA	NA	
A6	21	10906855	TPTE	NM_199259.2	G	C/G	utr-3	none	NA	NA	
A6	21	10910205	TPTE	NM_199259.2	C	C/G	intron	none	NA	NA	
A6	12	73015586	TRHDE	NM_013381.2	C	A/C	intron	none	NA	NA	
A6	8	67066656	TRIM55	NM_033058.2	T	C/T	intron	none	NA	NA	
A6	8	67066697	TRIM55	NM_033058.2	G	A/G	intron	none	NA	NA	
A6	7	100470924	TRIP6	NM_003302.2	G	A/G	coding-synonymous	none	477/477		1430
A6	8	72969151	TRPA1	NM_007332.2	C	C/G	splice-5	none	NA	NA	
A6	9	73250164	TRPM3	NM_001007471.2	G	C/G	stop-gained	SER,stop	532/1708		1595
A6	8	116430447	TRPS1	NM_014112.2	C	A/C	intron	none	NA	NA	
A6	3	180327637	TTC14	NM_001042601.2	A	A/T	utr-3	none	NA	NA	
A6	22	28504176	TTC28	NM_001145418.1	G	A/G	stop-gained	GLN,stop	553/2482		1657
A6	2	179558466	TTN	NM_001256850.1	A	A/G	intron	none	NA	NA	
A6	2	132240096	TUBA3D	NM_080386.3	T	C/T	intron	none	NA	NA	
A6	5	158697336	UBLCP1	NM_145049.3	A	A/G	intron	none	NA	NA	
A6	11	5530215	UBQLN3	NM_017481.2	G	G/T	missense	LEU,ILE	192/656		574
A6	11	89819886	UBTFL1	NM_001143975.1	C	A/C	missense	ARG,SER	257/394		769
A6	13	96648352	UGGT2	NM_020121.3	T	C/T	coding-synonymous	none	265/1517		795
A6	15	54614065	UNC13C	NM_001080534.1	G	G/T	intron	none	NA	NA	
A6	16	21985343	UQCRC2	NM_003366.2	T	C/T	intron	none	NA	NA	
A6	1	215956069	USH2A	NM_206933.2	T	C/T	intron	none	NA	NA	
A6	17	58313507	USP32	NM_032582.3	C	A/C	missense	VAL,PHE	411/1605		1231
A6	17	58343557	USP32	NM_032582.3	G	C/G	intron	none	NA	NA	
A6	15	50838702	USP50	NM_203494.4	G	C/G	coding-synonymous	none	7/335		21
A6	17	5033825	USP6	NM_004505.2	G	A/G	intron	none	NA	NA	
A6	6	144742866	UTRN	NM_007124.2	C	A/C	intron	none	NA	NA	
A6	10	118892106	VAX1	NM_199131.2	A	A/T	intron	none	NA	NA	

A6	10	135053187	VENTX	NM_014468.2	T	G/T	missense	SER,ARG	83/259		249
A6	2	63609097	WDPCP	NM_015910.5	C	A/C	missense	SER,ILE	523/747		1568
A6	4	177032833	WDR17	NM_170710.4	C	A/C	coding-synonymous	none	58/1323		174
A6	4	177098628	WDR17	NM_170710.4	A	A/G	coding-synonymous	none	1224/1323		3672
A6	10	1170135	WDR37	NM_014023.3	G	A/G	intron	none	NA	NA	
A6	1	85570319	WDR63	NM_145172.3	A	A/T	intron	none	NA	NA	
A6	5	37381647	WDR70	NM_018034.2	A	A/G	intron	none	NA	NA	
A6	7	116937642	WNT2	NM_003391.2	A	A/G	intron	none	NA	NA	
A6	2	168100314	XIRP2	NM_001079810.3	G	A/G	intron	none	NA	NA	
A6	14	75264720	YLPM1	NM_019589.2	A	A/G	missense	GLN,ARG	907/2147		2720
A6	3	167031868	ZBBX	NM_001199201.1	T	C/T	coding-synonymous	none	437/840		1311
A6	5	843591	ZDHHC11	NM_024786.2	A	A/C	intron	none	NA	NA	
A6	8	77754803	ZFHX4	NM_024721.4	C	C/T	intron	none	NA	NA	
A6	20	50769812	ZFP64	NM_018197.2	G	A/G	missense	ARG,CYS	307/682		919
A6	4	2341308	ZFYVE28	NM_001172656.1	C	A/C	coding-synonymous	none	131/858		393
A6	19	22154999	ZNF208	NM_007153.3	T	A/T	missense	LYS,ILE	946/1281		2837
A6	19	44891126	ZNF285	NM_152354.3	T	A/T	coding-synonymous	none	427/591		1281
A6	19	44891261	ZNF285	NM_152354.3	A	A/T	missense	ASP,GLU	382/591		1146
A6	19	44891269	ZNF285	NM_152354.3	C	C/G	missense	GLY,ARG	380/591		1138
A6	19	44891291	ZNF285	NM_152354.3	A	A/G	coding-synonymous	none	372/591		1116
A6	10	64159430	ZNF365	NM_014951.2	C	A/C	missense	SER,TYR	369/408		1106
A6	19	12462739	ZNF442	NM_030824.2	A	A/G	intron	none	NA	NA	
A6	3	40574228	ZNF621	NM_001098414.1	G	G/T	missense	VAL,LEU	323/440		967
A6	19	53740113	ZNF677	NM_182609.2	T	A/T	utr-3	none	NA	NA	
A6	19	53453366	ZNF816	NM_001031665.2	G	C/G	missense	ASN,LYS	554/652		1662
A6	19	52568357	ZNF841	NM_001136499.1	A	A/T	utr-3	none	NA	NA	
A6	19	23557495	ZNF91	NM_003430.2	C	C/T	coding-synonymous	none	34/1192		102
A6	7	50023160	ZPBP	NM_001159878.1	G	G/T	intron	none	NA	NA	
A7	12	58150783	40977	NM_138396.5	G	C/G	missense	TRP,CYS	143/347		429
A7	22	19707736	41157	NM_001009939.2	G	G/T	missense	GLY,TRP	122/347		364
A7	15	67524142	AAGAB	NM_024666.3	A	A/G	intron	none	NA	NA	
A7	17	66932987	ABCA8	NM_007168.2	G	G/T	intron	none	NA	NA	
A7	7	20698472	ABCB5	NM_001163941.1	G	A/G	intron	none	NA	NA	
A7	16	48239246	ABCC11	NM_032583.3	G	G/T	intron	none	NA	NA	
A7	11	17418566	ABCC8	NM_000352.3	G	G/T	missense	PRO,GLN	1339/1582		4016
A7	7	152513619	ACTR3B	NM_001040135.2	G	A/G	coding-synonymous	none	162/349		486
A7	5	33616176	ADAMTS12	NM_030955.2	A	A/T	coding-synonymous-	none	715/1595		2145
A7	15	79067211	ADAMTS7	NM_014272.3	A	A/G	intron	none	NA	NA	
A7	9	136412145	ADAMTSL2	NM_001145320.1	G	G/T	intron	none	NA	NA	
A7	5	7626398	ADCY2	NM_020546.2	G	G/T	missense	ARG,LEU	230/1092		689
A7	5	7784611	ADCY2	NM_020546.2	G	G/T	intron	none	NA	NA	
A7	6	147122299	ADGB	NM_024694.3	T	A/T	intron	none	NA	NA	
A7	8	67361246	ADHFE1	NM_144650.2	G	A/G	intron	none	NA	NA	

A7	7	150839002	AGAP3	NM_031946.4	G	A/G	missense	GLU,LYS	608/912		1822
A7	18	14837724	ANKRD30B	NM_001145029.1	A	A/T	intron	none	NA	NA	
A7	2	97779601	ANKRD36	NM_001164315.1	A	A/G	missense	HIS,ARG	42/1942		125
A7	2	97808242	ANKRD36	NM_001164315.1	G	G/T	intron	none	NA	NA	
A7	7	117868094	ANKRD7	NM_019644.3	T	C/T	intron	none	NA	NA	
A7	12	99478634	ANKS1B	NM_001204066.1	C	A/C	intron	none	NA	NA	
A7	11	26555954	ANO3	NM_031418.2	G	C/G	intron	none	NA	NA	
A7	10	81917448	ANXA11	NM_001157.2	G	C/G	missense	ILE,MET	469/506		1407
A7	2	201485963	AOX1	NM_001159.3	A	A/T	coding-synonymous	none	665/1339		1995
A7	2	68717417	APLF	NM_173545.2	T	C/T	intron	none	NA	NA	
A7	2	21233208	APOB	NM_000384.2	T	A/T	missense	MET,LEU	2178/4564		6532
A7	20	61907905	ARFGAP1	NM_018209.2	A	A/G	missense	LYS,GLU	82/407		244
A7	11	110461322	ARHGAP20	NM_001258415.1	C	C/G	intron	none	NA	NA	
A7	20	37266335	ARHGAP40	NM_001164431.1	C	C/G	intron	none	NA	NA	
A7	12	57869416	ARHGAP9	NM_001080156.1	C	C/G	intron	none	NA	NA	
A7	3	153958134	ARHGEF26	NM_001251962.1	G	A/G	intron	none	NA	NA	
A7	11	13408327	ARNTL	NM_001030272.1	A	A/C	utr-3	none	NA	NA	
A7	6	101214587	ASCC3	NM_006828.2	C	C/T	intron	none	NA	NA	
A7	4	156775539	ASIC5	NM_017419.2	C	A/C	intron	none	NA	NA	
A7	1	176903281	ASTN1	NM_004319.1	C	A/C	intron	none	NA	NA	
A7	2	25966031	ASXL2	NM_018263.4	T	G/T	missense	SER,ARG	1059/1436		3175
A7	7	117062360	ASZ1	NM_130768.2	C	C/T	missense	ALA,THR	87/476		259
A7	11	108202807	ATM	NM_000051.3	C	C/T	intron	none	NA	NA	
A7	1	160105408	ATP1A2	NM_000702.3	G	G/T	intron	none	NA	NA	
A7	5	172410859	ATP6V0E1	NM_003945.3	C	A/C	utr-5	none	NA	NA	
A7	3	142177844	ATR	NM_001184.3	T	A/T	missense	THR,SER	2487/2645		7459
A7	6	69685289	BAI3	NM_001704.2	G	C/G	intron	none	NA	NA	
A7	19	41916597	BCKDHA	NM_000709.3	C	C/G	missense	PRO,ARG	55/446		164
A7	6	56879893	BEND6	NM_152731.2	A	A/G	intron	none	NA	NA	
A7	20	55840861	BMP7	NM_001719.2	G	A/G	coding-synonymous	none	106/432		318
A7	14	105718881	BRF1	NM_001242786.1	G	C/G	coding-synonymous	none	54/585		162
A7	5	180431783	BTNL3	NM_197975.2	A	A/G	intron	none	NA	NA	
A7	12	40040152	C12orf40	NM_001031748.2	A	A/G	missense	HIS,ARG	75/653		224
A7	18	5196944	C18orf42	NM_001145194.1	C	A/C	intron	none	NA	NA	
A7	1	170916826	C1orf129	NM_001163629.1	G	C/G	intron	none	NA	NA	
A7	20	35737057	C20orf132	NM_152503.4	C	A/C	coding-synonymous	none	985/1053		2926
A7	20	3312939	C20orf194	NM_001009984.2	C	A/C	missense	VAL,PHE	351/1178		1051
A7	5	41904551	C5orf51	NM_175921.4	G	A/G	missense	GLU,LYS	28/295		82
A7	5	126387434	C5orf63	NM_001164478.1	G	A/G	utr-3	none	NA	NA	
A7	19	49142957	CA11	NM_001217.3	G	G/T	intron	none	NA	NA	
A7	16	1270177	CACNA1H	NM_001005407.1	G	G/T	missense	ARG,LEU	2076/2348		6227
A7	7	81978827	CACNA2D1	NM_000722.2	G	C/G	intron	none	NA	NA	
A7	1	174969189	CACYBP	NM_001007214.1	G	A/G	intron	none	NA	NA	

A7	3	62739345	CADPS	NM_003716.3	C	C/T	missense	ARG,HIS	220/1354		659
A7	19	47108950	CALM3	NM_005184.2	G	G/T	intron	none	NA	NA	
A7	7	94139434	CASD1	NM_022900.4	T	A/T	missense	ILE,ASN	13/798		38
A7	3	105397516	CBLB	NM_170662.3	A	A/C	intron	none	NA	NA	
A7	17	77808469	CBX4	NM_003655.2	C	C/T	coding-synonymous	none	324/561		972
A7	11	124845037	CCDC15	NM_025004.2	A	A/G	missense	LYS,GLU	188/952		562
A7	12	119937989	CCDC60	NM_178499.3	C	A/C	intron	none	NA	NA	
A7	2	55536345	CCDC88A	NM_001135597.1	C	C/G	missense	GLU,ASP	1374/1871		4122
A7	12	28605644	CCDC91	NM_018318.3	A	A/C	intron	none	NA	NA	
A7	17	32647868	CCL8	NM_005623.2	G	G/T	missense	ARG,ILE	81/100		242
A7	16	30364574	CD2BP2	NM_001243646.1	C	C/T	coding-synonymous	none	281/342		843
A7	17	62007248	CD79B	NM_000626.2	C	A/C	missense-near-splice	GLY,VAL	144/230		431
A7	11	64605505	CDC42BPG	NM_017525.2	A	A/T	intron	none	NA	NA	
A7	5	19473685	CDH18	NM_001167667.1	C	A/C	utr-3	none	NA	NA	
A7	16	61760933	CDH8	NM_001796.4	G	G/T	intron	none	NA	NA	
A7	5	26906966	CDH9	NM_016279.3	C	C/T	intron	none	NA	NA	
A7	1	1572825	CDK11B	NM_033486.1	A	A/G	coding-synonymous	none	420/698		1260
A7	3	138290150	CEP70	NM_024491.2	C	C/T	missense	GLY,SER	40/598		118
A7	17	62515415	CEP95	NM_138363.1	G	C/G	intron	none	NA	NA	
A7	2	169487621	CERS6	NM_001256126.1	T	A/T	intron	none	NA	NA	
A7	15	41562586	CHP1	NM_007236.4	A	A/C	intron	none	NA	NA	
A7	10	125771915	CHST15	NM_015892.3	G	A/G	missense	ARG,CYS	477/562		1429
A7	1	9794232	CLSTN1	NM_001009566.1	T	A/T	intron	none	NA	NA	
A7	3	139895021	CLSTN2	NM_022131.2	T	A/T	intron	none	NA	NA	
A7	7	147844726	CNTNAP2	NM_014141.5	C	A/C	coding-synonymous	none	900/1332		2698
A7	16	76523638	CNTNAP4	NM_033401.3	T	C/T	coding-synonymous	none	646/1309		1937
A7	7	94037360	COL1A2	NM_000089.3	T	C/T	intron	none	NA	NA	
A7	7	94040070	COL1A2	NM_000089.3	G	G/T	intron	none	NA	NA	
A7	4	109783500	COL25A1	NM_001256074.1	C	A/C	intron	none	NA	NA	
A7	13	110855901	COL4A1	NM_001845.4	A	A/G	intron	none	NA	NA	
A7	2	189904238	COL5A2	NM_000393.3	G	G/T	missense	LEU,ILE	1229/1500		3685
A7	11	36300046	COMMD9	NM_001101653.1	T	C/T	missense	LYS,GLU	58/157		172
A7	11	14515809	COPB1	NM_001144061.1	G	G/T	missense	PRO,THR	90/954		268
A7	1	207652744	CR2	NM_001006658.2	T	A/T	intron	none	NA	NA	
A7	1	197237343	CRB1	NM_001193640.1	T	C/T	utr-5	none	NA	NA	
A7	1	36945029	CSF3R	NM_000760.3	C	A/C	intron	none	NA	NA	
A7	10	68040176	CTNNA3	NM_001127384.1	C	C/T	intron	none	NA	NA	
A7	1	41466760	CTPS1	NM_001905.2	C	C/T	coding-synonymous	none	355/592		1065
A7	14	100182346	CYP46A1	NM_006668.1	G	G/T	intron	none	NA	NA	
A7	6	39867845	DAAM2	NM_001201427.1	C	A/C	intron	none	NA	NA	
A7	5	10748140	DAP	NM_004394.2	C	C/T	intron	none	NA	NA	
A7	13	36396866	DCLK1	NM_001195415.1	C	A/C	coding-synonymous-	none	211/423		633
A7	4	151142877	DCLK2	NM_001040260.3	A	A/T	missense	TYR,PHE	394/767		1181

A7	2	118582844	DDX18	NM_006773.3	G	C/G	intron	none	NA	NA	
A7	12	100657371	DEPDC4	NM_152317.2	G	A/G	missense	SER,PHE	153/295		458
A7	2	179323798	DFNB59	NM_001042702.3	G	C/G	intron	none	NA	NA	
A7	17	57663614	DHX40	NM_001166301.1	A	A/G	intron	none	NA	NA	
A7	3	52356673	DNAH1	NM_015512.4	C	C/T	missense	SER,LEU	72/4266		215
A7	7	21604085	DNAH11	NM_003777.3	G	A/G	intron	none	NA	NA	
A7	5	13729702	DNAH5	NM_001369.2	C	C/T	intron	none	NA	NA	
A7	17	72310405	DNAI2	NM_001172810.1	C	A/C	utr-3	none	NA	NA	
A7	7	157159330	DNAJB6	NM_005494.2	T	C/T	intron	none	NA	NA	
A7	10	98092087	DNTT	NM_001017520.1	T	C/T	intron	none	NA	NA	
A7	2	225669889	DOCK10	NM_014689.2	C	A/C	intron	none	NA	NA	
A7	3	109052904	DPPA4	NM_018189.3	C	C/T	intron	none	NA	NA	
A7	21	42006321	DSCAM	NM_001389.3	C	A/C	intron	none	NA	NA	
A7	11	103349846	DYNC2H1	NM_001080463.1	G	G/T	coding-synonymous	none	4270/4315		12810
A7	5	158204489	EBF1	NM_024007.3	A	A/G	missense	VAL,ALA	323/592		968
A7	3	129137029	EFCAB12	NM_207307.1	T	G/T	intron	none	NA	NA	
A7	17	45412698	EFCAB13	NM_001195192.1	G	G/T	missense	SER,ILE	56/785		167
A7	4	110861976	EGF	NM_001178130.1	T	C/T	intron	none	NA	NA	
A7	10	120824928	EIF3A	NM_003750.2	C	C/T	missense	GLY,SER	369/1383		1105
A7	1	79403964	ELTD1	NM_022159.3	T	A/T	missense-near-splice	ILE,PHE	133/691		397
A7	22	41574780	EP300	NM_001429.3	G	G/T	missense	GLN,HIS	2355/2415		7065
A7	4	66242824	EPHA5	NM_004439.5	A	A/G	intron	none	NA	NA	
A7	4	66509024	EPHA5	NM_004439.5	G	G/T	intron	none	NA	NA	
A7	3	134911584	EPHB1	NM_004441.4	G	G/T	coding-synonymous	none	683/985		2049
A7	17	19235029	EPN2	NM_001102664.1	A	A/T	intron	none	NA	NA	
A7	10	101911970	ERLIN1	NM_001100626.1	C	A/C	missense	GLY,VAL	322/349		965
A7	16	23706023	ERN2	NM_033266.3	C	A/C	intron	none	NA	NA	
A7	12	53664351	ESPL1	NM_012291.4	C	C/T	intron	none	NA	NA	
A7	11	108384286	EXPH5	NM_015065.2	T	A/T	missense	THR,SER	650/1990		1948
A7	2	225265944	FAM124B	NM_001122779.1	C	A/C	missense	SER,ILE	181/456		542
A7	15	99024096	FAM169B	NM_182562.2	G	A/G	intron	none	NA	NA	
A7	1	179023787	FAM20B	NM_014864.3	A	A/G	intron	none	NA	NA	
A7	9	99701411	FAM22G	NM_001045477.2	A	A/C	intron	none	NA	NA	
A7	16	67578682	FAM65A	NM_001193522.1	G	A/G	missense	VAL,ILE	944/1224		2830
A7	9	84560187	FAM75D3	NM_207416.2	T	C/T	intron	none	NA	NA	
A7	6	99322319	FBXL4	NM_012160.3	T	C/T	splice-3	none	NA	NA	
A7	1	149763115	FCGR1A	NM_000566.3	C	C/T	utr-3	none	NA	NA	
A7	1	159778653	FCRL6	NM_001004310.2	G	A/G	intron	none	NA	NA	
A7	12	95535254	FGD6	NM_018351.3	C	C/T	missense	ARG,GLN	916/1431		2747
A7	7	96114076	FLJ42280	NM_001201450.1	A	A/T	intron	none	NA	NA	
A7	7	128480826	FLNC	NM_001127487.1	T	G/T	intron	none	NA	NA	
A7	7	128493764	FLNC	NM_001127487.1	T	G/T	intron	none	NA	NA	
A7	20	14306457	FLRT3	NM_013281.3	T	A/T	missense	SER,CYS	566/650		1696

A7	1	240457955	FMN2	NM_020066.4	C	A/C	intron	none	NA	NA	
A7	14	29237222	FOXG1	NM_005249.4	A	A/T	missense	TYR,PHE	246/490		737
A7	10	13708225	FRMD4A	NM_018027.3	A	A/C	missense	LEU,ARG	492/1040		1475
A7	9	37724151	FRMPD1	NM_014907.2	A	A/T	intron	none	NA	NA	
A7	22	17472938	GAB4	NM_001037814.1	C	A/C	missense	GLN,HIS	101/575		303
A7	21	27134677	GABPA	NM_001197297.1	G	G/T	splice-5	none	NA	NA	
A7	5	161530900	GABRG2	NM_000816.3	T	A/T	missense	TYR,ASN	213/468		637
A7	5	161580054	GABRG2	NM_000816.3	C	C/G	intron	none	NA	NA	
A7	14	88411988	GALC	NM_000153.3	G	A/G	missense	HIS,TYR	527/686		1579
A7	7	30673525	GARS	NM_002047.2	A	A/C	utr-3	none	NA	NA	
A7	16	90106753	GAS8	NM_001481.2	A	A/T	missense	ILE,PHE	353/479		1057
A7	21	34132159	GCFC1	NM_013329.3	G	G/T	coding-synonymous	none	374/816		1122
A7	7	44189558	GCK	NM_000162.3	G	G/T	intron	none	NA	NA	
A7	5	74028873	GFM2	NM_032380.3	C	A/C	missense	VAL,LEU	521/780		1561
A7	22	25023556	GGT1	NM_001032364.2	C	C/T	missense	ALA,VAL	393/570		1178
A7	3	172165356	GHSR	NM_004122.2	A	A/T	missense	LEU,GLN	283/290		848
A7	12	110385176	GIT2	NM_001135213.1	C	C/T	missense	GLY,ASP	461/682		1382
A7	11	134183972	GLB1L3	NM_001080407.2	G	C/G	intron	none	NA	NA	
A7	11	134184257	GLB1L3	NM_001080407.2	C	C/G	missense	ASP,GLU	567/654		1701
A7	9	36148586	GLIPR2	NM_022343.2	C	C/G	missense	SER,ARG	55/155		165
A7	1	155733245	GON4L	NM_001037533.1	C	C/T	coding-synonymous	none	1528/2241		4584
A7	3	100365398	GPR128	NM_032787.2	C	C/T	intron	none	NA	NA	
A7	10	25886824	GPR158	NM_020752.2	C	C/T	missense	ARG,CYS	757/1216		2269
A7	6	110300638	GPR6	NM_005284.3	T	A/T	missense	PHE,TYR	108/363		323
A7	5	90073714	GPR98	NM_032119.3	T	C/T	intron	none	NA	NA	
A7	10	87487608	GRID1	NM_017551.2	C	A/C	intron	none	NA	NA	
A7	4	145032396	GYPA	NM_002099.6	C	C/T	utr-3	none	NA	NA	
A7	18	44641547	HDHD2	NM_032124.4	G	A/G	missense	ARG,TRP	190/260		568
A7	20	43056881	HNF4A	NM_000457.4	C	C/T	intron	none	NA	NA	
A7	14	21679831	HNRNPC	NM_001077442.1	C	A/C	intron	none	NA	NA	
A7	10	100185528	HPS1	NM_000195.3	C	A/C	intron	none	NA	NA	
A7	22	26854383	HPS4	NM_022081.4	C	C/T	intron	none	NA	NA	
A7	4	88278487	HSD17B11	NM_016245.3	T	A/T	coding-synonymous	none	213/301		639
A7	11	122928501	HSPA8	NM_006597.4	C	C/G	missense	GLY,ARG	628/647		1882
A7	1	22211837	HSPG2	NM_005529.5	C	A/C	missense	ASP,TYR	396/4392		1186
A7	9	21304892	IFNA5	NM_002169.2	A	A/T	missense	CYS,SER	122/190		364
A7	7	23381617	IGF2BP3	NM_006547.2	C	C/T	intron	none	NA	NA	
A7	15	81517993	IL16	NM_001172128.1	C	C/G	missense	GLN,GLU	85/1332		253
A7	19	14157335	IL27RA	NM_004843.3	T	A/T	missense	LEU,GLN	349/637		1046
A7	10	6061832	IL2RA	NM_000417.2	C	C/T	splice-5	none	NA	NA	
A7	6	76657138	IMPG1	NM_001563.2	G	G/T	missense	PRO,GLN	646/798		1937
A7	7	41739822	INHBA	NM_002192.2	G	A/G	stop-gained	GLN,stop	51/427		151
A7	1	156815072	INSRR	NM_001007792.1	G	G/T	intron	none	NA	NA	

A7	19	50162883	IRF3	NM_001197122.1	G	A/G	missense	ALA,VAL	441/453		1322
A7	5	52221380	ITGA1	NM_181501.1	G	G/T	intron	none	NA	NA	
A7	12	56096879	ITGA7	NM_001144996.1	C	C/T	missense	SER,ASN	97/1142		290
A7	10	15628607	ITGA8	NM_003638.1	G	A/G	missense	ALA,VAL	783/1064		2348
A7	10	7762891	ITIH2	NM_002216.2	G	G/T	missense	ASP,TYR	235/947		703
A7	1	227920358	JMJD4	NM_001161465.1	G	A/G	missense	SER,LEU	360/448		1079
A7	3	124196034	KALRN	NM_001024660.3	G	A/G	intron	none	NA	NA	
A7	17	73468206	KIAA0195	NM_014738.4	G	C/G	intron	none	NA	NA	
A7	17	73485526	KIAA0195	NM_014738.4	A	A/T	intron	none	NA	NA	
A7	2	241697805	KIF1A	NM_001244008.1	G	A/G	missense	ARG,CYS	852/1792		2554
A7	12	39751272	KIF21A	NM_001173463.1	A	A/G	intron	none	NA	NA	
A7	6	168439234	KIF25	NM_005355.3	C	A/C	missense-near-splice	LEU,ILE	107/333		319
A7	5	61648394	KIF2A	NM_001098511.2	A	A/T	intron	none	NA	NA	
A7	17	51902094	KIF2B	NM_032559.4	G	G/T	missense	SER,ILE	567/674		1700
A7	1	173726052	KLHL20	NM_014458.3	A	A/T	intron	none	NA	NA	
A7	20	60901962	LAMA5	NM_005560.3	C	C/T	missense	VAL,ILE	1725/3696		5173
A7	3	49159703	LAMB2	NM_002292.3	C	A/C	coding-synonymous	none	1558/1799		4674
A7	20	36979233	LBP	NM_004139.3	G	A/G	missense	GLU,LYS	87/482		259
A7	1	225607375	LBR	NM_002296.3	G	A/G	intron	none	NA	NA	
A7	9	138557645	LCN9	NM_001001676.1	G	A/G	intron	none	NA	NA	
A7	10	88445370	LDB3	NM_001080114.1	C	C/T	intron	none	NA	NA	
A7	9	126794807	LHX2	NM_004789.3	G	G/T	missense	GLY,CYS	348/407		1042
A7	19	55147165	LILRB1	NM_001081637.1	T	A/T	intron	none	NA	NA	
A7	19	54778822	LILRB2	NM_001080978.2	G	A/G	intron	none	NA	NA	
A7	1	99771279	LPPR4	NM_001166252.1	G	G/T	missense	GLU,ASP	277/706		831
A7	8	86041518	LRRCC1	NM_033402.4	A	A/T	coding-synonymous	none	510/1033		1530
A7	3	37125125	LRRFIP2	NM_001134369.1	A	A/G	splice-5	none	NA	NA	
A7	3	115560616	LSAMP	NM_002338.3	G	G/T	intron	none	NA	NA	
A7	18	32558465	MAPRE2	NM_001143826.2	G	A/G	intron	none	NA	NA	
A7	6	90437459	MDN1	NM_014611.1	A	A/G	intron	none	NA	NA	
A7	2	103347515	MFSD9	NM_032718.3	C	A/C	missense	GLY,VAL	89/475		266
A7	6	168299013	MLLT4	NM_001040000.2	G	G/T	missense	MET,ILE	482/1652		1446
A7	1	228295745	MRPL55	NM_181441.2	C	C/T	intron	none	NA	NA	
A7	2	105665841	MRPS9	NM_182640.2	G	G/T	intron	none	NA	NA	
A7	11	60476233	MS4A8B	NM_031457.1	T	C/T	coding-synonymous	none	171/251		513
A7	1	76280759	MSH4	NM_002440.3	A	A/G	coding-synonymous	none	251/937		753
A7	8	16001219	MSR1	NM_002445.3	G	G/T	intron	none	NA	NA	
A7	4	187454942	MTNR1A	NM_005958.3	C	A/C	coding-synonymous	none	318/351		954
A7	1	237052455	MTR	NM_000254.2	T	C/T	intron	none	NA	NA	
A7	7	100646966	MUC12	NM_001164462.1	A	A/C	coding-synonymous	none	4374/5336		13122
A7	19	9033233	MUC16	NM_024690.2	G	C/G	coding-synonymous	none	12131/14508		36393
A7	3	195505829	MUC4	NM_004532.5	G	A/G	intron	none	NA	NA	
A7	3	195509748	MUC4	NM_004532.5	G	C/G	intron	none	NA	NA	

A7	16	15797951	MYH11	NM_001040113.1	G	A/G	utr-3	none	NA	NA	
A7	13	109445989	MYO16	NM_001198950.1	G	G/T	splice-5	none	NA	NA	
A7	17	34866721	MYO19	NM_001163735.1	G	A/G	missense	PRO,SER	341/971		1021
A7	12	57431672	MYO1A	NM_001256041.1	G	A/G	missense	HIS,TYR	648/1044		1942
A7	17	31082576	MYO1D	NM_015194.1	G	A/G	coding-synonymous	none	467/1007		1401
A7	12	78334178	NAV3	NM_014903.4	C	C/T	missense	ALA,VAL	108/2364		323
A7	12	78452891	NAV3	NM_014903.4	G	A/G	missense	ASP,ASN	878/2364		2632
A7	13	35630062	NBEA	NM_015678.4	G	C/G	intron	none	NA	NA	
A7	8	90982627	NBN	NM_002485.4	C	C/T	coding-synonymous	none	287/755		861
A7	1	16918239	NBPF1	NM_017940.3	C	A/C	intron	none	NA	NA	
A7	21	22839020	NCAM2	NM_004540.3	T	C/T	missense	ILE,THR	583/838		1748
A7	4	121957787	NDNF	NM_024574.3	G	G/T	missense	LEU,ILE	447/569		1339
A7	4	115898364	NDST4	NM_022569.1	A	A/C	missense	SER,ALA	349/873		1045
A7	10	21076295	NEBL	NM_001173484.1	A	A/G	intron	none	NA	NA	
A7	16	84014773	NECAB2	NM_019065.2	T	G/T	intron	none	NA	NA	
A7	3	27355770	NEK10	NM_199347.2	C	C/T	intron	none	NA	NA	
A7	13	39618366	NHLRC3	NM_001012754.2	T	G/T	intron	none	NA	NA	
A7	5	43615864	NNT	NM_012343.3	T	C/T	intron	none	NA	NA	
A7	1	247901936	none	none	T	A/T	intergenic	none	NA	NA	
A7	2	89521169	none	none	C	A/C	intergenic	none	NA	NA	
A7	2	90044421	none	none	G	A/G	intergenic	none	NA	NA	
A7	3	97753725	none	none	G	G/T	intergenic	none	NA	NA	
A7	3	98072604	none	none	T	G/T	near-gene-5	none	NA	NA	
A7	4	117221005	none	none	G	G/T	intergenic	none	NA	NA	
A7	6	27782060	none	none	T	C/T	near-gene-3	none	NA	NA	
A7	7	89545844	none	none	G	G/T	intergenic	none	NA	NA	
A7	9	40715797	none	none	A	A/G	intergenic	none	NA	NA	
A7	12	38364157	none	none	G	C/G	intergenic	none	NA	NA	
A7	12	100583778	none	none	G	A/G	intergenic	none	NA	NA	
A7	14	22265970	none	none	G	A/G	intergenic	none	NA	NA	
A7	14	106354420	none	none	C	C/T	intergenic	none	NA	NA	
A7	15	20644185	none	none	T	C/T	intergenic	none	NA	NA	
A7	16	820069	none	none	G	A/G	near-gene-3	none	NA	NA	
A7	17	27188383	none	none	G	A/G	near-gene-3	none	NA	NA	
A7	17	39405881	none	none	G	A/G	near-gene-5	none	NA	NA	
A7	19	15197828	none	none	C	C/T	near-gene-5	none	NA	NA	
A7	20	26054674	none	none	G	A/G	intergenic	none	NA	NA	
A7	21	16525113	none	none	C	C/T	intergenic	none	NA	NA	
A7	21	33765599	none	none	C	C/T	near-gene-5	none	NA	NA	
A7	22	21832202	none	none	G	A/G	intergenic	none	NA	NA	
A7	6	108492739	NR2E1	NM_003269.3	G	G/T	missense	GLY,CYS	35/386		103
A7	10	84498302	NRG3	NM_001010848.3	C	A/C	intron	none	NA	NA	
A7	5	162881137	NUDCD2	NM_145266.4	G	C/G	intron	none	NA	NA	

A7	9	131750079	NUP188	NM_015354.1	A	A/G	intron	none	NA	NA	
A7	4	71063701	ODAM	NM_017855.3	C	C/G	missense	GLN,GLU	68/280		202
A7	11	132306138	OPCML	NM_001012393.1	G	G/T	intron	none	NA	NA	
A7	11	55735245	OR10AG1	NM_001005491.1	C	A/C	missense	CYS,PHE	232/302		695
A7	9	107457569	OR13D1	NM_001004484.1	T	A/T	missense	PHE,LEU	289/347		867
A7	9	35957264	OR2S2	NM_019897.2	C	C/T	missense	ASP,ASN	278/320		832
A7	11	51516094	OR4C46	NM_001004703.1	A	A/G	missense	ILE,MET	271/310		813
A7	14	20528225	OR4L1	NM_001004717.1	C	A/C	missense	LEU,ILE	8/313		22
A7	11	4843044	OR51F2	NM_001004753.1	A	A/T	coding-synonymous	none	143/343		429
A7	11	55588014	OR5D18	NM_001001952.1	C	A/C	coding-synonymous	none	303/314		909
A7	11	55873332	OR8H2	NM_001005200.1	C	C/T	stop-gained	GLN,stop	272/313		814
A7	14	23235855	OXA1L	NM_005015.3	C	C/G	missense	SER,TRP	42/496		125
A7	13	25671451	PABPC3	NM_030979.2	A	A/G	missense	GLU,GLY	372/632		1115
A7	11	35454112	PAMR1	NM_001001991.1	C	A/C	missense	TRP,LEU	652/721		1955
A7	1	176564516	PAPPA2	NM_020318.2	C	A/C	coding-synonymous	none	592/1792		1776
A7	10	51040935	PARG	NM_003631.2	C	A/C	missense	ARG,LEU	854/977		2561
A7	10	51087953	PARG	NM_003631.2	T	C/T	intron	none	NA	NA	
A7	11	125647619	PATE2	NM_212555.2	G	A/G	intron	none	NA	NA	
A7	1	164528980	PBX1	NM_001204961.1	G	A/G	utr-5	none	NA	NA	
A7	5	140573844	PCDHB10	NM_018930.3	C	C/T	coding-synonymous	none	573/801		1719
A7	5	140590211	PCDHB12	NM_018932.3	T	C/T	missense	TRP,ARG	578/796		1732
A7	7	82580093	PCLO	NM_014510.2	G	C/G	missense	GLN,GLU	3271/4936		9811
A7	1	144994494	PDE4DIP	NM_001002810.3	G	A/G	intron	none	NA	NA	
A7	12	9085124	PHC1	NM_004426.2	T	C/T	intron	none	NA	NA	
A7	20	34459748	PHF20	NM_016436.4	G	G/T	stop-gained	GLU,stop	427/1013		1279
A7	6	79692501	PHIP	NM_017934.5	G	C/G	intron	none	NA	NA	
A7	3	111637861	PHLDB2	NM_001134437.1	G	C/G	intron	none	NA	NA	
A7	10	98412553	PIK3AP1	NM_152309.2	C	C/G	missense	ARG,THR	205/806		614
A7	9	71395459	PIP5K1B	NM_003558.2	G	A/G	intron	none	NA	NA	
A7	9	26935252	PLAA	NM_001031689.2	G	A/G	intron	none	NA	NA	
A7	11	64024066	PLCB3	NM_000932.2	C	C/T	intron	none	NA	NA	
A7	20	9389588	PLCB4	NM_000933.3	C	A/C	intron	none	NA	NA	
A7	2	198949586	PLCL1	NM_006226.3	G	A/G	missense	GLY,SER	449/1096		1345
A7	5	41313660	PLCXD3	NM_001005473.2	G	C/G	utr-3	none	NA	NA	
A7	19	39914884	PLEKHG2	NM_022835.2	T	A/T	coding-synonymous	none	1037/1387		3111
A7	21	46698099	POFUT2	NM_015227.4	C	C/T	intron	none	NA	NA	
A7	19	1095232	POLR2E	NM_002695.3	C	A/C	intron	none	NA	NA	
A7	2	132021766	POTEE	NM_001083538.1	A	A/C	missense	LYS,THR	913/1076		2738
A7	22	16275415	POTEH	NM_001136213.1	G	A/G	intron	none	NA	NA	
A7	7	31735232	PPP1R17	NM_001145123.2	C	C/T	intron	none	NA	NA	
A7	7	94832890	PPP1R9A	NM_001166160.1	G	C/G	intron	none	NA	NA	
A7	11	45241012	PRDM11	NM_001256695.1	G	G/T	intron	none	NA	NA	
A7	5	122491588	PRDM6	NM_001136239.1	A	A/G	missense	GLN,ARG	304/596		911

A7	7	151476274	PRKAG2	NM_001040633.1	T	C/T	intron	none	NA	NA	
A7	7	151552258	PRKAG2	NM_016203.3	A	A/G	intron	none	NA	NA	
A7	1	2116704	PRKCZ	NM_001033581.1	G	A/G	utr-3	none	NA	NA	
A7	6	22292680	PRL	NM_000948.5	C	A/C	intron	none	NA	NA	
A7	12	50026791	PRPF40B	NM_001031698.2	A	A/C	intron	none	NA	NA	
A7	7	142460445	PRSS1	NM_002769.4	G	A/G	intron	none	NA	NA	
A7	19	43579894	PSG2	NM_031246.3	A	A/G	intron	none	NA	NA	
A7	20	48132883	PTGIS	NM_000961.3	T	G/T	intron	none	NA	NA	
A7	2	131117262	PTPN18	NM_001142370.1	A	A/C	intron	none	NA	NA	
A7	1	198608329	PTPRC	NM_001267798.1	A	A/T	intron	none	NA	NA	
A7	1	198687445	PTPRC	NM_002838.4	C	C/G	intron	none	NA	NA	
A7	18	8387240	PTPRM	NM_001105244.1	G	C/G	intron	none	NA	NA	
A7	12	81013020	PTPRQ	NM_001145026.1	C	A/C	intron	none	NA	NA	
A7	1	31423058	PUM1	NM_001020658.1	G	G/T	missense	HIS,GLN	924/1189		2772
A7	2	1658095	PXDN	NM_012293.1	T	C/T	intron	none	NA	NA	
A7	12	9312660	PZP	NM_002864.2	A	A/T	intron	none	NA	NA	
A7	6	163991856	QKI	NM_006775.2	G	C/G	utr-3	none	NA	NA	
A7	5	86627363	RASA1	NM_002890.2	G	A/G	intron	none	NA	NA	
A7	5	179555400	RASGEF1C	NM_175062.3	G	G/T	intron	none	NA	NA	
A7	6	46293308	RCAN2	NM_001251973.1	C	C/T	intron	none	NA	NA	
A7	11	66613520	RCE1	NM_001032279.1	T	A/T	missense	VAL,GLU	211/226		632
A7	12	107144576	RFX4	NM_001206691.1	G	G/T	intron	none	NA	NA	
A7	1	182640643	RGS8	NM_001102450.1	G	G/T	intron	none	NA	NA	
A7	6	72957672	RIMS1	NM_001168407.1	A	A/G	intron	none	NA	NA	
A7	9	77275434	RORB	NM_006914.3	G	A/G	intron	none	NA	NA	
A7	17	27047136	RPL23A	NM_000984.5	A	A/T	intron	none	NA	NA	
A7	11	126073305	RPUSD4	NM_001144827.1	C	A/C	utr-3	none	NA	NA	
A7	14	101347944	RTL1	NM_001134888.2	T	C/T	missense	ASN,SER	1061/1359		3182
A7	2	55200193	RTN4	NM_007008.2	C	C/T	utr-3	none	NA	NA	
A7	9	137323789	RXRA	NM_002957.4	A	A/G	missense	GLN,ARG	361/463		1082
A7	3	169642926	SAMD7	NM_182610.2	T	C/T	splice-5	none	NA	NA	
A7	22	44372601	SAMM50	NM_015380.4	G	C/G	intron	none	NA	NA	
A7	6	148840883	SASH1	NM_015278.3	A	A/G	missense	SER,GLY	355/1248		1063
A7	5	147258391	SCGB3A2	NM_054023.4	C	A/C	missense	LEU,MET	9/94		25
A7	5	79446669	SERINC5	NM_001174071.1	G	A/G	intron	none	NA	NA	
A7	2	73215302	SFXN5	NM_144579.2	G	A/G	intron	none	NA	NA	
A7	11	70507629	SHANK2	NM_012309.3	C	A/C	intron	none	NA	NA	
A7	4	42402862	SHISA3	NM_001080505.1	T	A/T	intron	none	NA	NA	
A7	3	164755590	SI	NM_001041.3	G	C/G	intron	none	NA	NA	
A7	20	3682110	SIGLEC1	NM_023068.3	A	A/C	coding-synonymous	none	469/1710		1407
A7	14	72117114	SIPA1L1	NM_015556.1	G	C/G	missense	GLU,ASP	627/1805		1881
A7	11	22364979	SLC17A6	NM_020346.2	T	G/T	intron	none	NA	NA	
A7	11	22399033	SLC17A6	NM_020346.2	C	C/T	missense	SER,LEU	499/583		1496

A7	20	19496302	SLC24A3	NM_020689.3	C	A/C	intron	none	NA	NA	
A7	5	128302154	SLC27A6	NM_001017372.1	G	G/T	coding-synonymous	none	108/620		324
A7	12	8085698	SLC2A3	NM_006931.2	C	C/T	missense	ALA,THR	52/497		154
A7	17	18874630	SLC5A10	NM_001039999.2	G	A/G	utr-3	none	NA	NA	
A7	19	47951332	SLC8A2	NM_015063.2	G	C/G	coding-synonymous	none	499/922		1497
A7	3	111988851	SLC9C1	NM_183061.1	C	A/C	coding-synonymous	none	229/1178		687
A7	12	21344488	SLCO1B1	NM_006446.4	A	A/G	intron	none	NA	NA	
A7	12	21008055	SLCO1B3	NM_019844.3	G	C/G	missense	ASP,HIS	60/703		178
A7	8	70585268	SLCO5A1	NM_001146008.1	A	A/T	utr-3	none	NA	NA	
A7	4	144468474	SMARCA5	NM_003601.3	C	A/C	intron	none	NA	NA	
A7	4	95196039	SMARCAD1	NM_001128429.2	A	A/G	splice-3	none	NA	NA	
A7	2	96944014	SNRNP200	NM_014014.4	G	A/G	coding-synonymous	none	1857/2137		5571
A7	2	96964838	SNRNP200	NM_014014.4	T	A/T	intron	none	NA	NA	
A7	7	17836600	SNX13	NM_015132.4	C	C/T	intron	none	NA	NA	
A7	2	32362085	SPAST	NM_014946.3	T	A/T	intron	none	NA	NA	
A7	1	16730342	SPATA21	NM_198546.1	G	A/G	intron	none	NA	NA	
A7	14	65246458	SPTB	NM_000347.5	G	A/G	coding-synonymous	none	1486/2138		4458
A7	2	54876364	SPTBN1	NM_003128.2	G	C/G	intron	none	NA	NA	
A7	6	35837513	SRPK1	NM_003137.4	C	C/T	missense	CYS,TYR	386/656		1157
A7	15	42983522	STARD9	NM_020759.2	G	G/T	missense	ARG,ILE	3249/4701		9746
A7	9	130438029	STXBP1	NM_001032221.3	G	A/G	intron	none	NA	NA	
A7	3	120876230	STXBP5L	NM_014980.2	T	C/T	intron	none	NA	NA	
A7	20	31589197	SUN5	NM_080675.3	C	C/G	intron	none	NA	NA	
A7	1	223438090	SUSD4	NM_001037175.2	C	A/C	coding-synonymous	none	202/291		606
A7	17	30300321	SUZ12	NM_015355.2	A	A/G	intron	none	NA	NA	
A7	10	29760242	SVIL	NM_003174.3	C	A/C	intron	none	NA	NA	
A7	6	152784658	SYNE1	NM_033071.3	C	C/T	intron	none	NA	NA	
A7	11	85407186	SYTL2	NM_001162951.1	A	A/T	intron	none	NA	NA	
A7	6	132966835	TAAR1	NM_138327.1	T	C/T	missense	ASP,GLY	103/340		308
A7	1	150465078	TARS2	NM_025150.3	G	C/G	intron	none	NA	NA	
A7	4	26744084	TBC1D19	NM_018317.2	A	A/T	intron	none	NA	NA	
A7	15	74177192	TBC1D21	NM_153356.1	G	A/G	coding-synonymous	none	146/337		438
A7	5	145886801	TCERG1	NM_001040006.1	C	C/G	intron	none	NA	NA	
A7	12	104380725	TDG	NM_003211.4	G	A/G	splice-3	none	NA	NA	
A7	14	90437654	TDP1	NM_001008744.1	G	C/G	intron	none	NA	NA	
A7	10	114046311	TECTB	NM_058222.1	A	A/G	intron	none	NA	NA	
A7	9	103088616	TEX10	NM_001161584.1	C	C/T	coding-synonymous	none	652/914		1956
A7	2	138376186	THSD7B	NM_001080427.1	A	A/T	intron	none	NA	NA	
A7	6	155565061	TIAM2	NM_001010927.2	C	C/T	intron	none	NA	NA	
A7	1	43767003	TIE1	NM_001253357.1	A	A/T	intron	none	NA	NA	
A7	9	82242460	TLE4	NM_007005.3	C	C/G	intron	none	NA	NA	
A7	9	120476745	TLR4	NM_003266.3	G	G/T	missense	ARG,MET	740/800		2219
A7	15	83796056	TM6SF1	NM_001144903.1	C	C/G	intron	none	NA	NA	

A7	1	165723612	TMCO1	NM_001256164.1	C	A/C	intron	none	NA	NA	
A7	5	140022146	TMCO6	NM_018502.3	A	A/T	intron	none	NA	NA	
A7	14	77710611	TMEM63C	NM_020431.2	G	A/G	intron	none	NA	NA	
A7	8	119945319	TNFRSF11B	NM_002546.3	C	A/C	missense	SER,ILE	84/402		251
A7	3	170811534	TNIK	NM_001161560.1	A	A/G	intron	none	NA	NA	
A7	14	21498710	TPPP2	NM_173846.4	C	A/C	utr-5	none	NA	NA	
A7	16	1279790	TPSB2	NM_024164.5	G	C/G	intron	none	NA	NA	
A7	13	20000598	TPTE2	NM_001141968.1	C	C/G	coding-synonymous	none	343/412		1029
A7	3	36873763	TRANK1	NM_014831.2	G	A/G	coding-synonymous	none	2393/2926		7179
A7	6	123576973	TRDN	NM_006073.3	G	G/T	intron	none	NA	NA	
A7	5	64892761	TRIM23	NM_001656.3	A	A/G	intron	none	NA	NA	
A7	11	6478722	TRIM3	NM_001248006.1	C	A/C	intron	none	NA	NA	
A7	7	75028166	TRIM73	NM_198924.2	T	G/T	intron	none	NA	NA	
A7	5	14369558	TRIO	NM_007118.2	G	G/T	missense	GLY,CYS	1048/3098		3142
A7	4	8467172	TRMT44	NM_152544.2	C	C/T	missense	ARG,TRP	460/758		1378
A7	17	3436201	TRPV3	NM_001258205.1	G	A/G	missense	THR,ILE	272/792		815
A7	17	73513786	TSEN54	NM_207346.2	G	A/G	intron	none	NA	NA	
A7	5	176079952	TSPAN17	NM_001006616.2	C	C/T	intron	none	NA	NA	
A7	12	3310332	TSPAN9	NM_001168320.1	G	G/T	intron	none	NA	NA	
A7	21	46021049	TSPEAR	NM_144991.2	G	A/G	intron	none	NA	NA	
A7	11	43427542	TTC17	NM_018259.5	G	G/T	intron	none	NA	NA	
A7	4	147796026	TTC29	NM_031956.2	C	C/T	missense	GLY,GLU	214/476		641
A7	14	76184168	TTLL5	NM_015072.4	C	C/G	intron	none	NA	NA	
A7	2	179559979	TTN	NM_001256850.1	C	C/T	intron	none	NA	NA	
A7	2	179587580	TTN	NM_001256850.1	G	A/G	missense	THR,ILE	7032/34351		21095
A7	2	179594970	TTN	NM_001256850.1	G	C/G	missense	HIS,ASP	5736/34351		17206
A7	2	179647077	TTN	NM_001256850.1	G	A/G	missense	ALA,VAL	1081/34351		3242
A7	13	19751346	TUBA3C	NM_006001.2	T	G/T	coding-synonymous	none	259/451		777
A7	6	112402461	TUBE1	NM_016262.4	C	C/T	intron	none	NA	NA	
A7	15	54841919	UNC13C	NM_001080534.1	T	C/T	intron	none	NA	NA	
A7	14	94107519	UNC79	NM_020818.3	A	A/T	coding-synonymous	none	1805/2459		5415
A7	6	144875782	UTRN	NM_007124.2	T	A/T	intron	none	NA	NA	
A7	6	133079091	VNN2	NM_078488.1	T	A/T	intron	none	NA	NA	
A7	9	79955452	VPS13A	NM_001018037.1	C	C/G	missense	LEU,VAL	2299/3136		6895
A7	7	38836579	VPS41	NM_014396.3	C	A/C	intron	none	NA	NA	
A7	10	50122072	WDFY4	NM_001006939.3	G	G/T	coding-synonymous	none	43/262		129
A7	12	64838659	XPOT	NM_007235.4	G	A/G	intron	none	NA	NA	
A7	9	88958079	ZCCHC6	NM_001185059.1	C	C/G	splice-3	none	NA	NA	
A7	12	122958634	ZCCHC8	NM_017612.3	C	C/T	missense	GLU,LYS	512/708		1534
A7	5	843658	ZDHHC11	NM_024786.2	A	A/G	intron	none	NA	NA	
A7	10	99214686	ZDHHC16	NM_032327.2	G	A/G	intron	none	NA	NA	
A7	3	113677439	ZDHHC23	NM_173570.3	A	A/G	intron	none	NA	NA	
A7	10	31610250	ZEB1	NM_001128128.2	T	G/T	utr-5	none	NA	NA	

A7	14	68235136	ZFYVE26	NM_015346.3	T	C/T	intron	none	NA	NA
A7	8	124266425	ZHX1	NM_001017926.2	G	A/G	missense	LEU,PHE	588/874	1762
A7	19	58579540	ZNF135	NM_001164529.1	C	C/G	utr-3	none	NA	NA
A7	19	57723733	ZNF264	NM_003417.4	G	G/T	missense	GLY,VAL	423/628	1268
A7	5	150275192	ZNF300	NM_001172831.1	G	A/G	missense	PRO,SER	553/621	1657
A7	10	64429930	ZNF365	NM_199451.2	T	A/T	intron	none	NA	NA
A7	5	71757123	ZNF366	NM_152625.1	G	A/G	coding-synonymous	none	67/745	201
A7	18	72632410	ZNF407	NM_001146189.1	T	C/T	intron	none	NA	NA
A7	5	178373596	ZNF454	NM_001178089.1	A	A/C	intron	none	NA	NA
A7	7	57193591	ZNF479	NM_033273.1	A	A/G	intron	none	NA	NA
A7	19	37210972	ZNF567	NM_152603.2	A	A/T	missense	TYR,PHE	418/617	1253
A7	1	40954866	ZNF642	NM_198494.2	A	A/G	missense	TYR,CYS	109/527	326
A7	9	115759592	ZNF883	NM_001101338.1	T	C/T	coding-synonymous	none	316/380	948
A7	10	58119477	ZWINT	NM_001005413.1	G	A/G	missense	ARG,TRP	132/231	394
A8	19	1056019	ABCA7	NM_019112.3	C	A/C	intron	none	NA	NA
A8	10	116251751	ABLIM1	NM_001003407.1	T	C/T	intron	none	NA	NA
A8	11	34180876	ABTB2	NM_145804.2	G	A/G	coding-synonymous	none	888/1026	2664
A8	7	152513741	ACTR3B	NM_001040135.2	A	A/T	intron	none	NA	NA
A8	7	87795098	ADAM22	NM_004194.3	T	C/T	intron	none	NA	NA
A8	12	43860568	ADAMTS20	NM_025003.3	T	C/T	coding-synonymous	none	418/1911	1254
A8	15	79058317	ADAMTS7	NM_014272.3	C	C/T	coding-synonymous	none	1312/1687	3936
A8	11	67051402	ADRBK1	NM_001619.3	G	A/G	coding-synonymous	none	491/690	1473
A8	1	110560290	AHCYL1	NM_001242673.1	T	G/T	intron	none	NA	NA
A8	8	41641649	ANK1	NM_000037.3	G	A/G	intron	none	NA	NA
A8	6	90340654	ANKRD6	NM_001242809.1	G	A/G	coding-synonymous	none	705/728	2115
A8	5	141044445	ARAP3	NM_022481.5	A	A/C	intron	none	NA	NA
A8	13	52532569	ATP7B	NM_000053.3	G	C/G	missense	LEU,VAL	745/1466	2233
A8	19	41931678	B3GNT8	NM_198540.2	A	A/T	missense	TYR,ASN	336/398	1006
A8	16	88110156	BANP	NM_001173539.1	G	C/G	intron	none	NA	NA
A8	16	4649428	C16orf96	NM_001145011.1	C	C/T	intron	none	NA	NA
A8	1	65098259	CACHD1	NM_020925.2	C	C/T	intron	none	NA	NA
A8	5	26885680	CDH9	NM_016279.3	G	C/G	intron	none	NA	NA
A8	1	1639490	CDK11A	NM_024011.2	G	C/G	intron	none	NA	NA
A8	1	1573988	CDK11B	NM_033486.1	C	C/T	intron	none	NA	NA
A8	20	3766952	CENPB	NM_001810.5	C	C/T	missense	GLY,GLU	60/600	179
A8	22	29095773	CHEK2	NM_001005735.1	G	C/G	intron	none	NA	NA
A8	16	23403866	COG7	NM_153603.3	C	C/T	intron	none	NA	NA
A8	X	107782759	COL4A5	NM_000495.4	G	C/C	intron	none	NA	NA
A8	20	56098120	CTCFL	NM_001269040.1	C	C/T	intron	none	NA	NA
A8	19	41354417	CYP2A6	NM_000762.5	G	C/G	intron	none	NA	NA
A8	14	24513014	DHRS4L1	NM_001082488.1	G	A/G	intron	none	NA	NA
A8	16	20996807	DNAH3	NM_017539.1	G	C/G	missense	ILE,MET	2419/4117	7257
A8	1	98015206	DPYD	NM_000110.3	T	A/T	coding-synonymous	none	478/1026	1434

A8	7	55259515	EGFR	NM_005228.3	T	G/T	missense	LEU,ARG	858/1211		2573
A8	10	120809529	EIF3A	NM_003750.2	T	C/T	intron	none	NA	NA	
A8	9	23705056	ELAVL2	NM_001171195.1	C	C/T	missense	ARG,HIS	116/347		347
A8	8	28575429	EXTL3	NM_001440.2	C	C/T	missense	PRO,LEU	618/920		1853
A8	8	139207433	FAM135B	NM_015912.3	A	A/C	intron	none	NA	NA	
A8	1	179041158	FAM20B	NM_014864.3	C	C/G	missense	PRO,ARG	370/410		1109
A8	9	97080692	FAM22F	NM_017561.1	C	C/G	utr-3	none	NA	NA	
A8	9	97080706	FAM22F	NM_017561.1	T	C/T	utr-3	none	NA	NA	
A8	20	33875060	FAM83C	NM_178468.5	G	A/G	coding-synonymous	none	508/748		1522
A8	16	89881045	FANCA	NM_000135.2	T	G/T	intron	none	NA	NA	
A8	6	52943477	FBXO9	NM_012347.4	G	C/G	intron	none	NA	NA	
A8	18	34298093	FHOD3	NM_025135.2	C	C/T	coding-synonymous	none	769/1440		2307
A8	6	76024168	FILIP1	NM_015687.2	C	A/C	missense	LYS,ASN	460/1214		1380
A8	1	171086231	FMO3	NM_001002294.2	C	C/G	intron	none	NA	NA	
A8	11	49175368	FOLH1	NM_001014986.1	C	C/T	intron	none	NA	NA	
A8	12	89917015	GALNT4	NM_001199777.1	G	C/G	intron	none	NA	NA	
A8	6	53380946	GCLC	NM_001197115.1	A	A/T	intron	none	NA	NA	
A8	10	5808636	GDI2	NM_001115156.1	T	G/T	intron	none	NA	NA	
A8	17	38121996	GSDMA	NM_178171.4	G	A/G	missense	GLY,GLU	19/446		56
A8	22	24323303	GSTT2	NM_000854.3	G	A/G	intron	none	NA	NA	
A8	11	18373884	GTF2H1	NM_001142307.1	A	A/G	missense	HIS,ARG	384/549		1151
A8	7	74158003	GTF2I	NM_001163636.1	A	A/G	intron	none	NA	NA	
A8	5	140058715	HARS	NM_001258040.1	G	C/G	intron	none	NA	NA	
A8	12	112708285	HECTD4	NM_001109662.3	G	A/G	intron	none	NA	NA	
A8	5	149389957	HMGXB3	NM_014983.2	G	A/G	missense	GLY,ASP	199/1293		596
A8	14	23746218	HOMEZ	NM_020834.2	G	A/G	coding-synonymous	none	73/551		219
A8	1	21092032	HP1BP3	NM_016287.3	G	C/G	intron	none	NA	NA	
A8	9	21367554	IFNA13	NM_006900.3	A	A/G	coding-synonymous	none	152/191		456
A8	9	21187255	IFNA4	NM_021068.2	G	A/G	coding-synonymous	none	92/190		276
A8	18	22028183	IMPACT	NM_018439.3	C	A/C	intron	none	NA	NA	
A8	3	100947844	IMPG2	NM_016247.3	G	C/G	intron	none	NA	NA	
A8	11	9459613	IPO7	NM_006391.2	T	G/T	intron	none	NA	NA	
A8	12	113645651	IQCD	NM_138451.1	G	A/G	coding-synonymous	none	107/348		321
A8	13	46937313	KIAA0226L	NM_025113.2	T	C/T	missense	THR,ALA	288/663		862
A8	1	47154164	KIAA0494	NM_014774.2	C	A/C	missense	GLY,VAL	283/496		848
A8	17	6510135	KIAA0753	NM_014804.2	T	G/T	intron	none	NA	NA	
A8	5	61661200	KIF2A	NM_001098511.2	T	A/T	intron	none	NA	NA	
A8	12	52884509	KRT6A	NM_005554.3	G	A/G	coding-synonymous	none	307/565		921
A8	17	39305800	KRTAP4-5	NM_033188.3	T	A/T	missense	SER,CYS	74/182		220
A8	3	134343862	KY	NM_178554.4	G	A/G	intron	none	NA	NA	
A8	2	136558272	LCT	NM_002299.2	C	C/G	missense	GLY,ARG	1591/1928		4771
A8	1	160793351	LY9	NM_001261456.1	C	C/T	missense	THR,ILE	518/642		1553
A8	10	30914928	LYZL2	NM_183058.2	A	A/T	intron	none	NA	NA	

A8	6	119510801	MAN1A1	NM_005907.3	C	C/T	intron	none	NA	NA
A8	19	17845182	MAP1S	NM_018174.4	A	A/G	missense	ASN,SER	1042/1060	3125
A8	X	135307140	MAP7D3	NM_001173516.1	G	A/A	intron	none	NA	NA
A8	6	20115599	MBOAT1	NM_001080480.1	T	C/T	intron	none	NA	NA
A8	5	112437550	MCC	NM_001085377.1	C	A/C	coding-synonymous	none	428/1020	1284
A8	3	179109986	MFN1	NM_033540.2	T	C/T	utr-3	none	NA	NA
A8	3	195505836	MUC4	NM_004532.5	G	C/G	intron	none	NA	NA
A8	3	108102602	MYH15	NM_014981.1	G	A/G	intron	none	NA	NA
A8	17	10550455	MYH3	NM_002470.3	A	A/T	intron	none	NA	NA
A8	1	183556154	NCF2	NM_000433.3	G	G/T	intron	none	NA	NA
A8	5	149900766	NDST1	NM_001543.4	G	A/G	utr-5	none	NA	NA
A8	20	25470559	NINL	NM_025176.4	C	A/C	missense	ARG,SER	516/1383	1548
A8	2	89417381	none	none	C	C/T	intergenic	none	NA	NA
A8	2	89417387	none	none	T	C/T	intergenic	none	NA	NA
A8	7	142223845	none	none	T	C/T	intergenic	none	NA	NA
A8	12	9572194	none	none	G	A/G	intergenic	none	NA	NA
A8	15	20649683	none	none	C	C/T	intergenic	none	NA	NA
A8	15	22489847	none	none	C	A/C	intergenic	none	NA	NA
A8	16	2712662	none	none	C	C/T	intergenic	none	NA	NA
A8	22	21832241	none	none	C	C/T	intergenic	none	NA	NA
A8	X	153496121	OPN1MW2	NM_001048181.2	C	A/A	coding-synonymous	none	283/365	849
A8	9	125487011	OR1L4	NM_001005235.1	T	C/T	missense	VAL,ALA	248/312	743
A8	9	128691924	PBX3	NM_001134778.1	C	C/T	intron	none	NA	NA
A8	2	173435578	PDK1	NM_002610.3	C	C/T	intron	none	NA	NA
A8	4	152682084	PET112	NM_004564.2	G	G/T	missense	PHE,LEU	17/558	51
A8	16	72001074	PKD1L3	NM_181536.1	A	A/C	coding-synonymous	none	809/1733	2427
A8	1	208257762	PLXNA2	NM_025179.3	C	C/T	missense	ARG,HIS	754/1895	2261
A8	10	118394497	PNLIPRP2	NM_005396.4	G	G/T	intron	none	NA	NA
A8	10	118196297	PNLIPRP3	NM_001011709.2	T	C/T	coding-synonymous	none	42/468	124
A8	X	24760284	POLA1	NM_016937.3	C	A/A	intron	none	NA	NA
A8	7	76241245	POMZP3	NM_012230.3	A	A/T	intron	none	NA	NA
A8	18	14513537	POTEC	NM_001137671.1	C	C/T	intron	none	NA	NA
A8	7	39379604	POU6F2	NM_001166018.1	A	A/G	missense	ASN,SER	292/656	875
A8	5	149200199	PPARGC1B	NM_001172698.1	A	A/C	intron	none	NA	NA
A8	7	94827790	PPP1R9A	NM_001166160.1	T	C/T	coding-synonymous	none	628/1375	1884
A8	1	186270729	PRG4	NM_001127708.1	T	C/T	missense	SER,PRO	28/1364	82
A8	1	171535451	PRRC2C	NM_015172.3	A	A/G	missense	ASN,SER	2064/2818	6191
A8	7	142460107	PRSS1	NM_002769.4	G	A/G	intron	none	NA	NA
A8	7	142460148	PRSS1	NM_002769.4	T	C/T	intron	none	NA	NA
A8	7	142460187	PRSS1	NM_002769.4	T	A/T	intron	none	NA	NA
A8	19	799503	PTBP1	NM_002819.4	G	A/G	intron	none	NA	NA
A8	3	62216840	PTPRG	NM_002841.3	G	G/T	intron	none	NA	NA
A8	20	37186921	RALGAPB	NM_020336.2	T	C/T	intron	none	NA	NA

A8		4	74477431	RASSF6		NM_001270391.1	A	A/G	intron	none	NA	NA	
A8		20	35684675	RBL1		NM_002895.2	G	C/G	intron	none	NA	NA	
A8		15	56385791	RFX7		NM_022841.5	C	C/G	missense	ASP,HIS	1379/1461		4135
A8	X		119210882	RHOXF2B		NM_001099685.1	A	G/G	missense	CYS,ARG	151/289		451
A8		12	107236394	RIC8B		NM_018157.2	T	G/T	coding-synonymous	none	288/521		864
A8		16	67680546	RLTPR		NM_001013838.1	G	C/G	intron	none	NA	NA	
A8		7	6820386	RSPH10B2		NM_001099697.1	T	A/T	intron	none	NA	NA	
A8		19	36018407	SBSN		NM_001166034.1	T	C/T	coding-synonymous	none	259/591		777
A8		1	31351463	SDC3		NM_014654.3	T	G/T	intron	none	NA	NA	
A8		1	151337603	SELENBP1		NM_001258288.1	C	C/G	intron	none	NA	NA	
A8		1	151339022	SELENBP1		NM_001258288.1	G	C/G	intron	none	NA	NA	
A8		6	146234593	SHPRH		NM_001042683.2	T	C/T	intron	none	NA	NA	
A8		8	92406041	SLC26A7		NM_052832.2	A	A/G	missense	LYS,ARG	598/657		1793
A8		16	30208451	SLX1A		NM_001014999.2	G	C/G	intron	none	NA	NA	
A8		20	35457611	SOGA1		NM_080627.2	G	C/G	missense	SER,CYS	306/1662		917
A8		17	48632555	SPATA20		NM_001258372.1	C	C/T	intron	none	NA	NA	
A8		16	2812422	SRRM2		NM_016333.3	A	A/G	coding-synonymous	none	631/2753		1893
A8		15	74483392	STRA6		NM_001142617.1	G	G/T	intron	none	NA	NA	
A8		6	10891884	SYCP2L		NM_001040274.2	G	A/G	intron	none	NA	NA	
A8		12	102125286	SYCP3		NM_001177948.1	C	C/T	intron	none	NA	NA	
A8		17	15640998	TBC1D26		NM_178571.4	A	A/G	intron	none	NA	NA	
A8		6	13316887	TBC1D7		NM_001143964.2	C	C/T	coding-synonymous	none	145/294		435
A8		4	48169691	TEC		NM_003215.2	T	G/T	intron	none	NA	NA	
A8		11	57075772	TNKS1BP1		NM_033396.2	A	A/C	intron	none	NA	NA	
A8		13	20006716	TPTE2		NM_001141968.1	A	A/C	missense	ASN,LYS	262/412		786
A8		6	123592210	TRDN		NM_006073.3	C	C/G	intron	none	NA	NA	
A8		7	75028050	TRIM73		NM_198924.2	A	A/G	intron	none	NA	NA	
A8		8	72935085	TRPA1		NM_007332.2	T	A/T	utr-3	none	NA	NA	
A8		2	179596843	TTN		NM_001256850.1	G	A/G	missense	ALA,VAL	5301/34351		15902
A8		6	139597891	TXLNB		NM_153235.3	T	G/T	intron	none	NA	NA	
A8		4	115585134	UGT8		NM_001128174.1	C	C/G	intron	none	NA	NA	
A8		11	17515863	USH1C		NM_005709.3	C	C/T	utr-3	none	NA	NA	
A8		2	61577352	USP34		NM_014709.3	G	A/G	intron	none	NA	NA	
A8		16	8988387	USP7		NM_003470.2	G	C/G	intron	none	NA	NA	
A8		2	85804746	VAMP8		NM_003761.4	G	C/G	utr-5	none	NA	NA	
A8		3	14208718	XPC		NM_001145769.1	C	C/T	missense	ARG,GLN	154/904		461
A8		7	100348529	ZAN		NM_003386.1	G	A/G	intron	none	NA	NA	
A8		1	52769629	ZFYVE9		NM_004799.2	A	A/T	intron	none	NA	NA	
A8		10	43132305	ZNF33B		NM_006955.1	T	C/T	intron	none	NA	NA	
A8		19	57036242	ZNF471		NM_020813.2	A	A/T	missense	LYS,ILE	269/627		806
A9		4	164506969		40969	NM_001166373.1	C	A/C	missense	ASP,TYR	119/290		355
A9		22	19708262		41157	NM_001009939.2	C	C/G	intron	none	NA	NA	
A9		4	57220292	AASDH		NM_181806.2	C	C/T	coding-synonymous	none	432/1099		1296

A9	2	215976396	ABCA12	NM_173076.2	C	A/C	missense	LEU,PHE	29/2596		87
A9	1	94496425	ABCA4	NM_000350.2	A	A/G	intron	none	NA	NA	
A9	17	67246120	ABCA5	NM_018672.3	G	C/G	missense	SER,CYS	1564/1643		4691
A9	17	67109309	ABCA6	NM_080284.2	C	A/C	intron	none	NA	NA	
A9	2	169869830	ABCB11	NM_003742.2	C	C/T	stop-gained	TRP,stop	114/1322		341
A9	7	87092073	ABCB4	NM_000443.3	C	C/T	splice-5	none	NA	NA	
A9	7	20685666	ABCB5	NM_001163941.1	T	G/T	missense	PHE,VAL	296/1258		886
A9	12	40013159	ABCD2	NM_005164.3	G	G/T	missense	GLN,LYS	87/741		259
A9	18	19263519	ABHD3	NM_138340.4	G	A/G	intron	none	NA	NA	
A9	18	47318671	ACAA2	NM_006111.2	C	A/C	stop-gained	GLU,stop	202/398		604
A9	15	89416161	ACAN	NM_001135.3	G	C/G	missense	GLY,ALA	2375/2432		7124
A9	1	55081704	ACOT11	NM_015547.3	G	C/G	intron	none	NA	NA	
A9	16	20423031	ACSM5	NM_017888.2	G	C/G	intron	none	NA	NA	
A9	20	33508392	ACSS2	NM_001076552.2	C	C/T	coding-synonymous	none	354/715		1062
A9	2	74141776	ACTG2	NM_001199893.1	A	A/G	intron	none	NA	NA	
A9	1	155028183	ADAM15	NM_001261464.1	G	A/G	intron	none	NA	NA	
A9	8	39525556	ADAM18	NM_014237.2	C	A/C	missense	PRO,THR	456/740		1366
A9	16	77356401	ADAMTS18	NM_199355.2	G	A/G	intron	none	NA	NA	
A9	12	43748141	ADAMTS20	NM_025003.3	A	A/T	intron	none	NA	NA	
A9	12	43856824	ADAMTS20	NM_025003.3	G	G/T	intron	none	NA	NA	
A9	12	43944794	ADAMTS20	NM_025003.3	G	C/G	missense	SER,TRP	124/1911		371
A9	15	84657490	ADAMTSL3	NM_207517.2	G	G/T	missense	ARG,MET	1255/1692		3764
A9	1	154557896	ADAR	NM_001025107.2	C	A/C	intron	none	NA	NA	
A9	3	123010383	ADCY5	NM_001199642.1	C	C/T	intron	none	NA	NA	
A9	16	4057531	ADCY9	NM_001116.3	C	A/C	missense	GLN,HIS	574/1354		1722
A9	6	147042727	ADGB	NM_024694.3	A	A/T	intron	none	NA	NA	
A9	6	147109410	ADGB	NM_024694.3	A	A/G	intron	none	NA	NA	
A9	4	100349885	ADH7	NM_000673.4	C	C/T	intron	none	NA	NA	
A9	X	147924518	AFF2	NM_001169122.1	C	A/A	missense	THR,ASN	368/1277		1103
A9	5	132270273	AFF4	NM_014423.3	T	C/T	missense	SER,GLY	162/1164		484
A9	5	76355408	AGGF1	NM_018046.4	C	C/G	intron	none	NA	NA	
A9	1	247076758	AHCTF1	NM_015446.4	G	C/G	intron	none	NA	NA	
A9	1	77883424	AK5	NM_012093.3	C	A/C	intron	none	NA	NA	
A9	10	5033924	AKR1C2	NM_001354.5	G	A/G	intron	none	NA	NA	
A9	12	105456836	ALDH1L2	NM_001034173.3	G	G/T	intron	none	NA	NA	
A9	13	52598553	ALG11	NM_001004127.2	C	C/G	coding-synonymous	none	229/493		687
A9	2	3729222	ALLC	NM_018436.3	A	A/T	splice-3	none	NA	NA	
A9	2	73754332	ALMS1	NM_015120.4	A	A/G	intron	none	NA	NA	
A9	18	56196209	ALPK2	NM_052947.3	C	C/G	intron	none	NA	NA	
A9	11	46563784	AMBRA1	NM_001267782.1	C	C/T	missense	GLU,LYS	505/1302		1513
A9	1	115216221	AMPD1	NM_000036.2	C	C/G	intron	none	NA	NA	
A9	1	178834359	ANGPTL1	NM_004673.3	G	C/G	missense	LEU,VAL	185/492		553
A9	19	8438572	ANGPTL4	NM_001039667.1	T	G/T	intron	none	NA	NA	

A9	2	241494453	ANKMY1	NM_016552.2	C	C/G	utr-5	none	NA	NA	
A9	10	90582774	ANKRD22	NM_144590.2	T	C/T	missense-near-splice	HIS,ARG	167/192		500
A9	10	37415016	ANKRD30A	NM_052997.2	G	G/T	intron	none	NA	NA	
A9	10	37486628	ANKRD30A	NM_052997.2	T	C/T	intron	none	NA	NA	
A9	5	79854576	ANKRD34B	NM_001004441.2	C	C/G	missense	ARG,SER	421/515		1263
A9	2	97873776	ANKRD36	NM_001164315.1	A	A/T	intron	none	NA	NA	
A9	2	97875309	ANKRD36	NM_001164315.1	T	C/T	intron	none	NA	NA	
A9	7	36447349	ANLN	NM_018685.2	A	A/T	missense	THR,SER	294/1125		880
A9	11	70009661	ANO1	NM_018043.5	A	A/C	intron	none	NA	NA	
A9	11	70016960	ANO1	NM_018043.5	G	G/T	intron	none	NA	NA	
A9	11	26664676	ANO3	NM_031418.2	A	A/G	intron	none	NA	NA	
A9	12	101368840	ANO4	NM_178826.3	G	A/G	intron	none	NA	NA	
A9	11	22272208	ANO5	NM_001142649.1	T	C/T	intron	none	NA	NA	
A9	15	29346418	APBA2	NM_001130414.1	C	A/C	missense	LEU,ILE	111/738		331
A9	5	112178534	APC	NM_000038.5	G	C/G	missense	GLU,GLN	2415/2844		7243
A9	12	7802082	APOBEC1	NM_001644.3	C	A/C	utr-3	none	NA	NA	
A9	17	58556684	APPBP2	NM_006380.2	G	C/G	intron	none	NA	NA	
A9	12	105569777	APPL2	NM_001251904.1	G	C/G	intron	none	NA	NA	
A9	12	105600888	APPL2	NM_001251904.1	C	C/G	missense	ARG,THR	197/671		590
A9	15	30927712	ARHGAP11B	NM_001039841.1	G	G/T	missense	TRP,LEU	228/268		683
A9	1	156933064	ARHGEF11	NM_014784.3	C	A/C	coding-synonymous	none	317/1523		951
A9	7	144077094	ARHGEF5	NM_005435.3	A	A/G	missense	LYS,ARG	1580/1598		4739
A9	14	58785582	ARID4A	NM_002892.3	G	A/G	intron	none	NA	NA	
A9	3	49008012	ARIH2	NM_006321.2	C	C/G	intron	none	NA	NA	
A9	4	57377329	ARL9	NM_206919.1	G	A/G	intron	none	NA	NA	
A9	8	66525715	ARMC1	NM_018120.4	T	A/T	intron	none	NA	NA	
A9	3	137982972	ARMC8	NM_001267041.1	G	C/G	splice-3	none	NA	NA	
A9	1	150786503	ARNT	NM_001197325.1	C	C/G	intron	none	NA	NA	
A9	5	94936594	ARSK	NM_198150.2	T	G/T	coding-synonymous	none	380/537		1140
A9	6	101075526	ASCC3	NM_006828.2	C	A/C	missense	GLY,CYS	1528/2203		4582
A9	1	155319075	ASH1L	NM_018489.2	C	C/G	intron	none	NA	NA	
A9	3	130737380	ASTE1	NM_014065.2	C	A/C	missense	GLY,TRP	495/680		1483
A9	2	24090927	ATAD2B	NM_001242338.1	C	A/C	intron	none	NA	NA	
A9	1	1403679	ATAD3C	NM_001039211.2	T	C/T	intron	none	NA	NA	
A9	11	72533553	ATG16L2	NM_033388.1	G	A/G	coding-synonymous	none	225/620		675
A9	2	38536700	ATL2	NM_001135673.1	C	C/T	intron	none	NA	NA	
A9	15	25932757	ATP10A	NM_024490.3	C	A/C	intron	none	NA	NA	
A9	3	194147881	ATP13A3	NM_024524.3	G	C/G	missense	ILE,MET	1016/1227		3048
A9	3	193002672	ATP13A5	NM_198505.2	C	C/T	intron	none	NA	NA	
A9	1	203668933	ATP2B4	NM_001001396.2	G	A/G	intron	none	NA	NA	
A9	3	113507643	ATP6V1A	NM_001690.3	C	C/G	missense	SER,CYS	267/618		800
A9	15	50279499	ATP8B4	NM_024837.2	G	G/T	intron	none	NA	NA	
A9	12	58197142	AVIL	NM_006576.3	C	A/C	missense	ARG,LEU	617/820		1850

A9	11	117162523	BACE1	NM_001207048.1	A	A/G	missense	TYR,HIS	183/402		547
A9	15	73007718	BBS4	NM_001252678.1	A	A/G	utr-5	none	NA	NA	
A9	4	122766847	BBS7	NM_018190.3	C	C/T	missense	GLU,LYS	348/673		1042
A9	1	156617641	BCAN	NM_021948.4	C	C/T	intron	none	NA	NA	
A9	1	156621215	BCAN	NM_021948.4	C	C/T	intron	none	NA	NA	
A9	20	49493010	BCAS4	NM_001010974.1	C	C/T	intron	none	NA	NA	
A9	2	127827694	BIN1	NM_004305.3	C	A/C	intron	none	NA	NA	
A9	8	11412785	BLK	NM_001715.2	G	A/G	intron	none	NA	NA	
A9	7	34086049	BMPER	NM_133468.4	G	G/T	intron	none	NA	NA	
A9	7	2580634	BRAT1	NM_152743.3	C	C/G	missense	GLU,ASP	458/822		1374
A9	14	105683938	BRF1	NM_001242786.1	C	C/T	missense	ARG,GLN	479/585		1436
A9	X	79984359	BRWD3	NM_153252.4	C	T/T	coding-synonymous	none	426/1803		1278
A9	3	49693386	BSN	NM_003458.3	C	C/T	missense	PRO,SER	2133/3927		6397
A9	10	93702311	BTAF1	NM_003972.2	A	A/G	missense	GLN,ARG	129/1850		386
A9	6	26390430	BTN2A2	NM_001197237.1	G	C/G	missense	GLU,GLN	308/524		922
A9	10	104621710	C10orf32	NM_001136200.1	T	A/T	intron	none	NA	NA	
A9	11	122830117	C11orf63	NM_024806.2	G	G/T	missense	GLN,HIS	767/779		2301
A9	12	53694018	C12orf10	NM_021640.3	C	C/T	missense	ARG,TRP	101/377		301
A9	12	88381658	C12orf50	NM_152589.1	G	A/G	coding-synonymous	none	262/415		786
A9	14	45369895	C14orf28	NM_001017923.1	G	A/G	missense	ARG,LYS	86/311		257
A9	18	33552613	C18orf21	NM_001201474.1	G	G/T	utr-5	none	NA	NA	
A9	20	35776146	C20orf132	NM_152503.4	C	C/T	intron	none	NA	NA	
A9	20	60990638	C20orf151	NM_080833.2	T	C/T	missense	ASP,GLY	208/665		623
A9	22	38340169	C22orf23	NM_001207062.1	C	C/G	utr-3	none	NA	NA	
A9	22	42089498	C22orf46	NM_001142964.1	T	G/T	missense	MET,ARG	83/244		248
A9	2	26802106	C2orf70	NM_001105519.1	G	G/T	intron	none	NA	NA	
A9	2	209036864	C2orf80	NM_001099334.2	C	A/C	intron	none	NA	NA	
A9	3	119456144	C3orf15	NM_033364.3	A	A/G	intron	none	NA	NA	
A9	3	49311528	C3orf62	NM_198562.2	G	A/G	coding-synonymous	none	164/268		492
A9	3	49311648	C3orf62	NM_198562.2	C	A/C	intron	none	NA	NA	
A9	3	58739467	C3orf67	NM_198463.2	C	C/G	intron	none	NA	NA	
A9	3	153220073	C3orf79	NM_001101337.1	C	A/C	intron	none	NA	NA	
A9	4	100443894	C4orf17	NM_032149.2	T	A/T	intron	none	NA	NA	
A9	5	37231005	C5orf42	NM_023073.3	G	C/G	missense	ALA,GLY	362/3198		1085
A9	6	42073945	C6orf132	NM_001164446.1	C	C/T	missense	GLU,LYS	569/1189		1705
A9	6	151773640	C6orf211	NM_024573.1	G	G/T	utr-5	none	NA	NA	
A9	6	11104291	C6orf228	NM_001135575.1	C	C/T	intron	none	NA	NA	
A9	6	11104367	C6orf228	NM_001135575.1	C	C/G	intron	none	NA	NA	
A9	5	40945548	C7	NM_000587.2	C	C/G	intron	none	NA	NA	
A9	1	57372309	C8A	NM_000562.2	C	A/C	intron	none	NA	NA	
A9	8	67592062	C8orf44	NM_001204173.1	G	G/T	utr-5	none	NA	NA	
A9	5	39306954	C9	NM_001737.3	C	C/G	intron	none	NA	NA	
A9	9	72435785	C9orf135	NM_001010940.1	C	C/G	utr-5	none	NA	NA	

A9	8	86377609	CA2	NM_000067.2	C	C/G	missense	SER,CYS	48/261		143
A9	1	181719550	CACNA1E	NM_000721.3	C	A/C	intron	none	NA	NA	
A9	3	54952657	CACNA2D3	NM_018398.2	G	G/T	intron	none	NA	NA	
A9	17	37340557	CACNB1	NM_000723.4	G	C/G	intron	none	NA	NA	
A9	10	18690745	CACNB2	NM_000724.3	C	C/T	intron	none	NA	NA	
A9	3	85932431	CADM2	NM_001167674.1	C	C/G	intron	none	NA	NA	
A9	3	85984930	CADM2	NM_001167674.1	T	A/T	coding-synonymous	none	229/436		687
A9	7	122028615	CADPS2	NM_001009571.3	C	A/C	intron	none	NA	NA	
A9	3	12858762	CAND2	NM_001162499.1	C	C/G	coding-synonymous	none	777/1237		2331
A9	12	30882263	CAPRIN2	NM_001002259.1	G	C/G	intron	none	NA	NA	
A9	11	104879873	CASP5	NM_001136109.1	C	C/T	intron	none	NA	NA	
A9	21	37518208	CBR3	NM_001236.3	G	A/G	intron	none	NA	NA	
A9	17	77770838	CBX8	NM_020649.2	G	C/G	utr-5	none	NA	NA	
A9	9	15623456	CCDC171	NM_173550.2	C	C/G	intron	none	NA	NA	
A9	2	55518783	CCDC88A	NM_001135597.1	C	C/G	intron	none	NA	NA	
A9	2	118715925	CCDC93	NM_019044.4	A	A/C	intron	none	NA	NA	
A9	2	135703664	CCNT2	NM_001241.3	C	C/T	intron	none	NA	NA	
A9	10	35790508	CCNY	NM_145012.4	T	C/T	intron	none	NA	NA	
A9	2	71058688	CD207	NM_015717.3	C	C/T	intron	none	NA	NA	
A9	1	160808172	CD244	NM_001166663.1	G	A/G	intron	none	NA	NA	
A9	1	160808700	CD244	NM_001166663.1	G	C/G	intron	none	NA	NA	
A9	7	80293602	CD36	NM_000072.3	C	A/C	intron	none	NA	NA	
A9	1	207499144	CD55	NM_000574.3	C	C/T	intron	none	NA	NA	
A9	11	60777380	CD6	NM_001254750.1	G	A/G	intron	none	NA	NA	
A9	6	44371631	CDC5L	NM_001253.3	G	G/T	missense	ASP,TYR	209/803		625
A9	8	95158205	CDH17	NM_001144663.1	G	C/G	coding-synonymous	none	706/833		2118
A9	18	59166674	CDH20	NM_031891.2	G	A/G	missense	GLY,ARG	168/802		502
A9	5	176016138	CDHR2	NM_001171976.1	C	A/C	stop-gained	SER,stop	988/1311		2963
A9	12	96704829	CDK17	NM_001170464.2	C	C/T	splice-5	none	NA	NA	
A9	16	22360674	CDR2	NM_001802.1	C	C/T	coding-synonymous	none	144/455		432
A9	1	214819230	CENPF	NM_016343.3	G	A/G	missense	ARG,LYS	2106/3115		6317
A9	14	80993414	CEP128	NM_152446.3	G	A/G	intron	none	NA	NA	
A9	4	56819441	CEP135	NM_025009.4	G	A/G	missense-near-splice	GLU,LYS	102/1141		304
A9	1	180062563	CEP350	NM_014810.4	G	A/G	coding-synonymous	none	2441/3118		7323
A9	6	109471434	CEP57L1	NM_001083535.1	G	C/G	missense	GLU,GLN	152/461		454
A9	1	196695688	CFH	NM_000186.3	G	A/G	coding-synonymous	none	654/1232		1962
A9	3	126679046	CHCHD6	NM_032343.2	G	A/G	utr-3	none	NA	NA	
A9	1	111784922	CHI3L2	NM_001025197.1	A	A/G	missense	LYS,ARG	360/381		1079
A9	15	78911129	CHRNA3	NM_000743.4	G	G/T	missense	LEU,MET	71/506		211
A9	17	4805049	CHRNE	NM_000080.3	G	G/T	intron	none	NA	NA	
A9	3	142840914	CHST2	NM_004267.4	A	A/T	missense	HIS,LEU	419/531		1256
A9	16	69171830	CIRH1A	NM_032830.2	G	G/T	intron	none	NA	NA	
A9	15	43990962	CKMT1A	NM_001015001.1	G	C/G	missense	GLU,GLN	379/418		1135

A9	15	43990988	CKMT1A	NM_001015001.1	G	A/G	intron	none	NA	NA	
A9	3	33738389	CLASP2	NM_015097.2	C	C/G	missense	VAL,LEU	78/1516		232
A9	1	86965533	CLCA1	NM_001285.3	C	A/C	coding-synonymous	none	850/915		2550
A9	1	11883007	CLCN6	NM_001256959.1	C	C/T	intron	none	NA	NA	
A9	4	184241116	CLDN22	NM_001111319.1	G	A/G	coding-synonymous	none	86/221		256
A9	12	10010214	CLEC2B	NM_005127.2	C	A/C	missense	GLN,HIS	32/150		96
A9	12	7889963	CLEC4C	NM_130441.2	A	A/G	intron	none	NA	NA	
A9	1	155239416	CLK2	NM_003993.2	C	C/T	missense	ASP,ASN	88/499		262
A9	14	95690211	CLMN	NM_024734.3	G	C/G	intron	none	NA	NA	
A9	3	150645803	CLRN1	NM_001195794.1	G	G/T	coding-synonymous	none	220/246		658
A9	1	36230091	CLSPN	NM_001190481.1	A	A/T	missense	TYR,ASN	120/1276		358
A9	8	87917231	CNBD1	NM_173538.2	T	C/T	intron	none	NA	NA	
A9	8	88296805	CNBD1	NM_173538.2	C	C/G	intron	none	NA	NA	
A9	1	224918347	CNIH3	NM_152495.1	C	C/T	intron	none	NA	NA	
A9	12	41422823	CNTN1	NM_001843.3	A	A/G	intron	none	NA	NA	
A9	7	146805370	CNTNAP2	NM_014141.5	G	G/T	stop-gained	GLY,stop	228/1332		682
A9	13	40325149	COG6	NM_001145079.1	G	A/G	intron	none	NA	NA	
A9	1	103478062	COL11A1	NM_001190709.1	C	A/C	intron	none	NA	NA	
A9	1	103540027	COL11A1	NM_001190709.1	C	C/G	intron	none	NA	NA	
A9	6	75818793	COL12A1	NM_004370.5	T	A/T	missense	THR,SER	2681/3064		8041
A9	6	75822888	COL12A1	NM_004370.5	G	C/G	intron	none	NA	NA	
A9	8	121282435	COL14A1	NM_021110.1	T	C/T	intron	none	NA	NA	
A9	7	94047184	COL1A2	NM_000089.3	G	G/T	intron	none	NA	NA	
A9	8	139647372	COL22A1	NM_152888.1	T	C/T	intron	none	NA	NA	
A9	1	86282608	COL24A1	NM_152890.5	G	C/G	intron	none	NA	NA	
A9	2	189923072	COL5A2	NM_000393.3	C	C/T	intron	none	NA	NA	
A9	3	48631006	COL7A1	NM_000094.3	G	A/G	coding-synonymous	none	130/2945		390
A9	3	15540165	COLQ	NM_005677.3	C	C/G	intron	none	NA	NA	
A9	17	17171179	COPS3	NM_001199125.1	C	C/G	intron	none	NA	NA	
A9	16	57490554	COQ9	NM_020312.3	G	A/G	missense	ALA,THR	173/319		517
A9	7	120770206	CPED1	NM_001105533.1	C	C/T	stop-gained	GLN,stop	479/784		1435
A9	3	9746681	CPNE9	NM_153635.2	G	A/G	intron	none	NA	NA	
A9	2	211518956	CPS1	NM_001122633.2	G	G/T	intron	none	NA	NA	
A9	11	68525016	CPT1A	NM_001031847.2	A	A/G	intron	none	NA	NA	
A9	20	20022884	CRNKL1	NM_016652.4	G	C/G	intron	none	NA	NA	
A9	11	122720969	CRTAM	NM_019604.2	G	G/T	intron	none	NA	NA	
A9	19	48337828	CRX	NM_000554.4	C	A/C	intron	none	NA	NA	
A9	21	35003772	CRYZL1	NM_145858.2	G	A/G	intron	none	NA	NA	
A9	1	34003122	CSMD2	NM_052896.3	C	A/C	missense	CYS,PHE	3096/3488		9287
A9	8	113402831	CSMD3	NM_052900.2	C	C/T	intron	none	NA	NA	
A9	8	113403106	CSMD3	NM_052900.2	A	A/G	intron	none	NA	NA	
A9	8	113504709	CSMD3	NM_052900.2	G	A/G	intron	none	NA	NA	
A9	8	68091968	CSPP1	NM_024790.6	C	C/G	intron	none	NA	NA	

A9		20	56090655	CTCFL	NM_001269040.1	G	G/T	intron	none	NA	NA	
A9		2	204737466	CTLA4	NM_001037631.2	T	G/T	missense	CYS,GLY	165/175		493
A9		11	10789670	CTR9	NM_014633.3	G	A/G	missense	ARG,LYS	641/1174		1922
A9		10	17130420	CUBN	NM_001081.3	G	A/G	intron	none	NA	NA	
A9		10	35321515	CUL2	NM_001198777.1	A	A/C	intron	none	NA	NA	
A9		5	134914208	CXCL14	NM_004887.4	C	C/T	missense	ARG,GLN	41/112		122
A9		3	45988374	CXCR6	NM_006564.1	C	C/T	missense	ALA,VAL	134/343		401
A9	X		35938069	CXorf22	NM_152632.3	C	A/A	missense	ASP,GLU	51/977		153
A9	X		125954675	CXorf64	NM_001122716.1	A	T/T	coding-synonymous	none	18/299		54
A9		12	109286631	DAO	NM_001917.4	C	C/G	intron	none	NA	NA	
A9		7	44097552	DBNL	NM_001014436.2	C	C/G	intron	none	NA	NA	
A9		7	44100346	DBNL	NM_001014436.2	G	G/T	intron	none	NA	NA	
A9		4	155155673	DCHS2	NM_017639.3	G	G/T	utr-3	none	NA	NA	
A9		11	108550195	DDX10	NM_004398.2	C	C/T	coding-synonymous	none	252/876		756
A9		12	31249342	DDX11	NM_001257144.1	C	C/G	intron	none	NA	NA	
A9		4	169158893	DDX60	NM_017631.5	C	A/C	missense	MET,ILE	1406/1713		4218
A9		4	169183798	DDX60	NM_017631.5	C	A/C	intron	none	NA	NA	
A9		8	91055088	DECR1	NM_001359.1	G	G/T	intron	none	NA	NA	
A9		6	35280129	DEF6	NM_022047.3	C	C/T	coding-synonymous	none	158/632		474
A9		16	90030666	DEF8	NM_001242816.1	C	C/T	missense	SER,LEU	354/442		1061
A9		1	153904887	DENND4B	NM_014856.2	T	A/T	coding-synonymous	none	1263/1497		3789
A9	X		50127571	DGKK	NM_001013742.2	T	C/C	intron	none	NA	NA	
A9		2	39070165	DHX57	NM_198963.1	G	A/G	missense	LEU,PHE	803/1387		2407
A9		13	60558045	DIAPH3	NM_001042517.1	T	C/T	intron	none	NA	NA	
A9		13	60558071	DIAPH3	NM_001042517.1	T	A/T	intron	none	NA	NA	
A9		12	51121592	DIP2B	NM_173602.2	G	A/G	coding-synonymous	none	1169/1577		3507
A9		10	403770	DIP2C	NM_014974.2	T	G/T	missense	LYS,GLN	968/1557		2902
A9		2	233113939	DIS3L2	NM_001257281.1	C	C/G	intron	none	NA	NA	
A9		3	38101884	DLEC1	NM_007335.2	G	C/G	intron	none	NA	NA	
A9		6	170592438	DLL1	NM_005618.3	G	A/G	coding-synonymous	none	643/724		1929
A9		2	172967059	DLX2	NM_004405.3	G	C/G	missense	GLN,GLU	70/329		208
A9		10	70209896	DNA2	NM_001080449.2	T	C/T	missense	ILE,MET	276/1061		828
A9		3	52366237	DNAH1	NM_015512.4	C	C/T	coding-synonymous	none	371/4266		1113
A9		17	76506408	DNAH17	NM_173628.3	A	A/C	intron	none	NA	NA	
A9		16	20981223	DNAH3	NM_017539.1	C	C/T	coding-synonymous	none	2783/4117		8349
A9		5	13876785	DNAH5	NM_001369.2	T	C/T	intron	none	NA	NA	
A9		6	38819306	DNAH8	NM_001206927.1	A	A/G	intron	none	NA	NA	
A9		9	34996447	DNAJB5	NM_001135004.2	G	A/G	missense	GLU,LYS	247/463		739
A9		2	225637990	DOCK10	NM_014689.2	C	C/G	missense	GLU,GLN	2030/2187		6088
A9		7	111418353	DOCK4	NM_014705.3	G	C/G	missense	LEU,VAL	1211/1967		3631
A9		9	379717	DOCK8	NM_001190458.1	C	C/T	intron	none	NA	NA	
A9		9	421003	DOCK8	NM_001190458.1	G	G/T	missense	VAL,PHE	1260/2000		3778
A9		11	118968049	DPAGT1	NM_001382.3	T	C/T	intron	none	NA	NA	

A9	3	109052632	DPPA4	NM_018189.3	C	C/T	intron	none	NA	NA	
A9	12	102295052	DRAM1	NM_018370.2	G	G/T	intron	none	NA	NA	
A9	11	117302445	DSCAML1	NM_020693.2	C	A/C	splice-3	none	NA	NA	
A9	11	117374594	DSCAML1	NM_020693.2	G	C/G	coding-synonymous	none	835/2114		2505
A9	18	28916593	DSG1	NM_001942.2	C	C/T	intron	none	NA	NA	
A9	6	7564208	DSP	NM_001008844.1	G	G/T	intron	none	NA	NA	
A9	20	30457235	DUSP15	NM_001012644.1	C	C/G	intron	none	NA	NA	
A9	14	102463470	DYNC1H1	NM_001376.4	C	C/T	coding-synonymous	none	1221/4647		3663
A9	2	71781013	DYSF	NM_001130455.1	C	A/C	missense	SER,ARG	670/2082		2010
A9	6	20480225	E2F3	NM_001243076.1	G	A/G	intron	none	NA	NA	
A9	1	41946763	EDN2	NM_001956.3	C	C/T	intron	none	NA	NA	
A9	13	78492268	EDNRB	NM_000115.3	G	G/T	missense	ASP,GLU	147/443		441
A9	22	43950887	EFCAB6	NM_022785.3	G	G/T	coding-synonymous	none	1170/1502		3510
A9	6	52344180	EFHC1	NM_001172420.1	A	A/G	intron	none	NA	NA	
A9	2	25326712	EFR3B	NM_014971.1	G	G/T	intron	none	NA	NA	
A9	15	82520535	EFTUD1	NM_001040610.2	G	A/G	missense	LEU,PHE	305/1070		913
A9	5	38412703	EGFLAM	NM_001205301.1	G	G/T	missense	GLY,TRP	483/1018		1447
A9	2	31472360	EHD3	NM_014600.2	G	G/T	intron	none	NA	NA	
A9	3	184909967	EHHADH	NM_001166415.1	C	A/C	utr-3	none	NA	NA	
A9	8	117767880	EIF3H	NM_003756.2	C	C/T	intron	none	NA	NA	
A9	3	184043122	EIF4G1	NM_001194946.1	C	C/T	coding-synonymous	none	981/1607		2943
A9	11	10823677	EIF4G2	NM_001042559.2	G	A/G	coding-synonymous	none	406/870		1218
A9	7	37382380	ELMO1	NM_001206480.1	G	A/G	intron	none	NA	NA	
A9	20	45000260	ELMO2	NM_133171.3	C	A/C	stop-gained	GLU,stop	551/721		1651
A9	4	101368693	EMCN	NM_001159694.1	G	G/T	intron	none	NA	NA	
A9	2	55179771	EML6	NM_001039753.2	C	C/T	coding-synonymous	none	1418/1959		4254
A9	8	120608323	ENPP2	NM_001040092.2	T	A/T	intron	none	NA	NA	
A9	8	110348433	ENY2	NM_001193557.1	G	G/T	missense-near-splice	ARG,LEU	23/97		68
A9	20	34778129	EPB41L1	NM_001258329.1	A	A/T	intron	none	NA	NA	
A9	3	89157055	EPHA3	NM_005233.5	C	A/C	intron	none	NA	NA	
A9	4	66231640	EPHA5	NM_004439.5	A	A/T	intron	none	NA	NA	
A9	17	19232749	EPN2	NM_001102664.1	C	C/T	intron	none	NA	NA	
A9	12	56474126	ERBB3	NM_001005915.1	C	C/T	coding-synonymous	none	14/184		42
A9	2	212529932	ERBB4	NM_001042599.1	T	C/T	intron	none	NA	NA	
A9	3	55733440	ERC2	NM_015576.1	G	A/G	missense	SER,LEU	938/958		2813
A9	9	102782868	ERP44	NM_015051.1	C	A/C	intron	none	NA	NA	
A9	20	13750660	ESF1	NM_016649.3	G	G/T	missense	PRO,THR	471/852		1411
A9	14	64749417	ESR2	NM_001040275.1	G	A/G	missense	SER,LEU	96/496		287
A9	8	95705147	ESRP1	NM_001034915.2	G	A/G	intron	none	NA	NA	
A9	12	56530579	ESYT1	NM_001184796.1	G	G/T	missense	ALA,SER	572/1115		1714
A9	2	67637185	ETAA1	NM_019002.3	G	C/G	utr-3	none	NA	NA	
A9	17	74077375	EXOC7	NM_001013839.2	C	A/C	utr-3	none	NA	NA	
A9	11	44135832	EXT2	NM_000401.3	C	C/G	missense	LEU,VAL	275/752		823

A9	6	65302976	EYS	NM_001142800.1	C	C/G	intron	none	NA	NA	
A9	17	40856659	EZH1	NM_001991.3	C	C/T	missense	ASP,ASN	660/748		1978
A9	6	123101515	FABP7	NM_001446.3	C	A/C	coding-synonymous	none	51/133		153
A9	2	45379	FAM110C	NM_001077710.2	C	C/T	intron	none	NA	NA	
A9	5	16478048	FAM134B	NM_001034850.2	G	A/G	intron	none	NA	NA	
A9	8	139207522	FAM135B	NM_015912.3	A	A/T	coding-synonymous	none	284/1407		852
A9	8	139380345	FAM135B	NM_015912.3	C	C/G	intron	none	NA	NA	
A9	5	137277825	FAM13B	NM_001101800.1	C	C/T	intron	none	NA	NA	
A9	5	177156685	FAM153A	NM_173663.3	G	C/G	intron	none	NA	NA	
A9	10	116590548	FAM160B1	NM_001135051.1	T	A/T	intron	none	NA	NA	
A9	7	135421825	FAM180A	NM_205855.3	G	C/G	intron	none	NA	NA	
A9	9	71998864	FAM189A2	NM_001127608.1	G	G/T	missense-near-splice	GLY,VAL	209/451		626
A9	13	46161357	FAM194B	NM_182542.2	C	A/C	missense	ALA,SER	233/697		697
A9	4	159048729	FAM198B	NM_001031700.2	G	A/G	missense	HIS,TYR	472/528		1414
A9	4	159091679	FAM198B	NM_001031700.2	G	G/T	missense	ASP,GLU	283/528		849
A9	10	89117778	FAM22D	NM_001009610.1	T	C/T	utr-5	none	NA	NA	
A9	7	129084033	FAM40B	NM_001134336.1	C	C/T	intron	none	NA	NA	
A9	1	78268978	FAM73A	NM_001270384.1	C	C/T	stop-gained	GLN,stop	133/634		397
A9	9	90744643	FAM75C2	NM_001166137.1	T	G/T	missense	GLU,ASP	1103/1135		3309
A9	15	59806676	FAM81A	NM_152450.2	C	A/C	intron	none	NA	NA	
A9	5	150947114	FAT2	NM_001447.2	C	C/T	missense	ARG,LYS	460/4350		1379
A9	4	126371698	FAT4	NM_024582.4	G	G/T	missense	GLY,VAL	3176/4982		9527
A9	14	92343775	FBLN5	NM_006329.3	G	A/G	intron	none	NA	NA	
A9	17	18647508	FBXW10	NM_001267585.1	G	A/G	utr-5	none	NA	NA	
A9	1	207131762	FCAMR	NM_001122979.2	C	C/T	utr-3	none	NA	NA	
A9	1	159277607	FCER1A	NM_002001.3	C	C/T	missense	THR,ILE	220/258		659
A9	11	72553674	FCHSD2	NM_014824.2	C	C/G	missense	GLU,GLN	640/741		1918
A9	1	157668038	FCRL3	NM_052939.3	C	C/G	intron	none	NA	NA	
A9	1	157485761	FCRL5	NM_001195388.1	G	A/G	intron	none	NA	NA	
A9	2	97335910	FER1L5	NM_001113382.1	G	G/T	stop-gained	GLU,stop	468/2094		1402
A9	12	2908917	FKBP4	NM_002014.3	G	G/T	intron	none	NA	NA	
A9	7	72745684	FKBP6	NM_001135211.1	C	C/T	stop-gained	GLN,stop	160/323		478
A9	7	96115813	FLJ42280	NM_001201450.1	G	G/T	intron	none	NA	NA	
A9	7	96125676	FLJ42280	NM_001201450.1	T	G/T	intron	none	NA	NA	
A9	3	57994238	FLNB	NM_001164317.1	C	C/G	utr-5	none	NA	NA	
A9	13	28913440	FLT1	NM_002019.4	G	C/G	intron	none	NA	NA	
A9	13	28578119	FLT3	NM_004119.2	C	C/G	utr-3	none	NA	NA	
A9	9	132658249	FNBP1	NM_015033.2	C	A/C	missense	VAL,PHE	572/618		1714
A9	9	132665179	FNBP1	NM_015033.2	T	C/T	missense	GLU,GLY	466/618		1397
A9	6	159670158	FNDC1	NM_032532.2	C	A/C	missense	ALA,ASP	1593/1895		4778
A9	8	42914140	FNTA	NM_002027.2	C	C/T	intron	none	NA	NA	
A9	14	65499485	FNTB	NM_001202558.1	A	A/T	intron	none	NA	NA	
A9	9	20820853	FOCAD	NM_017794.3	T	C/T	intron	none	NA	NA	

A9	13	32785076	FRY	NM_023037.2	A	A/G	missense	GLN,ARG	1499/3014		4496
A9	4	48503538	FRYL	NM_015030.1	C	C/G	intron	none	NA	NA	
A9	19	35651707	FXYD5	NM_001164605.1	A	A/G	intron	none	NA	NA	
A9	5	39137982	FYB	NM_001243093.1	T	G/T	intron	none	NA	NA	
A9	4	46125843	GABRG1	NM_173536.3	G	G/T	missense	LEU,MET	30/466		88
A9	15	27725992	GABRG3	NM_033223.4	G	G/T	intron	none	NA	NA	
A9	2	158115244	GALNT5	NM_014568.1	A	A/T	missense	ASN,ILE	217/941		650
A9	4	173232751	GALNTL6	NM_001034845.2	T	C/T	intron	none	NA	NA	
A9	11	22759309	GAS2	NM_001143830.1	A	A/G	coding-synonymous	none	156/314		468
A9	10	8105736	GATA3	NM_001002295.1	G	G/T	intron	none	NA	NA	
A9	2	27731204	GCKR	NM_001486.3	C	A/C	intron	none	NA	NA	
A9	12	7843212	GDF3	NM_020634.1	C	C/G	missense	ARG,SER	119/365		357
A9	6	55264030	GFRAL	NM_207410.2	T	A/T	missense	HIS,GLN	335/395		1005
A9	17	61995983	GH1	NM_000515.3	G	C/G	intron	none	NA	NA	
A9	7	150325392	GIMAP6	NM_001244071.1	G	G/T	intron	none	NA	NA	
A9	2	69172496	GKN2	NM_182536.2	C	A/C	utr-3	none	NA	NA	
A9	4	175598325	GLRA3	NM_001042543.1	G	G/T	coding-synonymous	none	277/450		831
A9	3	52729234	GLT8D1	NM_001010983.1	G	A/G	coding-synonymous	none	299/372		897
A9	11	58711177	GLYATL1	NM_001220494.1	G	A/G	intron	none	NA	NA	
A9	11	58723396	GLYATL1	NM_001220494.1	G	C/G	missense	GLU,GLN	269/303		805
A9	12	6952126	GNB3	NM_002075.2	C	C/G	intron	none	NA	NA	
A9	1	204170714	GOLT1A	NM_198447.1	C	C/T	intron	none	NA	NA	
A9	7	23309676	GPNMB	NM_001005340.1	G	A/G	missense	MET,ILE	449/573		1347
A9	4	22389500	GPR125	NM_145290.2	G	G/T	missense	ALA,GLU	1265/1322		3794
A9	3	100368656	GPR128	NM_032787.2	G	C/G	intron	none	NA	NA	
A9	8	142367991	GPR20	NM_005293.2	G	G/T	coding-synonymous	none	11/359		33
A9	14	88478101	GPR65	NM_003608.3	A	A/T	missense	ILE,LEU	304/338		910
A9	5	89975444	GPR98	NM_032119.3	G	A/G	missense	ARG,HIS	1841/6307		5522
A9	11	123475955	GRAMD1B	NM_020716.1	G	G/T	intron	none	NA	NA	
A9	5	152870660	GRIA1	NM_000827.3	C	A/C	intron	none	NA	NA	
A9	11	105795530	GRIA4	NM_000829.3	T	A/T	intron	none	NA	NA	
A9	4	94376856	GRID2	NM_001510.2	G	G/T	missense	ARG,LEU	530/1008		1589
A9	7	126409794	GRM8	NM_000845.2	A	A/T	intron	none	NA	NA	
A9	12	13237938	GSG1	NM_001080554.2	C	C/G	utr-3	none	NA	NA	
A9	11	106856964	GUCY1A2	NM_000855.2	G	C/G	intron	none	NA	NA	
A9	6	105280972	HACE1	NM_020771.3	T	A/T	missense	GLU,VAL	160/910		479
A9	20	7915258	HAO1	NM_017545.2	G	A/G	coding-synonymous	none	54/371		162
A9	20	30681721	HCK	NM_001172129.1	G	A/G	missense	ARG,GLN	362/506		1085
A9	5	45396745	HCN1	NM_021072.3	C	A/C	missense	GLY,VAL	360/891		1079
A9	6	55119838	HCRTR2	NM_001526.3	T	A/T	intron	none	NA	NA	
A9	14	31638756	HECTD1	NM_015382.2	G	C/G	intron	none	NA	NA	
A9	7	43547665	HECW1	NM_015052.3	G	G/T	coding-synonymous	none	1267/1607		3801
A9	17	65144725	HELZ	NM_014877.3	G	C/G	missense	LEU,VAL	861/1943		2581

A9	7	35673253	HERPUD2	NM_022373.4	A	A/T	utr-3	none	NA	NA
A9	3	57233913	HESX1	NM_003865.2	C	A/C	missense	GLY,TRP	12/186	34
A9	7	75197621	HIP1	NM_001243198.1	C	A/C	intron	none	NA	NA
A9	12	123341157	HIP1R	NM_003959.1	G	A/G	intron	none	NA	NA
A9	11	33375137	HIPK3	NM_001048200.1	C	C/G	utr-3	none	NA	NA
A9	6	26056336	HIST1H1C	NM_005319.3	G	A/G	coding-synonymous	none	107/214	321
A9	1	185956721	HMCN1	NM_031935.2	A	A/C	intron	none	NA	NA
A9	7	26236251	HNRNPA2B1	NM_002137.3	T	C/T	missense	HIS,ARG	168/342	503
A9	12	122292612	HPD	NM_001171993.1	C	C/T	coding-synonymous	none	98/355	294
A9	18	22057513	HRH4	NM_001143828.1	C	C/G	stop-gained	SER,stop	299/303	896
A9	1	162766299	HSD17B7	NM_016371.2	C	C/G	intron	none	NA	NA
A9	14	65008258	HSPA2	NM_021979.3	C	C/T	coding-synonymous	none	231/640	691
A9	4	128722487	HSPA4L	NM_014278.2	G	G/T	intron	none	NA	NA
A9	11	20398147	HTATIP2	NM_001098520.1	C	C/G	missense	ARG,GLY	143/277	427
A9	X	113887140	HTR2C	NM_000868.2	T	A/A	intron	none	NA	NA
A9	X	113887196	HTR2C	NM_000868.2	G	T/T	intron	none	NA	NA
A9	3	183823783	HTR3E	NM_001256613.1	G	C/G	intron	none	NA	NA
A9	16	71149479	HYDIN	NM_001198542.1	C	C/T	intron	none	NA	NA
A9	14	76455173	IFT43	NM_001102564.1	C	C/T	intron	none	NA	NA
A9	15	65627798	IGDCC3	NM_004884.3	C	C/T	intron	none	NA	NA
A9	17	47109355	IGF2BP1	NM_001160423.1	G	G/T	intron	none	NA	NA
A9	3	118621391	IGSF11	NM_001015887.1	C	C/G	missense	GLN,HIS	424/432	1272
A9	11	133801502	IGSF9B	NM_014987.1	G	A/G	intron	none	NA	NA
A9	19	18187015	IL12RB1	NM_005535.1	A	A/G	intron	none	NA	NA
A9	2	103039608	IL18RAP	NM_003853.2	C	A/C	intron	none	NA	NA
A9	5	35875958	IL7R	NM_002185.3	G	G/T	intron	none	NA	NA
A9	4	143043309	INPP4B	NM_001101669.1	C	A/C	missense	ASP,TYR	703/925	2107
A9	1	44427047	IPO13	NM_014652.3	G	A/G	intron	none	NA	NA
A9	14	24653055	IPO4	NM_024658.3	T	A/T	missense	ASP,VAL	640/1082	1919
A9	11	9441967	IPO7	NM_006391.2	C	C/T	stop-gained	GLN,stop	246/1039	736
A9	15	78780444	IREB2	NM_004136.2	T	C/T	intron	none	NA	NA
A9	16	313904	ITFG3	NM_032039.2	G	A/G	intron	none	NA	NA
A9	10	15647734	ITGA8	NM_003638.1	C	C/T	coding-synonymous	none	653/1064	1959
A9	16	31425801	ITGAD	NM_005353.2	C	C/G	missense	LEU,VAL	676/1162	2026
A9	16	31429527	ITGAD	NM_005353.2	C	A/C	intron	none	NA	NA
A9	3	52817087	ITIH1	NM_001166434.2	G	A/G	missense	GLU,LYS	207/770	619
A9	9	5080531	JAK2	NM_004972.3	A	A/G	splice-3	none	NA	NA
A9	20	42747217	JPH2	NM_020433.4	G	G/T	missense	LEU,MET	406/697	1216
A9	3	123813573	KALRN	NM_001024660.3	G	C/G	utr-5	none	NA	NA
A9	7	150649621	KCNH2	NM_000238.3	G	A/G	coding-synonymous	none	483/1160	1449
A9	1	23384090	KDM1A	NM_001009999.2	C	A/C	intron	none	NA	NA
A9	4	55984911	KDR	NM_002253.2	C	A/C	missense	ARG,MET	73/1357	218
A9	8	136657454	KHDRBS3	NM_006558.1	G	G/T	intron	none	NA	NA

A9		16	15694357	KIAA0430	NM_001184998.1	C	C/T	missense	GLU,LYS	1581/1743		4741
A9		4	123122098	KIAA1109	NM_015312.3	C	C/G	intron	none	NA	NA	
A9		7	86544218	KIAA1324L	NM_001142749.2	C	C/G	intron	none	NA	NA	
A9		3	113775178	KIAA1407	NM_020817.1	C	A/C	intron	none	NA	NA	
A9		11	33572805	KIAA1549L	NM_012194.2	G	G/T	missense-near-splic	VAL,LEU	944/1850		2830
A9		2	176812453	KIAA1715	NM_030650.1	T	C/T	intron	none	NA	NA	
A9		2	8890205	KIDINS220	NM_020738.2	A	A/G	intron	none	NA	NA	
A9		11	28045489	KIF18A	NM_031217.3	T	A/T	intron	none	NA	NA	
A9		1	45232773	KIF2C	NM_006845.3	C	C/G	coding-synonymous	none	700/726		2100
A9	X		69606609	KIF4A	NM_012310.4	G	A/A	intron	none	NA	NA	
A9		6	39688399	KIF6	NM_145027.4	T	A/T	intron	none	NA	NA	
A9		4	55594034	KIT	NM_000222.2	C	A/C	missense	THR,ASN	607/977		1820
A9		4	55604704	KIT	NM_000222.2	T	A/T	missense	LEU,HIS	971/977		2912
A9		2	170606138	KLHL23	NM_001199290.1	C	C/G	missense	GLN,GLU	525/559		1573
A9		19	51452075	KLK5	NM_001077491.1	G	G/T	intron	none	NA	NA	
A9		19	51453452	KLK5	NM_001077491.1	C	C/T	intron	none	NA	NA	
A9		12	53071746	KRT1	NM_006121.3	G	G/T	intron	none	NA	NA	
A9		12	53342925	KRT8	NM_000224.2	C	C/G	utr-5	none	NA	NA	
A9		21	31962646	KRTAP22-2	NM_001164434.1	G	C/G	missense	SER,ARG	16/46		48
A9		17	25936266	KSR1	NM_014238.1	C	C/G	coding-synonymous	none	597/763		1791
A9		6	108843595	LACE1	NM_145315.3	G	A/G	coding-synonymous	none	471/482		1413
A9		6	129636938	LAMA2	NM_000426.3	C	C/T	missense	ALA,VAL	1256/3123		3767
A9		1	183101775	LAMC1	NM_002293.3	T	C/T	intron	none	NA	NA	
A9		1	183201737	LAMC2	NM_005562.2	C	C/G	intron	none	NA	NA	
A9		12	50822769	LARP4	NM_001170803.1	C	C/T	missense	SER,PHE	73/583		218
A9		7	73631094	LAT2	NM_014146.3	T	G/T	intron	none	NA	NA	
A9		13	21553833	LATS2	NM_014572.2	C	C/T	coding-synonymous	none	923/1089		2769
A9		1	32742015	LCK	NM_001042771.1	G	A/G	missense	GLU,LYS	237/510		709
A9		1	43212355	LEPRE1	NM_001146289.1	G	C/G	utr-3	none	NA	NA	
A9		1	202287831	LGR6	NM_001017403.1	C	C/T	coding-synonymous	none	800/968		2400
A9		7	104546760	LHFPL3	NM_199000.2	G	C/G	utr-3	none	NA	NA	
A9		7	104546789	LHFPL3	NM_199000.2	G	C/G	utr-3	none	NA	NA	
A9		19	55175179	LILRB4	NM_001081438.1	G	G/T	intron	none	NA	NA	
A9		12	50589532	LIMA1	NM_001113546.1	G	A/G	intron	none	NA	NA	
A9		7	73513564	LIMK1	NM_001204426.1	C	C/T	stop-gained	GLN,stop	168/614		502
A9		15	75105343	LMAN1L	NM_021819.2	G	G/T	stop-gained	GLY,stop	50/527		148
A9		13	76414567	LMO7	NM_005358.5	G	A/G	stop-gained	TRP,stop	891/1350		2672
A9		6	160963977	LPA	NM_005577.2	T	C/T	intron	none	NA	NA	
A9		5	1479591	LPCAT1	NM_024830.3	G	A/G	intron	none	NA	NA	
A9		19	821391	LPPR3	NM_001270366.1	C	C/T	intron	none	NA	NA	
A9		4	151738457	LRBA	NM_001199282.2	G	C/G	intron	none	NA	NA	
A9		13	47289876	LRCH1	NM_001164211.1	G	G/T	intron	none	NA	NA	
A9		13	47315637	LRCH1	NM_001164211.1	G	A/G	intron	none	NA	NA	

A9	2	141812864	LRP1B	NM_018557.2	A	A/G	intron	none	NA	NA	
A9	4	52860968	LRRC66	NM_001024611.1	T	C/T	coding-synonymous	none	740/881		2220
A9	1	70486840	LRRC7	NM_020794.2	T	G/T	intron	none	NA	NA	
A9	6	43475172	LRRC73	NM_001012974.1	G	C/G	intron	none	NA	NA	
A9	8	86049756	LRRCC1	NM_033402.4	G	C/G	missense	ARG,THR	796/1033		2387
A9	1	74646535	LRRIQ3	NM_001105659.1	C	C/T	intron	none	NA	NA	
A9	15	101562620	LRRK1	NM_024652.3	C	C/G	missense	GLN,GLU	629/2016		1885
A9	9	130224515	LRSAM1	NM_001005373.3	A	A/G	intron	none	NA	NA	
A9	21	30302786	LTN1	NM_015565.2	C	A/C	missense	ARG,LEU	1808/1813		5423
A9	2	160710182	LY75	NM_001198759.1	C	A/C	missense	TRP,CYS	874/1874		2622
A9	8	143846229	LYNX1	NM_023946.2	T	A/T	intron	none	NA	NA	
A9	1	39901172	MACF1	NM_012090.4	C	A/C	intron	none	NA	NA	
A9	X	140995881	MAGEC1	NM_005462.4	G	C/C	missense	LEU,PHE	897/1143		2691
A9	X	49019343	MAGIX	NM_001099681.1	C	T/T	intron	none	NA	NA	
A9	9	139746963	MAMDC4	NM_206920.2	C	C/T	intron	none	NA	NA	
A9	5	179192397	MAML1	NM_014757.4	G	G/T	missense	GLY,VAL	129/1017		386
A9	12	53880410	MAP3K12	NM_001193511.1	T	C/T	intron	none	NA	NA	
A9	3	48039993	MAP4	NM_001134364.1	C	C/G	intron	none	NA	NA	
A9	2	102456244	MAP4K4	NM_001242559.1	G	A/G	intron	none	NA	NA	
A9	22	22142896	MAPK1	NM_002745.4	G	C/G	intron	none	NA	NA	
A9	8	99045815	MATN2	NM_002380.3	C	C/G	missense	GLN,GLU	915/957		2743
A9	8	99047887	MATN2	NM_002380.3	G	C/G	missense	GLU,GLN	940/957		2818
A9	6	52143784	MCM3	NM_001270472.1	T	A/T	intron	none	NA	NA	
A9	21	47662772	MCM3AP	NM_003906.3	C	A/C	missense	LEU,PHE	1790/1981		5370
A9	3	168845735	MECOM	NM_001105077.3	C	C/T	missense	GLU,LYS	119/1117		355
A9	1	29529807	MECR	NM_001024732.1	C	C/T	intron	none	NA	NA	
A9	16	3304384	MEFV	NM_000243.2	G	A/G	coding-synonymous	none	228/782		684
A9	10	15008652	MEIG1	NM_001080836.2	G	A/G	intron	none	NA	NA	
A9	2	66795785	MEIS1	NM_002398.2	C	C/G	intron	none	NA	NA	
A9	15	37385840	MEIS2	NM_001220482.1	C	C/T	missense	GLY,ASP	194/471		581
A9	3	158541771	MFSD1	NM_001167903.1	C	C/G	intron	none	NA	NA	
A9	11	12260885	MICAL2	NM_014632.2	C	C/T	intron	none	NA	NA	
A9	1	12082851	MIIP	NM_021933.3	C	C/T	intron	none	NA	NA	
A9	7	151962446	MLL3	NM_170606.2	C	C/T	intron	none	NA	NA	
A9	7	151970714	MLL3	NM_170606.2	C	A/C	intron	none	NA	NA	
A9	8	89054156	MMP16	NM_005941.4	A	A/G	intron	none	NA	NA	
A9	1	113237523	MOV10	NM_001130079.1	G	A/G	intron	none	NA	NA	
A9	9	13126667	MPDZ	NM_001261406.1	G	C/G	intron	none	NA	NA	
A9	1	156708286	MRPL24	NM_024540.3	C	C/T	intron	none	NA	NA	
A9	17	36453270	MRPL45	NM_032351.4	G	A/G	intron	none	NA	NA	
A9	11	73556055	MRPL48	NM_016055.5	G	C/G	intron	none	NA	NA	
A9	8	98718765	MTDH	NM_178812.3	A	A/T	intron	none	NA	NA	
A9	22	30822674	MTFP1	NM_001003704.2	G	C/G	intron	none	NA	NA	

A9	22	30412671	MTMR3	NM_021090.3	C	C/T	missense	SER,PHE	553/1199		1658
A9	1	11194547	MTOR	NM_004958.3	G	A/G	intron	none	NA	NA	
A9	1	11319475	MTOR	NM_004958.3	G	A/G	utr-5	none	NA	NA	
A9	4	100503179	MTTP	NM_000253.2	C	C/T	missense	SER,PHE	60/895		179
A9	13	29599249	MTUS2	NM_001033602.2	G	C/G	coding-synonymous	none	148/1380		444
A9	13	29599411	MTUS2	NM_001033602.2	G	A/G	coding-synonymous	none	202/1380		606
A9	19	8966823	MUC16	NM_024690.2	G	C/G	intron	none	NA	NA	
A9	19	9010958	MUC16	NM_024690.2	G	A/G	intron	none	NA	NA	
A9	11	1267955	MUC5B	NM_002458.2	C	C/G	missense	THR,SER	3282/5763		9845
A9	11	47369499	MYBPC3	NM_000256.3	G	C/G	intron	none	NA	NA	
A9	13	77631208	MYCBP2	NM_015057.4	G	A/G	coding-synonymous	none	4450/4679		13350
A9	17	10224727	MYH13	NM_003802.2	C	C/T	intron	none	NA	NA	
A9	14	23900682	MYH7	NM_000257.2	G	G/T	missense	PHE,LEU	247/1936		741
A9	20	33580207	MYH7B	NM_020884.3	G	A/G	intron	none	NA	NA	
A9	17	18062191	MYO15A	NM_016239.3	G	C/G	intron	none	NA	NA	
A9	13	109859053	MYO16	NM_001198950.1	C	A/C	missense	GLN,LYS	1838/1881		5512
A9	12	57440868	MYO1A	NM_001256041.1	G	A/G	missense	ARG,CYS	152/1044		454
A9	15	59497603	MYO1E	NM_004998.3	C	C/T	missense	GLU,LYS	538/1109		1612
A9	15	52504092	MYO5C	NM_018728.3	G	A/G	intron	none	NA	NA	
A9	5	150042609	MYOZ3	NM_001122853.2	C	C/T	intron	none	NA	NA	
A9	2	1907130	MYT1L	NM_015025.2	C	C/T	intron	none	NA	NA	
A9	16	66857560	NAE1	NM_001018159.1	C	A/C	intron	none	NA	NA	
A9	2	73868272	NAT8	NM_003960.3	G	G/T	missense	LEU,ILE	162/228		484
A9	2	15378564	NBAS	NM_015909.3	G	G/T	intron	none	NA	NA	
A9	3	47037294	NBEAL2	NM_015175.2	G	C/G	missense	LEU,PHE	663/2755		1989
A9	1	16908903	NBPF1	NM_017940.3	G	C/G	intron	none	NA	NA	
A9	11	134047144	NCAPD3	NM_015261.2	C	C/G	missense	GLN,HIS	997/1499		2991
A9	17	8370334	NDEL1	NM_001025579.2	G	C/G	utr-3	none	NA	NA	
A9	4	115751176	NDST4	NM_022569.1	A	A/G	intron	none	NA	NA	
A9	7	123185795	NDUFA5	NM_005000.2	A	A/T	intron	none	NA	NA	
A9	19	1393208	NDUFS7	NM_024407.4	C	C/T	intron	none	NA	NA	
A9	8	91929885	NECAB1	NM_022351.4	A	A/G	intron	none	NA	NA	
A9	18	56033318	NEDD4L	NM_001144964.1	C	C/G	missense	PRO,ALA	520/855		1558
A9	4	170327832	NEK1	NM_001199397.1	C	C/T	missense	VAL,ILE	1097/1287		3289
A9	11	20969131	NELL1	NM_006157.3	C	C/T	intron	none	NA	NA	
A9	20	50049264	NFATC2	NM_001136021.2	C	A/C	stop-gained	GLU,stop	668/902		2002
A9	16	28970250	NFATC2IP	NM_032815.3	C	C/G	intron	none	NA	NA	
A9	20	34262237	NFS1	NM_001198989.1	C	A/C	intron	none	NA	NA	
A9	14	52520376	NID2	NM_007361.3	A	A/G	coding-synonymous	none	450/1376		1350
A9	6	124125432	NKAIN2	NM_001040214.1	T	C/T	intron	none	NA	NA	
A9	1	247597585	NLRP3	NM_001079821.2	G	G/T	intron	none	NA	NA	
A9	3	139279718	NMNAT3	NM_001200047.1	C	C/G	utr-3	none	NA	NA	
A9	9	33473872	NOL6	NM_022917.4	G	A/G	utr-5	none	NA	NA	

A9	1	148344491	none	none	T	A/T	intergenic	none	NA	NA
A9	1	171621867	none	none	C	C/T	near-gene-5	none	NA	NA
A9	1	194029227	none	none	G	C/G	intergenic	none	NA	NA
A9	2	89292438	none	none	G	A/G	intergenic	none	NA	NA
A9	2	90139644	none	none	C	A/C	intergenic	none	NA	NA
A9	2	179372785	none	none	T	G/T	intergenic	none	NA	NA
A9	3	27148278	none	none	C	C/G	intergenic	none	NA	NA
A9	3	69187925	none	none	G	C/G	intergenic	none	NA	NA
A9	3	98002683	none	none	T	C/T	near-gene-3	none	NA	NA
A9	4	178911558	none	none	G	A/G	intergenic	none	NA	NA
A9	4	190905711	none	none	C	C/G	intergenic	none	NA	NA
A9	6	133138829	none	none	G	C/G	near-gene-3	none	NA	NA
A9	7	72338030	none	none	G	A/G	intergenic	none	NA	NA
A9	7	142364442	none	none	C	A/C	intergenic	none	NA	NA
A9	7	142479900	none	none	C	C/T	intergenic	none	NA	NA
A9	8	22109615	none	none	G	G/T	near-gene-3	none	NA	NA
A9	8	49666480	none	none	T	G/T	intergenic	none	NA	NA
A9	9	89591723	none	none	G	C/G	intergenic	none	NA	NA
A9	9	115125217	none	none	G	C/G	intergenic	none	NA	NA
A9	10	51630926	none	none	G	G/T	intergenic	none	NA	NA
A9	10	51630953	none	none	C	C/T	intergenic	none	NA	NA
A9	11	62186427	none	none	T	C/T	near-gene-5	none	NA	NA
A9	11	118852001	none	none	C	C/T	near-gene-3	none	NA	NA
A9	12	9573125	none	none	C	C/T	intergenic	none	NA	NA
A9	12	48654162	none	none	C	A/C	intergenic	none	NA	NA
A9	12	95702283	none	none	T	C/T	intergenic	none	NA	NA
A9	14	22038888	none	none	C	C/G	near-gene-5	none	NA	NA
A9	14	101490256	none	none	G	G/T	intergenic	none	NA	NA
A9	14	106111195	none	none	C	C/G	intergenic	none	NA	NA
A9	14	106376491	none	none	G	A/G	intergenic	none	NA	NA
A9	14	106757603	none	none	G	G/T	intergenic	none	NA	NA
A9	14	106805173	none	none	G	G/T	intergenic	none	NA	NA
A9	14	106805181	none	none	G	C/G	intergenic	none	NA	NA
A9	14	107048592	none	none	C	A/C	intergenic	none	NA	NA
A9	15	43991450	none	none	G	C/G	near-gene-3	none	NA	NA
A9	16	33020971	none	none	C	A/C	intergenic	none	NA	NA
A9	16	88620471	none	none	C	C/G	intergenic	none	NA	NA
A9	17	6921238	none	none	G	A/G	near-gene-3	none	NA	NA
A9	18	19192247	none	none	G	C/G	near-gene-5	none	NA	NA
A9	19	54189708	none	none	G	C/G	intergenic	none	NA	NA
A9	21	38593747	none	none	C	C/T	near-gene-3	none	NA	NA
A9	22	23263429	none	none	T	C/T	intergenic	none	NA	NA
A9	22	32072970	none	none	C	C/T	intergenic	none	NA	NA

A9	22	32435600	none	none	C	C/T	intergenic	none	NA	NA	
A9	X	110776570	none	none	G	T/T	intergenic	none	NA	NA	
A9	6	155776106	NOX3	NM_015718.2	A	A/C	intron	none	NA	NA	
A9	15	24922422	NPAP1	NM_018958.2	G	C/G	missense	VAL,LEU	470/1157		1408
A9	5	32712623	NPR3	NM_000908.3	C	C/T	coding-synonymous	none	247/541		741
A9	17	38251262	NR1D1	NM_021724.3	C	C/T	missense	ALA,THR	454/615		1360
A9	2	206562395	NRP2	NM_003872.2	G	C/G	missense	LYS,ASN	67/927		201
A9	11	64436123	NRXN2	NM_015080.3	C	A/C	intron	none	NA	NA	
A9	14	79432554	NRXN3	NM_004796.4	C	A/C	missense	ALA,ASP	488/1062		1463
A9	8	126378989	NSMCE2	NM_173685.2	G	C/G	intron	none	NA	NA	
A9	15	88423603	NTRK3	NM_001012338.2	G	G/T	stop-gained	TYR,stop	744/840		2232
A9	14	32031351	NUBPL	NM_025152.2	G	A/G	coding-synonymous	none	62/320		186
A9	5	162884608	NUDCD2	NM_145266.4	G	C/G	coding-synonymous	none	66/158		198
A9	1	229613355	NUP133	NM_018230.2	G	A/G	missense	SER,PHE	582/1157		1745
A9	1	224468966	NVL	NM_001243146.1	C	A/C	intron	none	NA	NA	
A9	11	114442003	NXPE4	NM_001077639.1	A	A/G	missense	ILE,THR	431/545		1292
A9	7	8791187	NXPH1	NM_152745.2	G	G/T	missense	ASP,TYR	202/272		604
A9	8	133050992	OC90	NM_001080399.2	C	A/C	intron	none	NA	NA	
A9	15	28116858	OCA2	NM_000275.2	C	C/G	intron	none	NA	NA	
A9	7	44737390	OGDH	NM_001165036.1	C	C/T	intron	none	NA	NA	
A9	10	15103655	OLAH	NM_001039702.2	C	C/G	intron	none	NA	NA	
A9	11	133402371	OPCML	NM_001012393.1	G	C/G	utr-5	none	NA	NA	
A9	8	54142231	OPRK1	NM_000912.3	G	C/G	missense	ARG,GLY	257/381		769
A9	6	154429197	OPRM1	NM_000914.3	T	C/T	intron	none	NA	NA	
A9	6	154431445	OPRM1	NM_000914.3	T	A/T	intron	none	NA	NA	
A9	10	13154512	OPTN	NM_001008211.1	C	C/G	coding-synonymous	none	143/578		429
A9	11	57995622	OR10Q1	NM_001004471.2	G	A/G	coding-synonymous	none	242/320		726
A9	11	6942699	OR2D3	NM_001004684.1	T	G/T	missense	VAL,GLY	156/331		467
A9	11	55406702	OR4P4	NM_001004124.1	C	C/T	missense	THR,ILE	290/313		869
A9	11	5172944	OR52A1	NM_012375.2	A	A/G	missense	LEU,SER	219/313		656
A9	11	5510813	OR52D1	NM_001005163.2	C	A/C	missense	PRO,THR	293/319		877
A9	11	5080005	OR52E2	NM_001005164.2	G	C/G	missense	PRO,ALA	285/326		853
A9	3	98002531	OR5H2	NM_001005482.1	C	A/C	missense	PRO,HIS	267/315		800
A9	11	6815957	OR6A2	NM_003696.2	T	C/T	stop-lost	stop,TRP	328/328		983
A9	11	6816255	OR6A2	NM_003696.2	C	A/C	missense	ALA,SER	229/328		685
A9	1	247875981	OR6F1	NM_001005286.1	G	G/T	missense	SER,TYR	26/309		77
A9	11	123624733	OR6X1	NM_001005188.1	G	C/G	missense	PRO,ARG	165/313		494
A9	11	56058117	OR8H1	NM_001005199.1	C	A/C	missense	CYS,PHE	141/312		422
A9	11	57958334	OR9Q2	NM_001005283.2	G	A/G	coding-synonymous	none	124/315		372
A9	7	103848314	ORC5	NM_002553.3	A	A/G	coding-synonymous	none	13/436		39
A9	2	179255658	OSBPL6	NM_001201480.1	A	A/T	intron	none	NA	NA	
A9	4	109576901	OSTC	NM_001267817.1	G	G/T	intron	none	NA	NA	
A9	16	21747871	OTOA	NM_001161683.1	T	G/T	intron	none	NA	NA	

A9	2	26690286	OTOF	NM_004802.3	C	C/T	missense	GLY,SER	625/1231		1873
A9	5	41842688	OXCT1	NM_000436.3	G	A/G	intron	none	NA	NA	
A9	3	151094602	P2RY12	NM_022788.3	G	C/G	intron	none	NA	NA	
A9	4	57319915	PAICS	NM_001079524.1	C	C/G	missense	ARG,GLY	367/426		1099
A9	16	23641476	PALB2	NM_024675.3	C	C/T	missense	GLU,LYS	667/1187		1999
A9	4	169815668	PALLD	NM_001166108.1	A	A/T	intron	none	NA	NA	
A9	13	28855610	PAN3	NM_175854.7	G	A/G	intron	none	NA	NA	
A9	11	124487282	PANX3	NM_052959.2	A	A/T	missense	GLU,VAL	146/393		437
A9	1	176668407	PAPPA2	NM_020318.2	T	A/T	missense	PHE,TYR	973/1792		2918
A9	6	162394198	PARK2	NM_004562.2	C	A/C	intron	none	NA	NA	
A9	12	53856399	PCBP2	NM_001098620.2	G	C/G	intron	none	NA	NA	
A9	5	140236927	PCDHA9	NM_018900.2	C	A/C	intron	none	NA	NA	
A9	5	140250582	PCDHA9	NM_018900.2	A	A/G	intron	none	NA	NA	
A9	5	140480733	PCDHB3	NM_018937.2	T	A/T	missense	LEU,HIS	167/797		500
A9	15	44057824	PDIA3	NM_005313.4	C	C/G	intron	none	NA	NA	
A9	4	39874515	PDS5A	NM_001100399.1	G	C/G	intron	none	NA	NA	
A9	7	94293025	PEG10	NM_001040152.1	G	G/T	stop-gained	GLU,stop	53/326		157
A9	7	94293846	PEG10	NM_001040152.1	G	C/G	stop-lost	stop,TYR	326/326		978
A9	1	7879264	PER3	NM_016831.1	G	C/G	intron	none	NA	NA	
A9	4	152592509	PET112	NM_004564.2	C	C/T	intron	none	NA	NA	
A9	3	179519809	PEX5L	NM_001256750.1	C	C/G	missense	SER,THR	561/625		1682
A9	12	53693047	PFDN5	NM_002624.3	C	C/G	intron	none	NA	NA	
A9	6	144098580	PHACTR2	NM_001100164.1	G	A/G	intron	none	NA	NA	
A9	4	129789038	PHF17	NM_199320.2	C	C/T	missense	ARG,CYS	511/843		1531
A9	1	120263850	PHGDH	NM_006623.3	G	A/G	missense	ALA,THR	66/534		196
A9	18	60570193	PHLPP1	NM_194449.3	C	C/G	intron	none	NA	NA	
A9	1	114248637	PHTF1	NM_006608.2	G	C/G	missense	LEU,VAL	516/763		1546
A9	18	10680110	PIEZO2	NM_022068.2	C	C/T	intron	none	NA	NA	
A9	20	33173395	PIGU	NM_080476.4	G	C/G	intron	none	NA	NA	
A9	19	18271510	PIK3R2	NM_005027.2	G	A/G	intron	none	NA	NA	
A9	17	8741957	PIK3R6	NM_001010855.2	G	C/G	missense	SER,CYS	38/755		113
A9	X	15403057	PIR	NM_001018109.2	C	A/A	utr-3	none	NA	NA	
A9	7	47814773	PKD1L1	NM_138295.3	A	A/G	intron	none	NA	NA	
A9	8	110473910	PKHD1L1	NM_177531.4	G	A/G	intron	none	NA	NA	
A9	8	110511053	PKHD1L1	NM_177531.4	G	A/G	intron	none	NA	NA	
A9	3	119348179	PLA1A	NM_001206960.1	C	A/C	intron	none	NA	NA	
A9	9	26905472	PLAA	NM_001031689.2	C	A/C	utr-3	none	NA	NA	
A9	9	26910458	PLAA	NM_001031689.2	G	G/T	intron	none	NA	NA	
A9	20	8717924	PLCB1	NM_015192.2	G	C/G	intron	none	NA	NA	
A9	17	40825956	PLEKHH3	NM_024927.4	C	C/T	coding-synonymous	none	98/794		294
A9	20	21224888	PLK1S1	NM_001163022.1	A	A/G	missense	GLU,GLY	526/571		1576
A9	3	145878679	PLOD2	NM_000935.2	C	A/C	missense	SER,ILE	33/738		98
A9	7	100855305	PLOD3	NM_001084.4	C	A/C	intron	none	NA	NA	

A9	7	132192784	PLXNA4	NM_001105543.1	C	A/C	coding-synonymous	none	223/493		669
A9	3	48459796	PLXNB1	NM_001130082.1	G	A/G	intron	none	NA	NA	
A9	6	99857111	PNISR	NM_015491.1	G	A/G	missense	SER,LEU	204/806		611
A9	5	74877102	POLK	NM_016218.2	G	C/G	missense	GLU,GLN	255/871		763
A9	5	74879282	POLK	NM_016218.2	G	A/G	intron	none	NA	NA	
A9	13	28240083	POLR1D	NM_001206559.1	G	A/G	missense	ARG,GLN	93/95		278
A9	17	7402314	POLR2A	NM_000937.4	G	C/G	intron	none	NA	NA	
A9	1	145608446	POLR3C	NM_006468.6	C	A/C	missense	VAL,PHE	121/535		361
A9	14	77752936	POMT2	NM_013382.5	C	C/T	intron	none	NA	NA	
A9	6	105609812	POPDC3	NM_022361.4	C	A/C	utr-5	none	NA	NA	
A9	13	38154853	POSTN	NM_001135934.1	G	C/G	intron	none	NA	NA	
A9	8	43211878	POTEA	NM_001002920.1	C	C/T	intron	none	NA	NA	
A9	2	130872914	POTEF	NM_001099771.2	G	C/G	intron	none	NA	NA	
A9	3	87325460	POU1F1	NM_000306.2	G	C/G	intron	none	NA	NA	
A9	20	62153187	PPDPF	NM_024299.2	G	A/G	coding-synonymous	none	100/115		300
A9	12	80180231	PPP1R12A	NM_001143885.1	C	C/T	missense	GLU,LYS	944/1031		2830
A9	17	48222543	PPP1R9B	NM_032595.3	G	C/G	missense	SER,CYS	480/818		1435
A9	14	94696965	PPP4R4	NM_058237.1	T	A/T	coding-synonymous	none	112/874		336
A9	19	46888226	PPP5C	NM_001204284.1	C	C/G	intron	none	NA	NA	
A9	1	156766982	PRCC	NM_005973.4	C	C/T	intron	none	NA	NA	
A9	11	82560329	PRCP	NM_005040.2	T	A/T	intron	none	NA	NA	
A9	11	129814820	PRDM10	NM_020228.2	C	C/T	missense	ARG,GLN	203/1161		608
A9	5	23509329	PRDM9	NM_020227.2	C	C/T	intron	none	NA	NA	
A9	1	173450607	PRDX6	NM_004905.2	C	C/G	missense	LEU,VAL	80/225		238
A9	6	105733284	PREP	NM_002726.4	C	C/T	intron	none	NA	NA	
A9	7	151552271	PRKAG2	NM_016203.3	A	A/G	intron	none	NA	NA	
A9	17	64728986	PRKCA	NM_002737.2	G	C/G	intron	none	NA	NA	
A9	2	37496830	PRKD3	NM_005813.3	C	C/T	missense-near-splice	ASP,ASN	569/891		1705
A9	5	35086356	PRLR	NM_000949.5	C	C/G	missense	ASP,HIS	53/623		157
A9	1	171485028	PRRC2C	NM_015172.3	G	A/G	intron	none	NA	NA	
A9	1	171485077	PRRC2C	NM_015172.3	G	C/G	intron	none	NA	NA	
A9	16	2871244	PRSS21	NM_001270452.1	C	C/T	intron	none	NA	NA	
A9	3	46758918	PRSS50	NM_013270.4	G	A/G	intron	none	NA	NA	
A9	15	55975959	PRTG	NM_173814.4	C	C/G	intron	none	NA	NA	
A9	8	87076735	PSKH2	NM_033126.1	G	G/T	missense	ALA,GLU	104/386		311
A9	14	35778262	PSMA6	NM_002791.1	C	C/G	intron	none	NA	NA	
A9	14	35779825	PSMA6	NM_002791.1	G	A/G	intron	none	NA	NA	
A9	2	86359633	PTCD3	NM_017952.5	C	C/G	intron	none	NA	NA	
A9	2	120620254	PTPN4	NM_002830.3	G	A/G	intron	none	NA	NA	
A9	12	71091963	PTPRR	NM_001207015.1	C	C/T	intron	none	NA	NA	
A9	20	41408880	PTPRT	NM_007050.5	G	A/G	coding-synonymous	none	182/1442		546
A9	7	121652954	PTPRZ1	NM_001206838.1	C	C/G	intron	none	NA	NA	
A9	1	226108912	PYCR2	NM_013328.2	T	C/T	missense	THR,ALA	265/321		793

A9	16	538932	RAB11FIP3	NM_001142272.1	G	A/G	coding-synonymous	none	103/461	309
A9	8	117864086	RAD21	NM_006265.2	C	C/G	intron	none	NA	NA
A9	8	85799695	RALYL	NM_001100391.1	A	A/G	intron	none	NA	NA
A9	6	13711206	RANBP9	NM_005493.2	T	C/T	missense	ILE,VAL	178/730	532
A9	2	173865984	RAPGEF4	NM_001100397.1	C	C/T	intron	none	NA	NA
A9	11	63304498	RARRES3	NM_004585.3	A	A/G	intron	none	NA	NA
A9	12	113565738	RASAL1	NM_001193520.1	G	G/T	intron	none	NA	NA
A9	15	79341904	RASGRF1	NM_001145648.1	C	C/G	missense	GLU,ASP	186/1258	558
A9	8	53570186	RB1CC1	NM_001083617.1	C	A/C	missense	ALA,SER	735/1592	2203
A9	2	79348789	REG1A	NM_002909.4	A	A/T	missense	THR,SER	56/167	166
A9	7	103123423	RELN	NM_005045.3	G	C/G	intron	none	NA	NA
A9	7	103197556	RELN	NM_005045.3	C	A/C	stop-gained	GLY,stop	1889/3461	5665
A9	1	204124966	REN	NM_000537.3	G	A/G	coding-synonymous	none	347/407	1041
A9	1	163131782	RGS5	NM_001195303.2	G	C/G	intron	none	NA	NA
A9	5	96498865	RIOK2	NM_018343.2	C	C/T	missense	ARG,GLN	520/553	1559
A9	16	67680601	RLTPR	NM_001013838.1	C	A/C	intron	none	NA	NA
A9	1	145687115	RNF115	NM_014455.2	C	C/T	intron	none	NA	NA
A9	X	106038995	RNF128	NM_024539.3	C	T/T	utr-3	none	NA	NA
A9	7	5760784	RNF216	NM_207111.3	C	C/G	missense	LYS,ASN	508/924	1524
A9	7	156451355	RNF32	NM_001184996.1	G	C/G	intron	none	NA	NA
A9	11	124735664	ROBO3	NM_022370.3	C	C/G	intron	none	NA	NA
A9	11	124754600	ROBO4	NM_019055.5	C	C/T	utr-3	none	NA	NA
A9	1	151798505	RORC	NM_001001523.1	G	A/G	utr-5	none	NA	NA
A9	17	37359213	RPL19	NM_000981.3	G	A/G	intron	none	NA	NA
A9	14	75377976	RPS6KL1	NM_031464.4	G	G/T	coding-synonymous	none	213/550	639
A9	16	11933456	RSL1D1	NM_015659.2	C	A/C	intron	none	NA	NA
A9	7	5998522	RSPH10B	NM_173565.3	G	A/G	intron	none	NA	NA
A9	2	55200585	RTN4	NM_007008.2	C	A/C	intron	none	NA	NA
A9	4	159566117	RXFP1	NM_001253727.1	C	A/C	missense	PRO,GLN	418/785	1253
A9	3	45763628	SACM1L	NM_014016.3	G	G/T	intron	none	NA	NA
A9	13	23939407	SACS	NM_014363.4	G	C/G	missense	GLN,GLU	119/4580	355
A9	14	21993765	SALL2	NM_005407.1	G	C/G	missense	PRO,ALA	33/1008	97
A9	14	55255501	SAMD4A	NM_001161576.2	G	C/G	intron	none	NA	NA
A9	7	92734015	SAMD9	NM_001193307.1	G	A/G	missense	PRO,SER	466/1590	1396
A9	7	92734778	SAMD9	NM_001193307.1	G	C/G	coding-synonymous	none	211/1590	633
A9	6	155143363	SCAF8	NM_014892.3	A	A/G	intron	none	NA	NA
A9	11	61957733	SCGB1D1	NM_006552.1	G	A/G	utr-5	none	NA	NA
A9	3	38888471	SCN11A	NM_014139.2	G	A/G	missense	SER,PHE	1697/1792	5090
A9	3	38908993	SCN11A	NM_014139.2	G	C/G	intron	none	NA	NA
A9	3	38949602	SCN11A	NM_014139.2	G	A/G	coding-synonymous	none	437/1792	1311
A9	2	166915215	SCN1A	NM_001165963.1	C	A/C	intron	none	NA	NA
A9	2	166170314	SCN2A	NM_001040142.1	C	A/C	intron	none	NA	NA
A9	2	167055089	SCN9A	NM_002977.3	A	A/G	utr-3	none	NA	NA

A9	4	141264764	SCOC	NM_001153446.1	C	C/G	intron	none	NA	NA	
A9	17	26982258	SDF2	NM_006923.3	C	C/T	intron	none	NA	NA	
A9	7	4172003	SDK1	NM_001079653.1	C	C/T	utr-5	none	NA	NA	
A9	17	71419681	SDK2	NM_001144952.1	T	G/T	intron	none	NA	NA	
A9	16	5050728	SEC14L5	NM_014692.1	A	A/G	missense	GLU,GLY	348/697		1043
A9	1	177930717	SEC16B	NM_033127.2	G	C/G	intron	none	NA	NA	
A9	4	83750244	SEC31A	NM_001077206.2	G	C/G	intron	none	NA	NA	
A9	4	83750312	SEC31A	NM_001077206.2	G	A/G	intron	none	NA	NA	
A9	9	91972954	SECISBP2	NM_024077.3	G	A/G	missense	ARG,GLN	770/855		2309
A9	20	13856607	SEL1L2	NM_025229.1	C	C/T	intron	none	NA	NA	
A9	11	94918660	SESN3	NM_144665.2	A	A/T	intron	none	NA	NA	
A9	14	99876660	SETD3	NM_032233.2	G	A/G	intron	none	NA	NA	
A9	3	9512264	SETD5	NM_001080517.1	C	C/T	missense	SER,PHE	949/1443		2846
A9	10	7213051	SFMBT2	NM_001018039.1	G	A/G	intron	none	NA	NA	
A9	10	7262355	SFMBT2	NM_001018039.1	C	C/T	intron	none	NA	NA	
A9	10	81319415	SFTPA2	NM_001098668.2	A	A/C	intron	none	NA	NA	
A9	1	162381697	SH2D1B	NM_053282.4	G	A/G	missense	PRO,LEU	37/133		110
A9	8	19221642	SH2D4A	NM_001174159.1	G	C/G	missense	ASP,HIS	256/455		766
A9	10	105526886	SH3PXD2A	NM_014631.2	C	A/C	missense	LYS,ASN	65/1106		195
A9	13	46357825	SIAH3	NM_198849.2	A	A/T	missense	LEU,GLN	168/270		503
A9	3	113303847	SIDT1	NM_017699.2	A	A/T	intron	none	NA	NA	
A9	4	147204281	SLC10A7	NM_001029998.3	C	C/G	intron	none	NA	NA	
A9	15	48593517	SLC12A1	NM_000338.2	C	C/T	coding-synonymous	none	1034/1100		3102
A9	20	44669411	SLC12A5	NM_001134771.1	G	C/G	intron	none	NA	NA	
A9	3	124867794	SLC12A8	NM_001195483.1	G	C/G	intron	none	NA	NA	
A9	8	20036790	SLC18A1	NM_001135691.2	G	C/G	missense	ILE,MET	110/526		330
A9	11	64368317	SLC22A12	NM_144585.2	G	C/G	missense	GLY,ALA	502/554		1505
A9	11	62747115	SLC22A6	NM_004790.4	A	A/G	intron	none	NA	NA	
A9	14	92790446	SLC24A4	NM_153646.3	G	C/G	intron	none	NA	NA	
A9	8	118174182	SLC30A8	NM_001172811.1	A	A/C	intron	none	NA	NA	
A9	14	58056077	SLC35F4	NM_001206920.1	A	A/T	coding-synonymous	none	147/485		441
A9	14	58060857	SLC35F4	NM_001206920.1	T	G/T	intron	none	NA	NA	
A9	21	43987129	SLC37A1	NM_018964.3	G	A/G	coding-synonymous	none	435/534		1305
A9	7	140045774	SLC37A3	NM_032295.2	C	C/G	intron	none	NA	NA	
A9	10	18242165	SLC39A12	NM_001145195.1	A	A/T	utr-5	none	NA	NA	
A9	10	18276549	SLC39A12	NM_001145195.1	C	C/G	missense	SER,CYS	413/692		1238
A9	3	11062045	SLC6A1	NM_003042.3	G	G/T	intron	none	NA	NA	
A9	1	110716715	SLC6A17	NM_001010898.2	G	A/G	missense	VAL,MET	189/728		565
A9	5	1411303	SLC6A3	NM_001044.4	C	C/T	intron	none	NA	NA	
A9	17	28538318	SLC6A4	NM_001045.4	C	A/C	intron	none	NA	NA	
A9	11	20636185	SLC6A5	NM_004211.3	C	C/T	intron	none	NA	NA	
A9	5	149584500	SLC6A7	NM_014228.3	G	A/G	intron	none	NA	NA	
A9	19	33359435	SLC7A9	NM_001126335.1	C	A/C	coding-synonymous	none	2/488		6

A9	3	143387856	SLC9A9	NM_173653.3	T	A/T	intron	none	NA	NA	
A9	4	20512126	SLIT2	NM_004787.1	A	A/T	missense	GLU,VAL	308/1530		923
A9	13	86368172	SLITRK6	NM_032229.2	T	C/T	coding-synonymous	none	824/842		2472
A9	10	105770723	SLK	NM_014720.2	G	A/G	intron	none	NA	NA	
A9	4	95197529	SMARCAD1	NM_001128429.2	G	G/T	coding-synonymous	none	616/1029		1848
A9	3	47702964	SMARCC1	NM_003074.3	T	C/T	intron	none	NA	NA	
A9	3	47727623	SMARCC1	NM_003074.3	G	A/G	missense	SER,LEU	434/1106		1301
A9	14	62234117	SNAPC1	NM_003082.3	C	C/T	intron	none	NA	NA	
A9	2	70122200	SNRNP27	NM_006857.2	C	C/T	intron	none	NA	NA	
A9	2	1251254	SNTG2	NM_018968.3	G	C/G	intron	none	NA	NA	
A9	18	67992556	SOCS6	NM_004232.3	C	C/G	missense	GLN,GLU	218/536		652
A9	10	106918730	SORCS3	NM_014978.1	C	A/C	coding-synonymous	none	570/1223		1710
A9	2	39294861	SOS1	NM_005633.3	T	C/T	missense	ASN,ASP	41/1334		121
A9	22	38369684	SOX10	NM_006941.3	G	A/G	missense	PRO,SER	407/467		1219
A9	1	118548234	SPAG17	NM_206996.2	G	G/T	intron	none	NA	NA	
A9	6	44337656	SPATS1	NM_145026.3	T	G/T	intron	none	NA	NA	
A9	1	231488744	SPRTN	NM_001010984.2	G	C/G	utr-3	none	NA	NA	
A9	1	158615098	SPTA1	NM_003126.2	A	A/G	coding-synonymous	none	1358/2420		4074
A9	15	42165874	SPTBN5	NM_016642.2	C	C/T	intron	none	NA	NA	
A9	15	42180179	SPTBN5	NM_016642.2	G	A/G	coding-synonymous	none	167/3640		501
A9	19	56001242	SSC5D	NM_001144950.1	G	A/G	intron	none	NA	NA	
A9	7	149476927	SSPO	NM_198455.2	T	A/T	intron	none	NA	NA	
A9	7	99787030	STAG3	NM_012447.2	C	C/T	intron	none	NA	NA	
A9	7	99808604	STAG3	NM_012447.2	C	A/C	intron	none	NA	NA	
A9	X	7223016	STS	NM_000351.4	G	T/T	intron	none	NA	NA	
A9	11	125490106	STT3A	NM_152713.3	C	C/G	intron	none	NA	NA	
A9	3	121138123	STXBP5L	NM_014980.2	C	A/C	utr-3	none	NA	NA	
A9	1	172526580	SUCO	NM_014283.3	C	C/T	intron	none	NA	NA	
A9	20	46305982	SULF2	NM_001161841.1	G	A/G	intron	none	NA	NA	
A9	17	73164374	SUMO2	NM_001005849.1	G	C/G	utr-3	none	NA	NA	
A9	15	91832745	SV2B	NM_001167580.1	C	C/G	intron	none	NA	NA	
A9	10	29775560	SVIL	NM_003174.3	G	C/G	intron	none	NA	NA	
A9	20	58475079	SYCP2	NM_014258.2	T	A/T	intron	none	NA	NA	
A9	14	64628964	SYNE2	NM_015180.4	G	A/G	coding-synonymous	none	5423/6886		16269
A9	18	40853748	SYT4	NM_020783.3	C	C/G	missense	ASP,HIS	216/426		646
A9	6	132910074	TAAR5	NM_003967.2	G	G/T	missense	ALA,ASP	251/338		752
A9	17	34163131	TAF15	NM_003487.3	C	C/G	intron	none	NA	NA	
A9	10	8006780	TAF3	NM_031923.2	C	C/T	missense	ALA,VAL	436/930		1307
A9	17	61472996	TANC2	NM_025185.3	G	C/G	intron	none	NA	NA	
A9	17	61473358	TANC2	NM_025185.3	G	C/G	intron	none	NA	NA	
A9	5	9630127	TAS2R1	NM_019599.2	G	C/G	coding-synonymous	none	6/300		18
A9	17	15641943	TBC1D26	NM_178571.4	C	C/T	intron	none	NA	NA	
A9	17	18541378	TBC1D28	NM_001039397.2	G	A/G	intron	none	NA	NA	

A9	4	107168448	TBCK	NM_001163435.1	G	C/G	intron	none	NA	NA	
A9	7	45148652	TBRG4	NM_001261834.1	C	C/T	missense	ARG,GLN	73/643		218
A9	11	67400341	TBX10	NM_005995.4	G	G/T	intron	none	NA	NA	
A9	17	59544833	TBX4	NM_018488.2	C	A/C	intron	none	NA	NA	
A9	18	53128207	TCF4	NM_001083962.1	T	G/T	intron	none	NA	NA	
A9	5	149747530	TCOF1	NM_000356.3	G	A/G	intron	none	NA	NA	
A9	1	179609705	TDRD5	NM_001199085.1	T	C/T	intron	none	NA	NA	
A9	4	65165647	TECRL	NM_001010874.4	A	A/T	intron	none	NA	NA	
A9	10	114057878	TECTB	NM_058222.1	G	C/G	missense	ARG,SER	241/330		723
A9	17	56676958	TEX14	NM_001201457.1	T	A/T	missense	ASP,VAL	589/1498		1766
A9	6	10400692	TFAP2A	NM_001032280.2	G	A/G	coding-synonymous	none	332/432		996
A9	2	43732662	THADA	NM_001083953.1	C	C/T	intron	none	NA	NA	
A9	21	32639084	TIAM1	NM_003253.2	C	C/T	missense	ALA,THR	69/1592		205
A9	6	54216244	TINAG	NM_014464.3	C	C/G	intron	none	NA	NA	
A9	6	54216252	TINAG	NM_014464.3	C	C/T	intron	none	NA	NA	
A9	15	70349912	TLE3	NM_001105192.1	G	A/G	coding-synonymous	none	379/770		1137
A9	15	63029028	TLN2	NM_015059.2	G	G/T	intron	none	NA	NA	
A9	11	64879521	TM7SF2	NM_003273.2	G	C/G	missense	GLU,GLN	12/419		34
A9	17	72815748	TMEM104	NM_017728.3	G	A/G	intron	none	NA	NA	
A9	12	129180632	TMEM132C	NM_001136103.2	G	G/T	missense	GLY,VAL	638/1109		1913
A9	1	54509169	TMEM59	NM_004872.3	T	A/T	coding-synonymous	none	140/324		420
A9	21	43795945	TMPRSS3	NM_001256317.1	C	C/G	missense	GLU,ASP	408/454		1224
A9	8	119945261	TNFRSF11B	NM_002546.3	G	A/G	coding-synonymous	none	103/402		309
A9	6	47277197	TNFRSF21	NM_014452.3	G	A/G	coding-synonymous	none	17/656		51
A9	6	47277252	TNFRSF21	NM_014452.3	G	C/G	utr-5	none	NA	NA	
A9	1	74701202	TNNI3K	NM_001112808.2	C	C/T	intron	none	NA	NA	
A9	1	74833622	TNNI3K	NM_001112808.2	T	A/T	stop-gained	LEU,stop	533/937		1598
A9	16	24818153	TNRC6A	NM_014494.2	A	A/T	intron	none	NA	NA	
A9	7	47344505	TNS3	NM_022748.11	G	A/G	missense	SER,LEU	916/1446		2747
A9	22	41832290	TOB2	NM_016272.3	C	C/T	utr-3	none	NA	NA	
A9	3	133368168	TOPBP1	NM_007027.3	C	C/T	intron	none	NA	NA	
A9	8	95944207	TP53INP1	NM_001135733.1	G	C/G	intron	none	NA	NA	
A9	1	154140441	TPM3	NM_001043351.1	T	C/T	intron	none	NA	NA	
A9	2	1467719	TPO	NM_000547.5	A	A/G	intron	none	NA	NA	
A9	1	186312634	TPR	NM_003292.2	T	C/T	intron	none	NA	NA	
A9	13	19998947	TPTE2	NM_001141968.1	G	C/G	intron	none	NA	NA	
A9	13	20038448	TPTE2	NM_001141968.1	T	C/T	intron	none	NA	NA	
A9	4	118006173	TRAM1L1	NM_152402.2	C	C/T	missense	SER,ASN	126/370		377
A9	4	184614951	TRAPPC11	NM_021942.5	A	A/G	intron	none	NA	NA	
A9	6	123576273	TRDN	NM_006073.3	G	G/T	intron	none	NA	NA	
A9	12	72893507	TRHDE	NM_013381.2	C	A/C	intron	none	NA	NA	
A9	5	180687446	TRIM52	NM_032765.2	C	C/G	missense	GLU,ASP	123/298		369
A9	4	189026137	TRIML2	NM_173553.1	G	A/G	intron	none	NA	NA	

A9		5	14271085	TRIO	NM_007118.2	A	A/C	intron	none	NA	NA	
A9		5	14398903	TRIO	NM_007118.2	T	A/T	intron	none	NA	NA	
A9	X		100293025	TRMT2B	NM_001167970.1	G	C/C	missense	GLN,GLU	107/505		319
A9		12	49723737	TROAP	NM_005480.3	C	C/G	missense	SER,CYS	421/779		1262
A9		4	122828685	TRPC3	NM_001130698.1	T	C/T	missense	ILE,MET	610/922		1830
A9		15	31330261	TRPM1	NM_001252020.1	C	C/T	missense	GLU,LYS	826/1643		2476
A9		7	98540683	TRRAP	NM_001244580.1	G	G/T	intron	none	NA	NA	
A9		11	18548342	TSG101	NM_006292.3	G	A/G	missense	LEU,PHE	8/391		22
A9		1	231700631	TSNAX	NM_005999.2	C	C/G	missense	GLN,GLU	285/291		853
A9		21	46057525	TSPEAR	NM_144991.2	C	C/G	intron	none	NA	NA	
A9		21	46011324	TSPEAR	NM_144991.2	T	C/T	intron	none	NA	NA	
A9		1	231076299	TTC13	NM_001122835.2	G	A/G	coding-synonymous	none	173/807		517
A9		3	180322033	TTC14	NM_001042601.2	T	G/T	missense	ASP,GLU	169/440		507
A9		9	135261986	TTF1	NM_001205296.1	C	C/T	missense	ASP,ASN	264/391		790
A9		20	36641926	TTI1	NM_014657.1	G	C/G	stop-gained	SER,stop	98/1090		293
A9		1	1114661	TTLL10	NM_001130045.1	C	C/T	coding-synonymous	none	22/674		66
A9		20	30496510	TTLL9	NM_001008409.2	G	A/G	intron	none	NA	NA	
A9		2	179634728	TTN	NM_001256850.1	A	A/G	intron	none	NA	NA	
A9		6	3225580	TUBB2B	NM_178012.4	G	A/G	missense	ALA,VAL	248/446		743
A9		15	70991880	UACA	NM_001008224.1	C	A/C	coding-synonymous	none	53/1404		159
A9		3	23258994	UBE2E2	NM_152653.3	T	C/T	intron	none	NA	NA	
A9		1	10231222	UBE4B	NM_001105562.2	G	A/G	missense	MET,ILE	1120/1303		3360
A9		1	19482048	UBR4	NM_020765.2	T	C/T	missense	ILE,VAL	2063/5184		6187
A9		8	30620972	UBXN8	NM_005671.2	C	A/C	intron	none	NA	NA	
A9		6	34834953	UHRF1BP1	NM_017754.3	G	C/G	intron	none	NA	NA	
A9		6	34835069	UHRF1BP1	NM_017754.3	G	G/T	coding-synonymous	none	1160/1441		3480
A9		21	43510329	UMODL1	NM_001004416.2	G	A/G	intron	none	NA	NA	
A9		15	54817945	UNC13C	NM_001080534.1	G	A/G	intron	none	NA	NA	
A9		17	33482570	UNC45B	NM_001033576.1	G	C/G	intron	none	NA	NA	
A9		5	176301097	UNC5A	NM_133369.2	G	G/T	missense	ALA,SER	339/843		1015
A9		14	94039221	UNC79	NM_020818.3	A	A/G	intron	none	NA	NA	
A9		14	94128846	UNC79	NM_020818.3	T	A/T	intron	none	NA	NA	
A9		13	115048453	UPF3A	NM_023011.3	C	C/G	intron	none	NA	NA	
A9		1	215960153	USH2A	NM_206933.2	A	A/C	missense	CYS,GLY	3416/5203		10246
A9		1	55562348	USP24	NM_015306.2	C	C/T	intron	none	NA	NA	
A9		17	5073990	USP6	NM_004505.2	G	C/G	missense	ARG,PRO	1245/1407		3734
A9		8	42262361	VDAC3	NM_001135694.2	T	G/T	intron	none	NA	NA	
A9		4	177608701	VEGFC	NM_005429.2	C	A/C	intron	none	NA	NA	
A9		6	133015373	VNN1	NM_004666.2	T	A/T	intron	none	NA	NA	
A9		8	100589291	VPS13B	NM_017890.4	C	C/T	intron	none	NA	NA	
A9		20	2840398	VPS16	NM_022575.2	G	A/G	coding-synonymous	none	29/840		87
A9		13	53000977	VPS36	NM_016075.2	G	A/G	intron	none	NA	NA	
A9		15	42455630	VPS39	NM_015289.2	G	G/T	intron	none	NA	NA	

A9	7	38781666	VPS41	NM_014396.3	C	A/C	coding-synonymous	none	726/855		2178
A9	10	50294091	VSTM4	NM_001031746.3	C	A/C	intron	none	NA	NA	
A9	7	12391270	VWDE	NM_001135924.1	T	A/T	missense	ASN,ILE	1272/1591		3815
A9	7	12417372	VWDE	NM_001135924.1	T	C/T	intron	none	NA	NA	
A9	4	85707092	WDFY3	NM_014991.4	G	A/G	intron	none	NA	NA	
A9	10	50121585	WDFY4	NM_001006939.3	G	C/G	missense	LEU,VAL	206/262		616
A9	14	55448137	WDHD1	NM_001008396.2	G	G/T	intron	none	NA	NA	
A9	10	122648679	WDR11	NM_018117.11	G	C/G	missense	LEU,PHE	737/1225		2211
A9	2	203745573	WDR12	NM_018256.3	T	C/T	utr-3	none	NA	NA	
A9	1	224585900	WDR26	NM_001115113.2	C	C/T	missense	ARG,LYS	542/646		1625
A9	1	224592243	WDR26	NM_001115113.2	T	C/T	coding-synonymous	none	380/646		1140
A9	2	20135955	WDR35	NM_001006657.1	G	C/G	intron	none	NA	NA	
A9	2	20136033	WDR35	NM_001006657.1	G	G/T	coding-synonymous	none	798/1182		2394
A9	3	167272709	WDR49	NM_178824.3	C	A/C	intron	none	NA	NA	
A9	3	113023862	WDR52	NM_001164496.1	C	A/C	intron	none	NA	NA	
A9	3	122134057	WDR5B	NM_019069.3	G	C/G	missense	LEU,VAL	107/331		319
A9	15	78587978	WDR61	NM_025234.1	G	C/G	intron	none	NA	NA	
A9	4	184129164	WWC2	NM_024949.5	C	C/G	coding-synonymous	none	100/1193		300
A9	13	21375045	XPO4	NM_022459.4	C	C/G	missense	MET,ILE	634/1152		1902
A9	1	180724066	XPR1	NM_001135669.1	C	C/T	intron	none	NA	NA	
A9	3	142037481	XRN1	NM_001042604.1	G	C/G	stop-gained	SER,stop	1524/1694		4571
A9	8	64087908	YTHDF3	NM_152758.4	C	C/T	missense	SER,LEU	23/586		68
A9	8	64087916	YTHDF3	NM_152758.4	C	C/T	stop-gained	GLN,stop	26/586		76
A9	3	167068190	ZBBX	NM_001199201.1	C	C/T	intron	none	NA	NA	
A9	6	109798055	ZBTB24	NM_014797.2	T	A/T	missense	HIS,LEU	344/698		1031
A9	19	47597953	ZC3H4	NM_015168.1	G	A/G	intron	none	NA	NA	
A9	1	31821726	ZCCHC17	NM_016505.2	C	C/G	missense	LEU,VAL	157/242		469
A9	3	28533650	ZCWPW2	NM_001040432.1	C	C/T	coding-synonymous	none	215/357		643
A9	3	28533664	ZCWPW2	NM_001040432.1	G	A/G	coding-synonymous-	none	219/357		657
A9	3	28533673	ZCWPW2	NM_001040432.1	G	G/T	intron	none	NA	NA	
A9	X	128940276	ZDHHC9	NM_001008222.2	G	A/A	utr-3	none	NA	NA	
A9	8	77761689	ZFHX4	NM_024721.4	A	A/G	intron	none	NA	NA	
A9	8	40554717	ZMAT4	NM_001135731.1	G	G/T	intron	none	NA	NA	
A9	11	6964464	ZNF215	NM_013250.2	A	A/C	intron	none	NA	NA	
A9	7	111967960	ZNF277	NM_021994.2	A	A/T	intron	none	NA	NA	
A9	19	44891217	ZNF285	NM_152354.3	T	C/T	missense	GLU,GLY	397/591		1190
A9	19	44891242	ZNF285	NM_152354.3	C	C/T	missense	VAL,ILE	389/591		1165
A9	6	87966952	ZNF292	NM_015021.1	C	C/T	missense	PRO,LEU	1202/2724		3605
A9	7	99669838	ZNF3	NM_017715.2	G	G/T	intron	none	NA	NA	
A9	7	148875650	ZNF398	NM_020781.3	C	C/T	intron	none	NA	NA	
A9	7	57193934	ZNF479	NM_033273.1	T	C/T	intron	none	NA	NA	
A9	19	22836890	ZNF492	NM_020855.2	G	G/T	intron	none	NA	NA	
A9	8	125987957	ZNF572	NM_152412.2	C	C/T	coding-synonymous	none	25/530		75

A9	19	52519489	ZNF614	NM_025040.3	G	A/G	coding-synonymous	none	454/586	1362
A9	19	52519513	ZNF614	NM_025040.3	C	C/G	missense	GLN,HIS	446/586	1338
A9	8	144378641	ZNF696	NM_030895.2	G	A/G	missense	GLU,LYS	266/375	796
A9	7	63505884	ZNF727	NM_001159522.1	C	C/T	utr-5	none	NA	NA
A9	7	88962840	ZNF804B	NM_181646.2	C	C/T	missense	ARG,TRP	182/1350	544
A9	20	57768918	ZNF831	NM_178457.1	A	A/G	coding-synonymous	none	948/1678	2844
A9	19	22575457	ZNF98	NM_001098626.1	G	G/T	missense	LEU,ILE	194/573	580
A9	1	71537845	ZRANB2	NM_005455.4	C	C/G	missense	GLU,GLN	158/321	472

Supplementary Table 4. All highly deleterious mutations

Sample	Chromosome	Position	Gene Symbol	Accession	ReferenceBase	SampleAlleles	FunctionGVS	AminoAcids	ProteinPosition	cDNAPosition	PolyPhen	GranthamScore	ScorePhastCons	ScoreGERP	Support
A9	4	164506969	40969	NM_001166373.1	C	A/C	missense	ASP,TYR	119/290	355	0.999	160	0.995	5.57	4
A7	12	58150783	40977	NM_138396.5	G	C/G	missense	TRP,CYS	143/347	429	1	215	1	5.01	4
A3	16	70310868	AARS	NM_001605.2	C	A/C	splice-5	none	NA	NA	unknown	NA	1	5.32	3
A1	17	79101635	AATK	NM_001080395.2	C	C/T	missense	VAL,MET	164/1375	490	1	21	0.984	3.48	3
A9	17	67246120	ABCA5	NM_018672.3	G	C/G	missense	SER,CYS	1564/1643	4691	0.99	112	1	4.52	4
A9	2	169869830	ABCB11	NM_003742.2	C	C/T	stop-gained	TRP,stop	114/1322	341	unknown	NA	0.881	5.41	3
A9	2	169869830	ABCB11	NM_003742.2	C	C/T	stop-gained	TRP,stop	114/1322	341	unknown	NA	0.881	5.41	3
A9	7	87092073	ABCB4	NM_000443.3	C	C/T	splice-5	none	NA	NA	unknown	NA	1	5.45	3
A7	11	17418566	ABCC8	NM_000352.3	G	G/T	missense	PRO,GLN	1339/1582	4016	1	76	1	4.62	3
A1	X	153008788	ABCD1	NM_000033.3	G	A/G	missense	ARG,GLN	660/746	1979	1	43	0.994	4.69	3
A6	3	111700646	ABHD10	NM_018394.2	C	C/T	missense	THR,MET	53/307	158	0.958	81	0.939	5.39	3
A3	1	179077016	ABL2	NM_001136000.2	T	G/T	missense	GLN,PRO	1011/1065	3032	0.971	76	0.956	5.65	3
A9	18	47318671	ACAA2	NM_006111.2	C	A/C	stop-gained	GLU,stop	202/398	604	unknown	NA	0.908	5.49	4
A6	20	33508924	ACSS2	NM_001076552.2	G	G/T	missense	GLY,VAL	433/715	1298	1	109	1	5.53	4
A6	1	236900503	ACTN2	NM_001103.2	C	A/C	missense	LEU,ILE	289/895	865	0.944	5	1	5.86	3
A7	5	7626398	ADCY2	NM_020546.2	G	G/T	missense	ARG,LEU	230/1092	689	0.965	102	1	5.09	4
A7	7	150839002	AGAP3	NM_031946.4	G	A/G	missense	GLU,LYS	608/912	1822	0.999	56	0.929	4.24	3
A9	14	105404821	AHNAK2	NM_138420.2	G	C/G	missense	SER,CYS	5656/5796	16967	0.998	112	0.155	5.38	3
A3	1	77987614	AK5	NM_012093.3	G	C/G	missense	GLU,GLN	446/537	1336	0.948	29	1	4.76	3
A1	11	67786318	ALDH3B1	NM_000694.2	C	C/T	missense	ARG,CYS	117/468	349	1	180	0.744	3.75	3
A9	11	46563784	AMBRA1	NM_001267782.1	C	C/T	missense	GLU,LYS	505/1302	1513	0.991	56	0.997	5.73	3
A5	12	47472266	AMIGO2	NM_001143668.1	C	C/T	missense	GLY,ARG	174/523	520	0.003	125	0.997	3	3
A9	5	79854576	ANKRD34B	NM_001004441.2	C	C/G	missense	ARG,SER	421/515	1263	0.999	110	0.99	5.18	4
A3	12	5939653	ANO2	NM_020373.2	T	C/T	missense	TYR,CYS	259/999	776	1	194	1	5.52	4
A7	10	81917448	ANXA11	NM_001157.2	G	C/G	missense	ILE,MET	469/506	1407	1	10	1	5.3	3
A9	15	29346418	APBA2	NM_001130414.1	C	A/C	missense	LEU,ILE	111/738	331	0.999	5	1	4.19	3
A3	5	115319073	AQPEP	NM_173800.4	T	A/T	missense	ILE,ASN	262/991	785	0.997	149	0.993	5.54	4
A6	20	47587788	ARFGEF2	NM_006420.2	C	C/T	missense	SER,PHE	441/1786	1322	0.921	155	0.997	4.85	4
A4	16	18806845	ARL6IP1	NM_015161.1	G	A/G	missense	ARG,CYS	117/204	349	1	180	1	5.31	4
A9	3	137982972	ARMC8	NM_001267041.1	G	C/G	splice-3	none	NA	NA	unknown	NA	1	5.61	3
A2	3	137991808	ARMC8	NM_001267041.1	G	A/G	splice-3	none	NA	NA	unknown	NA	1	5.39	3
A6	4	177190130	ASB5	NM_080874.3	C	C/T	missense	VAL,MET	44/330	130	0.999	21	0.972	5.84	3
A9	6	101075526	ASCC3	NM_006828.2	C	A/C	missense	GLY,CYS	1528/2203	4582	1	159	0.986	5.67	4
A9	3	130737380	ASTE1	NM_014065.2	C	A/C	missense	GLY,TRP	495/680	1483	1	184	0.441	5.71	3
A6	9	119976798	ASTN2	NM_014010.4	C	C/G	missense	ARG,PRO	285/1289	854	1	103	1	5.34	4
A7	2	25966031	ASXL2	NM_018263.4	T	G/T	missense	SER,ARG	1059/1436	3175	0.664	110	0.972	3.72	3
A7	7	117062360	ASZ1	NM_130768.2	C	C/T	missense	ALA,THR	87/476	259	0.998	58	1	5.64	3
A5	11	108160428	ATM	NM_000051.3	G	G/T	missense	VAL,PHE	1446/3057	4336	1	50	1	5.45	3
A9	3	113507643	ATP6V1A	NM_001690.3	C	C/G	missense	SER,CYS	267/618	800	1	112	1	5.61	4
A3	2	71187100	ATP6V1B1	NM_001692.3	C	C/G	stop-gained	TYR,stop	159/514	477	unknown	NA	1	4.47	4
A3	3	142180838	ATR	NM_001184.3	C	A/C	missense	TRP,LEU	2379/2645	7136	1	61	1	5.34	3
A9	X	76872153	ATRX	NM_000489.3	C	T/T	missense	GLU,LYS	1832/2493	5494	1	56	1	5.77	3
A3	12	74931999	ATXN7L3B	NM_001136262.1	A	A/C	missense	GLU,ALA	36/98	107	0.95	107	1	4.23	4
A9	12	58197142	AVIL	NM_006576.3	C	A/C	missense	ARG,LEU	617/820	1850	0.999	102	0.685	4.24	3
A8	19	41931678	B3GNT8	NM_198540.2	A	A/T	missense	TYR,ASN	336/398	1006	1	143	1	3.81	4
A3	20	48256228	B4GALT5	NM_004776.3	C	A/C	missense	ASP,TYR	302/389	904	1	160	0.996	5.41	4
A1	20	48273127	B4GALT5	NM_004776.3	C	C/G	missense	ARG,SER	76/389	228	0.397	110	1	4.78	3
A9	11	117162523	BACE1	NM_001207048.1	A	A/G	missense	TYR,HIS	183/402	547	0.988	83	1	6.03	3
A6	17	79073786	BAIAP2	NM_001144888.1	A	A/G	missense	TYR,CYS	183/535	548	1	194	1	5.27	4
A9	4	122766847	BBS7	NM_018190.3	C	C/T	missense	GLU,LYS	348/673	1042	0.991	56	0.994	5.69	3
A3	4	122747026	BBS7	NM_176824.2	C	A/C	missense	ASP,TYR	713/716	2137	0.977	160	1	5.81	4
A3	3	107492079	BBX	NM_001142568.1	G	G/T	missense	GLY,VAL	504/942	1511	1	109	1	5.01	4
A7	19	41916597	BCKDHA	NM_000709.3	C	C/G	missense	PRO,ARG	55/446	164	0.999	103	1	5.42	4
A3	20	61869263	BIRC7	NM_022161.2	G	A/G	missense	ASP,ASN	120/281	358	1	23	0.996	4.96	3

A9	10	93702311	BTAF1	NM_003972.2	A	A/G	missense	GLN,ARG	129/1850		386	0.941	43	1	5.71	3
A5	11	57513407	BTBD18	NM_001145101.1	C	C/T	missense	ARG,HIS	113/713		338	0.997	29	1	5.19	3
A9	11	122830117	C11orf63	NM_024806.2	G	G/T	missense	GLN,HIS	767/779		2301	1	24	1	3.01	3
A9	12	53694018	C12orf10	NM_021640.3	C	C/T	missense	ARG,TRP	101/377		301	0.952	101	0.997	2.33	4
A7	12	40040152	C12orf40	NM_001031748.2	A	A/G	missense	HIS,ARG	75/653		224	1	29	0.995	4.13	3
A9	5	37231005	C5orf42	NM_023073.3	G	C/G	missense	ALA,GLY	362/3198		1085	1	60	1	5.32	3
A7	5	41904551	C5orf51	NM_175921.4	G	A/G	missense	GLU,LYS	28/295		82	0.977	56	1	4.67	3
A6	5	41181474	C6	NM_000065.2	G	G/T	missense	ALA,ASP	305/935		914	1	126	0.837	6.06	3
A3	2	27461420	CAD	NM_004341.3	G	A/G	missense	ARG,GLN	1661/2226		4982	0.982	43	1	4.94	3
A7	3	62739345	CADPS	NM_003716.3	C	C/T	missense	ARG,HIS	220/1354		659	1	29	1	5.88	3
A3	11	15098867	CALCB	NM_000728.3	C	A/C	missense	THR,ASN	87/128		260	1	65	1	4.87	3
A3	3	49898284	CAMKV	NM_024046.3	G	G/T	missense-nea	LEU,ILE	214/502		640	0.999	5	1	5.51	3
A7	7	94139434	CASD1	NM_022900.4	T	A/T	missense	ILE,ASN	13/798		38	0.984	149	1	2.96	4
A6	7	107399152	CBLL1	NM_024814.2	G	G/T	missense	MET,ILE	335/492		1005	0.908	10	1	4.47	3
A7	11	124845037	CCDC15	NM_025004.2	A	A/G	missense	LYS,GLU	188/952		562	1	56	1	5.26	3
A6	4	77303868	CCDC158	NM_001042784.1	T	A/T	missense	GLU,VAL	270/1114		809	1	121	1	5.18	4
A3	2	26652583	CCDC164	NM_145038.2	C	C/T	stop-gained	GLN,stop	210/741		628	unknown	NA	0.431	5.35	3
A3	2	26652583	CCDC164	NM_145038.2	C	C/T	stop-gained	GLN,stop	210/741		628	unknown	NA	0.431	5.35	3
A6	6	151859426	CCDC170	NM_025059.3	G	G/T	stop-gained	GLU,stop	145/716		433	unknown	NA	0.469	5.68	3
A6	6	151859426	CCDC170	NM_025059.3	G	G/T	stop-gained	GLU,stop	145/716		433	unknown	NA	0.469	5.68	3
A3	1	157803225	CD5L	NM_005894.2	C	A/C	missense	GLY,CYS	266/348		796	1	159	1	4.88	4
A7	17	62007248	CD79B	NM_000626.2	C	A/C	missense-nea	GLY,VAL	144/230		431	1	109	0.999	5.6	4
A2	1	227219196	CDC42BPA	NM_003607.3	G	A/G	missense	SER,LEU	1158/1720		3473	0.905	145	0.993	5.71	4
A3	2	37873324	CDC42EP3	NM_006449.3	C	A/C	missense	GLY,VAL	136/255		407	0.421	109	0.998	4.98	3
A9	6	44371631	CDC5L	NM_001253.3	G	G/T	missense	ASP,TYR	209/803		625	1	160	1	6.04	4
A9	5	24505272	CDH10	NM_006727.3	C	C/G	missense	ASP,HIS	448/789		1342	1	81	1	5.57	3
A6	5	24491727	CDH10	NM_006727.3	C	C/T	missense	GLY,ARG	612/789		1834	1	125	0.727	5.89	3
A9	5	24537735	CDH10	NM_006727.3	A	A/G	missense	SER,PRO	94/789		280	0.996	74	1	5.88	3
A9	18	59166674	CDH20	NM_031891.2	G	A/G	missense	GLY,ARG	168/802		502	1	125	0.995	6.06	4
A3	10	73572642	CDH23	NM_001171933.1	A	A/G	missense	ILE,VAL	970/1115		2908	0.913	29	1	6.08	3
A6	1	1575687	CDK11B	NM_033486.1	G	A/G	missense	SER,LEU	316/698		947	0.978	145	0.475	3.98	3
A9	12	96704829	CDK17	NM_001170464.2	C	C/T	splice-5	none	NA	NA		unknown	NA	1	5.62	3
A6	13	26911733	CDK8	NM_001260.1	A	A/C	missense	GLN,PRO	53/465		158	0.983	76	1	5.64	3
A6	9	21974675	CDKN2A	NM_000077.4	A	A/T	splice-5	none	NA	NA		unknown	NA	0.931	4.89	3
A3	16	80667016	CDYL2	NM_152342.2	C	C/T	missense	SER,ASN	245/507		734	1	46	1	3.91	3
A9	6	109471434	CEP57L1	NM_001083535.1	G	C/G	missense	GLU,GLN	152/461		454	0.984	29	1	3.47	3
A7	3	138290150	CEP70	NM_024491.2	C	C/T	missense	GLY,SER	40/598		118	1	56	0.983	5.25	3
A5	15	93557925	CHD2	NM_001271.3	G	A/G	splice-3	none	NA	NA		unknown	NA	1	5.76	3
A3	17	7802715	CHD3	NM_001005271.2	T	A/T	missense	TRP,ARG	859/2060		2575	1	101	1	5.06	4
A1	20	40102012	CHD6	NM_032221.3	C	C/T	missense	ASP,ASN	872/2716		2614	1	23	1	5.44	3
A3	1	203153729	CHI3L1	NM_001276.2	C	C/T	missense	GLY,ARG	97/384		289	1	125	0.997	5.64	4
A5	3	439979	CHL1	NM_001253387.1	C	C/T	missense	PRO,LEU	1039/1209		3116	0.988	98	1	5.72	3
A3	3	184102932	CHRD	NM_003741.2	G	G/T	missense	GLY,VAL	575/956		1724	0.987	109	1	5.29	4
A3	1	240071088	CHRM3	NM_000740.2	G	G/T	missense	ALA,SER	113/591		337	1	99	0.968	5.9	3
A9	15	78911129	CHRNA3	NM_000743.4	G	G/T	missense	LEU,MET	71/506		211	1	15	0.998	3.95	3
A7	10	125771915	CHST15	NM_015892.3	G	A/G	missense	ARG,CYS	477/562		1429	1	180	0.992	5.16	4
A6	15	101718311	CHSY1	NM_014918.4	T	C/T	missense	ASN,SER	564/803		1691	0.943	46	1	4.49	3
A6	16	70211149	CLEC18C	NM_173619.2	G	G/T	missense	TRP,CYS	74/447		222	0.997	215	1	4.32	4
A9	7	146805370	CNTNAP2	NM_014141.5	G	G/T	stop-gained	GLY,stop	228/1332		682	unknown	NA	0.424	6.03	3
A9	7	146805370	CNTNAP2	NM_014141.5	G	G/T	stop-gained	GLY,stop	228/1332		682	unknown	NA	0.424	6.03	3
A3	2	125204375	CNTNAP5	NM_130773.2	T	A/T	missense	LEU,GLN	260/1307		779	0.995	113	0.991	5.87	4
A6	1	86252107	COL24A1	NM_152890.5	C	C/G	missense	GLY,ALA	1330/1715		3989	1	60	0.902	5.25	3
A9	2	228147099	COL4A3	NM_000091.4	G	G/T	missense	GLY,VAL	836/1671		2507	1	109	0.427	5.87	3
A7	2	189904238	COL5A2	NM_000393.3	G	G/T	missense	LEU,ILE	1229/1500		3685	0.901	5	0.998	5.28	3
A2	21	47552150	COL6A2	NM_001849.3	C	C/T	missense	SER,LEU	915/1020		2744	1	145	0.999	4.18	4
A6	2	238274562	COL6A3	NM_004369.3	G	A/G	missense	HIS,TYR	1873/3178		5617	0.944	83	0.987	5.34	3

A6	3	130113888	COL6A5	NM_153264.5	G	A/G	missense	GLY,ARG	1050/2527		3148	0.996		125	0.996	4.75	4
A3	3	48630819	COL7A1	NM_000094.3	T	A/T	stop-gained	LYS,stop	164/2945		490	unknown	NA		0.999	4.51	4
A7	11	36300046	COMMD9	NM_001101653.1	T	C/T	missense	LYS,GLU	58/157		172	0.999		56	0.997	5.91	3
A3	12	54741818	COPZ1	NM_016057.1	C	C/T	missense	HIS,TYR	144/178		430	0.931		83	1	3.91	3
A9	16	57490554	COQ9	NM_020312.3	G	A/G	missense	ALA,THR	173/319		517	0.947		58	1	5.57	3
A9	7	120770206	CPED1	NM_001105533.1	C	C/T	stop-gained	GLN,stop	479/784		1435	unknown	NA		1	5.34	4
A6	8	3253752	CSMD1	NM_033225.5	T	A/T	missense	SER,CYS	853/3565		2557	1		112	1	5.07	4
A9	1	34003122	CSMD2	NM_052896.3	C	A/C	missense	CYS,PHE	3096/3488		9287	0.994		205	1	5.66	4
A3	8	113348909	CSMD3	NM_052900.2	C	A/C	stop-gained	GLU,stop	2227/3539		6679	unknown	NA		1	5.64	4
A5	8	114186041	CSMD3	NM_052900.2	C	C/G	missense	GLY,ARG	207/3539		619	1		125	1	5.1	4
A5	3	37988703	CTDSPL	NM_001008392.1	G	G/T	splice-5	none	NA	NA		unknown	NA		1	5.2	3
A9	2	204737466	CTLA4	NM_001037631.2	T	G/T	missense	CYS,GLY	165/175		493	0.934		159	1	4.56	4
A3	11	10789992	CTR9	NM_014633.3	C	C/T	missense	ALA,VAL	688/1174		2063	0.998		64	1	5.93	3
A9	5	134914208	CXCL14	NM_004887.4	C	C/T	missense	ARG,GLN	41/112		122	1		43	1	4.23	3
A9	3	45988374	CXCR6	NM_006564.1	C	C/T	missense	ALA,VAL	134/343		401	0.957		64	1	5.57	3
A6	X	85950042	DACH2	NM_001139514.1	G	T/T	missense	TRP,LEU	251/572		752	1		61	1	4.99	3
A3	11	61505190	DAGLA	NM_006133.2	G	A/G	missense	VAL,MET	516/1043		1546	1		21	0.989	4.11	3
A6	2	136681995	DARS	NM_001349.2	T	A/T	missense	LYS,ILE	213/502		638	0.33		102	1	4.69	3
A7	4	151142877	DCLK2	NM_001040260.3	A	A/T	missense	TYR,PHE	394/767		1181	1		22	1	6.17	3
A1	10	70715988	DDX21	NM_004728.3	G	A/G	missense	GLY,ARG	3/784		7	0.016		125	1	4.35	3
A4	17	61889373	DDX42	NM_007372.2	G	G/T	stop-gained	GLU,stop	494/939		1480	unknown	NA		0.995	5.7	4
A9	16	90030666	DEF8	NM_001242816.1	C	C/T	missense	SER,LEU	354/442		1061	0.934		145	0.94	5.37	4
A7	12	100657371	DEPDC4	NM_152317.2	G	A/G	missense	SER,PHE	153/295		458	0.76		155	0.984	3.12	3
A5	22	19127200	DGCR14	NM_022719.2	C	C/T	missense	ALA,THR	205/477		613	0.957		58	0.944	4.47	3
A6	20	37631438	DHX35	NM_001190809.1	C	A/C	stop-gained	SER,stop	229/673		686	unknown	NA		1	5.58	4
A9	2	39070165	DHX57	NM_198963.1	G	A/G	missense	LEU,PHE	803/1387		2407	0.995		22	1	5.75	3
A8	11	83674033	DLG2	NM_001142699.1	G	C/G	missense	SER,CYS	412/976		1235	0.999		112	1	5.53	4
A9	2	172967059	DLX2	NM_004405.3	G	C/G	missense	GLN,GLU	70/329		208	0.942		29	1	4.57	3
A6	19	46287514	DMWD	NM_004943.1	C	A/C	missense	GLY,VAL	671/675		2012	1		109	1	4.09	4
A7	5	13789001	DNAH5	NM_001369.2	T	A/T	missense	HIS,LEU	2824/4625		8471	1		99	0.998	6.06	3
A7	17	11532779	DNAH9	NM_001372.3	G	G/T	stop-gained	GLU,stop	466/4487		1396	unknown	NA		1	5.61	4
A9	17	11790283	DNAH9	NM_001372.3	G	C/G	splice-5	none	NA	NA		unknown	NA		1	5.22	3
A4	2	225761013	DOCK10	NM_014689.2	G	A/G	stop-gained-n	ARG,stop	139/2187		415	unknown	NA		0.996	2.33	3
A9	2	225637990	DOCK10	NM_014689.2	C	C/G	missense	GLU,GLN	2030/2187		6088	0.999		29	1	5.63	3
A6	19	38706881	DPF1	NM_001135155.1	C	C/G	missense	GLY,ALA	293/415		878	0.999		60	0.997	4.54	3
A9	11	117302445	DSCAML1	NM_020693.2	C	A/C	splice-3	none	NA	NA		unknown	NA		0.977	3.71	3
A5	11	117375686	DSCAML1	NM_020693.2	G	A/G	missense	SER,LEU	772/2114		2315	0.999		145	0.993	4.32	4
A3	11	103006448	DYNC2H1	NM_001080463.1	G	G/T	splice-3	none	NA	NA		unknown	NA		1	5.3	3
A9	2	71781013	DYSF	NM_001130455.1	C	A/C	missense	SER,ARG	670/2082		2010	0.021		110	0.999	4.01	3
A7	5	158204489	EBF1	NM_024007.3	A	A/G	missense	VAL,ALA	323/592		968	1		64	1	5.72	3
A9	13	78492268	EDNRB	NM_000115.3	G	G/T	missense	ASP,GLU	147/443		441	1		45	1	4.21	3
A3	8	144668390	EEF1D	NM_001130053.2	C	A/C	missense-nea	GLY,VAL	429/648		1286	0.999		109	0.983	4.64	4
A3	8	133015495	EFR3A	NM_015137.4	C	C/T	missense	HIS,TYR	775/822		2323	0.956		83	1	4.57	3
A9	5	38412703	EGFLAM	NM_001205301.1	G	G/T	missense	GLY,TRP	483/1018		1447	1		184	0.98	5.92	4
A8	7	55259515	EGFR	NM_005228.3	T	G/T	missense	LEU,ARG	858/1211		2573	1		102	1	5.71	4
A2	10	64575656	EGR2	NM_000399.3	C	C/T	missense	GLY,GLU	45/477		134	0.999		98	1	4.57	3
A6	3	150293456	EIF2A	NM_032025.3	G	C/G	missense	GLN,HIS	468/586		1404	0.999		24	1	3.77	3
A8	9	23705056	ELAVL2	NM_001171195.1	C	C/T	missense	ARG,HIS	116/347		347	0.953		29	1	5.71	3
A3	13	41507652	ELF1	NM_001145353.1	T	A/T	missense	GLN,LEU	566/596		1697	0.913		113	0.999	4.64	4
A9	20	45000260	ELMO2	NM_133171.3	C	A/C	stop-gained	GLU,stop	551/721		1651	unknown	NA		1	4.69	4
A3	18	2891552	EMILIN2	NM_032048.2	G	A/G	missense	ARG,GLN	476/1054		1427	0.978		43	0.945	5.4	3
A6	4	111397606	ENPEP	NM_001977.3	C	A/C	stop-gained	TYR,stop	12/958		36	unknown	NA		0.998	3.88	4
A4	8	120581493	ENPP2	NM_001040092.2	C	C/T	missense	GLY,ARG	679/864		2035	1		125	1	5.37	4
A9	8	110348433	ENY2	NM_001193557.1	G	G/T	missense-nea	ARG,LEU	23/97		68	0.912		102	1	5.49	4
A7	22	41574780	EP300	NM_001429.3	G	G/T	missense	GLN,HIS	2355/2415		7065	0.98		24	1	4.49	3
A3	3	89462359	EPHA3	NM_005233.5	G	G/T	stop-gained	GLU,stop	611/984		1831	unknown	NA		1	5.95	4

A9	3	55733440	ERC2	NM_015576.1	G	A/G	missense	SER,LEU	938/958		2813	0.382	145	1	5.66	3
A7	10	101911970	ERLIN1	NM_001100626.1	C	A/C	missense	GLY,VAL	322/349		965	0.023	109	1	3.61	3
A9	20	13750660	ESF1	NM_016649.3	G	G/T	missense	PRO,THR	471/852		1411	1	38	1	5.88	3
A9	14	64749417	ESR2	NM_001040275.1	G	A/G	missense	SER,LEU	96/496		287	0.969	145	0.811	5.56	3
A9	1	216824408	ESRRG	NM_001134285.2	C	A/C	missense	ALA,SER	143/436		427	0.997	99	0.99	5.74	3
A3	1	216741423	ESRRG	NM_001134285.2	C	A/C	missense	VAL,LEU	180/436		538	0.997	32	1	5.5	3
A6	7	13949287	ETV1	NM_001163147.1	C	C/G	missense	ASP,HIS	281/455		841	1	81	1	5.98	3
A1	5	453852	EXOC3	NM_007277.4	G	A/G	stop-gained	TRP,stop	244/746		732	unknown	NA	0.999	4.71	4
A8	8	28575429	EXTL3	NM_001440.2	C	C/T	missense	PRO,LEU	618/920		1853	0.998	98	0.988	6.04	3
A9	17	40856659	EZH1	NM_001991.3	C	C/T	missense	ASP,ASN	660/748		1978	1	23	1	5.4	3
A6	5	76028649	F2R	NM_001992.3	G	G/T	missense	ARG,LEU	200/426		599	1	102	0.992	5.07	4
A6	5	74137469	FAM169A	NM_015566.2	G	G/T	stop-gained	CYS,stop	11/671		33	unknown	NA	1	3.5	4
A3	5	74130407	FAM169A	NM_015566.2	C	A/C	stop-gained	GLU,stop	112/671		334	unknown	NA	1	4.99	4
A9	9	71998864	FAM189A2	NM_001127608.1	G	G/T	missense-nea	GLY,VAL	209/451		626	0.983	109	0.998	3.99	4
A9	4	159091679	FAM198B	NM_001031700.2	G	G/T	missense	ASP,GLU	283/528		849	1	45	1	3.64	3
A9	4	159048729	FAM198B	NM_001031700.2	G	A/G	missense	HIS,TYR	472/528		1414	0.986	83	1	5.67	3
A3	15	49868985	FAM227B	NM_152647.2	C	C/G	missense	ASP,HIS	167/509		499	0.997	81	1	3.86	3
A9	1	78268978	FAM73A	NM_001270384.1	C	C/T	stop-gained	GLN,stop	133/634		397	unknown	NA	1	4.72	4
A2	11	92620165	FAT3	NM_001008781.2	G	C/G	splice-3	none	NA	NA		unknown	NA	1	5.73	3
A9	4	126371698	FAT4	NM_024582.4	G	G/T	missense	GLY,VAL	3176/4982		9527	0.973	109	0.959	5.63	4
A3	3	13663411	FBLN2	NM_001004019.1	C	A/C	stop-gained	CYS,stop	764/1232		2292	unknown	NA	1	2.28	3
A3	5	127686559	FBN2	NM_001999.3	C	C/T	splice-5	none	NA	NA		unknown	NA	1	3.72	3
A6	5	135272567	FBXL21	NM_012159.4	C	C/T	missense	SER,LEU	95/435		284	0.999	145	0.985	4.07	4
A7	6	99322319	FBXL4	NM_012160.3	T	C/T	splice-3	none	NA	NA		unknown	NA	1	5.82	3
A9	2	97335910	FER1L5	NM_001113382.1	G	G/T	stop-gained	GLU,stop	468/2094		1402	unknown	NA	0.997	4.89	4
A3	3	14860589	FGD5	NM_152536.3	G	G/T	missense	GLY,VAL	4/1463		11	1	109	0.47	5.61	3
A7	12	95535254	FGD6	NM_018351.3	C	C/T	missense	ARG,GLN	916/1431		2747	1	43	1	5.79	3
A8	6	76024168	FILIP1	NM_015687.2	C	A/C	missense	LYS,ASN	460/1214		1380	0.995	94	1	3.85	3
A9	7	72745684	FKBP6	NM_001135211.1	C	C/T	stop-gained	GLN,stop	160/323		478	unknown	NA	1	3.2	4
A3	14	86089608	FLRT2	NM_013231.4	C	C/T	missense	ARG,TRP	584/661		1750	1	101	1	6.17	4
A7	20	14306457	FLRT3	NM_013281.3	T	A/T	missense	SER,CYS	566/650		1696	0.986	112	1	5.98	4
A9	1	240374482	FMN2	NM_020066.4	A	A/T	stop-gained	LYS,stop	1338/1723		4012	unknown	NA	1	4.8	4
A9	6	159670158	FNDC1	NM_032532.2	C	A/C	missense	ALA,ASP	1593/1895		4778	0.924	126	0.586	5.46	3
A7	14	29237222	FOXG1	NM_005249.4	A	A/T	missense	TYR,PHE	246/490		737	0.95	22	1	4	3
A6	13	39263675	FREM2	NM_207361.4	G	C/G	missense	ASP,HIS	732/3170		2194	1	81	0.998	5.97	3
A7	10	13708225	FRMD4A	NM_018027.3	A	A/C	missense	LEU,ARG	492/1040		1475	0.997	102	1	5.05	4
A9	13	32785076	FRY	NM_023037.2	A	A/G	missense	GLN,ARG	1499/3014		4496	0.943	43	1	5.57	3
A6	14	31084728	G2E3	NM_017769.3	A	A/T	missense	TYR,PHE	616/707		1847	1	22	0.914	5.69	3
A7	21	27134677	GABPA	NM_001197297.1	G	G/T	splice-5	none	NA	NA		unknown	NA	1	5.44	3
A3	15	26825465	GABRB3	NM_000814.5	C	A/C	splice-5	none	NA	NA		unknown	NA	1	5.35	3
A7	5	161530900	GABRG2	NM_000816.3	T	A/T	missense	TYR,ASN	213/468		637	1	143	0.997	5.56	4
A9	17	34071874	GAS2L2	NM_139285.3	C	C/G	stop-lost	stop,SER	881/881		2642	unknown	NA	0.994	1.9	3
A6	17	34074146	GAS2L2	NM_139285.3	G	G/T	missense	THR,LYS	325/881		974	0.994	78	1	4.13	3
A6	17	9821353	GAS7	NM_001130831.1	G	A/G	stop-gained	GLN,stop	364/413		1090	unknown	NA	0.995	5.08	4
A7	16	90106753	GAS8	NM_001481.2	A	A/T	missense	ILE,PHE	353/479		1057	0.999	21	1	3.57	3
A6	2	237074876	GBX2	NM_001485.2	G	C/G	missense	SER,CYS	243/349		728	0.985	112	1	4.42	4
A2	5	154305493	GEMIN5	NM_001252156.1	T	G/T	missense	ASN,HIS	407/1508		1219	0.999	68	1	6.17	3
A7	5	74028873	GFM2	NM_032380.3	C	A/C	missense	VAL,LEU	521/780		1561	0.995	32	1	5.07	3
A7	22	25023556	GGT1	NM_001032364.2	C	C/T	missense	ALA,VAL	393/570		1178	1	64	0.99	3.36	3
A7	9	36148586	GLIPR2	NM_022343.2	C	C/G	missense	SER,ARG	55/155		165	1	110	1	5.61	4
A3	6	49494382	GLYATL3	NM_001010904.1	C	C/T	stop-gained	GLN,stop	208/289		622	unknown	NA	1	6.06	4
A7	10	25886824	GPR158	NM_020752.2	C	C/T	missense	ARG,CYS	757/1216		2269	0.989	180	0.996	5.54	4
A2	17	36484002	GPR179	NM_001004334.2	A	A/C	missense	ILE,SER	1817/2368		5450	0.029	142	0.989	4.94	3
A7	6	110300638	GPR6	NM_005284.3	T	A/T	missense	PHE,TYR	108/363		323	1	22	1	4.7	3
A6	11	123479502	GRAMD1B	NM_020716.1	G	G/T	missense	ARG,LEU	407/739		1220	0.999	102	0.988	5.29	4
A9	4	94376856	GRID2	NM_001510.2	G	G/T	missense	ARG,LEU	530/1008		1589	0.999	102	0.984	5.97	4

A8	17	38121996	GSDMA	NM_178171.4	G	A/G	missense	GLY,GLU	19/446		56	1	98	1	5.36	3
A9	6	105280972	HACE1	NM_020771.3	T	A/T	missense	GLU,VAL	160/910		479	0.991	121	1	5.09	4
A9	20	30681721	HCK	NM_001172129.1	G	A/G	missense	ARG,GLN	362/506		1085	1	43	1	4.87	3
A9	5	45396745	HCN1	NM_021072.3	C	A/C	missense	GLY,VAL	360/891		1079	0.999	109	0.999	5.18	4
A7	18	44641547	HDHD2	NM_032124.4	G	A/G	missense	ARG,TRP	190/260		568	0.98	101	0.925	4.33	4
A3	14	73965039	HEATR4	NM_001220484.1	T	G/T	missense	GLN,PRO	789/1027		2366	0.93	76	1	4.07	3
A9	17	65144725	HELZ	NM_014877.3	G	C/G	missense	LEU,VAL	861/1943		2581	0.999	32	1	4.66	3
A1	15	28456220	HERC2	NM_004667.5	G	A/G	missense	LEU,PHE	2333/4835		6997	0.995	22	1	4.84	3
A9	7	26236251	HNRNPA2B1	NM_002137.3	T	C/T	missense	HIS,ARG	168/342		503	0.996	29	1	4.89	3
A9	18	22057513	HRH4	NM_001143828.1	C	C/G	stop-gained	SER,stop	299/303		896	unknown	NA	0.085	6.03	3
A9	18	22057513	HRH4	NM_001143828.1	C	C/G	stop-gained	SER,stop	299/303		896	unknown	NA	0.085	6.03	3
A7	11	122928501	HSPA8	NM_006597.4	C	C/G	missense	GLY,ARG	628/647		1882	0.281	125	1	4.65	3
A7	1	22211837	HSPG2	NM_005529.5	C	A/C	missense	ASP,TYR	396/4392		1186	1	160	0.944	5.1	4
A9	11	20398147	HTATIP2	NM_001098520.1	C	C/G	missense	ARG,GLY	143/277		427	0.997	125	0.997	3.94	4
A6	1	220275522	IARS2	NM_018060.3	G	G/T	missense	ARG,LEU	201/1013		602	1	102	0.966	5.96	4
A3	3	118647545	IGSF11	NM_001015887.1	C	A/C	stop-gained	GLY,stop	79/432		235	unknown	NA	1	4.66	4
A9	3	118621391	IGSF11	NM_001015887.1	C	C/G	missense	GLN,HIS	424/432		1272	0.999	24	1	5.13	3
A3	11	18735881	IGSF22	NM_173588.3	C	A/C	missense	GLY,CYS	581/1327		1741	1	159	0.988	3.49	4
A7	7	41739822	INHBA	NM_002192.2	G	A/G	stop-gained	GLN,stop	51/427		151	unknown	NA	1	5.49	4
A9	4	143043309	INPP4B	NM_001101669.1	C	A/C	missense	ASP,TYR	703/925		2107	1	160	1	5.87	4
A3	19	7166398	INSR	NM_000208.2	G	A/G	missense	THR,MET	543/1383		1628	0.999	81	0.935	5.04	3
A9	14	24653055	IPO4	NM_024658.3	T	A/T	missense	ASP,VAL	640/1082		1919	0.017	152	0.937	3.44	3
A9	11	9441967	IPO7	NM_006391.2	C	C/T	stop-gained	GLN,stop	246/1039		736	unknown	NA	1	5.17	4
A3	12	66638740	IRAK3	NM_001142523.1	G	G/T	missense	VAL,PHE	306/536		916	0.982	50	1	5.13	3
A7	10	15628607	ITGA8	NM_003638.1	G	A/G	missense	ALA,VAL	783/1064		2348	1	64	0.958	5.32	3
A3	10	7763715	ITIH2	NM_002216.2	G	C/G	missense	ARG,THR	281/947		842	1	71	1	5.29	3
A7	10	7762891	ITIH2	NM_002216.2	G	G/T	missense	ASP,TYR	235/947		703	0.806	160	0.992	4.33	3
A9	9	5080531	JAK2	NM_004972.3	A	A/G	splice-3	none	NA	NA		unknown	NA	0.998	6.06	3
A3	11	30032691	KCNA4	NM_002233.3	G	G/T	missense	PRO,GLN	512/654		1535	1	76	0.998	5.57	3
A6	12	4920098	KCNA6	NM_002235.3	C	A/C	missense	ASN,LYS	297/530		891	1	94	1	3.42	3
A1	11	17801111	KCNC1	NM_001112741.1	G	G/T	missense	GLY,VAL	538/586		1613	unknown	109	1	5.7	3
A3	20	49620875	KCNG1	NM_002237.3	A	A/C	missense	TYR,ASP	415/514		1243	1	160	1	5.49	4
A6	1	210857470	KCNH1	NM_002238.3	C	C/T	missense	ARG,GLN	681/963		2042	1	43	1	4.64	3
A3	1	202724575	KDM5B	NM_006618.3	A	A/T	stop-gained	TYR,stop	454/1545		1362	unknown	NA	0.986	-0.843	3
A9	4	55984911	KDR	NM_002253.2	C	A/C	missense	ARG,MET	73/1357		218	1	91	1	5.47	3
A1	4	123270710	KIAA1109	NM_015312.3	G	G/T	missense	GLY,VAL	4515/5006		13544	1	109	0.982	5.83	4
A1	10	24783445	KIAA1217	NM_001098500.1	A	A/G	missense	MET,VAL	486/1265		1456	0.998	21	1	5.78	3
A1	4	37446864	KIAA1239	NM_001144990.1	A	A/T	missense	ASP,VAL	1085/1743		3254	0.999	152	0.979	5.95	4
A3	1	33237091	KIAA1522	NM_001198972.1	C	A/C	missense	PRO,THR	712/1036		2134	1	38	0.981	4.53	3
A6	3	113378545	KIAA2018	NM_001009899.2	G	C/G	missense	GLN,GLU	662/2246		1984	0.951	29	1	5.27	3
A6	20	16488746	KIF16B	NM_001199865.1	C	C/G	splice-3	none	NA	NA		unknown	NA	0.998	5.86	3
A7	2	241697805	KIF1A	NM_001244008.1	G	A/G	missense	ARG,CYS	852/1792		2554	1	180	1	5.28	4
A9	4	55594034	KIT	NM_000222.2	C	A/C	missense	THR,ASN	607/977		1820	1	65	0.997	6.17	3
A9	4	55604704	KIT	NM_000222.2	T	A/T	missense	LEU,HIS	971/977		2912	1	99	0.961	5.72	3
A5	12	25380276	KRAS	NM_004985.3	T	A/T	missense	GLN,LEU	61/189		182	0.984	113	1	5.77	4
A3	7	149420833	KRBA1	NM_032534.2	G	G/T	stop-gained	GLU,stop	261/1031		781	unknown	NA	0.431	5.26	3
A3	7	149420833	KRBA1	NM_032534.2	G	G/T	stop-gained	GLU,stop	261/1031		781	unknown	NA	0.431	5.26	3
A6	1	209800312	LAMB3	NM_000228.2	C	A/C	missense	GLN,HIS	499/1173		1497	0.995	24	1	4.64	3
A9	12	50822769	LARP4	NM_001170803.1	C	C/T	missense	SER,PHE	73/583		218	0.997	155	1	3.56	4
A9	1	32742015	LCK	NM_001042771.1	G	A/G	missense	GLU,LYS	237/510		709	0.975	56	0.996	5.05	3
A3	2	48925889	LHCGR	NM_000233.3	G	G/T	missense	SER,TYR	244/700		731	0.952	144	1	5.87	4
A7	9	126794807	LHX2	NM_004789.3	G	G/T	missense	GLY,CYS	348/407		1042	1	159	0.986	5.67	4
A9	7	73513564	LIMK1	NM_001204426.1	C	C/T	stop-gained	GLN,stop	168/614		502	unknown	NA	1	5.59	4
A9	15	75105343	LMAN1L	NM_021819.2	G	G/T	stop-gained	GLY,stop	50/527		148	unknown	NA	0.992	4.87	4
A3	1	87797844	LMO4	NM_006769.3	A	A/C	missense	LYS,THR	49/166		146	0.999	78	1	5.57	3
A9	13	76414567	LMO7	NM_005358.5	G	A/G	stop-gained	TRP,stop	891/1350		2672	unknown	NA	0.999	5.45	4

A6	13	28155582	LNX2	NM_153371.3	C	A/C	missense	ASP,TYR	87/691		259	1		160	1	6.06	4
A3	4	62936624	LPHN3	NM_015236.4	T	C/T	stop-lost	stop,GLN	1470/1470		4408	unknown	NA		1	5.5	4
A7	2	141528455	LRP1B	NM_018557.2	C	C/G	missense	CYS,SER	1874/4600		5621	0.999		112	0.998	5.7	4
A7	3	37125125	LRRFIP2	NM_001134369.1	A	A/G	splice-5	none	NA	NA		unknown	NA		1	5.53	3
A3	12	40645032	LRRK2	NM_198578.3	A	A/G	splice-3	none	NA	NA		unknown	NA		0.942	5.66	3
A6	2	77745719	LRRTM4	NM_001134745.1	T	A/T	missense	ILE,PHE	426/591		1276	0.986		21	1	5.68	3
A9	21	30302786	LTN1	NM_015565.2	C	A/C	missense	ARG,LEU	1808/1813		5423	1		102	0.999	4.38	4
A9	2	160710182	LY75	NM_001198759.1	C	A/C	missense	TRP,CYS	874/1874		2622	1		215	1	4.93	4
A5	8	56910929	LYN	NM_001111097.2	G	A/G	missense	GLU,LYS	338/492		1012	1		56	0.997	5.52	3
A3	19	35800926	MAG	NM_001199216.1	G	A/G	missense	VAL,MET	436/602		1306	1		21	1	4.8	3
A9	5	179192397	MAML1	NM_014757.4	G	G/T	missense	GLY,VAL	129/1017		386	1		109	1	4.97	4
A2	14	65560493	MAX	NM_002382.3	C	C/G	missense	ARG,PRO	35/161		104	1		103	1	6.04	4
A3	14	47566170	MDGA2	NM_001113498.2	C	A/C	missense	GLY,VAL	361/1026		1082	0.999		109	1	5.65	4
A3	11	86157441	ME3	NM_001014811.1	G	G/T	missense	ALA,ASP	490/605		1469	0.983		126	1	5.34	4
A1	19	42861030	MEGF8	NM_001410.2	C	C/G	missense	ALA,GLY	1509/2779		4526	0.996		60	0.934	5.11	3
A9	15	37385840	MEIS2	NM_001220482.1	C	C/T	missense	GLY,ASP	194/471		581	0.991		94	1	6.03	3
A3	1	171753178	METTL13	NM_001007239.1	A	A/C	missense-nea	GLN,PRO	151/544		452	1		76	1	4.11	3
A3	15	89444909	MFGE8	NM_001114614.1	G	A/G	missense	ALA,VAL	248/336		743	1		64	0.993	5.36	3
A7	2	103347515	MFSD9	NM_032718.3	C	A/C	missense	GLY,VAL	89/475		266	1		109	0.978	5.18	4
A6	3	69990385	MITF	NM_000248.3	A	A/G	splice-3	none	NA	NA		unknown	NA		0.993	5.9	3
A6	14	75506640	MLH3	NM_001040108.1	T	A/T	missense	THR,SER	1182/1454		3544	0.977		58	1	5.81	3
A3	12	49426235	MLL2	NM_003482.3	G	A/G	stop-gained	GLN,stop	4085/5538		12253	unknown	NA		1	5.4	4
A7	12	49436527	MLL2	NM_003482.3	G	A/G	stop-gained	GLN,stop	1927/5538		5779	unknown	NA		1	5.36	4
A7	12	49434618	MLL2	NM_003482.3	G	A/G	missense	SER,LEU	2312/5538		6935	0.008		145	1	4.13	3
A7	12	49433784	MLL2	NM_003482.3	G	A/G	missense	SER,LEU	2590/5538		7769	0.001		145	1	4.3	3
A1	7	156798528	MNX1	NM_001165255.1	G	A/G	missense	ARG,CYS	86/190		256	1		180	0.998	4.25	4
A3	7	24681389	MPP6	NM_016447.2	G	G/T	stop-gained	GLU,stop	58/541		172	unknown	NA		1	4.88	4
A3	6	151358213	MTHFD1L	NM_001242767.1	C	C/T	missense	ALA,VAL	937/980		2810	1		64	1	5.27	3
A9	22	30412671	MTMR3	NM_021090.3	C	C/T	missense	SER,PHE	553/1199		1658	0.989		155	1	6.01	4
A9	4	100503179	MTTP	NM_000253.2	C	C/T	missense	SER,PHE	60/895		179	1		155	0.996	5.76	4
A3	3	108188955	MYH15	NM_014981.1	T	A/T	missense	GLU,ASP	516/1947		1548	0.947		45	0.985	3.79	3
A7	13	109445989	MYO16	NM_001198950.1	G	G/T	splice-5	none	NA	NA		unknown	NA		0.997	5.76	3
A9	12	57440868	MYO1A	NM_001256041.1	G	A/G	missense	ARG,CYS	152/1044		454	1		180	1	5.19	4
A3	11	20057524	NAV2	NM_001111018.1	G	C/G	missense	VAL,LEU	866/2366		2596	0.999		32	1	5.88	3
A7	12	78452891	NAV3	NM_014903.4	G	A/G	missense	ASP,ASN	878/2364		2632	0.997		23	1	5.82	3
A3	16	15781348	NDE1	NM_001143979.1	G	A/G	missense	ARG,LYS	168/336		503	0.982		26	0.986	5.86	3
A7	4	115898364	NDST4	NM_022569.1	A	A/C	missense	SER,ALA	349/873		1045	0.999		99	1	5.89	3
A9	18	56033318	NEDD4L	NM_001144964.1	C	C/G	missense	PRO,ALA	520/855		1558	0.992		27	1	5.54	3
A6	4	170347383	NEK1	NM_001199397.1	G	A/G	missense	SER,LEU	963/1287		2888	0		145	0.999	3.64	3
A2	12	45173637	NELL2	NM_001145107.1	G	G/T	stop-gained	CYS,stop	218/867		654	unknown	NA		1	3.54	4
A6	12	45004645	NELL2	NM_001145107.1	C	A/C	missense	GLY,VAL	568/867		1703	0.999		109	1	4.97	4
A9	20	50049264	NFATC2	NM_001136021.2	C	A/C	stop-gained	GLU,stop	668/902		2002	unknown	NA		1	5.31	4
A3	16	68157000	NFATC3	NM_004555.3	A	A/G	missense	LYS,ARG	405/1069		1214	0.999		26	1	5.52	3
A8	20	25470559	NINL	NM_025176.4	C	A/C	missense	ARG,SER	516/1383		1548	0.372		110	0.999	3.56	3
A6	10	134598878	NKX6-2	NM_177400.2	G	G/T	missense	ALA,GLU	162/278		485	1		107	1	3.77	4
A3	1	880127	NOC2L	NM_015658.3	C	A/C	missense	ALA,SER	733/750		2197	0.996		99	1	4.88	3
A6	18	31599352	NOL4	NM_001198546.1	T	C/T	missense	LYS,ARG	329/575		986	0.996		26	1	5.75	3
A6	9	35806520	NPR2	NM_003995.3	A	A/T	missense	TYR,PHE	835/1048		2504	1		22	1	6.17	3
A9	17	38251262	NR1D1	NM_021724.3	C	C/T	missense	ALA,THR	454/615		1360	0.993		58	1	4.93	3
A7	6	108492739	NR2E1	NM_003269.3	G	G/T	missense	GLY,CYS	35/386		103	1		159	1	5.39	4
A3	7	129394899	NRF1	NM_001040110.1	A	A/T	missense	THR,SER	464/504		1390	0.979		58	0.972	5.27	3
A9	2	206562395	NRP2	NM_003872.2	G	C/G	missense	LYS,ASN	67/927		201	1		94	1	4.98	3
A9	2	50280503	NRXN1	NM_001135659.1	G	A/G	missense	SER,LEU	1385/1548		4154	0.788		145	1	5.65	3
A9	14	79432554	NRXN3	NM_004796.4	C	A/C	missense	ALA,ASP	488/1062		1463	1		126	1	5.73	4
A3	5	176694614	NSD1	NM_022455.4	G	G/T	missense	CYS,PHE	1733/2697		5198	1		205	1	5.58	4
A9	15	88423603	NTRK3	NM_001012338.2	G	G/T	stop-gained	TYR,stop	744/840		2232	unknown	NA		1	5.49	4

A9	1	229613355	NUP133	NM_018230.2	G	A/G	missense	SER,PHE	582/1157		1745	1		155	1	5.38	4
A9	7	8791187	NXPH1	NM_152745.2	G	G/T	missense	ASP,TYR	202/272		604	0.989		160	1	6.06	4
A9	1	228480406	OBSCN	NM_001098623.1	C	C/T	missense	ARG,CYS	3596/7969		10786	1		180	0.609	3.49	3
A6	1	228401896	OBSCN	NM_001098623.1	C	C/T	missense	PRO,LEU	427/7969		1280	1		98	0.902	4.82	3
A3	2	10581806	ODC1	NM_002539.1	C	C/T	missense	GLY,GLU	357/462		1070	1		98	0.998	5.66	3
A6	4	183673029	ODZ3	NM_001080477.1	C	C/T	missense	ARG,CYS	1236/2700		3706	1		180	1	5.38	4
A3	10	50943396	OGDHL	NM_001143996.1	A	A/T	missense-nea	TYR,ASN	914/954		2740	1		143	1	5.4	4
A3	1	102269978	OLFM3	NM_058170.2	T	C/T	missense	TYR,CYS	398/459		1193	1		194	1	5.77	4
A9	8	54142231	OPRK1	NM_000912.3	G	C/G	missense	ARG,GLY	257/381		769	1		125	0.999	4.84	4
A7	11	55735245	OR10AG1	NM_001005491.1	C	A/C	missense	CYS,PHE	232/302		695	1		205	0.978	4.36	4
A9	11	5172944	OR52A1	NM_012375.2	A	A/G	missense	LEU,SER	219/313		656	0.963		145	0.403	4.21	3
A9	11	5510813	OR52D1	NM_001005163.2	C	A/C	missense	PRO,THR	293/319		877	0.994		38	0.995	5.58	3
A9	11	5080005	OR52E2	NM_001005164.2	G	C/G	missense	PRO,ALA	285/326		853	1		27	1	3.44	3
A6	11	58170842	OR5B3	NM_001005469.1	C	A/C	missense	GLY,VAL	14/315		41	0.989		109	0.974	4.05	4
A9	11	6815957	OR6A2	NM_003696.2	T	C/T	stop-lost	stop,TRP	328/328		983	unknown	NA		0.046	4.92	3
A9	11	6815957	OR6A2	NM_003696.2	T	C/T	stop-lost	stop,TRP	328/328		983	unknown	NA		0.046	4.92	3
A1	7	103841381	ORC5	NM_002553.3	A	A/C	missense	VAL,GLY	64/436		191	0.999		109	1	6.08	4
A3	4	4199402	OTOP1	NM_177998.1	G	G/T	missense	ARG,SER	387/613		1159	0.998		110	0.997	4.9	4
A7	14	23235855	OXA1L	NM_005015.3	C	C/G	missense	SER,TRP	42/496		125	0.988		177	0.005	3.24	3
A3	11	73988086	P4HA3	NM_182904.3	G	G/T	missense	GLN,LYS	427/545		1279	0.999		53	1	5.38	3
A6	1	17431497	PADI2	NM_007365.2	T	A/T	missense	GLU,VAL	51/666		152	0.998		121	1	5.25	4
A9	4	57319915	PAICS	NM_001079524.1	C	C/G	missense	ARG,GLY	367/426		1099	0.943		125	1	5.44	4
A9	11	124487282	PANX3	NM_052959.2	A	A/T	missense	GLU,VAL	146/393		437	1		121	0.94	4.92	4
A6	1	176640106	PAPPA2	NM_020318.2	G	G/T	missense-nea	ARG,SER	664/1792		1992	1		110	1	5.4	4
A2	2	206023569	PARD3B	NM_057177.6	G	A/G	missense	GLU,LYS	520/1137		1558	0.997		56	1	5.86	3
A7	10	51040935	PARG	NM_003631.2	C	A/C	missense	ARG,LEU	854/977		2561	0.985		102	0.996	5.67	4
A6	10	55782806	PCDH15	NM_001142763.1	G	G/T	missense	ALA,GLU	796/1963		2387	1		107	1	5.76	4
A6	10	56106181	PCDH15	NM_001142763.1	C	C/G	missense	ASP,HIS	185/1963		553	1		81	1	5.35	3
A6	13	58240866	PCDH17	NM_001040429.2	A	A/T	missense	ASP,VAL	899/1160		2696	1		152	1	5.83	4
A6	4	138451349	PCDH18	NM_019035.3	C	A/C	missense	ASP,TYR	632/1136		1894	1		160	0.827	4.96	3
A2	5	140710444	PCDHGA1	NM_018912.2	C	C/T	missense	ARG,CYS	65/932		193	1		180	0.998	3.42	4
A2	2	120404627	PCDP1	NM_001029996.3	G	C/G	splice-5	none	NA	NA		unknown	NA		0.996	4.26	3
A7	7	82580093	PCLO	NM_014510.2	G	C/G	missense	GLN,GLU	3271/4936		9811	0.998		29	1	5.16	3
A3	9	78710897	PCSK5	NM_001190482.1	G	A/G	missense	SER,ASN	329/1861		986	1		46	1	5.8	3
A3	12	20786627	PDE3A	NM_000921.4	C	A/C	missense-nea	SER,ARG	587/1142		1761	0.993		110	1	5.89	4
A6	4	55129912	PDGFRA	NM_006206.4	C	C/T	missense	PRO,LEU	149/1090		446	1		98	0.941	5.38	3
A9	7	94293846	PEG10	NM_001040152.1	G	C/G	stop-lost	stop,TYR	326/326		978	unknown	NA		1	4.2	4
A9	3	179519809	PEX5L	NM_001256750.1	C	C/G	missense	SER,THR	561/625		1682	0.999		58	1	5.46	3
A9	4	129789038	PHF17	NM_199320.2	C	C/T	missense	ARG,CYS	511/843		1531	1		180	1	5.28	4
A7	20	34459748	PHF20	NM_016436.4	G	G/T	stop-gained	GLU,stop	427/1013		1279	unknown	NA		0.904	2.04	3
A9	17	8741957	PIK3R6	NM_001010855.2	G	C/G	missense	SER,CYS	38/755		113	1		112	1	3.79	4
A7	8	110431421	PKHD1L1	NM_177531.4	C	A/C	stop-gained	SER,stop	819/4244		2456	unknown	NA		0.986	5.04	4
A3	16	81953096	PLCG2	NM_002661.3	G	G/T	missense	GLY,CYS	688/1266		2062	1		159	1	3.73	4
A7	2	198949586	PLCL1	NM_006226.3	G	A/G	missense	GLY,SER	449/1096		1345	1		56	1	5.94	3
A2	5	41382519	PLCXD3	NM_001005473.2	C	C/T	missense	ARG,GLN	74/322		221	0.997		43	1	6.07	3
A3	1	16054780	PLEKHM2	NM_015164.2	G	G/T	missense	GLY,TRP	617/1020		1849	1		184	0.959	5.61	4
A8	1	208257762	PLXNA2	NM_025179.3	C	C/T	missense	ARG,HIS	754/1895		2261	1		29	1	5.7	3
A3	3	129286421	PLXND1	NM_015103.2	T	A/T	missense	THR,SER	1334/1926		4000	0.999		58	0.995	5.3	3
A9	6	99857111	PNISR	NM_015491.1	G	A/G	missense	SER,LEU	204/806		611	0.081		145	1	4.59	3
A3	14	39646810	PNN	NM_002687.3	C	A/C	missense	SER,TYR	114/718		341	0.999		144	1	5.45	4
A9	5	74877102	POLK	NM_016218.2	G	C/G	missense	GLU,GLN	255/871		763	0.988		29	1	5.53	3
A6	14	77765073	POMT2	NM_013382.5	C	A/C	missense	ARG,LEU	322/751		965	0.024		102	1	5.25	3
A9	12	81661781	PPFIA2	NM_001220473.1	G	G/T	missense	SER,ARG	1132/1248		3396	1		110	0.966	-2.72	3
A9	17	48222543	PPP1R9B	NM_032595.3	G	C/G	missense	SER,CYS	480/818		1435	1		112	0.966	4.59	4
A8	1	186270729	PRG4	NM_001127708.1	T	C/T	missense	SER,PRO	28/1364		82	1		74	0.999	5.62	3
A9	2	37496830	PRKD3	NM_005813.3	C	C/T	missense-nea	ASP,ASN	569/891		1705	1		23	1	4.93	3

A1	1	71477992	PTGER3	NM_001126044.1	C	A/C	missense	CYS,PHE	358/391		1073	1	205	1	5.65	4
A3	1	44069828	PTPRF	NM_002840.3	G	G/T	missense	SER,ILE	1002/1908		3005	0.999	142	1	4.92	4
A4	3	61975376	PTPRG	NM_002841.3	G	A/G	missense	ASP,ASN	90/1446		268	0.998	23	0.992	5.92	3
A9	1	226108912	PYCR2	NM_013328.2	T	C/T	missense	THR,ALA	265/321		793	0.999	58	0.998	4.2	3
A3	12	57677714	R3HDM2	NM_014925.3	C	A/C	missense	ARG,LEU	341/977		1022	0.999	102	1	4.89	4
A3	7	66236972	RABGEF1	NM_014504.2	C	C/G	missense	PRO,ARG	29/492		86	0.002	103	1	4.37	3
A9	8	53570186	RB1CC1	NM_001083617.1	C	A/C	missense	ALA,SER	735/1592		2203	1	99	1	5.35	3
A7	11	66613520	RCE1	NM_001032279.1	T	A/T	missense	VAL,GLU	211/226		632	0.099	121	1	5.57	3
A6	1	167663442	RCSD1	NM_052862.3	G	G/T	missense	SER,ILE	126/417		377	1	142	1	5.18	4
A1	12	56114970	RDH5	NM_001199771.1	T	A/T	missense	MET,LYS	1/319		2	0.963	95	0.996	4.98	3
A9	7	103197556	RELN	NM_005045.3	C	A/C	stop-gained	GLY,stop	1889/3461		5665	unknown	NA	1	5.77	4
A2	13	34403994	RFC3	NM_002915.3	A	A/T	missense	ASP,VAL	138/357		413	1	152	1	5.37	4
A8	15	56385791	RFX7	NM_022841.5	C	C/G	missense	ASP,HIS	1379/1461		4135	0.999	81	1	5.87	3
A3	12	107245339	RIC8B	NM_018157.2	G	G/T	missense	ALA,SER	425/521		1273	0.986	99	1	5.46	3
A3	18	40503661	RIT2	NM_002930.2	A	A/T	missense	VAL,ASP	101/218		302	0.998	152	0.974	5.45	4
A3	19	54710219	RPS9	NM_001013.3	G	G/T	missense	GLY,VAL	99/195		296	0.934	109	1	4.49	4
A9	4	159566117	RXFP1	NM_001253727.1	C	A/C	missense	PRO,GLN	418/785		1253	1	76	0.998	5.56	3
A3	19	39034441	RYR1	NM_000540.2	G	G/T	missense	ALA,SER	3980/5039		11938	0.999	99	1	4.31	3
A3	15	34147114	RYR3	NM_001036.3	G	A/G	splice-5	none	NA	NA		unknown	NA	1	4.72	3
A9	15	34129911	RYR3	NM_001036.3	G	C/G	missense	LEU,PHE	3910/4871		11730	0.993	22	1	5.16	3
A6	15	33962623	RYR3	NM_001036.3	T	G/T	missense	VAL,GLY	1909/4871		5726	0.256	109	0.995	5.99	3
A9	13	23939407	SACS	NM_014363.4	G	C/G	missense	GLN,GLU	119/4580		355	0.998	29	1	5.62	3
A9	14	21993765	SALL2	NM_005407.1	G	C/G	missense	PRO,ALA	33/1008		97	0.999	27	1	4.01	3
A3	18	76753438	SALL3	NM_171999.3	C	C/G	missense	PRO,ALA	483/1301		1447	1	27	0.977	4.87	3
A7	3	169642926	SAMD7	NM_182610.2	T	C/T	splice-5	none	NA	NA		unknown	NA	1	5.16	3
A9	7	92734015	SAMD9	NM_001193307.1	G	A/G	missense	PRO,SER	466/1590		1396	1	74	0.998	4.38	3
A7	6	148840883	SASH1	NM_015278.3	A	A/G	missense	SER,GLY	355/1248		1063	0.991	56	1	5.43	3
A3	3	38781037	SCN10A	NM_006514.2	C	A/C	missense	GLY,VAL	750/1957		2249	0.9	109	0.867	3.27	3
A9	3	38888471	SCN11A	NM_014139.2	G	A/G	missense	SER,PHE	1697/1792		5090	0.998	155	0.078	5.46	3
A9	2	165987782	SCN3A	NM_001081676.1	C	A/C	missense	GLY,VAL	797/1952		2390	1	109	0.943	5.61	4
A3	3	38645204	SCN5A	NM_000335.4	G	G/T	missense-nea	THR,LYS	630/2016		1889	0.996	78	1	4.08	3
A9	16	5050728	SEC14L5	NM_014692.1	A	A/G	missense	GLU,GLY	348/697		1043	0.999	98	0.995	4.36	3
A3	9	139357349	SEC16A	NM_014866.1	C	C/T	missense	GLY,ASP	1628/2358		4883	1	94	0.995	5.56	3
A9	20	13869128	SEL1L2	NM_025229.1	C	C/T	missense	GLY,ARG	194/689		580	1	125	1	5.7	4
A6	20	13850834	SEL1L2	NM_025229.1	G	G/T	missense	LEU,ILE	374/689		1120	0.951	5	1	4.7	3
A6	1	169676488	SELL	NM_000655.4	T	A/T	missense	GLN,LEU	205/386		614	0.967	113	0.997	5.61	4
A6	3	9483899	SETD5	NM_001080517.1	C	A/C	missense	PHE,LEU	349/1443		1047	0.999	22	1	5.49	3
A3	9	135150809	SETX	NM_015046.5	T	A/T	splice-3	none	NA	NA		unknown	NA	1	5.76	3
A9	8	19221642	SH2D4A	NM_001174159.1	G	C/G	missense	ASP,HIS	256/455		766	1	81	1	5.88	3
A9	10	105526886	SH3PXD2A	NM_014631.2	C	A/C	missense	LYS,ASN	65/1106		195	1	94	1	3.3	3
A9	13	46357825	SIAH3	NM_198849.2	A	A/T	missense	LEU,GLN	168/270		503	1	113	0.999	5.19	4
A7	14	72117114	SIPA1L1	NM_015556.1	G	C/G	missense	GLU,ASP	627/1805		1881	1	45	1	4.58	3
A7	11	22399033	SLC17A6	NM_020346.2	C	C/T	missense	SER,LEU	499/583		1496	0.987	145	1	5.98	4
A3	20	61588932	SLC17A9	NM_022082.3	G	G/T	missense-nea	GLY,TRP	133/437		397	1	184	1	4.57	4
A3	6	160664743	SLC22A2	NM_003058.3	G	G/T	missense	PHE,LEU	380/556		1140	1	22	0.997	5.55	3
A3	5	150859026	SLC36A1	NM_078483.2	C	C/T	missense	ARG,CYS	379/477		1135	1	180	0.982	5.77	4
A9	10	18276549	SLC39A12	NM_001145195.1	C	C/G	missense	SER,CYS	413/692		1238	1	112	0.387	5.03	3
A3	5	492096	SLC9A3	NM_004174.2	G	A/G	missense	ALA,VAL	101/835		302	0.999	64	0.929	4.48	3
A3	12	21471776	SLCO1A2	NM_021094.3	C	C/T	missense	GLU,LYS	48/671		142	1	56	1	5.68	3
A7	12	21008055	SLCO1B3	NM_019844.3	G	C/G	missense	ASP,HIS	60/703		178	0.997	81	0.986	3.76	3
A9	4	20512126	SLIT2	NM_004787.1	A	A/T	missense	GLU,VAL	308/1530		923	1	121	1	5.5	4
A9	19	11138536	SMARCA4	NM_001128844.1	A	A/T	stop-gained	LYS,stop	1098/1648		3292	unknown	NA	0.939	5.04	4
A7	4	95196039	SMARCAD1	NM_001128429.2	A	A/G	splice-3	none	NA	NA		unknown	NA	0.984	5.55	3
A6	2	88409988	SMYD1	NM_198274.3	C	A/C	missense	PRO,HIS	477/491		1430	1	77	0.999	4.94	3
A9	18	67992556	SOCS6	NM_004232.3	C	C/G	missense	GLN,GLU	218/536		652	0.982	29	1	5.12	3
A8	20	35457611	SOGA1	NM_080627.2	G	C/G	missense	SER,CYS	306/1662		917	0.997	112	0.996	4.7	4

A3	5	151043705	SPARC	NM_003118.3	C	A/C	missense	ASP,TYR	276/304	826	0.941	160	1	5.44	4
A7	6	35837513	SRPK1	NM_003137.4	C	C/T	missense	CYS,TYR	386/656	1157	0.214	194	1	4.17	3
A1	16	2817796	SRRM2	NM_016333.3	C	C/T	missense	ARG,TRP	2423/2753	7267	0.997	101	0.998	4.96	4
A3	20	42088515	SRSF6	NM_006275.5	T	C/T	missense	CYS,ARG	121/345	361	0.997	180	0.998	5.86	4
A9	X	123181202	STAG2	NM_001042749.1	A	C/C	splice-3	none	NA	NA	unknown	NA	1	5.69	3
A3	2	191898336	STAT4	NM_001243835.1	C	C/T	missense	VAL,ILE	578/749	1732	0.998	29	1	5.65	3
A3	8	99719502	STK3	NM_001256312.1	G	A/G	missense	THR,ILE	158/520	473	0.994	89	1	5.56	3
A3	9	130416015	STXBP1	NM_001032221.3	A	A/T	missense	SER,CYS	37/595	109	1	112	0.996	5.36	4
A3	1	172579248	SUCO	NM_014283.3	G	G/T	missense	ARG,ILE	1205/1255	3614	0.999	97	1	5.56	3
A5	8	70498648	SULF1	NM_001128204.1	C	C/T	stop-gained	ARG,stop	157/872	469	unknown	NA	1	5.65	4
A6	6	152457899	SYNE1	NM_033071.3	A	A/T	missense	CYS,SER	8457/8750	25369	0.587	112	1	5.71	3
A3	14	64469765	SYNE2	NM_015180.4	G	G/T	missense	ASP,TYR	1372/6886	4114	0.986	160	0.691	4.76	3
A3	17	35900600	SYNRG	NM_001163544.1	C	A/C	missense	ARG,LEU	1005/1237	3014	0.994	102	0.998	3.32	4
A9	18	40853748	SYT4	NM_020783.3	C	C/G	missense	ASP,HIS	216/426	646	1	81	1	5.87	3
A9	6	132910074	TAAR5	NM_003967.2	G	G/T	missense	ALA,ASP	251/338	752	0.997	126	1	5.58	4
A2	17	80828207	TBCD	NM_005993.4	C	C/T	missense	ARG,CYS	476/1193	1426	1	180	0.829	5.53	3
A9	10	114057878	TECTB	NM_058222.1	G	C/G	missense	ARG,SER	241/330	723	0.999	110	1	3.16	4
A6	17	56643176	TEX14	NM_001201457.1	T	A/T	missense	LYS,ILE	1345/1498	4034	1	102	1	5.35	4
A3	3	30691868	TGFBR2	NM_001024847.2	A	A/T	stop-gained	LYS,stop	149/593	445	unknown	NA	1	4.77	4
A7	9	120476745	TLR4	NM_003266.3	G	G/T	missense	ARG,MET	740/800	2219	0.998	91	0.921	3.1	3
A2	10	98319418	TM9SF3	NM_020123.3	G	A/G	missense	ARG,TRP	220/590	658	1	101	0.997	3.88	4
A3	1	205241097	TMCC2	NM_001242925.1	G	G/T	missense	VAL,LEU	581/632	1741	0.998	32	1	4.26	3
A9	12	129180632	TMEM132C	NM_001136103.2	G	G/T	missense	GLY,VAL	638/1109	1913	1	109	0.467	4.62	3
A3	5	87493579	TMEM161B	NM_153354.3	C	A/C	missense	ALA,SER	365/488	1093	0.997	99	1	5.93	3
A3	1	45120613	TMEM53	NM_024587.2	A	A/G	missense	LEU,PRO	151/278	452	0.984	98	0.991	5.67	3
A9	1	74833622	TNNI3K	NM_001112808.2	T	A/T	stop-gained	LEU,stop	533/937	1598	unknown	NA	0.992	5.9	4
A3	17	7577127	TP53	NM_000546.5	C	A/C	stop-gained	GLU,stop	271/394	811	unknown	NA	1	5.13	4
A5	17	7578478	TP53	NM_000546.5	G	C/G	missense	PRO,ARG	151/394	452	1	103	0.974	4.63	4
A6	17	7577544	TP53	NM_000546.5	A	A/G	missense	MET,THR	246/394	737	1	81	1	4.62	3
A1	17	7577535	TP53	NM_000546.5	C	C/G	missense	ARG,THR	249/394	746	1	71	0.998	3.64	3
A1	3	127292479	TPRA1	NM_001136053.1	G	A/G	missense	THR,MET	336/374	1007	1	81	0.972	4.7	3
A5	16	2220691	TRAF7	NM_032271.2	T	C/T	missense	LEU,PRO	103/671	308	0.983	98	1	4.79	3
A3	11	4411255	TRIM21	NM_003141.3	C	A/C	stop-gained	GLU,stop	129/476	385	unknown	NA	0.849	4.32	3
A3	11	4411255	TRIM21	NM_003141.3	C	A/C	stop-gained	GLU,stop	129/476	385	unknown	NA	0.849	4.32	3
A7	5	14369558	TRIO	NM_007118.2	G	G/T	missense	GLY,CYS	1048/3098	3142	1	159	1	5.83	4
A7	4	8467172	TRMT44	NM_152544.2	C	C/T	missense	ARG,TRP	460/758	1378	1	101	0.451	4.75	3
A9	12	49723737	TROAP	NM_005480.3	C	C/G	missense	SER,CYS	421/779	1262	0.979	112	0	3.09	3
A6	8	72969151	TRPA1	NM_007332.2	C	C/G	splice-5	none	NA	NA	unknown	NA	1	5.58	3
A6	9	73250164	TRPM3	NM_001007471.2	G	C/G	stop-gained	SER,stop	532/1708	1595	unknown	NA	1	6.17	4
A7	17	3436201	TRPV3	NM_001258205.1	G	A/G	missense	THR,ILE	272/792	815	1	89	1	4.96	3
A9	11	18548342	TSG101	NM_006292.3	G	A/G	missense	LEU,PHE	8/391	22	0.96	22	1	4.8	3
A6	22	28504176	TTC28	NM_001145418.1	G	A/G	stop-gained	GLN,stop	553/2482	1657	unknown	NA	0.883	4.88	3
A6	22	28504176	TTC28	NM_001145418.1	G	A/G	stop-gained	GLN,stop	553/2482	1657	unknown	NA	0.883	4.88	3
A7	4	147796026	TTC29	NM_031956.2	C	C/T	missense	GLY,GLU	214/476	641	1	98	0.99	5.07	3
A9	9	135261986	TTF1	NM_001205296.1	C	C/T	missense	ASP,ASN	264/391	790	0.999	23	0.99	5.81	3
A9	20	36641926	TTI1	NM_014657.1	G	C/G	stop-gained	SER,stop	98/1090	293	unknown	NA	0.903	5.33	4
A3	2	179639851	TTN	NM_001256850.1	C	C/G	missense	CYS,SER	2196/34351	6587	1	112	1	5	4
A6	2	179629344	TTN	NM_001256850.1	C	C/G	missense	GLU,GLN	3300/34351	9898	1	29	0.995	5.69	3
A9	2	179444468	TTN	NM_001256850.1	A	A/G	missense	TYR,HIS	20845/34351	62533	1	83	0.993	5.28	3
A4	2	179416642	TTN	NM_001256850.1	C	C/T	missense	GLY,ARG	28688/34351	86062	1	125	1	5.76	4
A8	2	179596843	TTN	NM_001256850.1	G	A/G	missense	ALA,VAL	5301/34351	15902	1	64	1	6.17	3
A3	2	179596522	TTN	NM_001256850.1	G	G/T	missense	LEU,MET	5377/34351	16129	1	15	1	5.93	3
A3	2	179480115	TTN	NM_001256850.1	C	C/T	missense	ARG,HIS	14545/34351	43634	0.999	29	1	5.96	3
A9	2	179429821	TTN	NM_001256850.1	C	C/G	missense	ARG,PRO	25372/34351	76115	0.994	103	1	5	4
A3	2	179452075	TTN	NM_001256850.1	G	G/T	missense	ALA,ASP	19647/34351	58940	0.193	126	0.993	3.17	3
A3	1	162560255	UAP1	NM_003115.4	G	G/T	missense	GLY,CYS	438/506	1312	1	159	1	4.93	4

A5	9	33973212	UBAP2	NM_018449.2	C	C/T	missense	GLY,ARG	182/1120		544	1	125	1	6.07	4
A9	1	10231222	UBE4B	NM_001105562.2	G	A/G	missense	MET,ILE	1120/1303		3360	0.999	10	1	5.91	3
A9	5	176301097	UNC5A	NM_133369.2	G	G/T	missense	ALA,SER	339/843		1015	0.998	99	1	5.47	3
A9	1	215972345	USH2A	NM_206933.2	C	A/C	missense	ASP,TYR	3288/5203		9862	1	160	0.649	5.81	3
A7	1	216062253	USH2A	NM_206933.2	C	C/G	missense	GLY,ARG	2580/5203		7738	1	125	0.336	5.11	3
A9	1	215960153	USH2A	NM_206933.2	A	A/C	missense	CYS,GLY	3416/5203		10246	0.999	159	0.863	4.88	3
A6	17	58313507	USP32	NM_032582.3	C	A/C	missense	VAL,PHE	411/1605		1231	0.976	50	1	5.56	3
A9	17	5073990	USP6	NM_004505.2	G	C/G	missense	ARG,PRO	1245/1407		3734	0.99	103	0.994	2.35	4
A1	3	51457234	VPRBP	NM_001171904.1	C	A/C	stop-gained	GLY,stop	1010/1454		3028	unknown	NA	1	5.88	4
A1	3	184682289	VPS8	NM_001009921.2	G	A/G	missense	ALA,THR	1073/1429		3217	1	58	0.996	5.8	3
A3	1	159827945	VSIG8	NM_001013661.1	G	G/T	missense	ALA,ASP	122/415		365	0.998	126	0.999	4.86	4
A6	2	63609097	WDPCP	NM_015910.5	C	A/C	missense	SER,ILE	523/747		1568	0.833	142	0.999	3.62	3
A3	19	990856	WDR18	NM_024100.3	G	G/T	missense	TRP,LEU	201/433		602	0.936	61	1	3.73	3
A8	3	14208718	XPC	NM_001145769.1	C	C/T	missense	ARG,GLN	154/904		461	1	43	0.998	4.79	3
A9	3	142037481	XRN1	NM_001042604.1	G	C/G	stop-gained	SER,stop	1524/1694		4571	unknown	NA	0.991	3.88	4
A5	20	21314236	XRN2	NM_012255.3	C	C/T	missense	ARG,TRP	305/951		913	1	101	1	5.43	4
A9	8	64087916	YTHDF3	NM_152758.4	C	C/T	stop-gained	GLN,stop	26/586		76	unknown	NA	1	6.17	4
A9	8	64087908	YTHDF3	NM_152758.4	C	C/T	missense	SER,LEU	23/586		68	0.997	145	1	6.17	4
A9	6	109798055	ZBTB24	NM_014797.2	T	A/T	missense	HIS,LEU	344/698		1031	0.999	99	1	5.04	3
A9	1	31821726	ZCCHC17	NM_016505.2	C	C/G	missense	LEU,VAL	157/242		469	0.941	32	1	5.34	3
A7	9	88958079	ZCCHC6	NM_001185059.1	C	C/G	splice-3	none	NA	NA		unknown	NA	0.997	5.08	3
A6	20	50769812	ZFP64	NM_018197.2	G	A/G	missense	ARG,CYS	307/682		919	1	180	1	5.86	4
A3	16	68592421	ZFP90	NM_133458.2	G	A/G	missense	GLY,GLU	69/637		206	1	98	1	6.03	3
A7	8	124266425	ZHX1	NM_001017926.2	G	A/G	missense	LEU,PHE	588/874		1762	0.989	22	1	5.3	3
A7	19	57723733	ZNF264	NM_003417.4	G	G/T	missense	GLY,VAL	423/628		1268	1	109	0.996	1.98	4
A6	19	44891269	ZNF285	NM_152354.3	C	C/G	missense	GLY,ARG	380/591		1138	1	125	0.938	0.95	3
A9	6	87966952	ZNF292	NM_015021.1	C	C/T	missense	PRO,LEU	1202/2724		3605	0.974	98	0.997	5.65	3
A6	10	64159430	ZNF365	NM_014951.2	C	A/C	missense	SER,TYR	369/408		1106	0.104	144	0.994	4.85	3
A8	19	57036242	ZNF471	NM_020813.2	A	A/T	missense	LYS,ILE	269/627		806	0.996	102	0.999	4.09	4
A1	7	88389324	ZNF804B	NM_181646.2	C	C/T	missense	LEU,PHE	12/1350		34	1	22	1	4.06	3
A3	20	47877057	ZNFX1	NM_021035.2	C	A/C	missense	GLY,CYS	787/1919		2359	1	159	0.999	5.4	4

Supplementary Table 5. Significantly mutated genes

Gene Symbol	No. of nonsyn somatic mutations	samples mutated in Exome study	samples studied in Exome study	samples studied in Exome study	Contains highly deleterious mutations	No. of samples mutated in COSMIC	No. of samples studied in COSMIC	% of samples mutated in COSMIC	Cancer gene census in COSMIC	Length	P-value FCPT	P-value LRT	P-value CT	Supported by three methods
TP53	5	5	9	0.56	Yes	2292	6157	0.37	Yes	2261	1.6765E-05	4.9623E-08	1.1432E-08	yes
MLL2	5	3	9	0.33	Yes	64	431	0.15	Yes	18074	0.002656637	2.5714E-06	1.7635E-05	yes
NEK1	2	2	9	0.22	Yes	15	457	0.03		4366	0.029181032	6.171E-06	0.00011233	yes
OR52A1	2	2	9	0.22	Yes	6	423	0.01		942	0.007724892	9.8522E-06	4.2347E-06	yes
IGSF11	3	2	9	0.22	Yes	19	423	0.04		2739	0.014989485	3.6592E-05	3.7649E-05	yes
BBS7	2	1	9	0.11	Yes	11	423	0.03		2395	0.035859478	0.00010813	5.5975E-05	yes
PAPPA2	3	3	9	0.33	Yes	97	428	0.23		7061	0.041911842	0.00017868	0.0002513	yes
CDH10	3	2	9	0.22	Yes	83	428	0.19	Yes	4036	0.024577197	0.00055326	9.0296E-05	yes
TTN	13	6	9	0.67	Yes	244	467	0.52		138148	0.00121483	0.00066143	4.4806E-05	yes
B4GALT5	2	1	9	0.11	Yes	7	423	0.02		1194	0.058295381	0.00025472	0.00027761	
OR6A2	2	2	9	0.22	Yes	10	424	0.02		987	0.064678041	0.0003084	0.00016902	
ZNF642	2	2	9	0.22		5	424	0.01		2147	0.077082559	0.00041353	0.00029837	
ZNF804B	2	2	9	0.22	Yes	66	426	0.15		4062	0.081201905	0.00043048	0.00040654	
SLC27A6	2	2	9	0.22		20	424	0.05		2098	0.094132871	0.00059095	0.00057038	
SH3BP1	2	2	9	0.22		5	423	0.01		3069	0.117656068	8.4297E-05	0.00084572	
WDR18	2	2	9	0.22	Yes	2	423	0		1329	0.120182638	0.00094935	0.00057463	
PEG10	2	2	9	0.22	Yes	6	423	0.01		2547	0.126512727	0.00010156	0.00091695	
CTR9	2	1	9	0.11	Yes	12	425	0.03		3701	0.133088591	0.000106	0.00101429	
ASTN2	2	1	9	0.11	Yes	38	423	0.09		5398	0.139556888	0.00114445	0.00113684	
TEX14	2	2	9	0.22	Yes	23	591	0.04		4724	0.15324889	0.00013816	0.00145123	
ITIH2	2	2	9	0.22	Yes	23	423	0.05		3573	0.15486977	0.00148781	0.00158791	
IFNA5	1	1	9	0.11		3	424	0.01		573	0.15552072	0.00162416	0.00219526	
TTC14	2	2	9	0.22		20	423	0.05		4711	0.156854097	0.00014932	0.00127775	
FAM198B	2	2	9	0.22	Yes	12	425	0.03		1817	0.158284024	0.00161879	0.0012248	
MYO1A	2	2	9	0.22	Yes	13	433	0.03		5124	0.169616199	0.00171079	0.00166848	
FAM169A	2	2	9	0.22	Yes	12	425	0.03		2366	0.189493835	0.00023671	0.00185539	
PABPC1L	2	2	9	0.22		9	424	0.02		6429	0.214536186	0.00277152	0.00354304	
CSMD3	3	2	9	0.22	Yes	221	438	0.5		12037	0.216080434	0.00356232	0.00402412	
EGFR	2	2	9	0.22	Yes	12707	47277	0.27	Yes	5882	0.238269915	0.00347997	0.00422725	
KIT	2	2	9	0.22	Yes	24	1849	0.01	Yes	3309	0.238295451	0.00371159	0.00295263	
C10orf2	2	1	9	0.11		6	423	0.01		4022	0.239246788	0.00367736	0.00309616	
CCDC15	2	1	9	0.11	Yes	10	423	0.02		3854	0.242958011	0.00387509	0.00219086	
STARD9	3	3	9	0.33						19225	0.249389098	0.02259844	0.00721801	
ESRRG	2	2	9	0.22	Yes	27	425	0.06		7454	0.25947945	0.00047907	0.00315935	
HESX1	1	1	9	0.11		1	431	0		737	0.267525033	0.000389	0.00087342	
DNAH9	3	2	9	0.22	Yes	96	428	0.22		14184	0.272298417	0.00562346	0.00730982	

CD160	1	1	9	0.11		2	423	0		866	0.281768604	0.00043938	0.00153729
PAICS	1	1	9	0.11	Yes					1580	0.287708615	0.00045483	0.00161221
CLRN1	1	1	9	0.11		7	423	0.02		1210	0.296989838	0.00049767	0.0013416
COPB1	2	1	9	0.11		11	423	0.03		6461	0.304656892	0.00635023	0.0040804
RYR3	3	3	9	0.33	Yes	106	423	0.25		16016	0.305385434	0.03655609	0.01040561
HIST1H4K	1	1	9	0.11		3	423	0.01		315	0.306191614	0.00054942	0.00178337
GAS2L2	2	2	9	0.22	Yes	12	432	0.03		3173	0.319683576	0.00080159	0.00389345
HDHD2	1	1	9	0.11	Yes	1	424	0		798	0.322505909	0.00062023	0.00197113
SEL1L2	2	2	9	0.22	Yes	23	424	0.05		6299	0.330151753	0.00767685	0.00547715
USH2A	3	3	9	0.33	Yes	170	432	0.39		16539	0.33139281	0.00865341	0.01275949
ATXN7L3B	1	1	9	0.11	Yes					297	0.333721178	0.00068891	0.00289842
NEUROG2	1	1	9	0.11		6	423	0.01		822	0.337698608	0.00070244	0.00138493
YTHDF3	2	2	9	0.22	Yes					20788	0.337847135	0.00703223	0.00960595
FNBP1	2	2	9	0.22					Yes	8780	0.342897511	0.00820445	0.0070706
LAMB3	2	2	9	0.22	Yes	12	423	0.03		3751	0.355999099	0.00104686	0.00697295
PLCXD3	2	2	9	0.22	Yes	20	431	0.05		975	0.365659653	0.00086186	0.00271479
NELL2	2	2	9	0.22	Yes	28	424	0.07		5185	0.371785696	0.00116089	0.00741991
SHISA2	1	1	9	0.11		7	423	0.02		894	0.373752899	0.00092571	0.00302434
TLR4	2	2	9	0.22	Yes	58	608	0.1		5964	0.373864581	0.01037984	0.00641109
ARL6IP1	1	1	9	0.11	Yes	4	423	0.01		917	0.375053598	0.00092556	0.00283836
TCL1B	1	1	9	0.11		5	423	0.01		396	0.375053598	0.0009382	0.00502828
KRTAP22-2	1	1	9	0.11						141	0.378262555	0.00097119	0.00210804
KRTAP4-5	1	1	9	0.11		4	423	0.01		549	0.37965987	0.00096708	0.00318969
ARHGEF18	2	1	9	0.11		4	434	0.01		6132	0.380140923	0.01059122	0.00928046
PRAMEF13	1	1	9	0.11		3	424	0.01		1434	0.380901934	0.00095037	0.0024029
C5orf51	1	1	9	0.11	Yes	2	423	0		1196	0.382050424	0.00097153	0.00302309
LRRD1	1	1	9	0.11						6708	0.387789307	0.00092061	0.00788987
GABPA	1	1	9	0.11	Yes	5	431	0.01		2096	0.387789307	0.00099001	0.00283614
ENY2	1	1	9	0.11	Yes	1	431	0		1442	0.398548066	0.00108543	0.0024167
F2R	1	1	9	0.11	Yes	7	423	0.02		1508	0.398548066	0.00108812	0.00234279
VENTX	1	1	9	0.11		8	423	0.02		786	0.39982765	0.0011129	0.00520233
CT47B1	1	1	9	0.11		8	423	0.02		906	0.407464302	0.00116481	0.00045154
BTBD18	1	1	9	0.11	Yes					2292	0.408572742	0.0011286	0.00507872
MYH15	2	2	9	0.22	Yes	41	426	0.1		6419	0.40975934	0.01293474	0.00862874
DOCK10	2	1	9	0.11	Yes	31	423	0.07		11018	0.413253376	0.01201452	0.01473921
SLC35G3	1	1	9	0.11						1020	0.413628577	0.00121206	0.00249071
REG1A	1	1	9	0.11		25	423	0.06		516	0.415322423	0.00125133	0.00488005
HTATIP2	1	1	9	0.11	Yes	3	423	0.01		1257	0.41549927	0.00122705	0.00253668
HHLA1	1	1	9	0.11		2	423	0		1876	0.430343253	0.00133583	0.00472995
IMPG1	1	1	9	0.11		26	424	0.06		2694	0.432050575	0.00132711	0.0042841
AATK	2	1	9	0.11	Yes	8	779	0.01		7445	0.43334831	0.01478567	0.01133355
C6orf195	1	1	9	0.11	Yes	2	423	0		387	0.433759383	0.00142764	0.00368183

MYO16	2	2	9	0.22	Yes	44	423	0.1		7124	0.44436445	0.00187045	0.01477585
NAV3	2	2	9	0.22	Yes	103	434	0.24		14433	0.449997145	0.00157141	0.01682363
RHOXF2B	1	1	9	0.11						879	0.452404419	0.00159118	0.00519128
MZT2A	1	1	9	0.11		1	423	0		1550	0.452641655	0.00156521	0.00343794
FAM194B	1	1	9	0.11						3178	0.452841265	0.00149939	0.00577672
TEKT4	1	1	9	0.11		14	423	0.03		1509	0.453634861	0.00158966	0.00315851
ABHD10	1	1	9	0.11	Yes	7	423	0.02		1198	0.455993715	0.00162022	0.00473165
TM9SF3	1	1	9	0.11	Yes	2	423	0		4146	0.461904816	0.00158705	0.00778245
ELAVL2	1	1	9	0.11	Yes	18	423	0.04		2210	0.461904816	0.00165989	0.00536403
UNC13C	2	2	9	0.22		55	423	0.13		7521	0.462447405	0.01772117	0.01401363
KIAA2018	2	2	9	0.22	Yes	27	426	0.06		6767	0.466181538	0.00218557	0.01155565
OR4P4	1	1	9	0.11		24	425	0.06		942	0.46936025	0.00180522	0.00626509
MAX	1	1	9	0.11	Yes	3	565	0.01		1601	0.471599815	0.00176823	0.00389445
RIT2	1	1	9	0.11	Yes	11	567	0.02		708	0.476989677	0.00189283	0.00431214
COMMD9	1	1	9	0.11	Yes	1	423	0		1305	0.478426806	0.00188812	0.00776113
OTOP1	1	1	9	0.11	Yes	14	424	0.03		1857	0.479382123	0.0018606	0.00404613
CXCL14	1	1	9	0.11	Yes	2	431	0		415	0.479800041	0.00193483	0.00963938
KLK4	1	1	9	0.11		8	433	0.02		868	0.482032873	0.00193257	0.00873402
C9orf135	1	1	9	0.11		1	423	0		2015	0.483717731	0.0019003	0.00443686
B3GNT8	1	1	9	0.11	Yes	5	423	0.01		1197	0.484004803	0.00194429	0.00604441
SRSF6	1	1	9	0.11	Yes					1208	0.48769175	0.00200933	0.00637451
OR1L4	1	1	9	0.11		11	423	0.03		939	0.492719573	0.00208214	0.00496526
DSCAML1	2	1	9	0.11	Yes	46	427	0.11		7205	0.495656326	0.02077352	0.01844516
PARG	1	1	9	0.11	Yes	1	557	0		3016	0.498247583	0.00206045	0.00540645
NKX6-2	1	1	9	0.11	Yes	2	423	0		843	0.498247583	0.00217595	0.00786352
SSNA1	1	1	9	0.11		2	423	0		2286	0.498925855	0.00211045	0.00449579
PSKH2	1	1	9	0.11		25	508	0.05		2476	0.499281059	0.00209354	0.00494703
FOXG1	1	1	9	0.11	Yes	19	567	0.03		1473	0.500696202	0.00215797	0.00683733
MTMR12	1	1	9	0.11		10	431	0.02		2826	0.50167089	0.00208934	0.00595597
OR4C46	1	1	9	0.11		30	424	0.07		933	0.504791725	0.00225589	0.00423001
RCE1	1	1	9	0.11	Yes	2	431	0		1274	0.511505791	0.00232658	0.00583186
KRAS	1	1	9	0.11	Yes	3343	20941	0.16	Yes	726	0.513892332	0.00241003	0.0108876
C20orf151	1	1	9	0.11		3	423	0.01		2034	0.516956191	0.00235558	0.00514714
POLR3G	1	1	9	0.11		4	424	0.01		948	0.51705773	0.00244685	0.00508539
ASB14	1	1	9	0.11		1	431	0		2503	0.517133125	0.00232334	0.0081789
WDR87	2	2	9	0.22		2	423	0		8811	0.518086859	0.0239174	0.01653125
CHST2	1	1	9	0.11		12	423	0.03		1596	0.519853231	0.00242337	0.00780807
OR5A1	1	1	9	0.11		13	424	0.03		951	0.520631257	0.00249008	0.00960018
OR5H14	1	1	9	0.11		16	423	0.04		936	0.524813921	0.00256981	0.00495618
LYN	1	1	9	0.11	Yes	8	645	0.01		2291	0.525652494	0.00248251	0.00790591
AVIL	1	1	9	0.11	Yes	8	423	0.02		2997	0.525695707	0.00241782	0.00700462
COL4A3	2	1	9	0.11	Yes	20	434	0.05		7066	0.526337768	0.00319821	0.01378485

CDKN2A	1	1	9	0.11	Yes	341	2925	0.12		4378	0.528004036	0.00252106	0.00864337
ANKRD22	1	1	9	0.11		2	423	0		1054	0.532792747	0.00268923	0.00712733
MATN2	2	2	9	0.22		14	423	0.03		8470	0.534510012	0.00324991	0.02330294
XRN2	1	1	9	0.11	Yes	12	423	0.03		3077	0.537748319	0.00261091	0.00982719
C22orf46	1	1	9	0.11						1562	0.539768704	0.00278958	0.01092302
TAAR1	1	1	9	0.11		10	423	0.02		1023	0.539966922	0.0028242	0.00125638
OR8H2	1	1	9	0.11		27	424	0.06		942	0.541408926	0.00285178	0.00603826
RIOK2	1	1	9	0.11		9	457	0.02		3280	0.541612454	0.00267748	0.00993866
MAP1S	1	1	9	0.11		5	423	0.01		3244	0.543732201	0.00275583	0.0064695
OXA1L	1	1	9	0.11	Yes	5	431	0.01		1972	0.547262178	0.002843	0.00608518
HIST1H2AA	1	1	9	0.11		7	424	0.02		399	0.547656933	0.00300495	0.01292513
OR2D3	1	1	9	0.11		12	424	0.03		996	0.551113022	0.00302778	0.00982312
R3HDM2	1	1	9	0.11	Yes	3	423	0.01		5699	0.551993262	0.00267481	0.01352009
RDH5	1	1	9	0.11	Yes	2	423	0		1987	0.555436138	0.00303997	0.01231363
SH2D1B	1	1	9	0.11		4	424	0.01		999	0.557980821	0.00316135	0.0111516
CRX	1	1	9	0.11		10	423	0.02		2034	0.558264657	0.0031198	0.01484494
ATR	2	1	9	0.11	Yes	33	648	0.05		10133	0.558398705	0.02982665	0.02429714
SAMD7	1	1	9	0.11	Yes	14	424	0.03		1372	0.558669524	0.00313892	0.00836789
PANX3	1	1	9	0.11	Yes	10	425	0.02		1191	0.558763234	0.00314753	0.01364189
MNX1	1	1	9	0.11	Yes	4	565	0.01		1783	0.558846334	0.00318547	0.01549292
GLYATL3	1	1	9	0.11	Yes	1	423	0		913	0.559416213	0.00318918	0.01607931
CHI3L1	1	1	9	0.11	Yes	3	424	0.01		1651	0.559811728	0.00311118	0.00893556
FCER1A	1	1	9	0.11		16	557	0.03		948	0.563236717	0.00327442	0.01387595
CTLA4	1	1	9	0.11	Yes	4	423	0.01		1235	0.564372012	0.00325709	0.00862971
SIAH3	1	1	9	0.11	Yes	7	423	0.02		2973	0.564662516	0.00320957	0.01452094
OR13D1	1	1	9	0.11		10	424	0.02		1044	0.567542485	0.00335862	0.01017503
LMO4	1	1	9	0.11	Yes	1	431	0		1792	0.567732881	0.00329848	0.01402
FGD6	1	1	9	0.11	Yes	9	423	0.02		5671	0.568279153	0.00298828	0.01594787
TRAM1L1	1	1	9	0.11		9	423	0.02		1113	0.569748058	0.0033926	0.00838398
SERPINA9	1	1	9	0.11		15	433	0.03		4750	0.569926629	0.00304094	0.01531166
GABRR1	1	1	9	0.11		15	423	0.04		1852	0.570465673	0.0033459	0.0134253
GPR65	1	1	9	0.11	Yes	8	423	0.02		1017	0.570658913	0.00342751	0.01611738
HNRNPA2B1	1	1	9	0.11	Yes	10	423	0.02	Yes	2608	0.570694008	0.00338495	0.00835681
CLEC2B	1	1	9	0.11		1	423	0		599	0.574815086	0.00356023	0.00986221
RFC3	1	1	9	0.11	Yes	2	432	0		1140	0.577278813	0.00355565	0.0123312
ZWINT	1	1	9	0.11		10	433	0.02		5021	0.57794763	0.00325826	0.01398258
ELMOD2	1	1	9	0.11		1	423	0		1287	0.579817213	0.00360546	0.00802824
SYNRG	1	1	9	0.11	Yes	14	425	0.03		8410	0.580203741	0.00305107	0.01997711
CYLC2	1	1	9	0.11		14	423	0.03		1062	0.580500365	0.00364369	0.0165477
OPRK1	1	1	9	0.11	Yes	8	423	0.02		1816	0.581091292	0.00354216	0.00656567
SEC14L5	1	1	9	0.11	Yes	9	423	0.02		2136	0.58173601	0.00349262	0.00768119
LTN1	1	1	9	0.11	Yes	15	558	0.03		8330	0.581864339	0.00305027	0.02152117

CCL8	1	1	9	0.11		1	423	0		418	0.582483534	0.00375065	0.01181615
TNIP3	1	1	9	0.11		9	423	0.02		2009	0.58509244	0.00368859	0.01201859
PRDM6	1	1	9	0.11						2689	0.585443369	0.00360056	0.00741
IARS2	1	1	9	0.11	Yes	17	425	0.04		4639	0.587077796	0.00343431	0.0115641
OR2S2	1	1	9	0.11		5	423	0.01		963	0.58880298	0.0038364	0.01491016
YY1	1	1	9	0.11		1	431	0		2328	0.589761727	0.00375266	0.00616507
NR2E1	1	1	9	0.11	Yes	13	424	0.03		1987	0.590261079	0.00377275	0.01661468
TRAF7	1	1	9	0.11	Yes	8	566	0.01		2163	0.595509607	0.00380701	0.00254923
GFRAL	1	1	9	0.11		28	423	0.07		1212	0.596771364	0.00401872	0.01820307
PET112	1	1	9	0.11		5	423	0.01		4161	0.598884121	0.0037094	0.01167587
C14orf28	1	1	9	0.11		4	424	0.01		1477	0.598969207	0.00405832	0.01420875
ENPP2	1	1	9	0.11	Yes	25	432	0.06		4722	0.602822777	0.00386778	0.00836535
ADAMTS20	1	1	9	0.11		40	431	0.09		6806	0.606148657	0.00363105	0.01926477
EGFLAM	1	1	9	0.11	Yes	31	425	0.07		3629	0.606148657	0.00391806	0.01113859
GHSR	1	1	9	0.11		16	557	0.03		1980	0.606644199	0.00409893	0.01315819
ASB5	1	1	9	0.11	Yes	18	432	0.04		1717	0.608039943	0.00428641	0.02271776
FOXK1	1	1	9	0.11		7	423	0.02		2893	0.608043199	0.00408078	0.01395944
USP6	1	1	9	0.11	Yes	15	565	0.03	Yes	5036	0.608061802	0.00383833	0.01424071
IL27RA	1	1	9	0.11		7	423	0.02	Yes	1953	0.608523916	0.00417729	0.0160981
SSBP3	1	1	9	0.11		3	423	0.01		3708	0.609135543	0.00418347	0.01302023
VPS8	1	1	9	0.11	Yes	15	423	0.04		7197	0.609522867	0.00382835	0.01916598
ZNF696	1	1	9	0.11		3	423	0.01		2509	0.609814809	0.00434589	0.02142245
C7orf72	1	1	9	0.11		1	423	0		1344	0.611455466	0.00438895	0.02065811
NRF1	1	1	9	0.11	Yes	7	423	0.02		2276	0.612125074	0.00432497	0.0212727
POLR1D	1	1	9	0.11		4	423	0.01		4091	0.613280714	0.00433791	0.01806046
SLC36A1	1	1	9	0.11	Yes	8	423	0.02		2647	0.613423648	0.00425329	0.0128299
HCK	1	1	9	0.11	Yes	17	645	0.03		2048	0.613423648	0.00433039	0.0110384
AKR1C4	1	1	9	0.11		4	423	0.01		1841	0.615971634	0.00451808	0.01978097
NFAT5	1	1	9	0.11		10	423	0.02		6508	0.616538952	0.00393251	0.01573447
SETD5	2	2	9	0.22	Yes	11	423	0.03	Yes	12154	0.621575581	0.04172048	0.03120287
ALLC	1	1	9	0.11		12	423	0.03		1840	0.623178195	0.00465399	0.02054449
FKBP6	1	1	9	0.11	Yes	10	431	0.02		1325	0.624586807	0.00474322	0.0241824
GRAMD1B	1	1	9	0.11	Yes	17	423	0.04		3067	0.624745024	0.00447	0.01066284
POMT2	1	1	9	0.11	Yes	5	423	0.01		5800	0.624831451	0.00422029	0.01595723
IFNA4	1	1	9	0.11		6	423	0.01		573	0.625934833	0.00491418	0.01547541
CALCB	1	1	9	0.11	Yes	1	423	0		518	0.627880321	0.00497124	0.01615887
SECISBP2	1	1	9	0.11		10	424	0.02		11635	0.628207167	0.00421998	0.02250265
SUDS3	1	1	9	0.11		1	423	0		1782	0.628281936	0.00483628	0.01115577
GRID2	1	1	9	0.11	Yes	36	425	0.08		5322	0.628676456	0.00439525	0.01438646
GABRG2	1	1	9	0.11	Yes	21	423	0.05		1723	0.629091844	0.00486548	0.01833687
ASZ1	1	1	9	0.11	Yes	10	423	0.02		1617	0.631303109	0.00493597	0.01805667
FBXL4	1	1	9	0.11	Yes	4	431	0.01		1887	0.631364616	0.00489777	0.01144863

EMILIN2	1	1	9	0.11	Yes	14	423	0.03		3688	0.637401267	0.00490175	0.01486235
TCF7	1	1	9	0.11		2	423	0		4741	0.639163335	0.00473979	0.01390275
CHCHD3	1	1	9	0.11		3	423	0.01		3589	0.639396348	0.00506483	0.01976882
EMB	1	1	9	0.11		4	424	0.01		1888	0.639836003	0.00520582	0.01746697
SVIP	1	1	9	0.11						2000	0.640246921	0.00535041	0.0204897
C12orf10	1	1	9	0.11	Yes	5	424	0.01		1700	0.642784408	0.00522388	0.02357911
CCZ1	1	1	9	0.11		1	423	0		1895	0.64298395	0.00527535	0.02315157
RXRA	1	1	9	0.11		6	423	0.01		12304	0.643315795	0.0049394	0.0127694
AMIGO2	1	1	9	0.11	Yes	3	423	0.01		1572	0.643695299	0.00532569	0.02705205
CHI3L2	1	1	9	0.11		8	423	0.02		2534	0.644068828	0.0052399	0.0114114
CDH20	1	1	9	0.11	Yes	29	423	0.07		2439	0.645649796	0.00524015	0.02161366
PKHD1L1	2	2	9	0.22	Yes	86	423	0.2		13080	0.647229432	0.04694777	0.04417017
GPR6	1	1	9	0.11	Yes	7	423	0.02		3261	0.647335087	0.00511389	0.01082772
SEC14L3	1	1	9	0.11		3	423	0.01		2288	0.64799883	0.00537609	0.02352646
NOL4	1	1	9	0.11	Yes	18	423	0.04		2360	0.651130829	0.00544822	0.02043616
GSDMA	1	1	9	0.11	Yes	1	423	0		1371	0.652790124	0.00565913	0.01563365
KCNG1	1	1	9	0.11	Yes	10	424	0.02		3364	0.652904213	0.00518916	0.02018506
ZNF799	1	1	9	0.11		4	423	0.01		2125	0.654834708	0.005653	0.01281002
IL2RA	1	1	9	0.11		6	424	0.01	Yes	2052	0.655126441	0.00572839	0.01771039
SLC1A6	1	1	9	0.11		12	423	0.03		2719	0.655649082	0.00552072	0.02376293
TPRA1	1	1	9	0.11	Yes	4	424	0.01	Yes	2323	0.655977594	0.00557943	0.02811344
ELMOD3	1	1	9	0.11		4	423	0.01		5083	0.658626396	0.00545676	0.01821697
DEPDC4	1	1	9	0.11	Yes	4	423	0.01		2920	0.659325746	0.00584492	0.021629
FAIM	1	1	9	0.11						866	0.659546986	0.00603445	0.02282369
TFCP2L1	1	1	9	0.11		7	423	0.02		1485	0.659863337	0.00588193	0.02035425
OR10AG1	1	1	9	0.11	Yes	23	423	0.05		909	0.659961892	0.0060547	0.01370318
MFGE8	1	1	9	0.11	Yes	5	424	0.01		2424	0.66021183	0.00589822	0.02813468
KCNH1	1	1	9	0.11	Yes	34	426	0.08		3399	0.660368877	0.00556964	0.01766272
SLC2A3	1	1	9	0.11		14	423	0.03		2013	0.661624996	0.00594925	0.02835556
COPZ1	1	1	9	0.11	Yes	4	423	0.01		1846	0.661624996	0.00596664	0.02579548
LBP	1	1	9	0.11		13	424	0.03		1530	0.662788998	0.00600651	0.02636401
GPR98	2	2	9	0.22		68	423	0.16		29789	0.664395008	0.03631894	0.06828407
PCDHB3	1	1	9	0.11		22	424	0.05		2394	0.665309311	0.00588299	0.02601801
INHBC	1	1	9	0.11		4	423	0.01		1385	0.666051259	0.0061685	0.02098664
DGCR14	1	1	9	0.11	Yes	6	423	0.01		1524	0.667081528	0.0061657	0.02707896
CNTNAP2	1	1	9	0.11	Yes	76	423	0.18		10406	0.667452995	0.00554097	0.01747843
USP36	1	1	9	0.11		11	566	0.02		4001	0.667519972	0.00569734	0.01479643
OR5H2	1	1	9	0.11		12	424	0.03		948	0.667877243	0.00636474	0.02290563
SLC17A6	1	1	9	0.11	Yes	24	425	0.06		1791	0.667952146	0.00617872	0.02136002
CHL1	1	1	9	0.11	Yes	28	424	0.07		10911	0.667988202	0.00539565	0.02590502
BACE1	1	1	9	0.11	Yes	5	423	0.01		3225	0.66849512	0.0058825	0.01028011
PHF17	1	1	9	0.11	Yes	9	423	0.02		4385	0.669838531	0.00574128	0.01267887

FSD2	1	1	9	0.11		9	423	0.02		2592	0.671434686	0.0061656	0.01714961
FLRT2	1	1	9	0.11	Yes	24	423	0.06		5606	0.671869907	0.00609995	0.02597083
ATL3	1	1	9	0.11		2	423	0		3160	0.672041589	0.00617453	0.01840455
ZBTB24	1	1	9	0.11	Yes	8	423	0.02		2112	0.672157513	0.0062862	0.0232573
ADRA1D	1	1	9	0.11		7	557	0.01		1725	0.672373777	0.0064286	0.01761858
SLC6A17	1	1	9	0.11		12	423	0.03		2764	0.672976346	0.00613001	0.02479095
NAT8	1	1	9	0.11		2	423	0		687	0.674560805	0.0066602	0.02081352
BIRC7	1	1	9	0.11	Yes	9	566	0.02		1385	0.675071182	0.00648762	0.01753352
MCTS1	1	1	9	0.11		4	431	0.01		2819	0.676166972	0.00651125	0.01918351
NT5DC2	1	1	9	0.11		3	423	0.01		4151	0.678164054	0.00614883	0.01464242
OR4L1	1	1	9	0.11		15	424	0.04		942	0.6784097	0.0068046	0.01434029
DNAH5	2	1	9	0.11	Yes	92	425	0.22		14678	0.67879379	0.05325491	0.04659419
ZNF471	1	1	9	0.11	Yes	7	423	0.02		1893	0.678875903	0.00665151	0.02777
ODC1	1	1	9	0.11	Yes	4	423	0.01		2171	0.679932231	0.00664413	0.032582
RALBP1	1	1	9	0.11		3	557	0.01		2342	0.680133576	0.00659417	0.02621211
TPTE2	1	1	9	0.11		21	424	0.05		1958	0.680610866	0.0066952	0.0321822
ORC5	1	1	9	0.11	Yes	9	432	0.02		2624	0.681740747	0.00674532	0.03133147
FEZ1	1	1	9	0.11		8	431	0.02		3687	0.682906146	0.00650673	0.02657182
PCDH15	2	2	9	0.22	Yes	73	426	0.17		14997	0.686776999	0.00768371	0.05231994
CD79B	1	1	9	0.11	Yes	3	557	0.01	Yes	765	0.687891482	0.00723158	0.02429515
WDR5B	1	1	9	0.11		2	423	0		996	0.688338511	0.00724263	0.02550147
LILRB2	1	1	9	0.11		29	424	0.07		3033	0.688730348	0.00670348	0.03489912
GAS7	1	1	9	0.11	Yes	17	565	0.03	Yes	1850	0.689585706	0.00704949	0.03430546
DCLK2	1	1	9	0.11	Yes	7	457	0.02		2802	0.689958552	0.00692982	0.03244653
SCGN	1	1	9	0.11		8	423	0.02		935	0.690113962	0.00731623	0.0267593
APOB	2	1	9	0.11		92	427	0.22		14982	0.690251923	0.05762652	0.0434434
TAF15	1	1	9	0.11		1	565	0	Yes	1844	0.690430527	0.00710757	0.03184536
OR5B3	1	1	9	0.11	Yes	17	423	0.04		948	0.691432418	0.00740111	0.01399222
ADCY2	1	1	9	0.11	Yes	42	423	0.1		7097	0.691768693	0.00646562	0.01893015
RANBP9	1	1	9	0.11		7	619	0.01		3097	0.691870881	0.00703674	0.01997279
OR5J2	1	1	9	0.11		28	423	0.07		942	0.694466013	0.00754842	0.0138819
ZNF682	1	1	9	0.11		6	423	0.01		2056	0.6950617	0.0073521	0.03115277
CDHR2	1	1	9	0.11		21	424	0.05		4094	0.695517158	0.00674022	0.01739143
CA2	1	1	9	0.11		5	424	0.01		946	0.696126017	0.00762912	0.02443956
PLEKHM2	1	1	9	0.11	Yes	5	423	0.01		4115	0.696356563	0.00683835	0.02558788
ARFGAP1	1	1	9	0.11		6	423	0.01		9285	0.697569043	0.00684453	0.01733082
PYCR2	1	1	9	0.11	Yes	3	423	0.01		6249	0.698107164	0.0069115	0.02463723
OR9K2	1	1	9	0.11		13	423	0.03		1011	0.698699006	0.00774693	0.02576552
PHGDH	1	1	9	0.11		4	423	0.01		5790	0.699155742	0.00694372	0.02323886
TGFBR2	1	1	9	0.11	Yes	10	779	0.01		2846	0.699580318	0.00731522	0.03354415
ZNF707	1	1	9	0.11						6278	0.699669145	0.00703536	0.02421045
TTC29	1	1	9	0.11	Yes	5	423	0.01		2957	0.699724556	0.00753561	0.03253921

OR4A15	1	1	9	0.11		43	424	0.1		1038	0.699900488	0.00780176	0.02648297
ODAM	1	1	9	0.11		8	423	0.02		921	0.700360099	0.00784551	0.02423254
STK3	1	1	9	0.11	Yes	6	779	0.01		5834	0.70037041	0.00752923	0.02619141
LILRA1	1	1	9	0.11		23	425	0.05		4892	0.701355284	0.00713128	0.02954352
NDC80	1	1	9	0.11		6	423	0.01		3002	0.70159213	0.00758081	0.01578062
TDG	1	1	9	0.11		13	566	0.02		2661	0.702783684	0.00766584	0.03466586
KIAA1804	1	1	9	0.11		15	645	0.02		3472	0.703502182	0.00755024	0.03112185
OR52E2	1	1	9	0.11	Yes	10	423	0.02		981	0.703629079	0.00799983	0.01505057
OR4K15	1	1	9	0.11		23	424	0.05		1050	0.704032811	0.00800678	0.0275338
OR6X1	1	1	9	0.11		8	423	0.02		942	0.704837069	0.00806959	0.02533753
TMEM53	1	1	9	0.11	Yes	3	423	0.01		6901	0.706163629	0.00712284	0.01945241
CENPB	1	1	9	0.11		4	432	0.01		2223	0.706383269	0.00783168	0.03613296
GTF2H1	1	1	9	0.11		6	566	0.01		3501	0.706751273	0.00773768	0.01744722
GABBR2	1	1	9	0.11		21	425	0.05		3252	0.706769168	0.00760799	0.0395979
DUOX2	1	1	9	0.11		24	424	0.06		4746	0.707234073	0.00713295	0.02009465
GZMM	1	1	9	0.11						789	0.70739989	0.00819632	0.02648107
OLFM4	1	1	9	0.11		19	425	0.04		3722	0.707505088	0.00782935	0.02783434
PPFIA2	1	1	9	0.11	Yes	34	431	0.08		12413	0.707649239	0.00618461	0.04150307
SCGB3A2	1	1	9	0.11		1	423	0		1450	0.707924743	0.00823463	0.02543633
RPS9	1	1	9	0.11	Yes	2	423	0		907	0.708317186	0.00820994	0.03030363
NOM1	1	1	9	0.11		6	423	0.01		3331	0.708962149	0.00776136	0.01895978
NR1D1	1	1	9	0.11	Yes	2	431	0		1869	0.709566865	0.00800878	0.03450754
RTL1	1	1	9	0.11		5	423	0.01		4080	0.710560092	0.0074256	0.01844492
XPC	1	1	9	0.11	Yes	5	566	0.01	Yes	4103	0.710908858	0.00751793	0.02418754
PCDH17	1	1	9	0.11	Yes	49	426	0.12		3541	0.711337178	0.00762733	0.02506422
TRPC3	1	1	9	0.11		13	424	0.03		3924	0.71190664	0.00766238	0.025855
BAIAP2	1	1	9	0.11	Yes	6	424	0.01		6073	0.712265855	0.00736997	0.02045815
OR6F1	1	1	9	0.11		34	423	0.08		930	0.712271084	0.00846834	0.02610416
PDE9A	1	1	9	0.11		6	423	0.01		5034	0.712593936	0.00751431	0.0187066
ELTD1	1	1	9	0.11		43	425	0.1		2249	0.713462855	0.00825959	0.03401382
USH1C	1	1	9	0.11		22	423	0.05		4126	0.713632172	0.0077478	0.03341864
LRRFIP2	1	1	9	0.11	Yes	4	423	0.01		5663	0.71369413	0.0080048	0.0211846
GALR1	1	1	9	0.11		12	557	0.02		1059	0.714167631	0.00851347	0.03241148
STXBP1	1	1	9	0.11	Yes	7	423	0.02		3692	0.714296791	0.00799104	0.03838424
GYPB	1	1	9	0.11						1358	0.714752774	0.0085739	0.02965045
BBS4	1	1	9	0.11		7	424	0.02		4047	0.71619285	0.00808552	0.02153691
PRDM9	1	1	9	0.11		71	429	0.17		3692	0.716288119	0.00795497	0.02324528
ERC2	1	1	9	0.11	Yes	10	423	0.02		14245	0.716457941	0.00746201	0.02903104
TBRG4	1	1	9	0.11		8	424	0.02		4985	0.717137221	0.00760834	0.02752575
OR52D1	1	1	9	0.11	Yes	7	425	0.02		960	0.718256676	0.00881157	0.02615316
IQUB	1	1	9	0.11		18	425	0.04		2861	0.71893232	0.00844569	0.02755732
KIAA0226L	1	1	9	0.11		7	423	0.02		3620	0.719024535	0.00817844	0.01369868

OR4C15	1	1	9	0.11		30	424	0.07		1116	0.719352502	0.00885446	0.02828499
MAG	1	1	9	0.11	Yes	11	557	0.02		2453	0.71937917	0.00842184	0.0324939
DARS	1	1	9	0.11	Yes	3	423	0.01		2719	0.720551568	0.00850519	0.03193571
LCK	1	1	9	0.11	Yes	13	779	0.02	Yes	3293	0.721733492	0.00825662	0.04244066
GHR	1	1	9	0.11		17	565	0.03		3616	0.722736739	0.00864995	0.02687279
MITF	1	1	9	0.11	Yes	5	557	0.01	Yes	4892	0.723283382	0.00825432	0.02547275
FAM19A5	1	1	9	0.11		6	423	0.01		1597	0.723812715	0.00905488	0.03472548
PIEZO2	1	1	9	0.11						9037	0.724071312	0.007107	0.03705838
DDX21	1	1	9	0.11	Yes	5	431	0.01		2562	0.724271924	0.00874825	0.04048196
OR13A1	1	1	9	0.11		8	423	0.02		990	0.724282614	0.00912956	0.03035261
MYO1E	1	1	9	0.11		9	423	0.02		5384	0.725052748	0.00804567	0.02804649
EBF1	1	1	9	0.11	Yes	16	424	0.04	Yes	7657	0.725267611	0.00830669	0.02711061
ADAD1	1	1	9	0.11		18	423	0.04		2927	0.725313274	0.00878316	0.02964794
GPR125	1	1	9	0.11		24	424	0.06		9258	0.726235287	0.0073998	0.03300025
OLFM3	1	1	9	0.11	Yes	20	424	0.05		5205	0.726812063	0.00870769	0.02151724
EHHADH	2	2	9	0.22		8	424	0.02		3873	0.726940026	0.00859263	0.02720245
CHST15	1	1	9	0.11	Yes	5	424	0.01		3664	0.727598539	0.00868343	0.04393761
COQ9	1	1	9	0.11	Yes	6	423	0.01		2219	0.727650949	0.00898378	0.03660706
SERPINA3	1	1	9	0.11		17	431	0.04		3607	0.730833182	0.00885536	0.03663791
STAT4	1	1	9	0.11	Yes	11	753	0.01		2979	0.731138587	0.00913376	0.04199259
SDHA	1	1	9	0.11		12	431	0.03		4850	0.731329543	0.00855204	0.01721141
SULF1	1	1	9	0.11	Yes	13	423	0.03		8255	0.731539643	0.00831102	0.02988642
TSG101	1	1	9	0.11	Yes	6	423	0.01		2736	0.732081329	0.009188	0.04221821
SHBG	1	1	9	0.11		2	423	0		2033	0.732500196	0.00930138	0.03405125
SLCO1A2	1	1	9	0.11	Yes	21	423	0.05		3670	0.732951195	0.00920991	0.02992158
CEP70	1	1	9	0.11	Yes	5	423	0.01		3566	0.733101212	0.00922055	0.03018935
EZH1	1	1	9	0.11	Yes	9	431	0.02		2503	0.733101212	0.00926283	0.04100892
DSC3	1	1	9	0.11		27	424	0.06		2769	0.733194422	0.00926598	0.02975596
TNFRSF11B	1	1	9	0.11		7	432	0.02		1262	0.733226369	0.00965611	0.03397647
NDST4	1	1	9	0.11	Yes	38	424	0.09		2883	0.733298722	0.00925239	0.0295644
MTA1	1	1	9	0.11		5	424	0.01		4730	0.733518661	0.0085778	0.03026736
INHBA	1	1	9	0.11	Yes	26	612	0.04		3696	0.733533709	0.00928751	0.04116383
TAAR5	1	1	9	0.11	Yes	15	423	0.04		1017	0.734870139	0.0098209	0.02898813
LRP1B	2	2	9	0.22	Yes	204	619	0.33		17772	0.736750839	0.07242122	0.06656737
GDF3	1	1	9	0.11		7	431	0.02		1101	0.736853664	0.00993338	0.03079225
PABPC3	1	1	9	0.11		16	423	0.04		3713	0.737025963	0.009125	0.03668919
POU6F2	1	1	9	0.11		16	423	0.04		6689	0.73762978	0.00881502	0.04004666
GLIPR2	1	1	9	0.11	Yes	2	423	0		4338	0.738809714	0.01003612	0.03340445
CASD1	1	1	9	0.11	Yes	12	423	0.03		2653	0.740216802	0.00977103	0.04094042
GALC	1	1	9	0.11		10	423	0.02		3898	0.741163769	0.00971613	0.04561966
GALNT5	1	1	9	0.11		18	433	0.04		3706	0.741523361	0.00967861	0.03251393
ALDH1L1	1	1	9	0.11		18	423	0.04		4430	0.741989054	0.0091701	0.02709864

PADI2	1	1	9	0.11	Yes	12	431	0.03		7913	0.742286254	0.00921657	0.0394249
ERLIN1	1	1	9	0.11	Yes					1267	0.74250434	0.01029092	0.03355274
CDYL2	1	1	9	0.11	Yes	8	423	0.02		2852	0.742821744	0.00981303	0.03341423
PCDHB12	1	1	9	0.11		35	424	0.08		5031	0.74354672	0.00887316	0.02529256
SOX10	1	1	9	0.11		4	431	0.01		2477	0.744552362	0.00990041	0.04875518
CDK17	1	1	9	0.11	Yes	11	645	0.02		3159	0.744690497	0.00992784	0.0202737
ABCA4	1	1	9	0.11		36	425	0.08		10052	0.745006665	0.00799618	0.04238984
OR51A7	1	1	9	0.11		13	424	0.03		2808	0.746125054	0.01011022	0.03981051
ACAA2	1	1	9	0.11	Yes	7	424	0.02		1224	0.74617049	0.0105599	0.03300553
C12orf40	1	1	9	0.11	Yes	14	424	0.03		5072	0.747276529	0.01005941	0.02063503
SLC26A7	1	1	9	0.11		22	423	0.05		6621	0.747344658	0.00993405	0.00981055
ZCCHC8	1	1	9	0.11		7	423	0.02		2858	0.747349365	0.01006059	0.05092902
EGR2	1	1	9	0.11	Yes	10	423	0.02		2968	0.748697048	0.01024554	0.02947595
C9orf142	1	1	9	0.11						6045	0.749682368	0.00938843	0.04422883
KRBA1	1	1	9	0.11	Yes	17	423	0.04		9702	0.749998466	0.00854397	0.03730157
PLIN4	1	1	9	0.11		14	423	0.03		4092	0.750405174	0.00978822	0.04918439
GPR158	1	1	9	0.11	Yes	48	424	0.11		8905	0.750583823	0.00948916	0.03189153
SLCO5A1	1	1	9	0.11		23	424	0.05		5645	0.750677986	0.00993612	0.05065082
POTEE	1	1	9	0.11						3453	0.751666387	0.01017742	0.02854122
RAD51AP1	1	1	9	0.11		5	432	0.01		3560	0.752071893	0.01049232	0.04419007
TECTB	1	1	9	0.11	Yes	9	423	0.02		1306	0.752296232	0.01099065	0.0350132
PAX1	1	1	9	0.11		21	431	0.05		3163	0.753110138	0.01030825	0.04258304
SLC35G5	2	2	9	0.22						1020	0.753508084	0.01112155	0.03207207
FBLN2	1	1	9	0.11	Yes	10	423	0.02		6141	0.754590558	0.00954964	0.03712235
SMARCAD1	1	1	9	0.11	Yes	13	432	0.03		3608	0.754816213	0.01053987	0.02238945
VSIG8	1	1	9	0.11	Yes	1	423	0		1266	0.754834992	0.0111525	0.03760182
CRTAM	1	1	9	0.11		7	423	0.02		1351	0.756055538	0.01126825	0.03604404
LIMK1	1	1	9	0.11	Yes					3123	0.756261264	0.01068537	0.05047923
TAF3	1	1	9	0.11		16	423	0.04		3257	0.756542828	0.01072985	0.03437438
SASH1	1	1	9	0.11	Yes	17	424	0.04		5318	0.757536795	0.00997678	0.03433455
TUBB2B	1	1	9	0.11		1	423	0		3173	0.757551594	0.01067751	0.02857451
FBXL21	1	1	9	0.11	Yes	4	431	0.01		4973	0.757621366	0.01076115	0.03599098
PRDM10	1	1	9	0.11		15	423	0.04	Yes	4610	0.758107335	0.01015059	0.04378199
GABRG1	1	1	9	0.11		33	424	0.08		1560	0.758280111	0.01139953	0.0394223
OBP2A	1	1	9	0.11		4	423	0.01		1260	0.758280111	0.01143446	0.03680966
NCAM2	1	1	9	0.11		40	426	0.09		4448	0.759029037	0.01066182	0.02762691
NDEL1	1	1	9	0.11		4	423	0.01		1534	0.759104878	0.01147643	0.03861154
INTU	1	1	9	0.11		14	423	0.03		4301	0.759380031	0.01066488	0.02750697
ANO2	1	1	9	0.11	Yes	20	423	0.05		7033	0.760813358	0.010343	0.04283787
CEP135	1	1	9	0.11		23	424	0.05	Yes	3634	0.760873772	0.01093794	0.03975653
LRRTM4	1	1	9	0.11	Yes	45	423	0.11		4027	0.761530462	0.01098655	0.03918246
TPO	1	1	9	0.11		32	431	0.07		6586	0.761711785	0.0100208	0.04005156

CPSF2	1	1	9	0.11		6	423	0.01		3348	0.762457135	0.01112655	0.02186617
CHCHD6	1	1	9	0.11		3	423	0.01		3848	0.762779596	0.01122515	0.04609032
FAM20B	1	1	9	0.11		6	423	0.01		1292	0.762887524	0.01180654	0.03708421
DDR2	1	1	9	0.11		2	779	0		3884	0.7643028	0.01096423	0.04247186
DLX2	1	1	9	0.11	Yes	6	423	0.01		1253	0.764417589	0.01182707	0.04478325
CCKAR	1	1	9	0.11		14	557	0.03		1302	0.765001363	0.01194291	0.04000761
AKNAD1	1	1	9	0.11		10	423	0.02		4471	0.765707327	0.01106345	0.01370501
SELL	1	1	9	0.11	Yes	5	423	0.01		3577	0.766610062	0.01138085	0.03535499
MTTP	1	1	9	0.11	Yes	17	423	0.04		3718	0.767329568	0.01152463	0.05003594
TTF1	1	1	9	0.11	Yes	10	423	0.02		3523	0.767937336	0.01148968	0.04099266
DMWD	1	1	9	0.11	Yes	4	423	0.01		2241	0.768393548	0.0122327	0.04005904
ZDHHC3	1	1	9	0.11		3	423	0.01		4318	0.76911884	0.01164617	0.05150971
ABCD1	1	1	9	0.11	Yes	5	431	0.01		3644	0.769479942	0.01142583	0.045908
CDK8	1	1	9	0.11	Yes	9	695	0.01		5644	0.770252274	0.01164865	0.0407966
CHSY1	1	1	9	0.11	Yes	5	424	0.01		6475	0.770446248	0.01113094	0.04209719
CHRM2	1	1	9	0.11		23	424	0.05		4526	0.770643963	0.01247248	0.03716809
ZFP90	1	1	9	0.11	Yes	9	423	0.02		4324	0.770959365	0.0118005	0.0510543
PBX1	1	1	9	0.11		1	565	0	Yes	16758	0.77122376	0.01177334	0.05451585
JMJD4	1	1	9	0.11		2	431	0		7773	0.77152846	0.01116552	0.05578508
SYT4	1	1	9	0.11	Yes	20	424	0.05		1560	0.771892783	0.01254205	0.04002208
OGDHL	1	1	9	0.11	Yes	28	424	0.07		6779	0.773553932	0.01109262	0.04778806
SLC9A3	1	1	9	0.11	Yes	16	423	0.04		2622	0.773594854	0.01203439	0.0557992
ZNF98	1	1	9	0.11		16	423	0.04	Yes	1731	0.774110476	0.01272034	0.04151539
C1orf129	1	1	9	0.11		15	423	0.04		3479	0.774299973	0.01205705	0.0539346
ATRX	1	1	9	0.11	Yes	33	435	0.08	Yes	21908	0.774441957	0.00796856	0.09650731
SCN5A	1	1	9	0.11	Yes	25	423	0.06		7343	0.774987976	0.0104343	0.03544556
C17orf72	1	1	9	0.11						1215	0.775164674	0.01289175	0.03656847
EFR3A	1	1	9	0.11	Yes	13	423	0.03		4232	0.775271925	0.01203448	0.042753
CCDC164	1	1	9	0.11	Yes	16	424	0.04		3465	0.775782685	0.01217908	0.036562
PLK1S1	1	1	9	0.11						7390	0.775836621	0.01176567	0.03391183
FAM73A	1	1	9	0.11	Yes	6	424	0.01		6429	0.776097843	0.01208766	0.04208057
PTPRJ	1	1	9	0.11		13	432	0.03		5185	0.77619987	0.01154636	0.02564137
FCGR2B	1	1	9	0.11					Yes	1305	0.777167712	0.01308184	0.01945515
CPZ	1	1	9	0.11		23	424	0.05		3685	0.777542341	0.01225458	0.04150098
ZCCHC17	1	1	9	0.11	Yes					1719	0.777722928	0.01308908	0.03931797
JPH2	1	1	9	0.11		18	425	0.04		2120	0.779556171	0.01313229	0.04773677
CNTNAP5	1	1	9	0.11	Yes	82	423	0.19		4559	0.779640883	0.0120222	0.03788499
EVPL	1	1	9	0.11		14	423	0.03		6168	0.78020517	0.01095348	0.04607808
ANKRD34B	1	1	9	0.11	Yes	13	423	0.03		1602	0.78048866	0.01331726	0.04162905
NPR2	1	1	9	0.11	Yes	10	459	0.02		4529	0.780522993	0.01197578	0.05532298
SOCS6	1	1	9	0.11	Yes	9	566	0.02		1611	0.780540262	0.01324163	0.04675717
CD5L	1	1	9	0.11	Yes	25	424	0.06		1576	0.780772207	0.01337176	0.03991007

IRAK3	1	1	9	0.11	Yes	17	779	0.02		1979	0.781003722	0.01334555	0.04299534
DNAJB5	1	1	9	0.11		4	431	0.01		3735	0.781640007	0.0126365	0.0392251
JAK2	1	1	9	0.11	Yes	20	1812	0.01	Yes	4837	0.782487242	0.01252361	0.02896652
FAM124B	1	1	9	0.11		7	423	0.02		1464	0.782536265	0.01354092	0.04012023
ZNF300	1	1	9	0.11		12	423	0.03		3746	0.783102568	0.01278496	0.0375178
DAGLA	1	1	9	0.11	Yes	15	425	0.04		3604	0.783634506	0.01250578	0.05098507
IPO7	1	1	9	0.11	Yes	11	431	0.03		4190	0.783639492	0.01275568	0.0415508
FRMD4A	1	1	9	0.11	Yes	9	431	0.02		12326	0.78398241	0.01193494	0.03648282
ULK4	1	1	9	0.11		3	457	0.01		6126	0.784450511	0.0123778	0.01668768
DRP2	1	1	9	0.11		9	431	0.02		3080	0.785033701	0.013007	0.0513165
PTGS2	1	1	9	0.11		10	432	0.02		6390	0.785255183	0.01251037	0.01927941
AQPEP	1	1	9	0.11	Yes					5409	0.78604686	0.01276285	0.05372928
PRKD3	1	1	9	0.11	Yes	9	646	0.01		4395	0.786066312	0.01299141	0.05837845
SH2D4A	1	1	9	0.11	Yes	7	423	0.02		1682	0.787536262	0.01395726	0.04542516
PAN2	1	1	9	0.11		17	566	0.03		4547	0.78757852	0.01266526	0.05044137
PUM1	1	1	9	0.11		13	424	0.03		12100	0.787703512	0.00994701	0.06144322
ZNF615	1	1	9	0.11		12	424	0.03		4088	0.787808665	0.01310046	0.04365376
NAV2	1	1	9	0.11	Yes	33	424	0.08		9738	0.788106623	0.01072811	0.04749548
ELF1	1	1	9	0.11	Yes	10	431	0.02		5420	0.788306654	0.01311389	0.04314054
INSR	1	1	9	0.11	Yes	22	781	0.03		4467	0.789140942	0.01273312	0.06187191
KIF25	1	1	9	0.11		7	423	0.02		1762	0.789494934	0.0141671	0.0449973
CCDC158	1	1	9	0.11	Yes	23	423	0.05		4257	0.790315041	0.01336968	0.06031559
OR8H1	1	1	9	0.11		18	425	0.04		1872	0.791138876	0.01429692	0.04753752
PCSK5	1	1	9	0.11	Yes	18	423	0.04		6741	0.791669526	0.01342908	0.06369171
SALL4	1	1	9	0.11		19	423	0.04		5602	0.792749728	0.01282213	0.04874432
GAS8	1	1	9	0.11	Yes	6	431	0.01		8344	0.793226101	0.01266362	0.05329652
AMBRA1	1	1	9	0.11	Yes	9	424	0.02		8649	0.794070319	0.01226491	0.04438796
PALB2	1	1	9	0.11		19	708	0.03	Yes	4105	0.79432971	0.01372361	0.06137624
MYH3	1	1	9	0.11		28	423	0.07		5940	0.794918999	0.01269261	0.05386251
GEMIN5	1	1	9	0.11	Yes	10	431	0.02		4611	0.795327927	0.01348222	0.04893826
SRPK1	1	1	9	0.11	Yes	6	457	0.01		4353	0.795358813	0.01380663	0.04470502
HRH4	1	1	9	0.11	Yes	10	423	0.02		1901	0.7953932	0.01474892	0.04793639
CDC42EP3	1	1	9	0.11	Yes	3	431	0.01		1570	0.795803593	0.014841	0.04528093
LARP4	1	1	9	0.11	Yes	7	423	0.02		4254	0.79675817	0.01393762	0.04379158
ARHGEF35	1	1	9	0.11		3	423	0.01		1458	0.797026327	0.01504491	0.0223363
G2E3	1	1	9	0.11	Yes	8	565	0.01		4961	0.797279872	0.01422025	0.0568181
SMARCA4	1	1	9	0.11	Yes	54	731	0.07	Yes	6375	0.798198175	0.01255023	0.0488092
WDR35	1	1	9	0.11		16	423	0.04		5035	0.798565243	0.01371829	0.03672662
ITGA8	1	1	9	0.11	Yes	52	432	0.12		4499	0.79863553	0.01410498	0.06416264
SOS1	1	1	9	0.11		27	754	0.04		5003	0.798969199	0.01384462	0.01695105
GBX2	1	1	9	0.11	Yes	2	423	0		2107	0.799677003	0.01509594	0.05532215
ZNF614	1	1	9	0.11		9	423	0.02		2135	0.800816755	0.01531038	0.05072175

ABL2	1	1	9	0.11	Yes	29	782	0.04	Yes	5165	0.801644183	0.01379904	0.03635757
AFF4	1	1	9	0.11		9	431	0.02		6435	0.802300783	0.01375192	0.04115748
IPO4	1	1	9	0.11	Yes	10	432	0.02		6056	0.802583248	0.01318177	0.07504213
ESAM	1	1	9	0.11		4	423	0.01		1643	0.803148904	0.01571113	0.02395663
PRDX6	1	1	9	0.11		5	423	0.01		2076	0.804022115	0.0157281	0.02859429
PAPD7	1	1	9	0.11		9	424	0.02		1749	0.80404333	0.01566005	0.05226269
KCNA6	1	1	9	0.11	Yes	17	424	0.04		1593	0.804128154	0.01566301	0.05264253
ADCY8	1	1	9	0.11		56	427	0.13		3899	0.804170371	0.01457824	0.06984951
PDGFRA	1	1	9	0.11	Yes	45	1157	0.04	Yes	4160	0.804323873	0.01465151	0.06664584
GATM	1	1	9	0.11		6	423	0.01		2291	0.804487957	0.01572423	0.05167077
STEAP4	1	1	9	0.11		5	423	0.01		1959	0.805057109	0.01580504	0.05100744
C6orf132	1	1	9	0.11	Yes					3633	0.805107374	0.01498916	0.05247704
HEATR4	1	1	9	0.11	Yes	7	424	0.02		5480	0.806008728	0.01429376	0.05761324
BRF1	1	1	9	0.11		5	423	0.01		5122	0.806749957	0.01435199	0.06396581
SPARC	1	1	9	0.11	Yes	5	423	0.01		1569	0.808045736	0.01622165	0.04834978
PIR	1	1	9	0.11		3	431	0.01		2754	0.808270845	0.0161793	0.03155999
PARP4	1	1	9	0.11		15	423	0.04		5663	0.80872045	0.01447864	0.04155099
DNA2	1	1	9	0.11		7	423	0.02		5374	0.808772634	0.0147039	0.04000047
DEF8	1	1	9	0.11	Yes	7	423	0.02		5387	0.809082486	0.01453621	0.06503327
PCDHGA1	1	1	9	0.11	Yes	32	424	0.08		5283	0.809119718	0.0143303	0.07429627
ARHGAP11B	1	1	9	0.11		1	423	0		4193	0.810577818	0.01645557	0.05264929
MYBPC1	1	1	9	0.11		19	424	0.04	Yes	6408	0.811630507	0.01513744	0.05376194
ABCB5	1	1	9	0.11		32	424	0.08		4573	0.812602888	0.01546236	0.07334921
ADD2	1	1	9	0.11		13	424	0.03		4859	0.812882003	0.01552836	0.07029517
TRPV3	1	1	9	0.11	Yes	12	423	0.03		4067	0.812961988	0.01540522	0.07726585
ABCB11	1	1	9	0.11	Yes	21	423	0.05		4272	0.812961988	0.01560176	0.06691404
TOR1AIP2	1	1	9	0.11		10	424	0.02		1824	0.813737832	0.01695098	0.0483152
CYP2A7	1	1	9	0.11		7	423	0.02		1705	0.814087797	0.01692472	0.05187573
MYO19	1	1	9	0.11		8	423	0.02		3666	0.81468102	0.01595428	0.05896995
GIT2	1	1	9	0.11		4	431	0.01		4275	0.815621751	0.01575884	0.07480417
DIP2C	1	1	9	0.11		22	424	0.05		6734	0.815832383	0.0144754	0.06724033
ZNF845	1	1	9	0.11		7	423	0.02		5506	0.816000652	0.01557687	0.03827306
ABCB4	1	1	9	0.11	Yes	28	424	0.07		4510	0.816287594	0.01587662	0.07276373
WDR26	1	1	9	0.11		6	424	0.01		4624	0.816322073	0.01582154	0.07580145
CLSPN	1	1	9	0.11		18	434	0.04		4984	0.817459926	0.01598266	0.05094575
NDE1	1	1	9	0.11	Yes	5	557	0.01		6644	0.818134155	0.0160693	0.07412973
RCSD1	1	1	9	0.11	Yes	2	423	0		4942	0.818165673	0.01748861	0.0511258
CADPS	1	1	9	0.11	Yes	32	611	0.05		8531	0.818264984	0.01431303	0.05276396
ZNF117	1	1	9	0.11		6	423	0.01		2582	0.818352572	0.01735135	0.05887207
ARHGEF5	1	1	9	0.11		8	432	0.02		7841	0.8186961	0.0141316	0.07006648
KIAA1239	1	1	9	0.11	Yes	1	423	0		5250	0.818794068	0.01593865	0.05714926
PTGER3	1	1	9	0.11	Yes	13	565	0.02		2396	0.820990673	0.01769606	0.03771757

USP47	1	1	9	0.11		13	566	0.02		9678	0.821032675	0.01586263	0.04368353
GRINA	1	1	9	0.11		2	423	0		3585	0.822087972	0.01680115	0.0619115
DGKK	1	1	9	0.11		15	431	0.03		7753	0.822560981	0.01661862	0.05286656
ZNF264	1	1	9	0.11	Yes	8	423	0.02		1896	0.823030685	0.01813876	0.05275109
MFSD9	1	1	9	0.11	Yes	6	423	0.01		2239	0.82374361	0.01817391	0.05584855
ABCD2	1	1	9	0.11		23	424	0.05		2253	0.823992013	0.01817346	0.03490045
NDST1	1	1	9	0.11		9	423	0.02		4976	0.824144134	0.0167572	0.07671735
PGM2	1	1	9	0.11		8	423	0.02		2321	0.825365455	0.01836191	0.03523591
CTDSPL	1	1	9	0.11	Yes					3209	0.825435411	0.01838763	0.05735235
ALDH3B1	1	1	9	0.11	Yes					9437	0.825523996	0.01688846	0.05976526
VWDE	1	1	9	0.11						5281	0.827172349	0.01711027	0.0338588
EXOC3	1	1	9	0.11	Yes	12	423	0.03		5412	0.827280316	0.01704811	0.08202385
KDM5B	1	1	9	0.11	Yes					6336	0.827362069	0.01669219	0.0661547
METTL13	1	1	9	0.11	Yes	16	423	0.04	Yes	7221	0.827600089	0.01587739	0.07905962
BAGE2	1	1	9	0.11						3308	0.828077002	0.01874489	0.05898187
EFTUD1	1	1	9	0.11		11	423	0.03		5135	0.828163902	0.01735535	0.0730172
LNX2	1	1	9	0.11	Yes	7	431	0.02		2100	0.828519369	0.0188422	0.05765654
FMN2	1	1	9	0.11	Yes	74	424	0.17		7324	0.828643142	0.01617052	0.08174889
ESR2	1	1	9	0.11	Yes	5	423	0.01		4524	0.828771868	0.01732578	0.0781616
PLXND1	1	1	9	0.11	Yes	22	423	0.05		8403	0.829591093	0.01530544	0.0807836
THSD7A	1	1	9	0.11		53	423	0.13		6315	0.829707658	0.01669881	0.06850064
TRIM21	1	1	9	0.11	Yes	8	431	0.02		1856	0.829885614	0.01918493	0.05189208
POPDC3	1	1	9	0.11		9	423	0.02		2792	0.829969365	0.01896042	0.06215584
KCNA4	1	1	9	0.11	Yes	35	423	0.08		2516	0.830636951	0.01920873	0.05590754
MPP6	1	1	9	0.11	Yes	5	424	0.01		2807	0.831234121	0.01915716	0.06211032
FANCL	1	1	9	0.11		4	566	0.01		2578	0.831975757	0.01931921	0.0601532
CHRM3	1	1	9	0.11	Yes	19	423	0.04		6149	0.832516247	0.01936397	0.06187186
ITIH1	1	1	9	0.11		18	423	0.04		4473	0.833445447	0.01804422	0.07433104
GPNMB	1	1	9	0.11		16	425	0.04		6660	0.834103381	0.01798942	0.05849527
TRMT2B	1	1	9	0.11		8	431	0.02		2835	0.834217264	0.01976054	0.03233072
NUP133	1	1	9	0.11	Yes	17	431	0.04		6045	0.834822236	0.0179666	0.06361919
GFRA2	1	1	9	0.11						2785	0.835001468	0.01971767	0.0637201
TSNAX	1	1	9	0.11		4	423	0.01		7385	0.835431006	0.01975175	0.06519632
KCNT2	1	1	9	0.11		38	425	0.09		8034	0.836094907	0.0181531	0.06143688
CDC5L	1	1	9	0.11	Yes	14	434	0.03		2457	0.836174401	0.01989565	0.03980487
C11orf63	1	1	9	0.11	Yes	13	424	0.03		2424	0.836410507	0.0199091	0.06531017
PLXNA2	1	1	9	0.11	Yes	19	424	0.04		11078	0.836753251	0.01642496	0.07562928
COL11A1	1	1	9	0.11		107	428	0.25		6749	0.83711283	0.01757544	0.06410675
ZNF567	1	1	9	0.11		6	423	0.01		5225	0.837795139	0.01876103	0.05217653
GLYATL1	1	1	9	0.11		19	423	0.04		4341	0.838156135	0.02027564	0.06221075
ZFP64	1	1	9	0.11	Yes	11	423	0.03		7463	0.838519762	0.01726633	0.06830109
MTHFD1L	1	1	9	0.11	Yes	8	431	0.02		5655	0.838843619	0.01867012	0.08070245

PLCL1	1	1	9	0.11	Yes	41	433	0.09	7167	0.839342917	0.01852397	0.06670469
EDNRB	2	1	9	0.11	Yes	16	557	0.03	2878	0.839537133	0.0203524	0.06849424
PLOD2	1	1	9	0.11		11	424	0.03	2817	0.839658967	0.02040015	0.04240804
SMYD1	1	1	9	0.11	Yes	16	431	0.04	2301	0.839932546	0.02054054	0.03852821
ANKRD36	1	1	9	0.11		3	423	0.01	6702	0.840415264	0.01828229	0.04766518
MTMR3	1	1	9	0.11	Yes				5200	0.84058029	0.01906415	0.07485432
HACE1	1	1	9	0.11	Yes	15	566	0.03	7066	0.841057942	0.01895101	0.08401311
DHX35	1	1	9	0.11	Yes	11	432	0.03	2333	0.841423863	0.02072266	0.06665663
SAMD9L	1	1	9	0.11	Yes	24	424	0.06	6814	0.841863074	0.01887053	0.06331446
ESF1	1	1	9	0.11	Yes	10	423	0.02	2931	0.841901122	0.0206936	0.07104571
GABRB3	1	1	9	0.11	Yes	39	423	0.09	3536	0.841960616	0.0207488	0.06929143
SLC18A1	1	1	9	0.11		10	423	0.02	2653	0.84314299	0.02123751	0.03251638
GPR179	1	1	9	0.11	Yes	14	424	0.03	8397	0.843586253	0.01729098	0.06460242
MSH4	1	1	9	0.11		21	566	0.04	2871	0.843976784	0.02109236	0.06974498
HAPLN1	1	1	9	0.11		13	423	0.03	2414	0.844340242	0.0212553	0.06599181
SLC39A12	2	2	9	0.22	Yes	35	424	0.08	2391	0.844601091	0.02139761	0.06235298
SLC22A12	1	1	9	0.11		9	423	0.02	1918	0.844716799	0.02143497	0.06172859
HMGXB3	1	1	9	0.11					4614	0.844792497	0.01974215	0.07355542
NDNF	1	1	9	0.11		10	424	0.02	2807	0.845250158	0.02146511	0.06427253
STAG2	1	1	9	0.11	Yes	19	433	0.04	6831	0.845875041	0.01972919	0.08558857
ASXL2	1	1	9	0.11	Yes	6	423	0.01	6776	0.845956112	0.01876619	0.06990679
KIAA1217	1	1	9	0.11	Yes	17	425	0.04	8917	0.846399929	0.01771065	0.07569759
HCN1	1	1	9	0.11	Yes	78	428	0.18	2697	0.846832762	0.02159757	0.07021084
CNGA3	1	1	9	0.11		17	423	0.04	2339	0.846875175	0.02172376	0.06577846
PRG4	1	1	9	0.11	Yes	33	423	0.08	8322	0.846933674	0.01801238	0.03193144
PNISR	1	1	9	0.11	Yes	7	423	0.02	11841	0.847171006	0.0196321	0.06753741
MYOM2	1	1	9	0.11		30	424	0.07	9586	0.847974148	0.01826302	0.05703623
UAP1	1	1	9	0.11	Yes	6	432	0.01	3781	0.848152858	0.02189866	0.06813691
HRNR	1	1	9	0.11		55	426	0.13	8559	0.848169115	0.01745692	0.08527444
SLCO1B3	1	1	9	0.11	Yes	18	424	0.04	3122	0.848431388	0.02184469	0.07206508
CKMT1A	2	1	9	0.11		3	423	0.01	2325	0.848847658	0.0220513	0.06733672
P4HA3	1	1	9	0.11	Yes	4	423	0.01	2607	0.848972184	0.02222797	0.06171437
SYNE2	2	2	9	0.22	Yes	55	426	0.13	27737	0.849470348	0.12977966	0.1411826
YLPM1	1	1	9	0.11		20	423	0.05	9332	0.849495112	0.01818121	0.06784927
BTAF1	1	1	9	0.11	Yes	15	424	0.04	7213	0.84983639	0.01944097	0.05064583
SMARCC1	1	1	9	0.11		14	434	0.03	6489	0.85005088	0.02025834	0.08668808
TBCD	1	1	9	0.11	Yes				8321	0.850693338	0.01877727	0.0732313
PNN	1	1	9	0.11	Yes	9	423	0.02	3117	0.850901971	0.02230779	0.07230159
ATP6V1A	1	1	9	0.11	Yes	14	424	0.03	2774	0.851023817	0.02244173	0.06831497
DNAH10	1	1	9	0.11		13	424	0.03	15220	0.851261251	0.01473185	0.09168511
COL7A1	1	1	9	0.11	Yes	47	423	0.11	9918	0.851266481	0.01680559	0.09783554
CXorf22	1	1	9	0.11		18	432	0.04	2993	0.851280527	0.02233142	0.07408796

PPP1R12A	1	1	9	0.11		3	431	0.01		6430	0.851446364	0.02017924	0.04708449
EIF3A	1	1	9	0.11		17	431	0.04		6830	0.851574419	0.01992467	0.10946296
CLK2	1	1	9	0.11		8	458	0.02		6720	0.851726637	0.02009513	0.07910354
SLIT2	1	1	9	0.11	Yes	48	429	0.11		6862	0.851750549	0.01992581	0.07805725
LMAN1L	1	1	9	0.11	Yes	7	423	0.02		1942	0.851992916	0.02287321	0.05965638
SYNE1	2	2	9	0.22	Yes	122	428	0.29		39858	0.852023936	0.08777822	0.17985551
LPHN3	1	1	9	0.11	Yes	65	607	0.11		8961	0.852074764	0.0190865	0.06312024
SCN11A	1	1	9	0.11	Yes	22	423	0.05		5963	0.852971546	0.0205594	0.09378164
RXFP1	1	1	9	0.11	Yes	22	423	0.05		3833	0.85382265	0.02286587	0.07284332
CLEC18C	1	1	9	0.11	Yes	1	423	0		2042	0.85519099	0.0233174	0.06672707
COL6A2	1	1	9	0.11	Yes	13	423	0.03		4548	0.856953657	0.02144023	0.06954266
NCOA2	1	1	9	0.11		23	565	0.04	Yes	7301	0.857282478	0.02021463	0.07961504
ADAM18	1	1	9	0.11		24	432	0.06		3661	0.857943124	0.0235862	0.07705191
EXTL3	1	1	9	0.11	Yes	7	423	0.02		8561	0.858187506	0.02138151	0.09663513
ARFGEF2	1	1	9	0.11	Yes	17	432	0.04		5870	0.858221523	0.02138588	0.09669575
LGI2	1	1	9	0.11		13	423	0.03		3038	0.859065634	0.02375328	0.07938064
DCAF11	1	1	9	0.11						5905	0.860045143	0.02188627	0.09039505
NXPH1	1	1	9	0.11	Yes	7	423	0.02		3081	0.860211008	0.02415551	0.07411693
TNS3	1	1	9	0.11		16	423	0.04		9786	0.860312053	0.01933928	0.10790617
GLB1L3	1	1	9	0.11		4	423	0.01		2672	0.860504221	0.02422122	0.07404204
ZRANB2	1	1	9	0.11		4	424	0.01		6298	0.860540805	0.02388617	0.08541855
GSG1	1	1	9	0.11		4	423	0.01		2390	0.8607235	0.0244246	0.06889286
MEIS2	1	1	9	0.11	Yes	13	423	0.03		9248	0.860835913	0.0218029	0.09859765
GGT1	1	1	9	0.11	Yes	6	423	0.01		5292	0.861854051	0.0219557	0.09979636
RIC8B	1	1	9	0.11	Yes	7	423	0.02		3109	0.862780037	0.02456524	0.07912761
CNTNAP4	1	1	9	0.11		27	423	0.06		9264	0.863412221	0.0207713	0.06405853
PLB1	1	1	9	0.11		27	424	0.06		10348	0.864138269	0.01911868	0.06995744
GC	1	1	9	0.11		17	424	0.04		3148	0.865102685	0.02529913	0.07242916
MECOM	1	1	9	0.11		23	753	0.03		6307	0.865463419	0.02264945	0.09925174
TRIM52	1	1	9	0.11		3	423	0.01		4417	0.866138272	0.02540578	0.07663393
CEP57L1	1	1	9	0.11	Yes	6	423	0.01		4005	0.86716157	0.02551654	0.05027188
TRPM1	1	1	9	0.11		40	427	0.09		6411	0.867318232	0.0227592	0.10777304
NOL6	1	1	9	0.11		8	424	0.02		5890	0.868187818	0.02342201	0.09113458
DACH2	1	1	9	0.11	Yes	25	432	0.06		3113	0.868429304	0.02591616	0.07756798
ATG2B	1	1	9	0.11		25	424	0.06		8911	0.868456903	0.02187482	0.06447672
ODZ3	1	1	9	0.11	Yes	48	423	0.11		12914	0.868994317	0.01924928	0.06249867
KCNC1	1	1	9	0.11	Yes	6	423	0.01		3270	0.869711332	0.02561549	0.0961539
ADAM29	1	1	9	0.11		23	567	0.04		6506	0.871146877	0.02663216	0.07596325
MAGEE1	1	1	9	0.11	Yes	20	431	0.05		2877	0.871416014	0.0266396	0.07774651
NPAT	1	1	9	0.11		19	423	0.04		9381	0.872136378	0.02299754	0.03148812
DPF1	1	1	9	0.11	Yes	4	423	0.01		3041	0.872621785	0.0264951	0.09098683
LRRCC1	1	1	9	0.11		16	424	0.04		3753	0.872674735	0.02652626	0.09045449

ETV1	1	1	9	0.11	Yes	12	565	0.02	Yes	4090	0.872695906	0.02694017	0.07836931
CECR1	1	1	9	0.11		7	424	0.02		3059	0.872801678	0.02705718	0.07570898
ME3	1	1	9	0.11	Yes	7	423	0.02		3779	0.872833384	0.02671923	0.08592048
PEX5L	1	1	9	0.11	Yes	23	423	0.05		3818	0.872970634	0.02688358	0.08209167
ATP6V1B1	1	1	9	0.11	Yes	5	423	0.01		2627	0.873012819	0.02682969	0.05227526
PPP1R9B	1	1	9	0.11	Yes	4	423	0.01		7652	0.873160297	0.02677853	0.08659913
ANLN	1	1	9	0.11		17	424	0.04		7243	0.873305914	0.02394377	0.10968088
ITGA7	1	1	9	0.11		16	433	0.04		5474	0.873643485	0.02438943	0.09639577
NEDD4L	1	1	9	0.11	Yes	11	565	0.02		3104	0.874713733	0.02715055	0.0872954
ADCY10	1	1	9	0.11		25	426	0.06		7920	0.875400228	0.02404128	0.08281739
CAD	1	1	9	0.11	Yes	34	433	0.08		9873	0.875885962	0.02124611	0.12852824
GAB4	1	1	9	0.11		22	425	0.05		2666	0.876278744	0.02797434	0.04253093
NFATC3	1	1	9	0.11	Yes	18	423	0.04		11977	0.876650758	0.02267652	0.06995286
PGBD2	1	1	9	0.11		6	423	0.01		3116	0.878378635	0.02827449	0.08294032
LRRK2	1	1	9	0.11	Yes	50	647	0.08		9132	0.87889384	0.02409527	0.06435899
SAMD9	1	1	9	0.11	Yes	25	424	0.06		8625	0.878946797	0.0245924	0.05832726
MOCS1	1	1	9	0.11		6	423	0.01		2992	0.879242471	0.02864405	0.04570803
UBQLN3	1	1	9	0.11		16	423	0.04		2637	0.8795251	0.02887199	0.07514677
FLT3	1	1	9	0.11		28	1475	0.02	Yes	3688	0.879787333	0.02841477	0.08977009
UBE4B	1	1	9	0.11	Yes	14	431	0.03		9149	0.880298209	0.0250477	0.12123966
PTPRG	1	1	9	0.11	Yes	14	620	0.02		12295	0.881252603	0.02506915	0.12707843
ZNF208	1	1	9	0.11		59	423	0.14		7271	0.881360603	0.02635592	0.06741095
PARD3B	1	1	9	0.11	Yes	21	423	0.05		8444	0.881875339	0.02564072	0.11418605
ZNF268	1	1	9	0.11		7	423	0.02		9682	0.882584028	0.02471051	0.06192922
EPHA3	1	1	9	0.11	Yes	61	830	0.07		3268	0.882867723	0.02938166	0.08713907
CBLL1	1	1	9	0.11	Yes	9	431	0.02	Yes	3225	0.883702186	0.02967745	0.08573847
POLR3C	1	1	9	0.11		4	423	0.01		3914	0.884061	0.0298156	0.08487902
ZNF621	1	1	9	0.11		8	423	0.02		3897	0.884116054	0.02987799	0.04886168
AK5	1	1	9	0.11	Yes	24	435	0.06		5099	0.884664428	0.02970749	0.09241566
LPPR4	1	1	9	0.11		35	424	0.08	Yes	3350	0.885027839	0.02999965	0.0875858
PIGR	1	1	9	0.11		13	424	0.03		2885	0.885064085	0.03008357	0.08567594
DHX57	1	1	9	0.11	Yes	18	431	0.04		8935	0.886010336	0.02623116	0.12544935
RABGEF1	1	1	9	0.11	Yes	8	432	0.02		5837	0.886854679	0.03034253	0.09297787
TRPA1	2	2	9	0.22	Yes	39	432	0.09		4341	0.887013771	0.03029711	0.09538725
ZNF365	1	1	9	0.11	Yes	7	423	0.02		7158	0.887234175	0.03062673	0.08867639
CHRNA3	1	1	9	0.11	Yes	5	423	0.01		4014	0.887269379	0.03040475	0.09462803
PXDNL	1	1	9	0.11		21	423	0.05		7503	0.887812731	0.02655427	0.12871845
FRY	1	1	9	0.11	Yes	36	425	0.08		11547	0.888949676	0.02402165	0.09885474
APPL2	1	1	9	0.11		9	423	0.02		3556	0.889454458	0.03100536	0.09660606
GOLGA4	1	1	9	0.11		12	423	0.03		12463	0.889553628	0.02622132	0.07197645
LY9	1	1	9	0.11		14	423	0.03		9873	0.889622919	0.02794798	0.10249469
BRAT1	1	1	9	0.11		7	431	0.02		3395	0.889769934	0.03138447	0.08964884

EIF2A	1	1	9	0.11	Yes	3	423	0.01		3930	0.889990814	0.03130094	0.05726241
ANKRD18A	1	1	9	0.11		3	423	0.01		6686	0.890202575	0.03100189	0.10258611
KIAA0494	1	1	9	0.11		4	423	0.01		3788	0.890320896	0.03148935	0.0550283
TIAM1	1	1	9	0.11		43	567	0.08		8254	0.890939695	0.02731156	0.09722343
ENPEP	1	1	9	0.11	Yes	21	424	0.05		4507	0.891227283	0.03155324	0.09707588
KIF1A	1	1	9	0.11	Yes	25	423	0.06		8107	0.891297273	0.02622274	0.11579424
PTPRN2	1	1	9	0.11		32	432	0.07		3301	0.892171949	0.03176374	0.09945546
OBSCN	2	2	9	0.22	Yes	73	457	0.16		44435	0.892279227	0.09077611	0.24693393
CHD2	1	1	9	0.11	Yes	14	432	0.03		14099	0.892604869	0.02782424	0.12721716
FAM189A2	1	1	9	0.11	Yes					4942	0.892729624	0.03232718	0.0902882
FCHSD2	1	1	9	0.11		5	432	0.01		3949	0.892803112	0.03196849	0.09953306
ALG3	1	1	9	0.11		6	423	0.01		3402	0.894049052	0.03275571	0.09071452
PCDP1	1	1	9	0.11	Yes					5593	0.894464902	0.0325434	0.09912117
RPS6KL1	1	1	9	0.11		4	457	0.01		3162	0.89449679	0.03292119	0.09043293
UBAP2	1	1	9	0.11	Yes	10	423	0.02		13812	0.895122084	0.02887575	0.11723647
BCKDHA	1	1	9	0.11	Yes	4	423	0.01		3690	0.895249946	0.03268581	0.10209202
DNAH17	1	1	9	0.11		25	423	0.06		13698	0.89541343	0.0223254	0.12318521
EPPK1	1	1	9	0.11		24	423	0.06		7266	0.895788242	0.02849554	0.13203051
PRLR	1	1	9	0.11		26	424	0.06		3645	0.896252184	0.0335229	0.09069683
GFM2	1	1	9	0.11	Yes	5	423	0.01		4331	0.897009957	0.03341605	0.09937744
TM7SF2	1	1	9	0.11		8	423	0.02		4087	0.89714825	0.03351013	0.05994216
RBL1	1	1	9	0.11		15	620	0.02		4289	0.897645362	0.03335752	0.06765575
LHX2	1	1	9	0.11	Yes	4	423	0.01		4212	0.89860646	0.0331298	0.11889312
DDX42	1	1	9	0.11	Yes	12	431	0.03		3725	0.898906412	0.03405147	0.10044288
KIAA1522	1	1	9	0.11	Yes	5	423	0.01		3461	0.899219957	0.03457386	0.09128046
ZNF292	1	1	9	0.11	Yes	21	423	0.05		11830	0.899246562	0.0287267	0.11203774
AGAP3	1	1	9	0.11	Yes	7	423	0.02		10541	0.899408281	0.0289935	0.14511267
MLH3	1	1	9	0.11	Yes	13	433	0.03		9164	0.900373874	0.02964132	0.1360675
CHD3	1	1	9	0.11	Yes	14	432	0.03		13593	0.900452377	0.02545934	0.15673396
CDH18	1	1	9	0.11		65	427	0.15	Yes	5760	0.900533023	0.03461954	0.10119469
CDK5RAP2	1	1	9	0.11		17	431	0.04		8701	0.90118194	0.03029288	0.12542507
TMEM161B	1	1	9	0.11	Yes	9	423	0.02		5945	0.901324711	0.03467733	0.06695348
KIAA0430	1	1	9	0.11		13	425	0.03		9261	0.901453489	0.02975453	0.14077727
CACNA1H	1	1	9	0.11		23	423	0.05		14010	0.901527675	0.0250364	0.13227223
BTN2A2	1	1	9	0.11		11	424	0.03		3387	0.902228399	0.03556704	0.0946109
TMEM132C	1	1	9	0.11	Yes	1	423	0		3597	0.902285366	0.03517472	0.10361669
KIF20B	1	1	9	0.11		15	425	0.04		11216	0.902352155	0.03030357	0.10075718
KIF2B	1	1	9	0.11		49	424	0.12		3720	0.903127039	0.03547569	0.10416109
ASTE1	1	1	9	0.11	Yes	6	423	0.01		4239	0.903211324	0.03552596	0.10378734
IFI44	1	1	9	0.11		10	423	0.02		4904	0.903728718	0.03546289	0.10948694
C6	1	1	9	0.11	Yes	43	425	0.1		4369	0.904283527	0.03602458	0.10218044
PARP9	1	1	9	0.11		10	557	0.02		3985	0.904324949	0.03614296	0.10001813

ATP13A3	1	1	9	0.11		10	424	0.02		4675	0.904751451	0.03584847	0.0691968
SDK1	1	1	9	0.11		46	426	0.11		9689	0.905004041	0.03024018	0.15475645
OTOF	1	1	9	0.11		31	425	0.07		7665	0.905913586	0.03098639	0.14362629
POM121	1	1	9	0.11		12	432	0.03		4003	0.90729276	0.03723049	0.10274174
RTN4	1	1	9	0.11		7	423	0.02		12589	0.907349857	0.03113929	0.11049915
TPTE	1	1	9	0.11		66	612	0.11		4987	0.907470493	0.03660216	0.11716492
METTL3	1	1	9	0.11		5	424	0.01	Yes	4774	0.907765617	0.03737281	0.10391253
MYO15A	1	1	9	0.11		26	423	0.06		14733	0.908000826	0.02728547	0.15801537
AARS	1	1	9	0.11	Yes	5	423	0.01		3765	0.908700887	0.03769117	0.0630975
INPP4B	1	1	9	0.11	Yes	25	432	0.06		6859	0.90956527	0.03771334	0.11259122
WDPCP	1	1	9	0.11	Yes	19	423	0.04		5840	0.909609766	0.03770981	0.11304692
REV3L	1	1	9	0.11		20	435	0.05		12872	0.909879686	0.03061635	0.05245984
SP140	1	1	9	0.11		5	423	0.01		4401	0.91003406	0.03828169	0.10533547
CDC42BPA	1	1	9	0.11	Yes	28	646	0.04		12319	0.910045621	0.0318162	0.11674276
NRXN1	1	1	9	0.11	Yes	29	423	0.07		12005	0.910062771	0.0315429	0.16143885
KDR	1	1	9	0.11	Yes	58	920	0.06	Yes	4161	0.910867827	0.03837373	0.11080676
MDGA2	1	1	9	0.11	Yes	38	425	0.09		5179	0.911524364	0.03829215	0.11813657
EPB41L3	1	1	9	0.11		41	424	0.1		4485	0.911641308	0.03836936	0.11764494
ABCA5	1	1	9	0.11	Yes	17	423	0.04		5177	0.911868401	0.03838457	0.07621377
PAMR1	1	1	9	0.11		8	423	0.02		3879	0.912307907	0.03910932	0.10910729
PIK3R6	1	1	9	0.11	Yes					4643	0.91241729	0.03930757	0.06225202
CENPE	1	1	9	0.11		30	437	0.07		9465	0.912623908	0.03468177	0.13119608
PIK3AP1	1	1	9	0.11		24	424	0.06		4461	0.912901068	0.0391215	0.0702957
TTC28	1	1	9	0.11	Yes					8656	0.913226744	0.03359108	0.10116055
NFATC2	1	1	9	0.11	Yes	21	424	0.05		3743	0.913470575	0.03904313	0.07689022
SLC13A5	1	1	9	0.11		9	423	0.02		3753	0.913732534	0.03974001	0.109661
SETX	1	1	9	0.11	Yes	26	424	0.06	Yes	13643	0.91407986	0.03333949	0.15466185
LEPRE1	1	1	9	0.11		15	423	0.04		4523	0.914388344	0.03984892	0.1134465
CAMKV	1	1	9	0.11	Yes	1	645	0		3780	0.914405969	0.04013719	0.10816682
TTI1	1	1	9	0.11	Yes	13	423	0.03		4266	0.914411843	0.04006608	0.10956001
CDH23	1	1	9	0.11	Yes	41	423	0.1		21414	0.915793731	0.02330902	0.14838802
CENPF	1	1	9	0.11		31	423	0.07		12171	0.915904454	0.03366857	0.07898484
MAML1	1	1	9	0.11	Yes	14	753	0.02		8420	0.915908205	0.04068421	0.11147297
UNC5A	1	1	9	0.11	Yes	14	423	0.03		4061	0.916176757	0.04058905	0.1156545
CHRD	1	1	9	0.11	Yes	22	424	0.05		4098	0.916614082	0.0410184	0.11170369
IFT80	1	1	9	0.11		10	558	0.02		6805	0.916732786	0.0404958	0.1223703
DLG2	1	1	9	0.11	Yes	29	424	0.07		7884	0.917143551	0.0406447	0.12332952
ANXA11	1	1	9	0.11	Yes	6	423	0.01		5793	0.917378571	0.04132242	0.11310054
RNF216	1	1	9	0.11		8	431	0.02		5541	0.918281718	0.04147467	0.11857279
ST7	1	1	9	0.11		5	424	0.01		7257	0.918408021	0.04144454	0.12026463
POLK	1	1	9	0.11	Yes	11	432	0.03		6578	0.919316809	0.04156352	0.12640427
FBN2	1	1	9	0.11	Yes	73	424	0.17		9975	0.919547489	0.0350853	0.15729232

PHTF1	1	1	9	0.11		7	423	0.02		9278	0.919634435	0.04168289	0.1271749
TMPRSS3	1	1	9	0.11		2	423	0		4959	0.919661271	0.04226745	0.11707439
ACTN2	1	1	9	0.11	Yes	44	423	0.1		4931	0.919741702	0.04162988	0.12909059
EXT2	1	1	9	0.11		15	557	0.03	Yes	5628	0.920142143	0.04211167	0.12424247
AFF2	1	1	9	0.11		35	432	0.08		4479	0.920871577	0.04287367	0.07029261
XPO4	1	1	9	0.11		10	433	0.02		5437	0.920929313	0.04230554	0.12807219
DUSP27	1	1	9	0.11		41	425	0.1		4301	0.921065542	0.04305874	0.1162338
COL6A5	1	1	9	0.11	Yes	19	423	0.04		11285	0.923068527	0.03614945	0.12283605
FAM5C	1	1	9	0.11		84	435	0.19		5535	0.923205767	0.04345652	0.12937533
USP32	1	1	9	0.11	Yes	19	566	0.03		4957	0.923756646	0.04398874	0.12556788
LAMA2	1	1	9	0.11		69	425	0.16		11111	0.923950648	0.03628052	0.16827378
SLC22A2	1	1	9	0.11	Yes	15	423	0.04		5841	0.924366047	0.04403692	0.13049474
TMCC2	1	1	9	0.11	Yes	3	424	0.01		6383	0.925167956	0.0444612	0.13102207
ZBTB17	1	1	9	0.11		4	424	0.01		11899	0.92589687	0.03355775	0.16411354
PDE3A	1	1	9	0.11	Yes	26	424	0.06		5185	0.925938821	0.04495056	0.13031015
COL12A1	1	1	9	0.11		59	427	0.14		12832	0.926124281	0.03499752	0.14646767
RB1CC1	1	1	9	0.11	Yes	18	425	0.04	Yes	6643	0.926849209	0.0449427	0.08982139
VPS13C	1	1	9	0.11		38	423	0.09		12320	0.926980355	0.0370599	0.13166638
PPP1R9A	1	1	9	0.11		21	433	0.05		5010	0.9273125	0.04599329	0.12664843
ITGAD	1	1	9	0.11		36	433	0.08		4138	0.927544922	0.04640899	0.07164567
TRAF3IP2	1	1	9	0.11		5	431	0.01		6116	0.928382206	0.04689074	0.07175608
NUP155	1	1	9	0.11		22	431	0.05		5795	0.931186951	0.04757477	0.13978573
PCDH18	1	1	9	0.11	Yes	20	423	0.05		5495	0.931286115	0.04785501	0.13650923
ESYT1	1	1	9	0.11		8	423	0.02		4533	0.931423751	0.04832148	0.13081469
IGSF22	1	1	9	0.11	Yes	17	425	0.04		5078	0.933566581	0.04900752	0.14201547
SPHKAP	1	1	9	0.11		62	437	0.14		5139	0.934779908	0.05023454	0.13643894
C20orf194	1	1	9	0.11		13	424	0.03		5899	0.935031645	0.05042484	0.1362754
COL1A2	1	1	9	0.11		42	425	0.1		4328	0.935174893	0.05123719	0.12612129
PRRC2C	1	1	9	0.11						18419	0.935850414	0.03455568	0.14443175
MAGEC1	1	1	9	0.11	Yes	54	433	0.12		4785	0.936095461	0.05193022	0.12588273
PLCG2	1	1	9	0.11	Yes	18	753	0.02		6236	0.936165525	0.0499283	0.15490354
CSF2RB	1	1	9	0.11		17	423	0.04		4275	0.936729935	0.05224074	0.07226902
PKD1L3	1	1	9	0.11		3	423	0.01		5385	0.937024839	0.05165715	0.13940096
LMO7	1	1	9	0.11	Yes	11	557	0.02		12984	0.937052571	0.04084608	0.19376128
NOC2L	1	1	9	0.11	Yes	5	423	0.01		7943	0.937211521	0.05123595	0.14720504
DDX60	1	1	9	0.11		19	423	0.04	Yes	6782	0.938185742	0.05126249	0.15688137
ZCCHC6	1	1	9	0.11	Yes	12	423	0.03		6500	0.939201219	0.05263025	0.09252528
FAM65A	1	1	9	0.11		12	423	0.03		10795	0.939469046	0.04369439	0.16820528
NALCN	1	1	9	0.11		63	426	0.15		7626	0.939990786	0.05244677	0.15953037
COL5A2	1	1	9	0.11	Yes	45	424	0.11		4836	0.941136895	0.05518246	0.07705006
PHF20	1	1	9	0.11	Yes	10	423	0.02		6900	0.941147335	0.05390879	0.15236383
APBA2	1	1	9	0.11	Yes	30	428	0.07		4696	0.941185592	0.05439763	0.08853353

HELZ	1	1	9	0.11	Yes	21	424	0.05		6128	0.942832563	0.05527473	0.15282728
COL6A3	1	1	9	0.11	Yes	52	425	0.12		17712	0.944158703	0.04346822	0.21357678
VPRBP	1	1	9	0.11	Yes	12	423	0.03		5813	0.944346576	0.05661006	0.15257334
NTRK3	1	1	9	0.11	Yes	56	1555	0.04	Yes	8287	0.944501078	0.05657995	0.15465534
AXDND1	1	1	9	0.11		26	424	0.06		8451	0.945937205	0.05723841	0.16247526
KIF16B	1	1	9	0.11	Yes	22	433	0.05		7059	0.946135165	0.05721301	0.16498573
COL24A1	1	1	9	0.11	Yes	48	424	0.11		8459	0.94647007	0.05874403	0.08860944
NRXN3	1	1	9	0.11	Yes	33	424	0.08		8664	0.94676271	0.05749592	0.10799301
NRP2	1	1	9	0.11	Yes	17	423	0.04		8215	0.946847657	0.05807751	0.1014548
UBR4	1	1	9	0.11		35	432	0.08		25608	0.947149277	0.03350078	0.17843887
CHD6	1	1	9	0.11	Yes	40	432	0.09		15399	0.947368536	0.0458153	0.20755207
SCN10A	1	1	9	0.11	Yes	44	424	0.1		5952	0.947991497	0.05945928	0.15912087
ELMO2	1	1	9	0.11	Yes	7	423	0.02		6963	0.948035618	0.05933206	0.16111835
ADCY9	1	1	9	0.11		9	424	0.02		6343	0.948754966	0.05850042	0.17901249
CCDC168	1	1	9	0.11						7362	0.948803983	0.05921426	0.10919938
ASCC3	1	1	9	0.11	Yes	33	426	0.08		7301	0.949062437	0.05937696	0.17232485
HSPA8	1	1	9	0.11	Yes	13	432	0.03		6667	0.949088186	0.05991182	0.16644083
BSN	1	1	9	0.11		35	426	0.08		11811	0.949423818	0.0482644	0.19182225
SLC17A9	1	1	9	0.11	Yes	7	425	0.02		7749	0.949630547	0.0612257	0.1576698
FILIP1	1	1	9	0.11	Yes	21	425	0.05		7691	0.94968689	0.05976939	0.17495614
NCAPD3	1	1	9	0.11		23	431	0.05		9702	0.949832987	0.06061693	0.10368434
ADAMTSL3	1	1	9	0.11		41	566	0.07		7143	0.950053858	0.06121697	0.09892416
BBX	1	1	9	0.11	Yes	17	424	0.04		7769	0.950403447	0.06056644	0.17415288
SCN3A	1	1	9	0.11	Yes	44	425	0.1		7575	0.951190312	0.06130702	0.17500439
MYH6	1	1	9	0.11		52	425	0.12		5931	0.951721637	0.06181582	0.1755965
ERBB4	1	1	9	0.11		63	834	0.08		6897	0.951777536	0.06221797	0.1071622
GOLGB1	1	1	9	0.11		30	425	0.07		19453	0.952135101	0.0435481	0.14662304
KIAA0947	1	1	9	0.11		11	423	0.03		7319	0.952457311	0.0627443	0.10901857
LAMA5	1	1	9	0.11		35	423	0.08		13918	0.953474436	0.04766897	0.238611
TROAP	1	1	9	0.11	Yes	14	423	0.03		5507	0.954139216	0.0662352	0.15732058
LCT	1	1	9	0.11		55	424	0.13		6543	0.955693228	0.06622051	0.17679434
MYH7	1	1	9	0.11		51	424	0.12		6333	0.956174653	0.06576989	0.18736243
ACSS2	1	1	9	0.11	Yes	7	432	0.02		8184	0.956796676	0.06810871	0.101885
HERC2	1	1	9	0.11	Yes	73	435	0.17		15953	0.957269873	0.04909172	0.25635003
DDX11	1	1	9	0.11		21	433	0.05		6536	0.957638703	0.06882672	0.10490796
NINL	1	1	9	0.11	Yes	21	425	0.05	Yes	7534	0.95886066	0.06938345	0.11473902
RASGRF1	1	1	9	0.11		21	567	0.04		8006	0.958877902	0.06804104	0.12905804
ABCA12	1	1	9	0.11		41	425	0.1		8170	0.959116163	0.06888853	0.19353317
WDR11	1	1	9	0.11		7	432	0.02		9426	0.959203712	0.06892533	0.19430339
MPRIP	1	1	9	0.11		9	424	0.02		12444	0.95939927	0.06904041	0.19571295
ANKMY1	1	1	9	0.11		13	423	0.03		6893	0.959462773	0.06976241	0.18963585
CLASP2	1	1	9	0.11		11	423	0.03		8990	0.960040949	0.06963064	0.1983805

EXPH5	1	1	9	0.11		30	424	0.07		18636	0.960631617	0.0516499	0.19989686
RFX7	1	1	9	0.11	Yes	7	423	0.02		7921	0.960764814	0.07137893	0.19147702
DNAH1	1	1	9	0.11		24	423	0.06		14730	0.960902788	0.0527236	0.24790096
LRIG1	1	1	9	0.11		10	431	0.02		9844	0.961157349	0.07126236	0.19774357
ABCC8	1	1	9	0.11	Yes	32	424	0.08		6829	0.961833517	0.07223196	0.19784437
SIPA1L1	1	1	9	0.11	Yes	23	424	0.05		11714	0.962477239	0.07311315	0.19853293
DOCK8	1	1	9	0.11		17	423	0.04		8462	0.963140564	0.0735488	0.1294311
EP300	1	1	9	0.11	Yes	28	581	0.05	Yes	7338	0.963513571	0.07560931	0.11473763
PITRM1	1	1	9	0.11		4	423	0.01		10239	0.964905086	0.07441765	0.2201665
FAM186A	1	1	9	0.11						7861	0.965062407	0.07779997	0.19361428
CSMD1	1	1	9	0.11	Yes					27037	0.965353823	0.04343874	0.30261699
SEC16A	1	1	9	0.11	Yes	14	423	0.03		17982	0.965617959	0.0548716	0.27268417
ATP13A2	1	1	9	0.11		11	425	0.03		6918	0.966125165	0.07770615	0.20955179
FAT2	1	1	9	0.11		48	424	0.11		16519	0.966161522	0.05690464	0.25630363
GLI1	1	1	9	0.11		20	431	0.05		6721	0.966182049	0.08054739	0.10705295
XRN1	1	1	9	0.11	Yes	15	425	0.04		10327	0.967918974	0.0791178	0.22389921
ZNFX1	1	1	9	0.11	Yes	14	423	0.03		8390	0.96808243	0.08143232	0.20793625
FGD5	1	1	9	0.11	Yes	21	432	0.05		8344	0.97019618	0.08366377	0.14023394
KIAA1549	1	1	9	0.11					Yes	8854	0.970487214	0.08230104	0.23731268
CCDC88A	1	1	9	0.11		21	423	0.05		15268	0.972288347	0.08459281	0.24809583
SALL3	1	1	9	0.11	Yes	33	426	0.08		13427	0.972590841	0.08098656	0.19846957
ZNF135	1	1	9	0.11		25	425	0.06		10419	0.973117405	0.08883143	0.23127112
TRIO	1	1	9	0.11	Yes	30	458	0.07		12787	0.973278944	0.08678035	0.24819737
MCM3AP	1	1	9	0.11		20	433	0.05		9247	0.973492022	0.08902677	0.23601489
RIN3	1	1	9	0.11		18	558	0.03		11518	0.974616585	0.09181815	0.23565994
ATP7B	1	1	9	0.11		19	425	0.04		9185	0.974833562	0.09240228	0.23542683
SH3PXD2A	1	1	9	0.11	Yes	13	431	0.03		8888	0.97497654	0.09134535	0.24486038
NSD1	1	1	9	0.11	Yes	21	423	0.05	Yes	10337	0.975193047	0.09213085	0.24330806
MLLT4	1	1	9	0.11		15	566	0.03	Yes	10957	0.975318912	0.08941084	0.26340885
C5orf42	1	1	9	0.11	Yes	21	424	0.05		12030	0.976312467	0.09238502	0.26068121
VPS13A	1	1	9	0.11		37	424	0.09		13338	0.976798782	0.09171988	0.27323071
MEGF8	1	1	9	0.11	Yes	17	423	0.04		8718	0.976862953	0.09544282	0.15916166
FER1L5	1	1	9	0.11	Yes	6	423	0.01		9309	0.977083777	0.09709979	0.15144332
FREM2	1	1	9	0.11	Yes	33	425	0.08		10305	0.977284819	0.09557543	0.25739688
SRRM2	1	1	9	0.11	Yes	31	423	0.07		36226	0.978191557	0.03907262	0.39407483
APC	1	1	9	0.11		36	1714	0.02	Yes	13207	0.979293209	0.09830996	0.27703295
LRRK1	1	1	9	0.11		22	645	0.03		11817	0.979582328	0.09988989	0.17813754
ATM	1	1	9	0.11	Yes	54	858	0.06	Yes	13559	0.979703076	0.09835282	0.19047174
IL16	1	1	9	0.11		20	431	0.05		10567	0.979812478	0.10418843	0.25281604
DYSF	1	1	9	0.11	Yes	51	425	0.12		10770	0.98029206	0.10275721	0.17298675
DYNC2H1	1	1	9	0.11	Yes	8	423	0.02		13664	0.98059485	0.09928368	0.29556558
RELN	1	1	9	0.11	Yes	69	423	0.16	Yes	12405	0.981310081	0.10441642	0.28073213

SOGA1	1	1	9	0.11	Yes	16	423	0.04		11425	0.981782527	0.10505929	0.18766275
TRPM3	1	1	9	0.11	Yes	36	425	0.08		12288	0.982305826	0.1057709	0.29322928
FNDC1	1	1	9	0.11	Yes	20	423	0.05		11801	0.983644342	0.10806416	0.30871321
DCHS2	2	1	9	0.11		49	424	0.12		14506	0.984667428	0.10941545	0.3235463
BCL11A	1	1	9	0.11		22	566	0.04	Yes	15309	0.984689833	0.10635363	0.33904454
SALL2	1	1	9	0.11	Yes	11	423	0.03		11267	0.98475619	0.11957643	0.27660908
DNAH3	1	1	9	0.11		49	425	0.12		13913	0.986301993	0.11770146	0.32097941
DOCK4	1	1	9	0.11		27	423	0.06		16286	0.988084818	0.12098021	0.3493869
FAT4	1	1	9	0.11	Yes	93	427	0.22		15311	0.988136179	0.12258993	0.3439115
CSMD2	1	1	9	0.11	Yes	91	427	0.21		15908	0.988430869	0.12678359	0.33406469
EEF1D	1	1	9	0.11	Yes	2	432	0		13942	0.989399803	0.12667784	0.35986023
LRP2	1	1	9	0.11		79	426	0.19		15776	0.989627242	0.12855566	0.3586156
ACAN	1	1	9	0.11		37	423	0.09		13481	0.990196914	0.13877599	0.3359811
RYR1	1	1	9	0.11	Yes	85	426	0.2		15769	0.990929737	0.13124972	0.3847286
FAT3	1	1	9	0.11	Yes	31	423	0.07		18361	0.992299743	0.14009107	0.27153211
PRKDC	1	1	9	0.11		49	780	0.06		20460	0.992544306	0.13956725	0.40497389
PTPRF	1	1	9	0.11	Yes	23	431	0.05		23217	0.99484762	0.15076653	0.45275368
SETD2	1	1	9	0.11		22	572	0.04	Yes	25313	0.995858877	0.15663312	0.47946582
KALRN	1	1	9	0.11		25	432	0.06		26646	0.995898114	0.16425086	0.46225318
PCLO	1	1	9	0.11	Yes	94	423	0.22		23839	0.996188682	0.16891159	0.46476064
NBEAL2	1	1	9	0.11		9	423	0.02		19531	0.996254569	0.16864476	0.46861632
AHNAK2	1	1	9	0.11	Yes	12	423	0.03		19794	0.996441103	0.17748981	0.45753568
SACS	1	1	9	0.11	Yes	44	434	0.1		29036	0.996832106	0.16061197	0.37865034
HSPG2	1	1	9	0.11	Yes	37	426	0.09		23077	0.997098071	0.17356801	0.5017685
MUC12	1	1	9	0.11					Yes	32497	0.99917285	0.20012419	0.61622173
ARMC8	2	1	9	0.11	Yes					3558	1	1	1
FAM22F	2	2	9	0.22		2	423	0		3541	1	1	1
FCGR1A	2	2	9	0.22						2618	1	1	1
LHFPL3	2	2	9	0.22		6	423	0.01	Yes	1392	1	1	1
OR2L2	2	2	9	0.22						942	1	1	1
MUC16	1	1	9	0.11		180	557	0.32	Yes	50598	1	1	1
MUC5B	1	1	9	0.11		67	423	0.16		44057	1	1	1
KIAA1109	1	1	9	0.11	Yes	58	425	0.14		35907	1	1	1
WDFY4	1	1	9	0.11						26184	1	1	1
MTOR	1	1	9	0.11		40	592	0.07		12437	1	1	1
MYH2	1	1	9	0.11		79	426	0.19		12095	1	1	1
ZEB1	1	1	9	0.11		36	425	0.08		10940	1	1	1
FLNB	1	1	9	0.11		23	557	0.04		9670	1	1	1
RASA4	1	1	9	0.11		1	565	0		8927	1	1	1
CRB1	1	1	9	0.11		53	424	0.13		8188	1	1	1
LY75	1	1	9	0.11	Yes					7928	1	1	1
POLR1A	1	1	9	0.11		15	424	0.04		7619	1	1	1

ZFAT	1	1	9	0.11		16	424	0.04		7618	1	1	1
IFT172	1	1	9	0.11						7245	1	1	1
MYH11	1	1	9	0.11					Yes	6960	1	1	1
WDR64	1	1	9	0.11		16	423	0.04		6822	1	1	1
SCN9A	1	1	9	0.11		30	423	0.07		6817	1	1	1
ZNF841	1	1	9	0.11		3	423	0.01		6726	1	1	1
ZHX1	1	1	9	0.11	Yes					6516	1	1	1
NBPF14	1	1	9	0.11		13	423	0.03		6254	1	1	1
RORC	1	1	9	0.11		5	431	0.01		5952	1	1	1
FCAMR	1	1	9	0.11		3	423	0.01		5949	1	1	1
PAPOLA	1	1	9	0.11		6	424	0.01		5920	1	1	1
PRKCZ	1	1	9	0.11						5537	1	1	1
FBXW10	1	1	9	0.11		11	423	0.03		5510	1	1	1
FAM22D	1	1	9	0.11		2	423	0		5343	1	1	1
PLAA	1	1	9	0.11		3	423	0.01		4967	1	1	1
KIAA2022	1	1	9	0.11		32	433	0.07		4921	1	1	1
KRT8	1	1	9	0.11						4860	1	1	1
ATXN3	1	1	9	0.11		6	557	0.01		4762	1	1	1
ROBO4	1	1	9	0.11		38	425	0.09		4670	1	1	1
C11orf9	1	1	9	0.11		12	423	0.03		4607	1	1	1
FLRT3	1	1	9	0.11	Yes					4466	1	1	1
TXNRD1	1	1	9	0.11		5	423	0.01		4265	1	1	1
STXBP5L	1	1	9	0.11		26	425	0.06		4260	1	1	1
ZNF816	1	1	9	0.11						4239	1	1	1
HIPK3	1	1	9	0.11		10	458	0.02		4166	1	1	1
ARNTL	1	1	9	0.11		4	423	0.01	Yes	3780	1	1	1
NMNAT3	1	1	9	0.11		4	423	0.01		3501	1	1	1
EXOC7	1	1	9	0.11						3425	1	1	1
ETAA1	1	1	9	0.11		8	423	0.02		3325	1	1	1
OPCML	1	1	9	0.11		17	424	0.04		3272	1	1	1
IMPDH2	1	1	9	0.11		2	432	0		3204	1	1	1
CWC22	1	1	9	0.11		7	423	0.02		3186	1	1	1
KLHL23	1	1	9	0.11						3086	1	1	1
TSN	1	1	9	0.11		3	432	0.01		3051	1	1	1
TPM2	1	1	9	0.11		2	423	0		2996	1	1	1
QKI	1	1	9	0.11		5	431	0.01		2958	1	1	1
CAPG	1	1	9	0.11		5	431	0.01		2817	1	1	1
LHCGR	1	1	9	0.11	Yes					2787	1	1	1
CBX8	1	1	9	0.11		4	431	0.01		2732	1	1	1
ANGPTL1	1	1	9	0.11						2689	1	1	1
LRCH1	1	1	9	0.11		11	424	0.03		2606	1	1	1
MFN1	1	1	9	0.11		13	424	0.03		2528	1	1	1

RPUSD4	1	1	9	0.11		6	424	0.01		2483	1	1	1
GARS	1	1	9	0.11		9	423	0.02		2446	1	1	1
ZDHHC9	1	1	9	0.11		8	431	0.02		2380	1	1	1
OPN1MW	1	1	9	0.11		1	424	0		2322	1	1	1
TNFRSF21	1	1	9	0.11		7	432	0.02		2302	1	1	1
LMBRD2	1	1	9	0.11		12	423	0.03		2192	1	1	1
ZNF677	1	1	9	0.11		6	423	0.01		2141	1	1	1
SLC5A10	1	1	9	0.11						2127	1	1	1
RNF128	1	1	9	0.11		6	431	0.01		2068	1	1	1
HERPUD2	1	1	9	0.11		6	423	0.01		2051	1	1	1
DDX51	1	1	9	0.11		5	431	0.01	Yes	2046	1	1	1
DNAI2	1	1	9	0.11		19	423	0.04		2045	1	1	1
DCDC2	1	1	9	0.11		11	424	0.03		2039	1	1	1
WDR12	1	1	9	0.11		6	423	0.01		2032	1	1	1
C6orf211	1	1	9	0.11	Yes	7	424	0.02		2018	1	1	1
DCDC1	1	1	9	0.11		17	424	0.04		1956	1	1	1
PUS3	1	1	9	0.11						1905	1	1	1
MMP3	1	1	9	0.11		7	431	0.02		1904	1	1	1
KCNK10	1	1	9	0.11		20	424	0.05		1874	1	1	1
C8orf44	1	1	9	0.11						1826	1	1	1
TPPP2	1	1	9	0.11		3	423	0.01		1603	1	1	1
ZNF727	1	1	9	0.11						1512	1	1	1
CCDC50	1	1	9	0.11						1485	1	1	1
IRF3	1	1	9	0.11		4	423	0.01		1461	1	1	1
LRP2BP	1	1	9	0.11		3	423	0.01		1461	1	1	1
ZNF556	1	1	9	0.11		9	424	0.02		1383	1	1	1
GXYLT1	1	1	9	0.11		8	424	0.02		1347	1	1	1
TOB2	1	1	9	0.11		4	431	0.01		1345	1	1	1
ATP5J2	1	1	9	0.11						1280	1	1	1
CERS6	1	1	9	0.11						1212	1	1	1
IQCJ	1	1	9	0.11						1188	1	1	1
ATXN7L3	1	1	9	0.11		7	423	0.02		1164	1	1	1
TIMD4	1	1	9	0.11		22	425	0.05		1164	1	1	1
CXCR6	1	1	9	0.11	Yes					1032	1	1	1
SLC39A2	1	1	9	0.11		4	423	0.01		957	1	1	1
OR5B12	1	1	9	0.11		26	424	0.06		948	1	1	1
TMX1	1	1	9	0.11						920	1	1	1
RNASE1	1	1	9	0.11		1	423	0		828	1	1	1
APOBEC1	1	1	9	0.11		10	431	0.02		824	1	1	1
CALCA	1	1	9	0.11		2	423	0		803	1	1	1
R3HDML	1	1	9	0.11		14	425	0.03		777	1	1	1
GKN2	1	1	9	0.11		6	423	0.01		747	1	1	1

C18orf21	1	1	9	0.11				717	1	1	1
C22orf23	1	1	9	0.11				696	1	1	1
GYPA	1	1	9	0.11	4	423	0.01	647	1	1	1
C5orf63	1	1	9	0.11				606	1	1	1
PTP4A3	1	1	9	0.11	3	431	0.01	597	1	1	1
VAMP8	1	1	9	0.11				543	1	1	1
C17orf102	1	1	9	0.11	3	423	0.01	510	1	1	1
HBG1	1	1	9	0.11	2	423	0	453	1	1	1
SPANXN3	1	1	9	0.11	9	431	0.02	432	1	1	1
SUMO2	1	1	9	0.11				300	1	1	1
ATP6V0E1	1	1	9	0.11	2	423	0	283	1	1	1
SCGB1D1	1	1	9	0.11	4	424	0.01	282	1	1	1

Supplementary Table 6. Insertion and deletion

Sample ID	Chr	Start	Ends	Reads	Indel type	Indel base	Mut type	Gene Symbol	Gene ID	Transcript ID	mRNA to Chr	indel position	Function
A1	chr1	153907279	153907287	9	-9	CTGCTGCTG	Hete	DENND4B	9909	CCDS44228.1	-	exon Num=11	coding indel
A1	chr10	88994266	88994268	10	-3	CTC	Hete	FAM22A	728118	CCDS44452.1	+	exon Num=7	coding indel
A1	chr11	44297153	44297153	14	-1	C	Hete	ALX4	60529	CCDS31468.1	-	exon Num=3	frameshift
A1	chr11	107207442	107207442	11	-1	A	Hete	CWF19L2	143884	CCDS8336.2	-	adj_exon Num=4	spliceSite
A1	chr17	57141769	57141769	11	-1	A	Hete	TRIM37	4591	CCDS45746.1	-	adj_exon Num=16	spliceSite
A1	chr18	60645666	60645666	7	-1	G	Hete	PHLPP1	23239	CCDS45881.1	+	exon Num=17	frameshift
A1	chr19	19745427	19745427	7	-1	G	Hete	GMIP	51291	CCDS12408.1	-	exon Num=4	frameshift
A1	chr2	74709241	74709241	5	-1	C	Hete	CCDC142	84865	CCDS1945.1	-	exon Num=9	frameshift
A1	chr2	242794121	242794121	16	6	GGCTCC	Hete	PDCD1	5133	CCDS33428.1	-	exon Num=2	coding indel
A1	chr20	57899480	57899480	15	1	C	Hete	EDN3	1908	CCDS13478.1	+	exon Num=4	frameshift
A1	chr3	107097081	107097085	14	-5	AAATG	Hete	CCDC54	84692	CCDS2949.1	+	exon Num=1	frameshift
A1	chr5	64892934	64892934	19	1	T	Hete	TRIM23	373	CCDS3986.1	-	exon Num=5	frameshift
A1	chr9	116346117	116346117	30	1	A	Hete	RGS3	5998	CCDS43869.1	+	exon Num=20	frameshift
A1	chr9	116346119	116346119	30	2	GT	Hete	RGS3	5998	CCDS43869.1	+	exon Num=20	frameshift
A1	chrX	132888207	132888207	9	1	A	Homo	GPC3	2719	CCDS14638.1	-	adj_exon Num=6	spliceSite
A2	chr1	47610224	47610224	41	-1	G	Hete	CYP4A22	284541	CCDS30707.1	+	exon Num=8	frameshift
A2	chr1	205632218	205632253	7	-36	GCCGACAGCCCTTCTGCTGGCTCGGTGGGGCCCAGC	Hete	SLC45A3	85414	CCDS1458.1	-	exon Num=3	coding indel
A2	chr12	63964641	63964641	11	-1	A	Hete	DPY19L2	283417	CCDS31851.1	-	adj_exon Num=3	spliceSite
A2	chr6	70411439	70411439	17	1	A	Hete	LMBRD1	55788	CCDS4969.1	-	adj_exon Num=6	spliceSite
A2	chr7	37960263	37960275	7	-13	AGGCAGTGGCAGC	Hete	EPDR1	54749	CCDS5454.1	+	exon Num=1	frameshift
A2	chr7	106888947	106888947	23	-1	A	Hete	COG5	10466	CCDS5743.1	-	adj_exon Num=6	spliceSite
A2	chrX	51239296	51239309	8	-14	TCCTCGAGGCAGCC	Hete	NUDT11	55190	CCDS43952.1	-	exon Num=2	frameshift
A3	chr12	64020310	64020310	8	2	AA	Hete	DPY19L2	283417	CCDS31851.1	-	adj_exon Num=16	spliceSite
A3	chr13	21746601	21746601	15	-1	G	Hete	SKA3	221150	CCDS31946.1	-	exon Num=7	frameshift
A3	chr13	36241674	36241682	11	-9	TTTGGCTCA	Hete	NBEA	26960	CCDS45026.1	+	exon Num=56	coding indel
A3	chr17	37880981	37880981	69	12	GCATACGTGATG	Hete	ERBB2	2064	CCDS45667.1	+	exon Num=19	coding indel
A3	chr20	1896052	1896054	32	-3	CGA	Hete	SIRPA	140885	CCDS13022.1	+	exon Num=2	coding indel
A3	chr22	51183416	51183451	12	-36	ACCCCCGCCCCCACCCCCACCTCCAGCCTCACCTTT	Hete	ACR	49	CCDS14101.1	+	exon Num=5	coding indel
A3	chr3	9543998	9543998	12	2	AG	Hete	LHFPL4	375323	CCDS33691.1	-	adj_exon Num=1	spliceSite
A4	chr17	56056604	56056604	55	3	TGC	Hete	VEZF1	7716	CCDS32687.1	-	exon Num=2	coding indel
A4	chr17	65955758	65955758	27	9	CCTCCAGCC	Hete	BPTF	2186	CCDS11672.1	+	exon Num=26	coding indel
A4	chr19	41095087	41095112	10	-26	GAGGACAGTCCTGTCCAACAGGGAGG	Hete	SHKBP1	92799	CCDS12560.1	+	adj_exon Num=15	spliceSite
A4	chr2	128770731	128770731	8	1	A	Hete	SAP130	79595	CCDS2153.1	-	adj_exon Num=15	spliceSite
A4	chr2	141625835	141625835	8	-1	A	Hete	LRP1B	53353	CCDS2182.1	-	adj_exon Num=66	spliceSite
A4	chr4	54327211	54327211	10	1	A	Hete	LNX1	84708	CCDS47057.1	-	adj_exon Num=1	spliceSite
A4	chr7	99686615	99686615	12	6	GGCGGC	Hete	COPS6	10980	CCDS5682.1	+	exon Num=1	coding indel
A4	chr8	108315599	108315599	53	1	A	Hete	ANGPT1	284	CCDS6306.1	-	adj_exon Num=5	spliceSite
A5	chr1	203186950	203186950	32	24	AGACCATGGCCCCGCCCAGTCCCT	Hete	CHIT1	1118	CCDS1436.1	-	exon Num=2	coding indel
A5	chr1	223536703	223536705	16	-3	TGC	Hete	SUSD4	55061	CCDS41471.1	-	exon Num=8	coding indel
A5	chr11	1651614	1651643	55	-30	TACTGCTGCCAGTCCAGCTGCTGTAAGCCC	Hete	KRTAP5-5	439915	CCDS41592.1	+	exon Num=1	coding indel
A5	chr13	73409508	73409508	12	1	A	Hete	PIBF1	10464	CCDS31991.1	+	adj_exon Num=8	spliceSite
A5	chr8	103573011	103573037	25	-27	TGCAACCCCTGCAGCCCCTGCAACCCG	Hete	ODF1	4956	CCDS6293.1	+	exon Num=2	coding indel
A6	chr1	2430086	2430086	13	9	GTGGGGGCC	Homo	PLCH2	9651	NM_014638	+	exon Num=17	coding indel
A6	chr10	105233174	105233174	8	18	GGGGGCTCAGGCACTGCG	Hete	CALHM3	119395	CCDS44476.1	-	exon Num=1	coding indel
A6	chr11	17449417	17449417	35	-1	G	Hete	ABCC8	6833	CCDS31437.1	-	exon Num=25	frameshift
A6	chr11	56020594	56020594	27	-1	T	Hete	OR5T3	390154	CCDS31524.1	+	exon Num=1	frameshift
A6	chr12	53189414	53189431	69	-18	CCAAAGCCACCAGCCCCT	Hete	KRT3	3850	CCDS44895.1	-	exon Num=9	coding indel
A6	chr14	92258905	92258905	26	1	A	Hete	TC2N	123036	CCDS9897.1	-	adj_exon Num=4	spliceSite
A6	chr15	74425963	74425964	7	-2	GG	Hete	ISLR2	57611	CCDS10259.1	+	exon Num=1	frameshift
A6	chr17	59482922	59482922	5	1	C	Hete	TBX2	6909	CCDS11627.2	+	exon Num=6	frameshift
A6	chr2	46093	46094	8	-2	TC	Hete	FAM110C	642273	CCDS42645.1	-	exon Num=2	frameshift
A6	chr20	30556350	30556350	5	1	C	Hete	XKR7	343702	CCDS33459.1	+	exon Num=1	frameshift
A6	chr5	127607730	127607730	32	-1	G	Hete	FBN2	2201	CCDS34222.1	-	exon Num=4	frameshift
A6	chr6	147648415	147648415	36	5	AAAAG	Hete	STXBP5	134957	CCDS5211.1	+	adj_exon Num=18	spliceSite

A6	chr7	29923627	29923627	6	13	CCCCCCCCCCCCC	Hete	WIPF3	644150	NM_001080529	+	exon Num=4	frameshift
A6	chr7	127235493	127235493	25	-1	G	Hete	FSCN3	29999	CCDS34746.1	+	exon Num=2	frameshift
A6	chr7	138310792	138310792	6	-1	G	Hete	SVOPL	136306	CCDS5848.1	-	exon Num=4	frameshift
A6	chr9	111617059	111617059	5	1	C	Hete	ACTL7B	10880	CCDS6771.1	-	exon Num=1	frameshift
A6	chrX	9707626	9707626	11	-1	G	Homo	GPR143	4935	CCDS14134.1	-	exon Num=2	frameshift
A7	chr11	76804834	76804834	17	-1	C	Hete	CAPN5	726	CCDS8248.1	+	exon Num=2	frameshift
A7	chr15	63447933	63447933	11	-1	A	Hete	RPS27L	51065	CCDS42048.1	-	adj_exon Num=2	spliceSite
A7	chr7	80302698	80302709	10	-12	TATTGTGCCTAT	Hete	CD36	948	CCDS34673.1	+	exon Num=11	coding indel
A8	chr1	2458000	2458008	9	-9	GCTGCCGCT	Hete	PANK4	55229	CCDS42.1	-	exon Num=19	coding indel
A8	chr1	26608890	26608891	8	-2	CC	Hete	UBXN11	91544	CCDS41288.1	-	exon Num=1	frameshift
A8	chr1	26608893	26608896	8	-4	GGAC	Hete	UBXN11	91544	CCDS41288.1	-	exon Num=1	frameshift
A8	chr1	248524967	248524969	6	-3	ATG	Homo	OR2T4	127074	CCDS31113.1	+	exon Num=1	coding indel
A8	chr19	23545530	23545530	6	1	A	Hete	ZNF91	7644	CCDS42541.1	-	adj_exon Num=1	spliceSite
A8	chr2	26785482	26785482	10	1	G	Hete	C2orf70	339778	CCDS42661.1	+	exon Num=1	frameshift
A8	chr3	37778478	37778478	32	1	G	Hete	ITGA9	3680	CCDS2669.1	+	adj_exon Num=20	spliceSite
A8	chr7	27135309	27135317	11	-9	GGTGGCGAT	Hete	HOXA1	3198	CCDS5401.1	-	exon Num=2	coding indel
A8	chr9	79244209	79244209	12	-1	A	Hete	PRUNE2	158471	CCDS47982.1	-	adj_exon Num=4	spliceSite
A8	chr9	90534191	90534201	11	-11	CATCTTGTCTC	Homo	FAM75C1	441452	NM_001145124	+	exon Num=2	frameshift
A9	chr1	103356059	103356059	26	-1	C	Hete	COL11A1	1301	CCDS778.1	-	exon Num=10	frameshift
A9	chr1	120612003	120612004	5	-2	GG	Hete	NOTCH2	4853	CCDS908.1	-	exon Num=34	frameshift
A9	chr10	35314130	35314130	19	1	A	Hete	CUL2	8453	CCDS7179.1	-	adj_exon Num=5	spliceSite
A9	chr10	75000737	75000742	12	-6	GCGGAG	Hete	FAM149B1	317662	CCDS44435.1	+	exon Num=14	coding indel
A9	chr10	75000748	75000756	12	-9	CCAGTCTCT	Hete	FAM149B1	317662	CCDS44435.1	+	exon Num=14	coding indel
A9	chr12	21623284	21623284	13	3	AAA	Hete	RECQL	5965	CCDS31756.1	-	adj_exon Num=1	spliceSite
A9	chr16	3727520	3727520	18	-1	C	Hete	TRAP1	10131	CCDS10508.1	-	exon Num=13	frameshift
A9	chr16	67913784	67913804	5	-21	GTAGCAGCAGCAGCAGCAGCA	Hete	EDC4	23644	CCDS10849.1	+	exon Num=16	coding indel
A9	chr17	4803338	4803344	6	-7	ACCGAAG	Hete	C17orf107	100130311	CCDS45591.1	+	exon Num=2	frameshift
A9	chr17	39262018	39262018	27	-1	C	Hete	KRTAP4-9	100132386	NM_001146041	+	exon Num=1	frameshift
A9	chr17	79093270	79093270	8	6	GGGCGT	Hete	AATK	9625	CCDS45807.1	-	exon Num=2	coding indel
A9	chr19	57327346	57327346	22	1	T	Hete	PEG3	5178	CCDS12948.1	-	exon Num=1	frameshift
A9	chr2	175292581	175292593	10	-13	TCAAATTTATCAG	Hete	SCRN3	79634	CCDS2258.1	+	exon Num=7	frameshift
A9	chr20	1592043	1592044	8	-2	CA	Homo	SIRPB1	10326	CCDS46571.1	-	exon Num=4	frameshift
A9	chr20	1592048	1592048	8	-1	G	Homo	SIRPB1	10326	CCDS46571.1	-	exon Num=4	frameshift
A9	chr21	22707963	22707963	9	-1	G	Hete	NCAM2	4685	CCDS42910.1	+	exon Num=7	frameshift
A9	chr4	88537268	88537294	52	-27	GACAGCAGTGACAGCAGCGACAGCAGT	Hete	DSPP	1834	CCDS43248.1	+	exon Num=4	coding indel
A9	chr6	90577712	90577728	7	-17	CTTTGCCCAGACATGGA	Hete	CASP8AP2	9994	NM_012115	+	exon Num=7	frameshift
A9	chr6	139204007	139204015	9	-9	AGGTAAATG	Hete	ECT2L	345930	CCDS43508.1	+	exon Num=14	coding indel
A9	chrX	152719970	152719973	5	-4	GGGA	Homo	HAUS7	55559	CCDS35438.1	-	adj_exon Num=2	spliceSite

Supplementary Table 7. Copy number variations

Sample ID	chromosome	start	end	Genes affected	Copy num variation	Mutations	Indels
A1	chr1	151210657	151210741	PIP5K1A	Gain	No	No
A1	chr10	69597691	69597937	DNAJC12	Gain	No	No
A1	chr10	70191962	70192072	DNA2	Gain	Yes	No
A1	chr10	70225423	70225569	DNA2	Gain	Yes	No
A1	chr10	70227879	70228063	DNA2	Gain	Yes	No
A1	chr10	70246900	70246969	SLC25A16	Gain	No	No
A1	chr10	70276507	70276600	SLC25A16	Gain	No	No
A1	chr10	70497601	70497646	CCAR1	Gain	No	No
A1	chr10	70506917	70507032	CCAR1	Gain	No	No
A1	chr10	70532744	70532856	CCAR1	Gain	No	No
A1	chr10	70545892	70545975	CCAR1	Gain	No	No
A1	chr10	70546302	70546449	CCAR1	Gain	No	No
A1	chr10	70951410	70951522	SUPV3L1	Gain	No	No
A1	chr10	71018531	71018715	HKDC1	Gain	No	No
A1	chr10	73832260	73832306	SPOCK2	Gain	No	No
A1	chr10	73972944	73973089	ASCC1	Gain	No	No
A1	chr10	74100742	74100928	DNAJB12	Gain	No	No
A1	chr10	74103123	74103269	DNAJB12	Gain	No	No
A1	chr10	74776603	74776653	P4HA1	Gain	No	No
A1	chr10	74916325	74916413	ECD	Gain	No	No
A1	chr10	75139636	75139712	ANXA7	Gain	No	No
A1	chr10	75143334	75143448	ANXA7	Gain	No	No
A1	chr10	75156276	75156341	ANXA7	Gain	No	No
A1	chr10	75264607	75264748	USP54	Gain	No	No
A1	chr10	75302775	75302889	USP54	Gain	No	No
A1	chr10	75510865	75511001	SEC24C	Gain	No	No
A1	chr10	75523613	75523741	SEC24C	Gain	No	No
A1	chr10	75549474	75549582	ZSWIM8	Gain	No	No
A1	chr10	75553622	75553802	ZSWIM8	Gain	No	No
A1	chr10	75557987	75558211	ZSWIM8,ZSWIM8-AS1	Gain	No	No
A1	chr10	75560260	75560317	ZSWIM8,ZSWIM8-AS1	Gain	No	No
A1	chr10	76735156	76735212	KAT6B	Gain	No	No

A1	chr10	79576764	79576854	DLG5	Gain	No	No
A1	chr10	79613111	79613295	DLG5	Gain	No	No
A1	chr10	79613984	79614128	DLG5	Gain	No	No
A1	chr10	79628886	79628955	DLG5	Gain	No	No
A1	chr10	79743962	79744056	POLR3A	Gain	No	No
A1	chr10	79745830	79745917	POLR3A	Gain	No	No
A1	chr10	79750811	79750925	POLR3A	Gain	No	No
A1	chr10	79759738	79759876	POLR3A	Gain	No	No
A1	chr10	79767459	79767624	POLR3A	Gain	No	No
A1	chr10	79769294	79769433	POLR3A	Gain	No	No
A1	chr10	79770228	79770298	POLR3A	Gain	No	No
A1	chr10	79773407	79773548	POLR3A	Gain	No	No
A1	chr10	79777332	79777474	POLR3A	Gain	No	No
A1	chr10	79778919	79779023	POLR3A	Gain	No	No
A1	chr10	79781617	79781780	POLR3A	Gain	No	No
A1	chr10	79781902	79782142	POLR3A	Gain	No	No
A1	chr10	79796951	79797062	RPS24	Gain	No	No
A1	chr10	81036937	81037082	ZMIZ1	Gain	No	No
A1	chr10	81063771	81063932	ZMIZ1	Gain	No	No
A1	chr10	81067161	81067328	ZMIZ1	Gain	No	No
A1	chr10	81192313	81192514	ZCCHC24	Gain	No	No
A1	chr10	81371337	81371397	SFTPA1	Gain	No	No
A1	chr10	81841596	81841700	TMEM254	Gain	No	No
A1	chr10	81904655	81904784	PLAC9	Gain	No	No
A1	chr11	47447417	47447501	PSMC3	Gain	No	No
A1	chr11	64136917	64137091	RPS6KA4	Gain	No	No
A1	chr11	118883941	118883972	CCDC84	Gain	No	No
A1	chr14	21789417	21789561	RPGRIP1	Gain	No	No
A1	chr14	21834597	21834688	SUPT16H	Gain	No	No
A1	chr14	31424825	31424899	STRN3	Gain	No	No
A1	chr14	31535331	31535540	AP4S1	Gain	No	No
A1	chr14	31539048	31539135	AP4S1	Gain	No	No
A1	chr14	31582309	31582395	HECTD1	Gain	No	No
A1	chr14	31614012	31614147	HECTD1	Gain	No	No

A1	chr14	31647225	31647460	HECTD1	Gain	No	No
A1	chr14	31765114	31765269	HEATR5A	Gain	No	No
A1	chr14	31775937	31776085	HEATR5A	Gain	No	No
A1	chr14	31787429	31787498	HEATR5A	Gain	No	No
A1	chr14	34993902	34994013	EAPP	Gain	No	No
A1	chr14	34998563	34998681	EAPP	Gain	No	No
A1	chr14	35002649	35002745	EAPP	Gain	No	No
A1	chr14	35005299	35005481	EAPP	Gain	No	No
A1	chr14	35036891	35036977	SNX6	Gain	No	No
A1	chr14	35037059	35037219	SNX6	Gain	No	No
A1	chr14	35044961	35045048	SNX6	Gain	No	No
A1	chr14	35050766	35050806	SNX6	Gain	No	No
A1	chr14	35055435	35055511	SNX6	Gain	No	No
A1	chr14	35062250	35062356	SNX6	Gain	No	No
A1	chr14	35066755	35066851	SNX6	Gain	No	No
A1	chr14	35072553	35072677	SNX6	Gain	No	No
A1	chr14	35074801	35074923	SNX6	Gain	No	No
A1	chr14	35078843	35078948	SNX6	Gain	No	No
A1	chr14	35269429	35269596	BAZ1A	Gain	No	No
A1	chr14	35476488	35476593	SRP54	Gain	No	No
A1	chr14	35487897	35487971	SRP54	Gain	No	No
A1	chr14	35560275	35560413	PPP2R3C	Gain	No	No
A1	chr14	35564253	35564390	PPP2R3C	Gain	No	No
A1	chr14	35568457	35568590	PPP2R3C	Gain	No	No
A1	chr14	35585815	35585943	PPP2R3C	Gain	No	No
A1	chr14	35871599	35871869	NFKBIA	Gain	No	No
A1	chr14	36240147	36240205	RALGAPA1	Gain	No	No
A1	chr14	39591633	39591747	GEMIN2	Gain	No	No
A1	chr14	39602864	39602923	GEMIN2	Gain	No	No
A1	chr16	18591531	18591657	ABCC6P1	Gain	No	No
A1	chr16	28777402	28777502	NPIPB9	Gain	No	No
A1	chr16	29001094	29001160	LAT	Gain	No	No
A1	chr16	30128990	30129105	MAPK3	Gain	No	No
A1	chr16	30516564	30516621	ITGAL	Gain	No	No

A1	chr17	40726116	40726228	PSMC3IP	Gain	No	No
A1	chr19	10655458	10655542	ATG4D	Gain	No	No
A1	chr19	10689567	10689639	AP1M2	Gain	No	No
A1	chr19	11606764	11606980	ZNF653	Gain	No	No
A1	chr19	12783660	12783728	WDR83	Gain	No	No
A1	chr19	12883807	12883858	HOOK2	Gain	No	No
A1	chr19	17628518	17628659	PGLS	Gain	No	No
A1	chr19	49416730	49416821	NUCB1	Gain	No	No
A1	chr19	50905868	50905998	POLD1	Gain	No	No
A1	chr20	35386489	35386564	DSN1	Gain	No	No
A1	chr20	35569441	35569514	SAMHD1	Gain	No	No
A1	chr20	47707473	47707559	CSE1L	Gain	No	No
A1	chr21	37518373	37518860	CBR3,CBR3-AS1	Gain	No	No
A1	chr21	37518553	37518653	CBR3,CBR3-AS1	Gain	No	No
A1	chr21	37536838	37536891	DOPEY2	Gain	No	No
A1	chr21	37610898	37610999	DOPEY2	Gain	No	No
A1	chr21	37612062	37612253	DOPEY2	Gain	No	No
A1	chr21	37632974	37633030	DOPEY2	Gain	No	No
A1	chr21	37635843	37635948	DOPEY2	Gain	No	No
A1	chr21	37636045	37636112	DOPEY2	Gain	No	No
A1	chr21	37642310	37642455	DOPEY2	Gain	No	No
A1	chr21	37649318	37649398	DOPEY2	Gain	No	No
A1	chr21	37650272	37650382	DOPEY2	Gain	No	No
A1	chr21	37650506	37650653	DOPEY2	Gain	No	No
A1	chr21	37652582	37652644	DOPEY2	Gain	No	No
A1	chr21	37660311	37660448	DOPEY2	Gain	No	No
A1	chr21	37661342	37661504	DOPEY2	Gain	No	No
A1	chr21	37705943	37706016	MORC3	Gain	No	No
A1	chr21	37710029	37710244	MORC3	Gain	No	No
A1	chr21	37713696	37713844	MORC3	Gain	No	No
A1	chr21	37716876	37717005	MORC3	Gain	No	No
A1	chr21	37728917	37729022	MORC3	Gain	No	No
A1	chr21	37732252	37732375	MORC3	Gain	No	No
A1	chr21	37732481	37732556	MORC3	Gain	No	No

A1	chr21	37734480	37734526	MORC3	Gain	No	No
A1	chr21	37744671	37744829	MORC3	Gain	No	No
A1	chr21	37766844	37766948	CHAF1B	Gain	No	No
A1	chr21	37781058	37781128	CHAF1B	Gain	No	No
A1	chr21	37783760	37783902	CHAF1B	Gain	No	No
A1	chr21	38539856	38539922	TTC3	Gain	No	No
A1	chr21	38560795	38560896	TTC3	Gain	No	No
A1	chr21	38600523	38600674	DSCR3	Gain	No	No
A1	chr21	40717071	40717200	HMGN1	Gain	No	No
A1	chr21	40720217	40720265	HMGN1	Gain	No	No
A1	chr21	40720347	40720377	HMGN1	Gain	No	No
A1	chr21	43291602	43291730	PRDM15	Gain	No	No
A1	chr21	44296811	44296877	WDR4	Gain	No	No
A1	chr21	44433177	44433348	PKNOX1	Gain	No	No
A1	chr21	44486352	44486487	CBS	Gain	No	No
A1	chr21	44840113	44840337	SIK1	Gain	No	No
A1	chr21	45168875	45168961	PDXK	Gain	No	No
A1	chr21	45217511	45217576	RRP1	Gain	No	No
A1	chr21	45388998	45389160	AGPAT3	Gain	No	No
A1	chr21	45397926	45398002	AGPAT3	Gain	No	No
A1	chr21	45451971	45452053	TRAPPC10	Gain	No	No
A1	chr21	45475578	45475774	TRAPPC10	Gain	No	No
A1	chr21	45533633	45533728	PWP2	Gain	No	No
A1	chr21	45540228	45540377	PWP2	Gain	No	No
A1	chr21	45651162	45651327	ICOSLG	Gain	No	No
A1	chr21	45674499	45674588	DNMT3L	Gain	No	No
A1	chr21	45675949	45676037	DNMT3L	Gain	No	No
A1	chr21	45679319	45679432	DNMT3L	Gain	No	No
A1	chr21	45680677	45680722	DNMT3L	Gain	No	No
A1	chr21	45681036	45681149	DNMT3L	Gain	No	No
A1	chr21	45710977	45711093	AIRE	Gain	No	No
A1	chr21	45712875	45713058	AIRE	Gain	No	No
A1	chr21	45733795	45733904	PFKL	Gain	No	No
A1	chr21	45739233	45739298	PFKL	Gain	No	No

A1	chr21	45773483	45773748	TRPM2	Gain	No	No
A1	chr21	45774530	45774619	TRPM2	Gain	No	No
A1	chr21	45783996	45784165	TRPM2	Gain	No	No
A1	chr21	45815296	45815434	TRPM2	Gain	No	No
A1	chr21	45819178	45819324	TRPM2	Gain	No	No
A1	chr21	45825024	45825143	TRPM2	Gain	No	No
A1	chr21	46898240	46898267	COL18A1	Gain	No	No
A1	chr21	47359943	47360113	PCBP3	Gain	No	No
A1	chr21	47418034	47418085	COL6A1	Gain	No	No
A1	chr21	47423033	47423063	COL6A1	Gain	No	No
A1	chr21	47614404	47614575	LSS	Gain	No	No
A1	chr21	47680706	47680820	MCM3AP	Gain	Yes	No
A1	chr21	47681193	47681325	MCM3AP	Gain	Yes	No
A1	chr21	47684030	47684131	MCM3AP	Gain	Yes	No
A1	chr21	47686895	47687056	MCM3AP	Gain	Yes	No
A1	chr21	47690314	47690477	MCM3AP	Gain	Yes	No
A1	chr21	47692474	47692743	MCM3AP	Gain	Yes	No
A1	chr21	47693301	47693499	MCM3AP	Gain	Yes	No
A1	chr21	47695099	47695239	MCM3AP	Gain	Yes	No
A1	chr21	47697440	47697631	MCM3AP	Gain	Yes	No
A1	chr21	47700410	47700489	MCM3AP	Gain	Yes	No
A1	chr21	47711247	47711376	YBEY	Gain	No	No
A1	chr21	47746290	47746503	PCNT	Gain	No	No
A1	chr21	47754310	47754682	PCNT	Gain	No	No
A1	chr21	47766041	47766122	PCNT	Gain	No	No
A1	chr21	47771342	47771454	PCNT	Gain	No	No
A1	chr21	47773017	47773240	PCNT	Gain	No	No
A1	chr21	47775366	47775541	PCNT	Gain	No	No
A1	chr21	47817927	47818044	PCNT	Gain	No	No
A1	chr21	47838117	47838220	PCNT	Gain	No	No
A1	chr21	47841883	47842038	PCNT	Gain	No	No
A1	chr21	47847535	47847709	PCNT	Gain	No	No
A1	chr21	47924273	47924402	DIP2A	Gain	No	No
A1	chr21	47929169	47929289	DIP2A	Gain	No	No

A1	chr21	47954497	47954600	DIP2A	Gain	No	No
A1	chr21	47957358	47957452	DIP2A	Gain	No	No
A1	chr21	47959789	47959904	DIP2A	Gain	No	No
A1	chr22	42341916	42342009	CENPM	Gain	No	No
A1	chr5	134015291	134015418	SEC24A	Gain	No	No
A1	chr7	5434070	5434226	TNRC18	Gain	No	No
A1	chr7	5530861	5531080	FBXL18	Gain	No	No
A1	chr7	5965262	5965603	CCZ1	Gain	Yes	No
A1	chr7	5984694	5984809	RSPH10B	Gain	No	No
A1	chr7	6085701	6085782	EIF2AK1	Gain	Yes	No
A1	chr7	6426842	6426914	RAC1	Gain	No	No
A1	chr7	6441498	6441658	RAC1	Gain	No	No
A1	chr7	6485583	6485735	DAGLB	Gain	No	No
A1	chr7	6545450	6545538	GRID2IP	Gain	Yes	No
A1	chr7	6634065	6634231	C7orf26	Gain	No	No
A1	chr7	6785718	6785819	PMS2CL	Gain	No	No
A1	chr7	6826508	6826652	RSPH10B2	Gain	No	No
A1	chr7	23545756	23545885	TRA2A	Gain	No	No
A1	chr8	95777938	95778037	DPY19L4	Gain	No	No
A1	chr8	95780622	95780781	DPY19L4	Gain	No	No
A1	chr8	95782679	95782799	DPY19L4	Gain	No	No
A1	chr8	95793311	95793394	DPY19L4	Gain	No	No
A1	chr8	98673299	98673401	MTDH	Gain	No	No
A1	chr8	98718853	98718978	MTDH	Gain	No	No
A1	chr8	98725889	98725997	MTDH	Gain	No	No
A1	chr8	98817580	98817692	LAPTM4B	Gain	No	No
A1	chr8	99718694	99718862	STK3	Gain	Yes	No
A1	chr8	99786966	99787047	STK3	Gain	Yes	No
A1	chr8	100025987	100026163	VPS13B	Gain	No	No
A1	chr8	100050650	100050794	VPS13B	Gain	No	No
A1	chr8	100108539	100108660	VPS13B	Gain	No	No
A1	chr8	100975112	100975206	RGS22	Gain	No	No
A1	chr8	100990144	100990303	RGS22	Gain	No	No
A1	chr8	101054029	101054144	RGS22	Gain	No	No

A1	chr8	101196183	101196290	SPAG1	Gain	No	No
A1	chr8	101203617	101203724	SPAG1	Gain	No	No
A1	chr8	101716524	101716618	PABPC1	Gain	No	No
A1	chr8	101717816	101717901	PABPC1	Gain	No	No
A1	chr8	101718878	101719033	PABPC1	Gain	No	No
A1	chr8	101719114	101719225	PABPC1	Gain	No	No
A1	chr8	101724589	101724685	PABPC1	Gain	No	No
A1	chr8	101724879	101725017	PABPC1	Gain	No	No
A1	chr8	101725314	101725409	PABPC1	Gain	No	No
A1	chr8	101727689	101727829	PABPC1	Gain	No	No
A1	chr8	101730000	101730116	PABPC1	Gain	No	No
A1	chr8	101730314	101730508	PABPC1	Gain	No	No
A1	chr8	101936182	101936278	YWHAZ	Gain	No	No
A1	chr8	101936362	101936526	YWHAZ	Gain	No	No
A1	chr8	103358463	103358623	UBR5	Gain	No	No
A1	chr8	103841499	103841714	AZIN1	Gain	No	No
A1	chr8	104225146	104225313	BAALC	Gain	No	No
A1	chr8	125516511	125516588	TATDN1	Gain	No	No
A1	chr8	125520714	125520755	TATDN1	Gain	No	No
A1	chr8	125520755	125520817	TATDN1	Gain	No	No
A1	chr8	125520843	125520929	TATDN1	Gain	No	No
A1	chr8	125528127	125528271	TATDN1	Gain	No	No
A1	chr8	125531058	125531122	TATDN1	Gain	No	No
A1	chr8	125534391	125534441	TATDN1	Gain	No	No
A1	chr8	125562001	125562227	NDUFB9	Gain	No	No
A1	chr8	125575022	125575233	NDUFB9,MTSS1	Gain	No	No
A1	chr8	125579311	125579419	NDUFB9,MTSS1	Gain	No	No
A1	chr8	125597327	125597402	MTSS1	Gain	No	No
A1	chr8	125601860	125601952	MTSS1	Gain	No	No
A1	chr8	125603391	125603476	MTSS1	Gain	No	No
A1	chr8	125711766	125711840	MTSS1	Gain	No	No
A1	chr8	126021413	126021585	SQLE	Gain	No	No
A1	chr8	126049478	126049575	KIAA0196	Gain	No	No
A1	chr8	126056066	126056146	KIAA0196	Gain	No	No

A1	chr8	126059448	126059573	KIAA0196	Gain	No	No
A1	chr8	126062805	126062907	KIAA0196	Gain	No	No
A1	chr8	126067832	126067913	KIAA0196	Gain	No	No
A1	chr8	126087239	126087353	KIAA0196	Gain	No	No
A1	chr8	126369460	126369561	NSMCE2	Gain	No	No
A1	chr8	130863080	130863160	FAM49B	Gain	No	No
A1	chr8	131092147	131092217	ASAP1	Gain	No	No
A1	chr8	131172109	131172210	ASAP1	Gain	No	No
A1	chr8	145151239	145151397	CYC1	Gain	No	No
A1	chr9	130223451	130223536	LRSAM1	Gain	No	No
A1	chrX	48339538	48339591	FTSJ1	Gain	No	No
A1	chrX	153176153	153176288	ARHGAP4	Gain	No	No
A1	chrX	153631862	153631963	DNASE1L1	Gain	No	No
A1	chr10	17495557	17495656	ST8SIA6	Loss	No	No
A1	chr10	22617528	22617627	BMI1,COMMD3-BMI1	Loss	No	No
A1	chr10	32635861	32635976	EPC1	Loss	No	No
A1	chr10	42947056	42947131	CCNYL2	Loss	No	No
A1	chr10	129245657	129245809	DOCK1	Loss	No	No
A1	chr10	129859942	129859979	PTPRE	Loss	No	No
A1	chr10	131638508	131638598	EBF3	Loss	No	No
A1	chr10	131666045	131666176	EBF3	Loss	No	No
A1	chr10	135350566	135350754	CYP2E1	Loss	No	No
A1	chr11	117714824	117714921	FXYD6,FXYD6-FXYD2	Loss	No	No
A1	chr12	2113447	2113677	CACNA1C,DCP1B	Loss	No	No
A1	chr12	31135998	31136085	TSPAN11	Loss	No	No
A1	chr12	41330582	41330707	CNTN1	Loss	No	No
A1	chr12	80770902	80770982	OTOGL	Loss	No	No
A1	chr14	104640042	104640205	KIF26A	Loss	No	No
A1	chr15	35838319	35838404	DPH6	Loss	No	No
A1	chr15	74369743	74369794	GOLGA6A	Loss	No	No
A1	chr17	67081168	67081323	ABCA6	Loss	No	No
A1	chr2	39931220	39931334	TMEM178A	Loss	No	No
A1	chr2	75105814	75106048	HK2	Loss	No	No
A1	chr2	139537760	139537811	NXPH2	Loss	No	No

A1	chr21	17181127	17181204	USP25	Loss	No	No
A1	chr21	28315698	28315866	ADAMTS5	Loss	No	No
A1	chr3	38913683	38913785	SCN11A	Loss	Yes	No
A1	chr4	3533709	3533876	LRPAP1	Loss	No	No
A1	chr4	6860151	6860233	KIAA0232	Loss	No	No
A1	chr4	24914438	24914600	CCDC149	Loss	No	No
A1	chr4	90759402	90759447	SNCA,LOC644248	Loss	No	No
A1	chr4	119216932	119217011	PRSS12	Loss	No	No
A1	chr4	151174625	151174708	DCLK2	Loss	Yes	No
A1	chr4	187159409	187159519	KLKB1	Loss	No	No
A1	chr5	56247920	56247957	MIER3	Loss	No	No
A1	chr6	70964665	70964719	COL9A1	Loss	No	No
A1	chr6	70983749	70983785	COL9A1	Loss	No	No
A1	chr6	70984421	70984475	COL9A1	Loss	No	No
A1	chr6	73001636	73001749	RIMS1	Loss	No	No
A1	chr6	75798821	75798890	COL12A1	Loss	Yes	No
A1	chr6	75833045	75833120	COL12A1	Loss	Yes	No
A1	chr6	79713446	79713575	PHIP,IRAK1BP1	Loss	No	No
A1	chr6	80720530	80720674	TTK	Loss	No	No
A1	chr6	84311303	84311318	SNAP91	Loss	No	No
A1	chr6	96973172	96973270	UFL1	Loss	No	No
A1	chr6	96990769	96990892	UFL1	Loss	No	No
A1	chr6	117645494	117645578	ROS1	Loss	No	No
A1	chr6	123637601	123637640	TRDN	Loss	No	No
A1	chr6	128563665	128563747	PTPRK	Loss	No	No
A1	chr6	129794358	129794497	LAMA2	Loss	Yes	No
A1	chr6	131925317	131925460	MED23	Loss	No	No
A1	chr6	135306478	135306547	HBS1L	Loss	No	No
A1	chr6	135776871	135777064	AHI1	Loss	No	No
A1	chr7	142481126	142481380	PRSS3P2	Loss	No	No
A1	chr7	142482211	142482399	PRSS3P2	Loss	No	No
A1	chr8	2007273	2007366	MYOM2	Loss	Yes	No
A1	chr8	2813110	2813290	CSMD1	Loss	Yes	No
A1	chr8	2830638	2830821	CSMD1	Loss	Yes	No

A1	chr8	3015418	3015488	CSMD1	Loss	Yes	No
A1	chr8	7806650	7806777	ZNF705B	Loss	No	No
A1	chr8	10697247	10697409	PINX1	Loss	No	No
A1	chr8	14022088	14022211	SGCZ	Loss	No	No
A1	chr8	16962921	16963093	MICU3	Loss	No	No
A1	chr8	17843467	17843596	PCM1	Loss	No	No
A1	chr8	24200613	24200694	ADAM28	Loss	No	No
A1	chr8	24321394	24321473	ADAM7	Loss	No	No
A1	chr8	39550118	39550199	ADAM18	Loss	Yes	No
A1	chr8	39646187	39646259	ADAM2	Loss	No	No
A1	chr9	2622099	2622373	VLDLR,FLJ35024	Loss	No	No
A1	chr9	111843118	111843223	TMEM245	Loss	No	No
A1	chrY	24457006	24457119	RBMY2FP	Loss	No	No
A2	chr17	1340083	1413093	CRK	Gain	No	No
A2	chr17	1456297	1586995	PITPNA	Gain	No	No
A2	chr17	2240807	2304258	SGSM2	Gain	No	No
A2	chr17	3831956	3854683	ATP2A3	Gain	No	No
A2	chr17	4842354	4800108	SLC25A11	Gain	No	No
A2	chr17	7133126	7193390	DVL2	Gain	No	No
A2	chr17	7293952	7317812	PLSCR3,C17orf61-PLSCR3	Gain	No	No
A2	chr17	7482805	7579937	CD68	Gain	No	No
A2	chr17	8027360	8054163	HES7	Gain	No	No
A2	chr17	18065898	18152722	MYO15A	Gain	Yes	No
A2	chr17	59557450	60005377	TBX4	Gain	No	No
A2	chr17	61710040	61623306	MAP3K3	Gain	No	No
A2	chr17	61864393	63632092	DDX42	Gain	Yes	No
A2	chr17	62079092	62050278	C17orf72	Gain	Yes	No
A2	chr17	72947999	72921787	HID1	Gain	No	No
A2	chr17	73234732	73242877	GGA3	Gain	No	No
A2	chr17	73257628	73231854	GGA3	Gain	No	No
A2	chr17	73497548	73494709	CASKIN2	Gain	No	No
A2	chr17	73698559	73775044	SAP30BP	Gain	No	No
A2	chr17	73824867	73851501	UNC13D	Gain	No	No
A2	chr17	73906617	74151815	FBF1	Gain	No	No

A2	chr17	74002926	74020201	EVPL	Gain	Yes	No
A2	chr17	74077073	74099868	ZACN	Gain	No	No
A2	chr17	74150130	74383941	RNF157,RNF157-AS1	Gain	No	No
A2	chr17	74169782	73571290	RNF157	Gain	No	No
A2	chr17	74536586	74477627	PRCD	Gain	No	No
A2	chr17	76419777	76495194	PGS1	Gain	No	No
A2	chr17	78081598	78063683	GAA	Gain	No	No
A2	chr17	78183078	78225200	SGSH,CARD14	Gain	No	No
A2	chr17	78237372	78359421	RNF213	Gain	No	No
A2	chr17	78681640	78936377	RPTOR	Gain	No	No
A2	chr17	78968380	79139872	CHMP6	Gain	No	No
A2	chr17	79105701	79203276	AATK	Gain	Yes	Yes
A2	chr17	79414061	79165135	BAHCC1	Gain	No	No
A2	chr17	79766717	79818544	GCGR	Gain	Yes	No
A2	chr17	79857117	79995573	ANAPC11	Gain	No	No
A2	chr17	80059345	80206890	CCDC57	Gain	No	No
A2	chr17	80194543	80400516	SLC16A3	Gain	No	No
A2	chr17	80574399	80656598	WDR45B	Gain	No	No
A2	chr17	80709939	78519591	TBCD	Gain	Yes	No
A2	chr18	116841	210031	ROCK1P1	Gain	No	No
A2	chr18	321661	649964	COLEC12	Gain	No	No
A2	chr18	659640	706578	TYMS	Gain	No	No
A2	chr18	2595414	3253426	NDC80	Gain	Yes	No
A2	chr18	2847806	2762234	EMILIN2	Gain	Yes	No
A2	chr18	9775274	9593872	RAB31	Gain	No	No
A2	chr18	12801968	12785843	PTPN2	Gain	No	No
A2	chr18	13387339	10468649	LDLRAD4	Gain	No	No
A2	chr18	74589964	74721882	ZNF236	Gain	No	No
A2	chr19	8587517	8617048	MYO1F	Gain	No	No
A2	chr20	40126807	42574678	CHD6	Gain	Yes	No
A2	chr20	42194736	42345136	SGK2	Gain	No	No
A2	chr20	42223332	42839717	IFT52	Gain	No	No
A2	chr20	43243174	43150726	PKIG	Gain	No	No
A2	chr20	43385538	43113170	RIMS4	Gain	No	No

A2	chr20	43937081	44054885	MATN4,RBPJL	Gain	No	No
A2	chr20	44539790	44486048	PLTP	Gain	No	No
A2	chr20	44746905	44987402	CD40	Gain	No	No
A2	chr20	44997529	45035271	ELMO2	Gain	Yes	No
A2	chr20	45618639	45242364	EYA2	Gain	No	No
A2	chr20	46252654	46281816	NCOA3	Gain	No	No
A2	chr20	46288142	46415360	SULF2	Gain	No	No
A2	chr20	49204338	49236669	FAM65C	Gain	No	No
A2	chr22	50954875	50928073	NCAPH2	Gain	No	No
A2	chr4	72222724	73434516	SLC4A4	Gain	No	No
A2	chr4	72429509	74486287	SLC4A4	Gain	No	No
A2	chr4	72611867	73013918	GC	Gain	Yes	No
A2	chr4	72649677	73935472	GC	Gain	Yes	No
A2	chr4	75023828	76439640	MTHFD2L	Gain	No	No
A2	chr4	75178734	76587233	EPGN	Gain	No	No
A2	chr4	76836020	76928641	NAAA	Gain	No	No
A2	chr4	76997033	77232283	ART3	Gain	No	No
A2	chr4	77025749	77069668	ART3	Gain	No	No
A2	chr4	77084377	77228142	SCARB2	Gain	No	No
A2	chr4	77476761	76551201	SHROOM3	Gain	No	No
A2	chr5	144941	254621	PLEKHG4B	Gain	No	No
A2	chr5	446264	443258	EXOC3	Gain	Yes	No
A2	chr5	1053463	1112172	SLC12A7	Gain	No	No
A2	chr5	1214067	1633021	SLC6A19	Gain	No	No
A2	chr8	37620100	37963256	PROSC	Gain	No	No
A2	chr8	38090504	38034248	DDHD2	Gain	No	No
A2	chr8	38132560	38205733	WHSC1L1	Gain	No	No
A2	chr8	38271435	38315052	FGFR1	Gain	No	No
A2	chr8	38373926	38846181	C8orf86	Gain	No	No
A2	chr8	41519393	41555639	ANK1	Gain	No	No
A2	chr8	42046451	42129723	PLAT	Gain	No	No
A2	chr8	42286275	42743014	SLC20A2	Gain	No	No
A2	chr8	43002090	43173765	HGSNAT	Gain	No	No
A2	chr8	141542514	141595410	AGO2	Gain	No	No

A2	chr8	144885816	144896288	SCRIB	Gain	No	No
A2	chr8	144919927	145067583	NRBP2	Gain	No	No
A2	chr8	145107071	145140646	OPLAH	Gain	No	No
A2	chr8	145577437	145545071	TMEM249	Gain	No	No
A2	chr8	145608281	145538385	ADCK5	Gain	No	No
A2	chr8	145618445	145634556	ADCK5	Gain	No	No
A2	chr8	145641193	145732555	SLC39A4	Gain	No	No
A2	chr8	145655786	145742574	TONSL	Gain	No	No
A2	chr9	131380265	131860427	SPTAN1	Gain	No	No
A2	chr9	139905637	139917504	ABCA2	Gain	No	No
A2	chrX	69510540	70127660	KIF4A	Gain	No	No
A2	chrX	70375063	70679558	NLGN3	Gain	No	No
A2	chr1	103380259	103548528	COL11A1	Loss	Yes	Yes
A2	chr1	115399852	115487076	SYCP1	Loss	No	No
A2	chr10	78647048	78870101	KCNMA1	Loss	No	No
A2	chr13	19997018	20077417	TPTE2	Loss	Yes	No
A2	chr13	20567202	20304484	ZMYM2	Loss	No	No
A2	chr13	21148518	21925418	IFT88	Loss	No	No
A2	chr13	21553829	21476913	LATS2	Loss	No	No
A2	chr13	22069299	23985879	MICU2	Loss	No	No
A2	chr13	25255699	25285547	ATP12A	Loss	No	No
A2	chr13	25458100	25487229	CENPJ	Loss	No	No
A2	chr13	25825786	25453433	MTMR6	Loss	No	No
A2	chr13	26828755	28931822	CDK8	Loss	Yes	No
A2	chr13	27216397	26586760	WASF3	Loss	No	No
A2	chr13	28748408	29898848	PAN3	Loss	No	No
A2	chr13	31712543	31898032	HSPH1	Loss	No	No
A2	chr13	32605436	32973809	FRY,FRY-AS1	Loss	Yes	No
A2	chr13	35615069	45150701	NBEA	Loss	No	Yes
A2	chr13	36804911	36801518	CCDC169,CCDC169-SOHLH2	Loss	No	No
A2	chr13	37583450	37625720	EXOSC8,SUPT20H	Loss	No	No
A2	chr13	38138655	38357497	POSTN	Loss	No	No
A2	chr13	38924135	40301685	UFM1	Loss	No	No
A2	chr13	39586219	39621289	PROSER1	Loss	No	No

A2	chr13	42144603	42486304	VWA8	Loss	No	No
A2	chr13	42622897	42793930	DGKH	Loss	No	No
A2	chr13	43787665	43987124	ENOX1	Loss	No	No
A2	chr13	45517576	45768889	NUFIP1	Loss	No	No
A2	chr13	46539414	46733821	ZC3H13	Loss	No	No
A2	chr13	48881415	49957077	RB1	Loss	No	No
A2	chr13	50276521	21999817	KPNA3	Loss	No	No
A2	chr13	51463744	51608295	RNASEH2B-AS1	Loss	No	No
A2	chr13	51528040	52701640	RNASEH2B	Loss	No	No
A2	chr13	51825603	52004519	FAM124A	Loss	No	No
A2	chr13	53226830	53313947	SUGT1	Loss	No	No
A2	chr13	53624103	60738119	OLFM4	Loss	Yes	No
A2	chr13	77625131	77756057	MYCBP2	Loss	No	No
A2	chr13	78129991	78216943	SCEL	Loss	No	No
A2	chr13	96453835	95050895	UGGT2	Loss	No	No
A2	chr13	99481560	99607805	DOCK9	Loss	No	No
A2	chr13	101710290	108519460	NALCN,NALCN-AS1	Loss	Yes	No
A2	chr13	103275226	103328765	TPP2	Loss	No	No
A2	chr13	107209395	109831981	ARGLU1	Loss	No	No
A2	chr16	34740696	46723682	LOC100130700	Loss	No	No
A2	chr17	26085906	26125908	NOS2	Loss	No	No
A2	chr17	26369687	27005981	NLK	Loss	No	No
A2	chr17	27998952	27857637	SSH2	Loss	No	No
A2	chr17	29483000	29687721	NF1	Loss	No	No
A2	chr17	29849377	30791138	RAB11FIP4	Loss	No	No
A2	chr17	30804538	31203902	PSMD11	Loss	No	No
A2	chr17	31342988	28268856	ASIC2	Loss	No	No
A2	chr17	33310020	33332088	LIG3	Loss	No	No
A2	chr17	35880281	35960450	SYNRG	Loss	Yes	No
A2	chr17	36719062	38802099	SRCIN1	Loss	No	No
A2	chr17	38024454	38068750	ZPBP2	Loss	No	No
A2	chr17	38546216	39183454	TOP2A	Loss	No	No
A2	chr18	76829396	77208984	ATP9B	Loss	No	No
A2	chr21	19647513	19775970	TMPRSS15	Loss	No	No

A2	chr21	27484295	30426807	APP	Loss	No	No
A2	chr21	28304322	32639299	ADAMTS5	Loss	No	No
A2	chr21	30303476	30442658	LTN1	Loss	Yes	No
A2	chr21	30925865	30909706	GRIK1	Loss	No	No
A2	chr21	35107348	35206728	ITSN1	Loss	No	No
A2	chr21	38459546	38572625	TTC3	Loss	No	No
A2	chr21	40568423	40670508	BRWD1	Loss	No	No
A2	chr3	361365	443381	CHL1	Loss	Yes	No
A2	chr3	1189610	1445292	CNTN6	Loss	No	No
A2	chr3	2613099	3095659	CNTN4	Loss	No	No
A2	chr3	3194139	3189387	CRBN	Loss	No	No
A2	chr3	4558159	4508966	ITPR1	Loss	No	No
A2	chr3	25639395	25705863	RARB	Loss	No	No
A2	chr3	26751003	27493989	LRRC3B	Loss	No	No
A2	chr3	30819670	32411813	GADL1	Loss	No	No
A2	chr4	41941212	109087953	TMEM33	Loss	No	No
A2	chr4	78526977	79367787	CXCL13	Loss	No	No
A2	chr4	79367880	79833341	FRAS1	Loss	No	No
A2	chr4	80898774	80992808	ANTXR2	Loss	No	No
A2	chr4	81256873	78740544	C4orf22	Loss	No	No
A2	chr4	82013539	82074875	PRKG2	Loss	No	No
A2	chr4	83989590	84255966	COPS4	Loss	No	No
A2	chr4	85598351	85781775	WDFY3	Loss	No	No
A2	chr4	87869649	87732261	AFF1	Loss	No	No
A2	chr4	88239480	88375607	HSD17B13	Loss	No	No
A2	chr4	88940609	89625761	PKD2	Loss	No	No
A2	chr4	89015728	95583688	ABCG2	Loss	No	No
A2	chr4	89199295	89364249	PPM1K	Loss	No	No
A2	chr4	89772170	89427319	FAM13A	Loss	No	No
A2	chr4	90816051	95206220	MMRN1	Loss	No	No
A2	chr4	93225549	94690601	GRID2	Loss	Yes	No
A2	chr4	96091304	94006430	UNC5C	Loss	No	No
A2	chr4	100125878	100208190	ADH6,LOC100507053	Loss	No	No
A2	chr4	100434145	100815157	C4orf17	Loss	No	No

A2	chr4	101950322	102117273	PPP3CA	Loss	No	No
A2	chr4	103446668	103533763	NFKB1	Loss	No	No
A2	chr4	103790134	103826807	CISD2	Loss	No	No
A2	chr4	104003237	105413216	BDH2	Loss	No	No
A2	chr4	106638749	106639196	GSTCD	Loss	No	No
A2	chr4	107016638	107957453	TBCK	Loss	No	No
A2	chr4	109663201	109684235	ETNPPL	Loss	No	No
A2	chr4	110384056	110402937	SEC24B	Loss	No	No
A2	chr4	110737308	110723335	GAR1	Loss	No	No
A2	chr4	111543411	114680570	PITX2	Loss	No	No
A2	chr4	113970784	113825670	ANK2	Loss	No	No
A2	chr4	115577914	119273922	UGT8	Loss	No	No
A2	chr4	119444473	119632077	CEP170P1	Loss	No	No
A2	chr4	120160372	123350947	USP53	Loss	No	No
A2	chr4	120980578	122085495	MAD2L1	Loss	No	No
A2	chr4	121615928	122683047	PRDM5	Loss	No	No
A2	chr4	122607442	122737602	ANXA5	Loss	No	No
A2	chr4	122748881	122791652	BBS7	Loss	Yes	No
A2	chr4	125631154	128802319	ANKRD50	Loss	No	No
A2	chr4	126237566	126414087	FAT4	Loss	Yes	No
A2	chr4	129012155	130014764	LARP1B	Loss	No	No
A2	chr4	139100372	140196546	SLC7A11	Loss	No	No
A2	chr4	140058783	140307384	ELF2	Loss	No	No
A2	chr4	141311781	141334241	CLGN	Loss	No	No
A2	chr4	141832297	144387378	RNF150	Loss	No	No
A2	chr4	144498560	146017263	FREM3	Loss	No	No
A2	chr4	146686130	152096826	ZNF827	Loss	No	No
A2	chr4	147179843	149358014	SLC10A7	Loss	No	No
A2	chr4	148559533	151505845	PRMT10	Loss	No	No
A2	chr4	148743877	148985658	ARHGAP10	Loss	No	No
A2	chr4	151199004	151850217	LRBA	Loss	No	No
A2	chr4	152198324	153021969	SH3D19,PRSS48	Loss	No	No
A2	chr4	152487289	153809459	FAM160A1	Loss	No	No
A2	chr4	153562079	155490951	TMEM154	Loss	No	No

A2	chr4	154191489	155312449	TRIM2		Loss	No	No
A2	chr4	154330122	154556723	MND1		Loss	No	No
A2	chr4	155506428	155533902	FGA		Loss	No	No
A2	chr4	159091398	159629865	FAM198B		Loss	Yes	No
A2	chr4	159747067	159817017	FNIP2		Loss	No	No
A2	chr4	160188997	164050500	RAPGEF2		Loss	No	No
A2	chr4	164466747	163032564		1-Mar	Loss	No	No
A2	chr4	166385541	166150065	CPE		Loss	No	No
A2	chr4	166960490	169433563	TLL1		Loss	No	No
A2	chr4	170321375	170523829	NEK1		Loss	Yes	No
A2	chr4	170652854	174239710	C4orf27		Loss	No	No
A2	chr4	172735562	186611765	GALNTL6		Loss	No	No
A2	chr4	175220222	175750466	CEP44		Loss	No	No
A2	chr4	177017613	176733400	WDR17		Loss	No	No
A2	chr4	184570603	184629727	RWDD4		Loss	No	No
A2	chr4	185940082	186370821	HELT		Loss	No	No
A2	chr4	186168447	186285120	SNX25		Loss	No	No
A2	chr4	187075621	187130426	FAM149A		Loss	No	No
A2	chr4	187188289	187476537	F11		Loss	No	No
A2	chr4	187525530	187210156	FAT1		Loss	No	No
A2	chr5	49963904	50130849	PARP8		Loss	No	No
A2	chr5	52978947	55213165	NDUFS4		Loss	No	No
A2	chr5	53409031	66458616	ARL15		Loss	No	No
A2	chr5	54466359	54993820	CDC20B		Loss	No	No
A2	chr5	55238497	55272121	IL6ST		Loss	No	No
A2	chr5	57749809	57755966	PLK2		Loss	No	No
A2	chr5	59899217	59895087	DEPDC1B		Loss	No	No
A2	chr5	63496634	64863442	RNF180		Loss	No	No
A2	chr5	64023933	64850773	SREK1IP1		Loss	No	No
A2	chr5	64466443	64769779	ADAMTS6		Loss	No	No
A2	chr5	64931127	64920187	TRAPPC13		Loss	No	No
A2	chr5	65018022	65473486	SGTB		Loss	No	No
A2	chr5	66212810	72383580	MAST4		Loss	No	No
A2	chr5	70818115	70858347	BDP1		Loss	No	No

A2	chr5	70888752	70806978	MCCC2	Loss	No	No
A2	chr5	72980665	72875200	ARHGEF28	Loss	No	No
A2	chr5	74017028	73932323	HEXB	Loss	No	No
A2	chr5	74064755	74137504	NSA2	Loss	No	No
A2	chr5	74323288	74655964	GCNT4	Loss	Yes	No
A2	chr5	74970023	74807454	POC5	Loss	No	No
A2	chr5	75008200	74893614	POC5	Loss	No	No
A2	chr5	75427474	167798538	SV2C	Loss	No	No
A2	chr5	76173498	75998415	S100Z	Loss	No	No
A2	chr5	76251477	78359610	CRHBP	Loss	No	No
A2	chr5	76621363	76607878	PDE8B	Loss	No	No
A2	chr5	77805606	78265015	LHFPL2	Loss	No	No
A2	chr5	79286416	79379107	MTX3	Loss	No	No
A2	chr5	80256507	81549248	RASGRF2	Loss	No	No
A2	chr5	80625946	81591363	ACOT12	Loss	No	No
A2	chr5	82940181	83680685	HAPLN1	Loss	Yes	No
A2	chr5	88056804	90460033	MEF2C	Loss	No	No
A2	chr5	93076014	94990071	FAM172A	Loss	No	No
A2	chr5	93964515	94786144	ANKRD32	Loss	No	No
A2	chr5	94044216	93388932	MCTP1	Loss	No	No
A2	chr5	94417129	101834720	MCTP1	Loss	No	No
A2	chr5	94994019	95768985	SPATA9	Loss	No	No
A2	chr5	96096513	96139646	CAST	Loss	No	No
A2	chr5	96215267	96251473	ERAP2	Loss	No	No
A2	chr5	96271345	96363518	LOC101929747	Loss	No	No
A2	chr5	98115283	98262238	RGMB	Loss	No	No
A2	chr5	99897757	102363942	FAM174A	Loss	No	No
A2	chr5	102465009	102530726	PPIP5K2	Loss	No	No
A2	chr5	107216737	110820748	FBXL17	Loss	No	No
A2	chr5	108717203	110408630	PJA2	Loss	No	No
A2	chr5	112102022	111755010	APC	Loss	Yes	No
A2	chr5	112238076	114506919	REEP5	Loss	No	No
A2	chr5	114552561	114956377	PGGT1B	Loss	No	No
A2	chr5	115423193	115840678	COMMD10	Loss	No	No

A2	chr5	118433673	122754209	DMXL1		Loss	No	No
A2	chr5	122881110	122941056	CSNK1G3		Loss	No	No
A2	chr5	126213876	126791299		3-Mar	Loss	No	No
A2	chr5	127448505	127520548	SLC12A2		Loss	No	No
A2	chr5	127597427	128369335	FBN2		Loss	Yes	Yes
A2	chr5	129240522	130772074	CHSY3		Loss	No	No
A2	chr5	130982782	129070802	FNIP1		Loss	No	No
A2	chr5	131295228	131607159	ACSL6		Loss	No	No
A2	chr5	131785341	132101221	C5orf56		Loss	No	No
A2	chr5	132219031	133655188	AFF4		Loss	Yes	No
A2	chr5	134229181	59481514	TXNDC15		Loss	No	No
A2	chr5	134914404	135290723	CXCL14		Loss	Yes	No
A2	chr5	135272282	137354835	FBXL21		Loss	Yes	No
A2	chr5	136315020	146833260	SPOCK1		Loss	No	No
A2	chr5	137666675	137488449	CDC25C		Loss	No	No
A2	chr5	138117611	137763775	CTNNA1		Loss	No	No
A2	chr5	141249081	141316922	PCDH1		Loss	No	No
A2	chr5	142150291	142593653	ARHGAP26		Loss	No	No
A2	chr5	145198941	145895676	PRELID2		Loss	No	No
A2	chr5	145317385	145888808	SH3RF2		Loss	No	No
A2	chr5	147276546	147822399	C5orf46		Loss	No	No
A2	chr5	149384413	149430024	HMGXB3		Loss	Yes	No
A2	chr5	149562330	151304397	CDX1		Loss	No	No
A2	chr5	149576604	149669403	SLC6A7		Loss	No	No
A2	chr5	150701606	151043796	SLC36A2		Loss	No	No
A2	chr5	153374478	153429577	FAM114A2		Loss	No	No
A2	chr5	156712348	156817676	CYFIP2		Loss	No	No
A2	chr5	158134986	158526788	EBF1		Loss	Yes	No
A2	chr5	159831446	161322874	SLU7		Loss	No	No
A2	chr5	159996502	160115101	ATP10B		Loss	No	No
A2	chr5	166711842	180377906	TENM2		Loss	No	No
A2	chr5	168096787	168678463	SLIT3		Loss	No	No
A2	chr5	168690604	169673235	SLIT3		Loss	No	No
A2	chr5	169309556	169931637	DOCK2,FAM196B		Loss	No	No

A2	chr5	170305100	170723018	RANBP17	Loss	No	No
A2	chr5	176307869	176323186	UNC5A	Loss	Yes	No
A2	chr5	177034302	177309753	B4GALT7	Loss	No	No
A2	chr5	178032303	178194336	CLK4	Loss	No	No
A2	chr5	179192326	178634716	MAML1	Loss	Yes	No
A2	chr8	1244293	6420784	DLGAP2	Loss	No	No
A2	chr8	1833766	6690422	ARHGEF10	Loss	No	No
A2	chr8	6312663	4852328	MCPH1	Loss	No	No
A2	chr8	17159662	16976284	MTMR7	Loss	No	No
A2	chr8	17581180	18666405	MTUS1	Loss	No	No
A2	chr8	17793097	23060274	PCM1	Loss	No	No
A2	chr8	19674917	20038598	INTS10	Loss	No	No
A2	chr8	20110292	20075793	LZTS1	Loss	No	No
A2	chr8	21824367	22009513	XPO7	Loss	No	No
A2	chr8	23540416	24193612	NKX3-1	Loss	No	No
A2	chr8	24196978	25202990	ADAM28	Loss	No	No
A2	chr8	24771273	24261601	NEFM	Loss	No	No
A2	chr8	25290019	26492416	KCTD9	Loss	No	No
A2	chr8	27348684	27401761	EPHX2	Loss	No	No
A2	chr8	27468117	27690650	CLU	Loss	No	No
A2	chr8	28932797	29053716	KIF13B	Loss	No	No
A2	chr8	30404808	30538554	RBPMS	Loss	No	No
A2	chr9	5029782	5126446	JAK2	Loss	Yes	No
A2	chr9	5361757	5892551	PLGRKT	Loss	No	No
A2	chr9	6534707	7170920	GLDC	Loss	No	No
A2	chr9	8319830	8733946	PTPRD	Loss	No	No
A2	chr9	13108934	13250371	MPDZ	Loss	No	No
A2	chr9	14719731	15458065	CER1	Loss	No	No
A2	chr9	14740146	14869242	FREM1	Loss	No	No
A2	chr9	15175016	15214247	TTC39B	Loss	No	No
A2	chr9	15466745	15506635	SNAPC3,PSIP1	Loss	No	No
A2	chr9	15563975	14674383	CCDC171	Loss	No	No
A2	chr9	16435552	33568891	BNC2	Loss	No	No
A2	chr9	19290701	19787017	DENND4C	Loss	No	No

A2	chr9	20715320	27258517	FOCAD	Loss	No	No
A2	chr9	20716103	21971207	FOCAD	Loss	No	No
A2	chr9	26861063	26947144	CAAP1	Loss	No	No
A2	chr9	32405482	32449079	ACO1	Loss	No	No
A2	chr9	32459368	32526322	DDX58	Loss	No	No
A2	chr9	35161988	34990809	UNC13B	Loss	No	No
A2	chr9	35800705	35810129	NPR2	Loss	Yes	No
A2	chr9	36052261	36846926	RECK	Loss	No	No
A2	chr9	37692634	37762114	FRMPD1	Loss	No	No
A2	chr9	38395736	38620360	ALDH1B1	Loss	No	No
A2	chr9	39078381	39178357	CNTNAP3	Loss	No	No
A2	chrX	36083759	37706889	CHDC2	Loss	No	No
A2	chrX	36303743	36403434	CXorf30	Loss	No	No
A2	chrY	6893075	6959724	TBL1Y	Loss	No	No
A2	chrY	9368074	15591197	TSPY10	Loss	No	No
A2	chrY	14518594	15016892	GYG2P1	Loss	No	No
A2	chrY	14821320	14969586	USP9Y	Loss	No	No
A2	chrY	21635790	21764197	BCORP1	Loss	No	No
A3	chr1	145517272	145555822	PEX11B	Gain	No	No
A3	chr1	150297352	150325704	PRPF3	Gain	No	No
A3	chr1	150598117	150477209	ENSA	Gain	No	No
A3	chr1	150900179	150937220	SETDB1	Gain	No	No
A3	chr1	151106876	151091007	SEMA6C	Gain	No	No
A3	chr1	151137554	151318809	LYSMD1,TNFAIP8L2-SCNM1	Gain	No	No
A3	chr1	153614721	153800824	CHTOP	Gain	No	No
A3	chr1	153635180	153719839	ILF2	Gain	No	No
A3	chr1	153941809	153958853	CREB3L4	Gain	No	No
A3	chr1	154164104	154310020	TPM3	Gain	No	No
A3	chr1	155307879	155532324	ASH1L	Gain	No	No
A3	chr1	155719448	155719422	MSTO2P	Gain	No	No
A3	chr1	155880446	155912625	RIT1	Gain	No	No
A3	chr1	161199455	161145885	TOMM40L	Gain	No	No
A3	chr1	234367162	235612283	SLC35F3	Gain	No	No
A3	chr11	66631244	66605916	PC	Gain	No	No

A3	chr11	67290016	67267480	CABP2	Gain	No	No
A3	chr11	67763081	68582955	UNC93B1	Gain	No	No
A3	chr11	68125117	68216743	LRP5	Gain	No	No
A3	chr11	68452391	68671303	GAL	Gain	No	No
A3	chr14	50053002	50110388	RPS29	Gain	No	No
A3	chr17	37571278	37673809	MED1	Gain	No	No
A3	chr17	41131566	41116515	PTGES3L,PTGES3L-AARSD1	Gain	No	No
A3	chr19	2097250	4224811	IZUMO4	Gain	No	No
A3	chr19	2477026	2425265	GADD45B	Gain	No	No
A3	chr19	3820179	3920796	ZFR2	Gain	No	No
A3	chr19	4292224	4323433	TMIGD2	Gain	No	No
A3	chr19	7075006	6417098	ZNF557	Gain	No	No
A3	chr19	8170991	8196172	FBN3	Gain	No	No
A3	chr19	8649778	8670694	ADAMTS10	Gain	No	No
A3	chr19	10091481	10107365	COL5A3	Gain	No	No
A3	chr19	10116748	11135114	COL5A3	Gain	No	No
A3	chr19	10226154	10394996	EIF3G	Gain	No	No
A3	chr19	10556893	10543247	PDE4A	Gain	No	No
A3	chr19	10939648	11266484	DNM2	Gain	No	No
A3	chr19	11141405	11032119	SMARCA4	Gain	Yes	No
A3	chr19	12917427	13060222	RNASEH2A	Gain	No	No
A3	chr19	13319569	13373647	CACNA1A	Gain	No	No
A3	chr19	13470419	12985766	CACNA1A	Gain	No	No
A3	chr19	16301330	16628152	FAM32A	Gain	No	No
A3	chr19	16859949	17169686	NWD1	Gain	No	No
A3	chr19	17010285	17108148	CPAMD8	Gain	No	No
A3	chr19	17170806	17324104	HAUS8	Gain	No	No
A3	chr19	17626981	17571725	PGLS	Gain	No	No
A3	chr19	17662594	17720908	FAM129C	Gain	No	No
A3	chr19	17750244	17919183	UNC13A	Gain	No	No
A3	chr19	17831744	17893981	MAP1S	Gain	Yes	No
A3	chr19	18054316	18197742	CCDC124	Gain	No	No
A3	chr19	18085900	17948872	KCNN1	Gain	No	No
A3	chr19	19206899	19145047	SLC25A42	Gain	No	No

A3	chr19	33090877	33134105	ANKRD27		Gain	No	No
A3	chr19	33464089	33701804	C19orf40		Gain	No	No
A3	chr19	33481419	33535270	RHPN2		Gain	No	No
A3	chr19	36321745	36340049	NPHS1		Gain	No	No
A3	chr19	39023129	39078204	RYR1		Gain	Yes	No
A3	chr19	39083921	39127595	MAP4K1		Gain	No	No
A3	chr19	40993603	41078098	SPTBN4		Gain	No	No
A3	chr19	47844041	48735067	C5AR2		Gain	No	No
A3	chr19	47856010	47884581	DHX34		Gain	No	No
A3	chr19	48182577	48389654	GLTSCR1		Gain	No	No
A3	chr19	49230263	49310331	RASIP1		Gain	No	No
A3	chr19	49961742	50029685	ALDH16A1		Gain	No	No
A3	chr19	50464262	50764892	SIGLEC11		Gain	No	No
A3	chr19	55693383	55718567	PTPRH		Gain	No	No
A3	chr5	908469	2752578	TRIP13		Gain	No	No
A3	chr5	33549311	33892124	ADAMTS12		Gain	No	No
A3	chr5	33951510	34925787	SLC45A2		Gain	No	No
A3	chr5	35068317	34829902	PRLR		Gain	Yes	No
A3	chr5	35617988	37752774	SPEF2		Gain	No	No
A3	chr5	35691138	35814713	SPEF2		Gain	No	No
A3	chr5	35965487	36227667	UGT3A1		Gain	No	No
A3	chr5	36241460	36302011	NADK2		Gain	No	No
A3	chr5	36953719	37371228	NIPBL		Gain	No	No
A3	chr5	37108394	37247847	C5orf42		Gain	Yes	No
A3	chr5	38482690	38463113	LIFR		Gain	No	No
A3	chr5	38869133	39364655	OSMR		Gain	No	No
A3	chr5	38942934	39203089	RICTOR		Gain	No	No
A3	chr5	39375115	39394523	DAB2		Gain	No	No
A3	chr5	40728446	41870294	TTC33		Gain	No	No
A3	chr5	40764615	40798297	PRKAA1		Gain	No	No
A3	chr5	40775029	41870791	PRKAA1		Gain	No	No
A3	chr5	43453759	43556101	C5orf28		Gain	No	No
A3	chr5	43609244	43412488	NNT		Gain	No	No
A3	chr5	133295184	133959727	C5orf15		Gain	No	No

A3	chr5	134029416	134344671	SEC24A	Gain	No	No
A3	chr5	171479932	171615346	STK10	Gain	No	No
A3	chr5	175305654	176719159	CPLX2	Gain	No	No
A3	chr5	176516550	176489152	FGFR4	Gain	No	No
A3	chr5	178504071	179037027	ZNF354C	Gain	No	No
A3	chr5	179029064	179072047	RUFY1	Gain	No	No
A3	chr5	179228783	179285840	MGAT4B	Gain	No	No
A3	chr5	179442302	180017838	RNF130	Gain	No	No
A3	chr7	591025	767313	PRKAR1B	Gain	No	No
A3	chr7	766846	300740	PRKAR1B,HEATR2	Gain	No	No
A3	chr7	891586	912199	SUN1	Gain	No	No
A3	chr7	1040105	2269778	C7orf50	Gain	No	No
A3	chr7	1578784	2608633	MAFK	Gain	No	No
A3	chr7	1606969	1656328	PSMG3	Gain	No	No
A3	chr7	1783939	2281833	ELFN1	Gain	No	No
A3	chr7	2472197	2419146	CHST12	Gain	No	No
A3	chr7	2568507	4901625	LFNG	Gain	No	No
A3	chr7	2646757	2998265	IQCE	Gain	No	No
A3	chr7	4998601	4949409	MMD2	Gain	No	No
A3	chr7	5028693	5254335	RNF216P1	Gain	Yes	No
A3	chr7	5348505	6441658	TNRC18	Gain	No	No
A3	chr7	5939914	6094335	CCZ1	Gain	Yes	No
A3	chr7	6150755	6048737	USP42	Gain	No	No
A3	chr7	6204903	4056984	CYTH3	Gain	No	No
A3	chr7	6629914	6663921	C7orf26	Gain	No	No
A3	chr7	7612066	12270741	MIOS	Gain	No	No
A3	chr7	8152814	7680806	ICA1	Gain	No	No
A3	chr7	19765136	20687271	TMEM196	Gain	No	No
A3	chr7	20193815	20449401	MACC1	Gain	No	No
A3	chr7	20826396	21985542	SP8	Gain	No	No
A3	chr7	22478037	23053770	STEAP1B	Gain	No	No
A3	chr7	24663284	24727498	MPP6	Gain	Yes	No
A3	chr7	24843933	24932240	OSBPL3	Gain	No	No
A3	chr7	24873705	24784373	OSBPL3	Gain	No	No

A3	chr7	26403944	27136007	SNX10	Gain	No	No
A3	chr7	26709709	26904341	SKAP2	Gain	No	No
A3	chr7	27570810	27867439	HIBADH	Gain	No	No
A3	chr7	27855967	28857796	TAX1BP1	Gain	No	No
A3	chr7	28725720	29943978	CREB5	Gain	No	No
A3	chr7	29035246	29160687	LOC100506497	Gain	No	No
A3	chr7	29237356	29549022	CHN2	Gain	No	No
A3	chr7	30029797	30113748	SCRN1	Gain	No	No
A3	chr7	32527296	32623779	LSM5	Gain	No	No
A3	chr7	32529636	33075600	LSM5	Gain	No	No
A3	chr7	33019975	33016111	FKBP9	Gain	No	No
A3	chr7	34097670	35225774	BMPER	Gain	No	No
A3	chr7	39446198	40132836	POU6F2	Gain	Yes	No
A3	chr7	43927013	43663219	URGCP,URGCP-MRPS24	Gain	No	No
A3	chr7	43992119	45067396	UBE2D4,POLR2J4	Gain	No	No
A3	chr7	44687042	44530385	OGDH	Gain	No	No
A3	chr7	56020871	56148365	MRPS17	Gain	No	No
A3	chr7	72880625	74016920	BAZ1B	Gain	No	No
A3	chr7	73510951	73535379	LIMK1	Gain	Yes	No
A3	chr7	74824182	75659842	SPDYE5,GATSL2	Gain	No	No
A3	chr7	75168642	74573201	HIP1	Gain	No	No
A3	chr7	100331649	100274196	ZAN	Gain	No	No
A3	chr7	100802339	100861011	AP1S1	Gain	No	No
A3	chrX	90689901	99957103	PABPC5	Gain	No	No
A3	chrX	96173507	96684744	DIAPH2	Gain	No	No
A3	chrX	98974476	100297301	XRCC6P5	Gain	No	No
A3	chrX	99885755	99926296	TSPAN6	Gain	No	No
A3	chrX	100356046	100417978	CENPI	Gain	No	No
A3	chrX	100530209	100548059	TAF7L	Gain	No	No
A3	chrX	100650715	100611256	RPL36A,RPL36A-HNRNPH2	Gain	No	No
A3	chrX	101153091	101098277	ZMAT1	Gain	No	No
A3	chrX	106061892	106145445	TBC1D8B	Gain	No	No
A3	chrX	107681171	107938669	COL4A6	Gain	No	No
A3	chrX	108619126	108926601	GUCY2F	Gain	No	No

A3	chrX	112065482	114542123	AMOT		Gain	No	No
A3	chrX	114357089	114882337	LRCH2		Gain	No	No
A3	chrX	115303498	117910474	AGTR2		Gain	No	No
A3	chrX	117117442	117820123	KLHL13		Gain	No	No
A3	chrX	118763280	118759342		6-Sep	Gain	No	No
A3	chrX	118780700	119010629		6-Sep	Gain	No	No
A3	chr1	72058499	77672416	NEGR1		Loss	No	No
A3	chr1	86488220	87549962	COL24A1		Loss	Yes	No
A3	chr1	103387073	103468347	COL11A1		Loss	Yes	Yes
A3	chr1	103471393	103574052	COL11A1		Loss	Yes	Yes
A3	chr1	104068577	104094414	RNPC3		Loss	No	No
A3	chr1	104297336	104238307	AMY1C		Loss	No	No
A3	chr11	27528262	30974115	LIN7C		Loss	No	No
A3	chr12	40114588	41410718	C12orf40		Loss	Yes	No
A3	chr12	79679566	82148002	SYT1		Loss	No	No
A3	chr12	80862486	81072504	PTPRQ		Loss	No	No
A3	chr12	83455546	88480275	TMTC2		Loss	No	No
A3	chr12	85408093	88374133	TSPAN19		Loss	No	No
A3	chr18	14763684	14852737	ANKRD30B		Loss	No	No
A3	chr18	29622141	31187640	RNF125		Loss	No	No
A3	chr18	39535198	40503728	PIK3C3		Loss	No	No
A3	chr18	52896077	53254367	TCF4		Loss	No	No
A3	chr2	189842819	189863045	COL3A1,MIR1245A,MIR1245B		Loss	No	No
A3	chr3	108271080	108308491	KIAA1524		Loss	No	No
A3	chr6	64497997	66205500	EYS		Loss	No	No
A3	chr6	69348552	71162274	BAI3		Loss	No	No
A3	chr6	72943475	72926946	RIMS1		Loss	No	No
A3	chr6	75797292	75893860	COL12A1		Loss	Yes	No
A3	chr6	93955015	94129300	EPHA7		Loss	No	No
A3	chr6	96972257	97537866	UFL1		Loss	No	No
A3	chr6	128150620	128718833	THEMIS		Loss	No	No
A3	chr6	146209155	146276490	SHPRH		Loss	No	No
A3	chr7	38429404	38574611	AMPH		Loss	No	No
A3	chr7	38766508	38948800	VPS41		Loss	No	No

A3	chrX	21392535	21667145	CNKSR2	Loss	No	No
A3	chrX	23311130	31986631	LOC100873065	Loss	No	No
A3	chrX	32235032	33146544	DMD	Loss	No	No
A3	chrX	37527620	38037597	LANCL3	Loss	No	No
A4	chr16	585315	703803	CAPN15	Gain	No	No
A4	chr16	707761	772590	WDR90	Gain	No	No
A4	chr16	732952	778424	JMJD8	Gain	No	No
A4	chr16	772581	745923	FAM173A	Gain	No	No
A4	chr16	1550563	1486812	TELO2	Gain	No	No
A4	chr16	2012061	2031550	RPS2	Gain	No	No
A4	chr16	2564080	2579096	ATP6V0C	Gain	No	No
A4	chr16	30127956	30106467	MAPK3	Gain	No	No
A4	chr16	67916860	67943609	EDC4	Gain	No	Yes
A4	chr16	68264743	68344868	ESRP2	Gain	No	No
A4	chr16	89704243	89762772	DPEP1	Gain	No	No
A4	chr19	622148	571579	POLRMT	Gain	No	No
A4	chr19	852290	815081	ELANE	Gain	No	No
A4	chr19	1041223	1065570	ABCA7	Gain	No	No
A4	chr19	1073504	1095391	HMHA1	Gain	No	No
A4	chr19	1298549	1271035	EFNA2	Gain	No	No
A4	chr19	1417498	1357093	DAZAP1	Gain	No	No
A4	chr19	1526901	1578537	PLK5	Gain	No	No
A4	chr19	1952525	1997462	CSNK1G2	Gain	No	No
A4	chr19	2109871	2123405	AP3D1	Gain	No	No
A4	chr19	3544806	3578111	MFSD12	Gain	No	No
A4	chr19	3586492	3626813	GIPC3	Gain	No	No
A4	chr19	3641702	4055245	PIP5K1C	Gain	No	No
A4	chr19	4033097	3961510	PIAS4	Gain	No	No
A4	chr19	4446045	5119863	UBXN6,MIR4746	Gain	No	No
A4	chr19	4659942	4932968	C19orf10	Gain	No	No
A4	chr19	5610649	5691678	SAFB2	Gain	No	No
A4	chr19	7599635	7619158	PNPLA6	Gain	No	No
A4	chr19	7914882	7988136	EVI5L	Gain	No	No
A4	chr19	10420890	10450345	FDX1L	Gain	No	No

A4	chr19	10946278	11266484	TMED1	Gain	No	No
A4	chr19	12825634	12761036	TNPO2	Gain	No	No
A4	chr19	12941095	13059627	RTBDN	Gain	No	No
A4	chr19	18304673	18279356	MPV17L2	Gain	No	No
A4	chr19	19015579	19035117	COPE	Gain	No	No
A4	chr3	38035077	38048191	VILL	Gain	No	No
A4	chr5	176793177	176733960	RGS14	Gain	No	No
A4	chr5	176936802	176918147	DOK3	Gain	No	No
A4	chr7	99724990	99677279	MBLAC1	Gain	No	No
A4	chr8	125052866	125740748	FER1L6-AS1,FER1L6	Gain	No	No
A4	chr8	125332326	125500859	TMEM65	Gain	No	No
A4	chr8	142146656	142199245	DENND3	Gain	No	No
A4	chr8	142354442	143626368	LOC731779	Gain	No	No
A4	chr8	145006104	145066776	PLEC	Gain	No	No
A4	chr8	145668059	145640803	TONSL	Gain	No	No
A4	chr9	140001982	139735639	MAN1B1	Gain	No	No
A4	chr9	140059637	140006682	GRIN1	Gain	No	No
A4	chr9	140126112	140138159	SLC34A3	Gain	No	No
A4	chr9	140138034	140350938	TUBB4B	Gain	No	No
A4	chr10	49383888	49459734	FRMPD2	Loss	No	No
A4	chr10	55568451	56129035	PCDH15	Loss	Yes	No
A4	chr10	89516545	89717776	ATAD1	Loss	No	No
A4	chr10	106121780	106209962	CCDC147	Loss	No	No
A4	chr10	106602549	104853066	SORCS3	Loss	No	No
A4	chr10	108357108	108924466	SORCS1	Loss	No	No
A4	chr10	122263329	123353481	PPAPDC1A	Loss	No	No
A4	chr15	32895569	34145883	GOLGA8R	Loss	No	No
A4	chr15	51675981	52708498	GLDN	Loss	No	No
A4	chr17	10323334	10426502	MYH8	Loss	No	No
A4	chr18	10672686	11148761	PIEZO2	Loss	Yes	No
A4	chr18	61255884	61468246	SERPINB13	Loss	No	No
A4	chr2	9002719	8958923	MBOAT2	Loss	No	No
A4	chr2	226491632	1983549	NYAP2	Loss	No	No
A4	chr21	41414297	42080697	DSCAM	Loss	No	No

A4	chr4	178243612	186611765	NEIL3	Loss	No	No
A4	chr4	184114751	184233621	WWC2	Loss	No	No
A4	chr5	112170647	111643187	APC	Loss	Yes	No
A4	chr5	127640648	127693087	FBN2	Loss	Yes	Yes
A4	chr5	145643053	145836898	RBM27	Loss	No	No
A4	chr8	3205513	4852328	CSMD1	Loss	Yes	No
A4	chrX	79698020	85404112	FAM46D	Loss	No	No
A4	chrX	83319978	86924596	RPS6KA6	Loss	No	No
A5	chr1	117641454	118463047	TTF2	Gain	No	No
A5	chr1	118496287	119683295	WDR3	Gain	No	No
A5	chr1	119441650	120906419	TBX15	Gain	No	No
A5	chr1	120477735	120471835	NOTCH2	Gain	No	Yes
A5	chr1	144930583	145557112	PDE4DIP	Gain	No	No
A5	chr1	144997082	145281704	PDE4DIP	Gain	No	No
A5	chr1	145477066	144995082	LIX1L	Gain	No	No
A5	chr1	145578061	145541915	PIAS3	Gain	No	No
A5	chr1	145715525	145827103	CD160	Gain	Yes	No
A5	chr1	145747041	145312729	PDZK1	Gain	No	No
A5	chr1	146461118	144823985	NBPF12	Gain	No	No
A5	chr1	146986157	147416212	LINC00624	Gain	No	No
A5	chr1	148010112	148346929	NBPF14	Gain	Yes	No
A5	chr1	148338371	149982686	LOC100288142	Gain	No	No
A5	chr1	149287450	150449041	LOC388692	Gain	No	No
A5	chr1	149783501	149858961	HIST2H2BF	Gain	No	No
A5	chr1	149908046	149885739	MTMR11	Gain	No	No
A5	chr1	150245182	150237480	C1orf54	Gain	No	No
A5	chr1	150464820	150486265	TARS2	Gain	No	No
A5	chr1	150533370	150692014	ADAMTSL4	Gain	No	No
A5	chr1	150684308	150830936	HORMAD1	Gain	No	No
A5	chr1	150917393	151018641	SETDB1	Gain	No	No
A5	chr1	150969300	150968114	FAM63A	Gain	No	No
A5	chr1	151026706	151196882	CDC42SE1	Gain	No	No
A5	chr1	151104162	151091007	SEMA6C	Gain	No	No
A5	chr1	151131141	151345210	TNFAIP8L2,TNFAIP8L2-SCNM1	Gain	No	No

A5	chr1	151142994	151147393	TMOD4	Gain	No	No
A5	chr1	151258750	151665959	ZNF687	Gain	No	No
A5	chr1	151413403	151804348	POGZ	Gain	No	No
A5	chr1	151490981	151763010	CGN	Gain	No	No
A5	chr1	151694561	151736040	RIIAD1	Gain	No	No
A5	chr1	151798405	153800824	RORC	Gain	Yes	No
A5	chr1	153599426	153745557	S100A13	Gain	No	No
A5	chr1	153662180	153958853	NPR1	Gain	No	No
A5	chr1	153934826	154235981	SLC39A1	Gain	No	No
A5	chr1	153940997	153916873	CREB3L4	Gain	No	No
A5	chr1	154141780	154144580	TPM3	Gain	No	No
A5	chr1	154309823	154531120	ATP8B2	Gain	No	No
A5	chr1	154458418	154575102	SHE	Gain	No	No
A5	chr1	154685939	154427057	KCNN3	Gain	No	No
A5	chr1	155261546	155271225	PKLR	Gain	No	No
A5	chr1	155270671	155232176	PKLR	Gain	No	No
A5	chr1	155279579	155294600	FDPS	Gain	No	No
A5	chr1	155402970	155706854	ASH1L	Gain	No	No
A5	chr1	155874101	155912625	RIT1	Gain	No	No
A5	chr1	155885663	156213123	KIAA0907	Gain	No	No
A5	chr1	156100407	155584758	LMNA	Gain	No	No
A5	chr1	156211950	156147542	PMF1-BGLAP	Gain	No	No
A5	chr1	156220648	164790863	SMG5	Gain	No	No
A5	chr1	156785797	156830938	NTRK1,SH2D2A	Gain	No	No
A5	chr1	156863522	156777137	PEAR1	Gain	No	No
A5	chr1	156905922	156955965	ARHGEF11	Gain	No	No
A5	chr1	157647977	157767610	FCRL3	Gain	No	No
A5	chr1	158149736	158326381	CD1D	Gain	No	No
A5	chr1	159015086	159913418	IFI16	Gain	No	No
A5	chr1	159032274	159169666	AIM2	Gain	No	No
A5	chr1	159272095	159869906	FCER1A	Gain	Yes	No
A5	chr1	159895239	160065036	TAGLN2	Gain	No	No
A5	chr1	160147200	160183055	ATP1A4	Gain	No	No
A5	chr1	160188113	160232318	DCAF8	Gain	No	No

A5	chr1	160519706	160493052	CD84	Gain	No	No
A5	chr1	160914815	161039760	ITLN2	Gain	No	No
A5	chr1	161014701	161059385	USF1	Gain	No	No
A5	chr1	161130409	161141010	USP21	Gain	No	No
A5	chr1	161183653	161197527	NDUFS2	Gain	No	No
A5	chr1	161275897	161326630	MPZ	Gain	No	No
A5	chr1	161514490	161596192	FCGR3A	Gain	No	No
A5	chr1	162535801	163038782	UAP1	Gain	Yes	No
A5	chr1	164743665	165738159	PBX1,LOC100505795	Gain	Yes	No
A5	chr1	166810191	167874436	POGK	Gain	No	No
A5	chr1	167905796	167905439	MPC2	Gain	No	No
A5	chr1	168161445	169677997	TIPRL	Gain	No	No
A5	chr1	169338710	169337201	BLZF1	Gain	No	No
A5	chr1	169679576	169858031	SELL	Gain	Yes	No
A5	chr1	169767997	170248073	C1orf112	Gain	No	No
A5	chr1	171481170	171763667	PRRC2C	Gain	Yes	No
A5	chr1	172107947	172522510	DNM3,MIR214,DNM3OS	Gain	No	No
A5	chr1	173835772	173886516	GAS5	Gain	No	No
A5	chr1	176998498	178490946	ASTN1	Gain	No	No
A5	chr1	177995289	178876002	LOC730102	Gain	No	No
A5	chr1	178252698	178436556	RASAL2	Gain	No	No
A5	chr1	179311144	206632293	SOAT1	Gain	No	No
A5	chr1	179335538	179821946	AXDND1	Gain	Yes	No
A5	chr1	179561736	179660407	TDRD5	Gain	No	No
A5	chr1	179712297	180167169	FAM163A	Gain	No	No
A5	chr1	179955303	180068120	CEP350	Gain	No	No
A5	chr1	180243071	182357885	LHX4,LOC100527964	Gain	No	No
A5	chr1	181452685	181765994	CACNA1E	Gain	No	No
A5	chr1	182571100	182794955	RGS16	Gain	No	No
A5	chr1	183430900	183605076	SMG7-AS1	Gain	No	No
A5	chr1	183711260	184041432	RGL1	Gain	No	No
A5	chr1	183909714	185121067	COLGALT2	Gain	No	No
A5	chr1	184671944	185245800	EDEM3	Gain	No	No
A5	chr1	185125609	186269345	TRMT1L	Gain	No	No

A5	chr1	186283760	193028523	TPR	Gain	No	No
A5	chr1	186348898	186386786	C1orf27	Gain	No	No
A5	chr1	190762635	193075244	LOC440704	Gain	No	No
A5	chr1	193074393	196577561	GLRX2	Gain	No	No
A5	chr1	193105632	196716634	CDC73	Gain	No	No
A5	chr1	196794606	197036397	CFHR1	Gain	No	No
A5	chr1	197521384	197447585	DENND1B	Gain	No	No
A5	chr1	198828001	200952222	MIR181A1HG	Gain	No	No
A5	chr1	200008785	201687883	NR5A2	Gain	No	No
A5	chr1	201338460	201934671	TNNT2	Gain	No	No
A5	chr1	202149238	202130716	PTPRVP	Gain	No	No
A5	chr1	202172904	201844356	LGR6	Gain	No	No
A5	chr1	202544129	203696699	PPP1R12B	Gain	No	No
A5	chr1	202862366	202920292	KLHL12	Gain	No	No
A5	chr1	203097912	203198860	ADORA1	Gain	No	No
A5	chr1	203702350	205385424	ATP2B4	Gain	No	No
A5	chr1	204966297	207224192	NFASC	Gain	No	No
A5	chr1	205022227	205197899	CNTN2	Gain	No	No
A5	chr1	205064000	205091150	RBBP5	Gain	No	No
A5	chr1	205307622	205351272	KLHDC8A	Gain	No	No
A5	chr1	205313261	205417526	KLHDC8A	Gain	No	No
A5	chr1	205688654	205819276	NUCKS1	Gain	No	No
A5	chr1	206141445	206667324	FAM72A	Gain	No	No
A5	chr1	206648145	206822542	IKBKE	Gain	No	No
A5	chr1	206758511	208391347	RASSF5	Gain	No	No
A5	chr1	206810219	206945839	DYRK3	Gain	No	No
A5	chr1	207224032	207318317	PFKFB2,YOD1	Gain	No	No
A5	chr1	207646889	207959027	CR2	Gain	No	No
A5	chr1	209605477	209796855	MIR205HG	Gain	No	No
A5	chr1	209824261	209908295	LAMB3	Gain	Yes	No
A5	chr1	210536195	210522410	HHAT	Gain	No	No
A5	chr1	210948689	211307457	KCNH1	Gain	Yes	No
A5	chr1	211486061	211544812	RCOR3	Gain	No	No
A5	chr1	211605660	211848972	LINC00467	Gain	No	No

A5	chr1	211836113	212274426	NEK2	Gain	No	No
A5	chr1	212118125	212002665	INTS7	Gain	No	No
A5	chr1	212502476	212532129	PPP2R5A	Gain	No	No
A5	chr1	212617680	212788603	NENF	Gain	No	No
A5	chr1	213170496	218578674	ANGEL2	Gain	No	No
A5	chr1	214169811	215256774	PROX1	Gain	No	No
A5	chr1	215802155	216595882	USH2A	Gain	Yes	No
A5	chr1	217804694	220978616	SPATA17	Gain	No	No
A5	chr1	218536675	220253196	TGFB2	Gain	No	No
A5	chr1	220141941	231954990	EPRS	Gain	No	No
A5	chr1	220291498	222721444	IARS2	Gain	Yes	No
A5	chr1	221915322	223408442	DUSP10	Gain	No	No
A5	chr1	222889025	222895856	BROX	Gain	No	No
A5	chr1	222919872	222885864	FAM177B	Gain	No	No
A5	chr1	224340843	224517891	FBXO28	Gain	No	No
A5	chr1	224585928	224622001	WDR26	Gain	Yes	No
A5	chr1	224868659	226374423	CNIH3	Gain	No	No
A5	chr1	225155381	225616557	DNAH14	Gain	Yes	No
A5	chr1	226109779	227083804	PYCR2	Gain	Yes	No
A5	chr1	226420796	226488906	LIN9	Gain	No	No
A5	chr1	226825379	227842177	ITPKB	Gain	No	No
A5	chr1	227182523	227173041	CDC42BPA	Gain	Yes	No
A5	chr1	228284963	228291022	ARF1	Gain	No	No
A5	chr1	228353174	228596452	IBA57-AS1	Gain	No	No
A5	chr1	228550276	228238622	OBSCN	Gain	Yes	No
A5	chr1	228676660	229424590	RNF187	Gain	No	No
A5	chr1	228873419	230410310	RHOU	Gain	No	No
A5	chr1	229566992	230850336	ACTA1	Gain	No	No
A5	chr1	229763367	230827310	URB2	Gain	No	No
A5	chr1	231003925	231487317	C1orf198	Gain	No	No
A5	chr1	231079551	231762680	TTC13	Gain	No	No
A5	chr1	231856593	235316080	DISC1,TSNAX-DISC1	Gain	No	No
A5	chr1	232002268	235490283	DISC1,TSNAX-DISC1	Gain	No	No
A5	chr1	232162180	235993724	DISC1,TSNAX-DISC1	Gain	No	No

A5	chr1	235505022	236924473	GGPS1	Gain	No	No
A5	chr1	237433796	242511544	RYR2	Gain	No	No
A5	chr1	238025699	247492917	LOC100130331	Gain	No	No
A5	chr1	240966203	241519126	RGS7	Gain	No	No
A5	chr1	243259292	245005560	LOC731275	Gain	No	No
A5	chr1	243291550	244006584	CEP170	Gain	No	No
A5	chr1	244574588	245246990	ADSS	Gain	No	No
A5	chr1	247005995	247202839	AHCTF1	Gain	No	No
A5	chr1	247322307	247320705	ZNF124	Gain	No	No
A5	chr1	249141250	249213345	ZNF672	Gain	No	No
A5	chr10	1230800	5141641	ADARB2	Gain	No	No
A5	chr10	3110818	3109899	PFKP	Gain	No	No
A5	chr10	49393595	50854720	FRMPD2	Gain	No	No
A5	chr10	72201230	72360662	NODAL	Gain	No	No
A5	chr10	73434868	73575704	CDH23	Gain	Yes	No
A5	chr10	75574760	75634349	CAMK2G	Gain	No	No
A5	chr10	79553765	79686348	DLG5	Gain	No	No
A5	chr10	80921809	81070941	ZMIZ1	Gain	No	No
A5	chr10	88702073	88992742	MMRN2	Gain	No	No
A5	chr10	88753118	88277848	AGAP11	Gain	No	No
A5	chr10	93582052	93787057	TNKS2	Gain	No	No
A5	chr10	96336418	96084861	HELLS	Gain	No	No
A5	chr10	97081719	97197312	SORBS1	Gain	No	No
A5	chr10	98133352	99236501	TLL2	Gain	No	No
A5	chr10	98761914	98924647	SLIT1	Gain	No	No
A5	chr10	99640005	99771094	CRTAC1	Gain	No	No
A5	chr10	100010821	102897546	LOXL4	Gain	No	No
A5	chr10	100242392	125806240	HPSE2	Gain	No	No
A5	chr10	102045853	102027437	BLOC1S2	Gain	No	No
A5	chr10	102107820	102289263	SCD	Gain	No	No
A5	chr10	102734741	102790141	SEMA4G	Gain	No	No
A5	chr10	102763263	115312949	LZTS2	Gain	No	No
A5	chr10	103361173	103347163	DPCD	Gain	No	No
A5	chr10	103587616	103871293	KCNIP2,KCNIP2-AS1	Gain	No	No

A5	chr10	104019806	104377185	GBF1	Gain	No	No
A5	chr10	104156009	104162286	NFKB2	Gain	No	No
A5	chr10	104228711	104211300	TMEM180	Gain	No	No
A5	chr10	105254317	106035092	NEURL	Gain	No	No
A5	chr10	105794378	105881954	COL17A1	Gain	No	No
A5	chr10	105939597	106983007	WDR96	Gain	No	No
A5	chr10	106088622	111970073	ITPRIP	Gain	No	No
A5	chr10	111892062	114186617	ADD3	Gain	No	No
A5	chr10	111985761	112630662	MXI1	Gain	No	No
A5	chr10	112696360	113941571	SHOC2	Gain	No	No
A5	chr10	114077636	115423829	GUCY2GP	Gain	No	No
A5	chr10	114710008	114920450	TCF7L2	Gain	Yes	No
A5	chr10	115515017	115535625	PLEKHS1	Gain	No	No
A5	chr10	116056736	115485649	AFAP1L2	Gain	No	No
A5	chr10	116245055	122629426	ABLIM1	Gain	No	No
A5	chr10	117825083	117971157	GFRA1	Gain	No	No
A5	chr10	118435909	128019078	HSPA12A	Gain	No	No
A5	chr10	119001189	118615262	SLC18A2	Gain	No	No
A5	chr10	120101238	120925204	FAM204A	Gain	No	No
A5	chr10	120446109	123353481	CACUL1	Gain	No	No
A5	chr10	121336122	121356541	TIAL1	Gain	No	No
A5	chr10	121341970	121619396	TIAL1	Gain	No	No
A5	chr10	122610686	124216868	WDR11-AS1	Gain	Yes	No
A5	chr10	123549683	123687578	ATE1	Gain	No	No
A5	chr10	124248417	124658230	HTRA1	Gain	No	No
A5	chr10	124697220	124768366	C10orf88	Gain	No	No
A5	chr10	124907637	124922344	HMX2	Gain	No	No
A5	chr10	126370175	134691591	FAM53B	Gain	No	No
A5	chr10	126714650	127417673	CTBP2	Gain	No	No
A5	chr10	127434107	129845947	C10orf137	Gain	No	No
A5	chr10	127524672	127511837	BCCIP	Gain	No	No
A5	chr10	128952115	127727998	DOCK1,FAM196A	Gain	No	No
A5	chr10	129899521	129924020	MKI67	Gain	No	No
A5	chr10	131878000	134166901	LINC00959	Gain	No	No

A5	chr10	134705836	95178	TTC40	Gain	No	No
A5	chr10	135061057	135169009	MIR202	Gain	No	No
A5	chr10	135196355	135367832	PAOX	Gain	No	No
A5	chr12	88256	362913	LOC100288778	Gain	No	No
A5	chr12	520912	1017200	CCDC77	Gain	No	No
A5	chr12	1136913	1225199	ERC1	Gain	No	No
A5	chr12	2950007	2910522	LOC100507424	Gain	No	No
A5	chr12	3103903	3149842	TEAD4	Gain	No	No
A5	chr12	4382901	4665668	CCND2	Gain	No	No
A5	chr12	4599687	4796720	C12orf4	Gain	No	No
A5	chr12	6493198	6451283	LTBR	Gain	No	No
A5	chr12	6571403	6677498	VAMP1,TAPBPL	Gain	No	No
A5	chr12	6673315	6665249	NOP2	Gain	No	No
A5	chr12	6857935	6927708	MLF2	Gain	No	No
A5	chr12	7260903	7362838	C1RL	Gain	No	No
A5	chr12	7509975	7656414	CD163L1	Gain	No	No
A5	chr12	7970426	7870152	SLC2A14	Gain	No	No
A5	chr12	8023774	9833629	SLC2A14	Gain	No	No
A5	chr12	10365488	10565215	GABARAPL1	Gain	No	No
A5	chr12	10532300	10787285	KLRK1,KLRC4-KLRK1	Gain	No	No
A5	chr12	12277498	13366755	LRP6	Gain	No	No
A5	chr12	12482217	13529359	MANSC1	Gain	No	No
A5	chr12	12510019	14706346	LOH12CR1	Gain	No	No
A5	chr12	13714409	15274053	GRIN2B	Gain	No	No
A5	chr12	14923653	15114562	HIST4H4	Gain	No	No
A5	chr12	15273996	16517344	RERG	Gain	No	No
A5	chr12	15776091	16430619	EPS8	Gain	No	No
A5	chr12	18473895	18801352	PIK3C2G	Gain	No	No
A5	chr12	21487521	21807611	SLCO1A2	Gain	Yes	No
A5	chr12	21958931	21958245	ABCC9	Gain	No	No
A5	chr12	24365354	25261269	SOX5	Gain	No	No
A5	chr12	25261222	25348094	LRMP	Gain	No	No
A5	chr12	25347819	27568880	CASC1	Gain	No	No
A5	chr12	26208168	32531141	RASSF8	Gain	No	No

A5	chr12	27450642	27863888	STK38L	Gain	Yes	No
A5	chr12	28410132	29936743	CCDC91	Gain	No	No
A5	chr12	31079837	31256665	TSPAN11	Gain	No	No
A5	chr12	31542257	31648853	DENND5B	Gain	No	No
A5	chr12	32717070	33031966	FGD4	Gain	No	No
A5	chr13	52509728	52657488	ATP7B	Gain	Yes	No
A5	chr15	22925776	22980178	CYFIP1	Gain	No	No
A5	chr15	23441538	23892993	GOLGA8EP	Gain	No	No
A5	chr15	23576069	26108349	LOC440243	Gain	No	No
A5	chr15	42276633	42298319	PLA2G4E	Gain	No	No
A5	chr16	34625677	48265850	LOC283914	Gain	No	No
A5	chr16	46617427	34983208	SHCBP1	Gain	No	No
A5	chr16	48581917	49823457	N4BP1	Gain	No	No
A5	chr16	50300450	25123317	ADCY7	Gain	No	No
A5	chr16	52553317	56388993	TOX3	Gain	No	No
A5	chr16	55358470	55513544	IRX6	Gain	No	No
A5	chr16	55844428	55867075	CES1	Gain	No	No
A5	chr16	56468665	56718108	NUDT21	Gain	No	No
A5	chr16	56938175	57609004	SLC12A3	Gain	No	No
A5	chr16	57849327	58019450	KIFC3,LOC388282	Gain	No	No
A5	chr16	57921758	58633415	CNGB1	Gain	No	No
A5	chr16	58148713	58312541	C16orf80	Gain	No	No
A5	chr16	58197957	57832194	CSNK2A2	Gain	No	No
A5	chr16	58437001	58552447	GINS3	Gain	No	No
A5	chr16	61689373	74455368	CDH8	Gain	No	No
A5	chr16	66822071	66785525	CCDC79	Gain	No	No
A5	chr16	67143914	67195840	C16orf70	Gain	No	No
A5	chr16	67233027	67224107	ELMO3	Gain	No	No
A5	chr16	67323392	67323403	PLEKHG4,KCTD19	Gain	No	No
A5	chr16	67977376	68002597	LCAT	Gain	No	No
A5	chr16	68000655	67943609	SLC12A4	Gain	No	No
A5	chr16	68264291	68391169	ESRP2	Gain	No	No
A5	chr16	69167360	69202937	CIRH1A	Gain	No	No
A5	chr16	69363899	69375223	PDF,COG8	Gain	No	No

A5	chr16	69820898	69975644	WWP2	Gain	No	No
A5	chr16	70497071	70433436	FUK	Gain	No	No
A5	chr16	70569210	70714928	SF3B3	Gain	No	No
A5	chr16	71065313	71264625	HYDIN	Gain	No	No
A5	chr16	71967322	72135511	PKD1L3	Gain	Yes	No
A5	chr16	75298248	75565538	BCAR1	Gain	No	No
A5	chr17	25746134	25976586	TBC1D3P5	Gain	No	No
A5	chr17	26940095	26874867	SGK494,SPAG5-AS1	Gain	No	No
A5	chr17	26943302	26976294	KIAA0100,SPAG5-AS1	Gain	No	No
A5	chr17	26945808	27044012	KIAA0100	Gain	No	No
A5	chr17	27055831	27068589	NEK8	Gain	No	No
A5	chr17	27594515	27899715	NUFIP2	Gain	No	No
A5	chr17	27942962	28513493	CORO6	Gain	No	No
A5	chr17	29848932	31203902	RAB11FIP4	Gain	No	No
A5	chr17	30357674	30696473	LRRC37B	Gain	No	No
A5	chr17	33434006	33469334	RAD51D,RAD51L3-RFFL	Gain	No	No
A5	chr17	34861135	35414171	MYO19	Gain	Yes	No
A5	chr17	36617148	36896695	ARHGAP23	Gain	No	No
A5	chr17	36958952	37557909	CWC25	Gain	No	No
A5	chr17	37342202	37682569	CACNB1	Gain	No	No
A5	chr17	37441730	37934020	FBXL20	Gain	No	No
A5	chr17	38121935	38134019	GSDMA	Gain	Yes	No
A5	chr17	38182584	38504716	MED24	Gain	No	No
A5	chr17	38498270	39661865	RARA	Gain	No	No
A5	chr17	39190136	40622236	KRTAP1-3	Gain	No	No
A5	chr17	40024961	39992209	ACLY	Gain	No	No
A5	chr17	40441419	40461502	STAT5A	Gain	No	No
A5	chr17	40574644	40729579	PTRF	Gain	No	No
A5	chr17	40734691	40718299	FAM134C	Gain	No	No
A5	chr17	40761357	39978069	FAM134C	Gain	No	No
A5	chr17	40819932	40849968	PLEKHH3	Gain	No	No
A5	chr17	40857105	38804103	EZH1	Gain	Yes	No
A5	chr17	41179199	41353797	RND2	Gain	No	No
A5	chr17	41561333	41623036	DHX8	Gain	No	No

A5	chr17	42260297	42266956	ASB16-AS1	Gain	No	No
A5	chr17	42328526	42340302	SLC4A1	Gain	No	No
A5	chr17	42837089	42855610	ADAM11	Gain	No	No
A5	chr17	43003781	43012784	KIF18B	Gain	No	No
A5	chr17	43191734	43333782	PLCD3	Gain	No	No
A5	chr17	43209614	43221543	PLCD3	Gain	No	No
A5	chr17	45567531	45906036	MRPL45P2	Gain	No	No
A5	chr17	45885903	45899147	OSBPL7	Gain	No	No
A5	chr17	46799679	46806111	PRAC1	Gain	No	No
A5	chr17	48183248	48155648	PDK2	Gain	No	No
A5	chr17	48638428	48704832	CACNA1G-AS1	Gain	No	No
A5	chr17	49197714	48762236	SPAG9	Gain	No	No
A5	chr17	56288019	56343687	MKS1	Gain	No	No
A5	chr17	58136782	58236906	HEATR6	Gain	No	No
A5	chr17	61729916	61623306	MAP3K3	Gain	No	No
A5	chr17	61910428	61974021	SMARCD2	Gain	No	No
A5	chr17	62574609	62504838	SMURF2	Gain	No	No
A5	chr17	71419524	72619897	SDK2	Gain	No	No
A5	chr17	72218623	71394324	TTYH2	Gain	No	No
A5	chr17	72694714	72815982	CD300LF,RAB37	Gain	No	No
A5	chr17	74080086	74099868	EXOC7	Gain	Yes	No
A5	chr17	74141502	74300606	RNF157,RNF157-AS1	Gain	No	No
A5	chr17	74148435	73571290	RNF157,RNF157-AS1	Gain	No	No
A5	chr17	76109628	76135321	TMC6	Gain	No	No
A5	chr17	77073511	77812853	ENGASE	Gain	No	No
A5	chr17	77090532	76800060	RBFOX3	Gain	No	No
A5	chr17	78078353	78022560	GAA	Gain	No	No
A5	chr17	78113468	79091232	EIF4A3	Gain	No	No
A5	chr17	78152306	78180884	CARD14	Gain	No	No
A5	chr17	78187602	78223086	SGSH	Gain	No	No
A5	chr17	78357476	77987408	RNF213,LOC100294362	Gain	No	No
A5	chr17	78445531	79155132	NPTX1	Gain	No	No
A5	chr17	79495416	79639185	FSCN2	Gain	No	No
A5	chr17	79792378	79895115	PPP1R27	Gain	No	No

A5	chr17	80086368	80085784	CCDC57	Gain	No	No
A5	chr18	18533538	20606449	ROCK1	Gain	No	No
A5	chr18	19263470	19411367	ABHD3	Gain	No	No
A5	chr18	20735788	20714634	CABLES1	Gain	No	No
A5	chr18	21644103	21451625	TTC39C	Gain	No	No
A5	chr18	21891947	21977833	OSBPL1A	Gain	No	No
A5	chr18	23612362	23670611	SS18	Gain	No	No
A5	chr18	23806846	23937758	TAF4B	Gain	No	No
A5	chr18	24126691	19102791	KCTD1	Gain	No	No
A5	chr2	9613044	10922501	CPSF3	Gain	No	No
A5	chr2	9633026	9985447	ADAM17	Gain	No	No
A5	chr2	23785021	24480962	KLHL29	Gain	No	No
A5	chr2	24194145	24255816	UBXN2A	Gain	No	No
A5	chr2	25459804	25376630	DNMT3A	Gain	No	No
A5	chr2	25472525	26532973	DNMT3A	Gain	No	No
A5	chr2	26682875	26350820	OTOF	Gain	Yes	No
A5	chr2	27245079	27322708	MAPRE3	Gain	No	No
A5	chr2	27435218	27435475	ATRAID	Gain	Yes	No
A5	chr2	27479253	27530776	SLC30A3	Gain	No	No
A5	chr2	27702892	27745461	IFT172	Gain	Yes	No
A5	chr2	29217590	31348119	FAM179A	Gain	No	No
A5	chr2	30953595	32249325	CAPN13	Gain	No	No
A5	chr2	42806246	42556156	MTA3	Gain	No	No
A5	chr2	45419278	46609737	UNQ6975	Gain	No	No
A5	chr2	49247224	47288110	FSHR	Gain	No	No
A5	chr2	53999043	55449629	ASB3,CHAC2,GPR75-ASB3	Gain	No	No
A5	chr2	55155449	54786024	EML6	Gain	No	No
A5	chr2	62934031	69208112	EHBP1	Gain	No	No
A5	chr2	69581620	69870406	GFPT1	Gain	No	No
A5	chr2	70008651	70406774	ANXA4	Gain	No	No
A5	chr2	71159896	71212629	VAX2	Gain	No	No
A5	chr2	72960199	73777564	EXOC6B	Gain	No	No
A5	chr2	73115442	73467671	SPR	Gain	No	No
A5	chr2	74369398	74601862	BOLA3	Gain	No	No

A5	chr2	74604558	74708665	DCTN1	Gain	No	No
A5	chr2	74710448	74808974	TTC31	Gain	No	No
A5	chr2	74746166	74756409	DQX1	Gain	No	No
A5	chr2	74761008	74758829	LOXL3	Gain	No	No
A5	chr2	84658631	85872206	SUCLG1	Gain	No	No
A5	chr2	85777677	85770157	GGCX	Gain	No	No
A5	chr2	86669140	86718180	KDM3A	Gain	No	No
A5	chr2	95595763	95954754	LOC442028	Gain	No	No
A5	chr2	96261949	96693300	TRIM43	Gain	No	No
A5	chr2	96991484	97779673	ITPRIPL1	Gain	No	No
A5	chr2	97492569	97506251	CNNM3	Gain	No	No
A5	chr2	108478049	109103104	RGPD4	Gain	No	No
A5	chr2	109287236	110959071	LIMS1	Gain	No	No
A5	chr2	112561281	114036498	ANAPC1	Gain	No	No
A5	chr2	113939922	114252826	PSD4	Gain	No	No
A5	chr2	125530375	128707982	CNTNAP5	Gain	Yes	No
A5	chr2	189852806	190445537	COL3A1	Gain	No	No
A5	chr2	189917636	190044605	COL5A2	Gain	Yes	No
A5	chr2	190656515	190334971	PMS1	Gain	No	No
A5	chr20	816710	944763	FAM110A	Gain	No	No
A5	chr20	2637584	2736470	NOP56	Gain	No	No
A5	chr20	2846044	2796476	PTPRA,VPS16	Gain	No	No
A5	chr20	2944917	2845963	PTPRA	Gain	No	No
A5	chr20	3193814	3215540	ITPA	Gain	No	No
A5	chr20	3650192	3654931	ADAM33	Gain	No	No
A5	chr20	3669801	3928926	SIGLEC1	Gain	No	No
A5	chr20	3778268	3739325	CDC25B	Gain	No	No
A5	chr20	10630283	11900484	JAG1	Gain	No	No
A5	chr20	18724743	20634231	DTD1	Gain	No	No
A5	chr20	25433337	25281520	NINL	Gain	Yes	No
A5	chr20	30407335	40944636	MYLK2	Gain	No	No
A5	chr20	30458504	30898984	TTLL9	Gain	No	No
A5	chr20	30659464	31315949	HCK	Gain	Yes	No
A5	chr20	31097761	31367951	C20orf112	Gain	No	No

A5	chr20	31413730	31769223	MAPRE1	Gain	No	No
A5	chr20	31571580	31984890	SUN5	Gain	No	No
A5	chr20	31873838	32162067	BPIFB1	Gain	No	No
A5	chr20	32264910	32260228	E2F1	Gain	No	No
A5	chr20	33565434	8770222	MYH7B	Gain	Yes	No
A5	chr20	34064273	34099803	CEP250	Gain	No	No
A5	chr20	34203808	34110417	SPAG4	Gain	No	No
A5	chr20	34287554	34328809	ROMO1	Gain	No	No
A5	chr20	34763472	35532652	EPB41L1	Gain	No	No
A5	chr20	35422777	35423002	SOGA1	Gain	Yes	No
A5	chr20	36935968	37662960	BPI	Gain	No	No
A5	chr20	39708724	40141634	TOP1	Gain	No	No
A5	chr20	39797662	39802951	PLCG1	Gain	No	No
A5	chr20	43566701	43881792	PABPC1L	Gain	Yes	No
A5	chr20	60884115	60928307	ADRM1	Gain	No	No
A5	chr20	60966304	61461033	CABLES2	Gain	No	No
A5	chr20	61915466	61953463	ARFGAP1	Gain	Yes	No
A5	chr20	62275566	62338090	STMN3	Gain	No	No
A5	chr20	62607044	62599305	SAMD10	Gain	No	No
A5	chr22	19338880	19167751	HIRA	Gain	No	No
A5	chr22	20024282	19998031	TANGO2	Gain	No	No
A5	chr22	20073209	20114880	DGCR8	Gain	No	No
A5	chr22	21133584	21335649	SERPIND1,PI4KA	Gain	No	No
A5	chr22	21335035	21351255	AIFM3	Gain	No	No
A5	chr22	22664100	23656901	BMS1P20	Gain	No	No
A5	chr22	24038800	24226616	GUSBP11,RGL4	Gain	No	No
A5	chr22	24121373	19918621	MMP11	Gain	No	No
A5	chr22	24437586	24574596	CABIN1	Gain	No	No
A5	chr22	25202407	25313721	SGSM1	Gain	No	No
A5	chr22	30403037	30782748	MTMR3	Gain	Yes	No
A5	chr22	31368033	31496939	TUG1	Gain	No	No
A5	chr22	31981053	32014537	SFI1	Gain	No	No
A5	chr22	35478510	35743987	ISX	Gain	No	No
A5	chr22	36962399	37499693	CACNG2	Gain	No	No

A5	chr22	37168446	37336479	IFT27	Gain	No	No
A5	chr22	38010196	38028522	GGA1	Gain	No	No
A5	chr22	38308357	38370205	MICALL1	Gain	No	No
A5	chr22	39353526	39448358	APOBEC3A	Gain	Yes	No
A5	chr22	42456927	42478069	NAGA	Gain	No	No
A5	chr22	43820913	44235905	MPPED1	Gain	No	No
A5	chr22	46759892	48972383	CELSR1	Gain	No	No
A5	chr22	50599392	51160865	MOV10L1	Gain	No	No
A5	chr22	50599796	50882546	MOV10L1	Gain	No	No
A5	chr22	50613860	50757432	PANX2	Gain	No	No
A5	chr22	50696671	50687180	MAPK12	Gain	No	No
A5	chr22	50959393	50946135	NCAPH2	Gain	No	No
A5	chr3	167727653	167813417	GOLIM4	Gain	No	No
A5	chr3	168269641	169513399	EGFEM1P	Gain	No	No
A5	chr3	168511802	171574497	EGFEM1P	Gain	No	No
A5	chr3	168806787	168864093	MECOM	Gain	Yes	No
A5	chr3	168862784	180337758	MECOM	Gain	Yes	No
A5	chr3	169098974	169899537	MECOM	Gain	Yes	No
A5	chr3	169637245	169706147	SAMD7	Gain	Yes	No
A5	chr3	170783975	172098883	TNIK	Gain	No	No
A5	chr3	172468474	172241297	ECT2	Gain	No	No
A5	chr3	173116237	177534784	NLGN1	Gain	No	No
A5	chr3	174577110	175473206	NAALADL2	Gain	No	No
A5	chr3	178525130	189713231	KCNMB2	Gain	No	No
A5	chr3	179046070	178785597	ZNF639	Gain	No	No
A5	chr3	179122977	179306193	GNB4	Gain	No	No
A5	chr3	182538047	184026842	ATP11B	Gain	No	No
A5	chr3	183171799	183824783	LINC00888	Gain	No	No
A5	chr3	183432931	183526958	YEATS2	Gain	No	No
A5	chr3	183819272	183911795	HTR3E	Gain	No	No
A5	chr3	184033276	184107617	EIF4G1	Gain	No	No
A5	chr3	184059511	184079439	FAM131A	Gain	No	No
A5	chr3	184529930	184770402	VPS8	Gain	Yes	No
A5	chr3	184870415	184971886	C3orf70	Gain	No	No

A5	chr3	185304030	185344181	SENP2	Gain	No	No
A5	chr3	186358148	186462199	FETUB	Gain	No	No
A5	chr3	186459310	186947685	KNG1	Gain	No	No
A5	chr3	187930720	188590551	LPP	Gain	No	No
A5	chr3	188889762	190580465	TPRG1	Gain	No	No
A5	chr3	188956474	191179245	TPRG1	Gain	No	No
A5	chr3	189349215	189507631	TP63	Gain	No	No
A5	chr3	193151633	195943621	ATP13A4	Gain	No	No
A5	chr3	194150800	194349238	ATP13A3	Gain	Yes	No
A5	chr3	195390494	195538844	SDHAP2	Gain	Yes	No
A5	chr3	195590235	195609266	TNK2	Gain	No	No
A5	chr3	195622116	195803993	TNK2	Gain	No	No
A5	chr3	196612021	196876667	SENP5	Gain	No	No
A5	chr3	196888510	196754781	DLG1	Gain	No	No
A5	chr3	197346610	197476570	LOC220729	Gain	No	No
A5	chr5	143161	254621	PLEKHG4B	Gain	No	No
A5	chr5	473333	433062	SLC9A3	Gain	Yes	No
A5	chr5	892623	1038927	BRD9	Gain	No	No
A5	chr5	892968	2753469	TRIP13	Gain	No	No
A5	chr5	5057674	6632869	LINC01020	Gain	No	No
A5	chr5	6583715	6746540	LINC01018	Gain	No	No
A5	chr5	7301437	7897360	LOC442132	Gain	No	No
A5	chr5	7859271	10382055	MTRR	Gain	No	No
A5	chr5	9641983	16617167	LOC285692	Gain	No	No
A5	chr5	16668385	33751653	MYO10	Gain	No	No
A5	chr5	17384951	23528706	LOC401177	Gain	No	No
A5	chr5	19473154	19839351	CDH18	Gain	Yes	No
A5	chr5	21750972	22078971	CDH12	Gain	No	No
A5	chr5	31267452	32276848	CDH6	Gain	No	No
A5	chr5	31405783	31529212	DROSHA	Gain	No	No
A5	chr5	35991248	36226665	UGT3A1	Gain	No	No
A5	chr5	36249103	36302011	RANBP3L	Gain	No	No
A5	chr5	38490291	38463113	LIFR	Gain	No	No
A5	chr6	43466715	43470355	TJAP1	Gain	No	No

A5	chr6	43492208	43543812	POLR1C,XPO5	Gain	No	No
A5	chr6	44268892	44281063	AARS2	Gain	Yes	No
A5	chr6	44310832	45165507	SPATS1	Gain	No	No
A5	chr7	2400344	2420377	EIF3B	Gain	No	No
A5	chr7	2773084	2998265	GNA12	Gain	No	No
A5	chr7	22349587	23808793	RAPGEF5	Gain	No	No
A5	chr7	30791750	30922608	INMT	Gain	No	No
A5	chr7	44146144	44047412	AEBP1	Gain	No	No
A5	chr7	44259658	44524883	CAMK2B	Gain	No	No
A5	chr7	44555401	44580914	NPC1L1	Gain	No	No
A5	chr7	44620691	44806262	TMED4	Gain	No	No
A5	chr7	45026123	45144081	SNHG15	Gain	No	No
A5	chr7	73814699	74016920	CLIP2	Gain	No	No
A5	chr7	75141912	75601779	PMS2P3	Gain	No	No
A5	chr7	95625259	98582667	DYNC1I1	Gain	No	No
A5	chr7	99070514	98991742	ZNF789	Gain	No	No
A5	chr7	99526462	99673253	GJC3	Gain	No	No
A5	chr7	99700297	99662663	AP4M1	Gain	No	No
A5	chr7	99704692	99717481	AP4M1	Gain	No	No
A5	chr7	99754227	99819111	C7orf43	Gain	No	No
A5	chr7	99938567	100029312	STAG3L5P,STAG3L5P-PVRIG2P-PILRB	Gain	No	No
A5	chr7	100061585	100246446	C7orf61	Gain	No	No
A5	chr7	100201460	100180082	PCOLCE,PCOLCE-AS1	Gain	No	No
A5	chr7	100238308	100197425	TFR2	Gain	No	No
A5	chr7	100370907	100320466	ZAN	Gain	No	No
A5	chr7	100877558	100861011	CLDN15	Gain	No	No
A5	chr7	128517537	128529093	KCP	Gain	No	No
A5	chr7	129855813	129940754	SSMEM1	Gain	No	No
A5	chr7	131817807	132193538	PLXNA4	Gain	No	No
A5	chr7	142560884	142482399	EPHB6	Gain	No	No
A5	chr7	143079685	143104771	ZYX	Gain	No	No
A5	chr7	149486252	149523027	SSPO	Gain	No	No
A5	chr7	150646515	150712801	KCNH2	Gain	No	No
A5	chr7	150761288	150749843	SLC4A2	Gain	No	No

A5	chr7	150873198	150972337	ASB10	Gain	No	No
A5	chr8	32298261	33370230	NRG1	Gain	No	No
A5	chr8	32405727	32622558	NRG1	Gain	No	No
A5	chr8	33246480	38315052	FUT10	Gain	No	No
A5	chr8	37727937	48196683	RAB11FIP1	Gain	No	No
A5	chr8	38250130	38264981	LETM2	Gain	No	No
A5	chr8	38644721	38704337	TACC1	Gain	No	No
A5	chr8	39356852	39587583	ADAM3A	Gain	No	No
A5	chr8	39602348	39695808	ADAM2	Gain	No	No
A5	chr8	40438683	41906820	ZMAT4	Gain	No	No
A5	chr8	41517958	42025328	ANK1	Gain	No	No
A5	chr8	48689404	48888408	PRKDC	Gain	Yes	No
A5	chr8	53028834	53627026	ST18	Gain	No	No
A5	chr8	53450990	51664671	FAM150A	Gain	No	No
A5	chr8	54963571	55014577	LYPLA1	Gain	No	No
A5	chr8	56985367	57123859	RPS20	Gain	No	No
A5	chr8	57218155	59412720	SDR16C5	Gain	No	No
A5	chr8	58196996	59571966	LINC00588	Gain	No	No
A5	chr8	58988093	62627199	FAM110B	Gain	No	No
A5	chr8	59720676	74440039	TOX	Gain	No	No
A5	chr8	61121308	61880307	CA8	Gain	No	No
A5	chr8	62602231	64100303	ASPH	Gain	No	No
A5	chr8	63927638	65291391	GGH	Gain	No	No
A5	chr8	66701116	67067945	PDE7A	Gain	No	No
A5	chr8	67344717	67478478	ADHFE1	Gain	No	No
A5	chr8	67705850	67592259	SGK3,C8orf44-SGK3	Gain	No	No
A5	chr8	67755686	68105837	SGK3,C8orf44-SGK3	Gain	No	No
A5	chr8	67955314	71619388	COPS5	Gain	No	No
A5	chr8	68864602	68658620	PREX2	Gain	No	No
A5	chr8	69020343	69728180	PREX2	Gain	No	No
A5	chr8	70476150	70408161	SULF1	Gain	Yes	No
A5	chr8	70584567	70747299	SLCO5A1	Gain	Yes	No
A5	chr8	71595747	74872053	XKR9	Gain	No	No
A5	chr8	72123390	72268746	EYA1	Gain	No	No

A5	chr8	72877643	73951454	LOC100132891	Gain	No	No
A5	chr8	75149442	75275288	JPH1	Gain	No	No
A5	chr8	75617927	75941752	FLJ39080	Gain	No	No
A5	chr8	77913118	80680098	PEX2	Gain	No	No
A5	chr8	79648708	77768536	IL7	Gain	No	No
A5	chr8	82192717	82633539	LOC101927085	Gain	No	No
A5	chr8	85258353	86089889	RALYL	Gain	No	No
A5	chr8	86354036	93029591	CA3	Gain	No	No
A5	chr8	92261515	92406267	SLC26A7	Gain	Yes	No
A5	chr8	95142853	95229531	CDH17	Gain	No	No
A5	chr8	95220710	95709189	CDH17	Gain	No	No
A5	chr8	96037213	97243743	NDUFAF6	Gain	No	No
A5	chr8	97172514	97343323	GDF6	Gain	No	No
A5	chr8	97252021	99306621	MTERFD1	Gain	No	No
A5	chr8	99045269	98735263	MATN2	Gain	Yes	No
A5	chr8	99538969	96281118	STK3	Gain	Yes	No
A5	chr8	99954605	102731876	STK3	Gain	Yes	No
A5	chr8	100025493	100883925	VPS13B	Gain	No	No
A5	chr8	100548863	101158099	VPS13B	Gain	No	No
A5	chr8	100974621	101117630	RGS22	Gain	No	No
A5	chr8	101174506	101252999	SPAG1	Gain	No	No
A5	chr8	101589246	101961128	SNX31	Gain	No	No
A5	chr8	104153481	104417089	BAALC,C8orf56	Gain	No	No
A5	chr8	104312183	104078675	FZD6	Gain	No	No
A5	chr8	104387965	110346350	CTHRC1	Gain	No	No
A5	chr8	105544134	107726213	LRP12	Gain	No	No
A5	chr8	110654956	110539249	SYBU	Gain	No	No
A5	chr8	116981908	118183407	LINC00536	Gain	No	No
A5	chr8	118532951	124031545	MED30	Gain	No	No
A5	chr8	119634177	133493004	SAMD12,SAMD12-AS1	Gain	No	No
A5	chr8	120605991	119634184	ENPP2	Gain	Yes	No
A5	chr8	124146353	124408705	TBC1D31	Gain	No	No
A5	chr8	124747994	125500859	ANXA13	Gain	No	No
A5	chr8	124968231	126096264	FER1L6	Gain	No	No

A5	chr8	124996377	125740748	FER1L6	Gain	No	No
A5	chr8	128427856	133590763	POU5F1B	Gain	No	No
A5	chr8	131070199	128903244	ASAP1	Gain	No	No
A5	chr8	131792546	133811418	ADCY8	Gain	Yes	No
A5	chr8	133726240	133740232	TMEM71	Gain	No	No
A5	chr8	134072344	134296572	SLA,TG	Gain	No	No
A5	chr8	136533479	141461482	KHDRBS3	Gain	No	No
A5	chr8	141468379	141685598	TRAPPC9	Gain	No	No
A5	chr8	142366586	143783131	GPR20	Gain	No	No
A5	chr8	144124374	144079080	C8orf31	Gain	No	No
A5	chr8	144241614	144344875	LY6H	Gain	No	No
A5	chr8	144416909	144672251	TOP1MT	Gain	No	No
A5	chr8	144641501	144645231	GSDMD	Gain	No	No
A5	chr8	144648362	144654928	MROH6	Gain	No	No
A5	chr8	144674816	144691784	EEF1D	Gain	Yes	No
A5	chr8	144917989	145067583	NRBP2	Gain	No	No
A5	chr8	145018815	145060635	PLEC	Gain	No	No
A5	chr8	145160542	145141119	MAF1	Gain	No	No
A5	chr8	145512775	145268368	BOP1	Gain	No	No
A5	chr8	145699114	146005665	KIFC2	Gain	No	No
A5	chr8	146028254	146278829	ZNF517	Gain	No	No
A5	chr9	16870643	33568891	BNC2	Gain	No	No
A5	chr9	32552231	33001639	TOPORS,TOPORS-AS1	Gain	No	No
A5	chr9	33048103	33278223	SMU1	Gain	No	No
A5	chr9	33366626	34017187	NFX1	Gain	No	No
A5	chr9	33934051	34381598	UBAP2	Gain	Yes	No
A5	chr9	34568009	34637109	CNTFR	Gain	No	No
A5	chr9	34616026	34614195	DCTN3	Gain	No	No
A5	chr9	35057372	35108734	VCP	Gain	No	No
A5	chr9	35478238	35539105	ATP8B5P	Gain	No	No
A5	chr9	35749276	35737005	GBA2	Gain	No	No
A5	chr9	36581640	36677680	MELK	Gain	No	No
A5	chr9	37438099	37762682	ZBTB5	Gain	No	No
A5	chr9	37887593	39355916	SLC25A51	Gain	No	No

A5	chr9	39456879	39178357	LOC653501	Gain	No	No
A5	chr9	39887005	41325311	SPATA31A2	Gain	No	No
A5	chr9	43133229	43894319	ANKRD20A3	Gain	No	No
A5	chr9	69112839	69653179	PGM5P2	Gain	No	No
A5	chr9	73149965	73461507	TRPM3	Gain	Yes	No
A5	chr9	86451614	86530593	KIF27	Gain	No	No
A5	chr9	86570295	86595184	C9orf64	Gain	No	No
A5	chr9	87285291	87482346	NTRK2	Gain	No	No
A5	chr9	88430793	88694256	LOC389765	Gain	No	No
A5	chr9	88714939	88968145	GOLM1	Gain	No	No
A5	chr9	88842769	88637217	C9orf153	Gain	No	No
A5	chr9	90113884	86153348	DAPK1	Gain	No	No
A5	chr9	90534169	90584834	SPATA31C1	Gain	No	No
A5	chr9	91652907	92033352	SHC3	Gain	No	No
A5	chr9	94972489	95148578	IARS	Gain	No	No
A5	chr9	95738489	95796963	FGD3	Gain	No	No
A5	chr9	95993169	96081655	WNK2	Gain	No	No
A5	chr9	96082648	96827103	WNK2,C9orf129	Gain	No	No
A5	chr9	96097655	96324586	C9orf129	Gain	No	No
A5	chr9	97521930	95432547	C9orf3	Gain	No	No
A5	chr9	97572243	98279247	C9orf3	Gain	No	No
A5	chr9	97822975	102861330	C9orf3	Gain	No	No
A5	chr9	97869347	98011651	FANCC	Gain	No	No
A5	chr9	98740342	100053769	LOC101928170	Gain	No	No
A5	chr9	98997588	99160596	HSD17B3	Gain	No	No
A5	chr9	99271954	99250556	CDC14B	Gain	No	No
A5	chr9	99403532	99631344	AAED1	Gain	No	No
A5	chr9	99800199	100991499	CTSV	Gain	No	No
A5	chr9	100054837	100919942	LOC100499484-C9ORF174,LOC100499484	Gain	No	No
A5	chr9	100286422	99735230	TMOD1	Gain	No	No
A5	chr9	101507840	101910066	ANKS6	Gain	No	No
A5	chr9	102137419	102609897	NAMA	Gain	No	No
A5	chr9	102677459	103111654	STX17	Gain	No	No
A5	chr9	130536268	130493879	SH2D3C	Gain	No	No

A5	chr9	131038865	131012635	SWI5	Gain	No	No
A5	chrX	46521462	47074527	SLC9A7	Gain	No	No
A5	chrX	47082950	47088043	CDK16	Gain	No	No
A5	chrX	48830130	48858675	GRIPAP1	Gain	No	No
A5	chrX	49076931	49056661	CACNA1F	Gain	No	No
A5	chrX	49103191	49040370	CCDC22	Gain	No	No
A5	chrX	50131524	50213737	DGKK	Gain	Yes	No
A5	chrX	53264964	53659516	IQSEC2	Gain	No	No
A5	chrX	54777435	54824673	ITIH6	Gain	No	No
A5	chrX	70341176	70361220	MED12	Gain	No	No
A5	chrX	70443541	70468651	GJB1	Gain	No	No
A5	chrX	100490848	100515158	DRP2	Gain	Yes	No
A5	chrX	107447549	107812051	COL4A6	Gain	No	No
A5	chrX	107819139	107863656	COL4A5	Gain	No	No
A5	chrX	118603623	118716639	SLC25A5	Gain	No	No
A5	chrX	150840654	152960344	PASD1	Gain	No	No
A5	chrX	152801579	152835214	ATP2B3	Gain	No	No
A5	chrX	153060128	153060040	SSR4	Gain	No	No
A5	chrX	153128822	153174623	L1CAM	Gain	No	No
A5	chrX	153590037	153524346	FLNA	Gain	No	No
A5	chrX	153663484	153699981	ATP6AP1	Gain	No	No
A5	chr11	129312729	129830977	BARX2	Loss	No	No
A5	chr11	129734860	129762904	NFRKB	Loss	No	No
A5	chr11	129979323	130011969	APLP2	Loss	No	No
A5	chr11	130078304	130786382	ST14	Loss	No	No
A5	chr11	130274817	130543083	ADAMTS8	Loss	No	No
A5	chr11	132812820	134018713	OPCML	Loss	Yes	No
A5	chr11	134123433	134122810	ACAD8	Loss	No	No
A5	chr11	134147219	134257588	GLB1L3	Loss	Yes	No
A5	chr11	134212647	134244612	GLB1L2	Loss	No	No
A5	chr13	38156502	38357497	POSTN	Loss	No	No
A5	chr13	60348322	60686320	DIAPH3	Loss	No	No
A5	chr13	96540145	96675406	UGGT2	Loss	No	No
A5	chr16	16330757	16367932	NOMO3	Loss	No	No

A5	chr18	13752324	14852737	RNMT	Loss	No	No
A5	chr18	50432413	51013328	DCC	Loss	No	No
A5	chr18	59770026	59828618	PIGN	Loss	No	No
A5	chr2	141643706	142012210	LRP1B	Loss	Yes	Yes
A5	chr21	14414822	16340847	ANKRD30BP2	Loss	No	No
A5	chr21	14995125	15755509	POTED	Loss	No	No
A5	chr21	22652897	22906977	NCAM2	Loss	Yes	Yes
A5	chr3	72842066	73657835	SHQ1	Loss	No	No
A5	chr3	73110809	75263699	PPP4R2	Loss	No	No
A5	chr3	74315631	71050210	CNTN3	Loss	No	No
A5	chr3	81548260	77694055	GBE1	Loss	No	No
A5	chr3	85851196	85775698	CADM2,CADM2-AS2	Loss	Yes	No
A5	chr5	70307101	70358623	NAIP	Loss	No	No
A5	chr5	177156023	177171968	FAM153A	Loss	No	No
A5	chr8	363072	417746	FBXO25	Loss	No	No
A5	chr8	614199	1645465	ERICH1	Loss	No	No
A5	chr8	11216722	11302317	TDH	Loss	No	No
A5	chr8	11606427	11710988	GATA4	Loss	No	No
A5	chr8	21900655	22108680	FGF17	Loss	No	No
A5	chr8	21965643	21960139	NUDT18	Loss	No	No
A5	chr8	22881700	22886115	TNFRSF10B	Loss	No	No
A5	chr8	23103768	23307510	CHMP7	Loss	No	No
A5	chr8	23423620	24193612	SLC25A37	Loss	No	No
A5	chr8	27255064	27312128	PTK2B	Loss	No	No
A5	chr8	27358444	27401761	EPHX2	Loss	No	No
A5	chr8	27468662	28019595	CLU	Loss	No	No
A5	chr8	27516012	28595180	SCARA3	Loss	No	No
A5	chr8	27884478	27507317	NUGGC	Loss	No	No
A5	chr8	28206641	28413488	ZNF395	Loss	No	No
A5	chr8	28331288	26223922	FBXO16	Loss	No	No
A5	chr8	28950261	29102956	KIF13B	Loss	No	No
A5	chr8	30040590	30569605	DCTN6	Loss	No	No
A5	chr8	30648712	30890317	PPP2CB	Loss	No	No
A5	chrY	21751968	23563448	TXLNG2P	Loss	No	No

A6	chr12	25398207	25398329	KRAS	Gain	Yes	No
A6	chr8	144874045	144874083	SCRIB	Gain	No	No
A6	chr9	129456024	129456091	LMX1B	Gain	No	No
A6	chr13	24863136	25052414	SPATA13	Loss	No	No
A6	chr13	26043114	26586760	ATP8A2	Loss	No	No
A6	chr13	29041039	28931822	FLT1	Loss	No	No
A6	chr13	37399564	37582972	RFXAP	Loss	No	No
A6	chr13	42701598	42793930	DGKH	Loss	No	No
A6	chr13	46050335	46093229	COG3	Loss	No	No
A6	chr13	51530493	52960321	RNASEH2B	Loss	No	No
A6	chr13	77754261	77901177	MYCBP2	Loss	No	No
A6	chr13	96212385	95900007	CLDN10	Loss	No	No
A6	chr13	98018712	98829521	MBNL2	Loss	No	No
A6	chr13	99448459	99630338	DOCK9	Loss	No	No
A6	chr13	103257142	103309557	TPP2	Loss	No	No
A6	chr13	110807629	111563117	COL4A1	Loss	No	No
A6	chr13	111857635	111953879	ARHGEF7	Loss	No	No
A6	chr13	113030650	113242499	SPACA7	Loss	No	No
A6	chr13	113439448	114201694	ATP11A	Loss	No	No
A6	chr13	113792770	113824844	F10	Loss	No	No
A6	chr13	113873258	113699675	CUL4A	Loss	No	No
A6	chr13	114292132	111160568	TFDP1	Loss	No	No
A6	chr13	114751085	114839312	RASA3	Loss	No	No
A6	chr13	115002119	115030715	CDC16	Loss	No	No
A6	chr19	1045000	1065570	ABCA7	Loss	No	No
A6	chr19	10248507	10292753	DNMT1	Loss	No	No
A6	chr19	11348720	11363634	DOCK6	Loss	No	No
A6	chr19	11489366	11527733	EPOR	Loss	No	No
A6	chr19	17612081	17786850	SLC27A1	Loss	No	No
A6	chr19	33090609	33134105	ANKRD27	Loss	No	No
A6	chr19	36047814	36587981	ATP4A	Loss	No	No
A6	chr19	39907489	39955689	PLEKHG2	Loss	No	No
A6	chr2	202512413	202672366	MPP4	Loss	No	No
A6	chr2	206562267	203421254	NRP2	Loss	Yes	No

A6	chr3	8775485	9146526	SSUH2	Loss	No	No
A6	chr3	10379859	11340329	ATP2B2	Loss	No	No
A6	chr3	13383495	12983365	NUP210	Loss	No	No
A6	chr3	13975635	14214562	FGD5P1	Loss	Yes	No
A6	chr3	16242125	17550097	GALNT15	Loss	No	No
A6	chr3	19190016	23847579	KCNH8	Loss	No	No
A6	chr3	30032572	27394410	RBMS3	Loss	No	No
A6	chr3	31617887	32815367	STT3B	Loss	No	No
A6	chr3	33255419	33481897	SUSD5	Loss	No	No
A6	chr3	35724339	35835988	ARPP21	Loss	No	No
A6	chr3	37356910	36986548	GOLGA4	Loss	Yes	No
A6	chr3	38674525	38164228	SCN5A	Loss	Yes	No
A6	chr3	39093506	39145044	WDR48	Loss	No	No
A6	chr3	41290979	33614846	ULK4	Loss	Yes	No
A6	chr3	42705288	42835791	ZBTB47	Loss	No	No
A6	chr3	46026347	45565600	FYCO1	Loss	No	No
A6	chr3	48964894	48362564	ARIH2	Loss	No	No
A6	chr3	49215914	49363278	C3orf84	Loss	No	No
A6	chr3	50402786	50513630	CACNA2D2	Loss	No	No
A6	chr3	53212419	52713739	PRKCD	Loss	No	No
A6	chr3	54157539	60522712	CACNA2D3	Loss	No	No
A6	chr3	58484091	62306199	KCTD6	Loss	No	No
A6	chr3	61734551	62278231	PTPRG	Loss	Yes	No
A6	chr3	62388755	62751659	CADPS	Loss	Yes	No
A6	chr3	64507841	64641551	ADAMTS9	Loss	No	No
A6	chr3	66431902	66287124	LRIG1	Loss	Yes	No
A6	chr3	69105001	69129524	UBA3	Loss	No	No
A6	chr4	3317796	3433904	RGS12	Loss	No	No
A6	chr4	5617148	6083482	EVC2	Loss	No	No
A6	chr4	6288819	7736101	WFS1	Loss	No	No
A6	chr4	7774546	7873807	AFAP1,AFAP1-AS1	Loss	No	No
A6	chr4	10078927	9922196	WDR1	Loss	No	No
A6	chr4	13578461	13621691	BOD1L1	Loss	No	No
A6	chr4	15477538	16077741	CC2D2A	Loss	No	No

A6	chr4	20255186	20619273	SLIT2	Loss	Yes	No
A6	chr4	24544562	25030200	DHX15	Loss	No	No
A6	chr4	24896613	25831778	CCDC149	Loss	No	No
A6	chr4	25158477	25366788	SEPSECS	Loss	No	No
A6	chr4	26322321	27019606	RBPJ	Loss	No	No
A6	chr4	36075310	36286213	ARAP2	Loss	No	No
A6	chr4	41614788	41608021	LIMCH1	Loss	No	No
A6	chr4	42037291	42629126	SLC30A9	Loss	No	No
A6	chr6	491124	633117	EXOC2	Loss	No	No
A6	chr6	1624706	5216971	GMDS	Loss	No	No
A6	chr6	7845372	8064647	BMP6	Loss	No	No
A6	chr8	1998868	6420784	MYOM2	Loss	Yes	No
A6	chr8	3263548	4852328	CSMD1	Loss	Yes	No
A6	chr8	13959884	22021059	SGCZ	Loss	No	No
A6	chr8	19675785	19805851	INTS10	Loss	No	No
A6	chr8	25154028	25199325	DOCK5	Loss	No	No
A6	chr8	25209246	25267718	DOCK5	Loss	No	No
A6	chr8	32617717	27312128	NRG1	Loss	No	No
A6	chr8	35425615	37730697	UNC5D	Loss	No	No
A6	chr8	38873636	39142436	ADAM9	Loss	No	No
A6	chr8	39603990	39695808	ADAM2	Loss	No	No
A6	chr9	19057958	19096767	HAUS6	Loss	No	No
A7	chr1	153965490	153800824	NUP210L	Gain	No	No
A7	chr1	155733102	155716726	GON4L	Gain	No	No
A7	chr16	3454908	3659567	ZNF174	Gain	No	No
A7	chr16	3708037	3736137	DNASE1	Gain	No	No
A7	chr16	4606490	4797026	C16orf96	Gain	No	No
A7	chr16	4721318	4897303	MGRN1	Gain	No	No
A7	chr16	5083702	5133758	NAGPA	Gain	No	No
A7	chr16	5094122	4733933	C16orf89	Gain	No	No
A7	chr16	9010882	9892321	USP7	Gain	No	No
A7	chr16	10721360	11016347	TEKT5	Gain	No	No
A7	chr16	11647388	11830089	LITAF	Gain	No	No
A7	chr16	11846524	11991892	ZC3H7A	Gain	No	No

A7	chr16	11933551	12061925	RSL1D1	Gain	No	No
A7	chr16	12093153	12371893	SNX29	Gain	No	No
A7	chr16	16101672	16205439	ABCC1	Gain	No	No
A7	chr16	28109297	28223239	XPO6	Gain	No	No
A7	chr16	28549326	28503156	NUPR1	Gain	No	No
A7	chr16	28847646	28603111	ATXN2L	Gain	No	No
A7	chr16	28877332	28857729	SH2B1	Gain	No	No
A7	chr16	28893771	28855328	ATP2A1	Gain	No	No
A7	chr16	28915741	28935936	ATP2A1	Gain	No	No
A7	chr16	28986421	29002104	SPNS1	Gain	No	No
A7	chr18	14830164	20833900	ANKRD30B	Gain	No	No
A7	chr18	21452983	21891470	LAMA3	Gain	No	No
A7	chr18	21901649	23670611	OSBPL1A	Gain	No	No
A7	chr18	22669428	23759094	ZNF521	Gain	No	No
A7	chr18	24441094	28673606	AQP4	Gain	No	No
A7	chr18	24512956	28622781	CHST9,AQP4-AS1	Gain	No	No
A7	chr18	28710755	28739492	DSC1	Gain	No	No
A7	chr18	29412046	29645972	TRAPPC8	Gain	No	No
A7	chr20	30409241	35646772	MYLK2	Gain	No	No
A7	chr20	33545900	33589329	MYH7B	Gain	Yes	No
A7	chr20	33578178	33735161	MYH7B	Gain	Yes	No
A7	chr20	33894468	34099803	UQCC1	Gain	No	No
A7	chr20	33969720	35399876	UQCC1	Gain	No	No
A7	chr20	34110836	34330258	C20orf173	Gain	No	No
A7	chr20	34142142	34117481	ERGIC3	Gain	No	No
A7	chr20	47837988	47860614	DDX27	Gain	No	No
A7	chr3	169953017	170016898	PRKCI	Gain	No	No
A7	chr3	183728238	183824783	ABCC5,ABCC5-AS1	Gain	No	No
A7	chr3	185307901	185344181	SENP2	Gain	No	No
A7	chr3	195053756	196159345	ACAP2	Gain	No	No
A7	chr3	195780288	195803993	TFRC	Gain	No	No
A7	chr3	195925146	196043102	ZDHHC19	Gain	No	No
A7	chr3	196509496	196554204	PAK2	Gain	No	No
A7	chr5	38944547	39203089	RICTOR	Gain	No	No

A7	chr7	2739867	2644625	AMZ1		Gain	No	No
A7	chr7	5402280	6591067	TNRC18		Gain	No	No
A7	chr7	5769062	6045657	RNF216		Gain	Yes	No
A7	chr7	5941287	6098860	CCZ1		Gain	Yes	No
A7	chr7	44663915	44747335	OGDH		Gain	No	No
A7	chr7	55872950	56008433		14-Sep	Gain	No	No
A7	chr7	73815834	74016920	CLIP2		Gain	No	No
A7	chr7	100344160	100395419	ZAN		Gain	No	No
A7	chr1	215812496	216595882	USH2A		Loss	Yes	No
A7	chr10	15824153	15902519	FAM188A		Loss	No	No
A7	chr10	45465612	45984865	RASSF4		Loss	No	No
A7	chr10	52103251	52619745	SGMS1		Loss	No	No
A7	chr10	52834233	56424050	PRKG1		Loss	No	No
A7	chr10	61802429	62149634	ANK3		Loss	No	No
A7	chr10	63173216	63856707	TMEM26		Loss	No	No
A7	chr10	63440900	64383010	C10orf107		Loss	No	No
A7	chr10	71582124	71716798	COL13A1		Loss	No	No
A7	chr10	72576566	72518041	SGPL1		Loss	No	No
A7	chr10	80968092	81070941	ZMIZ1		Loss	No	No
A7	chr10	85901217	85973104	GHITM		Loss	No	No
A7	chr10	87373163	89118188	GRID1		Loss	No	No
A7	chr10	90350341	90773124	LIPJ		Loss	No	No
A7	chr10	93185037	92509351	HECTD2,LOC100188947		Loss	No	No
A7	chr10	93237957	93261091	HECTD2,LOC100188947		Loss	No	No
A7	chr10	93579695	93621912	TNKS2		Loss	No	No
A7	chr10	94679738	95137186	EXOC6		Loss	No	No
A7	chr10	95430526	95721672	FRA10AC1		Loss	No	No
A7	chr10	96094390	96827114	NOC3L		Loss	No	No
A7	chr10	96356616	96084861	HELLS		Loss	No	No
A7	chr10	96698414	97402963	CYP2C9		Loss	No	No
A7	chr10	97078076	97200937	SORBS1		Loss	No	No
A7	chr10	98762460	98945683	SLIT1		Loss	No	No
A7	chr10	99619214	105992120	GOLGA7B		Loss	No	No
A7	chr10	100249807	100221576	HPSE2		Loss	No	No

A7	chr10	101606717	101985774	ABCC2	Loss	No	No
A7	chr10	101668709	104859776	DNMBP	Loss	No	No
A7	chr10	105330628	106015029	NEURL	Loss	No	No
A7	chr10	105648829	106039229	OBFC1	Loss	No	No
A7	chr10	106118098	106214848	CCDC147	Loss	No	No
A7	chr10	108339126	108924466	SORCS1	Loss	No	No
A7	chr10	112350748	114186617	SMC3	Loss	No	No
A7	chr10	113914386	113941571	GPAM	Loss	No	No
A7	chr10	114059369	115423829	TECTB	Loss	Yes	No
A7	chr10	114220282	115312949	VTI1A	Loss	No	No
A7	chr10	116731893	118427834	TRUB1	Loss	No	No
A7	chr10	119774575	121212788	RAB11FIP2	Loss	No	No
A7	chr10	121558054	123353481	INPP5F	Loss	No	No
A7	chr10	122612035	124152857	WDR11	Loss	Yes	No
A7	chr10	124376743	124403252	DMBT1	Loss	No	No
A7	chr10	127409772	127442414	C10orf137	Loss	No	No
A7	chr10	127668729	127529899	FANK1	Loss	No	No
A7	chr10	128795011	127727998	DOCK1	Loss	No	No
A7	chr10	129839138	134671292	PTPRE	Loss	No	No
A7	chr10	134735607	95178	TTC40	Loss	No	No
A7	chr10	135340866	135367832	CYP2E1	Loss	No	No
A7	chr11	1248904	1283406	MUC5B	Loss	Yes	No
A7	chr11	4159424	5264822	RRM1	Loss	No	No
A7	chr11	5757677	6232365	OR52N4	Loss	No	No
A7	chr11	6541467	6593254	DNHD1	Loss	No	No
A7	chr11	6632047	6640692	ILK	Loss	No	No
A7	chr11	7023939	7694122	ZNF214	Loss	No	No
A7	chr11	7570670	8248647	PPFIBP2	Loss	No	No
A7	chr11	9228219	8673450	DENND5A	Loss	No	No
A7	chr11	10602001	10655618	MRVI1,MRVI1-AS1	Loss	No	No
A7	chr11	11906013	12348813	USP47	Loss	Yes	No
A7	chr11	13375847	13408812	ARNTL	Loss	Yes	No
A7	chr11	14856527	21594955	PDE3B	Loss	No	No
A7	chr11	16007749	16362798	SOX6	Loss	No	No

A7	chr11	22214721	22296293	ANO5	Loss	No	No
A7	chr11	26694948	27434407	SLC5A12	Loss	No	No
A7	chr11	27520905	30974115	LIN7C	Loss	No	No
A7	chr11	30253443	31828010	FSHB	Loss	No	No
A7	chr11	34937676	35547579	APIP	Loss	No	No
A7	chr11	92498044	3115065	FAT3	Loss	Yes	No
A7	chr11	101324380	100996889	TRPC6	Loss	No	No
A7	chr11	102272269	102714342	TMEM123	Loss	No	No
A7	chr11	102816374	104839325	MMP13	Loss	No	No
A7	chr11	104866466	104916051	CASP5	Loss	No	No
A7	chr11	104970089	105929836	CARD17	Loss	No	No
A7	chr11	107197071	107328572	CWF19L2	Loss	No	Yes
A7	chr11	108412378	110561352	EXPH5	Loss	Yes	No
A7	chr11	113186964	113320810	TTC12	Loss	No	No
A7	chr11	113264202	113577095	ANKK1	Loss	No	No
A7	chr12	247432	995250	IQSEC3,LOC574538	Loss	No	No
A7	chr12	252404	319209	IQSEC3,LOC574538	Loss	No	No
A7	chr12	3387586	3806201	TSPAN9	Loss	No	No
A7	chr12	7060433	7053815	PTPN6	Loss	No	No
A7	chr12	8286364	8803185	CLEC4A	Loss	No	No
A7	chr12	10194064	10171399	CLEC9A	Loss	No	No
A7	chr12	10853852	10787285	YBX3	Loss	No	No
A7	chr12	12588561	15747991	LOH12CR1	Loss	No	No
A7	chr12	13061176	13232943	GPRC5A	Loss	No	No
A7	chr12	14767800	16430619	GUCY2C	Loss	No	No
A7	chr12	18434937	18801352	PIK3C2G	Loss	No	No
A7	chr12	20852485	20903726	SLCO1C1	Loss	No	No
A7	chr12	29524460	30904067	ERGIC2	Loss	No	No
A7	chr12	30784828	30829521	IPO8	Loss	No	No
A7	chr12	39064530	41423021	CPNE8	Loss	No	No
A7	chr12	39695281	39763713	KIF21A	Loss	No	No
A7	chr12	44913787	46285701	NELL2	Loss	Yes	No
A7	chr12	45609769	45686717	PLEKHA8P1	Loss	No	No
A7	chr12	46591502	46633676	SLC38A1	Loss	No	No

A7	chr12	46756066	47472848	SLC38A2	Loss	No	No
A7	chr12	47162092	47186966	SLC38A4	Loss	No	No
A7	chr12	48105415	48196057	ENDOU	Loss	No	No
A7	chr12	48516429	48501977	PFKM	Loss	No	No
A7	chr12	49908337	50062337	SPATS2	Loss	No	No
A7	chr12	50025183	50031607	PRPF40B	Loss	No	No
A7	chr12	52056547	51899714	SCN8A	Loss	No	No
A7	chr12	52628938	52995322	KRT7	Loss	No	No
A7	chr12	52984619	53045959	KRT72	Loss	No	No
A7	chr12	53085721	53917168	KRT77	Loss	No	No
A7	chr12	53447194	53400280	TENC1,LOC283335	Loss	No	No
A7	chr12	54955227	54982443	PDE1B	Loss	No	No
A7	chr12	55367260	56154424	TESPA1	Loss	No	No
A7	chr12	57011178	57406659	BAZ2A	Loss	No	No
A7	chr12	58019018	57994861	SLC26A10	Loss	No	No
A7	chr12	58220778	59308117	CTDSP2	Loss	No	No
A7	chr12	64238540	64875801	SRGAP1	Loss	No	No
A7	chr12	64838854	65639719	XPOT	Loss	No	No
A7	chr12	66221780	66639042	HMGA2	Loss	No	No
A7	chr12	70671911	70740104	CNOT2	Loss	No	No
A7	chr12	70918250	71031220	PTPRB	Loss	No	No
A7	chr12	72666528	73050788	TRHDE,TRHDE-AS1	Loss	No	No
A7	chr12	74678804	75892500	LOC100507377	Loss	No	No
A7	chr12	75716956	77243250	CAPS2	Loss	No	No
A7	chr12	75895702	76468019	GLIPR1,KRR1	Loss	No	No
A7	chr12	78225068	78598918	NAV3	Loss	Yes	No
A7	chr12	79611282	82873016	SYT1	Loss	No	No
A7	chr12	80172329	80328731	PPP1R12A	Loss	Yes	No
A7	chr12	80605728	80770982	OTOGL	Loss	No	No
A7	chr12	81205243	81647449	LIN7A	Loss	No	No
A7	chr12	81763068	81072504	PPFIA2	Loss	Yes	No
A7	chr12	82748273	85286087	CCDC59	Loss	No	No
A7	chr12	83080933	89860699	TMTC2	Loss	No	No
A7	chr12	91449172	91502777	KERA	Loss	No	No

A7	chr13	32691470	33226144	FRY	Loss	Yes	No
A7	chr13	34395268	33692400	RFC3	Loss	Yes	No
A7	chr13	48954300	49741439	RB1	Loss	No	No
A7	chr14	59730158	59834317	DAAM1	Loss	No	No
A7	chr14	81864638	80271533	STON2	Loss	No	No
A7	chr15	20636345	20666595	HERC2P3	Loss	Yes	No
A7	chr15	20773683	20876597	GOLGA8CP	Loss	No	No
A7	chr15	22935859	23000243	CYFIP1	Loss	No	No
A7	chr15	25923859	26866681	ATP10A	Loss	No	No
A7	chr15	26961760	28456291	GABRB3	Loss	Yes	No
A7	chr15	28000022	28327041	OCA2	Loss	No	No
A7	chr15	29416889	31369187	FAM189A1	Loss	No	No
A7	chr15	29996425	30092905	TJP1	Loss	No	No
A7	chr15	31235092	31283807	FAN1,MTMR10	Loss	No	No
A7	chr15	31793892	33360233	OTUD7A	Loss	No	No
A7	chr15	32895386	34445270	GOLGA8R	Loss	No	No
A7	chr15	34549842	38852206	SLC12A6	Loss	No	No
A7	chr15	37186917	37390400	MEIS2	Loss	Yes	No
A7	chr15	38228514	39886641	TMCO5A	Loss	No	No
A7	chr15	40581472	40566477	PLCB2	Loss	No	No
A7	chr15	42067364	42116767	MAPKBP1	Loss	No	No
A7	chr15	42131010	42128749	JMJD7-PLA2G4B	Loss	No	No
A7	chr15	42140343	42169554	JMJD7-PLA2G4B,PLA2G4B	Loss	No	No
A7	chr15	42362106	42448839	PLA2G4D	Loss	No	No
A7	chr15	42453879	42500502	VPS39	Loss	No	No
A7	chr15	45386760	45402908	DUOX2	Loss	Yes	No
A7	chr15	45424115	45421102	DUOX1	Loss	No	No
A7	chr15	48060777	48593579	SEMA6D	Loss	No	No
A7	chr15	49126965	48937147	SHC4	Loss	No	No
A7	chr15	49917309	50528589	DTWD1	Loss	No	No
A7	chr15	50154441	50399205	ATP8B4	Loss	No	No
A7	chr15	50534145	50550714	HDC	Loss	No	No
A7	chr15	52446136	54825264	GNB5	Loss	No	No
A7	chr15	56382730	57490023	RFX7	Loss	Yes	No

A7	chr15	62147069	62336454	VPS13C	Loss	Yes	No
A7	chr15	63579622	63128272	APH1B	Loss	No	No
A7	chr15	67358194	67786704	SMAD3	Loss	No	No
A7	chr15	68596109	69652470	ITGA11	Loss	No	No
A7	chr15	69452972	70388600	GLCE	Loss	No	No
A7	chr15	69738347	69349501	KIF23	Loss	No	No
A7	chr15	82443805	82555104	EFTUD1	Loss	Yes	No
A7	chr15	86686929	87572283	AGBL1	Loss	No	No
A7	chr15	88419987	88799399	NTRK3	Loss	Yes	No
A7	chr16	19883137	20362161	GPRC5B	Loss	No	No
A7	chr16	20422791	20570769	ACSM5	Loss	No	No
A7	chr16	20471428	20817795	ACSM2A	Loss	No	No
A7	chr16	24769659	24834334	TNRC6A	Loss	No	No
A7	chr16	47495209	47730399	PHKB	Loss	No	No
A7	chr16	50659395	53326964	NKD1	Loss	No	No
A7	chr16	53639392	53730207	RPGRIP1L	Loss	No	No
A7	chr16	55559419	57684263	LPCAT2	Loss	No	No
A7	chr16	68248231	80838175	NFATC3	Loss	Yes	No
A7	chr16	70690862	70380951	IL34	Loss	No	No
A7	chr16	70972516	71025299	HYDIN	Loss	No	No
A7	chr16	72056260	72146811	DHODH	Loss	No	No
A7	chr16	77323160	78005830	ADAMTS18	Loss	No	No
A7	chr17	9448488	12664024	STX8	Loss	No	No
A7	chr17	10346607	10370101	MYH4	Loss	No	No
A7	chr17	11985215	12921381	MAP2K4	Loss	No	No
A7	chr17	18001583	18046154	DRG2	Loss	No	No
A7	chr17	18345751	18544666	KRT16P1	Loss	No	No
A7	chr17	19999943	20209395	SPECC1	Loss	No	No
A7	chr17	21092023	21191403	DHRS7B	Loss	No	No
A7	chr18	5394675	5489160	EPB41L3	Loss	Yes	No
A7	chr18	5890183	6909023	TMEM200C	Loss	No	No
A7	chr18	5960092	6311655	L3MBTL4	Loss	No	No
A7	chr18	9275521	8828962	ANKRD12	Loss	No	No
A7	chr18	9886585	10549093	TXNDC2	Loss	No	No

A7	chr18	34238037	34360022	FHOD3	Loss	No	No
A7	chr18	42618449	43547305	SETBP1	Loss	No	No
A7	chr18	43652121	44236996	PSTPIP2	Loss	No	No
A7	chr18	44683806	48510933	IER3IP1	Loss	No	No
A7	chr18	46145908	48248469	CTIF	Loss	No	No
A7	chr18	47109925	47797910	LIPG	Loss	No	No
A7	chr18	48422178	48466756	ME2	Loss	No	No
A7	chr18	48573289	52610030	SMAD4	Loss	No	No
A7	chr18	50278423	51053129	DCC	Loss	No	No
A7	chr18	52895451	53254367	TCF4	Loss	No	No
A7	chr18	54293392	55270175	TXNL1	Loss	No	No
A7	chr18	54339727	56648849	WDR7	Loss	No	No
A7	chr18	55368270	55816791	ATP8B1	Loss	No	No
A7	chr18	56118311	56898002	MIR122	Loss	No	No
A7	chr18	56148481	56279049	ALPK2	Loss	No	No
A7	chr18	56348401	56413023	MALT1	Loss	No	No
A7	chr18	56939592	61420354	RAX	Loss	No	No
A7	chr18	56998649	60033993	LMAN1	Loss	No	No
A7	chr18	59739905	60986185	PIGN	Loss	No	No
A7	chr18	61064266	64239553	VPS4B	Loss	No	No
A7	chr18	61582744	61571124	SERPINB10	Loss	No	No
A7	chr18	61653491	67614669	SERPINB8	Loss	No	No
A7	chr18	67671042	70534810	RTTN	Loss	No	No
A7	chr18	70205887	71807547	CBLN2	Loss	No	No
A7	chr18	71924064	74672811	CYB5A	Loss	No	No
A7	chr2	1639179	1418274	PXDN	Loss	No	No
A7	chr2	3405547	3341920	TRAPPC12	Loss	No	No
A7	chr2	7137046	8967259	RNF144A	Loss	No	No
A7	chr2	10095043	10807404	GRHL1	Loss	No	No
A7	chr2	20824491	20189884	HS1BP3	Loss	No	No
A7	chr2	26683514	26609404	OTOF	Loss	Yes	No
A7	chr2	29226357	31215873	FAM179A	Loss	No	No
A7	chr2	30457270	31806040	LBH	Loss	No	No
A7	chr2	31560506	31637611	XDH	Loss	No	No

A7	chr2	44436348	45832580	PPM1B	Loss	No	No
A7	chr2	50170841	51256332	NRXN1	Loss	Yes	No
A7	chr2	54161741	55433512	PSME4	Loss	No	No
A7	chr2	56411257	58381937	LOC101928647	Loss	No	No
A7	chr2	66662531	68051111	MEIS1	Loss	No	No
A7	chr2	68717321	69053957	APLF	Loss	No	No
A7	chr2	69585460	69784110	GFPT1	Loss	No	No
A7	chr2	70015185	70680459	ANXA4	Loss	No	No
A7	chr2	71595511	71662191	ZNF638	Loss	No	No
A7	chr2	72406443	73268060	EXOC6B	Loss	No	No
A7	chr2	74719430	74802706	TTC31	Loss	No	No
A7	chr2	79878677	77748874	CTNNA2	Loss	No	No
A7	chr2	84744942	88056945	DNAH6	Loss	No	No
A7	chr2	86437602	86718398	MRPL35	Loss	No	No
A7	chr2	88427469	89104922	FABP1	Loss	No	No
A7	chr2	97637637	97915910	FAM178B	Loss	No	No
A7	chr2	98127600	99198284	ANKRD36B	Loss	Yes	No
A7	chr2	98375355	98543950	TMEM131	Loss	No	No
A7	chr2	99943997	100081447	TXNDC9	Loss	No	No
A7	chr2	100627958	168074810	AFF3	Loss	No	No
A7	chr2	101541607	102505397	NPAS2	Loss	No	No
A7	chr2	102804315	102968497	IL1RL2	Loss	No	No
A7	chr2	103039637	103353337	IL18RAP	Loss	No	No
A7	chr2	106713173	106810795	UXS1	Loss	No	No
A7	chr2	109293061	110303814	LIMS1	Loss	No	No
A7	chr2	111525944	111431942	ACOXL	Loss	No	No
A7	chr2	116066814	113278002	DPP10	Loss	No	No
A7	chr2	118688612	118771739	CCDC93	Loss	No	No
A7	chr2	120362756	120414237	PCDP1	Loss	Yes	No
A7	chr2	120925452	122363756	EPB41L5	Loss	No	No
A7	chr2	121684936	120725548	GLI2	Loss	No	No
A7	chr2	121989401	128394488	TFCP2L1	Loss	Yes	No
A7	chr2	133483199	134275158	NCKAP5	Loss	No	No
A7	chr2	133551020	135975177	NCKAP5	Loss	No	No

A7	chr2	139307726	145154159	SPOPL	Loss	No	No
A7	chr2	149215771	150071772	MBD5	Loss	No	No
A7	chr2	152470789	152589699	NEB	Loss	No	No
A7	chr2	152732939	152424931	CACNB4	Loss	No	No
A7	chr2	154800906	165600385	GALNT13	Loss	No	No
A7	chr2	162224294	162903516	PSMD14	Loss	No	No
A7	chr2	163123988	163216062	IFIH1	Loss	No	No
A7	chr2	165809216	166243526	SLC38A11	Loss	No	No
A7	chr2	167266174	167334220	SCN7A	Loss	No	No
A7	chr2	169779448	168821236	ABCB11	Loss	Yes	No
A7	chr2	169989083	170219122	LRP2	Loss	Yes	No
A7	chr2	172248552	173679153	METTL8	Loss	No	No
A7	chr2	178370233	180409696	AGPS	Loss	No	No
A7	chr2	179402087	179669382	TTN,TTN-AS1	Loss	Yes	No
A7	chr2	189162218	190445537	GULP1	Loss	No	No
A7	chr2	190717380	190708806	PMS1	Loss	No	No
A7	chr2	211167211	210889940	MYL1	Loss	No	No
A7	chr2	212251577	217069119	ERBB4	Loss	Yes	No
A7	chr2	234923205	2001119	TRPM8	Loss	No	No
A7	chr20	7886800	9624987	HAO1	Loss	No	No
A7	chr20	9288446	16025284	PLCB4	Loss	No	No
A7	chr20	16351230	16496309	KIF16B	Loss	Yes	No
A7	chr22	18348689	18389652	MICAL3	Loss	No	No
A7	chr22	18769651	18918711	GGT3P	Loss	No	No
A7	chr3	3084596	3189387	CNTN4	Loss	No	No
A7	chr3	3133493	3118311	IL5RA	Loss	No	No
A7	chr3	8932072	9258673	RAD18	Loss	No	No
A7	chr3	11058744	11076384	SLC6A1,SLC6A1-AS1	Loss	No	No
A7	chr3	13360571	12943022	NUP210	Loss	No	No
A7	chr3	14163416	15045493	CHCHD4	Loss	No	No
A7	chr3	14724481	14814543	C3orf20	Loss	No	No
A7	chr3	15717404	15836813	ANKRD28	Loss	No	No
A7	chr3	16216183	17550097	GALNT15	Loss	No	No
A7	chr3	29322802	30032700	RBMS3	Loss	No	No

A7	chr3	30664690	32411813	TGFBR2	Loss	Yes	No
A7	chr3	31871531	33425693	OSBPL10	Loss	No	No
A7	chr3	33249299	33458326	SUSD5	Loss	No	No
A7	chr3	33557514	50336899	CLASP2	Loss	Yes	No
A7	chr3	37337577	36940750	GOLGA4	Loss	Yes	No
A7	chr3	37402733	38164228	GOLGA4	Loss	Yes	No
A7	chr3	37476334	38071154	C3orf35	Loss	No	No
A7	chr3	38207025	37998634	OXSR1	Loss	No	No
A7	chr3	38347444	38548459	SLC22A14	Loss	No	No
A7	chr3	39450096	38991860	RPSA	Loss	No	No
A7	chr3	39942277	40503152	MYRIP	Loss	No	No
A7	chr3	41265511	41280845	CTNNB1	Loss	No	No
A7	chr3	45879418	45436179	LZTFL1	Loss	No	No
A7	chr3	46398967	46944280	CCR2	Loss	No	No
A7	chr3	46713373	46747397	ALS2CL	Loss	No	No
A7	chr3	47030331	47018333	NBEAL2	Loss	Yes	Yes
A7	chr3	48607707	48611983	COL7A1	Loss	Yes	No
A7	chr3	48616351	48629900	COL7A1,MIR711	Loss	Yes	No
A7	chr3	49724581	49753147	MST1	Loss	No	No
A7	chr3	50307557	50314602	SEMA3B	Loss	No	No
A7	chr3	50384918	50383156	NPRL2	Loss	No	No
A7	chr3	50402318	50513630	CACNA2D2	Loss	No	No
A7	chr3	51349906	56593622	DOCK3	Loss	No	No
A7	chr3	52475279	52514311	SEMA3G	Loss	No	No
A7	chr3	52529355	52552629	STAB1	Loss	No	No
A7	chr3	52867632	52843025	MUSTN1,TMEM110-MUSTN1	Loss	No	No
A7	chr3	58090806	58141802	FLNB	Loss	Yes	No
A7	chr3	66431000	66421026	LRIG1	Loss	Yes	No
A7	chr3	69073205	68934461	TMF1	Loss	No	No
A7	chr3	130092453	130189811	COL6A5	Loss	Yes	No
A7	chr4	958963	996732	DGKQ	Loss	No	No
A7	chr4	2159584	7738899	POLN	Loss	No	No
A7	chr4	3176447	17844002	HTT	Loss	No	No
A7	chr4	5733151	6052406	EVC	Loss	No	No

A7	chr4	7770570	7873807	AFAP1,AFAP1-AS1	Loss	No	No
A7	chr4	8206893	8306006	SH3TC1	Loss	No	No
A7	chr4	17638150	18023483	FAM184B	Loss	No	No
A7	chr4	24529087	25014121	DHX15	Loss	No	No
A7	chr4	25262145	25366788	PI4K2B	Loss	No	No
A7	chr4	26321331	27019606	RBPJ	Loss	No	No
A7	chr4	30722036	36283651	PCDH7	Loss	No	No
A7	chr4	37590467	38134878	C4orf19	Loss	No	No
A7	chr4	41946809	109087953	TMEM33	Loss	No	No
A7	chr4	42003632	42629126	SLC30A9	Loss	No	No
A7	chr4	46053440	47913011	GABRG1	Loss	Yes	No
A7	chr4	47514520	47644066	ATP10D	Loss	No	No
A7	chr4	48140681	53522759	TEC	Loss	No	No
A7	chr4	48503639	48636507	FRYL	Loss	Yes	No
A7	chr4	48988264	48422460	CWH43	Loss	No	No
A7	chr4	62362838	72205222	LPHN3	Loss	Yes	No
A7	chr4	65146758	66509145	TECRL	Loss	No	No
A7	chr4	96104047	96470361	UNC5C	Loss	No	No
A7	chr4	100131238	100274202	ADH6,LOC100507053	Loss	No	No
A7	chr4	103647745	104119566	MANBA	Loss	No	No
A7	chr4	113541157	114680570	C4orf21	Loss	No	No
A7	chr4	128748459	128635248	HSPA4L	Loss	No	No
A7	chr4	129083348	130003528	LARP1B	Loss	No	No
A7	chr4	141446573	142145831	ELMOD2	Loss	Yes	No
A7	chr4	144797898	145061904	GYPE	Loss	No	No
A7	chr4	146073692	146046253	OTUD4	Loss	No	No
A7	chr4	151023629	151174708	DCLK2	Loss	Yes	No
A7	chr4	153245335	153893693	FBXW7	Loss	No	No
A7	chr4	153564239	155490951	TMEM154	Loss	No	No
A7	chr4	156849487	159092751	CTSO	Loss	No	No
A7	chr4	160251485	164050500	RAPGEF2	Loss	No	No
A7	chr4	178911632	185350224	LOC285501	Loss	No	No
A7	chr4	185678972	185724700	ACSL1	Loss	No	No
A7	chr6	488978	637861	EXOC2	Loss	No	No

A7	chr6	15374347	15663289	JARID2	Loss	No	No
A7	chr6	56324928	56496838	DST	Loss	No	No
A7	chr6	70840073	70916682	COL19A1	Loss	No	No
A7	chr6	101846860	102511923	GRIK2	Loss	No	No
A7	chr6	123590993	123958238	TRDN	Loss	No	No
A7	chr6	146956510	147123147	ADGB	Loss	No	No
A7	chr8	6357372	4852328	ANGPT2,MCPH1	Loss	No	No
A7	chr8	25266317	92406267	DOCK5	Loss	No	No
A7	chr8	35406809	36793643	UNC5D	Loss	No	No
A7	chr8	51306771	51664671	SNTG1	Loss	No	No
A7	chr8	61653817	61484672	CHD7	Loss	No	No
A7	chr8	62212235	62627199	CLVS1	Loss	No	No
A7	chr8	79650738	77768536	IL7	Loss	No	No
A7	chr8	87556967	92053203	CPNE3	Loss	No	No
A7	chr8	132952745	133645209	EFR3A	Loss	Yes	No
A7	chr8	134114796	133856563	SLA,TG	Loss	No	No
A7	chr9	177722	463687	CBWD1	Loss	No	No
A7	chr9	2635452	2652949	VLDLR	Loss	No	No
A7	chr9	3898645	4666256	GLIS3	Loss	No	No
A7	chr9	4722505	5126446	AK3	Loss	No	No
A7	chr9	4850296	5463121	RCL1	Loss	No	No
A7	chr9	15571621	14674383	CCDC171	Loss	No	No
A7	chr9	19325936	19033256	DENND4C	Loss	No	No
A7	chr9	19376253	22009312	RPS6	Loss	No	No
A7	chr9	20353522	20620833	MLLT3	Loss	No	No
A8	chr1	145588377	145516456	NUDT17	Gain	No	No
A8	chr1	150551318	150531942	MCL1	Gain	No	No
A8	chr1	150939231	150967175	CERS2	Gain	No	No
A8	chr1	153609047	153974461	CHTOP	Gain	No	No
A8	chr1	153634263	153745557	SNAPIN	Gain	No	No
A8	chr1	155880668	155989958	RIT1	Gain	No	No
A8	chr1	156568759	156708230	GPATCH4	Gain	No	No
A8	chr1	201832500	201860696	IPO9	Gain	No	No
A8	chr10	327139	532470	DIP2C	Gain	Yes	No

A8	chr10	860681	931700	LARP4B	Gain	Yes	No
A8	chr10	1206492	3215033	LINC00200	Gain	No	No
A8	chr10	3124579	3109899	PFKP	Gain	No	No
A8	chr10	5199835	5498236	AKR1CL1	Gain	No	No
A8	chr10	5762506	6557106	FAM208B	Gain	No	No
A8	chr10	5808203	5855512	GDI2	Gain	No	No
A8	chr10	6060015	6157274	IL2RA	Gain	Yes	No
A8	chr10	7605078	8057016	ITIH5	Gain	No	No
A8	chr10	11532830	15760898	USP6NL	Gain	Yes	No
A8	chr10	11639629	13672868	USP6NL	Gain	Yes	No
A8	chr10	11893995	13175581	PROSER2-AS1,PROSER2	Gain	No	No
A8	chr10	11971863	12077440	UPF2	Gain	No	No
A8	chr10	13040337	13380808	CCDC3	Gain	No	No
A8	chr10	13696415	14943261	FRMD4A	Gain	Yes	No
A8	chr10	14882073	14996431	HSPA14	Gain	No	No
A8	chr10	15145303	15197346	RPP38	Gain	No	No
A8	chr10	15151700	15880278	NMT2	Gain	No	No
A8	chr10	17195505	18134122	TRDMT1	Gain	No	No
A8	chr10	21823100	22029165	MLLT10	Gain	No	No
A8	chr10	22028958	35104253	MLLT10	Gain	No	No
A8	chr10	25140316	33619810	PRTFDC1	Gain	No	No
A8	chr10	25312104	26590048	THNSL1,ENKUR	Gain	No	No
A8	chr10	26727265	26501465	APBB1IP	Gain	No	No
A8	chr10	26939482	27389427	LINC00202-2	Gain	No	No
A8	chr10	26993605	27035726	PDSS1	Gain	No	No
A8	chr10	27447477	27443349	MASTL	Gain	No	No
A8	chr10	27493368	27529808	ACBD5	Gain	No	No
A8	chr10	28149553	28284109	ARMC4	Gain	No	No
A8	chr10	28287731	28527664	ARMC4	Gain	No	No
A8	chr10	29747362	29844037	SVIL	Gain	No	No
A8	chr10	29891192	30748430	SVIL	Gain	No	No
A8	chr10	32097576	32197854	ARHGAP12	Gain	No	No
A8	chr10	32856650	32594859	C10orf68,CCDC7	Gain	No	No
A8	chr10	33195990	33224486	ITGB1	Gain	No	No

A8	chr10	35300775	35360267	CUL2	Gain	No	Yes
A8	chr10	35379357	35426807	CUL2	Gain	No	Yes
A8	chr10	38121952	38667433	ZNF248	Gain	No	No
A8	chr11	123601194	123471256	ZNF202	Gain	No	No
A8	chr11	125221179	126301421	PKNOX2	Gain	No	No
A8	chr11	125465775	125496470	STT3A	Gain	No	No
A8	chr11	126137897	126139186	SRPR	Gain	No	No
A8	chr11	129722369	129830977	TMEM45B	Gain	No	No
A8	chr11	130058008	130340996	ST14	Gain	No	No
A8	chr12	49255791	49239565	RND1	Gain	No	No
A8	chr12	49742548	49718171	DNAJC22	Gain	No	No
A8	chr14	31570385	31675163	HECTD1	Gain	No	No
A8	chr14	32068494	31858211	NUBPL	Gain	No	No
A8	chr14	33204863	35008943	AKAP6	Gain	No	No
A8	chr14	34396165	35099160	EGLN3	Gain	No	No
A8	chr14	36946175	39606177	SFTA3	Gain	No	No
A8	chr14	38064105	39784003	FOXA1	Gain	No	No
A8	chr14	50050289	50110388	RPS29	Gain	No	No
A8	chr14	50586992	50778947	SOS2	Gain	No	No
A8	chr14	50798643	51194469	CDKL1,ATP5S	Gain	No	No
A8	chr14	53112871	53251382	ERO1L	Gain	No	No
A8	chr14	55308723	55251338	GCH1	Gain	Yes	No
A8	chr14	64407331	64679723	SYNE2	Gain	Yes	No
A8	chr14	64699746	64925025	ESR2	Gain	Yes	No
A8	chr14	64988204	65211060	ZBTB1	Gain	No	No
A8	chr14	67664852	68257490	FAM71D	Gain	No	No
A8	chr14	68151731	74454875	RDH11	Gain	No	No
A8	chr14	73406509	73404778	DCAF4	Gain	No	No
A8	chr14	74035762	74181128	ACOT2	Gain	No	No
A8	chr14	74358729	74349141	ZNF410	Gain	No	No
A8	chr14	74531524	74523961	ALDH6A1,CCDC176	Gain	No	No
A8	chr14	75537257	75528465	ZC2HC1C	Gain	No	No
A8	chr14	77808113	77920445	TMED8	Gain	No	No
A8	chr14	77860364	77935815	NOXRED1	Gain	No	No

A8	chr14	77984380	77857587	SPTLC2	Gain	No	No
A8	chr14	78234794	78227497	C14orf178	Gain	No	No
A8	chr14	88654310	89016963	KCNK10	Gain	Yes	No
A8	chr14	89082485	91252672	EML5	Gain	No	No
A8	chr14	90650454	90499558	KCNK13	Gain	No	No
A8	chr14	91113114	91957146	TTC7B	Gain	No	No
A8	chr14	91633812	92560175	C14orf159	Gain	No	No
A8	chr14	92047117	92191512	CATSPERB	Gain	No	No
A8	chr14	92343830	92790304	FBLN5	Gain	No	No
A8	chr14	92439060	93688720	TRIP11	Gain	No	No
A8	chr14	93760203	97029230	BTBD7	Gain	No	No
A8	chr14	93813536	94423216	UNC79	Gain	No	No
A8	chr14	93943903	94173689	UNC79	Gain	No	No
A8	chr14	94465682	94546093	LINC00521	Gain	No	No
A8	chr14	100135168	100774293	HHIPL1	Gain	No	No
A8	chr14	100531750	99872927	EVL	Gain	No	No
A8	chr14	102659799	103369766	WDR20	Gain	No	No
A8	chr14	102695157	103806898	MOK	Gain	No	No
A8	chr14	102792321	102829253	ZNF839	Gain	No	No
A8	chr14	103404440	103478525	CDC42BPB	Gain	No	No
A8	chr14	103871412	103970166	MARK3	Gain	No	No
A8	chr14	103964838	104516017	MARK3	Gain	No	No
A8	chr14	104151322	105071097	KLC1	Gain	No	No
A8	chr14	104202339	104263855	PPP1R13B	Gain	No	No
A8	chr14	105181620	105271048	INF2	Gain	No	No
A8	chr14	105477496	105518439	CDCA4	Gain	No	No
A8	chr14	105609818	105634444	JAG2	Gain	No	No
A8	chr14	105995058	105941242	TMEM121	Gain	No	No
A8	chr16	28916717	28935936	RABEP2	Gain	No	No
A8	chr16	30419365	30531288	ZNF771	Gain	No	No
A8	chr17	26495473	27028146	NLK	Gain	No	No
A8	chr17	26883844	26941211	PIGS	Gain	No	No
A8	chr17	27946668	28444190	CORO6	Gain	No	No
A8	chr17	28937906	29151778	LRRC37BP1	Gain	No	No

A8	chr17	29647390	30695033	EVI2A,NF1	Gain	No	No
A8	chr17	34892942	34951610	PIGW	Gain	No	No
A8	chr17	37099030	37949186	FBXO47	Gain	No	No
A8	chr17	38345117	38508322	RAPGEFL1	Gain	No	No
A8	chr17	38927405	40666478	KRT26	Gain	No	No
A8	chr17	39871683	39992209	GAST	Gain	No	No
A8	chr17	39911981	39928114	JUP	Gain	No	No
A8	chr17	40272242	40333296	KAT2A	Gain	No	No
A8	chr17	40354357	40384155	STAT5B	Gain	No	No
A8	chr17	40854894	36700204	EZH1	Gain	Yes	No
A8	chr17	41168323	41267796	VAT1	Gain	No	No
A8	chr17	42083391	42152455	NAGS	Gain	No	No
A8	chr17	42249413	42430470	ASB16	Gain	No	No
A8	chr17	42836567	42856607	ADAM11	Gain	No	No
A8	chr17	42927654	42971893	HIGD1B	Gain	No	No
A8	chr17	43342529	43517000	MAP3K14,MAP3K14-AS1	Gain	No	No
A8	chr17	46846350	46806111	TTLL6	Gain	No	No
A8	chr17	46871565	47022154	TTLL6	Gain	No	No
A8	chr17	47210130	47209872	B4GALNT2	Gain	No	No
A8	chr17	49091592	48769063	SPAG9	Gain	No	No
A8	chr17	56646552	56729363	TEX14	Gain	Yes	No
A8	chr17	57208641	57350197	SKA2	Gain	No	No
A8	chr17	57918626	58022879	VMP1	Gain	No	No
A8	chr17	59946394	60005377	INTS2	Gain	No	No
A8	chr17	61776158	61819330	LIMD2	Gain	No	No
A8	chr17	65716021	66033323	NOL11	Gain	No	No
A8	chr17	70427268	71433961	LINC00673	Gain	No	No
A8	chr17	72277948	72310410	DNAI2	Gain	Yes	No
A8	chr17	72948363	73035158	HID1	Gain	No	No
A8	chr17	73621606	73747192	MYO15B	Gain	No	No
A8	chr17	73805840	73819603	UNK	Gain	No	No
A8	chr17	73909782	74151398	FBF1	Gain	No	No
A8	chr17	73969705	73987704	ACOX1	Gain	No	No
A8	chr17	74157923	73571290	RNF157	Gain	No	No

A8	chr17	76422537	76568976	DNAH17	Gain	Yes	No
A8	chr17	76794989	76832454	USP36	Gain	Yes	No
A8	chr17	78083743	78063683	GAA	Gain	No	No
A8	chr17	78109802	79288281	EIF4A3	Gain	No	No
A8	chr17	78196453	78180884	SLC26A11	Gain	No	No
A8	chr17	79059453	79058693	BAIAP2	Gain	Yes	No
A8	chr17	80022001	80051647	DUS1L	Gain	No	No
A8	chr17	80332200	80400516	UTS2R	Gain	No	No
A8	chr2	60780350	61761038	BCL11A	Gain	Yes	No
A8	chr2	61258553	62115806	PEX13	Gain	No	No
A8	chr2	61361271	62363205	KIAA1841	Gain	No	No
A8	chr20	29631537	30157370	FRG1B	Gain	No	No
A8	chr20	29993897	30457400	DEFB121	Gain	No	No
A8	chr20	30457850	31027122	DUSP15	Gain	No	No
A8	chr20	31040038	31315949	C20orf112	Gain	No	No
A8	chr20	31368123	31367951	DNMT3B	Gain	No	No
A8	chr20	32194761	32228337	CBFA2T2	Gain	No	No
A8	chr20	32661624	34099803	RALY	Gain	No	No
A8	chr20	32873245	32666359	AHCY	Gain	No	No
A8	chr20	32891048	33147257	AHCY	Gain	No	No
A8	chr20	33054129	33422265	ITCH	Gain	No	No
A8	chr20	33865811	34147394	MMP24-AS1	Gain	No	No
A8	chr20	34389412	35399876	PHF20	Gain	Yes	No
A8	chr20	34995447	35128079	DLGAP4	Gain	No	No
A8	chr20	35173261	35176596	MYL9	Gain	No	No
A8	chr20	35284762	35350186	NDRG3	Gain	No	No
A8	chr20	35399275	35580246	DSN1	Gain	No	No
A8	chr20	35879489	35870025	GHRH	Gain	Yes	No
A8	chr20	36012552	36031283	SRC	Gain	No	No
A8	chr20	36560036	35752202	VSTM2L	Gain	No	No
A8	chr20	36611422	36766787	TTI1	Gain	Yes	No
A8	chr20	36888576	37005653	KIAA1755	Gain	No	No
A8	chr20	37117045	37217104	RALGAPB	Gain	No	No
A8	chr20	39766353	42170535	PLCG1	Gain	No	No

A8	chr20	43850349	44037246	SEMG2	Gain	No	No
A8	chr20	43935482	43945220	MATN4	Gain	No	No
A8	chr20	44098393	44054885	WFDC2	Gain	No	No
A8	chr20	44175905	45985633	EPPIN,EPPIN-WFDC6	Gain	No	No
A8	chr20	44567619	44166733	PCIF1	Gain	No	No
A8	chr20	47244398	48503435	PREX1	Gain	No	No
A8	chr20	47674989	47685359	CSE1L	Gain	No	No
A8	chr20	47732318	47770608	STAU1	Gain	No	No
A8	chr20	47862438	48553089	ZNFX1	Gain	Yes	No
A8	chr20	49354393	49626901	PARD6B	Gain	No	No
A8	chr20	60318618	60509278	CDH4	Gain	No	No
A8	chr20	60884771	60937608	LAMA5	Gain	Yes	No
A8	chr20	61150671	61430401	C20orf166	Gain	No	No
A8	chr20	61929261	61959850	COL20A1	Gain	No	No
A8	chr20	62039765	62103993	KCNQ2	Gain	No	No
A8	chr20	62273460	62339365	STMN3	Gain	No	No
A8	chr5	32415073	32276848	ZFR	Gain	No	No
A8	chr5	37044448	37371228	NIPBL	Gain	No	No
A8	chr6	24436803	24480187	GPLD1	Gain	No	No
A8	chr6	35443112	35745358	TEAD3	Gain	No	No
A8	chr6	42585146	42976213	UBR2	Gain	No	No
A8	chr6	42979966	42946981	MEA1,PPP2R5D	Gain	No	No
A8	chr6	43495896	43495469	POLR1C,XPO5	Gain	No	No
A8	chr6	43603576	43623428	MAD2L1BP	Gain	No	No
A8	chr6	43969789	44081946	C6orf223	Gain	Yes	No
A8	chr6	52369388	52441862	TRAM2	Gain	No	No
A8	chr6	52541884	52859059	TMEM14A	Gain	No	No
A8	chr6	52935854	52960462	FBXO9	Gain	No	No
A8	chr6	52991759	53156761	GCM1	Gain	Yes	No
A8	chr6	53364842	53409927	GCLC	Gain	Yes	No
A8	chr6	53380906	54254950	GCLC	Gain	Yes	No
A8	chr7	44302603	44467302	CAMK2B	Gain	No	No
A8	chr7	44618761	44806262	TMED4	Gain	No	No
A8	chr7	56146056	56156668	SUMF2	Gain	No	No

A8	chr7	65150571	66237065	LOC441242,INTS4L2	Gain	No	No
A8	chr7	65219513	65447301	LOC441242,CCT6P1	Gain	No	No
A8	chr7	66406834	66774702	TMEM248	Gain	No	No
A8	chr7	70880874	72755381	WBSCR17	Gain	No	No
A8	chr7	72861593	74016920	BAZ1B	Gain	No	No
A8	chr7	73630276	73639090	LAT2	Gain	No	No
A8	chr7	74225429	74565623	GTF2IRD2	Gain	No	No
A8	chr7	75028199	75046071	TRIM73,SPDYE5	Gain	No	No
A8	chr7	75048602	75104454	SPDYE5,POM121C	Gain	No	No
A8	chr7	75124298	75616173	SPDYE5	Gain	No	No
A8	chr7	75988038	76071388	YWHAG	Gain	No	No
A8	chr7	97820039	98741743	LMTK2	Gain	No	No
A8	chr7	98630516	98805089	SMURF1	Gain	No	No
A8	chr7	99074025	99006305	ZNF789	Gain	No	No
A8	chr7	99691182	99691470	MCM7	Gain	No	No
A8	chr7	99930064	100029312	PMS2P1	Gain	No	No
A8	chr7	100161456	100421895	AGFG2	Gain	No	No
A8	chr7	100173327	100183811	LRCH4	Gain	No	No
A8	chr7	100490785	100486285	ACHE	Gain	No	No
A8	chr7	100771673	100860067	SERPINE1	Gain	No	No
A8	chr7	102158092	102113612	RASA4B	Gain	Yes	No
A8	chr7	105173270	105205894	RINT1	Gain	No	No
A8	chr7	105250975	105148991	ATXN7L1	Gain	No	No
A8	chr8	25715834	26510913	EBF2	Gain	No	No
A8	chr8	27646363	28821407	ESCO2	Gain	No	No
A8	chr8	28285925	28331324	FBXO16	Gain	No	No
A8	chr8	28929136	29120610	KIF13B	Gain	No	No
A8	chr8	30001998	30609035	MBOAT4	Gain	No	No
A8	chr8	30538420	30569605	GSR	Gain	No	No
A8	chr8	68189516	71128999	ARFGEF1	Gain	No	No
A8	chr8	73142531	73951454	LOC392232	Gain	No	No
A8	chr8	81555269	80976828	ZNF704	Gain	No	No
A8	chr8	86021829	86129731	LRRCC1	Gain	Yes	No
A8	chr8	86376810	87755903	CA2	Gain	Yes	No

A8	chr8	89179897	97620698	MMP16	Gain	No	No
A8	chr8	95502166	95709189	KIAA1429	Gain	No	No
A8	chr8	97797091	99306621	CPQ	Gain	No	No
A8	chr8	100974914	101117630	RGS22	Gain	No	No
A8	chr8	103311075	103424917	UBR5	Gain	No	No
A8	chr8	105405011	109499136	DPYS	Gain	No	No
A8	chr8	120233826	121444363	MAL2	Gain	No	No
A8	chr8	124544137	124164392	FBXO32	Gain	No	No
A8	chr8	124820511	125500859	FAM91A1	Gain	No	No
A8	chr8	125025833	125740748	FER1L6-AS1,FER1L6	Gain	No	No
A8	chr8	131964113	133816277	ADCY8	Gain	Yes	No
A8	chr8	133015482	133687863	EFR3A	Gain	Yes	No
A8	chr8	139631697	141461482	COL22A1	Gain	No	No
A8	chr8	144391496	144623620	TOP1MT	Gain	No	No
A8	chr8	144649780	144654928	MROH6	Gain	No	No
A8	chr8	144789835	144804633	CCDC166	Gain	No	No
A8	chr8	145016559	145141119	PLEC	Gain	No	No
A8	chr8	145559716	145545071	SCRT1	Gain	No	No
A8	chr8	145579627	145537717	FBXL6	Gain	No	No
A8	chr8	145734551	146005665	MFSD3	Gain	No	No
A8	chrX	47930431	48054814	ZNF630	Gain	No	No
A8	chrX	47969343	48901043	SSX6	Gain	No	No
A8	chrX	48116656	48214704	SSX1	Gain	No	No
A8	chrX	48270192	48328644	SSX4B	Gain	No	No
A8	chrX	48433555	48814893	RBM3	Gain	No	No
A8	chrX	48457104	48379202	WDR13	Gain	No	No
A8	chrX	48776050	48858675	PIM2	Gain	No	No
A8	chrX	48919489	48931704	CCDC120	Gain	No	No
A8	chrX	49108124	49114984	FOXP3	Gain	No	No
A8	chrX	49114120	49055683	FOXP3	Gain	No	No
A8	chrX	49452053	49834399	PAGE1	Gain	No	No
A8	chrX	49689780	49855543	CLCN5	Gain	No	No
A8	chrX	49955419	51489326	AKAP4	Gain	No	No
A8	chrX	50028126	50213737	CCNB3	Gain	No	No

A8	chrX	52654027	52727138	SSX8	Gain	No	No
A8	chrX	54106664	54209691	FAM120C	Gain	No	No
A8	chrX	70367399	70678207	NLGN3	Gain	No	No
A8	chr1	8397875	92263028	SLC45A1	Loss	No	No
A8	chr1	19431078	19500941	UBR4	Loss	Yes	No
A8	chr1	25166350	16774469	CLIC4	Loss	No	No
A8	chr1	34066464	34554794	CSMD2	Loss	Yes	No
A8	chr1	55537989	55643805	USP24	Loss	No	No
A8	chr1	57140053	57420500	PRKAA2	Loss	No	No
A8	chr1	60287529	60336782	HOOK1	Loss	No	No
A8	chr1	64474976	64120137	ROR1	Loss	No	No
A8	chr1	65016276	61543300	CACHD1	Loss	No	No
A8	chr1	65656392	65352024	AK4	Loss	No	No
A8	chr1	65830317	65280523	DNAJC6	Loss	No	No
A8	chr1	66378927	66379086	PDE4B	Loss	No	No
A8	chr1	66999824	67207119	SGIP1	Loss	No	No
A8	chr1	68511644	68962454	GNG12-AS1	Loss	No	No
A8	chr1	74506896	76276492	LRRIQ3	Loss	No	No
A8	chr1	74701070	74667094	FPGT-TNNI3K	Loss	No	No
A8	chr1	76282057	76007201	MSH4	Loss	Yes	No
A8	chr1	77093136	90152170	ST6GALNAC3	Loss	No	No
A8	chr1	78470635	78959226	DNAJB4	Loss	No	No
A8	chr1	79115870	79107294	IFI44	Loss	Yes	No
A8	chr1	79357208	84791431	ELTD1	Loss	Yes	No
A8	chr1	84679835	84610231	PRKACB	Loss	No	No
A8	chr1	85035613	85136991	CTBS	Loss	No	No
A8	chr1	85537610	85594482	WDR63	Loss	No	No
A8	chr1	86252036	86622121	COL24A1	Loss	Yes	No
A8	chr1	86818541	86852769	ODF2L	Loss	No	No
A8	chr1	89149921	89528936	PKN2	Loss	No	No
A8	chr1	89598940	89735257	GBP7	Loss	No	No
A8	chr10	49371370	50824138	FRMPD2	Loss	No	No
A8	chr10	49609654	50189523	MAPK8	Loss	No	No
A8	chr10	51028210	50741024	PARG	Loss	Yes	No

A8	chr10	60036876	61115703	CISD1	Loss	No	No
A8	chr10	73572524	69407276	CDH23	Loss	Yes	No
A8	chr10	87379623	85984870	GRID1	Loss	No	No
A8	chr10	89527429	90708710	ATAD1	Loss	No	No
A8	chr10	100481413	125806240	HPSE2	Loss	No	No
A8	chr10	105797393	105796871	COL17A1	Loss	No	No
A8	chr10	115350336	115423829	NRAP	Loss	No	No
A8	chr10	115950719	116008524	TDRD1	Loss	No	No
A8	chr10	121556226	121588662	INPP5F	Loss	No	No
A8	chr10	124799981	128019078	ACADSB	Loss	No	No
A8	chr12	6230339	5963307	VWF	Loss	No	No
A8	chr12	9087719	9393135	PHC1	Loss	No	No
A8	chr12	9463736	14693836	LOC642846	Loss	No	No
A8	chr12	13093708	13128413	GPRC5D	Loss	No	No
A8	chr12	20709593	21392730	PDE3A	Loss	Yes	No
A8	chr12	30787016	30829521	IPO8	Loss	No	No
A8	chr12	64746673	64521504	C12orf56	Loss	No	No
A8	chr12	65448897	65639719	WIF1	Loss	No	No
A8	chr12	70793988	71974203	KCNMB4	Loss	No	No
A8	chr12	75676030	75892500	CAPS2	Loss	No	No
A8	chr12	76750405	77243250	OSBPL8	Loss	No	No
A8	chr12	89992893	89992520	ATP2B1	Loss	No	No
A8	chr12	97303529	97301634	NEDD1	Loss	No	No
A8	chr12	99166819	95419031	ANKS1B	Loss	No	No
A8	chr12	100811806	101520852	SLC17A8	Loss	No	No
A8	chr12	102108281	104707095	CHPT1	Loss	No	No
A8	chr12	105504902	105382031	KIAA1033	Loss	No	No
A8	chr12	113274295	113333678	RPH3A	Loss	No	No
A8	chr12	117271596	116675510	RNFT2	Loss	No	No
A8	chr13	101717755	114240164	NALCN	Loss	Yes	No
A8	chr13	110804680	110864965	COL4A1	Loss	No	No
A8	chr14	23858601	23876445	MYH6	Loss	Yes	No
A8	chr14	23939250	23888504	NGDN	Loss	No	No
A8	chr15	31779396	33360233	OTUD7A	Loss	No	No

A8	chr15	34112956	34445270	RYR3	Loss	Yes	No
A8	chr15	34547462	38852206	SLC12A6	Loss	No	No
A8	chr15	38243236	38988940	TMCO5A	Loss	No	No
A8	chr15	39874029	39886641	THBS1	Loss	No	No
A8	chr15	47704589	48633782	SEMA6D	Loss	No	No
A8	chr15	51504516	51868378	CYP19A1	Loss	No	No
A8	chr15	52194088	52675385	TMOD3	Loss	No	No
A8	chr15	53815414	54025358	WDR72	Loss	No	No
A8	chr15	55930746	56999348	PRTG	Loss	No	No
A8	chr15	57732574	51397473	CGNL1	Loss	No	No
A8	chr15	62939509	63092692	TLN2	Loss	No	No
A8	chr15	67938568	67529158	MAP2K5	Loss	No	No
A8	chr15	73408880	73575452	NEO1	Loss	No	No
A8	chr15	76998243	77176217	SCAPER	Loss	No	No
A8	chr15	81517639	81598886	IL16	Loss	Yes	No
A8	chr15	86279320	87572283	AKAP13	Loss	No	No
A8	chr15	93563241	95020022	CHD2	Loss	Yes	No
A8	chr16	50783486	50827575	CYLD	Loss	No	No
A8	chr16	76350309	83712066	CNTNAP4	Loss	Yes	No
A8	chr17	9066129	10370101	NTN1	Loss	No	No
A8	chr17	9774063	10451257	GLP2R	Loss	No	No
A8	chr18	21330881	21451625	LAMA3	Loss	No	No
A8	chr18	21464736	21535029	LAMA3	Loss	No	No
A8	chr18	24442145	28673606	AQP4	Loss	No	No
A8	chr18	29098201	29246335	DSG2	Loss	No	No
A8	chr18	30517365	31327399	CCDC178	Loss	No	No
A8	chr18	33557372	8394609	C18orf21	Loss	Yes	No
A8	chr18	39537534	40695657	PIK3C3	Loss	No	No
A8	chr18	44057216	44236996	LOXHD1	Loss	No	No
A8	chr18	47323835	47581748	ACAA2	Loss	Yes	No
A8	chr18	51880423	52610030	STARD6	Loss	No	No
A8	chr18	55833019	55816791	NEDD4L	Loss	Yes	No
A8	chr18	60582145	59966093	PHLPP1	Loss	No	Yes
A8	chr18	61151654	61420354	SERPINB5	Loss	No	No

A8	chr18	61765810	67614669	LINC00305	Loss	No	No
A8	chr18	70209038	71807547	CBLN2	Loss	No	No
A8	chr2	97779232	97833342	ANKRD36	Loss	Yes	No
A8	chr2	97847457	97881312	ANKRD36	Loss	Yes	No
A8	chr2	99412582	99220654	KIAA1211L	Loss	No	No
A8	chr2	99725851	98453620	TSGA10	Loss	No	No
A8	chr2	100018706	100081447	REV1	Loss	No	No
A8	chr2	108477221	109004270	RGPD4	Loss	No	No
A8	chr2	111406810	111435684	BUB1	Loss	No	No
A8	chr2	121712820	120684242	GLI2	Loss	No	No
A8	chr2	122104613	122286356	CLASP1	Loss	No	No
A8	chr2	125175019	125669115	CNTNAP5	Loss	Yes	No
A8	chr2	135011829	136026711	MGAT5	Loss	No	No
A8	chr2	137748461	138425431	THSD7B	Loss	No	No
A8	chr2	139322509	144966371	SPOPL	Loss	No	No
A8	chr2	152739764	152698602	CACNB4	Loss	No	No
A8	chr2	153004738	153573975	STAM2	Loss	No	No
A8	chr2	157182661	158167913	NR4A2	Loss	No	No
A8	chr2	157332609	157436300	GPD2	Loss	No	No
A8	chr2	158851690	158656012	UPP2	Loss	No	No
A8	chr2	160206218	160335232	BAZ2B	Loss	No	No
A8	chr2	160673316	160755570	LY75,LY75-CD302	Loss	Yes	No
A8	chr2	160798637	160919126	PLA2R1	Loss	No	No
A8	chr2	161132140	161350318	RBMS1	Loss	No	No
A8	chr2	162080425	162904011	TANK	Loss	No	No
A8	chr2	162276706	163216062	TBR1	Loss	No	No
A8	chr2	163080975	165476329	FAP	Loss	No	No
A8	chr2	165948763	166032954	SCN3A	Loss	Yes	No
A8	chr2	166532821	166243526	CSRNP3	Loss	No	No
A8	chr2	166606251	166806234	GALNT3	Loss	No	No
A8	chr2	169985522	170113759	LRP2	Loss	Yes	No
A8	chr2	170770569	170937194	UBR3	Loss	No	No
A8	chr2	173330266	173366629	ITGA6	Loss	No	No
A8	chr2	173686314	173662341	RAPGEF4	Loss	No	No

A8	chr2	178494126	178769914	PDE11A	Loss	No	No
A8	chr2	179312231	180409696	PRKRA	Loss	No	No
A8	chr2	179400458	180014121	TTN,TTN-AS1	Loss	Yes	No
A8	chr2	179644564	182787139	TTN	Loss	Yes	No
A8	chr2	179701620	179742870	CCDC141	Loss	No	No
A8	chr2	183582667	183846104	DNAJC10	Loss	No	No
A8	chr2	186678271	197298182	FSIP2	Loss	No	No
A8	chr2	188332479	188250358	TFPI	Loss	No	No
A8	chr2	191235569	191161679	INPP1	Loss	No	No
A8	chr2	191399284	191874730	TMEM194B	Loss	No	No
A8	chr2	192141612	192280968	MYO1B	Loss	No	No
A8	chr2	196602426	197565904	SLC39A10	Loss	No	No
A8	chr2	201467989	201994869	AOX1	Loss	No	No
A8	chr2	202248833	202285429	TRAK2	Loss	No	No
A8	chr2	205550903	206418127	PARD3B	Loss	Yes	No
A8	chr2	207310030	207474724	ADAM23	Loss	No	No
A8	chr2	209176959	209219369	PIKFYVE	Loss	No	No
A8	chr2	209271554	208866681	PTH2R	Loss	No	No
A8	chr2	215595134	215440653	BARD1	Loss	No	No
A8	chr2	216983765	215275225	XRCC5	Loss	No	No
A8	chr2	223339286	225400358	SGPP2	Loss	No	No
A8	chr2	225422375	222433505	CUL3	Loss	No	No
A8	chr2	225634927	225796385	DOCK10	Loss	Yes	No
A8	chr2	230633331	230744844	TRIP12	Loss	No	No
A8	chr2	230787206	231266522	FBXO36	Loss	No	No
A8	chr2	231090444	231176310	SP110	Loss	No	No
A8	chr2	234396884	234628333	USP40	Loss	No	No
A8	chr2	237240001	237406130	IQCA1	Loss	No	No
A8	chr2	238267848	238305490	COL6A3	Loss	Yes	No
A8	chr3	10861149	12475855	SLC6A11	Loss	No	No
A8	chr3	16535231	17550097	RFTN1	Loss	No	No
A8	chr3	20167396	23848912	KAT2B	Loss	No	No
A8	chr3	35723205	35778854	ARPP21	Loss	No	No
A8	chr3	37821392	38835501	ITGA9	Loss	No	Yes

A8	chr3	39453764	38988398	RPSA	Loss	No	No
A8	chr3	45744929	45877276	SACM1L	Loss	No	No
A8	chr3	51393837	56469175	DOCK3	Loss	No	No
A8	chr3	53535641	53844325	CACNA1D	Loss	No	No
A8	chr3	54798244	60522712	CACNA2D3	Loss	No	No
A8	chr3	58810196	62751659	C3orf67	Loss	No	No
A8	chr3	64526770	64672644	ADAMTS9	Loss	No	No
A8	chr3	65349139	65607763	MAGI1	Loss	Yes	No
A8	chr4	21950193	17844002	KCNIP4	Loss	No	No
A8	chr4	25270765	25420120	PI4K2B	Loss	No	No
A8	chr5	45695770	50130849	HCN1	Loss	Yes	No
A8	chr5	54619959	54718810	SKIV2L2	Loss	No	No
A8	chr5	56152426	56209816	MAP3K1	Loss	No	No
A8	chr5	58271483	59481514	PDE4D	Loss	No	No
A8	chr5	61781245	66465423	IPO11	Loss	No	No
A8	chr5	72311452	72383580	FCHO2	Loss	No	No
A8	chr5	73045661	72875200	ARHGEF28	Loss	No	No
A8	chr5	75858220	75998415	IQGAP2	Loss	No	No
A8	chr5	80338695	79952350	RASGRF2	Loss	No	No
A8	chr5	94803569	94882855	TTC37	Loss	No	No
A8	chr5	94927104	96062563	ARSK	Loss	No	No
A8	chr5	95224789	94786144	ELL2	Loss	No	No
A8	chr5	96112107	96139646	ERAP1	Loss	No	No
A8	chr5	96314841	96363518	LNPEP	Loss	No	No
A8	chr5	108133824	110820748	FER	Loss	No	No
A8	chr5	108719103	110407759	PJA2	Loss	No	No
A8	chr5	112136975	111643187	APC	Loss	Yes	No
A8	chr5	112850968	112929087	YTHDC2	Loss	No	No
A8	chr5	118451852	122754209	DMXL1	Loss	No	No
A8	chr5	118809602	118792063	HSD17B4	Loss	No	No
A8	chr5	126635687	126791299	MEGF10	Loss	No	No
A8	chr5	131055009	129070802	FNIP1	Loss	No	No
A8	chr5	145634505	145888808	RBM27	Loss	No	No
A8	chr5	147582583	147513450	SPINK6	Loss	No	No

A8	chr5	148521571	148431803	ABLIM3	Loss	No	No
A8	chr5	157181011	158753790	LSM11	Loss	No	No
A8	chr5	168149937	168678463	SLIT3	Loss	No	No
A8	chr5	169096291	169508988	DOCK2	Loss	No	No
A8	chr6	62407099	66205500	KHDRBS2	Loss	No	No
A8	chr6	64421062	71246114	PHF3	Loss	No	No
A8	chr6	70589427	71571716	COL19A1	Loss	No	No
A8	chr6	74473298	74530287	CD109	Loss	No	No
A8	chr6	76591405	79787224	MYO6	Loss	No	No
A8	chr6	76633350	76782395	IMPG1	Loss	Yes	No
A8	chr6	80715558	80752244	TTK	Loss	No	No
A8	chr6	83810423	83872638	DOPEY1	Loss	No	No
A8	chr6	85448214	86251761	TBX18	Loss	No	No
A8	chr6	90326277	91281526	ANKRD6	Loss	No	No
A8	chr6	101311939	101127628	ASCC3	Loss	Yes	No
A8	chr6	111214112	123958238	AMD1	Loss	No	No
A8	chr6	111650734	111737675	REV3L	Loss	Yes	No
A8	chr6	112393104	112375608	TUBE1	Loss	No	No
A8	chr6	112435278	112537670	LAMA4	Loss	No	No
A8	chr6	117050725	119149293	KPNA5	Loss	No	No
A8	chr6	118475607	136500279	SLC35F1	Loss	No	No
A8	chr6	127767552	128718833	KIAA0408,SOGA3	Loss	No	No
A8	chr6	131910604	131948812	MED23	Loss	No	No
A8	chr6	132618925	133846392	MOXD1	Loss	No	No
A8	chr6	134349518	135813429	SLC2A12	Loss	No	No
A8	chr6	135287466	135359164	HBS1L	Loss	No	No
A8	chr6	143090688	142917673	HIVEP2	Loss	No	No
A8	chr6	144837364	145167963	UTRN	Loss	No	No
A8	chr6	147527106	147704134	STXBP5	Loss	No	Yes
A8	chr6	152679507	152861156	SYNE1	Loss	Yes	No
A8	chr6	155596749	157743837	CLDN20,TFB1M	Loss	No	No
A8	chr6	159618462	159688958	FNDC1	Loss	Yes	No
A8	chr6	160645736	160888751	SLC22A2	Loss	Yes	No
A8	chr6	160952514	161032753	LPA	Loss	No	No

A8	chr6	161455290	160471684	MAP3K4	Loss	No	No
A8	chr6	165749611	165957056	PDE10A	Loss	No	No
A8	chr6	168265230	168352867	MLLT4	Loss	Yes	No
A8	chr8	2017565	2090322	MYOM2	Loss	Yes	No
A8	chr8	15480588	15615364	TUSC3	Loss	No	No
A8	chr8	21771049	21976578	DOK2	Loss	No	No
A8	chr8	23069628	32622558	TNFRSF10A	Loss	No	No
A8	chr8	23225509	24261601	LOXL2	Loss	No	No
A8	chr8	23708832	24193612	STC1	Loss	No	No
A8	chr8	30915887	31024746	WRN	Loss	No	No
A8	chr9	676889	179075	KANK1	Loss	No	No
A8	chr9	6420911	6504691	UHRF2	Loss	No	No
A8	chr9	18950755	19033256	FAM154A	Loss	No	No
A8	chr9	19378365	22009312	RPS6	Loss	No	No
A8	chr9	26886101	26962085	CAAP1	Loss	No	No
A8	chr9	34485434	33922876	DNAI1	Loss	No	No
A8	chr9	73736093	78804662	TRPM3	Loss	Yes	No
A8	chr9	75263512	75451267	TMC1	Loss	No	No
A8	chr9	77732419	80043952	OSTF1	Loss	No	No
A8	chr9	80880774	82337950	CEP78	Loss	No	No
A8	chr9	88924126	88968145	ZCCHC6	Loss	Yes	No
A8	chr9	90219868	86153348	DAPK1	Loss	No	No
A8	chr9	93606139	94118437	SYK	Loss	No	No
A8	chr9	100127954	100000849	LOC100499484-C9ORF174,CCDC180	Loss	No	No
A8	chr9	100190741	100258405	TDRD7	Loss	No	No
A8	chr9	101763120	102861330	COL15A1	Loss	No	No
A8	chr9	104237607	104324293	TMEM246	Loss	No	No
A8	chr9	106857579	106900446	SMC2	Loss	No	No
A8	chr9	107547676	107575003	ABCA1	Loss	No	No
A8	chr9	107646707	108147783	ABCA1	Loss	No	No
A8	chr9	109701384	111826847	ZNF462	Loss	No	No
A8	chr9	111640274	111678577	IKBKAP	Loss	No	No
A8	chr9	112225576	113312384	PTPN3	Loss	No	No
A8	chr9	113431050	113734500	MUSK	Loss	No	No

A8	chr9	117786251	117853433	TNC	Loss	No	No
A8	chr9	118949432	119144772	PAPPA	Loss	No	No
A8	chr9	123374673	123330666	MEGF9	Loss	No	No
A8	chr9	123852552	123920158	CNTRL	Loss	No	No
A8	chr9	124801550	124622722	TTLL11	Loss	No	No
A8	chr9	124914523	125946577	NDUFA8	Loss	No	No
A8	chr9	125777821	2191408	RABGAP1	Loss	No	No
A8	chrX	37553538	37850570	XK	Loss	No	No
A8	chrX	150868977	152955961	PRRG3	Loss	No	No
A9	chr11	65123263	65363467	TIGD3	Gain	No	No
A9	chr11	65337942	65403840	SSSCA1	Gain	No	No
A9	chr11	65408301	65407766	SIPA1	Gain	No	No
A9	chr11	65480818	65429676	KAT5	Gain	No	No
A9	chr11	66010382	65151172	PACS1	Gain	No	No
A9	chr11	68671190	68854674	MRPL21	Gain	No	No
A9	chr11	69518028	71506300	FGF19	Gain	No	No
A9	chr11	70253403	70281225	CTTN	Gain	No	No
A9	chr11	70331418	70858384	SHANK2	Gain	No	No
A9	chr12	465597	1017200	KDM5A	Gain	No	No
A9	chr12	1904464	2795008	CACNA2D4	Gain	No	No
A9	chr12	2921786	2910522	ITFG2	Gain	No	No
A9	chr12	3310342	3806201	TSPAN9	Gain	No	No
A9	chr12	6421324	7079916	PLEKHG6	Gain	No	No
A9	chr12	6493753	6451283	LTBR	Gain	No	No
A9	chr12	6602223	6640242	MRPL51	Gain	No	No
A9	chr12	6680034	6949018	CHD4	Gain	No	No
A9	chr12	8075416	8203216	SLC2A3	Gain	Yes	No
A9	chr12	9070225	9244025	PHC1	Gain	No	No
A9	chr12	12514139	15652528	LOH12CR1	Gain	No	No
A9	chr12	31106914	31256665	TSPAN11	Gain	No	No
A9	chr12	52695648	52995322	KRT86	Gain	Yes	No
A9	chr12	53342842	53607484	KRT8,KRT18	Gain	Yes	No
A9	chr12	53440809	53433486	LOC283335	Gain	No	No
A9	chr12	53447179	53496128	TENC1,LOC283335	Gain	No	No

A9	chr12	53579170	53693234	ZNF740	Gain	No	No
A9	chr12	53689622	53836517	PFDN5	Gain	No	No
A9	chr12	53875724	53900215	MAP3K12	Gain	No	No
A9	chr12	54791148	54799500	ITGA5	Gain	No	No
A9	chr12	54801180	56106089	ITGA5	Gain	No	No
A9	chr12	54960757	55042149	PDE1B	Gain	No	No
A9	chr12	56521985	56537413	ESYT1	Gain	Yes	No
A9	chr12	56626472	56719238	SLC39A5	Gain	No	No
A9	chr12	56668560	56652143	CS	Gain	No	No
A9	chr12	56811690	56824752	TIMELESS	Gain	No	No
A9	chr12	56915608	57065631	RBMS2	Gain	No	No
A9	chr12	57406161	57606044	TAC3	Gain	No	No
A9	chr12	57642488	57620232	STAC3	Gain	No	No
A9	chr12	57647547	57704246	R3HDM2	Gain	Yes	No
A9	chr12	57857447	57873207	GLI1	Gain	Yes	No
A9	chr12	57917811	57922792	MBD6	Gain	No	No
A9	chr12	57943846	57995131	KIF5A	Gain	No	No
A9	chr12	58021400	58015686	B4GALNT1	Gain	No	No
A9	chr12	58150711	58168550	9-Mar	Gain	No	No
A9	chr12	58162350	58114301	METTL1	Gain	No	No
A9	chr12	58191159	57941114	TSFM,AVIL	Gain	No	No
A9	chr12	58217686	58351052	CTDSP2	Gain	No	No
A9	chr12	76844630	77243250	OSBPL8	Gain	No	No
A9	chr12	77252495	80014987	CSRP2	Gain	No	No
A9	chr12	96273421	96413018	CCDC38	Gain	No	No
A9	chr14	20837502	20864108	TEP1	Gain	No	No
A9	chr14	21538418	21512392	NDRG2	Gain	No	No
A9	chr14	23851198	23876817	MYH6	Gain	Yes	No
A9	chr14	24000689	24026513	THTPA,ZFHX2	Gain	No	No
A9	chr14	24674925	24657629	TSSK4	Gain	No	No
A9	chr14	24702684	24686429	GMPR2	Gain	No	No
A9	chr14	24718319	24732416	TGM1	Gain	No	No
A9	chr14	30045686	31205033	PRKD1	Gain	No	No
A9	chr14	31050281	31091668	G2E3	Gain	Yes	No

A9	chr14	31364867	31495607	STRN3	Gain	No	No
A9	chr14	31922480	35099366	DTD2	Gain	No	No
A9	chr14	33408458	33684793	NPAS3	Gain	No	No
A9	chr14	35183640	35409702	CFL2	Gain	No	No
A9	chr14	36017713	36278350	RALGAPA1	Gain	No	No
A9	chr14	36942493	39606177	SFTA3	Gain	No	No
A9	chr14	36988189	39734625	NKX2-1	Gain	No	No
A9	chr14	37149866	37641865	SLC25A21	Gain	No	No
A9	chr14	39900767	45722605	FBXO33	Gain	No	No
A9	chr14	47314968	47687393	MDGA2	Gain	Yes	No
A9	chr14	68008550	68044916	PLEKHH1	Gain	No	No
A9	chr14	73706480	73963383	PAPLN	Gain	No	No
A9	chr14	74753403	74826711	ABCD4	Gain	No	No
A9	chr14	74968143	75352337	LTBP2	Gain	No	No
A9	chr14	89212528	91196540	EML5	Gain	No	No
A9	chr14	91626609	91526993	C14orf159	Gain	No	No
A9	chr14	92792211	92958584	SLC24A4	Gain	No	No
A9	chr14	94203586	93720136	PRIMA1	Gain	No	No
A9	chr14	94473356	94546093	LINC00521	Gain	No	No
A9	chr14	95602959	96011055	DICER1	Gain	No	No
A9	chr14	105611266	105624100	JAG2	Gain	No	No
A9	chr14	105677458	105246553	BRF1	Gain	Yes	No
A9	chr14	105833549	105859672	PACS2	Gain	No	No
A9	chr14	105905008	105935835	MTA1	Gain	Yes	No
A9	chr17	262938	2405625	C17orf97	Gain	No	No
A9	chr17	295626	1420182	FAM101B	Gain	No	No
A9	chr17	910404	5286863	ABR	Gain	No	No
A9	chr17	1012173	651272	ABR	Gain	No	No
A9	chr17	1395706	1549083	MYO1C	Gain	No	No
A9	chr17	1646129	1619927	SERPINF2	Gain	No	No
A9	chr17	1746096	2304258	RPA1	Gain	No	No
A9	chr17	2233572	3402700	TSR1	Gain	No	No
A9	chr17	3417886	3495677	TRPV3	Gain	Yes	No
A9	chr17	3518630	4852381	SHPK	Gain	No	No

A9	chr17	3571781	3629992	TAX1BP3,P2RX5-TAX1BP3	Gain	No	No
A9	chr17	3576521	3599698	P2RX5,P2RX5-TAX1BP3	Gain	No	No
A9	chr17	3717615	3794037	C17orf85	Gain	No	No
A9	chr17	3785550	3831621	CAMKK1	Gain	No	No
A9	chr17	4053184	4167274	CYB5D2	Gain	No	No
A9	chr17	4416541	4462733	SPNS2	Gain	No	No
A9	chr17	4442190	4458681	SPNS2	Gain	No	No
A9	chr17	4608493	4649414	LOC101559451	Gain	No	No
A9	chr17	4621980	4848517	ARRB2	Gain	No	No
A9	chr17	4637877	4619486	CXCL16	Gain	No	No
A9	chr17	4855122	4890960	ENO3	Gain	No	No
A9	chr17	4923243	5138155	KIF1C	Gain	No	No
A9	chr17	5126611	6554954	SCIMP,LOC100130950	Gain	No	No
A9	chr17	5290027	5323059	NUP88	Gain	No	No
A9	chr17	5322960	5372380	NUP88	Gain	No	No
A9	chr17	5404718	5418837	LOC728392	Gain	No	No
A9	chr17	5983690	6354385	WSCD1	Gain	No	No
A9	chr17	6428675	7606820	PITPNM3	Gain	No	No
A9	chr17	6493098	6616740	KIAA0753	Gain	No	No
A9	chr17	6918380	6915653	C17orf49,RNASEK-C17orf49	Gain	No	No
A9	chr17	7094585	7120928	DLG4	Gain	No	No
A9	chr17	7147869	7126698	CTDNEP1	Gain	No	No
A9	chr17	7218950	7292202	NEURL4	Gain	No	No
A9	chr17	7306292	7370646	C17orf61-PLSCR3	Gain	No	No
A9	chr17	7460415	7359260	TNFSF12,TNFSF12-TNFSF13	Gain	No	No
A9	chr17	7533411	7536701	SHBG	Gain	Yes	No
A9	chr17	7623038	7481562	DNAH2	Gain	No	No
A9	chr17	7942357	7991021	ALOX15B	Gain	No	No
A9	chr17	8021803	8113944	ALOXE3	Gain	No	No
A9	chr17	8131820	8222712	CTC1	Gain	No	No
A9	chr17	8339169	8274858	NDEL1	Gain	Yes	No
A9	chr17	8366637	8661877	NDEL1	Gain	Yes	No
A9	chr17	8380193	8534079	MYH10	Gain	No	No
A9	chr17	8784221	8814824	PIK3R5	Gain	No	No

A9	chr17	37226108	37228844	PLXDC1,LOC100131347	Gain	No	No
A9	chr17	37420427	37557909	FBXL20	Gain	No	No
A9	chr17	37809733	37791979	STARD3	Gain	No	No
A9	chr17	37829303	37826728	PGAP3	Gain	No	No
A9	chr17	37856230	37884915	ERBB2	Gain	No	Yes
A9	chr17	37898504	37882814	GRB7	Gain	No	No
A9	chr20	5086833	5100647	TMEM230	Gain	No	No
A9	chr20	7990846	9624987	TMX4	Gain	No	No
A9	chr20	22548431	25038818	LINC00261	Gain	No	No
A9	chr20	24943579	25058501	APMAP	Gain	No	No
A9	chr20	25436308	25281520	NINL	Gain	Yes	No
A9	chr20	40877326	41514572	PTPRT	Gain	No	No
A9	chr20	46288440	46386207	SULF2	Gain	No	No
A9	chr20	47254248	48884200	PREX1	Gain	No	No
A9	chr20	48260062	48732496	B4GALT5	Gain	Yes	No
A9	chr20	49253117	49626901	FAM65C	Gain	No	No
A9	chr20	49492533	53260117	BCAS4	Gain	No	No
A9	chr20	50221355	50384908	ATP9A	Gain	No	No
A9	chr20	52188259	52789638	ZNF217	Gain	No	No
A9	chr20	55066547	55790922	RTFDC1	Gain	No	No
A9	chr20	55967452	57016139	RBM38	Gain	No	No
A9	chr20	56185204	56188386	ZBP1	Gain	No	No
A9	chr20	57425584	58422766	GNAS,GNAS-AS1	Gain	No	No
A9	chr20	57607274	57601709	ATP5E,SLMO2-ATP5E	Gain	No	No
A9	chr20	57611525	57605484	SLMO2,SLMO2-ATP5E	Gain	No	No
A9	chr20	58441549	58507209	SYCP2	Gain	No	No
A9	chr20	59829881	60509278	CDH4	Gain	No	No
A9	chr20	60572605	62507228	TAF4	Gain	No	No
A9	chr20	60831112	60835181	OSBPL2	Gain	No	No
A9	chr20	60928065	61575044	LAMA5	Gain	Yes	No
A9	chr20	61833638	61991051	YTHDF1	Gain	No	No
A9	chr20	61917331	61961013	ARFGAP1	Gain	Yes	No
A9	chr20	62037541	62103993	KCNQ2	Gain	No	No
A9	chr20	62152636	62178857	PPDPF	Gain	No	No

A9	chr20	62187006	62374143	C20orf195	Gain	No	No
A9	chr20	62326680	62367494	RTEL1,RTEL1-TNFRSF6B	Gain	No	No
A9	chr20	62517368	62664453	TPD52L2	Gain	No	No
A9	chr20	62550777	62708363	DNAJC5	Gain	No	No
A9	chr20	62714732	62899363	OPRL1,C20orf201	Gain	No	No
A9	chr21	32496839	32639299	TIAM1	Gain	Yes	No
A9	chr21	34896315	34893322	GART	Gain	No	No
A9	chr21	34922967	34948199	SON	Gain	No	No
A9	chr21	35229039	36421255	ITSN1	Gain	No	No
A9	chr21	47660747	47648738	MCM3AP,MCM3AP-AS1	Gain	Yes	No
A9	chr22	39095807	39448358	JOSD1	Gain	No	No
A9	chr7	1477924	1544018	MICALL2	Gain	No	No
A9	chr7	73653252	73954298	RFC2	Gain	No	No
A9	chr7	100274315	100320466	GNB2	Gain	No	No
A9	chr8	144875145	144897549	SCRIB	Gain	No	No
A9	chr8	145003270	145066776	PLEC	Gain	No	No
A9	chr8	145577894	145545071	TMEM249	Gain	No	No
A9	chr8	145579087	145537717	TMEM249	Gain	No	No
A9	chr9	131006999	131051268	DNM1	Gain	No	No
A9	chr9	132614818	132591820	USP20	Gain	No	No
A9	chr9	139393350	139411837	NOTCH1	Gain	No	No
A9	chr9	139888217	139959637	C9orf142	Gain	Yes	No
A9	chr10	69692353	69832943	HERC4	Loss	No	No
A9	chr10	91465050	91528148	KIF20B	Loss	Yes	No
A9	chr10	96313882	95987208	HELLS	Loss	No	No
A9	chr10	102676326	102716294	FAM178A	Loss	No	No
A9	chr10	118084458	118661468	CCDC172	Loss	No	No
A9	chr18	29511291	30992074	TRAPPC8	Loss	No	No
A9	chr20	3677731	3944671	SIGLEC1	Loss	No	No
A9	chr20	13251150	13765579	ISM1	Loss	No	No
A9	chr20	13765467	13971265	ESF1	Loss	Yes	No
A9	chr20	15210585	16025284	MACROD2	Loss	No	No
A9	chr20	37601201	37662960	DHX35	Loss	Yes	No
A9	chr20	42225074	42355642	IFT52	Loss	No	No

A9	chr21	14417483	16340847	ANKRD30BP2	Loss	No	No
A9	chr21	15003261	15579254	POTED	Loss	No	No
A9	chr21	17135209	17250310	USP25	Loss	No	No
A9	chr21	17911408	18937929	LINC00478	Loss	No	No
A9	chr3	160222745	160254628	KPNA4	Loss	No	No
A9	chr8	1791518	6679560	ARHGEF10	Loss	No	No
A9	chr8	6582390	6914259	AGPAT5	Loss	No	No
A9	chr8	10383055	10697409	PRSS55	Loss	No	No
A9	chr8	10753656	11420619	XKR6	Loss	No	No
A9	chr8	12863710	15615364	KIAA1456	Loss	No	No
A9	chr8	13251061	12990809	DLC1	Loss	No	No
A9	chr8	17089935	17143936	CNOT7	Loss	No	No
A9	chr8	17396285	17421220	SLC7A2	Loss	No	No
A9	chr8	17916343	17942507	ASAH1	Loss	No	No
A9	chr8	22136853	22785421	PIWIL2	Loss	No	No
A9	chr8	22332466	22398657	PPP3CC	Loss	No	No
A9	chr8	22463248	22451810	CCAR2	Loss	No	No
A9	chr8	22938497	24261601	LOC286059	Loss	No	No
A9	chr8	22995700	22974950	TNFRSF10D	Loss	No	No
A9	chr8	23147378	23307510	R3HCC1	Loss	No	No
A9	chr8	23425834	24193612	SLC25A37	Loss	No	No
A9	chr8	29989186	30609035	LEPROTL1	Loss	No	No
A9	chr9	368017	463687	DOCK8	Loss	Yes	No
A9	chr9	2028986	2186228	SMARCA2	Loss	No	No
A9	chr9	2729445	2838517	KCNV2	Loss	No	No
A9	chr9	3228846	3395596	RFX3	Loss	No	No
A9	chr9	4544566	4666256	SLC1A1	Loss	No	No
A9	chr9	5021962	5126446	JAK2	Loss	Yes	No
A9	chr9	5357966	5773080	PLGRKT	Loss	No	No
A9	chr9	5522532	6015640	PDCD1LG2	Loss	No	Yes
A9	chr9	5768969	6592223	KIAA1432	Loss	No	No
A9	chr9	16276228	17487064	C9orf92	Loss	No	No
A9	chr9	38411387	38616041	IGFBPL1	Loss	No	No
A9	chr9	39461676	39178357	LOC653501	Loss	No	No

A9	chr9	71951128	71789348	FAM189A2	Loss	Yes	No
A9	chr9	72879219	74365301	SMC5	Loss	No	No
A9	chr9	75303624	75445598	TMC1	Loss	No	No
A9	chr9	77286704	80537261	RORB	Loss	No	No
A9	chr9	77343154	77473664	TRPM6	Loss	No	No
A9	chr9	80850990	80881983	CEP78	Loss	No	No
A9	chr9	84560086	85640836	SPATA31D3	Loss	No	No
A9	chr9	86457151	86530593	KIF27	Loss	No	No
A9	chr9	86894179	86243874	SLC28A3	Loss	No	No
A9	chr9	90252857	86004623	DAPK1	Loss	No	No
A9	chr9	91930084	91972480	CKS2	Loss	No	No

Supplementary Table 8. Signaling pathways with significant enrichment of mutated genes

Functions	-log(p-value)	No. of samples	Mutated genes
NF-κB Signaling	3.88	6	KRAS,IRAK3,EP300,TGFBR2,TLR4,LCK,NTRK3,PLCG2,KDR,PIK3R6,PDGFRA,INSR,EGFR,FOXO
p70S6K Signaling	2.62	6	CD79B,F2R,PLCG2,PIK3R6,LYN,KRAS,PLCL1,PRKD3,EGFR
JAK/Stat Signaling	2.45	4	STAT4,SOCS6,PIK3R6,KRAS,JAK2,PRKD3
Melanocyte Development and Pigmentation Signaling	2.42	5	ADCY2,MITF,PLCG2,PIK3R6,KIT,KRAS,EP300
FcγRIIB Signaling in B Lymphocytes	2.35	4	CD79B,PLCG2,PIK3R6,LYN,KRAS
cell morphogenesis	2.35	6	NRP2, EGR2, NRXN3, BAIAP2, STXBP1, DSCAML1, NRXN1, NR2E1, SLIT2, UNC5A, LHX2, FOXG1, GBX2, RELN, JAK2, CDH23
Axonal Guidance Signaling	2.26	7	PAPPA2,NRP2,UNC5A,NFATC3,KRAS,EPHA3,PLXNA2,SLIT2,PLXND1,LIMK1,HERC2,MAG,NTRK3,PLCG2,BAIAP2,PIK3R6,NFATC2,PLCL1,PRKD3
Chromatin modification	2.24	6	SMARCAD1, ENY2, EZH1, TTF1, SMYD1, CTR9, PHF17, EP300, CHD10, KDM5B, NSD1, SMARCA4, MLL2
Hereditary Breast Cancer Signaling	2.2	8	TP53,XPC,PIK3R6,KRAS,ATR,SMARCA4,RFC3,EP300
Netrin Signaling	2.19	4	UNC5A,NFATC3,RYR3,NFATC2,RYR1
Cell Cycle and DNA Damage Checkpoint Regulation	2.18	6	TP53, ATR, ARF, CDKN2A, EP300, FOXG1, GAS7, HERC2, INHBA, INSR, MITF, MYO16, RFC3, ORC5, P16INK4, PIK3R6 PPP1R9B, SMARCA4
PTEN Signaling	2.16	6	TGFBR2,NTRK3,KDR,PDGFRA,KRAS,INSR,FOXG1,EGFR
Hepatic Cholestasis	2.16	4	TLR4,ADCY2,ABCB4,ABCB11,SLCO1A2,INSR,IRAK3,PRKD3,SLCO1B3

Cell adhesion	2.16	5	NRP2, PPFIA2, NELL2, LMO7, DSCAML1, CBLL1, PNN, PCDHGA1, IGSF11, LAMB3, CDH20, COL7A1, FAT3, FAT4, COL6A3, COL6A2, CNTNAP2, CDH23, FLRT3, EGFR, FLRT2, COL4A3, MAG, PTPRF, NRXN3, CNTNAP5, SELL, HSPG2, STXBP1, PCDH15, ACTN2, MFGE8, NRXN1, PCDH17, EMILIN2, PCDH18, AMIGO2, FREM2, ITGA8, TROAP, RELN, COL24A1, ABL2, CDH10, CHL1
Synaptic Long Term Depression	2.14	5	PLCG2,GRID2,RYR3,LYN,RYR1,KRAS,PLCL1,PRKD3,NPR2
G Protein Signaling Mediated by Tubby	2.12	2	LCK,PLCG2,JAK2,INSR
Leptin Signaling in Obesity	2.1	3	ADCY2,PLCG2,PDE3A,PIK3R6,JAK2,PLCL1
B Cell Receptor Signaling	2.08	4	EBF1,CD79B,NFATC3,PLCG2,PIK3R6,LYN,NFATC2,KRAS,EP300

Supplementary Table 9. Clinical information of NSCLC patients

Samples	Gender	Age	Tumor type	Stage	
A1	f	75	ADC	IIA	99
A2	m	47	ADC	IIB	103
A3	m	61	ADC	IIA	502
A4	f	57	SCC	IIIB	543
A5	m	36	ADC	IIIA	541
A6	m	53	ADC	IB	503
A7	m	68	ADC	IIA	544
A8	f	75	ADC	IIB	546
A9	m	53	ADC	IIB	550
B1	m	45	ADC	IIIA	
B2	f	73	ADC	IIB	
B3	m	67	ADC	IIIB	
B4	f	60	ADC	IIIA	
B5	f	70	ADC	IIA	
B6	m	60	ADC	IIB	
B7	f	55	ADC	IIA	
B8	f	45	ADC	IIA	
B9	f	45	ADC	IIA	
B10	f	66	ADC	IIA	
B11	f	78	ADC	IIA	
B12	f	73	ADC	IIA	
B13	f	70	ADC	IIA	
B14	f	67	ADC	IIA	
B15	m	68	ADC	IIIA	
B16	f	66	ADC	I	
B17	m	50	ADC	IIA	
B18	f	55	ADC	IIA	
B19	m	68	ADC	IIA	
B20	f	61	ADC	IIA	
B21	m	60	ADC	IIA	
B22	m	73	ADC	IIB	
B23	m	46	ADC	IIIA	
B24	f	46	ADC	IIB	
B25	f	58	ADC	IIB	
B26	f	72	ADC	IIIB	
B27	m	60	ADC	IIIB	
B28	m	62	ADC	IIIA	
B29	m	66	SCC	IIIA	
B30	m	60	ADC	IIIA	
B31	m	60	SCC	IIIA	
B32	m	64	ADC	IIIA	
B33	m	43	SCC	IIIB	
B34	f	68	ADC	I	
B35	m	78	SCC	IIIA	
B36	m	40	ADC	IIIA	
B37	f	62	ADC	IIIA	
B38	m	54	ADC	I	
B39	m	50	ADC	IIA	
B40	m	71	ADC	IIIA	

B41	f	62	ADC	IIA
B42	m	51	ADC	IIA
B43	m	47	ADC	IIIA
B44	m	74	SCC	IIB
B45	m	54	ADC	IIA
B46	f	71	ADC	IIIA
B47	m	62	SCC	IIB
B48	m	75	ADC	IIA
B49	m	50	SCC	IIA
B50	m	70	ADC	IIIA
B51	f	70	SCC	IIB
B52	m	60	ADC	IIB
B53	m	45	SCC	I
B54	f	55	SCC	I
B55	f	68	ADC	IIB
B56	m	55	ADC	IIIA
B57	m	62	SCC	IIB
B58	m	55	SCC	IIB
B59	m	54	SCC	I
B60	m	62	ADC	I
B61	m	70	ADC	IIA
B62	m	65	SCC	IIB
B63	m	63	ADC	IIB
B64	m	69	SCC	IIA
B65	f	65	ADC	IIA
B66	f	62	ADC	IIB
B67	m	63	SCC	IIA
B68	m	61	ADC	IIB
B69	m	68	SCC	IIIB
B70	f	57	SCC	IIA
B71	f	74	SCC	IIA
B72	m	52	SCC	IIIA
B73	f	71	ADC	IIB
B74	m	52	ADC	IIA
B75	m	60	SCC	IIB
B76	m	62	ADC	IIB
B77	m	53	ADC	IIB
B78	m	65	ADC	IIB
B79	f	60	ADC	IIB
B80	m	57	ADC	IIB
B81	m	52	SCC	IB
B82	f	44	ADC	IIB
B83	m	71	SCC	IA
B84	m	56	ADC	IIIB
B85	m	56	ADC	IIA
B86	m	46	ADC	IIIB
B87	m	56	ADC	I
B88	m	77	ADC	IIA
B89	m	44	ADC	IIA
B90	m	58	ADC	IIA
B91	f	60	ADC	IIA

B92	m	56	ADC	IIIB
B93	m	55	ADC	IIIA
B94	m	62	ADC	IIA
B95	m	50	ADC	IIA
B96	f	75	ADC	IIA

Supplementary Table 10. Primers to amplify exons of MLL2

Long-range PCR	Primers (exoms covered)	Sequences (5'-3')
1	MLL2-E1-8-F	CCAGAGTGTGAGAAGAGCGTATG
1	MLL2-E1-8-R	CCACCTTAGGGCTCTCCTCTC
2	MLL2-E9-10-F	GCAGGTTGGAAGAACTGACATTG
2	MLL2-E9-10-R	TGCTGAAGGAGTGGCGAACAC
3	MLL2-E11-F	TATCTCCCTTGCTTGGAGAGC
3	MLL2-E11-R	TGAGTAACCATGAATCTGGGC
4	MLL2-E12-18-F	TGGGACTCCTGGGCTTATTAC
4	MLL2-E12-18-R	TGCCTGATGAAAGGAACATTG
5	MLL2-E19-23-F	GGTTGAAACTTGCAGTTCTGG
5	MLL2-E19-23-R	CCCATCAAATAACTTGCCAGC
6	MLL2-E24-30-F	GCTGGCAAGTTATTTGATGGG
6	MLL2-E24-30-R	TCAGTCTTACGGGCTATGTCG
7	MLL2-E31-32-F	TAAGGCTGTGTCCCATATCCC
7	MLL2-E31-32-R	ACATCCATAGAGGAAGGCGTG
8	MLL2-E33-34-F	AACGGCAGGTAAGTTGACACC
8	MLL2-E33-34-R	AGCCAAAGTTCTTTGTGTCCC
9	MLL2-E35-38-F	CAGGATGTTGAAGGGAATCG
9	MLL2-E35-38-R	TCTGCTGTTTCCGGACCTAAC
10	MLL2-E39-F	GGTGCTAGAGGAGCAGATTGG
10	MLL2-E39-R	TCTCTTCTGTCTGACCCAGGC
11	MLL2-E40-42-F	GAGTGAGCCTGGGTCAGACAG
11	MLL2-E40-42-R	TAAACACACAGGACAGCAGGC
12	MLL2-E43-47-F	CAAACTGGTAGGTGGGAGGAC
12	MLL2-E40-47-R	CTTGCCTCCCAAAGCACTG
13	MLL2-48-F	CTAGGGCAAAGAATGTGGAGG
13	MLL2-48-R	GGCTCAAACACTTTCACTGGG
14	MLL2-E49-50-F	CATGATAGGGAGGCTTGAAGG
14	MLL2-E49-50-R	TCCTAAATCCTCATAATGGGACC
15	MLL2-E51-54-F	CAGAGGAGGTGGGTGGTATG
15	MLL2-E51-54-R	TGGCTGCTACCTCTCTTCCC
